# *Astragalus* polysaccharide promotes the regeneration of intestinal stem cells through HIF‐1 signalling pathway

**DOI:** 10.1111/jcmm.18058

**Published:** 2023-12-14

**Authors:** Qianqian Ding, Xianpeng Zu, Wei Chen, Jiayun Xin, Xike Xu, Yanhui Lv, Xintong Wei, Jie Wang, Yanping Wei, Zhanhong Li, Jianming Cai, Jicong Du, Weidong Zhang

**Affiliations:** ^1^ School of Pharmacy Anhui University of Traditional Chinese Medicine Hefei China; ^2^ School of Pharmacy Naval Medical University Shanghai China; ^3^ School of Pharmacy Shandong University of Traditional Chinese Medicine Jinan China; ^4^ School of Pharmacy Guangdong Pharmaceutical University Guangzhou China; ^5^ Faculty of Naval Medicine Naval Medicine University Shanghai China

**Keywords:** *Astragalus* polysaccharide, HIF‐1 signalling, intestinal injury, intestinal stem cells, ionizing radiation

## Abstract

Ionizing radiation (IR)‐induced intestinal injury is usually accompanied by high lethality. Intestinal stem cells (ISCs) are critical and responsible for the regeneration of the damaged intestine. *Astragalus* polysaccharide (APS), one of the main active ingredients of *Astragalus membranaceus* (AM), has a variety of biological functions. This study was aimed to investigate the potential effects of APS on IR‐induced intestine injury via promoting the regeneration of ISCs. We have established models of IR‐induced intestinal injury and our results showed that APS played great radioprotective effects on the intestine. APS improved the survival rate of irradiated mice, reversed the radiation damage of intestinal tissue, increased the survival rate of intestinal crypts, the number of ISCs and the expression of intestinal tight junction‐related proteins after IR. Moreover, APS promoted the cell viability while inhibited the apoptosis of MODE‐K. Through organoid experiments, we found that APS promoted the regeneration of ISCs. Remarkably, the results of network pharmacology, RNA sequencing and RT‐PCR assays showed that APS significantly upregulated the HIF‐1 signalling pathway, and HIF‐1 inhibitor destroyed the radioprotection of APS. Our findings suggested that APS promotes the regeneration of ISCs through HIF‐1 signalling pathway, and it may be an effective radioprotective agent for IR‐induced intestinal injury.

## 1 INTRODUCTION

The intestinal tissue is one of the most rapidly renewing tissues which is renewed approximately every 5 days.^1^ Intestinal stem cells (ISCs), located at the base of the crypts, continuously differentiate, mature and migrate upwards to replenish the intestinal epithelial cells.^2^ ISCs are critical for intestinal homeostasis and responsible for the regeneration of the damaged intestine. Researches show that ISCs are marked by the expression of several markers such as Lgr5, OLFM4 and so on.^3^ However, ISCs is extremely radiosensitive. High doses of ionizing radiation (IR), such as nuclear accidents and local peritoneal abdominopelvic radiation therapy, can cause the death of ISCs and severe intestinal radiation damage.^4^ The intestinal radiation damage may lead to microflora translocation, bacteremia and electrolyte disturbances.^5^, ^6^ Although great progress has been made in the radioprotection, there is still no ideal treatment for intestinal injury caused by high‐dose IR.^7^ How to promote the regeneration of ISCs to alleviate IR‐induced intestinal damage is still a key problem needing urgent attention in the radioprotection field. Here we established mice and intestinal organoid models to explore the mechanism of ISCs regeneration after IR, and try to find novel potential intestinal radioprotection agents.

Recently, radioprotective agents targeting hypoxia‐inducible factor (HIF) have made great progress. Under normoxic conditions, HIF‐α is hydroxylated in vivo with prolyl hydroxylase (PHD) and then binds to tumour suppressor protein (von Hippel–Lindau protein, pVHL) for eventual degradation. However, under anoxic conditions, the HIF‐α transferred to the nucleus to form a heterodimer with the HIF‐β. Then the heterodimer interacts with a specific DNA sequence called HIF‐reactive elements (HREs). By binding to HRE, HIF activates or inhibits gene expression. Researches revealed that Hypoxia signalling pathway is critical to intestinal homeostasis.^8^, ^9^ The intestine naturally exists in a steep physiologic hypoxia gradient, and HIF regulates critical genes required for intestinal barrier function, such as intestinal trefoil factor and MDR1.^10^ Our group has previously reported that FG‐4592, a novel PHD inhibitor, could mitigate IR‐induced intestinal damage and promote ISCs recovery by upregulating TLR4.^11^ Thus, drugs that activate the HIF‐1 signalling pathway could be great effective radioprotective agents.

As one of the main active ingredients of *Astragalus membranaceus* (AM), *Astragalus* polysaccharide (APS) has a variety of biological functions. Previous studies have shown that APS has a variety of biological activities including antioxidant, antitumor, immunomodulatory and antiradiation.^12^ Zhang YM et al. reported APS inhibited IR‐induced bystander effects by modulating apoptosis of bone mesenchymal stem cells.^13^ Liu Y et al. reported that APS protected against IR‐induced liver damage.^14^ However, the radioprotective effects and mechanism of APS on the regeneration of ISCs have not been reported. In this study, we found that APS could promote the regeneration of ISCs through HIF‐1 signalling pathway, resulting in mitigating IR‐induced intestinal injury and improving mice survival rate. In addition, APS might be a potentially effective intestinal radioprotective agent.

## 2 MATERIALS AND METHODS

### 2.1 Chemicals and reagents

APS was purchased from Shanghai Yuanye Biological Technology Company (Shanghai, China). Normal saline (NS) was obtained from ChangHai Hospital (Shanghai, China) and PBS buffer was purchased from Hyclone company (New York, USA). RPMI 1640 medium and fetal bovine serum (FBS) were provided by Gibco (New York, USA). IntestiCult™ Organoid Growth Medium (06005) and Matrigel (356231) were purchased from Stem Cell Technologies (Canada). FITC‐dextran was purchased from Sigma‐Aldrich (Saint Louis, USA). 2‐Methoxyestradiol (2‐MeOE2) was supplied by Selleck Chemicals (Houston, USA). The apoptosis detection kit (FA101) was purchased from Transgen (Beijing, China). The PCR kit (RR036A and RR420A) was purchased from TAKARA (Japan). The CCK8 kit (CK04) was obtained from Donjindo (Osaka, Japan). The cytometric bead array (CBA) (740446) was purchased from Biolegend (USA). BCL2, BAX, C‐CASPASE3, ZO‐1, Occludin, AKT1, OLFM4, Axin, Hif‐1α, β‐actin and GAPDH antibodies were supplied by Cell Signalling Technology (Massachusetts, USA). The primes were supplied by Shenggong Biotech (Shanghai, China). The list of primers is shown in Table 1.

**TABLE 1:** qRT‐PCR primers for the 11 genes evaluated.

Gene symbol	Forward	Reverse
OLFM4	CAGCCACTTTCCAATTTCACTG	GCTGGACATACTCCTTCACCTTA
Lgr5	CCTACTCGAAGACTTACCCAGT	AGTGGTATAGACAGGTCTGTTGG
Axin2	TGACTCTCCTTCCAGATCCCA	TGCCCACACTAGGCTGACA
YAP1	ACCCTCGTTTTGCCATGAAC	TGTGCTGGGATTGATATTCCGTA
Bmi1	ATCCCCACTTAATGTGTGTCCT	CTTGCTGGTCTCCAAGTAACG
Cd44	CTTGCTGGTCTCCAAGTAACG	CAGTCCGGGAGATACTGTAGC
Hif‐1α	ACCTTCATCGGAAACTCCAAAG	CTGTTAGGCTGGGAAAAGTTAGG
NF‐κb1	ATGGCAGACGATGATCCCTAC	TGTTGACAGTGGTATTTCTGGTG
AKT1	ATGAACGACGTAGCCATTGTG	TTGTAGCCAATAAAGGTGCCAT
Iflng	ACAGCAAGGCGAAAAAGGATG	TGGTGGACCACTCGGATGA
Rela	TGCGATTCCGCTATAAATGCG	ACAAGTTCATGTGGATGAGGC

### 2.2 Irradiation

^60^Co (Naval Medical University, China) was used to establish the total body irradiation (TBI) model. To establish the model of IR‐induced intestinal injury, mice were put into special plastic boxes and then irradiated with 9.5 Gy. Irradiation of MODE‐K cells and the intestinal organoid was performed with the designated dose.

### 2.3 Cell culture and treatment

Mice intestinal epithelial cells (MODE‐K) were purchased from American Type Culture Collection and cultured in RPMI 1640 medium containing 10% FBS, 100 U/mL penicillin and 100 mg/mL streptomycin at 37°C in a 5% CO_2_ humidified chamber. The cells received 50 or 100 μg/mL APS before IR.

### 2.4 Animals and treatment

Male C57BL/6J mice, aged 6–8 weeks old, were purchased from Shanghai Sippe‐Bk Lab Animal Co., Ltd. (Shanghai, China). Lgr5‐EGFP‐IRES‐creERT2 mice, aged 6–8 weeks old, were purchased from Cyagen (Jiangsu, China). All mice were housed under controlled conditions of 20°C–22°C and relative humidity of 50%, a fixed 12 h light/dark cycle. Experimental protocols involving animals were approved by the Laboratory Animal Center of the Naval Medical University in conformance with the National Institute of Health Guide for the Care and Use of Laboratory Animals. Mice were assigned to three groups at random (1) Mice were administered by intragastric with NS (200 μL/mice) for 1 week. (2) Mice were administered by intragastric with NS (200 μL/mice) for 1 week before IR. (3) Mice were administered by intragastric with APS (900 mg/Kg, dissolved in NS) for 1 week before IR. At the end of the experiment, animals were injected intraperitoneally with pentobarbital sodium (1% v/v) for anaesthesia.

### 2.5 Intestinal organoid culture

Ten centimetre small intestine was collected from the anaesthetised mice and placed into cold PBS. Each intestinal segment was cut longitudinally and rinsed in PBS 10 times and cut into 2–3 mm fragments, then transferred to 15 mM EDTA/PBS without Ca^2+^/Mg^2+^, and incubated on the ice at rocking for 2 h. Next, the suspension enriched for crypts was filtered through a 70 μm cell strainer (BD Biosciences, San Diego, CA) to remove the residual villous material. The isolated crypts were centrifuged at 300 g for 3 min to separate crypts from single cells. The final fraction consisted of essentially pure crypts and was used for culture. At last, the pelleted crypts were re‐suspended in complete IntestiCult™ Organoid Growth Medium at the density of 200 crypts per 50 μL. The crypts suspension was then mixed with matrigel and seeded in pre‐warmed 24‐well plates. The organoid was subjected to Organoid Growth Medium containing 100 μg/mL APS before IR, and cultured continuously at 37°C in 5% CO_2_.

### 2.6 Cell viability and flow cytometry analysis

The viability of MODE‐K was assayed by the CCK‐8 kit. Cells were seeded into 96‐well plates at a density of 5 × 10^3^ cells per well. 50 or 100 μg/mL APS were administered when cells were adherent. Then the cells were irradiated at 0, 8 and 10 Gy. Optical density was measured at 450 nm approximately about 48 h after IR.

The apoptosis rate of MODE‐K was determined by using apoptosis detection kit. The cells were seeded into six‐well plates and received 100 μg/mL APS before 12 Gy IR. Following IR, the cells were stained with Annexin V‐fluorescein isothiocyanate (AV‐FITC) and propidium iodide‐phycoerythrin (PI‐PE). The cells were then analysed using flow cytometry (Beckman Cytoflex) according to the manufacturer's instructions.

The levels of inflammatory cytokines in mice serum were detected by CBA kit. In the presence or absence of APS treatment, the serum of mice was collected to evaluate the levels of inflammatory cytokines by CBA kit after 9.5 Gy IR on day 3.5, and then was analysed by flow cytometry (Beckman Cytoflex) according to the manufacturer's instructions.

Lgr5‐EGFP‐IRES‐creERT2 mice were used to detect the number of Lgr5^+^ ISCs by flow cytometry. The mice intestinal crypt cells were obtained (same steps as for the culture of intestinal organoid culture) and resuspended in 1 mL trypsin like‐enzymes together with 50 μL DNase (50 μg/mL). The solution was incubated in a water bath at 32°C for 2 min and added 10 mL of culture medium, then was filtered through a 40 μm strainer (to eliminate clumps and other impurities) and pelleted the single cells at 300 × g for 5 min. Finally, the obtained single cell suspensions were detected by flow cytometry (Beckman Cytoflex) according to the manufacturer's instructions.

### 2.7 Histopathology, immunohistochemistry and immunofluorescence

The small intestine was removed from anaesthetised mice and fixed in a 4% paraformaldehyde solution on day 3.5 after 9.5 Gy IR. haematoxylin and eosin staining was performed to assess the pathologic damage to the small intestine, including villus length and crypt number. Ki67 staining and TUNEL staining (Terminal deoxynucleotidyl transferase dUTP nick‐end labelling) were carried out to detect the proliferation and apoptosis of intestinal crypts. Immunofluorescence was used to analyse the expression of OLFM4, ZO‐1 and Occludin. All experimental protocols were performed as per the manufacturer's instructions.

### 2.8 FITC‐dextran assay for intestinal permeability

C57BL/6 mice were randomly divided into three groups (*n* = 6): Control group, IR group and APS group. Three groups of mice were fasted overnight for 12 h. On day 3.5 after 9.5 Gy IR, FITC‐dextran was dissolved in sterile saline, protected from light and gavaged at 600 mg/kg relative body weight. After 4 h of water fasting, the mice were anaesthetised with sodium pentobarbital, and the blood was collected by removing the eyeballs, and then centrifuged (3000 rpm, 10 min) in a place protected from light for a period of time to obtain the serum. The fluorescence values (excitation wavelength 490 nm, emission wavelength 520 nm) were measured using a BioTek Synergy Neo2 multifunctional fluorometric enzyme standard and quantified according to the calibration curve of serum FITC‐dextran concentration.

### 2.9 Interference with intestinal organoid using small molecule inhibitors

Small intestinal organoids were treated with APS (100 μg/mL) for 2 h, and then with the 2‐Methoxyestradiol (2‐MeOE2) (HIF‐1 inhibitor) (10 μM). After exposure to 6 Gy radiation, the morphology of small intestinal organoids was observed, and to count the budding length, budding rate and surface area of intestinal organoids.

### 2.10 Network pharmacology analysis

The structure of APS compounds was retrieved using the PubChem database (https://pubchem.ncbi.nlm.nih.gov/). The potential targets of APS (*p* > 0.9) were obtained from TargetNet platform (http://targetnet.scbdd.com/) according to the structure of the APS compounds. Based on the keyword “Irradiation‐induced Intestinal Injury”, 778 related genes were identified in the GeneCards database (https://www.genecards.org/).

### 2.11 RNA sequencing and functional enrichment analysis

Mice were grouped into the TBI group and TBI + APS group (*n* = 4). After three and half days of IR, mice small intestine was collected and Trizol (Invitrogen, USA) was used to extract RNA. NanoVue (GE, USA) was used to access RNA purity. Each RNA sample had a ratio of A260:A280 greater than 1.8 and an A260:A230 ratio greater than 2.0 respectively. Sequencing was carried out in OEbiotech (Shanghai, China) using the Illumina HiSeq 2500 system. Correlation testing, principal component analysis and cluster analysis were performed on the clean data, then the data were ready for differential expression gene (DEGs) analysis.

### 2.12 Statistical analysis

Statistical analysis was performed by SPSS 19 software. Two‐tailed Student's *t*‐test was used to analyse the differences between two groups. One‐way anova was employed to analyse the differences among three groups. Data were expressed as means ± the standard errors of means. The difference in survival rates were calculated using Kaplan–Meier analysis. *p* < 0.05 was considered statistically significant.

## 3 RESULTS

### 3.1 APS exhibited radioprotective effects in vivo

**FIGURE 1:**
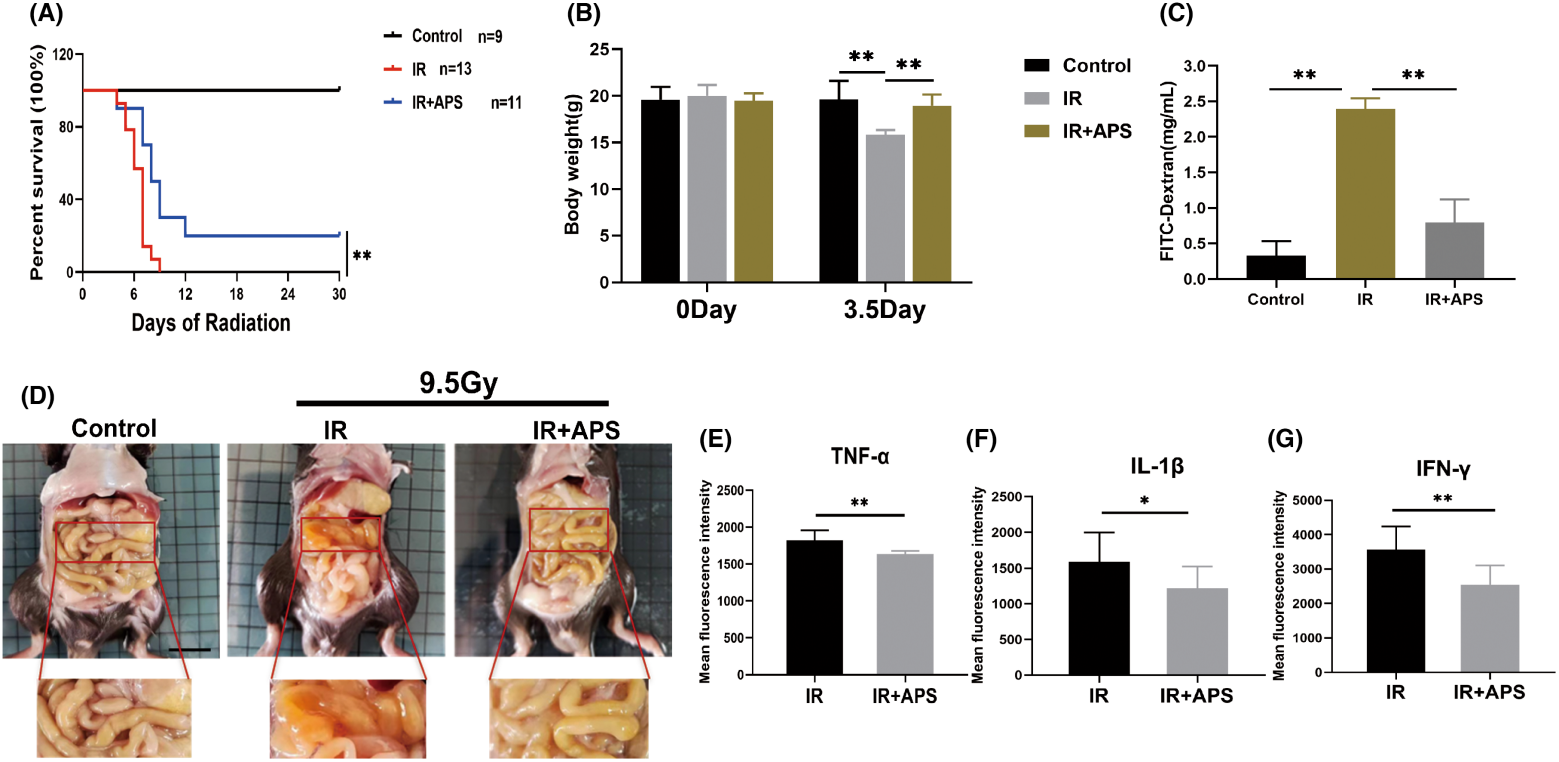
*Astragalus* polysaccharide (APS) exhibited radioprotective effects in vivo. (A) The survival rate of mice exposed to 9.5 Gy TBI. (B) The body weight of mice was recorded on day 1 and day 3.5 day after 9.5 Gy TBI. (C) Levels of FITC‐dextran in serum. (D) Representative images of mice intestine from control, IR + NS or IR + APS group on day 3.5 after 9.5 Gy TBI. (E–G). The levels of TNF‐α, IL‐1β, and IFN‐γ in serum of mice were detected via flow cytometry after 9.5 Gy TBI. The data were presented as mean ± SD. *p < 0.05, **p < 0.01.

To clarify the radioprotection effects of APS in vivo, C57BL/6J mice were given 900 mg/kg APS by intragastric administration 24 h and 2 h before 9.5 Gy TBI. As we can see, APS significantly improved the survival rate of mice after 9.5 Gy TBI (Figure 1A). Meanwhile, weight change of mice pre‐ and post‐IR of 9.5 Gy were recorded, and the result indicated that the body weight of IR + NS group mice was reduced substantially, while APS can save this weight loss (Figure 1B). In addition, we used oral FITC‐dextran to detect intestinal permeability, and found that the amount of FITC‐dextran entering the blood from the intestine increased after 9.5 Gy IR, while the concentration of FITC‐dextran in serum decreased after APS treatment, indicating that IR increased intestinal permeability, leading to increased intestinal leakage, which could be significantly saved by APS (Figure 1C). And then, compared with the mice in control group, the mice in IR + NS group had severe intestinal injury including ulceration and oedema, while APS attenuated IR‐induced intestinal purulent inflammation of mice (Figure 1D). Furthermore, we found that APS reversed the elevation of TNF‐α, IL‐1β, and IFN‐γ induced by IR (Figure 1E–G). Taken together, these results showed that APS exhibited radioprotective effects in vivo.

### 3.2 APS alleviated the IR‐induced intestinal damage in vitro

**FIGURE 2:**
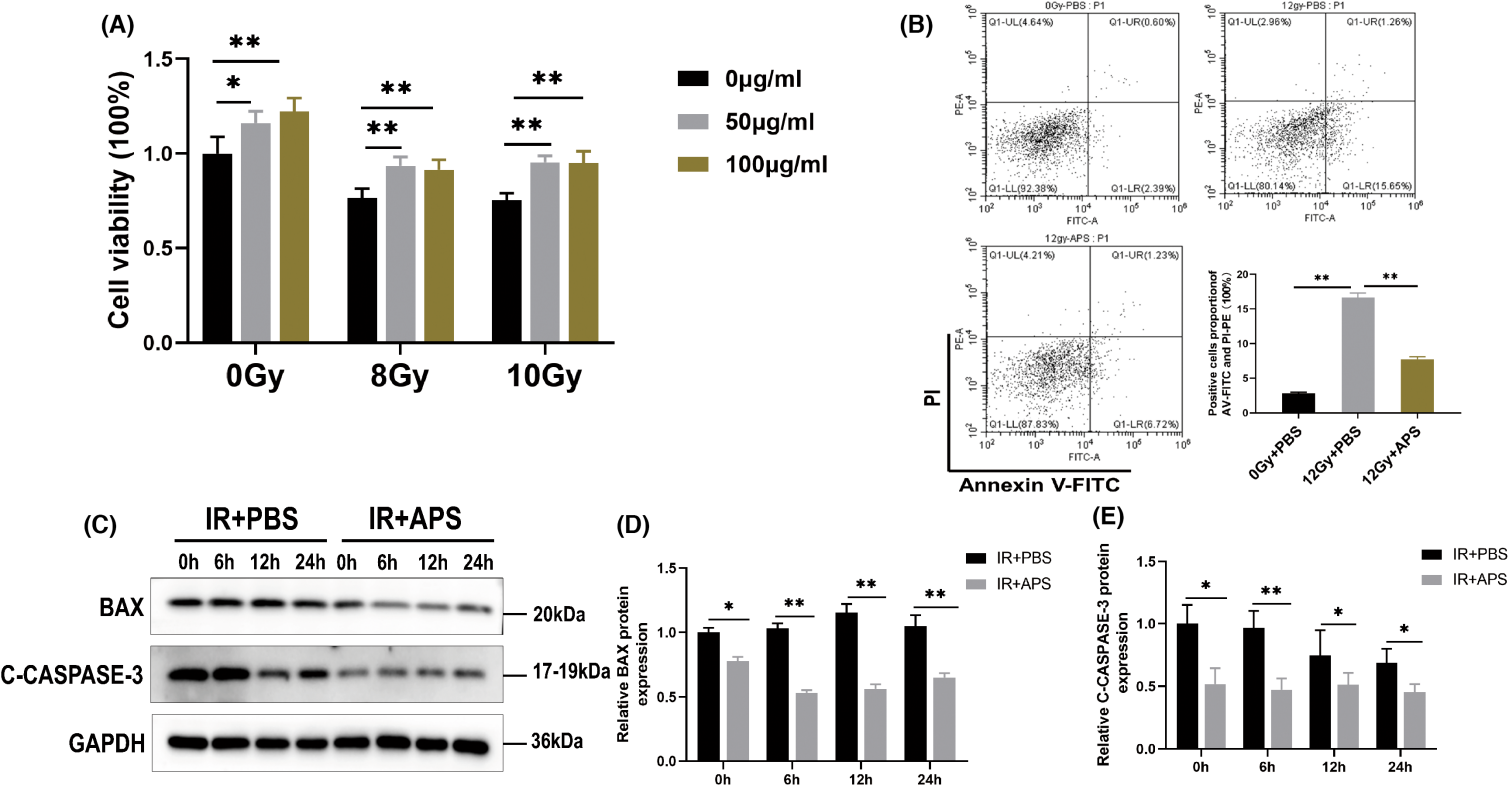
*Astragalus* polysaccharide (APS) alleviated the IR‐induced intestinal damage in vitro. (A) The radioprotective effects of APS on MODE‐K cell viability as measured by the CCK‐8 assay. (B) The radioprotective effects of APS on MODE‐K cell apoptosis was detected by flow cytometry after 12 Gy IR. (C) Western Blot detection of expression of cell apoptosis pathway‐related proteins in MODE‐K. (D, E) Densitometric analysis of BAX and C‐CASPASE3 proteins. The data were presented as mean ± SD. *p < 0.05, **p < 0.01.

In order to figure out the radioprotection of in vitro, MODE‐K cells were treated with 50 μg/mL or 100 μg/mL APS at 12 h and 2 h before IR, then cell viability was detected via CCK‐8 at 48 h after IR, and apoptosis of the cells was detected by flow cytometry at 24 h after IR. In comparison to the control group, APS promoted cell viability (Figure 2A) while reducing apoptosis (Figure 2B) of MODE‐K. In addition, APS reduced the protein expression level of BAX and C‐CASPASE3 in MODE‐K cells by using western blot (Figure 2C). And, by densitometric analysis, we found that the inhibitory effect of APS on the expression of C‐CASPASE3 and BAX was statistically significant (Figure 2D,E). These data revealed that APS alleviated the IR‐induced intestinal damage in vitro.

### 3.3 APS alleviated IR‐induced intestinal injury in vivo

**FIGURE 3:**
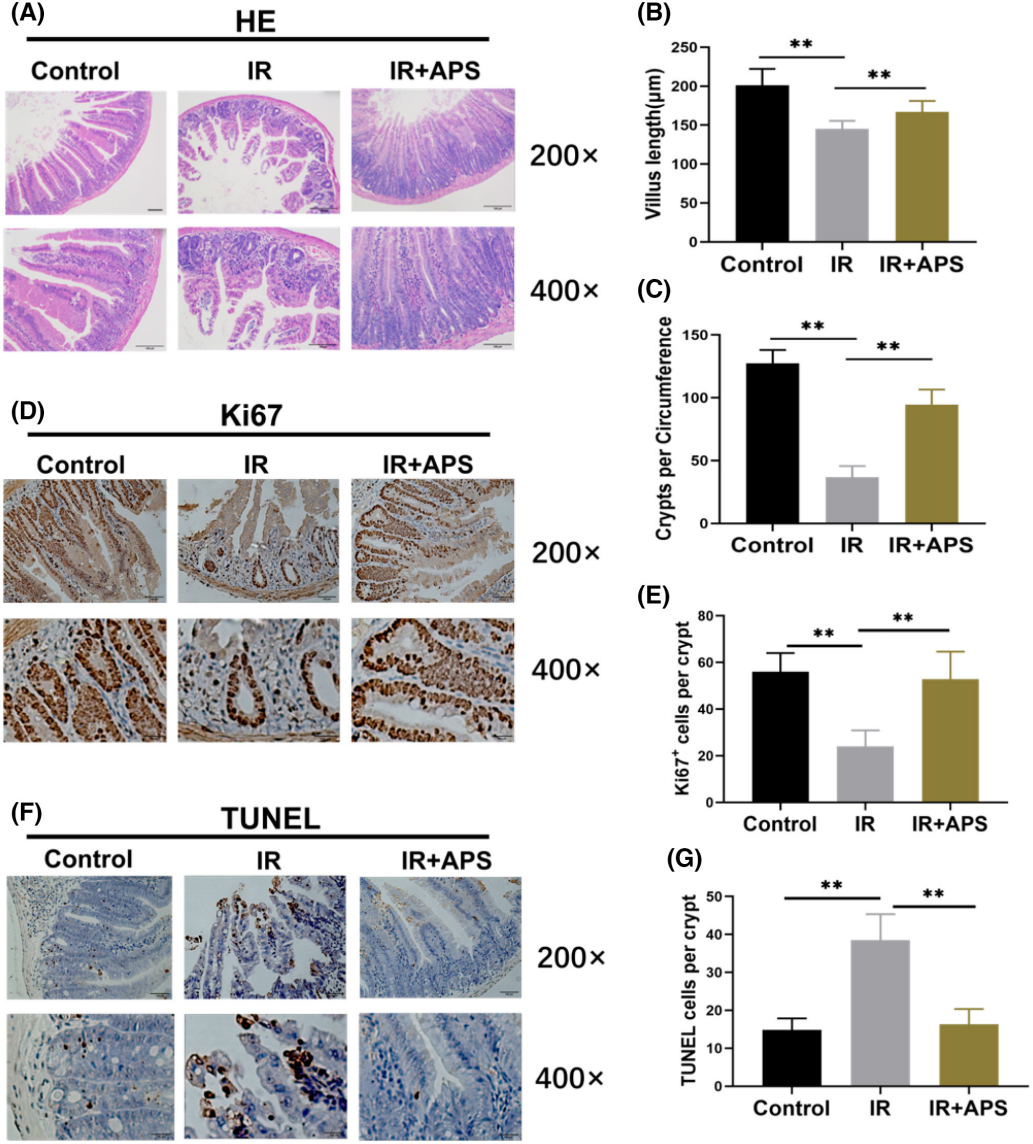
*Astragalus* polysaccharide (APS) alleviated IR induced intestine injury in vivo. (A) Representative images of haematoxylin and eosin staining in mice intestine sections from Control, IR and IR + APS group on day 3.5 after 9.5Gy TBI. Scale bar = 100 μm. (B) Villus length were evaluated in haematoxylin and eosin‐stained sections. (C) The number of crypts per circumference were evaluated in haematoxylin and eosin‐stained sections. (D) Representative images of Ki67 immunohistochemistry in mice intestine sections from Control, IR and IR + APS group on day 3.5 after 9.5 Gy TBI. Scale bar = 100 μm. (E) The number of Ki67 positive cells in each crypt. (F) Representative images of TUNEL immunohistochemistry in mice intestine sections from Control, IR or IR + APS group on day 3.5 after 9.5 Gy TBI. Scale bar = 100 μm. (G) The number of TUNEL positive cells in each crypt. The data were presented as mean ± SD. *p < 0.05, **p < 0.01.

Next, the small intestine of mice was collected after IR to evaluate the degree of intestinal injury. The results of haematoxylin and eosin staining confirmed that APS preserved the crypt‐villus structure and significantly increased the vills length and crypts cell number of mice after 9.5 Gy TBI (Figure 3A–C). Moreover, to specifically label proliferating TA cells, Ki67 immunohistochemistry was performed in small intestine on day 3.5 after 9.5 Gy TBI. The Ki67 immunohistochemistry result showed that the number of Ki67^+^ cells in APS group was significantly higher than that in mice of IR group (Figure 3D,E), which showed that APS could promote the regeneration of ISCs. By using TUNEL immunohistochemistry, we found that APS was able to obviously inhibit intestinal crypt apoptosis after 9.5 Gy TBI (Figure 3F,G). These results proved that APS alleviated IR induced intestinal injury in vivo.

### 3.4 APS alleviated the damage of intestinal mucosal barrier after IR

**FIGURE 4:**
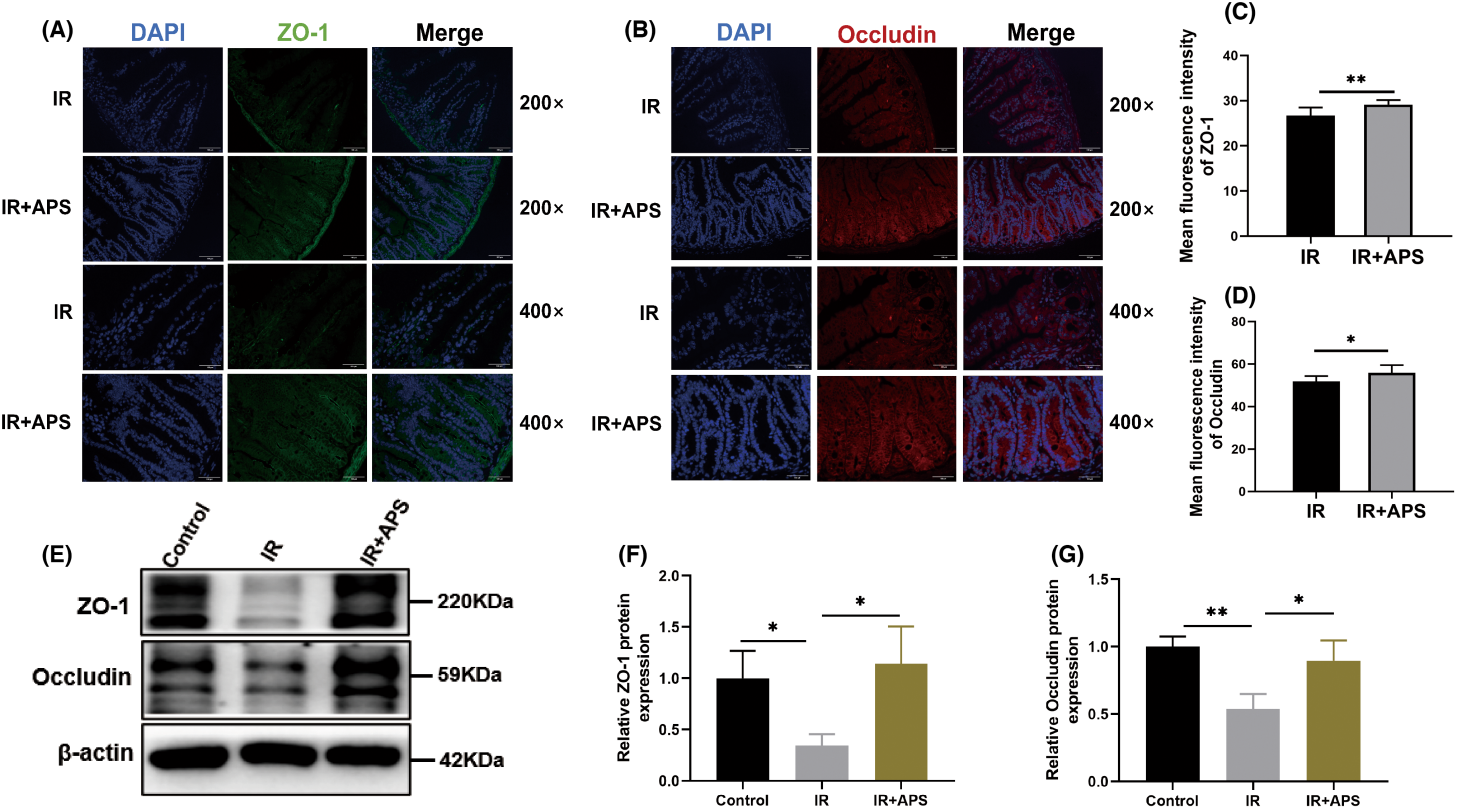
*Astragalus* polysaccharide (APS) alleviated the damage of intestinal mucosal barrier after IR. (A) Representative images of ZO‐1 immunofluorescence in intestinal sections from IR or IR + APS group on day 3.5 after 9.5 Gy TBI. Scale bar = 100 μm (B) representative images of Occludin immunofluorescence in intestinal sections from IR or IR + APS group on day 3.5 after 9.5 Gy TBI. Scale bar = 100 μm. (C) The mean fluorescence intensity of ZO‐1. (D). The mean fluorescence intensity of Occludin. (E) Western blot detection of expression of ZO‐1 and Occludin proteins in small intestine. (F, G) Densitometric analysis of ZO‐1 and Occludin proteins. The data were presented as mean ± SD. *p < 0.05, **p < 0.01.

The intestinal mucosal barrier is an important defence against pathogenic bacteria invasion, while IR damages the intestinal mucosal barrier greatly.^15^ ZO‐1 and Occludin are key proteins of intestinal tight junction proteins. Then immunofluorescence of ZO‐1 and Occludin was used to evaluate the effects of APS on intestinal mucosal barrier. The expression of ZO‐1 and Occludin in IR + APS group were significantly higher than those in the IR treated group, which means that APS could alleviate the damage of intestinal mucosal barrier after IR (Figure 4A–D). Meanwhile, the results of Western Blot showed that IR decreased the expression of ZO‐1 and Occludin proteins in small intestine tissues, while APS could increase the expression of these proteins, and the results were statistically significant (Figure 4E–G).

### 3.5 APS protected the intestinal organoid against IR‐induced injury

**FIGURE 5:**
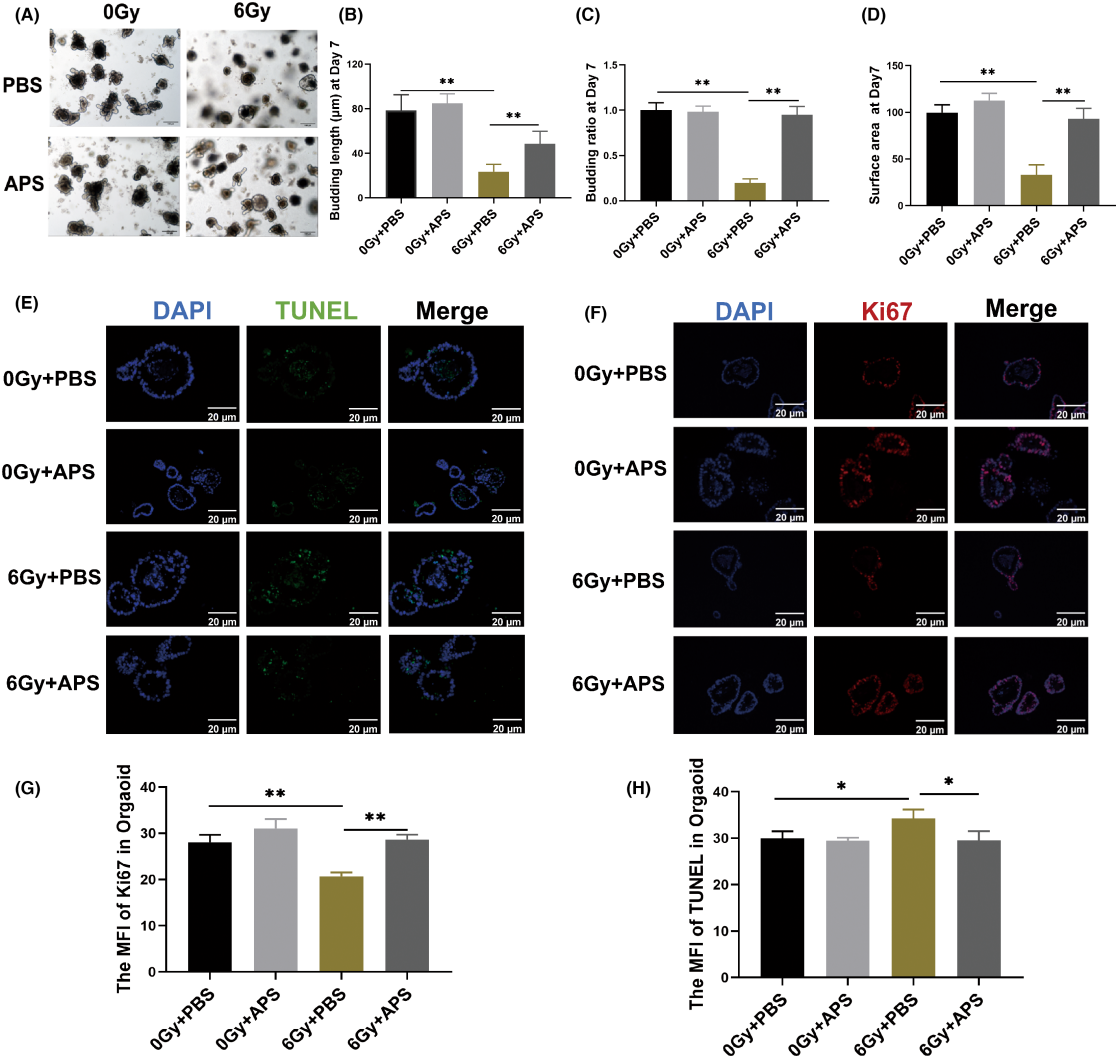
*Astragalus* polysaccharide (APS) protected the intestinal organoid against IR‐induced injury. (A) Representative images of intestinal organoid after IR. Scale bar = 100 μm. (B) The budding length of organoids on the seventh day. (C) The budding ratio of organoids on the seventh day. (D) The surface area of organoids on the seventh day. (E) Representative images of Ki67 immunofluorescence of PBS or APS‐treated organoids after 6 Gy TBI. Scale bar = 20 μm. (F) Representative images of TUNEL immunofluorescence of PBS or APS‐treated organoids after 6 Gy TBI. Scale bar = 20 μm. (G) The MFI of Ki‐67 in intestinal organoids. (H) The MFI of TUNEL in intestinal organoids. The data were presented as mean ± SD. *p < 0.05, **p < 0.01.

The intestinal organoid is a three‐dimensional cellular model and contain all types of enteric epithelial functional cells, including epithelial cells, goblet cells, paneth cells, enteroendocrine cells and ISCs.^16^ The intestinal organoid is a nice technology to study the function and regeneration of ISCs in vitro. We stimulated small intestinal organoids with 100 μg/mL APS or PBS 12 h and 2 h before 6 Gy IR. After 7 days, the small intestinal organoids were evaluated and we found that intestinal organoids in the 6 Gy + APS group have a significantly higher budding length and ratio as well as a greater surface area compared to the 6 Gy + PBS group (Figure 5A–D). These data showed that APS could promote the ability of intestinal organoid formation. Moreover, the results of Ki67 staining and TUNEL staining also showed that APS could promote the proliferation and inhibit the apoptosis of the intestinal organoids after 6 Gy IR (Figure 5E–H). These results showed that APS could significantly improve the proliferation and differentiation of irradiated ISCs.

### 3.6 APS upregulated the expression of ISCs after IR

**FIGURE 6:**
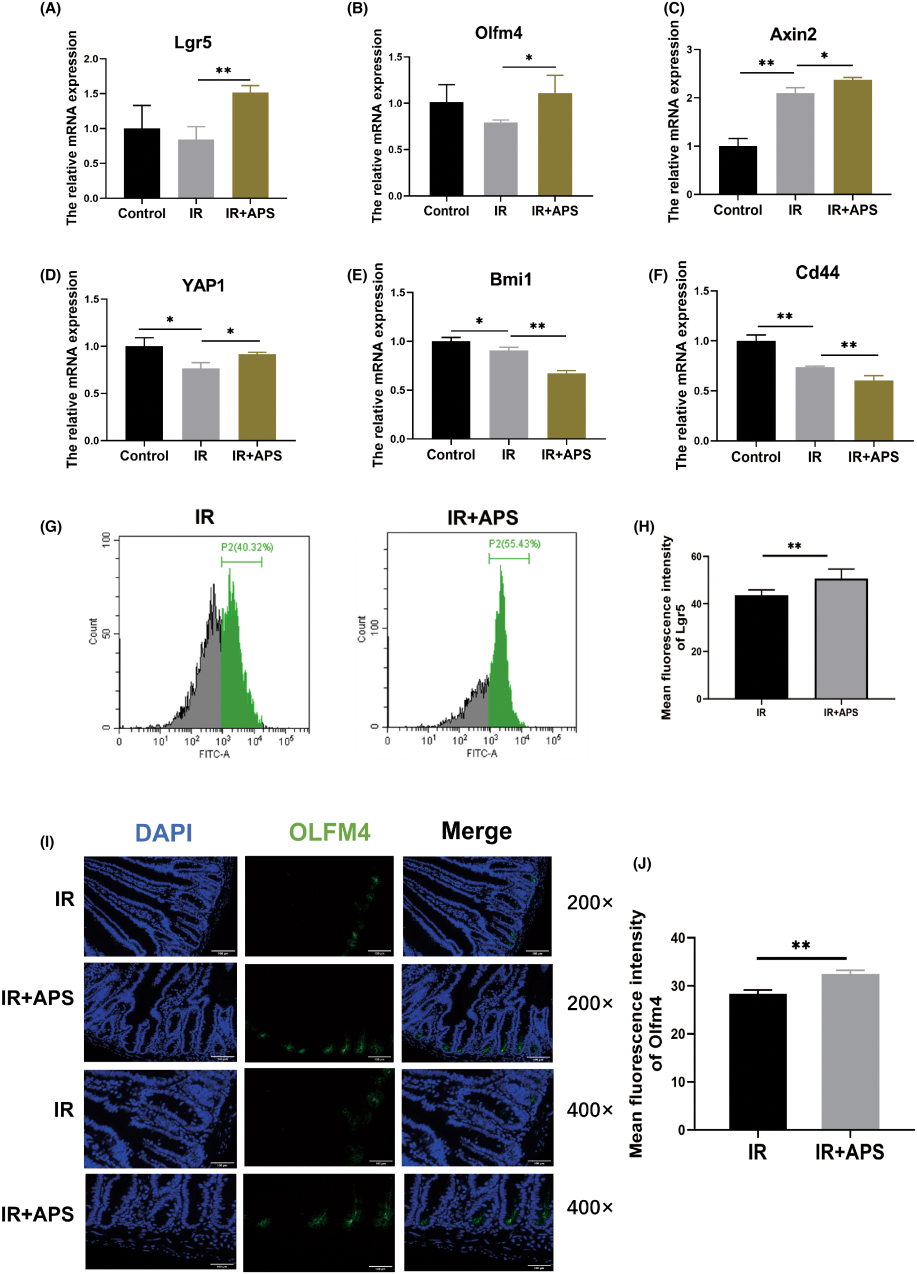
*Astragalus* polysaccharide (APS) upregulated the expression of ISCs after IR. (A–F) The expression of ISCs related genes. (G) The number of Lgr5^+^ ISCs was detected by flow cytometry after 9.5 Gy IR. (H) The mean fluorescence intensity of Lgr5. (I) Representative images of OLFM4 immunofluorescence of IR or IR + APS mice on day 3.5 after 9.5 Gy TBI. Scale bar = 100 μm. (J) The mean fluorescence intensity of OLFM4. The data were presented as mean ± SD. *p < 0.05, **p < 0.01.

ISCs are responsible for the homeostasis of intestinal tissue and important for the regeneration of injured intestinal epithelium.^17^ Thus, we attempted to explore the effects of APS on ISCs. The results of RT‐PCR assays showed that APS significantly up‐regulated the expression of several genes related to Lgr5^+^ ISCs such as Lgr5, OLFM4, Axin2, YAP1 and down‐regulated the expression of genes related to reverse ISCs which can rapidly revert to Lgr5^+^ ISCs to sustain intestinal homeostasis after IR, including Bmi1 and Cd44 (Figure 6A–D,F). Meanwhile, we used flow cytometry to detect the number of Lgr5^+^ cells in the intestinal tissues of Lgr5‐EGFP‐IRES‐creERT2 mice, and the results showed that APS could significantly increase the number of Lgr5^+^ ISCs in intestinal crypt stem cells after IR (Figure 6G,H). Moreover, OLFM4 is an important marker of Lgr5^+^ ISCs and expressed in intestinal epithelial cells residing at the base of the crypt, including the crypt base columnar cells. The numbers and functions of ISCs were assessed by analysing OLFM4^+^ ISCs. The results of OLFM4 immunofluorescence revealed that APS could significantly increase the number of OLFM4^+^ ISCs in the intestine of irradiated mice (Figure 6I,J). Taken together, APS could significantly up‐regulate the expression of genes related to ISCs, and increase the number of Lgr5^+^ and OLFM4^+^ ISCs in the intestinal tract of irradiated mice, suggesting that APS may play radioprotective effects by up‐regulating the number of ISCs after IR.

### 3.7 Identification of DEGs after APS treatment

**FIGURE 7:**
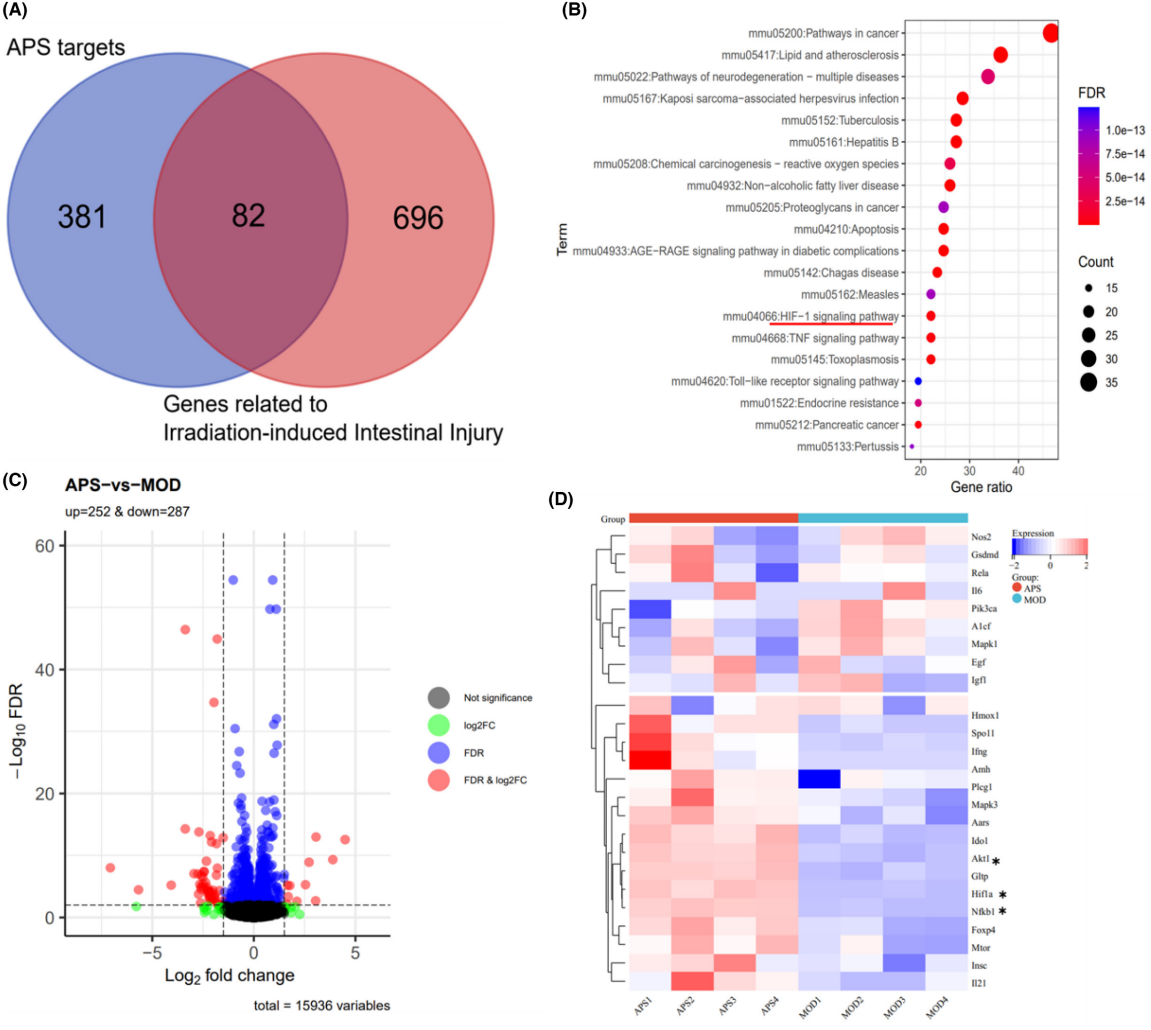
Identification of differential expressed genes (DEGs) after *Astragalus* polysaccharide (APS) treatment. (A) Intersection of targets and disease targets of APS. (B) KEGG pathway enrichment analysis of KEGG pathways within the network pharmacology. (C) Scatter plot of differently expressed genes in intestine tissue after APS treatment. (Each dot stands for a gene. Black represents genes with no differences, green represents genes with differences but no *p*‐values, blue represents genes with *p*‐values but no differences, and red represents genes with both *p*‐values and differences). (D) Heat map of DEGs between MOD mice and APS‐treated mice. “*”representation highligths revelant genes in the HIF‐1 signaling.

To figure out the possible mechanism underlying radioprotection of APS, we conducted the network pharmacology and transcriptome sequencing to further study the mechanism of APS on IR‐induced intestinal injury. Especially, the structure of compounds was obtained from the PubChem database, and the potential 463 targets of compounds were predicted via the TargetNet platform (Table S1). Additionally, the 778 genes associated with IR‐induced intestinal injury were predicted via the GeneCards database (Table S2). By using multiple network pharmacology databases, we found 82 genes in both potential APS targets and IR induced intestinal injury targets (Figure 7A), which might be the potential targets for APS to ameliorate IR‐induced intestinal injury. Next, the KEGG enrichment analysis was conducted and KEGG analysis results showed that these 82 genes enriched in apoptosis, pathway in cancer, HIF‐1 signalling pathway, Toll like receptor signalling pathway and so on (Figure 7B and Table S3). Meantime, 539 differentially expressed genes (DEGs) were identified by using RNA sequencing, including 252 up‐regulated DEGs and 287 down‐regulated DEGs (Figure 7C and Table S4). Combined analysis of network pharmacology and RNA sequencing data, we found that the key DEGs in HIF‐1 signalling pathway was significantly enriched, including HIF‐1α, AKT1 and NF‐κb1 (Figure 7D). These data suggested that HIF‐1 signalling pathway might play critical roles in the radioprotection of APS.

### 3.8 APS activated HIF‐1 signalling pathway in small intestine tissues

**FIGURE 8:**
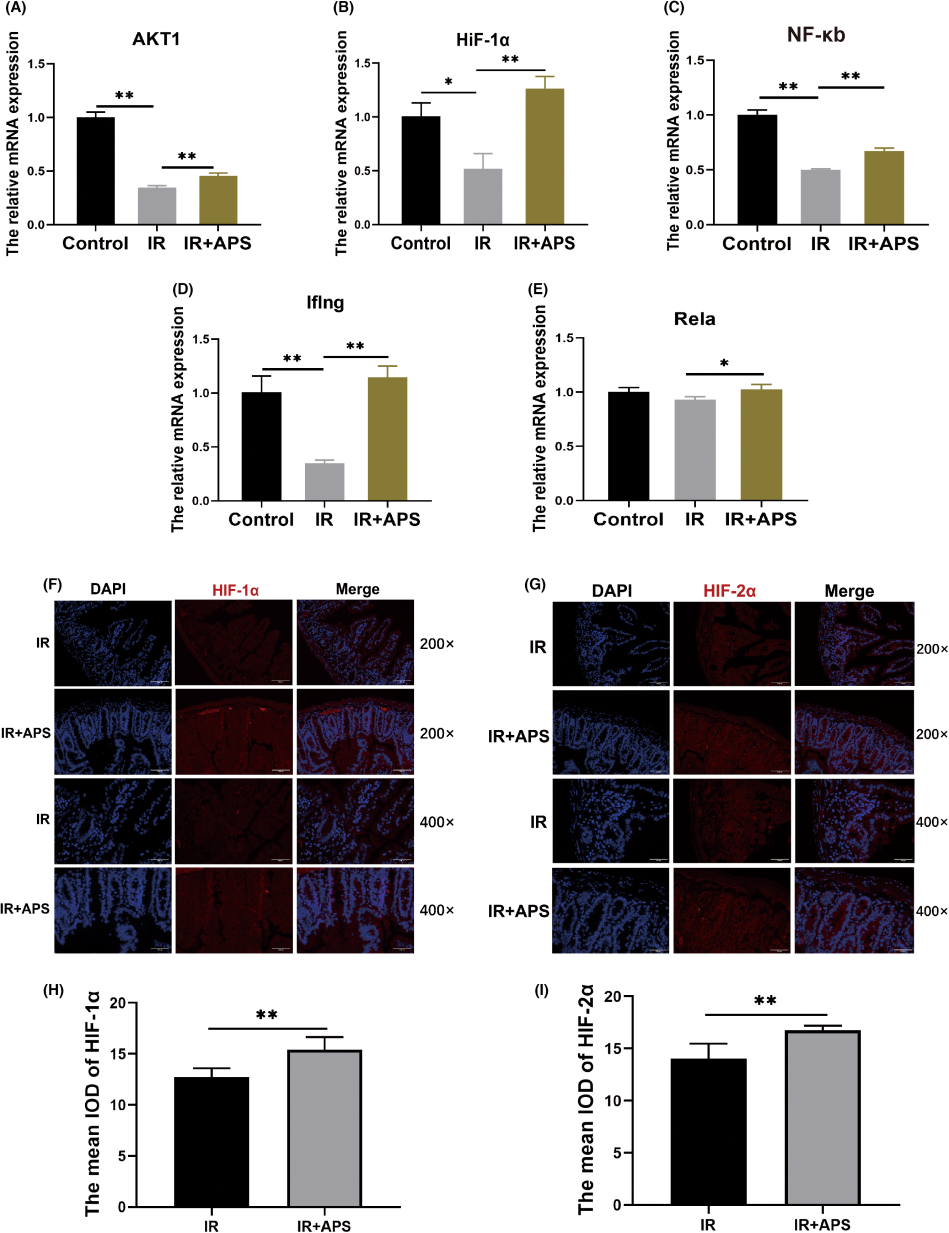
*Astragalus* polysaccharide (APS) activated HIF‐1 signalling pathway in small intestine tissues. (A–E) The RNA expression of HIF‐1α, AKT1, NF‐κb1, Iflng and Rela. (F) Representative images of HIF‐1α immunofluorescence of IR or IR + APS mice on day 3.5 after 9.5 Gy TBI. Scale bar = 100 μm. (G) Representative images of HIF‐2α immunofluorescence of IR or IR + APS mice on day 3.5 day after 9.5 Gy TBI. Scale bar = 100 μm. (H) The mean IOD of HIF‐1α. (I) The mean IOD of HIF‐2α. The data were presented as mean ± SD. *p < 0.05, **p < 0.01.

Combining the results of network pharmacology and RNA seq, we selected HIF‐1 signalling pathway as a key object in APS mediated radioprotection. First, the changes of key genes in HIF‐1 signalling pathway were verified by using RT‐PCR in intestine. The RT‐PCR results showed that APS significantly up‐regulated the expression of key genes such as HIF‐1α, AKT1, NF‐κb1 and Rela after IR (Figure 8A–E). Meanwhile, the results of HIF‐1α and HIF‐2α immunofluorescence revealed that the expression of HIF‐1α and HIF‐2α in IR + APS group were significantly higher than those in the IR treated group. (Figure 8F–I). The above results suggest that APS can indeed activate the HIF‐1 signalling pathway.

### 3.9 HIF‐1 signalling pathway played a critical role in the radioprotection of APS

**FIGURE 9:**
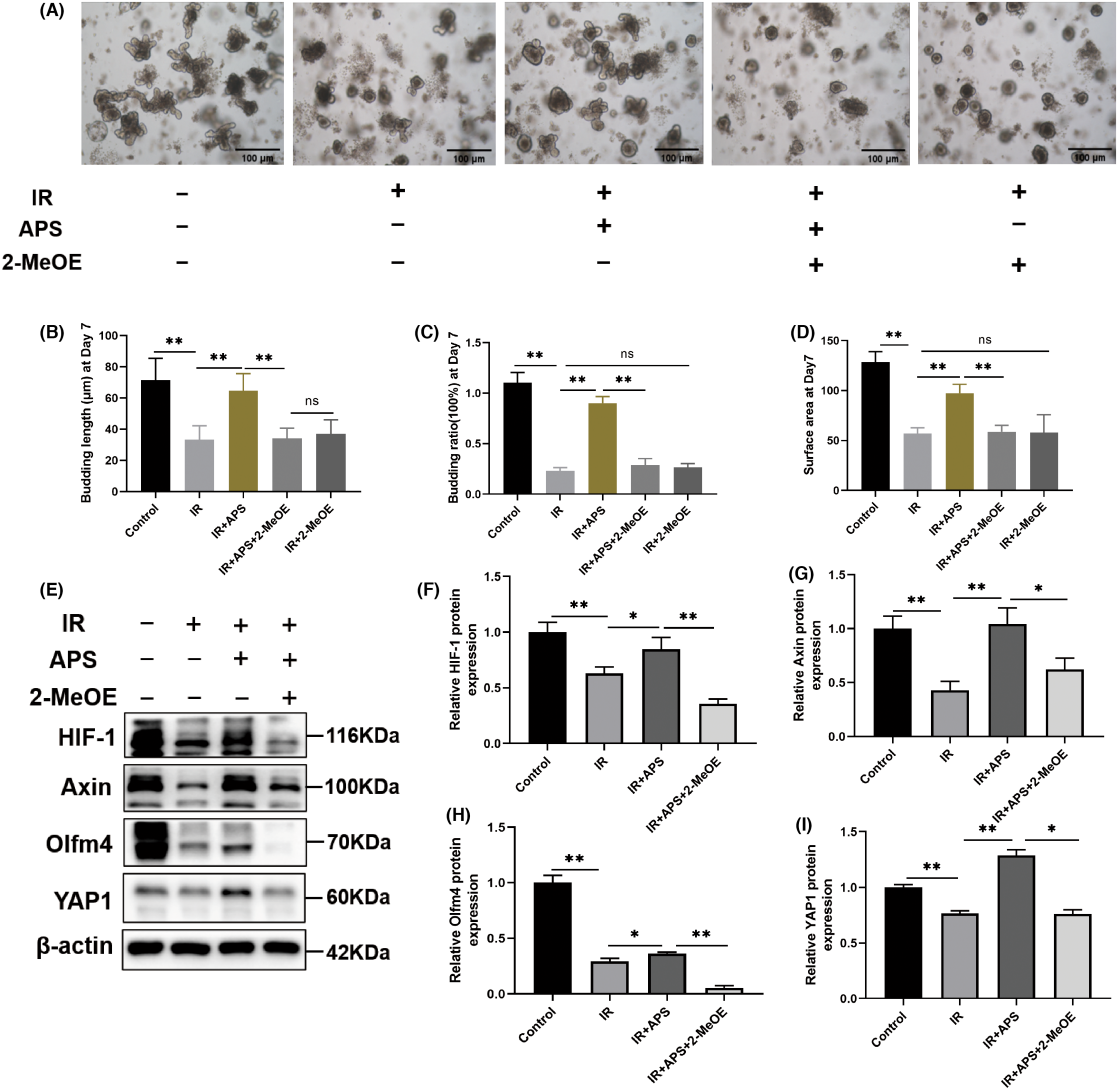
HIF‐1 signalling pathway played a critical role in the radioprotection of *Astragalus* polysaccharide (APS). (A) Representative images of intestinal organoids. Scale bar = 100 μm. (B–D) The budding length, budding rate and the surface area of intestinal organoids on day 7 after 6 Gy IR. (E) Western blot detection of expression of YAP1, Olfm4, Axin and HIF‐1 proteins in small intestine. (F–I) Densitometric analysis of YAP1, Olfm4, Axin and HIF‐1 proteins. The data were presented as mean ± SD. *p < 0.05, **p < 0.01.

To further explore the important role of HIF‐1 signalling pathway in APS protection against radiation intestinal injury, we used 2‐Methoxyestradiol (2‐MeOE2), a selective small molecule inhibitor to inhibit the HIF‐1α. The results showed that the growth of the IR organoids was significantly inhibited compared with the Control organoids, and a few individual organoids were able to germinate, but their size was much smaller than that of the Control group, while APS significantly improved the inhibition of the irradiated intestinal organoids, increasing the budding length and budding rate as well as the surface area. However, when the intestinal organoids were stimulated with 2‐MeOE, inhibiting the HIF‐1 signalling pathway, the proliferation‐promoting effect of APS on intestinal organoids disappeared abruptly, while the growth of intestinal organoids was also significantly inhibited when 2‐MeOE was administered alone, suggesting that the HIF‐1 signalling pathway plays a key role in the growth of intestinal organoids. (Figure 9A–D). Meanwhile, we found that APS stimulation promoted the expression of ISC‐related proteins (YAP1, Olfm4 and Axin) after irradiation by Western Blot assay, while the promotion effect of APS on the expression of these proteins no longer existed after disrupting the HIF‐1 signalling pathway with 2‐MeOE (Figure 9E–I), which also proved that APS can activate the HIF‐1 signalling pathway and thus promote the expression of ISCs‐related proteins, thus exerting an anti‐radioactive intestinal injury effect.

## 4 DISCUSSION

IR‐induced intestinal injury associates with high mortality, which is a worldwide problem requiring urgent attention.^18^ ISCs are the source of all types of intestinal cell reservoirs, providing the nascent force that keeps the intestine fresh and alive at all times, and are therefore responsible for the homeostasis of the intestine, essential for the recovery of damaged intestinal tissues.^19^, ^20^ As the intestinal cavity can be directly exposed to disease‐causing environmental factors, it is susceptible to chemical, pathogenic or irradiation‐induced damage.^21^, ^22^, ^23^ The intestine is highly susceptible to damage from IR, in part because IR causes the death of a large number of ISCs. Among them, Lgr5‐labelled mitotically active ISCs, also known as Crypt‐base columnar cells (CBCs), are major drivers of small intestinal epithelial renewal and contribute significantly to homeostatic regeneration in vivo, but they are highly sensitive to IR.^24^ When Lgr5^+^ ISCs are depleted, Bmi1^+^ cells rapidly revert to Lgr5^+^ ISCs to sustain intestinal homeostasis.^25^

The herbal resources used for both medicine and food have the characteristics of low toxicity, multi‐component, multi‐target and multi‐path to exert their medicinal effects, which have unique natural advantages over chemical anti‐radiation drugs in the research of anti‐radiation drugs, of which AM is one of the representatives. In this work, we found that APS, one of the main active ingredients of AM, had radioprotective effects on IR‐induced intestinal injury in vivo and in vitro. Through animal experiments, we found that APS effectively improved the survival rate, reduced weight loss and intestinal permeability of mice after IR, and APS was also capable of attenuating apoptosis as well as promoting MODE‐K cell viability after IR. To further explore the effects of APS on IR‐induced intestinal injury, we stained intestinal tissue using HE staining, Ki67 staining, and TUNEL staining and found that APS does indeed mitigate IR‐induced intestinal pathologic injury, promoting the promotion of small intestinal crypt cells and inhibiting their apoptosis. Meanwhile, immunofluorescence and Western Blot experiments revealed that APS could promote the expression of intestinal tight junction proteins and restore the integrity of the intestinal barrier.

Intestinal organoids have unlimited proliferation ability and can simulate many characteristics of the intestine.^16^ The unique feature of intestinal organoids is that they can retain the function of self‐renewal and differentiation of cells in the intestine, have a more stable genome and are closer to the normal physiological state than ordinary cells, and can better imitate the in vivo environment. Morphological and statistical analyses of cultured intestinal organoids treated with APS stimulation and radiation showed that APS significantly promoted the growth of intestinal organoids, as evidenced by higher germination rates, longer germination lengths and larger surface areas. In addition, the results of immunofluorescence Ki‐67 and TUNEL staining of intestinal organoids showed that APS promoted the proliferation and inhibited the apoptosis of irradiated small intestinal crypt cells.

IR can directly damage ISCs, impair the proliferation and differentiation of ISCs.^26^ Understanding the regulatory mechanisms of ISCs is key to preventing and treating IR‐induced intestinal injury. Given the important role of ISCs in IR‐induced intestinal injury, Lgr5‐EGFP‐IRES‐creERT2 mice were used to detect the number of Lgr5^+^ ISCs by flow cytometry. Our results showed that APS could facilitate the recovery of Lgr5^+^ ISCs after IR. OLFM4 is an important marker of Lgr5^+^ ISCs. Then our data of OLFM4 immunofluorescence suggested IR had a significant inhibitory effect on Lgr5^+^ ISCs, and APS significantly alleviated the suppression of OLFM4 after IR. Meanwhile, we examined the effects of APS on some ISCs related genes by quantitative PCR. The results showed that APS can promote expression of some genes such as Lgr5, OLFM4, Axin2 and YAP1. These results suggested that APS may play a radioprotective effect by promoting the regeneration of ISCs after IR.

**FIGURE 10:**
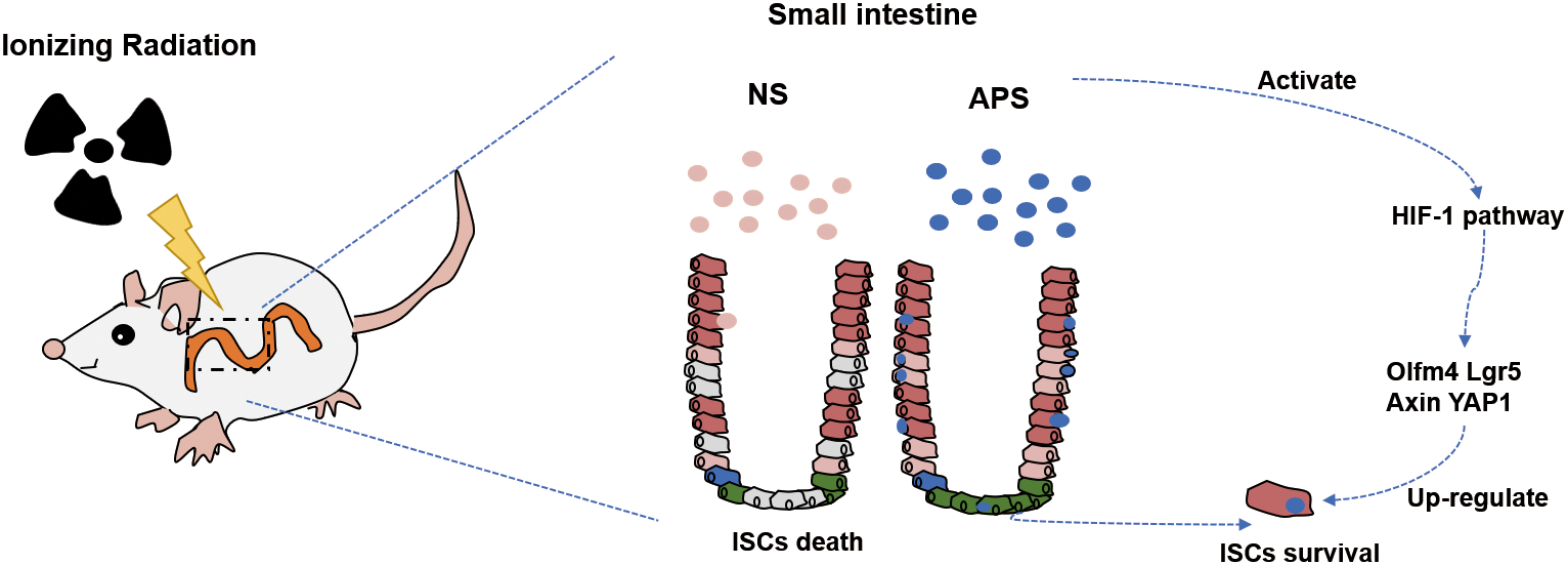
Schematic diagram of the anti‐radioactive intestinal injury effect of *Astragalus* polysaccharide (APS) by activating the HIF‐1 signalling pathway to promote the proliferation of ISCs.

Recent studies revealed that HIF signalling pathway is a new target for the treatment of IR‐induced intestinal injury. Yun Chen et al. reported that DFO, a hypoxia‐mimicking compound that upregulates HIF‐1 α expression, enhances ISCs proliferation and crypt class formation, and activates Lgr5 expression.^27^ Taniguchi et al. demonstrated that elevating HIF levels by dimethyloxallyl glycine (DMOG), a PhD inhibitor, could effectively attenuate IR‐induced intestinal injury and improve the survival rate of mice after IR.^28^ Olcina and Giaccia reported that activation of the HIF‐1 pathway represents a novel therapeutic strategy aimed at protecting and ameliorating IR induced gastrointestinal toxicity.^29^ To figure out the possible mechanism underlying radioprotection of APS, we conducted the network pharmacology and transcriptome sequencing to further study the mechanism of APS on IR‐induced intestinal injury. Combining the results of network pharmacology and transcriptome sequencing, the HIF‐1 signalling pathway was selected as a key pathway for APS‐mediated regeneration of ISCs. By using quantitative PCR, we found that APS could signifcantly upregulate the genes of HIF‐1 signalling pathway, including HIF‐1α, AKT1, Iflng, NF‐κb1 and Rela. Then 2‐MeOE2, the inhibitor of HIF‐1α, was used to verify whether the radioprotective effects of APS were dependent on HIF‐1α signalling pathway. The intestinal organoid results showed that the HIF‐1α inhibitor completely abolished the radioprotective effects of APS. However, there is still a doubt here, we can see that APS does promote the growth of IR organoids, and its growth‐promoting effect disappears after the administration of 2‐MeOE, but it is not entirely clear that APS promotes the growth of organoids through the HIF‐1 signalling pathway, because the growth of organoids is also limited by the administration of 2‐MeOE alone. Then, we found that APS stimulation promoted the expression of ISCs‐related proteins after irradiation by Western Blot assay, while 2‐MeOE can inhibit this effect. These results imply that intestinal radioprotection by APS is dependent on the HIF‐1 signalling pathway (Figure 10).

## 5 CONCLUSION

In conclusion, we demonstrated that APS protects the intestine from IR‐induced injury in vitro and in vivo. Next, we demonstrated that APS significantly upregulated the HIF‐1 signalling pathway, and HIF‐1α inhibitor destroyed the radioprotection of APS by using network pharmacology, RNA‐seq and small inhibitor. Taken together, our findings suggested that APS promoted the regeneration of ISCs through HIF‐1 signalling pathway, and APS may be an effective radioprotective agent for IR‐induced intestinal injury.

## AUTHOR CONTRIBUTIONS

**Qianqian Ding:** Conceptualization (supporting); data curation (supporting); methodology (equal); project administration (equal); resources (equal); writing – original draft (lead). **Xianpeng Zu:** Funding acquisition (lead); investigation (supporting); methodology (supporting); writing – original draft (supporting). **Wei Chen:** Investigation (supporting); methodology (supporting); validation (supporting); writing – original draft (supporting). **Jiayun Xin:** Investigation (supporting); methodology (supporting); visualization (supporting). **Xike Xu:** Investigation (supporting); methodology (supporting). **Yanhui Lv:** Visualization (equal). **Xintong Wei:** Visualization (equal). **Jie Wang:** Methodology (equal). **Yanping Wei:** Methodology (equal). **Zhanhong Li:** Methodology (equal). **Jianming Cai:** Methodology (equal); resources (equal). **Jicong Du:** Conceptualization (equal); funding acquisition (equal); writing – review and editing (equal). **Weidong Zhang:** Conceptualization (equal); funding acquisition (equal); methodology (equal); writing – review and editing (equal).

## FUNDING INFORMATION

This study was supported by the National Natural Science Foundation of China (82004215, 82003624, 82141203, 82004003, 82103779), the National Key Research and Development Program of China (2022YFC3502000, 2019YFC1711006), National Science and Technology Major Project of China (2018ZX09731016‐005, 2019ZX09201004‐003‐010), Shanghai Municipal Health Commission Project (20204Y0326), Science and Technology Commission of Shanghai Municipality (20YF1458700, 20YF1459000), Three‐year Action Plan for Shanghai TCM Development and Inheritance Program [ZY (2021‐2023)‐0401] and Sailing Program of Naval Medical University.

## CONFLICT OF INTEREST STATEMENT

The authors have no potential conflict of interests.

## CONSENT FOR PUBLICATION

All authors reached an agreement to publish the study in this journal.

## Supporting information


Table S1.

Table S2.

Table S3.

Table S4.

## Data Availability

All data generated or analysed during this study were included in this published article and its supplemental material.
