# Supplementary material for: Astragalus polysaccharide promotes the regeneration of intestinal stem cells through HIF‐1 signalling pathway
Source: J Cell Mol Med. 2023 Dec 14;28(3):e18058. doi: 10.1111/jcmm.18058 (PMC10844761; doi:10.1111/jcmm.18058)
Supplement: Supplementary file 1 — Table S1. [file JCMM-28-e18058-s002.pdf]

The list of 463 potential targets on APS aquired on the TargetNet platform

Compound	Target	Source
L-Arabinose	AKR1B1	TCMID:8912;TCMSP:TAR00288
L-Arabinose	AKR1B10	TCMID:7960
D(+)-Fucose	FUCA1	TCMID:1816;PubChem_Literature
D(+)-Fucose	FUCA2	TCMID:11044
D(+)-Fucose	MASP1	TCMID:619
D(+)-Fucose	COLEC11	TCMID:12712
D(+)-Fucose	FCSK	TCMID
L-Galactose	AKR1B1	TCMID:8912;TCMSP:TAR00288;PubChem_Literature
L-Galactose	GALC	TCMID:478
L-Galactose	GLA	TCMID:12513;TCMSP:TAR00609
L-Galactose	GLB1	TCMID:10709;TCMSP:TAR01173;PubChem_Literature
L-Galactose	LCT	SymMap:SMTT01926;HIT:T0594;TCMSP:TAR00719;PubChem_CTD
L-Galactose	AKR1B10	TCMID:7960
L-Galactose	GALM	TCMID:10325;PubChem_Literature
L-Arabinose	TLR9	TargetNet
L-Galactose	TLR9	TargetNet
D-Mannopyra	TLR9	TargetNet
D-(+)-Xylose	TLR9	TargetNet
D-Glucose	TLR9	TargetNet
L-Arabinose	SIGMAR1	TargetNet
D-(+)-Xylose	SIGMAR1	TargetNet
L-Arabinose	S1PR2	TargetNet
L-Galactose	S1PR2	TargetNet
D-Mannopyra	S1PR2	TargetNet
L-Rhamnose	S1PR2	TargetNet
D-(+)-Xylose	S1PR2	TargetNet
D(+)-Fucose	S1PR2	TargetNet
D-Glucose	S1PR2	TargetNet
L-Arabinose	RORA	TargetNet
L-Rhamnose	RORA	TargetNet
D-(+)-Xylose	RORA	TargetNet
D(+)-Fucose	RORA	TargetNet
L-Arabinose	RIPK2	TargetNet
L-Rhamnose	RIPK2	TargetNet
D-(+)-Xylose	RIPK2	TargetNet
D(+)-Fucose	RIPK2	TargetNet
L-Rhamnose	RELA	TargetNet
D(+)-Fucose	RELA	TargetNet
L-Arabinose	PTGS1	TargetNet
L-Rhamnose	PTGS1	TargetNet
D-(+)-Xylose	PTGS1	TargetNet
D(+)-Fucose	PTGS1	TargetNet
L-Arabinose	PTGFR	TargetNet
D-(+)-Xylose	PTGFR	TargetNet
L-Arabinose	PNMT	TargetNet
L-Galactose	PNMT	TargetNet
D-Mannopyra	PNMT	TargetNet
D-(+)-Xylose	PNMT	TargetNet
D-Glucose	PNMT	TargetNet
L-Arabinose	NR2F2	TargetNet
L-Galactose	NR2F2	TargetNet
D-Mannopyra	NR2F2	TargetNet
L-Rhamnose	NR2F2	TargetNet
D-(+)-Xylose	NR2F2	TargetNet
D(+)-Fucose	NR2F2	TargetNet
D-Glucose	NR2F2	TargetNet
L-Arabinose	NOS3	TargetNet

A total of 463 potential targets were obtained by searching for Astragalus polysaccharide compounds on Pubchem and HERB databases and predicting targets base on the SMILES structual formula of the coumpounds on the TargetNet platform(taking probability >0.9)

Continued from above

L-Galactose	NOS3	TargetNet
D-Mannopyra	NOS3	TargetNet
L-Rhamnose	NOS3	TargetNet
D-(+)-Xylose	NOS3	TargetNet
D(+)-Fucose	NOS3	TargetNet
D-Glucose	NOS3	TargetNet;PubChem_Literature;PubChem_CTD
L-Arabinose	NOS2	TargetNet
L-Rhamnose	NOS2	TargetNet
D-(+)-Xylose	NOS2	TargetNet
D(+)-Fucose	NOS2	TargetNet
L-Arabinose	NOS1	TargetNet
L-Galactose	NOS1	TargetNet
D-Mannopyra	NOS1	TargetNet
L-Rhamnose	NOS1	TargetNet
D-(+)-Xylose	NOS1	TargetNet
D(+)-Fucose	NOS1	TargetNet
D-Glucose	NOS1	TargetNet
L-Arabinose	MIF	TargetNet
L-Galactose	MIF	TargetNet
D-Mannopyra	MIF	TargetNet
L-Rhamnose	MIF	TargetNet
D-(+)-Xylose	MIF	TargetNet
D(+)-Fucose	MIF	TargetNet
D-Glucose	MIF	TargetNet
L-Arabinose	MGLL	TargetNet
L-Galactose	MGLL	TargetNet
D-Mannopyra	MGLL	TargetNet
L-Rhamnose	MGLL	TargetNet
D-(+)-Xylose	MGLL	TargetNet
D(+)-Fucose	MGLL	TargetNet
D-Glucose	MGLL	TargetNet
L-Arabinose	HTR5A	TargetNet
L-Rhamnose	HTR5A	TargetNet
D-(+)-Xylose	HTR5A	TargetNet
D(+)-Fucose	HTR5A	TargetNet
L-Arabinose	HTR1E	TargetNet
L-Galactose	HTR1E	TargetNet
D-Mannopyra	HTR1E	TargetNet
L-Rhamnose	HTR1E	TargetNet
D-(+)-Xylose	HTR1E	TargetNet
D(+)-Fucose	HTR1E	TargetNet
D-Glucose	HTR1E	TargetNet
L-Arabinose	HSD11B1	TargetNet
D-(+)-Xylose	HSD11B1	TargetNet
L-Arabinose	GRM4	TargetNet
L-Rhamnose	GRM4	TargetNet
D-(+)-Xylose	GRM4	TargetNet
D(+)-Fucose	GRM4	TargetNet
L-Arabinose	GRIN2B	TargetNet
D-(+)-Xylose	GRIN2B	TargetNet
L-Arabinose	GPR35	TargetNet
L-Rhamnose	GPR35	TargetNet
D-(+)-Xylose	GPR35	TargetNet
D(+)-Fucose	GPR35	TargetNet
L-Arabinose	GALR3	TargetNet
L-Galactose	GALR3	TargetNet
D-Mannopyra	GALR3	TargetNet
L-Rhamnose	GALR3	TargetNet

A total of 463 potential targets were obtained by searching for Astragalus polysaccharide compounds on Pubchem and HERB databases and predicting targets base on the SMILES structual formula of the coumpounds on the TargetNets platform(taking probability >0.9)

Continued from above

D-(+)-Xylose	GALR3	TargetNet
D(+)-Fucose	GALR3	TargetNet
D-Glucose	GALR3	TargetNet
L-Rhamnose	DUSP3	TargetNet
D(+)-Fucose	DUSP3	TargetNet
L-Arabinose	DRD5	TargetNet
L-Rhamnose	DRD5	TargetNet
D-(+)-Xylose	DRD5	TargetNet
D(+)-Fucose	DRD5	TargetNet
L-Arabinose	DPP7	TargetNet
D-(+)-Xylose	DPP7	TargetNet
L-Rhamnose	CYP1A2	TargetNet
D(+)-Fucose	CYP1A2	TargetNet
L-Arabinose	CYP17A1	TargetNet
D-(+)-Xylose	CYP17A1	TargetNet
L-Arabinose	CHRNA7	TargetNet
L-Rhamnose	CHRNA7	TargetNet
D-(+)-Xylose	CHRNA7	TargetNet
D(+)-Fucose	CHRNA7	TargetNet
L-Arabinose	CHRNA4	TargetNet
L-Galactose	CHRNA4	TargetNet
D-Mannopyra	CHRNA4	TargetNet
L-Rhamnose	CHRNA4	TargetNet
D-(+)-Xylose	CHRNA4	TargetNet
D(+)-Fucose	CHRNA4	TargetNet
D-Glucose	CHRNA4	TargetNet
L-Arabinose	CHRM4	TargetNet
L-Galactose	CHRM4	TargetNet
D-Mannopyra	CHRM4	TargetNet
L-Rhamnose	CHRM4	TargetNet
D-(+)-Xylose	CHRM4	TargetNet
D(+)-Fucose	CHRM4	TargetNet
D-Glucose	CHRM4	TargetNet
L-Arabinose	CHRM2	TargetNet
L-Rhamnose	CHRM2	TargetNet
D-(+)-Xylose	CHRM2	TargetNet
D(+)-Fucose	CHRM2	TargetNet
L-Arabinose	CHRM1	TargetNet
L-Rhamnose	CHRM1	TargetNet
D-(+)-Xylose	CHRM1	TargetNet
D(+)-Fucose	CHRM1	TargetNet
L-Arabinose	CHRFAM7	TargetNet
L-Rhamnose	CHRFAM7	TargetNet
D-(+)-Xylose	CHRFAM7	TargetNet
D(+)-Fucose	CHRFAM7	TargetNet
L-Arabinose	CES2	TargetNet
L-Galactose	CES2	TargetNet
D-Mannopyra	CES2	TargetNet
L-Rhamnose	CES2	TargetNet
D-(+)-Xylose	CES2	TargetNet
D(+)-Fucose	CES2	TargetNet
D-Glucose	CES2	TargetNet
L-Arabinose	CES1	TargetNet
L-Galactose	CES1	TargetNet
D-Mannopyra	CES1	TargetNet
L-Rhamnose	CES1	TargetNet
D-(+)-Xylose	CES1	TargetNet
D(+)-Fucose	CES1	TargetNet

A total of 463 potential targets were obtained by searching for Astragalus polysaccharide compounds on Pubchem and HERB databases and predicting targets base on the SMILES structual formula of the coumpounds on the TargetNets platform(taking probability >0.9)

D-Glucose	CES1	TargetNet
L-Arabinose	CDC25B	TargetNet
L-Galactose	CDC25B	TargetNet
D-Mannopyra	CDC25B	TargetNet
L-Rhamnose	CDC25B	TargetNet
D-(+)-Xylose	CDC25B	TargetNet
D(+)-Fucose	CDC25B	TargetNet
D-Glucose	CDC25B	TargetNet
L-Arabinose	CA9	TargetNet
L-Galactose	CA9	TargetNet
D-Mannopyra	CA9	TargetNet
L-Rhamnose	CA9	TargetNet
D-(+)-Xylose	CA9	TargetNet
D(+)-Fucose	CA9	TargetNet
D-Glucose	CA9	TargetNet
L-Arabinose	CA7	TargetNet
L-Galactose	CA7	TargetNet
D-Mannopyra	CA7	TargetNet
L-Rhamnose	CA7	TargetNet
D-(+)-Xylose	CA7	TargetNet
D(+)-Fucose	CA7	TargetNet
D-Glucose	CA7	TargetNet
L-Arabinose	CA6	TargetNet
L-Galactose	CA6	TargetNet
D-Mannopyra	CA6	TargetNet
L-Rhamnose	CA6	TargetNet
D-(+)-Xylose	CA6	TargetNet
D(+)-Fucose	CA6	TargetNet
D-Glucose	CA6	TargetNet
L-Arabinose	CA5B	TargetNet
L-Galactose	CA5B	TargetNet
D-Mannopyra	CA5B	TargetNet
L-Rhamnose	CA5B	TargetNet
D-(+)-Xylose	CA5B	TargetNet
D(+)-Fucose	CA5B	TargetNet
D-Glucose	CA5B	TargetNet
L-Arabinose	CA5A	TargetNet
L-Galactose	CA5A	TargetNet
D-Mannopyra	CA5A	TargetNet
L-Rhamnose	CA5A	TargetNet
D-(+)-Xylose	CA5A	TargetNet
D(+)-Fucose	CA5A	TargetNet
D-Glucose	CA5A	TargetNet
L-Arabinose	CA4	TargetNet
L-Galactose	CA4	TargetNet
D-Mannopyra	CA4	TargetNet
L-Rhamnose	CA4	TargetNet
D-(+)-Xylose	CA4	TargetNet
D(+)-Fucose	CA4	TargetNet
D-Glucose	CA4	TargetNet
L-Arabinose	CA2	TargetNet
L-Galactose	CA2	TargetNet
D-Mannopyra	CA2	TargetNet
L-Rhamnose	CA2	TargetNet
D-(+)-Xylose	CA2	TargetNet
D(+)-Fucose	CA2	TargetNet
D-Glucose	CA2	TargetNet
L-Arabinose	CA14	TargetNet

A total of 463 potential targets were obtained by searching for Astragalus polysaccharide compounds on Pubchem and HERB databases and predicting targets base on the SMILES structual formula of the coumpounds on the TargetNets platform(taking probability >0.9)

L-Galactose	CA14	TargetNet
D-Mannopyra	CA14	TargetNet
L-Rhamnose	CA14	TargetNet
D-(+)-Xylose	CA14	TargetNet
D(+)-Fucose	CA14	TargetNet
D-Glucose	CA14	TargetNet
L-Arabinose	CA13	TargetNet
L-Galactose	CA13	TargetNet
D-Mannopyra	CA13	TargetNet
L-Rhamnose	CA13	TargetNet
D-(+)-Xylose	CA13	TargetNet
D(+)-Fucose	CA13	TargetNet
D-Glucose	CA13	TargetNet
L-Arabinose	CA12	TargetNet
L-Galactose	CA12	TargetNet
D-Mannopyra	CA12	TargetNet
L-Rhamnose	CA12	TargetNet
D-(+)-Xylose	CA12	TargetNet
D(+)-Fucose	CA12	TargetNet
D-Glucose	CA12	TargetNet
L-Arabinose	CA1	TargetNet
L-Galactose	CA1	TargetNet
D-Mannopyra	CA1	TargetNet
L-Rhamnose	CA1	TargetNet
D-(+)-Xylose	CA1	TargetNet
D(+)-Fucose	CA1	TargetNet
D-Glucose	CA1	TargetNet
L-Arabinose	APP	TargetNet
L-Rhamnose	APP	TargetNet
D-(+)-Xylose	APP	TargetNet
D(+)-Fucose	APP	TargetNet
L-Arabinose	APOBEC3(	TargetNet
L-Rhamnose	APOBEC3(	TargetNet
D-(+)-Xylose	APOBEC3(	TargetNet
D(+)-Fucose	APOBEC3(	TargetNet
L-Arabinose	APOBEC3/	TargetNet
L-Galactose	APOBEC3/	TargetNet
D-Mannopyra	APOBEC3/	TargetNet
L-Rhamnose	APOBEC3/	TargetNet
D-(+)-Xylose	APOBEC3/	TargetNet
D(+)-Fucose	APOBEC3/	TargetNet
D-Glucose	APOBEC3/	TargetNet
L-Arabinose	ALPL	TargetNet
L-Rhamnose	ALPL	TargetNet
D-(+)-Xylose	ALPL	TargetNet;PubChem_Literature
D(+)-Fucose	ALPL	TargetNet
L-Arabinose	ALOX15	TargetNet
L-Rhamnose	ALOX15	TargetNet
D-(+)-Xylose	ALOX15	TargetNet
D(+)-Fucose	ALOX15	TargetNet
L-Arabinose	AHR	TargetNet
D-(+)-Xylose	AHR	TargetNet
L-Arabinose	ADRA2C	TargetNet
L-Galactose	ADRA2C	TargetNet
D-Mannopyra	ADRA2C	TargetNet
L-Rhamnose	ADRA2C	TargetNet
D-(+)-Xylose	ADRA2C	TargetNet
D(+)-Fucose	ADRA2C	TargetNet

A total of 463 potential targets were obtained by searching for Astragalus polysaccharide compounds on Pubchem and HERB databases and predicting targets base on the SMILES structual formula of the coumpounds on the TargetNets platform(taking probability >0.9)

Continued from above

D-Glucose	ADRA2C	TargetNet
L-Arabinose	ADRA2B	TargetNet
L-Galactose	ADRA2B	TargetNet
D-Mannopyra	ADRA2B	TargetNet
L-Rhamnose	ADRA2B	TargetNet
D-(+)-Xylose	ADRA2B	TargetNet
D(+)-Fucose	ADRA2B	TargetNet
D-Glucose	ADRA2B	TargetNet
L-Arabinose	ADRA2A	TargetNet
L-Rhamnose	ADRA2A	TargetNet
D-(+)-Xylose	ADRA2A	TargetNet
D(+)-Fucose	ADRA2A	TargetNet
D(+)-Fucose	CDCA4	PubChem_Literature
D(+)-Fucose	CD7	PubChem_Literature
D(+)-Fucose	MBL2	PubChem_Literature
D(+)-Fucose	NT5C2	PubChem_Literature
D(+)-Fucose	ACHE	PubChem_Literature
D(+)-Fucose	BANF1	PubChem_Literature
D(+)-Fucose	CBLN1	PubChem_Literature
D(+)-Fucose	TLR4	PubChem_Literature
D(+)-Fucose	IL6	PubChem_Literature
D(+)-Fucose	AKT1	PubChem_Literature
D(+)-Fucose	TNF	PubChem_Literature
D(+)-Fucose	SGTA	PubChem_Literature
D(+)-Fucose	LTF	DrugBank
L-Arabinose	AGPS	PubChem_Literature
L-Arabinose	ALB	PubChem_Literature
L-Arabinose	ARAF	PubChem_Literature
L-Arabinose	CASP3	PubChem_Literature
L-Arabinose	CAT	PubChem_Literature
L-Arabinose	CD4	PubChem_Literature
L-Arabinose	CD8A	PubChem_Literature
L-Arabinose	FUCA1	PubChem_Literature
L-Arabinose	GALE	PubChem_Literature
L-Arabinose	GALM	PubChem_Literature
L-Arabinose	HSPD1	PubChem_Literature
L-Arabinose	IFNLR1	PubChem_Literature
L-Arabinose	IL10	PubChem_Literature
L-Arabinose	IL2	PubChem_Literature
L-Arabinose	IL4	PubChem_Literature
L-Arabinose	IL6	PubChem_Literature
L-Arabinose	INS	PubChem_Literature
L-Arabinose	TFPI	PubChem_Literature
L-Arabinose	LAD1	PubChem_Literature
L-Arabinose	LIPE	PubChem_Literature
L-Arabinose	LYZ	PubChem_Literature
L-Arabinose	MAPK14	PubChem_Literature
L-Arabinose	MGAM	PubChem_Literature
L-Arabinose	ODC1	PubChem_Literature
L-Arabinose	VDAC1	PubChem_Literature
L-Arabinose	PPP1R17	PubChem_Literature
L-Arabinose	SERPINB1(	PubChem_Literature
L-Arabinose	RAD51	PubChem_Literature
L-Arabinose	RELA	PubChem_Literature
L-Arabinose	SLC35B1	PubChem_Literature
L-Arabinose	TKT	PubChem_Literature
L-Arabinose	TLR2	PubChem_Literature
L-Arabinose	TLR4	PubChem_Literature

A total of 463 potential targets were obtained by searching for Astragalus polysaccharide compounds on Pubchem and HERB databases and predicting targets base on the SMILES structual formula of the coumpounds on the TargetNets platform(taking probability >0.9)

L-Arabinose	TNF	PubChem_Literature
L-Arabinose	XYLB	PubChem_Literature
L-Arabinose	PADI1	PubChem_Literature
L-Arabinose	GLB1	PubChem_Literature
L-Arabinose	ALDOA	PubChem_Literature
L-Arabinose	GALK1	PubChem_Literature
L-Arabinose	APLF	PubChem_BioAssay
L-Galactose	A4GALT	PubChem_Literature
L-Galactose	AGPS	PubChem_Literature
L-Galactose	ALB	PubChem_Literature
L-Galactose	ARAF	PubChem_Literature
L-Galactose	C1orf168	PubChem_Literature
L-Galactose	ATP2B1	PubChem_Literature
L-Galactose	B4GALT1	PubChem_Literature
L-Galactose	CD4	PubChem_Literature
L-Galactose	CD8A	PubChem_Literature
L-Galactose	CBLN1	PubChem_Literature
L-Galactose	COQ10A	PubChem_Literature
L-Galactose	CTSA	PubChem_Literature
L-Galactose	CYB5D2	PubChem_Literature
L-Galactose	FUCA1	PubChem_Literature
L-Galactose	GAL	PubChem_Literature
L-Galactose	GALE	PubChem_Literature
L-Galactose	GALK1	PubChem_Literature;PubChem_CTD
L-Galactose	GALNS	PubChem_Literature
L-Galactose	GBGT1	PubChem_Literature
L-Galactose	GCG	PubChem_Literature
L-Galactose	GPT	PubChem_Literature;PubChem_CTD
L-Galactose	HSP90B1	PubChem_Literature
L-Galactose	IFNLR1	PubChem_Literature
L-Galactose	CD79A	PubChem_Literature
L-Galactose	IL10	PubChem_Literature
L-Galactose	IL6	PubChem_Literature;PubChem_CTD
L-Galactose	INS	PubChem_Literature
L-Galactose	ITGAM	PubChem_Literature
L-Galactose	LGALS1	PubChem_Literature
L-Galactose	LGALS3	PubChem_Literature
L-Galactose	LGALS4	PubChem_Literature
L-Galactose	LGALS7	PubChem_Literature
L-Galactose	LYZ	PubChem_Literature
L-Galactose	CLEC10A	PubChem_Literature
L-Galactose	SERPINB1[illegible]	PubChem_Literature
L-Galactose	SELE	PubChem_Literature
L-Galactose	SI	PubChem_Literature
L-Galactose	SLC2A1	PubChem_Literature
L-Galactose	SLC2A2	PubChem_Literature;PubChem_CTD
L-Galactose	SLC35B1	PubChem_Literature
L-Galactose	SLC3A2	PubChem_Literature
L-Galactose	SLC5A1	PubChem_Literature;PubChem_CTD
L-Galactose	TF	PubChem_Literature
L-Galactose	TG	PubChem_Literature
L-Galactose	TNF	PubChem_Literature;PubChem_CTD
L-Galactose	EPX	PubChem_Literature
L-Galactose	B4GALT7	PubChem_Literature
L-Galactose	B3GNT3	PubChem_Literature
L-Galactose	HK1	PubChem_Literature
L-Galactose	UGP2	PubChem_Literature
L-Galactose	ACPP	PubChem_Literature

A total of 463 potential targets were obtained by searching for Astragalus polysaccharide compounds on Pubchem and HERB databases and predicting targets base on the SMILES structual formula of the coumpounds on the TargetNets platform(taking probability >0.9)

L-Galactose	AMY2A	PubChem_Literature
L-Galactose	MAN2B2	PubChem_Literature
L-Galactose	MANBA	PubChem_Literature
L-Galactose	HEXDC	PubChem_Literature
L-Galactose	AMBP	PubChem_Literature
L-Galactose	CST4	PubChem_Literature
L-Galactose	NGLY1	PubChem_Literature
L-Galactose	ACHE	PubChem_CTD
L-Galactose	AGER	PubChem_CTD
L-Galactose	AIF1	PubChem_CTD
L-Galactose	AKR1B3	PubChem_CTD
L-Galactose	AOX1	PubChem_CTD
L-Galactose	AQP4	PubChem_CTD
L-Galactose	ATF4	PubChem_CTD
L-Galactose	ATG5	PubChem_CTD
L-Galactose	ATG7	PubChem_CTD
L-Galactose	ATOX1	PubChem_CTD
L-Galactose	ATP5F1A	PubChem_CTD
L-Galactose	BAX	PubChem_CTD
L-Galactose	BCL2	PubChem_CTD
L-Galactose	BDNF	PubChem_CTD
L-Galactose	BECN1	PubChem_CTD
L-Galactose	CASP12	PubChem_CTD
L-Galactose	CASP3	PubChem_CTD
L-Galactose	CASP9	PubChem_CTD
L-Galactose	CAT	PubChem_CTD
L-Galactose	CCN2	PubChem_CTD
L-Galactose	CDH5	PubChem_CTD
L-Galactose	CDKN2A	PubChem_CTD
L-Galactose	CHAT	PubChem_CTD
L-Galactose	CHRM1	PubChem_CTD
L-Galactose	COX1	PubChem_CTD
L-Galactose	CXCL8	PubChem_CTD
L-Galactose	CYBB	PubChem_CTD
L-Galactose	DDIT3	PubChem_CTD
L-Galactose	DNM1L	PubChem_CTD
L-Galactose	EIF2AK3	PubChem_CTD
L-Galactose	ERN1	PubChem_CTD
L-Galactose	FGF2	PubChem_CTD
L-Galactose	GALT	PubChem_CTD
L-Galactose	GAP43	PubChem_CTD
L-Galactose	GCLC	PubChem_CTD
L-Galactose	GDNF	PubChem_CTD
L-Galactose	GFAP	PubChem_CTD
L-Galactose	GLO1	PubChem_CTD
L-Galactose	GRIN1	PubChem_CTD
L-Galactose	GRIN2B	PubChem_CTD
L-Galactose	GSS	PubChem_CTD
L-Galactose	GSTD6	PubChem_CTD
L-Galactose	GSTE7	PubChem_CTD
L-Galactose	GUSB	PubChem_CTD
L-Galactose	HIF1A	PubChem_CTD
L-Galactose	HMOX1	PubChem_CTD
L-Galactose	HSF1	PubChem_CTD
L-Galactose	HSPA5	PubChem_CTD
L-Galactose	IFNB1	PubChem_CTD
L-Galactose	IL1B	PubChem_CTD
L-Galactose	IRF3	PubChem_CTD

A total of 463 potential targets were obtained by searching for Astragalus polysaccharide compounds on Pubchem and HERB databases and predicting targets base on the SMILES structual formula of the coumpounds on the TargetNets platform(taking probability >0.9)
