# Supplementary material for: Astragalus polysaccharide promotes the regeneration of intestinal stem cells through HIF‐1 signalling pathway
Source: J Cell Mol Med. 2023 Dec 14;28(3):e18058. doi: 10.1111/jcmm.18058 (PMC10844761; doi:10.1111/jcmm.18058)
Supplement: Supplementary file 2 — Table S2. [file JCMM-28-e18058-s004.pdf]

The list of 778 genes associated with IR-induced intestinal injury were predicted via the GeneCards database

No	Symbol	Descriptio	Category	GIFtS	GC id	Score ▼
1	TP53	Tumor Prc	Protein Cc	55	GC17M00	55.82
2	IL6	Interleukir	Protein Cc	53	GC07P022	44.78
3	FLNA	Filamin A	Protein Cc	51	GC0XM15	40
4	TNF	Tumor Ne	Protein Cc	54	GC06P087	38.61
5	CTNNB1	Catenin Be	Protein Cc	56	GC03P041	35.82
6	PIK3CA	Phosphati	Protein Cc	55	GC03P179	35.18
7	ERBB2	Erb-B2 Re	Protein Cc	58	GC17P039	33.29
8	IL10	Interleukir	Protein Cc	50	GC01M20	32.89
9	IL1B	Interleukir	Protein Cc	50	GC02M11	31.12
10	RET	Ret Proto-	Protein Cc	57	GC10P043	29.81
11	PTEN	Phosphata	Protein Cc	54	GC10P093	29.18
12	TLR4	Toll Like R	Protein Cc	54	GC09P117	27.61
13	IFNG	Interferon	Protein Cc	52	GC12M06	27.1
14	TGFB1	Transform	Protein Cc	55	GC19M04	26.44
15	CXCL8	C-X-C Mc	Protein Cc	44	GC04P073	26.23
16	EGFR	Epidermal	Protein Cc	58	GC07P055	26.07
17	MIR21	MicroRNA	RNA Gene	26	GC17P059	25.47
18	AKT1	AKT Serine	Protein Cc	56	GC14M10	24.23
19	CASP3	Caspase 3	Protein Cc	52	GC04M18	23.92
20	HMOX1	Heme Oxy	Protein Cc	55	GC22P035	23.4
21	NOS2	Nitric Oxic	Protein Cc	51	GC17M02	22.95
22	ABCB1	ATP Bindir	Protein Cc	53	GC07M08	22.76
23	MSH2	MutS Hon	Protein Cc	52	GC02P047	22.7
24	PMS2	PMS1 Hor	Protein Cc	51	GC07M00	22.42
25	EP300	E1A Bindir	Protein Cc	54	GC22P041	21.9
26	MUTYH	MutY DNA	Protein Cc	48	GC01M04	21.89
27	KIT	KIT Proto-	Protein Cc	56	GC04P054	21.79
28	BRCA2	BRCA2 DN	Protein Cc	50	GC13P032	21.67
29	CDH1	Cadherin 1	Protein Cc	52	GC16P068	21.29
30	MAPK1	Mitogen-A	Protein Cc	54	GC22M02	21.11
31	TYMP	Thymidine	Protein Cc	50	GC22M05	21.01
32	VEGFA	Vascular E	Protein Cc	51	GC06P043	20.72
33	CCND1	Cyclin D1	Protein Cc	55	GC11P069	20.39
34	EGF	Epidermal	Protein Cc	54	GC04P109	20.04
35	ICAM1	Intercellula	Protein Cc	53	GC19P010	19.9
36	SREBF1	Sterol Reg	Protein Cc	48	GC17M01	19.83
37	PTGS2	Prostaglar	Protein Cc	51	GC01M18	19.75
38	STAT3	Signal Tra	Protein Cc	56	GC17M04	19.6
39	FAS	Fas Cell St	Protein Cc	53	GC10P093	19.5
40	NTRK1	Neurotrop	Protein Cc	55	GC01P156	19.32
41	MPO	Myeloperc	Protein Cc	55	GC17M05	19.04
42	CDKN2A	Cyclin Dep	Protein Cc	52	GC09M02	18.82
43	MIR34A	MicroRNA	RNA Gene	25	GC01M00	18.79
44	BAX	BCL2 Assc	Protein Cc	52	GC19P048	18.76
45	TNFRSF1A	TNF Recep	Protein Cc	52	GC12M00	18.76
46	MYC	MYC Protc	Protein Cc	54	GC08P127	18.63
47	FGFR2	Fibroblast	Protein Cc	58	GC10M12	18.61
48	PDGFRA	Platelet De	Protein Cc	56	GC04P054	18.61
49	MMP9	Matrix Me	Protein Cc	56	GC20P046	18.6
50	IL17A	Interleukir	Protein Cc	47	GC06P052	18.38
51	BDNF	Brain Deri	Protein Cc	52	GC11M02	18.37
52	IL18	Interleukir	Protein Cc	47	GC11M11	18.29
53	TGFBR2	Transform	Protein Cc	54	GC03P030	18.09
54	SERPINE1	Serpin Far	Protein Cc	52	GC07P101	17.69
55	PPARG	Peroxisom	Protein Cc	55	GC03P012	17.48
56	NGF	Nerve Grc	Protein Cc	54	GC01M11	17.33
57	MTOR	Mechanist	Protein Cc	56	GC01M01	16.96

Using the keywords "Irradiation-induced intestinal injury" ,778 related genes were found in the GeneCards.Symbol=gene name ,Description=descriptive information about the gene , Category= categories of genesGIFtS=protein annotation scoring,GC id=number given by GeneCards,Score=gene Correspondence Scoring

Continued from above

58	KRT20	Keratin 20	Protein Cc	42	GC17M04	16.93
59	CDKN1A	Cyclin Dep	Protein Cc	51	GC06P087	16.76
60	SOD1	Superoxid	Protein Cc	56	GC21P031	16.66
61	BCL2	BCL2 Apo	Protein Cc	53	GC18M06	16.48
62	NIPBL	NIPBL Coh	Protein Cc	45	GC05P036	16.38
63	SRC	SRC Proto	Protein Cc	54	GC20P037	16.14
64	XIAP	X-Linked I	Protein Cc	54	GC0XP123	16.14
65	IL1A	Interleukin	Protein Cc	47	GC02M11	16.07
66	HRAS	HRas Prot	Protein Cc	55	GC11M00	15.91
67	CALCA	Calcitonin	Protein Cc	48	GC11M01	15.79
68	HMGB1	High Mob	Protein Cc	49	GC13M03	15.66
69	CHEK2	Checkpoir	Protein Cc	56	GC22M02	15.64
70	POLD1	DNA Poly	Protein Cc	48	GC19P070	15.61
71	ELANE	Elastase, N	Protein Cc	53	GC19P003	15.57
72	SOX9	SRY-Box T	Protein Cc	48	GC17P072	15.41
73	BRCA1	BRCA1 DN	Protein Cc	52	GC17M04	15.39
74	THBD	Thrombor	Protein Cc	49	GC20M02	15.35
75	JUN	Jun Proto-	Protein Cc	50	GC01M05	15.35
76	HIF1A	Hypoxia Ir	Protein Cc	51	GC14P061	15.11
77	ENG	Endoglin	Protein Cc	51	GC09M12	15.11
78	NFKB1	Nuclear Fa	Protein Cc	55	GC04P102	14.94
79	NPY	Neuropep	Protein Cc	47	GC07P024	14.92
80	IRF1	Interferon	Protein Cc	49	GC05M13	14.54
81	STAT1	Signal Tra	Protein Cc	55	GC02M19	14.53
82	CD36	CD36 Mol	Protein Cc	52	GC07P080	14.43
83	NFE2L2	NFE2 Like	Protein Cc	53	GC02M17	14.33
84	ATM	ATM Serir	Protein Cc	55	GC11P108	14.23
85	CDKN1B	Cyclin Dep	Protein Cc	50	GC12P022	14.22
86	IGF1	Insulin Lik	Protein Cc	50	GC12M10	14.15
87	EDN1	Endothelir	Protein Cc	51	GC06P012	14.14
88	MAP2K1	Mitogen-A	Protein Cc	55	GC15P066	14.06
89	F5	Coagulatic	Protein Cc	50	GC01M16	13.97
90	HGF	Hepatocyt	Protein Cc	54	GC07M08	13.83
91	MAPK14	Mitogen-A	Protein Cc	54	GC06P087	13.75
92	PDGFRB	Platelet De	Protein Cc	56	GC05M15	13.46
93	IGF2	Insulin Lik	Protein Cc	51	GC11M00	13.3
94	SMAD7	SMAD Far	Protein Cc	48	GC18M04	13.16
95	FGF2	Fibroblast	Protein Cc	48	GC04P122	13.16
96	SMAD2	SMAD Far	Protein Cc	53	GC18M04	13.03
97	PIK3R1	Phosphoir	Protein Cc	53	GC05P068	12.96
98	FOS	Fos Proto-	Protein Cc	52	GC14P075	12.89
99	CAT	Catalase	Protein Cc	54	GC11P034	12.88
100	NOS1	Nitric Oxic	Protein Cc	51	GC12M11	12.85
101	MAPK8	Mitogen-A	Protein Cc	52	GC10P048	12.81
102	MET	MET Protc	Protein Cc	56	GC07P116	12.73
103	MMP2	Matrix Me	Protein Cc	55	GC16P055	12.7
104	MIR155	MicroRNA	RNA Gene	23	GC21P025	12.6
105	MMP1	Matrix Me	Protein Cc	54	GC11M10	12.52
106	CASP10	Caspase 1	Protein Cc	50	GC02P201	12.46
107	HSPA4	Heat Shoc	Protein Cc	45	GC05P133	12.46
108	FASLG	Fas Liganc	Protein Cc	50	GC01P172	12.45
109	NOTCH1	Notch Rec	Protein Cc	55	GC09M13	12.37
110	GAL	Galanin Ar	Protein Cc	47	GC11P070	12.35
111	CALR	Calreticuli	Protein Cc	54	GC19P012	12.32
112	MDM2	MDM2 Pro	Protein Cc	55	GC12P068	12.24
113	RAC1	Rac Family	Protein Cc	51	GC07P006	12.15
114	BMP6	Bone Mor	Protein Cc	46	GC06P007	11.91
115	FGF7	Fibroblast	Protein Cc	46	GC15P049	11.91

Using the keywords "Irradiation-induced intestinal injury" ,778 related genes were found in the GeneCards.Symbol=gene name ,Description=descriptive information about the gene , Category= categories of genesGIFtS=protein annotation scoring,GC id=number given by GeneCards,Score=gene Correspondence Scoring

116	TERT	Telomeras	Protein Cc	53	GC05M00	11.9
117	KRT7	Keratin 7	Protein Cc	44	GC12P052	11.89
118	EPO	Erythropo	Protein Cc	47	GC07P10C	11.85
119	CASP8	Caspase 8	Protein Cc	55	GC02P201	11.85
120	CYCS	Cytochron	Protein Cc	51	GC07M02	11.82
121	HPRT1	Hypoxantl	Protein Cc	51	GC0XP134	11.82
122	SOD2	Superoxid	Protein Cc	51	GC06M15	11.77
123	ELN	Elastin	Protein Cc	45	GC07P074	11.72
124	IL2RA	Interleukir	Protein Cc	54	GC10M00	11.67
125	PARP1	Poly(ADP-	Protein Cc	53	GC01M22	11.66
126	HLA-A	Major Hist	Protein Cc	48	GC06P087	11.64
127	CASP1	Caspase 1	Protein Cc	51	GC11M10	11.53
128	IL7	Interleukir	Protein Cc	47	GC08M07	11.48
129	MAPK3	Mitogen-/	Protein Cc	51	GC16M03	11.25
130	IL33	Interleukir	Protein Cc	41	GC09P007	11.2
131	DPP4	Dipeptidyl	Protein Cc	53	GC02M16	11.14
132	BCL2L1	BCL2 Like	Protein Cc	50	GC20M03	11.11
133	KLF4	KLF Transc	Protein Cc	48	GC09M10	11.1
134	MITF	Melanocyt	Protein Cc	50	GC03P069	11.06
135	CP	Ceruloplas	Protein Cc	51	GC03M14	11.03
136	KITLG	KIT Ligand	Protein Cc	48	GC12M08	11
137	HSPB1	Heat Shoc	Protein Cc	54	GC07P076	10.99
138	SMC1A	Structural	Protein Cc	50	GC0XM05	10.97
139	RELA	RELA Prot	Protein Cc	54	GC11M06	10.86
140	CXCL12	C-X-C Mc	Protein Cc	46	GC10M04	10.82
141	MIR451A	MicroRNA	RNA Gene	20	GC17M02	10.76
142	SPTAN1	Spectrin A	Protein Cc	50	GC09P128	10.72
143	BUB1B	BUB1 Mitc	Protein Cc	52	GC15P04C	10.67
144	MYO9B	Myosin IXI	Protein Cc	46	GC19P069	10.63
145	MMP3	Matrix Me	Protein Cc	53	GC11M10	10.61
146	PLA2G4A	Phospholi	Protein Cc	51	GC01P186	10.58
147	NFKBIA	NFKB Inhil	Protein Cc	52	GC14M03	10.55
148	PRKCA	Protein Kir	Protein Cc	53	GC17P066	10.54
149	CASP9	Caspase 9	Protein Cc	49	GC01M01	10.51
150	PIK3CG	Phosphati	Protein Cc	50	GC07P106	10.47
152	YAP1	Yes1 Asso	Protein Cc	50	GC11P102	10.46
151	MIR200C	MicroRNA	RNA Gene	26	GC12P022	10.46
153	PECAM1	Platelet Ar	Protein Cc	43	GC17M06	10.38
154	MIR17	MicroRNA	RNA Gene	23	GC13P091	10.37
155	GAPDH	Glyceralde	Protein Cc	52	GC12P022	10.31
156	VCAM1	Vascular C	Protein Cc	48	GC01P10C	10.11
157	CDKN2B	Cyclin Der	Protein Cc	49	GC09M02	10.08
158	HSP90AA1	Heat Shoc	Protein Cc	52	GC14M10	10.06
159	CAV1	Caveolin 1	Protein Cc	51	GC07P116	10.06
160	GSTP1	Glutathior	Protein Cc	53	GC11P067	10.03
161	MYCN	MYCN Prc	Protein Cc	50	GC02P015	10.03
162	TF	Transferrir	Protein Cc	53	GC03P134	9.96
163	PTGS1	Prostaglar	Protein Cc	49	GC09P122	9.88
164	DNMT1	DNA Meth	Protein Cc	55	GC19M01	9.82
165	GH1	Growth Hc	Protein Cc	46	GC17M06	9.8
166	TRPV4	Transient	Protein Cc	51	GC12M10	9.77
167	MAPK10	Mitogen-/	Protein Cc	51	GC04M08	9.76
168	FERMT1	FERM Dor	Protein Cc	45	GC20M00	9.74
169	CDK4	Cyclin Der	Protein Cc	56	GC12M05	9.7
170	MMP12	Matrix Me	Protein Cc	50	GC11M10	9.65
171	TLR9	Toll Like R	Protein Cc	48	GC03M05	9.65
172	RB1	RB Transc	Protein Cc	50	GC13P048	9.65
173	SP1	Sp1 Trans	Protein Cc	49	GC12P053	9.63

Using the keywords "Irradiation-induced intestinal injury" ,778 related genes were found in the GeneCards.Symbol=gene name ,Description=descriptive information about the gene , Category= categories of genesGIFtS=protein annotation scoring,GC id=number given by GeneCards,Score=gene Correspondence Scoring

174	CXCR3	C-X-C Mo	Protein Co	46	GC0XM07	9.56
175	IGFBP3	Insulin Like	Protein Co	50	GC07M04	9.5
176	SIRT1	Sirtuin 1	Protein Co	52	GC10P067	9.49
177	PRL	Prolactin	Protein Co	45	GC06M02	9.47
178	CCL3	C-C Motif	Protein Co	44	GC17M03	9.46
179	CREB1	CAMP Res	Protein Co	51	GC02P207	9.44
180	PTK2	Protein Ty	Protein Co	50	GC08M14	9.38
181	SMC3	Structural	Protein Co	49	GC10P110	9.34
182	FN1	Fibronecti	Protein Co	52	GC02M21	9.3
183	PCNA	Proliferatir	Protein Co	53	GC20M00	9.3
184	RAF1	Raf-1 Prot	Protein Co	57	GC03M01	9.27
185	MCL1	MCL1 Apo	Protein Co	50	GC01M15	9.23
186	SOCS1	Suppresso	Protein Co	46	GC16M01	9.21
187	CDK1	Cyclin Dep	Protein Co	50	GC10P060	9.18
188	ABCC3	ATP Bindir	Protein Co	49	GC17P050	9.16
189	CDK2	Cyclin Dep	Protein Co	54	GC12P055	9.16
190	AURKA	Aurora Kir	Protein Co	53	GC20M05	9.08
191	HSPA8	Heat Shoc	Protein Co	51	GC11M12	9.06
192	CD274	CD274 Mo	Protein Co	47	GC09P005	9.05
193	POMC	Proopiom	Protein Co	51	GC02M02	9.04
194	CD40	CD40 Mol	Protein Co	52	GC20P046	8.98
195	NQO1	NAD(P)H (	Protein Co	50	GC16M06	8.91
196	ABL1	ABL Proto	Protein Co	55	GC09P130	8.89
197	PRKCD	Protein Kir	Protein Co	55	GC03P053	8.89
198	HDAC4	Histone D	Protein Co	54	GC02M23	8.85
199	SDC1	Syndecan	Protein Co	45	GC02M02	8.84
200	ESR1	Estrogen F	Protein Co	56	GC06P151	8.77
201	NINJ1	Ninjurin 1	Protein Co	43	GC09M09	8.74
202	MALAT1	Metastasis	RNA Gene	26	GC11P070	8.71
203	WWOX	WW Doma	Protein Co	48	GC16P078	8.62
204	THBS1	Thrombos	Protein Co	48	GC15P039	8.56
205	LIMA1	LIM Doma	Protein Co	41	GC12M05	8.51
206	C1S	Complem	Protein Co	50	GC12P022	8.46
207	ANXA5	Annexin A	Protein Co	48	GC04M12	8.44
208	CLU	Clusterin	Protein Co	50	GC08M02	8.43
209	HMOX2	Heme Oxy	Protein Co	48	GC16P004	8.42
210	TXN	Thioredox	Protein Co	48	GC09M11	8.41
211	WRN	WRN Rec(	Protein Co	48	GC08P031	8.37
212	GJA1	Gap Junct	Protein Co	53	GC06P121	8.34
213	DCN	Decorin	Protein Co	50	GC12M09	8.34
214	GNRH1	Gonadotro	Protein Co	45	GC08M02	8.33
215	NPM1	Nucleoph	Protein Co	53	GC05P171	8.32
216	MMP7	Matrix Me	Protein Co	50	GC11M10	8.3
217	NBN	Nibrin	Protein Co	50	GC08M08	8.27
218	TNFSF10	TNF Super	Protein Co	47	GC03M17	8.26
219	EGR1	Early Grow	Protein Co	50	GC05P138	8.21
220	TGFB3	Transform	Protein Co	52	GC14M07	8.2
221	KAT5	Lysine Ace	Protein Co	51	GC11P065	8.19
222	BLM	BLM RecQ	Protein Co	51	GC15P090	8.18
223	SNAI1	Snail Fami	Protein Co	48	GC20P049	8.18
224	GSR	Glutathior	Protein Co	52	GC08M03	8.17
225	NTRK2	Neurotrop	Protein Co	56	GC09P084	8.17
226	TGFB2	Transform	Protein Co	54	GC01P218	8.16
227	BAK1	BCL2 Anta	Protein Co	47	GC06M03	8.08
228	IL3	Interleukir	Protein Co	47	GC05P132	8.03
229	CDKN3	Cyclin Dep	Protein Co	44	GC14P054	7.99
230	APEX1	Apurinic/A	Protein Co	47	GC14P020	7.93
231	IL6R	Interleukir	Protein Co	53	GC01P154	7.92

Using the keywords "Irradiation-induced intestinal injury" ,778 related genes were found in the GeneCards.Symbol=gene name ,Description=descriptive information about the gene , Category= categories of genesGIFtS=protein annotation scoring,GC id=number given by GeneCards,Score=gene Correspondence Scoring

232	TNFRSF10	TNF Recep	Protein Cc	52	GC08M02	7.91
233	PPIG	Peptidylpr	Protein Cc	43	GC02P169	7.83
234	RANBP2	RAN Bindi	Protein Cc	49	GC02P108	7.78
235	PTK2B	Protein Ty	Protein Cc	51	GC08P027	7.73
236	TLR3	Toll Like R	Protein Cc	54	GC04P186	7.68
237	NRG1	Neureguli	Protein Cc	51	GC08P031	7.65
238	MIR205	MicroRNA	RNA Gene	23	GC01P209	7.62
239	AKT2	AKT Serine	Protein Cc	56	GC19M04	7.57
240	C1R	Complem	Protein Cc	49	GC12M00	7.55
241	CD80	CD80 Mol	Protein Cc	48	GC03M11	7.54
242	IKBKB	Inhibitor C	Protein Cc	56	GC08P042	7.49
243	XBP1	X-Box Bin	Protein Cc	47	GC22M02	7.47
244	GADD45A	Growth Ar	Protein Cc	48	GC01P067	7.42
245	IGF1R	Insulin Like	Protein Cc	57	GC15P098	7.4
246	TP63	Tumor Pro	Protein Cc	50	GC03P189	7.25
247	WNT1	Wnt Famil	Protein Cc	47	GC12P049	7.25
248	TRPV1	Transient	Protein Cc	50	GC17M00	7.22
249	BECN1	Beclin 1	Protein Cc	48	GC17M04	7.2
250	E2F1	E2F Trans	Protein Cc	45	GC20M03	7.2
251	BBC3	BCL2 Bind	Protein Cc	42	GC19M04	7.13
252	C11orf65	Chromoso	Protein Cc	34	GC11M10	7.11
253	BIRC5	Baculovira	Protein Cc	48	GC17P078	7.1
254	EWSR1	EWS RNA	Protein Cc	47	GC22P040	7.08
255	LYZ	Lysozyme	Protein Cc	49	GC12P069	7.05
256	EZH2	Enhancer	Protein Cc	56	GC07M14	7.05
257	FLII	FLII Actin	Protein Cc	45	GC17M01	7.05
258	ERCC6	ERCC Exci	Protein Cc	48	GC10M04	7.01
260	PLAUR	Plasminog	Protein Cc	47	GC19M04	7
259	MIR328	MicroRNA	RNA Gene	22	GC16M06	7
261	KRT5	Keratin 5	Protein Cc	48	GC12M05	6.95
262	RBL2	RB Transc	Protein Cc	47	GC16P053	6.9
263	ST14	ST14 Tran	Protein Cc	51	GC11P130	6.85
264	PML	PML Nucle	Protein Cc	48	GC15P073	6.83
265	BCL2L11	BCL2 Like	Protein Cc	47	GC02P111	6.81
266	IFI27	Interferon	Protein Cc	40	GC14P094	6.79
267	TNFRSF10	TNF Recep	Protein Cc	47	GC08M02	6.79
268	XRCC1	X-Ray Rep	Protein Cc	46	GC19M04	6.76
269	PSMD4	Proteason	Protein Cc	46	GC01P151	6.75
270	TYR	Tyrosinase	Protein Cc	53	GC11P089	6.74
271	CHUK	Compone	Protein Cc	54	GC10M10	6.71
272	ERN1	Endoplasn	Protein Cc	48	GC17M06	6.69
273	TCOF1	Treacle Ri	Protein Cc	44	GC05P150	6.66
274	IKBKG	Inhibitor C	Protein Cc	51	GC0XP154	6.65
276	EIF2AK3	Eukaryotic	Protein Cc	51	GC02M08	6.64
275	MIR23A	MicroRNA	RNA Gene	23	GC19M01	6.64
277	MMP13	Matrix Me	Protein Cc	54	GC11M10	6.62
278	OGG1	8-Oxogua	Protein Cc	50	GC03P012	6.6
279	GCLC	Glutamate	Protein Cc	47	GC06M05	6.57
280	IL6ST	Interleukir	Protein Cc	52	GC05M05	6.54
281	BAD	BCL2 Asso	Protein Cc	47	GC11M09	6.53
282	ADORA2B	Adenosine	Protein Cc	50	GC17P017	6.51
283	TERC	Telomeras	RNA Gene	31	GC03M16	6.51
284	DNM1L	Dynamin 1	Protein Cc	50	GC12P032	6.45
285	MMP8	Matrix Me	Protein Cc	51	GC11M10	6.44
286	TYMS	Thymidyla	Protein Cc	51	GC18P000	6.44
287	MIR320A	MicroRNA	RNA Gene	23	GC08M02	6.42
288	SEPTIN4	Septin 4	Protein Cc	39	GC17M05	6.4
289	ATP2B1	ATPase Pla	Protein Cc	46	GC12M08	6.39

Using the keywords "Irradiation-induced intestinal injury" ,778 related genes were found in the GeneCards.Symbol=gene name ,Description=descriptive information about the gene , Category= categories of genesGIFtS=protein annotation scoring,GC id=number given by GeneCards,Score=gene Correspondence Scoring

290	ATF3	Activating	Protein Cc	47	GC01P212	6.39
291	COX5A	Cytochron	Protein Cc	45	GC15M07·	6.38
292	CASP2	Caspase 2	Protein Cc	50	GC07P148	6.37
293	BID	BH3 Intera	Protein Cc	47	GC22M01	6.36
294	DNASE1	Deoxyribo	Protein Cc	45	GC16P003	6.36
295	IFNB1	Interferon	Protein Cc	47	GC09M02	6.35
296	TUG1	Taurine U	Protein Cc	25	GC22P030	6.27
297	ERCC2	ERCC Exci	Protein Cc	50	GC19M04	6.26
298	CCN4	Cellular C	Protein Cc	41	GC08P133	6.24
299	SNAP25	Synaptosc	Protein Cc	51	GC20P010	6.22
300	CSNK2B	Casein Kin	Protein Cc	50	GC06P087	6.22
301	KRT14	Keratin 14	Protein Cc	49	GC17M04	6.21
302	AMBP	Alpha-1-M	Protein Cc	47	GC09M11	6.14
303	HDAC1	Histone D	Protein Cc	52	GC01P032	6.13
304	LGALS1	Galectin 1	Protein Cc	45	GC22P037	6.11
305	CDKN1C	Cyclin Dep	Protein Cc	47	GC11M00	6.1
306	SIN3A	SIN3 Trans	Protein Cc	48	GC15M07	6.1
307	PLCG1	Phospholi	Protein Cc	50	GC20P041	6.07
308	NOX1	NADPH O	Protein Cc	45	GC0XM10	6.06
309	MAPK13	Mitogen-/	Protein Cc	51	GC06P087	6.05
310	CASP7	Caspase 7	Protein Cc	52	GC10P113	6.05
311	CHEK1	Checkpoir	Protein Cc	52	GC11P125	6.03
312	LCK	LCK Proto	Protein Cc	55	GC01P032	6.02
313	IL1RAPL2	Interleukir	Protein Cc	42	GC0XP10	6.01
314	PTAFR	Platelet A	Protein Cc	47	GC01M02	6.01
315	LPO	Lactopero	Protein Cc	42	GC17P058	5.99
316	UFD1	Ubiquitin I	Protein Cc	45	GC22M01	5.98
317	JUND	JunD Prot	Protein Cc	44	GC19M01	5.96
318	FANCL	FA Compl	Protein Cc	48	GC02M05	5.92
319	AIFM1	Apoptosis	Protein Cc	51	GC0XM13	5.9
320	RUNX2	RUNX Fan	Protein Cc	49	GC06P088	5.89
321	MAP2K2	Mitogen-/	Protein Cc	56	GC19M00	5.89
322	PPARD	Peroxisom	Protein Cc	48	GC06P087	5.88
323	FYN	FYN Proto	Protein Cc	49	GC06M11	5.84
324	UBE3A	Ubiquitin I	Protein Cc	50	GC15M02	5.82
325	ITGAV	Integrin S	Protein Cc	51	GC02P186	5.81
326	RAG2	Recombin	Protein Cc	44	GC11M03	5.79
327	HLA-G	Major Hist	Protein Cc	46	GC06P087	5.79
328	TOP1	DNA Topc	Protein Cc	51	GC20P041	5.75
329	RAD51	RAD51 Re	Protein Cc	54	GC15P040	5.74
330	SATB2	SATB Hon	Protein Cc	45	GC02M19	5.7
331	SPARC	Secreted I	Protein Cc	52	GC05M15	5.7
332	IL12B	Interleukir	Protein Cc	50	GC05M15	5.69
333	PRKCH	Protein Kir	Protein Cc	52	GC14P061	5.69
334	RPL5	Ribosoma	Protein Cc	50	GC01P092	5.67
335	CXCL5	C-X-C Mc	Protein Cc	43	GC04M07	5.64
336	MYH9	Myosin He	Protein Cc	51	GC22M03	5.59
337	LOX	Lysyl Oxid	Protein Cc	50	GC05M12	5.59
338	MAP3K1	Mitogen-/	Protein Cc	52	GC05P056	5.55
339	NDUFA13	NADH:Ubi	Protein Cc	46	GC19P019	5.54
340	DIABLO	Diablo IAF	Protein Cc	49	GC12M12	5.52
341	IL18R1	Interleukir	Protein Cc	46	GC02P102	5.48
342	PRAP1	Proline Ric	Protein Cc	36	GC10P133	5.46
343	SMPD1	Sphingom	Protein Cc	52	GC11P006	5.46
344	BTRC	Beta-Tran	Protein Cc	48	GC10P101	5.46
345	CCNB1	Cyclin B1	Protein Cc	49	GC05P069	5.41
346	UBAC2	UBA Dom	Protein Cc	40	GC13P099	5.41
347	ZEB1	Zinc Finge	Protein Cc	51	GC10P031	5.4

Using the keywords "Irradiation-induced intestinal injury" ,778 related genes were found in the GeneCards.Symbol=gene name ,Description=descriptive information about the gene , Category= categories of genesGIFtS=protein annotation scoring,GC id=number given by GeneCards,Score=gene Correspondence Scoring

348	AQP3	Aquaporir	Protein Cc	49	GC09M03	5.38
349	GLUL	Glutamate	Protein Cc	51	GC01M18	5.34
350	UBE2A	Ubiquitin (	Protein Cc	47	GC0XP119	5.3
351	PXN	Paxillin	Protein Cc	47	GC12M12	5.26
352	UGCG	UDP-Gluc	Protein Cc	46	GC09P111	5.23
353	GPX4	Glutathior	Protein Cc	48	GC19P001	5.23
354	DDB1	Damage S	Protein Cc	47	GC11M09	5.17
355	ACP1	Acid Phos	Protein Cc	45	GC02P000	5.17
356	BMI1	BMI1 Prot	Protein Cc	45	GC10P022	5.17
357	EIF4EBP1	Eukaryotic	Protein Cc	49	GC08P038	5.16
358	HDAC2	Histone D	Protein Cc	54	GC06M11	5.15
359	FMOD	Fibromodu	Protein Cc	45	GC01M20	5.15
360	RAD51C	RAD51 Pa	Protein Cc	46	GC17P058	5.12
361	FOSL2	FOS Like 2	Protein Cc	44	GC02P028	5.11
362	FADD	Fas Associ	Protein Cc	48	GC11P070	5.08
363	FARSB	Phenylalar	Protein Cc	48	GC02M22	5.06
364	MRE11	MRE11 Hc	Protein Cc	50	GC11M09	5.05
365	MAP3K5	Mitogen-/	Protein Cc	50	GC06M13	5.05
366	CCND2	Cyclin D2	Protein Cc	51	GC12P022	5.04
367	ASAH2	N-Acylspl	Protein Cc	44	GC10M05	5.03
368	RAD51B	RAD51 Pa	Protein Cc	39	GC14P067	5.02
369	MAPK9	Mitogen-/	Protein Cc	52	GC05M18	5
370	CASP6	Caspase 6	Protein Cc	51	GC04M10	5
371	HNRNPU	Heteroger	Protein Cc	45	GC01M24	4.98
372	DAXX	Death Dor	Protein Cc	45	GC06M03	4.96
373	TP73	Tumor Prc	Protein Cc	48	GC01P003	4.95
374	BCR	BCR Activ	Protein Cc	55	GC22P023	4.93
375	PARK7	Parkinsoni	Protein Cc	50	GC01P008	4.93
376	DCT	Dopachro	Protein Cc	48	GC13M09	4.92
377	H2AX	H2A.X Var	Protein Cc	45	GC11M11	4.91
378	ERCC1	ERCC Exci	Protein Cc	48	GC19M06	4.91
379	MAP2K4	Mitogen-/	Protein Cc	48	GC17P012	4.9
380	MIR130A	MicroRNA	RNA Gene	23	GC11P057	4.89
381	GTF2I	General Tı	Protein Cc	44	GC07P075	4.88
382	MADD	MAP Kinas	Protein Cc	46	GC11P047	4.87
383	MAPKAPK	MAPK Act	Protein Cc	50	GC01P206	4.84
384	LIG3	DNA Ligas	Protein Cc	48	GC17P034	4.84
385	CCN1	Cellular Cc	Protein Cc	41	GC01P085	4.84
386	SOX4	SRY-Box T	Protein Cc	45	GC06P021	4.84
387	FUS	FUS RNA I	Protein Cc	47	GC16P031	4.78
388	UBE2L3	Ubiquitin (	Protein Cc	47	GC22P021	4.77
389	FANCI	FA Compl	Protein Cc	45	GC15P089	4.76
390	BIRC2	Baculovira	Protein Cc	48	GC11P102	4.75
391	H4C16	H4 Histon	Protein Cc	39	GC12M02	4.75
392	BAG1	BAG Coch	Protein Cc	43	GC09M03	4.74
393	CD58	CD58 Mol	Protein Cc	43	GC01M11	4.74
394	SKP2	S-Phase K	Protein Cc	46	GC05P036	4.69
395	EIF2S1	Eukaryotic	Protein Cc	46	GC14P067	4.69
396	UBC	Ubiquitin (	Protein Cc	45	GC12M12	4.69
397	CFL1	Cofilin 1	Protein Cc	48	GC11M06	4.67
398	ASAH1	N-Acylspl	Protein Cc	51	GC08M01	4.67
399	WWTR1	WW Dom	Protein Cc	43	GC03M14	4.66
400	PTPA	Protein Ph	Protein Cc	44	GC09P129	4.65
401	ACACA	Acetyl-Co	Protein Cc	50	GC17M03	4.64
402	FANCD2	FA Compl	Protein Cc	48	GC03P010	4.64
403	JUNB	JunB Prot	Protein Cc	44	GC19P012	4.62
404	HLA-E	Major Hist	Protein Cc	44	GC06P087	4.6
405	ABCC4	ATP Bindi	Protein Cc	48	GC13M09	4.6

Using the keywords "Irradiation-induced intestinal injury" ,778 related genes were found in the GeneCards.Symbol=gene name ,Description=descriptive information about the gene , Category= categories of genesGIFtS=protein annotation scoring,GC id=number given by GeneCards,Score=gene Correspondence Scoring

406	RPL26	Ribosoma	Protein Co	45	GC17M00	4.59
407	JUP	Junction P	Protein Co	50	GC17M04	4.53
408	MIR181A2	MicroRNA	RNA Gene	22	GC09P124	4.53
409	PIM1	Pim-1 Pro	Protein Co	52	GC06P087	4.52
410	RBBP8	RB Binding	Protein Co	45	GC18P022	4.52
411	DHX9	DExH-Box	Protein Co	44	GC01P182	4.5
412	CAPN1	Calpain 1	Protein Co	53	GC11P070	4.45
413	FANCA	FA Compl	Protein Co	51	GC16M08	4.45
414	DUSP1	Dual Spec	Protein Co	49	GC05M17	4.44
415	SP3	Sp3 Trans	Protein Co	45	GC02M17	4.43
416	GHR	Growth H	Protein Co	49	GC05P042	4.39
417	PON2	Paraoxona	Protein Co	46	GC07M09	4.38
418	YWHAG	Tyrosine 3	Protein Co	51	GC07M07	4.37
419	CCKAR	Cholecyst	Protein Co	48	GC04M02	4.37
420	UCP2	Uncouplin	Protein Co	49	GC11M07	4.35
421	CCNE1	Cyclin E1	Protein Co	51	GC19P029	4.35
422	PEPD	Peptidase	Protein Co	48	GC19M03	4.34
423	EIF2AK2	Eukaryotic	Protein Co	51	GC02M03	4.33
424	MSI2	Musashi R	Protein Co	42	GC17P057	4.32
425	TRPM2	Transient	Protein Co	44	GC21P044	4.31
426	PRKN	Parkin RBI	Protein Co	47	GC06M16	4.29
427	CCNA2	Cyclin A2	Protein Co	48	GC04M12	4.22
428	BLK	BLK Proto	Protein Co	52	GC08P011	4.21
429	TEAD1	TEA Doma	Protein Co	49	GC11P012	4.2
430	HDAC6	Histone D	Protein Co	56	GC0XP048	4.19
431	PRKDC	Protein Ki	Protein Co	53	GC08M04	4.19
432	HSP90AB1	Heat Shoc	Protein Co	50	GC06P044	4.19
433	MTA1	Metastasis	Protein Co	46	GC14P105	4.18
434	GRB2	Growth Fa	Protein Co	48	GC17M07	4.17
435	SPHK1	Sphingosi	Protein Co	49	GC17P076	4.17
436	XPC	XPC Comp	Protein Co	48	GC03M02	4.16
437	TBL2	Transduci	Protein Co	43	GC07M07	4.16
438	SPTBN1	Spectrin B	Protein Co	48	GC02P054	4.1
439	UBE2T	Ubiquitin	Protein Co	47	GC01M20	4.05
440	APAF1	Apoptotic	Protein Co	48	GC12P098	4.05
441	KRT1	Keratin 1	Protein Co	49	GC12M05	4.04
442	DDB2	Damage S	Protein Co	48	GC11P047	4.03
443	PIDD1	P53-Induc	Protein Co	39	GC11M00	4.02
444	BCL2L14	BCL2 Like	Protein Co	41	GC12P012	4.01
445	HADHB	Hydroxyac	Protein Co	50	GC02P026	4.01
446	CDC25A	Cell Divisi	Protein Co	50	GC03M04	3.98
447	SNRNP70	Small Nuc	Protein Co	42	GC19P049	3.98
448	USP7	Ubiquitin	Protein Co	52	GC16M00	3.97
449	MSI1	Musashi R	Protein Co	43	GC12M12	3.97
450	FOSL1	FOS Like 1	Protein Co	47	GC11M09	3.96
451	TRIM28	Tripartite	Protein Co	46	GC19P058	3.96
452	TTF2	Transcript	Protein Co	42	GC01P117	3.95
453	ERCC4	ERCC Exci	Protein Co	48	GC16P013	3.88
454	RECQL4	RecQ Like	Protein Co	43	GC08M14	3.88
455	TOP2A	DNA Topo	Protein Co	54	GC17M04	3.87
456	TSC22D1	TSC22 Do	Protein Co	43	GC13M04	3.86
457	MAPRE1	Microtubu	Protein Co	47	GC20P032	3.85
458	PMAIP1	Phorbol-1	Protein Co	44	GC18P059	3.83
459	GNAI3	G Protein	Protein Co	48	GC01P109	3.83
460	MAD2L2	Mitotic Ar	Protein Co	45	GC01M01	3.82
461	OAT	Ornithine	Protein Co	50	GC10M12	3.79
462	ATR	ATR Serin	Protein Co	54	GC03M14	3.79
463	TPD52	Tumor Pro	Protein Co	43	GC08M08	3.77

Using the keywords "Irradiation-induced intestinal injury" ,778 related genes were found in the GeneCards.Symbol=gene name ,Description=descriptive information about the gene , Category= categories of genesGIFtS=protein annotation scoring,GC id=number given by GeneCards,Score=gene Correspondence Scoring

464	BCL2L2	BCL2 Like	Protein Cc	45	GC14P033	3.73
465	MAP2K3	Mitogen-/	Protein Cc	51	GC17P057	3.72
466	KRT17	Keratin 17	Protein Cc	47	GC17M04	3.71
467	MSL1	MSL Comp	Protein Cc	35	GC17P058	3.71
468	XRCC6	X-Ray Rep	Protein Cc	48	GC22P041	3.71
469	GFER	Growth Fa	Protein Cc	48	GC16P001	3.69
470	DCLRE1C	DNA Cros	Protein Cc	47	GC10M01	3.69
471	RBM3	RNA Bindi	Protein Cc	40	GC0XP048	3.67
472	MDM4	MDM4 Re	Protein Cc	47	GC01P204	3.65
473	GPX3	Glutathior	Protein Cc	44	GC05P150	3.64
474	XRCC2	X-Ray Rep	Protein Cc	43	GC07M15	3.64
475	LYN	LYN Proto	Protein Cc	51	GC08P055	3.63
476	KRT10	Keratin 10	Protein Cc	45	GC17M04	3.63
477	RAD52	RAD52 Hc	Protein Cc	43	GC12M00	3.62
478	XRCC5	X-Ray Rep	Protein Cc	47	GC02P216	3.6
479	MRTFA	Myocardir	Protein Cc	43	GC22M06	3.6
480	MX1	MX Dynar	Protein Cc	43	GC21P041	3.59
481	CMA1	Chymase	Protein Cc	47	GC14M02	3.57
482	SRSF1	Serine Anc	Protein Cc	44	GC17M05	3.56
483	XPA	XPA, DNA	Protein Cc	47	GC09M09	3.55
484	RANBP1	RAN Bindi	Protein Cc	46	GC22P020	3.54
485	USP9X	Ubiquitin	Protein Cc	51	GC0XP041	3.52
486	CXCL14	C-X-C Mc	Protein Cc	39	GC05M13	3.52
487	EFTUD2	Elongatior	Protein Cc	45	GC17M04	3.5
488	NONO	Non-POU	Protein Cc	48	GC0XP071	3.49
489	RUVBL1	RuvB Like	Protein Cc	47	GC03M12	3.49
490	RBPJ	Recombin	Protein Cc	49	GC04P026	3.47
491	CTBP2	C-Termina	Protein Cc	46	GC10M12	3.47
492	KRT16	Keratin 16	Protein Cc	45	GC17M04	3.46
493	NFIB	Nuclear Fa	Protein Cc	45	GC09M01	3.46
494	CRYAA	Crystallin	Protein Cc	48	GC21P043	3.45
495	ARF4	ADP Ribos	Protein Cc	44	GC03M05	3.45
496	RPS7	Ribosoma	Protein Cc	44	GC02P003	3.42
497	ING1	Inhibitor C	Protein Cc	45	GC13P110	3.4
498	NUPR1	Nuclear Pr	Protein Cc	37	GC16M02	3.36
499	NRF1	Nuclear R	Protein Cc	46	GC07P129	3.35
500	DHX30	DExH-Box	Protein Cc	43	GC03P047	3.35
501	PSMC6	Proteasom	Protein Cc	43	GC14P052	3.32
502	RFC4	Replicatio	Protein Cc	45	GC03M18	3.32
503	MANF	Mesencep	Protein Cc	43	GC03P051	3.3
504	CDC45	Cell Divisi	Protein Cc	46	GC22P019	3.29
505	YWHAZ	Tyrosine 3	Protein Cc	48	GC08M10	3.25
506	PRDX1	Peroxiredc	Protein Cc	52	GC01M04	3.25
507	MAP2K6	Mitogen-/	Protein Cc	50	GC17P069	3.25
508	PDCD5	Programm	Protein Cc	42	GC19P032	3.24
509	HAS2	Hyalurona	Protein Cc	43	GC08M12	3.23
510	KLK1	Kallikrein	Protein Cc	49	GC19M05	3.23
511	PTTG1	PTTG1 Re	Protein Cc	43	GC05P160	3.23
512	HM13	Histocomp	Protein Cc	41	GC20P031	3.23
513	NR2F1	Nuclear R	Protein Cc	47	GC05P093	3.22
514	TP53BP1	Tumor Prc	Protein Cc	46	GC15M04	3.22
515	CUL4B	Cullin 4B	Protein Cc	45	GC0XM12	3.2
516	MIR219A1	MicroRNA	RNA Gene	23	GC06P033	3.17
517	YES1	YES Proto	Protein Cc	51	GC18M00	3.15
518	TAB1	TGF-Beta	Protein Cc	45	GC22P041	3.14
519	RAN	RAN, Men	Protein Cc	47	GC12P130	3.13
520	SULF2	Sulfatase	Protein Cc	41	GC20M04	3.13
521	LY86	Lymphocy	Protein Cc	41	GC06P006	3.12

Using the keywords "Irradiation-induced intestinal injury" ,778 related genes were found in the GeneCards.Symbol=gene name ,Description=descriptive information about the gene , Category= categories of genesGIFtS=protein annotation scoring,GC id=number given by GeneCards,Score=gene Correspondence Scoring

522	TP53BP2	Tumor Prc	Protein Cc	43	GC01M22	3.12
523	XRCC3	X-Ray Rep	Protein Cc	45	GC14M10	3.1
524	HYAL1	Hyaluroni	Protein Cc	50	GC03M05	3.09
525	SENP3	SUMO Sp	Protein Cc	42	GC17P011	3.09
526	ATF1	Activating	Protein Cc	48	GC12P05C	3.08
527	HUWE1	HECT, UB/	Protein Cc	47	GC0XM05	3.08
528	PDCD6IP	Programm	Protein Cc	45	GC03P033	3.07
529	ATG5	Autophag	Protein Cc	47	GC06M10	3.06
530	HNRNPA1	Heteroger	Protein Cc	50	GC12P054	3.06
531	MAPK12	Mitogen-/	Protein Cc	51	GC22M05	3.05
532	PRPF8	Pre-MRN/	Protein Cc	44	GC17M00	3.05
533	EEF1A1	Eukaryotic	Protein Cc	45	GC06M07	3.04
534	ATXN10	Ataxin 10	Protein Cc	45	GC22P045	3.02
535	BRAT1	BRCA1 As	Protein Cc	40	GC07M00	3.02
536	RAD18	RAD18 E3	Protein Cc	44	GC03M00	3.01
537	PLEK	Pleckstrin	Protein Cc	43	GC02P068	3.01
538	TRIB1	Tribbles P	Protein Cc	41	GC08P125	3.01
539	RAD50	RAD50 Dc	Protein Cc	52	GC05P132	3
540	POLH	DNA Poly	Protein Cc	51	GC06P043	2.98
541	PAK2	P21 (RAC1	Protein Cc	50	GC03P196	2.97
542	CCL27	C-C Motif	Protein Cc	40	GC09M03	2.96
543	CGAS	Cyclic GM	Protein Cc	37	GC06M07	2.96
544	ENDOG	Endonucle	Protein Cc	45	GC09P128	2.96
545	DDR1	Discoidin I	Protein Cc	50	GC06P087	2.96
546	ANLN	Anillin, Ac	Protein Cc	45	GC07P036	2.95
547	CCT5	Chaperoni	Protein Cc	47	GC05P01C	2.94
548	DFFA	DNA Frag	Protein Cc	46	GC01M01	2.93
549	KPNB1	Karyopher	Protein Cc	46	GC17P047	2.93
550	RAD9A	RAD9 Che	Protein Cc	42	GC11P07C	2.92
551	MDC1	Mediator (	Protein Cc	43	GC06M06	2.9
552	GRK5	G Protein-	Protein Cc	46	GC10P119	2.89
553	MCTS1	MCTS1 Re	Protein Cc	39	GC0XP12C	2.89
554	PTMA	Prothymo	Protein Cc	44	GC02P231	2.87
555	MYH10	Myosin He	Protein Cc	48	GC17M00	2.86
556	SKP1	S-Phase K	Protein Cc	47	GC05M13	2.86
557	BTG1	BTG Anti-	Protein Cc	43	GC12M09	2.85
558	ID3	Inhibitor C	Protein Cc	42	GC01M02	2.84
559	TELO2	Telomere	Protein Cc	41	GC16P001	2.83
560	YWHAQ	Tyrosine 3	Protein Cc	49	GC02M00	2.83
561	MAT2A	Methionin	Protein Cc	50	GC02P085	2.82
562	RFC3	Replicatio	Protein Cc	43	GC13P033	2.81
563	HULC	Hepatocel	RNA Gene	23	GC06P008	2.81
564	CDC25C	Cell Divisi	Protein Cc	50	GC05M13	2.8
565	PGD	Phosphog	Protein Cc	51	GC01P01C	2.79
566	MPRIP	Myosin Ph	Protein Cc	40	GC17P017	2.77
567	GIT2	GIT ArfGA	Protein Cc	44	GC12M10	2.76
568	RPA1	Replicatio	Protein Cc	48	GC17P001	2.76
569	TPT1	Tumor Prc	Protein Cc	48	GC13M04	2.76
570	BACH1	BTB Doma	Protein Cc	44	GC21P029	2.75
571	SEC11A	SEC11 Ho	Protein Cc	41	GC15M08	2.74
572	SRPK1	SRSF Prot	Protein Cc	50	GC06M06	2.73
573	NCL	Nucleolin	Protein Cc	47	GC02M23	2.72
574	MIR449A	MicroRNA	RNA Gene	23	GC05M05	2.71
575	RBMS1	RNA Bindi	Protein Cc	42	GC02M16	2.7
576	KAT2B	Lysine Ace	Protein Cc	50	GC03P02C	2.7
577	ILF3	Interleukir	Protein Cc	40	GC19P01C	2.69
578	IRF7	Interferon	Protein Cc	50	GC11M00	2.69
579	SELENOH	Selenopro	Protein Cc	31	GC11P058	2.69

Using the keywords "Irradiation-induced intestinal injury" ,778 related genes were found in the GeneCards.Symbol=gene name ,Description=descriptive information about the gene , Category= categories of genesGIFtS=protein annotation scoring,GC id=number given by GeneCards,Score=gene Correspondence Scoring

Continued from above

580	DEFA4	Defensin /	Protein Cc	36	GC08M00	2.69
581	KIDINS220	Kinase D I	Protein Cc	44	GC02M00	2.68
582	DDX41	DEAD-Box	Protein Cc	47	GC05M17	2.68
583	RAD51D	RAD51 Pa	Protein Cc	41	GC17M03	2.67
584	UQCC2	Ubiquinol-	Protein Cc	39	GC06M03	2.67
585	PPFIBP1	PPFIA Binc	Protein Cc	43	GC12P027	2.64
586	BARD1	BRCA1 As	Protein Cc	50	GC02M21	2.62
587	NACA	Nascent P	Protein Cc	41	GC12M05	2.62
588	CARD16	Caspase R	Protein Cc	37	GC11M10	2.62
589	ERCC5	ERCC Exci	Protein Cc	47	GC13P102	2.61
590	DNAJC21	DnaJ Heat	Protein Cc	38	GC05P034	2.6
591	AFF4	ALF Trans	Protein Cc	44	GC05M13	2.59
592	CD180	CD180 Mc	Protein Cc	42	GC05M06	2.59
593	TNIP2	TNFAIP3 I	Protein Cc	41	GC04M00	2.59
594	PARP2	Poly(ADP-	Protein Cc	48	GC14P020	2.59
595	GRM5	Glutamate	Protein Cc	50	GC11M08	2.58
596	RPS3	Ribosoma	Protein Cc	46	GC11P078	2.56
597	SIAH1	Siah E3 Ub	Protein Cc	47	GC16M04	2.56
598	EIF2S3	Eukaryotic	Protein Cc	48	GC0XP024	2.55
599	CHD4	Chromodc	Protein Cc	47	GC12M00	2.54
600	CDC27	Cell Divisic	Protein Cc	44	GC17M04	2.54
601	ANAPC1	Anaphase	Protein Cc	44	GC02M11	2.53
602	CDK9	Cyclin Dep	Protein Cc	48	GC09P128	2.53
603	REV1	REV1 DNA	Protein Cc	43	GC02M09	2.52
604	CYFIP1	Cytoplasm	Protein Cc	42	GC15M02	2.52
605	GADD45G	GADD45G	Protein Cc	38	GC19M01	2.52
606	SMARCC1	SWI/SNF F	Protein Cc	45	GC03M04	2.52
607	SGK2	Serum/Glu	Protein Cc	45	GC20P043	2.51
608	TERF2	Telomeric	Protein Cc	44	GC16M06	2.51
609	CRADD	CASP2 An	Protein Cc	46	GC12P093	2.5
610	ABCF1	ATP Bindir	Protein Cc	43	GC06P030	2.5
611	UFM1	Ubiquitin I	Protein Cc	43	GC13P038	2.47
612	UBTF	Upstream	Protein Cc	45	GC17M04	2.45
613	UVRAG	UV Radiat	Protein Cc	43	GC11P075	2.43
614	CTDP1	CTD Phos	Protein Cc	44	GC18P079	2.42
616	DNASE1L3	Deoxyribo	Protein Cc	45	GC03M05	2.42
617	GSDMC	Gasdermir	Protein Cc	36	GC08M12	2.42
615	MIR26B	MicroRNA	RNA Gene	24	GC02P218	2.42
618	SAFB	Scaffold A	Protein Cc	42	GC19P005	2.41
619	ADGRL2	Adhesion	Protein Cc	43	GC01P081	2.41
620	SNW1	SNW Dom	Protein Cc	46	GC14M07	2.39
621	PRKACA	Protein Kir	Protein Cc	54	GC19M01	2.38
622	IP6K2	Inositol He	Protein Cc	41	GC03M04	2.38
623	CDC25B	Cell Divisic	Protein Cc	48	GC20P004	2.38
624	DMAP1	DNA Meth	Protein Cc	41	GC01P044	2.36
626	YWHAB	Tyrosine 3	Protein Cc	50	GC20P044	2.36
625	MIR494	MicroRNA	RNA Gene	18	GC14P110	2.36
627	SH3GLB1	SH3 Doma	Protein Cc	44	GC01P086	2.35
628	MIR216A	MicroRNA	RNA Gene	22	GC02M05	2.35
629	SYNCRIP	Synaptota	Protein Cc	42	GC06M08	2.34
630	TIMMDC1	Translocas	Protein Cc	40	GC03P119	2.34
631	EEF2	Eukaryotic	Protein Cc	51	GC19M00	2.34
632	KNSTRN	Kinetocho	Protein Cc	40	GC15P040	2.33
633	TRIM32	Tripartite I	Protein Cc	44	GC09P116	2.33
634	MCM7	Minichrom	Protein Cc	47	GC07M10	2.32
635	UBE2B	Ubiquitin (	Protein Cc	45	GC05P134	2.32
636	H2AC20	H2A Clust	Protein Cc	38	GC01P150	2.29
637	EIF3I	Eukaryotic	Protein Cc	41	GC01P032	2.29

Using the keywords "Irradiation-induced intestinal injury" ,778 related genes were found in the GeneCards.Symbol=gene name ,Description=descriptive information about the gene , Category= categories of genesGIFtS=protein annotation scoring,GC id=number given by GeneCards,Score=gene Correspondence Scoring

638	GNL3	G Protein	Protein Cc	45	GC03P052	2.29
639	HDGF	Heparin B	Protein Cc	43	GC01M15	2.28
640	PSME3	Proteason	Protein Cc	44	GC17P042	2.27
641	ALYREF	Aly/REF Ex	Protein Cc	41	GC17M08	2.26
642	RNF168	Ring Finge	Protein Cc	44	GC03M19	2.23
643	ASCC3	Activating	Protein Cc	41	GC06M10	2.23
644	KANK1	KN Motif /	Protein Cc	44	GC09P000	2.22
645	CDC20	Cell Divisi	Protein Cc	45	GC01P043	2.22
646	HYAL2	Hyaluroni	Protein Cc	46	GC03M05	2.22
647	NDUFB8	NADH:Ubi	Protein Cc	44	GC10M10	2.21
648	GYS1	Glycogen	Protein Cc	51	GC19M06	2.2
649	ATG3	Autophag	Protein Cc	43	GC03M11	2.2
650	MTHFD1L	Methylene	Protein Cc	44	GC06P150	2.19
651	ANP32A	Acidic Nu	Protein Cc	45	GC15M06	2.18
652	MAP4K4	Mitogen-/	Protein Cc	50	GC02P101	2.17
653	MARS1	Methionyl	Protein Cc	47	GC12P057	2.17
654	POLR3B	RNA Polyr	Protein Cc	46	GC12P106	2.17
655	PSMD2	Proteason	Protein Cc	45	GC03P184	2.17
656	USP5	Ubiquitin S	Protein Cc	47	GC12P022	2.16
657	DNAJB4	DnaJ Heat	Protein Cc	39	GC01P077	2.15
658	ANKFY1	Ankyrin Re	Protein Cc	40	GC17M00	2.14
659	FERMT2	FERM Dor	Protein Cc	41	GC14M05	2.14
660	RBX1	Ring-Box	Protein Cc	46	GC22P040	2.13
661	ATG4B	Autophag	Protein Cc	44	GC02P241	2.13
662	RNF8	Ring Finge	Protein Cc	44	GC06P087	2.12
663	ACY1	Aminoacy	Protein Cc	48	GC03P051	2.12
664	TMED10	Transmem	Protein Cc	44	GC14M07	2.12
665	PPP1CA	Protein Ph	Protein Cc	50	GC11M09	2.1
666	C1QBP	Complem	Protein Cc	47	GC17M00	2.09
667	DGKQ	Diacylglyc	Protein Cc	44	GC04M00	2.09
668	XAF1	XIAP Asso	Protein Cc	41	GC17P006	2.08
669	TERF2IP	TERF2 Inte	Protein Cc	44	GC16P075	2.07
670	WDR82	WD Repea	Protein Cc	39	GC03M05	2.07
671	SEC62	SEC62 Ho	Protein Cc	38	GC03P169	2.07
672	SUMO1	Small Ubi	Protein Cc	48	GC02M20	2.07
673	HRNR	Hornerin	Protein Cc	38	GC01M15	2.06
674	CCNA1	Cyclin A1	Protein Cc	44	GC13P036	2.06
675	TUFM	Tu Transla	Protein Cc	48	GC16M03	2.05
676	AP3B1	Adaptor R	Protein Cc	47	GC05M07	2.05
677	HNRNPD	Heteroger	Protein Cc	45	GC04M08	2.05
678	COPS3	COP9 Sigr	Protein Cc	42	GC17M01	2.04
679	KCTD5	Potassium	Protein Cc	37	GC16P002	2.03
680	STT3A	STT3 Olig	Protein Cc	48	GC11P125	2.03
681	MTHFS	Methenylt	Protein Cc	45	GC15M07	2.03
682	FZR1	Fizzy And	Protein Cc	44	GC19P003	2.03
683	PNN	Pinin, Des	Protein Cc	37	GC14P039	2.02
684	MAP4	Microtubu	Protein Cc	43	GC03M04	2.01
685	GRK6	G Protein-	Protein Cc	48	GC05P177	2
686	CLIC1	Chloride I	Protein Cc	45	GC06M06	2
687	ALDH1A3	Aldehyde	Protein Cc	47	GC15P100	1.95
688	DNAJB11	DnaJ Heat	Protein Cc	44	GC03P186	1.94
689	ICMT	Isoprenylc	Protein Cc	44	GC01M00	1.91
690	GOLGB1	Golgin B1	Protein Cc	38	GC03M12	1.9
691	DNAJA1	DnaJ Heat	Protein Cc	44	GC09P033	1.9
692	EIF4A3	Eukaryotic	Protein Cc	45	GC17M08	1.89
693	ATG12	Autophag	Protein Cc	41	GC05M11	1.89
694	MAST1	Microtubu	Protein Cc	44	GC19P014	1.88
695	BIK	BCL2 Inter	Protein Cc	43	GC22P043	1.87

Using the keywords "Irradiation-induced intestinal injury",778 related genes were found in the GeneCards.Symbol=gene name ,Description=descriptive information about the gene , Category= categories of genesGIFtS=protein annotation scoring,GC id=number given by GeneCards,Score=gene Correspondence Scoring

Continued from above

696	EIF3B	Eukaryotic	Protein Cc	41	GC07P002	1.87
697	DUSP2	Dual Spec	Protein Cc	43	GC02M09	1.86
698	RNF4	Ring Finge	Protein Cc	42	GC04P002	1.86
699	HAX1	HCLS1 Ass	Protein Cc	45	GC01P154	1.85
700	ZNF331	Zinc Finge	Protein Cc	40	GC19P07C	1.85
701	MYBBP1A	MYB Bindi	Protein Cc	41	GC17M00	1.85
702	HMMR	Hyalurona	Protein Cc	45	GC05P163	1.84
703	RAD23B	RAD23 Hc	Protein Cc	47	GC09P107	1.84
704	RPL3	Ribosoma	Protein Cc	44	GC22M05	1.84
705	TIE1	Tyrosine K	Protein Cc	47	GC01P043	1.84
706	PPP4C	Protein Ph	Protein Cc	47	GC16P041	1.83
707	CUL4A	Cullin 4A	Protein Cc	45	GC13P113	1.83
708	UBE2D2	Ubiquitin (	Protein Cc	45	GC05P139	1.81
709	H1-5	H1.5 Linke	Protein Cc	41	GC06M06	1.81
710	NSDHL	NAD(P) De	Protein Cc	46	GC0XP152	1.81
711	DOCK4	Dedicator	Protein Cc	42	GC07M11	1.8
712	PNPLA6	Patatin Lik	Protein Cc	45	GC19P007	1.79
713	ARF1	ADP Ribos	Protein Cc	47	GC01P228	1.79
714	NOLC1	Nucleolar	Protein Cc	43	GC10P102	1.79
715	GEMIN4	Gem Nucl	Protein Cc	41	GC17M00	1.79
716	CERT1	Ceramide	Protein Cc	42	GC05M07	1.78
717	RSL1D1	Ribosoma	Protein Cc	41	GC16M01	1.78
718	NUP93	Nucleopoi	Protein Cc	44	GC16P057	1.78
719	SLC45A2	Solute Car	Protein Cc	43	GC05M03	1.76
720	UIMC1	Ubiquitin I	Protein Cc	42	GC05M17	1.76
721	ZBTB7B	Zinc Finge	Protein Cc	41	GC01P155	1.75
722	DLST	Dihydrolip	Protein Cc	48	GC14P074	1.74
723	PCLAF	PCNA Clai	Protein Cc	37	GC15M11	1.74
724	MCPH1	Microceph	Protein Cc	42	GC08P006	1.73
725	SMARCA1	SWI/SNF F	Protein Cc	42	GC0XM12	1.72
726	CKAP5	Cytoskelet	Protein Cc	43	GC11M09	1.72
727	UBE2D3	Ubiquitin (	Protein Cc	48	GC04M10	1.71
728	DCX	Doublecor	Protein Cc	47	GC0XM11	1.7
729	RAD23A	RAD23 Hc	Protein Cc	45	GC19P014	1.69
730	RPL4	Ribosoma	Protein Cc	44	GC15M06	1.68
731	UFC1	Ubiquitin-	Protein Cc	40	GC01P161	1.68
732	UBE2N	Ubiquitin (	Protein Cc	50	GC12M09	1.67
733	PRPSAP2	Phosphori	Protein Cc	37	GC17P057	1.66
734	CDC14A	Cell Divisic	Protein Cc	47	GC01P10C	1.66
735	UBE2G2	Ubiquitin (	Protein Cc	45	GC21M04	1.65
736	MACROH2	MacroH2A	Protein Cc	43	GC05M13	1.65
737	CBX1	Chromobc	Protein Cc	44	GC17M04	1.64
738	H2AZ2	H2A.Z Var	Protein Cc	38	GC07M04	1.62
739	RECQL	RecQ Like	Protein Cc	44	GC12M02	1.6
740	TOPBP1	DNA Topc	Protein Cc	44	GC03M13	1.59
741	MAPK8IP3	Mitogen-A	Protein Cc	43	GC16P001	1.59
742	NDUFA9	NADH:Ubi	Protein Cc	46	GC12P004	1.59
743	CDK7	Cyclin Dep	Protein Cc	49	GC05P069	1.58
744	OTUB1	OTU Deuk	Protein Cc	44	GC11P063	1.58
745	ACVR1C	Activin A F	Protein Cc	45	GC02M15	1.55
746	RPA2	Replicatio	Protein Cc	47	GC01M02	1.54
747	MRTO4	MRT4 Hor	Protein Cc	37	GC01P019	1.54
748	UBQLN1	Ubiquilin 1	Protein Cc	44	GC09M08	1.52
749	RPS3A	Ribosoma	Protein Cc	43	GC04P151	1.46
750	GLG1	Golgi Glyc	Protein Cc	41	GC16M07	1.44
751	POLR2L	RNA Polyr	Protein Cc	43	GC11M00	1.43
752	PSMC1	Proteasom	Protein Cc	43	GC14P09C	1.42
753	NIBAN1	Niban Apc	Protein Cc	39	GC01M18	1.42

Using the keywords "Irradiation-induced intestinal injury" ,778 related genes were found in the GeneCards.Symbol=gene name ,Description=descriptive information about the gene , Category= categories of genesGIFtS=protein annotation scoring,GC id=number given by GeneCards,Score=gene Correspondence Scoring

754	NDUFB6	NADH:Ubi	Protein Cc	42	GC09M03	1.42
755	ATG4C	Autophag	Protein Cc	42	GC01P062	1.41
756	PPP2R2A	Protein Ph	Protein Cc	45	GC08P026	1.39
757	PRPS2	Phosphori	Protein Cc	47	GC0XP012	1.39
758	UBQLN2	Ubiquilin 2	Protein Cc	45	GC0XP056	1.38
759	PDCD6	Programm	Protein Cc	41	GC05P00C	1.36
760	DNAJB1P1	DnaJ Heat	Pseudoge	10	GC02M19	1.36
761	MAGED2	MAGE Far	Protein Cc	43	GC0XP054	1.32
762	ANP32B	Acidic Nuc	Protein Cc	40	GC09P097	1.3
763	CDT1	Chromatir	Protein Cc	46	GC16P088	1.3
764	ZC3H14	Zinc Finge	Protein Cc	43	GC14P088	1.3
765	PIP	Prolactin I	Protein Cc	41	GC07P143	1.28
766	SMG1	SMG1 Noi	Protein Cc	43	GC16M01	1.27
767	TCL1A	TCL1 Fam	Protein Cc	45	GC14M09	1.25
768	USP14	Ubiquitin S	Protein Cc	47	GC18P00C	1.23
769	RBBP7	RB Binding	Protein Cc	45	GC0XM01	1.22
770	RFC5	Replicatioi	Protein Cc	43	GC12P118	1.21
771	H1-1	H1.1 Linke	Protein Cc	37	GC06M02	1.2
772	UBE2E1	Ubiquitin (	Protein Cc	42	GC03P023	1.18
773	NPAT	Nuclear Pi	Protein Cc	37	GC11M10	1.16
774	USP11	Ubiquitin S	Protein Cc	44	GC0XP047	1.15
775	UBE2O	Ubiquitin (	Protein Cc	43	GC17M07	1.14
776	MIR219A2	MicroRNA	RNA Gene	18	GC09M12	1.13
777	H2BC11	H2B Clust	Protein Cc	37	GC06M06	1.09
778	USP15	Ubiquitin S	Protein Cc	50	GC12P062	1.03

Using the keywords “Irradiation-induced intestinal injury”,778 related genes were found in the GeneCards.Symbol=gene name ,Description=descriptive information about the gene , Category= categories of genesGIFtS=protein annotation scoring,GC id=number given by GeneCards,Score=gene Correspondence Scoring
