# Supplementary material for: Astragalus polysaccharide promotes the regeneration of intestinal stem cells through HIF‐1 signalling pathway
Source: J Cell Mol Med. 2023 Dec 14;28(3):e18058. doi: 10.1111/jcmm.18058 (PMC10844761; doi:10.1111/jcmm.18058)
Supplement: Supplementary file 3 — Table S3. [file JCMM-28-e18058-s001.pdf]

The list of KEGG pathway enrichment terms.

Continued from above

Category	Term	%	Count	FDR	PValue	Genes	List Total	Pop Hits	Pop Total	Fold Enrichment	Bonferroni	Benjamini
KEGG_PATHWAY	mmu05417:Lipid and atherosclerosis	36.36364	28	5.35E-24	6.37E-26	TNF, RELA, CASP9, MAPK8, CASP6, CASP3, AKT1, MAPK1, PLCG1, MAPK3, JUN, IFNB1, EIF2AK3, TNFRSF10B, MAPK14, SOD2, MMP9, NFKB1, ERN1, IL6, PIK3CA, IL1B, BCL2, CYCS, BAX, PPARG, TLR4, NFE2L2	73	216	8998	15.978184	1.43E-23	1.43E-23
KEGG_PATHWAY	mmu05200:Pathways in cancer	46.75325	36	1.52E-22	3.63E-24	XIAP, PTGS2, HIF1A, FGF2, RELA, CASP9, MAPK8, CASP3, AKT1, HMOX1, MAPK1, PLCG1, MAPK3, NQO1, JUN, TGFB1, NOS2, EGF, CDKN2A, HGF, MMP2, IGF1, MMP9, MTOR, NFKB1, VEGFA, IL6, RAD51, PIK3CA, SP1, BCL2, CYCS, BAX, PPARG, PPARD, NFE2L2	73	543	8998	8.1719519	8.17E-22	4.08E-22
KEGG_PATHWAY	mmu04933:AGE-RAGE signaling pathway in diabetic complications	24.67532	19	1.14E-18	4.07E-20	JUN, TGFB1, MMP2, MAPK14, TNF, NFKB1, RELA, VEGFA, IL6, MAPK8, PIK3CA, CASP3, IL1B, BCL2, AKT1, MAPK1, BAX, PLCG1, MAPK3	73	101	8998	23.187576	9.16E-18	3.05E-18
KEGG_PATHWAY	mmu05161:Hepatitis B	27.27273	21	1.24E-17	5.93E-19	JUN, PCNA, TGFB1, IFNB1, MAPK14, TNF, MMP9, NFKB1, RELA, CASP9, IL6, MAPK8, PIK3CA, CASP3, BCL2, AKT1, CYCS, MAPK1, BAX, TLR4, MAPK3	73	163	8998	15.880158	1.33E-16	3.33E-17
KEGG_PATHWAY	mmu05142:Chagas disease	23.37662	18	3.35E-17	1.99E-18	IL10, JUN, TGFB1, NOS2, IFNB1, MAPK14, TNF, NFKB1, RELA, IL6, MAPK8, PIK3CA, IL1B, TLR9, AKT1, MAPK1,	73	103	8998	21.54063	4.48E-16	8.96E-17
KEGG_PATHWAY	mmu05152:Tuberculosis	27.27273	21	6.15E-17	4.39E-18	IL10, TGFB1, NOS2, IFNB1, MAPK14, TNF, NFKB1, RELA, CASP9, IL6, MAPK8, CASP3, IL1B, TLR9, BCL2, AKT1, CYCS, MAPK1, BAX, TLR4, MAPK3	73	180	8998	14.380365	9.88E-16	1.65E-16

The 463 potential targets of APS were intersected with 778 genes associated with irradition induced intestinal injury to obtain 82 genes, which were then analyzed for KEGG pathway enrichment.P-values are used to measure the degree of differential expression or enrichment of genes or metabolic pathways under different conditions; the smaller the P-value, the more significant the difference or enrichment.

Continued from above

KEGG_PATHWAY	mmu04932:Non-alcoholic fatty liver disease	25.97403	20	7.05E-17	5.87E-18	SREBF1, NDUFB8, NDUFA13, JUN, TGFB1, EIF2AK3, MAPK14, TNF, NFKB1, RELA, ERN1, IL6, MAPK8, PIK3CA, CASP3, IL1B, AKT1, CYCS, BAX, PPARG	73	156	8998	15.802599	1.32E-15	1.89E-16
KEGG_PATHWAY	mmu04210:Apoptosis	24.67532	19	1.13E-16	1.08E-17	JUN, EIF2AK3, TNFRSF10B, XIAP, TNF, NFKB1, RELA, CASP9, ERN1, MAPK8, PIK3CA, CASP6, CASP3, BCL2, AKT1, CYCS, MAPK1, BAX, MAPK3	73	136	8998	17.220185	2.42E-15	3.03E-16
KEGG_PATHWAY	mmu05167:Kaposi sarcoma-associated herpesvirus infection	28.57143	22	1.81E-16	1.94E-17	BECN1, JUN, IFNB1, MAPK14, PTGS2, HIF1A, FGF2, MTOR, NFKB1, RELA, VEGFA, CASP9, IL6, MAPK8, PIK3CA, CASP3, AKT1, CYCS, MAPK1, BAX, PLCG1, MAPK3	73	224	8998	12.10592	4.37E-15	4.85E-16
KEGG_PATHWAY	mmu05145:Toxoplasmosis	22.07792	17	1.54E-15	1.83E-16	IL10, TGFB1, NOS2, XIAP, MAPK14, TNF, NFKB1, RELA, CASP9, MAPK8, CASP3, BCL2, AKT1, CYCS, MAPK1, TLR4, MAPK3	73	110	8998	19.049315	5.00E-14	4.11E-15
KEGG_PATHWAY	mmu04668:TNF signaling pathway	22.07792	17	2.17E-15	2.85E-16	JUN, IFNB1, MAPK14, PTGS2, TNF, MMP9, NFKB1, RELA, IL6, MAPK8, PIK3CA, CASP3, IL1B, AKT1, MAPK1, DNM1L, MAPK3	73	113	8998	18.543581	7.49E-14	5.82E-15
KEGG_PATHWAY	mmu04066:HIF-1 signaling pathway	22.07792	17	2.30E-15	3.29E-16	NOS2, EGF, IGF1, HIF1A, MTOR, NFKB1, RELA, VEGFA, IL6, PIK3CA, BCL2, AKT1, HMOX1, MAPK1, PLCG1, TLR4,	73	114	8998	18.380918	7.49E-14	6.17E-15
KEGG_PATHWAY	mmu05212:Pancreatic cancer	19.48052	15	3.42E-15	5.30E-16	TGFB1, CDKN2A, EGF, MTOR, NFKB1, RELA, VEGFA, CASP9, MAPK8, RAD51, PIK3CA, AKT1, MAPK1, BAX, MAPK3	73	76	8998	24.327686	1.25E-13	9.17E-15

The 463 potential targets of APS were intersected with 778 genes associated with irradition induced intestinal injury to obtain 82 genes, which were then analyzed for KEGG pathway enrichment.P-values are used to measure the degree of differential expression or enrichment of genes or metabolic pathways under different conditions; the smaller the P-value, the more significant the difference or enrichment.

Continued from above

KEGG_PATHWAY	mmu05208:Chemical carcinogenesis - reactive oxygen species	25.97403	20	2.94E-14	4.89E-15	NQO1, NDUFB8, NDUFA13, JUN, EGF, HGF, MAPK14, SOD2, HIF1A, NFKB1, RELA, VEGFA, MAPK8, PIK3CA, CAT, AKT1, HMOX1, MAPK1, NFE2L2, MAPK3	73	222	8998	11.104529	1.10E-12	7.87E-14
KEGG_PATHWAY	mmu05022:Pathways of neurodegeneration - multiple diseases	33.76623	26	4.36E-14	7.78E-15	BECN1, NDUFB8, NDUFA13, PTGS2, TNF, RELA, CASP9, MAPK8, CASP3, MAPK1, NOS1, PLCG1, MAPK3, NOS2, BDNF, EIF2AK3, MAPK14, MTOR, NFKB1, ERN1, IL6, IL1B, CAT, BCL2, CYCS, BAX	73	471	8998	6.8041765	1.75E-12	1.17E-13
KEGG_PATHWAY	mmu01522:Endocrine resistance	19.48052	15	5.55E-14	1.06E-14	JUN, CDKN2A, MMP2, IGF1, MAPK14, MMP9, MTOR, MAPK8, PIK3CA, SP1, BCL2, AKT1, MAPK1, BAX, MAPK3	73	93	8998	19.880689	2.37E-12	1.49E-13
KEGG_PATHWAY	mmu05205:Proteoglycans in cancer	24.67532	19	8.86E-14	1.82E-14	TGFB1, HGF, MMP2, IGF1, MAPK14, TNF, HIF1A, MMP9, FGF2, MTOR, DCN, VEGFA, PIK3CA, CASP3, AKT1, MAPK1, PLCG1, TLR4, MAPK3	73	205	8998	11.424123	4.10E-12	2.37E-13
KEGG_PATHWAY	mmu05162:Measles	22.07792	17	8.86E-14	1.90E-14	JUN, IFNB1, EIF2AK3, NFKB1, RELA, CASP9, IL6, MAPK8, PIK3CA, CASP3, IL1B, TLR9, BCL2, AKT1, CYCS, BAX, TLR4	73	146	8998	14.352224	4.27E-12	2.37E-13
KEGG_PATHWAY	mmu05133:Pertussis	18.18182	14	9.64E-14	2.18E-14	IL10, JUN, NOS2, MAPK14, TNF, NFKB1, RELA, IL6, MAPK8, CASP3, IL1B, MAPK1, TLR4, MAPK3	73	77	8998	22.410959	4.90E-12	2.58E-13
KEGG_PATHWAY	mmu04620:Toll-like receptor signaling pathway	19.48052	15	1.24E-13	3.02E-14	JUN, IFNB1, MAPK14, TNF, NFKB1, RELA, IL6, MAPK8, PIK3CA, IL1B, TLR9, AKT1, MAPK1, TLR4, MAPK3	73	100	8998	18.489041	6.79E-12	3.32E-13
KEGG_PATHWAY	mmu01521:EGFR tyrosine kinase inhibitor resistance	18.18182	14	1.24E-13	3.10E-14	EGF, HGF, IGF1, FGF2, MTOR, VEGFA, IL6, PIK3CA, BCL2, AKT1, MAPK1, BAX, PLCG1, MAPK3	73	79	8998	21.843593	6.97E-12	3.32E-13

The 463 potential targets of APS were intersected with 778 genes associated with irradition induced intestinal injury to obtain 82 genes, which were then analyzed for KEGG pathway enrichment.P-values are used to measure the degree of differential expression or enrichment of genes or metabolic pathways under different conditions; the smaller the P-value, the more significant the difference or enrichment.

Continued from above

KEGG_PATHWAY	mmu05163:Human cytomegalovirus infection	25.97403	20	2.55E-13	6.67E-14	CDKN2A, IFNB1, MAPK14, PTGS2, TNF, MTOR, NFKB1, RELA, VEGFA, CASP9, IL6, PIK3CA, SP1, CASP3, IL1B, AKT1, CYCS, MAPK1, BAX,	73	256	8998	9.6297089	1.50E-11	6.83E-13
KEGG_PATHWAY	mmu05135:Yersinia infection	20.77922	16	4.08E-13	1.12E-13	IL10, JUN, IFNB1, MAPK14, TNF, NFKB1, RELA, IL6, MAPK8, PIK3CA, IL1B, AKT1, MAPK1, PLCG1, TLR4, MAPK3	73	135	8998	14.608625	2.51E-11	1.09E-12
KEGG_PATHWAY	mmu05235:PD-L1 expression and PD-1 checkpoint pathway in cancer	18.18182	14	4.48E-13	1.33E-13	JUN, EGF, MAPK14, HIF1A, MTOR, NFKB1, RELA, PIK3CA, TLR9, AKT1, MAPK1, PLCG1, TLR4, MAPK3	73	88	8998	19.609589	3.00E-11	1.20E-12
KEGG_PATHWAY	mmu05210:Colorectal cancer	18.18182	14	4.48E-13	1.33E-13	JUN, TGFB1, EGF, MTOR, CASP9, MAPK8, PIK3CA, CASP3, BCL2, AKT1, CYCS, MAPK1, BAX, MAPK3	73	88	8998	19.609589	3.00E-11	1.20E-12
KEGG_PATHWAY	mmu05140:Leishmaniasis	16.88312	13	6.32E-13	1.95E-13	IL10, JUN, TGFB1, NOS2, MAPK14, PTGS2, TNF, NFKB1, RELA, IL1B, MAPK1, TLR4, MAPK3	73	70	8998	22.891194	4.40E-11	1.69E-12
KEGG_PATHWAY	mmu05170:Human immunodeficiency virus 1 infection	24.67532	19	8.85E-13	2.84E-13	JUN, IFNB1, MAPK14, TNF, MTOR, NFKB1, RELA, CASP9, MAPK8, PIK3CA, CASP3, BCL2, AKT1, CYCS, MAPK1, BAX, PLCG1, TLR4, MAPK3	73	240	8998	9.758105	6.40E-11	2.37E-12
KEGG_PATHWAY	mmu05418:Fluid shear stress and atherosclerosis	20.77922	16	1.20E-12	3.99E-13	NQO1, JUN, MMP2, MAPK14, TNF, MMP9, NFKB1, RELA, VEGFA, MAPK8, PIK3CA, IL1B, BCL2, AKT1, HMOX1, NFE2L2	73	147	8998	13.416084	8.97E-11	3.20E-12
KEGG_PATHWAY	mmu05132:Salmonella infection	24.67532	19	2.04E-12	7.05E-13	JUN, TNFRSF10B, MAPK14, TNF, NFKB1, RELA, IL6, MAPK8, PIK3CA, CASP3, IL1B, TLR9, BCL2, AKT1, CYCS, MAPK1, BAX, TLR4, MAPK3	73	253	8998	9.2567004	1.59E-10	5.47E-12

The 463 potential targets of APS were intersected with 778 genes associated with irradition induced intestinal injury to obtain 82 genes, which were then analyzed for KEGG pathway enrichment.P-values are used to measure the degree of differential expression or enrichment of genes or metabolic pathways under different conditions; the smaller the P-value, the more significant the difference or enrichment.

Continued from above

KEGG_PATHWAY	mmu05010:Alzheimer disease	28.57143	22	2.77E-12	9.91E-13	BECN1, NDUFB8, NDUFA13, NOS2, EIF2AK3, PTGS2, TNF, MTOR, NFKB1, RELA, CASP9, ERN1, IL6, MAPK8, PIK3CA, CASP3, IL1B, AKT1, CYCS, MAPK1, NOS1, MAPK3	73	383	8998	7.0802246	2.23E-10	7.43E-12
KEGG_PATHWAY	mmu04926:Relaxin signaling pathway	19.48052	15	3.03E-12	1.12E-12	JUN, TGFB1, NOS2, MMP2, MAPK14, MMP9, NFKB1, RELA, VEGFA, MAPK8, PIK3CA, AKT1, MAPK1, NOS1, MAPK3	73	129	8998	14.33259	2.52E-10	8.13E-12
KEGG_PATHWAY	mmu04625:C-type lectin receptor signaling pathway	18.18182	14	8.54E-12	3.25E-12	IL10, JUN, MAPK14, PTGS2, TNF, NFKB1, RELA, IL6, MAPK8, PIK3CA, IL1B, AKT1, MAPK1, MAPK3	73	112	8998	15.407534	7.32E-10	2.29E-11
KEGG_PATHWAY	mmu05164:Influenza A	20.77922	16	1.12E-11	4.40E-12	IFNB1, TNFRSF10B, TNF, NFKB1, RELA, CASP9, IL6, PIK3CA, CASP3, IL1B, AKT1, CYCS, MAPK1, BAX, TLR4,	73	173	8998	11.399794	9.89E-10	3.00E-11
KEGG_PATHWAY	mmu04657:IL-17 signaling pathway	16.88312	13	1.64E-11	6.64E-12	JUN, MAPK14, PTGS2, TNF, MMP9, NFKB1, RELA, IL6, MAPK8, CASP3, IL1B, MAPK1, MAPK3	73	93	8998	17.229931	1.49E-09	4.39E-11
KEGG_PATHWAY	mmu04010:MAPK signaling pathway	24.67532	19	2.17E-11	9.04E-12	JUN, TGFB1, BDNF, EGF, HGF, IGF1, MAPK14, TNF, FGF2, NFKB1, RELA, VEGFA, CDC25B, MAPK8, CASP3, IL1B, AKT1, MAPK1, MAPK3	73	294	8998	7.9658	2.04E-09	5.81E-11
KEGG_PATHWAY	mmu04380:Osteoclast differentiation	18.18182	14	4.28E-11	1.84E-11	JUN, TGFB1, IFNB1, MAPK14, TNF, NFKB1, RELA, MAPK8, PIK3CA, IL1B, AKT1, MAPK1, PPARG, MAPK3	73	128	8998	13.481592	4.13E-09	1.15E-10
KEGG_PATHWAY	mmu04068:FoxO signaling pathway	18.18182	14	5.61E-11	2.47E-11	IL10, TGFB1, EGF, IGF1, MAPK14, SOD2, SIRT1, IL6, MAPK8, PIK3CA, CAT, AKT1, MAPK1, MAPK3	73	131	8998	13.172854	5.56E-09	1.50E-10

The 463 potential targets of APS were intersected with 778 genes associated with irradition induced intestinal injury to obtain 82 genes, which were then analyzed for KEGG pathway enrichment.P-values are used to measure the degree of differential expression or enrichment of genes or metabolic pathways under different conditions; the smaller the P-value, the more significant the difference or enrichment.

Continued from above

KEGG_PATHWAY	mmu04659:Th17 cell differentiation	16.88312	13	6.38E-11	2.89E-11	JUN, TGFB1, MAPK14, HIF1A, MTOR, NFKB1, RELA, IL6, MAPK8, IL1B, MAPK1, PLCG1, MAPK3	73	105	8998	15.260796	6.49E-09	1.71E-10
KEGG_PATHWAY	mmu04621:NOD-like receptor signaling pathway	20.77922	16	1.90E-10	8.81E-11	JUN, IFNB1, XIAP, MAPK14, TNF, NFKB1, RELA, IL6, MAPK8, IL1B, BCL2, MAPK1, DNM1L, TLR4, ATG5, MAPK3	73	213	8998	9.2589877	1.98E-08	5.08E-10
KEGG_PATHWAY	mmu04211:Longevity regulating pathway	15.58442	12	2.07E-10	9.84E-11	PIK3CA, CAT, AKT1, BAX, PPARG, IGF1, SOD2, SIRT1, NFKB1, RELA, MTOR, ATG5	73	90	8998	16.434703	2.21E-08	5.54E-10
KEGG_PATHWAY	mmu05222:Small cell lung cancer	15.58442	12	2.90E-10	1.42E-10	CASP9, PIK3CA, NOS2, CASP3, BCL2, AKT1, XIAP, CYCS, BAX, PTGS2, NFKB1, RELA	73	93	8998	15.904551	3.18E-08	7.77E-10
KEGG_PATHWAY	mmu04722:Neurotrophin signaling pathway	16.88312	13	3.13E-10	1.57E-10	JUN, BDNF, MAPK14, NFKB1, RELA, MAPK8, PIK3CA, BCL2, AKT1, MAPK1, BAX, PLCG1, MAPK3	73	121	8998	13.242839	3.52E-08	8.39E-10
KEGG_PATHWAY	mmu04151:PI3K-Akt signaling pathway	24.67532	19	4.85E-10	2.48E-10	BDNF, IFNB1, EGF, HGF, IGF1, PRL, FGF2, MTOR, NFKB1, RELA, VEGFA, CASP9, IL6, PIK3CA, BCL2, AKT1, MAPK1, TLR4, MAPK3	73	359	8998	6.5235242	5.58E-08	1.30E-09
KEGG_PATHWAY	mmu05215:Prostate cancer	15.58442	12	5.38E-10	2.82E-10	CASP9, PIK3CA, EGF, BCL2, AKT1, MAPK1, IGF1, MMP9, NFKB1, RELA, MTOR, MAPK3	73	99	8998	14.940639	6.34E-08	1.44E-09
KEGG_PATHWAY	mmu04660:T cell receptor signaling pathway	15.58442	12	8.12E-10	4.35E-10	IL10, MAPK8, JUN, PIK3CA, AKT1, MAPK1, PLCG1, MAPK14, TNF, NFKB1, RELA,	73	103	8998	14.36042	9.78E-08	2.17E-09
KEGG_PATHWAY	mmu05160:Hepatitis C	18.18182	14	8.37E-10	4.58E-10	IFNB1, EGF, EIF2AK3, TNF, NFKB1, RELA, CASP9, PIK3CA, CASP3, AKT1, CYCS, MAPK1, BAX, MAPK3	73	165	8998	10.458447	1.03E-07	2.24E-09
KEGG_PATHWAY	mmu01524:Platinum drug resistance	14.28571	11	1.06E-09	5.95E-10	CASP9, PIK3CA, CDKN2A, CASP3, BCL2, AKT1, XIAP, CYCS, BAX, MAPK1, MAPK3	73	80	8998	16.948288	1.34E-07	2.85E-09

The 463 potential targets of APS were intersected with 778 genes associated with irradition induced intestinal injury to obtain 82 genes, which were then analyzed for KEGG pathway enrichment.P-values are used to measure the degree of differential expression or enrichment of genes or metabolic pathways under different conditions; the smaller the P-value, the more significant the difference or enrichment.

Continued from above

KEGG_PATHWAY	mmu04936:Alcoholic liver disease	16.88312	13	1.65E-09	9.41E-10	SREBF1, IFNB1, MAPK14, TNF, SIRT1, NFKB1, RELA, IL6, MAPK8, CASP3, IL1B, AKT1, TLR4	73	141	8998	11.364422	2.12E-07	4.41E-09
KEGG_PATHWAY	mmu05169:Epstein-Barr virus infection	19.48052	15	4.98E-09	2.91E-09	JUN, IFNB1, MAPK14, TNF, NFKB1, RELA, CASP9, IL6, MAPK8, PIK3CA, CASP3, BCL2, AKT1, CYCS, BAX	73	231	8998	8.0039139	6.54E-07	1.34E-08
KEGG_PATHWAY	mmu04215:Apoptosis - multiple species	10.38961	8	7.26E-09	4.48E-09	CASP9, BECN1, MAPK8, CASP3, BCL2, XIAP, CYCS, BAX	73	32	8998	30.815068	1.01E-06	1.94E-08
KEGG_PATHWAY	mmu05231:Choline metabolism in cancer	14.28571	11	7.26E-09	4.52E-09	MAPK8, JUN, PIK3CA, SP1, EGF, AKT1, MAPK1, PLCG1, HIF1A, MTOR, MAPK3	73	98	8998	13.835337	1.02E-06	1.94E-08
KEGG_PATHWAY	mmu05223:Non-small cell lung cancer	12.98701	10	7.26E-09	4.58E-09	CASP9, PIK3CA, CDKN2A, EGF, HGF, AKT1, BAX, MAPK1, PLCG1, MAPK3	73	72	8998	17.119483	1.03E-06	1.94E-08
KEGG_PATHWAY	mmu05218:Melanoma	12.98701	10	7.26E-09	4.58E-09	PIK3CA, CDKN2A, EGF, HGF, AKT1, BAX, MAPK1, IGF1, FGF2, MAPK3	73	72	8998	17.119483	1.03E-06	1.94E-08
KEGG_PATHWAY	mmu05214:Glioma	12.98701	10	9.13E-09	5.87E-09	PIK3CA, CDKN2A, EGF, AKT1, BAX, MAPK1, IGF1, PLCG1, MTOR, MAPK3	73	74	8998	16.656794	1.32E-06	2.45E-08
KEGG_PATHWAY	mmu05166:Human T-cell leukemia virus 1 infection	19.48052	15	1.23E-08	8.07E-09	JUN, TGFB1, CDKN2A, XIAP, TNF, NFKB1, RELA, IL6, MAPK8, PIK3CA, AKT1, MAPK1, BAX, RAN, MAPK3	73	250	8998	7.3956164	1.82E-06	3.30E-08
KEGG_PATHWAY	mmu05415:Diabetic cardiomyopathy	18.18182	14	1.41E-08	9.39E-09	NDUFB8, NDUFA13, TGFB1, MMP2, GSR, MAPK14, MMP9, MTOR, NFKB1, RELA, MAPK8, PIK3CA, SP1, AKT1	73	211	8998	8.1784068	2.11E-06	3.77E-08
KEGG_PATHWAY	mmu05225:Hepatocellular carcinoma	16.88312	13	1.55E-08	1.05E-08	NQO1, TGFB1, CDKN2A, HGF, MTOR, PIK3CA, AKT1, HMOX1, MAPK1, BAX, PLCG1, NFE2L2, MAPK3	73	174	8998	9.2091009	2.36E-06	4.14E-08
KEGG_PATHWAY	mmu05146:Amoebiasis	14.28571	11	1.55E-08	1.07E-08	IL10, IL6, TGFB1, PIK3CA, NOS2, IL1B, CASP3, TNF, TLR4, NFKB1, RELA	73	107	8998	12.671617	2.41E-06	4.16E-08

The 463 potential targets of APS were intersected with 778 genes associated with irradition induced intestinal injury to obtain 82 genes, which were then analyzed for KEGG pathway enrichment.P-values are used to measure the degree of differential expression or enrichment of genes or metabolic pathways under different conditions; the smaller the P-value, the more significant the difference or enrichment.

Continued from above

KEGG_PATHWAY	mmu05206:MicroRNAs in cancer	20.77922	16	1.67E-08	1.17E-08	CDKN2A, MIR34A, PTGS2, MMP9, SIRT1, MTOR, NFKB1, VEGFA, CDC25B, PIK3CA, CASP3, BCL2, HMOX1, MAPK1, PLCG1, MAPK3	73	303	8998	6.5087933	2.64E-06	4.47E-08
KEGG_PATHWAY	mmu04140:Autophagy - animal	15.58442	12	1.90E-08	1.36E-08	ERN1, BECN1, MAPK8, PIK3CA, EIF2AK3, BCL2, AKT1, MAPK1, HIF1A, MTOR, ATG5, MAPK3	73	142	8998	10.416361	3.06E-06	5.10E-08
KEGG_PATHWAY	mmu04370:VEGF signaling pathway	11.68831	9	2.16E-08	1.57E-08	CASP9, PIK3CA, AKT1, MAPK1, PLCG1, MAPK14, PTGS2, MAPK3, VEGFA	73	58	8998	19.126594	3.54E-06	5.80E-08
KEGG_PATHWAY	mmu05020:Prion disease	19.48052	15	2.66E-08	1.96E-08	NDUFB8, NDUFA13, EIF2AK3, MAPK14, TNF, CASP9, IL6, MAPK8, PIK3CA, CASP3, IL1B, CYCS, MAPK1, BAX, MAPK3	73	268	8998	6.8988959	4.41E-06	7.12E-08
KEGG_PATHWAY	mmu05134:Legionellosis	11.68831	9	3.16E-08	2.37E-08	CASP9, IL6, IL1B, CASP3, CYCS, TNF, TLR4, NFKB1, RELA	73	61	8998	18.185942	5.33E-06	8.46E-08
KEGG_PATHWAY	mmu05321:Inflammatory bowel disease	11.68831	9	3.55E-08	2.70E-08	IL10, IL6, JUN, TGFB1, IL1B, TNF, TLR4, NFKB1, RELA	73	62	8998	17.89262	6.08E-06	9.50E-08
KEGG_PATHWAY	mmu04014:Ras signaling pathway	18.18182	14	4.41E-08	3.41E-08	BDNF, EGF, HGF, IGF1, FGF2, NFKB1, RELA, VEGFA, MAPK8, PIK3CA, AKT1, MAPK1, PLCG1, MAPK3	73	235	8998	7.3431653	7.68E-06	1.18E-07
KEGG_PATHWAY	mmu04071:Sphingolipid signaling pathway	14.28571	11	5.69E-08	4.47E-08	MAPK8, PIK3CA, BCL2, AKT1, BAX, MAPK1, MAPK14, TNF, NFKB1, RELA, MAPK3	73	124	8998	10.934379	1.01E-05	1.52E-07
KEGG_PATHWAY	mmu05211:Renal cell carcinoma	11.68831	9	7.13E-08	5.69E-08	JUN, TGFB1, PIK3CA, HGF, AKT1, MAPK1, HIF1A, MAPK3,	73	68	8998	16.31386	1.28E-05	1.91E-07
KEGG_PATHWAY	mmu05171:Coronavirus disease - COVID-19	18.18182	14	7.98E-08	6.46E-08	JUN, IFNB1, MAPK14, TNF, NFKB1, RELA, IL6, MAPK8, PIK3CA, IL1B, MAPK1, PLCG1, TLR4, MAPK3	73	248	8998	6.9582413	1.45E-05	2.14E-07
KEGG_PATHWAY	mmu04917:Prolactin signaling pathway	11.68831	9	1.36E-07	1.12E-07	MAPK8, PIK3CA, AKT1, MAPK1, PRL, MAPK14, NFKB1, RELA, MAPK3	73	74	8998	14.991114	2.51E-05	3.64E-07

The 463 potential targets of APS were intersected with 778 genes associated with irradition induced intestinal injury to obtain 82 genes, which were then analyzed for KEGG pathway enrichment.P-values are used to measure the degree of differential expression or enrichment of genes or metabolic pathways under different conditions; the smaller the P-value, the more significant the difference or enrichment.

Continued from above

KEGG_PATHWAY	mmu05220:Chronic myeloid leukemia	11.68831	9	1.66E-07	1.38E-07	TGFB1, PIK3CA, CDKN2A, AKT1, BAX, MAPK1, NFKB1, RELA, MAPK3	73	76	8998	14.596611	3.10E-05	4.43E-07
KEGG_PATHWAY	mmu05014:Amyotrophic lateral sclerosis	20.77922	16	1.90E-07	1.61E-07	BECN1, NDUFB8, NDUFA13, NOS2, EIF2AK3, MAPK14, TNF, MTOR, CASP9, ERN1, CASP3, CAT, BCL2, CYCS, BAX, NOS1	73	369	8998	5.3446189	3.61E-05	5.09E-07
KEGG_PATHWAY	mmu05207:Chemical carcinogenesis - receptor activation	16.88312	13	2.13E-07	1.82E-07	JUN, EGF, XIAP, FGF2, MTOR, NFKB1, RELA, VEGFA, PIK3CA, BCL2, AKT1, MAPK1, MAPK3	73	225	8998	7.1217047	4.10E-05	5.70E-07
KEGG_PATHWAY	mmu04218:Cellular senescence	15.58442	12	2.28E-07	1.98E-07	IL6, TGFB1, PIK3CA, CDKN2A, AKT1, MAPK1, MAPK14, SIRT1, NFKB1, RELA, MTOR, MAPK3	73	184	8998	8.0387135	4.46E-05	6.10E-07
KEGG_PATHWAY	mmu05224:Breast cancer	14.28571	11	2.55E-07	2.25E-07	JUN, PIK3CA, SP1, EGF, AKT1, BAX, MAPK1, IGF1, FGF2, MTOR, MAPK3	73	147	8998	9.2235579	5.06E-05	6.84E-07
KEGG_PATHWAY	mmu05226:Gastric cancer	14.28571	11	3.04E-07	2.72E-07	TGFB1, PIK3CA, EGF, HGF, BCL2, AKT1, BAX, MAPK1, FGF2, MTOR, MAPK3	73	150	8998	9.0390868	6.11E-05	8.15E-07
KEGG_PATHWAY	mmu05144:Malaria	10.38961	8	3.31E-07	3.00E-07	IL10, IL6, TGFB1, IL1B, HGF, TLR9, TNF, TLR4	73	57	8998	17.299688	6.75E-05	8.86E-07
KEGG_PATHWAY	mmu04012:ErbB signaling pathway	11.68831	9	3.31E-07	3.03E-07	MAPK8, JUN, PIK3CA, EGF, AKT1, MAPK1, PLCG1, MTOR, MAPK3	73	84	8998	13.206458	6.82E-05	8.86E-07
KEGG_PATHWAY	mmu04510:Focal adhesion	15.58442	12	5.20E-07	4.83E-07	MAPK8, JUN, PIK3CA, EGF, HGF, BCL2, AKT1, XIAP, MAPK1, IGF1, MAPK3, VEGFA	73	201	8998	7.3588223	1.09E-04	1.39E-06
KEGG_PATHWAY	mmu04213:Longevity regulating pathway - multiple species	10.38961	8	5.75E-07	5.41E-07	PIK3CA, CAT, AKT1, IGF1, SOD2, SIRT1, MTOR, ATG5	73	62	8998	15.904551	1.22E-04	1.54E-06
KEGG_PATHWAY	mmu05016:Huntington disease	18.18182	14	6.65E-07	6.33E-07	BECN1, NDUFB8, NDUFA13, BDNF, SOD2, MTOR, CASP9, ERN1, MAPK8, SP1, CASP3, CYCS, BAX, PPARG	73	302	8998	5.7140524	1.42E-04	1.78E-06
KEGG_PATHWAY	mmu05219:Bladder cancer	9.090909	7	7.94E-07	7.66E-07	CDKN2A, EGF, MMP2, MAPK1, MMP9, MAPK3, VEGFA	73	41	8998	21.044437	1.72E-04	2.13E-06

The 463 potential targets of APS were intersected with 778 genes associated with irradition induced intestinal injury to obtain 82 genes, which were then analyzed for KEGG pathway enrichment.P-values are used to measure the degree of differential expression or enrichment of genes or metabolic pathways under different conditions; the smaller the P-value, the more significant the difference or enrichment.

Continued from above

KEGG_PATHWAY	mmu04664:Fc epsilon RI signaling pathway	10.38961	8	8.55E-07	8.35E-07	MAPK8, PIK3CA, AKT1, MAPK1, PLCG1, MAPK14, TNF, MAPK3	73	66	8998	14.940639	1.88E-04	2.29E-06
KEGG_PATHWAY	mmu04137:Mitophagy - animal	10.38961	8	1.04E-06	1.03E-06	BECN1, MAPK8, JUN, SP1, EIF2AK3, HIF1A, RELA, ATG5	73	68	8998	14.501209	2.31E-04	2.78E-06
KEGG_PATHWAY	mmu04915:Estrogen signaling pathway	12.98701	10	1.06E-06	1.06E-06	POMC, JUN, PIK3CA, SP1, MMP2, BCL2, AKT1, MAPK1, MMP9, MAPK3	73	134	8998	9.1985279	2.38E-04	2.84E-06
KEGG_PATHWAY	mmu05221:Acute myeloid leukemia	10.38961	8	1.25E-06	1.25E-06	PIK3CA, AKT1, MAPK1, NFKB1, RELA, MTOR, MAPK3, PPARD	73	70	8998	14.086888	2.82E-04	3.32E-06
KEGG_PATHWAY	mmu04115:p53 signaling pathway	10.38961	8	1.52E-06	1.52E-06	CASP9, CDKN2A, CASP3, BCL2, CYCS, BAX, TNFRSF10B, IGF1	73	72	8998	13.695586	3.42E-04	3.98E-06
KEGG_PATHWAY	mmu04064:NF-kappa B signaling pathway	11.68831	9	1.70E-06	1.70E-06	IL1B, BCL2, XIAP, PLCG1, PTGS2, TNF, TLR4, NFKB1, RELA	73	105	8998	10.565166	3.82E-04	4.39E-06
KEGG_PATHWAY	mmu04931:Insulin resistance	11.68831	9	2.41E-06	2.41E-06	SREBF1, IL6, MAPK8, PIK3CA, AKT1, TNF, NFKB1, RELA,	73	110	8998	10.084932	5.43E-04	6.17E-06
KEGG_PATHWAY	mmu05168:Herpes simplex virus 1 infection	20.77922	16	2.58E-06	2.58E-06	IFNB1, EIF2AK3, TNF, MTOR, NFKB1, RELA, CASP9, IL6, PIK3CA, CASP3, IL1B, TLR9, BCL2, AKT1, CYCS, BAX	73	459	8998	4.2966544	5.80E-04	6.52E-06
KEGG_PATHWAY	mmu01523:Antifolate resistance	7.792208	6	2.91E-06	2.91E-06	ABCC3, IL6, IL1B, TNF, NFKB1, RELA	73	29	8998	25.502126	6.56E-04	7.29E-06
KEGG_PATHWAY	mmu04935:Growth hormone synthesis, secretion and action	11.68831	9	3.84E-06	3.84E-06	MAPK8, PIK3CA, AKT1, MAPK1, IGF1, PLCG1, MAPK14, MTOR, MAPK3	73	117	8998	9.4815595	8.64E-04	9.50E-06
KEGG_PATHWAY	mmu05165:Human papillomavirus infection	18.18182	14	4.80E-06	4.80E-06	IFNB1, EGF, PTGS2, TNF, MTOR, NFKB1, RELA, VEGFA, PIK3CA, CASP3, AKT1, MAPK1, BAX, MAPK3	73	362	8998	4.7669719	0.001078	1.17E-05
KEGG_PATHWAY	mmu04658:Th1 and Th2 cell differentiation	10.38961	8	5.92E-06	5.92E-06	MAPK8, JUN, MAPK1, PLCG1, MAPK14, NFKB1, RELA, MAPK3	73	88	8998	11.205479	0.001331	1.43E-05
KEGG_PATHWAY	mmu05213:Endometrial cancer	9.090909	7	6.19E-06	6.19E-06	CASP9, PIK3CA, EGF, AKT1, BAX, MAPK1, MAPK3	73	58	8998	14.87624	0.001392	1.48E-05

The 463 potential targets of APS were intersected with 778 genes associated with irradition induced intestinal injury to obtain 82 genes, which were then analyzed for KEGG pathway enrichment.P-values are used to measure the degree of differential expression or enrichment of genes or metabolic pathways under different conditions; the smaller the P-value, the more significant the difference or enrichment.

Continued from above

KEGG_PATHWAY	mmu04015:Rap1 signaling pathway	14.28571	11	6.94E-06	6.94E-06	PIK3CA, EGF, HGF, AKT1, MAPK1, IGF1, PLCG1, MAPK14, FGF2, MAPK3, VEGFA	73	214	8998	6.3358085	0.001561	1.64E-05
KEGG_PATHWAY	mmu04914:Progesterone-mediated oocyte maturation	10.38961	8	7.96E-06	7.96E-06	MAPK8, PIK3CA, AKT1, MAPK1, IGF1, MAPK14, MAPK3, CDC25B	73	92	8998	10.718285	0.00179	1.87E-05
KEGG_PATHWAY	mmu04024:cAMP signaling pathway	14.28571	11	8.87E-06	8.87E-06	POMC, MAPK8, JUN, PIK3CA, BDNF, AKT1, MAPK1, ATP2B1, NFKB1, RELA, MAPK3	73	220	8998	6.1630137	0.001993	2.06E-05
KEGG_PATHWAY	mmu04217:Necroptosis	12.98701	10	1.01E-05	1.01E-05	MAPK8, IFNB1, IL1B, BCL2, XIAP, BAX, TNFRSF10B, DNM1L, TNF, TLR4	73	176	8998	7.0034247	0.002266	2.31E-05
KEGG_PATHWAY	mmu04622:RIG-I-like receptor signaling pathway	9.090909	7	1.86E-05	1.86E-05	MAPK8, IFNB1, MAPK14, TNF, NFKB1, RELA, ATG5	73	70	8998	12.326027	0.004175	4.23E-05
KEGG_PATHWAY	mmu04920:Adipocytokine signaling pathway	9.090909	7	2.02E-05	2.02E-05	POMC, MAPK8, AKT1, TNF, NFKB1, RELA, MTOR	73	71	8998	12.152421	0.004531	4.54E-05
KEGG_PATHWAY	mmu04650:Natural killer cell mediated cytotoxicity	10.38961	8	3.44E-05	3.44E-05	PIK3CA, IFNB1, CASP3, TNFRSF10B, MAPK1, PLCG1, TNF, MAPK3	73	115	8998	8.5746278	0.007714	7.67E-05
KEGG_PATHWAY	mmu04930:Type II diabetes mellitus	7.792208	6	3.74E-05	3.74E-05	MAPK8, PIK3CA, MAPK1, TNF, MTOR, MAPK3	73	48	8998	15.407534	0.008369	8.24E-05
KEGG_PATHWAY	mmu04613:Neutrophil extracellular trap formation	12.98701	10	3.82E-05	3.82E-05	PIK3CA, AKT1, MAPK1, PLCG1, MAPK14, TLR4, NFKB1, RELA, MTOR, MAPK3	73	208	8998	5.9259747	0.008561	8.35E-05
KEGG_PATHWAY	mmu04662:B cell receptor signaling pathway	9.090909	7	4.30E-05	4.30E-05	JUN, PIK3CA, AKT1, MAPK1, NFKB1, RELA, MAPK3	73	81	8998	10.652122	0.009618	9.21E-05
KEGG_PATHWAY	mmu05012:Parkinson disease	14.28571	11	4.30E-05	4.30E-05	CASP9, ERN1, NDUFB8, NDUFA13, MAPK8, CASP3, EIF2AK3, CYCS, BAX, PLCG1, NFE2L2	73	264	8998	5.1358447	0.009626	9.21E-05
KEGG_PATHWAY	mmu04919:Thyroid hormone signaling pathway	10.38961	8	4.53E-05	4.53E-05	CASP9, PIK3CA, AKT1, MAPK1, PLCG1, HIF1A, MTOR, MAPK3	73	120	8998	8.2173516	0.010134	9.61E-05

The 463 potential targets of APS were intersected with 778 genes associated with irradition induced intestinal injury to obtain 82 genes, which were then analyzed for KEGG pathway enrichment.P-values are used to measure the degree of differential expression or enrichment of genes or metabolic pathways under different conditions; the smaller the P-value, the more significant the difference or enrichment.

Continued from above

KEGG_PATHWAY	mmu04630:JAK-STAT signaling pathway	11.68831	9	5.42E-05	5.42E-05	IL10, IL6, PIK3CA, IFNB1, EGF, BCL2, AKT1, PRL, MTOR	73	168	8998	6.603229	0.01211	1.14E-04
KEGG_PATHWAY	mmu05323:Rheumatoid arthritis	9.090909	7	6.44E-05	6.44E-05	IL6, JUN, TGFB1, IL1B, TNF, TLR4, VEGFA	73	87	8998	9.9174933	0.014376	1.34E-04
KEGG_PATHWAY	mmu05017:Spinocerebellar ataxia	10.38961	8	1.26E-04	1.26E-04	ERN1, BECN1, MAPK8, PIK3CA, SP1, AKT1, CYCS, MTOR	73	141	8998	6.9934907	0.027929	2.60E-04
KEGG_PATHWAY	mmu05230:Central carbon metabolism in cancer	7.792208	6	2.15E-04	2.15E-04	PIK3CA, AKT1, MAPK1, HIF1A, MTOR, MAPK3	73	69	8998	10.718285	0.047277	4.40E-04
KEGG_PATHWAY	mmu05143:African trypanosomiasis	6.493506	5	2.51E-04	2.51E-04	IL10, IL6, IL1B, TLR9, TNF	73	39	8998	15.802599	0.054886	5.08E-04
KEGG_PATHWAY	mmu05203:Viral carcinogenesis	11.68831	9	4.58E-04	4.58E-04	JUN, PIK3CA, CDKN2A, CASP3, BAX, MAPK1, NFKB1, RELA, MAPK3	73	229	8998	4.8442902	0.097998	9.21E-04
KEGG_PATHWAY	mmu04152:AMPK signaling pathway	9.090909	7	5.12E-04	5.12E-04	SREBF1, PIK3CA, AKT1, PPARG, IGF1, SIRT1, MTOR	73	127	8998	6.7938734	0.108921	0.00102
KEGG_PATHWAY	mmu04912:GnRH signaling pathway	7.792208	6	7.39E-04	7.39E-04	MAPK8, JUN, MMP2, MAPK1, MAPK14, MAPK3	73	90	8998	8.2173516	0.153269	0.001459
KEGG_PATHWAY	mmu04371:Apelin signaling pathway	9.090909	7	7.65E-04	7.65E-04	BECN1, NOS2, AKT1, MAPK1, NOS1, MTOR, MAPK3	73	137	8998	6.2979702	0.158205	0.001497
KEGG_PATHWAY	mmu04910:Insulin signaling pathway	9.090909	7	8.26E-04	8.26E-04	SREBF1, MAPK8, PIK3CA, AKT1, MAPK1, MTOR, MAPK3	73	139	8998	6.2073519	0.169585	0.001601
KEGG_PATHWAY	mmu04550:Signaling pathways regulating pluripotency of stem cells	9.090909	7	8.57E-04	8.57E-04	PIK3CA, AKT1, MAPK1, IGF1, MAPK14, FGF2, MAPK3	73	140	8998	6.1630137	0.175468	0.001648
KEGG_PATHWAY	mmu04350:TGF-beta signaling pathway	7.792208	6	9.45E-04	9.45E-04	TGFB1, SP1, MAPK1, TNF, DCN, MAPK3	73	95	8998	7.7848594	0.191593	0.001802
KEGG_PATHWAY	mmu04723:Retrograde endocannabinoid signaling	9.090909	7	0.001145	0.001145	NDUFB8, NDUFA13, MAPK8, MAPK1, MAPK14, PTGS2, MAPK3	73	148	8998	5.8298778	0.227162	0.002164
KEGG_PATHWAY	mmu04072:Phospholipase D signaling pathway	9.090909	7	0.001185	0.001185	PIK3CA, EGF, AKT1, MAPK1, PLCG1, MTOR, MAPK3	73	149	8998	5.7907511	0.234194	0.002222

The 463 potential targets of APS were intersected with 778 genes associated with irradition induced intestinal injury to obtain 82 genes, which were then analyzed for KEGG pathway enrichment.P-values are used to measure the degree of differential expression or enrichment of genes or metabolic pathways under different conditions; the smaller the P-value, the more significant the difference or enrichment.

Continued from above

KEGG_PATHWAY	mmu04150:mTOR signaling pathway	9.090909	7	0.001551	0.001551	PIK3CA, AKT1, MAPK1, IGF1, TNF, MTOR, MAPK3	73	157	8998	5.495681	0.294767	0.002884
KEGG_PATHWAY	mmu04623:Cytosolic DNA-sensing pathway	6.493506	5	0.001572	0.001572	IL6, IFNB1, IL1B, NFKB1, RELA	73	63	8998	9.7825614	0.298171	0.0029
KEGG_PATHWAY	mmu05202:Transcriptional misregulation in cancer	10.38961	8	0.002012	0.002012	IL6, SP1, BAX, PPARG, IGF1, MMP9, NFKB1, RELA	73	224	8998	4.4021526	0.364421	0.003681
KEGG_PATHWAY	mmu04020:Calcium signaling	10.38961	8	0.002967	0.002967	NOS2, EGF, HGF, PLCG1, ATP2B1, NOS1, FGF2, VEGFA	73	240	8998	4.1086758	0.487549	0.005384
KEGG_PATHWAY	mmu05216:Thyroid cancer	5.194805	4	0.003141	0.003141	BAX, MAPK1, PPARG, MAPK3	73	37	8998	13.325435	0.507275	0.005654
KEGG_PATHWAY	mmu04611:Platelet activation	7.792208	6	0.003179	0.003179	PIK3CA, AKT1, MAPK1, MAPK14, MAPK3, PTGS1	73	125	8998	5.9164932	0.511491	0.005677
KEGG_PATHWAY	mmu04960:Aldosterone-regulated sodium reabsorption	5.194805	4	0.003391	0.003391	PIK3CA, MAPK1, IGF1, MAPK3	73	38	8998	12.974766	0.534291	0.005983
KEGG_PATHWAY	mmu04750:Inflammatory mediator regulation of TRP channels	7.792208	6	0.003404	0.003404	MAPK8, PIK3CA, IL1B, IGF1, PLCG1, MAPK14	73	127	8998	5.82332	0.535673	0.005983
KEGG_PATHWAY	mmu04062:Chemokine signaling pathway	9.090909	7	0.004243	0.004243	PIK3CA, AKT1, MAPK1, PLCG1, NFKB1, RELA, MAPK3	73	192	8998	4.4938642	0.615831	0.0074
KEGG_PATHWAY	mmu04666:Fc gamma R-mediated phagocytosis	6.493506	5	0.00644	0.00644	PIK3CA, AKT1, MAPK1, PLCG1, MAPK3	73	93	8998	6.6268965	0.766296	0.011146
KEGG_PATHWAY	mmu05030:Cocaine addiction	5.194805	4	0.006567	0.006567	JUN, BDNF, NFKB1, RELA	73	48	8998	10.27169	0.7729	0.011279
KEGG_PATHWAY	mmu04261:Adrenergic signaling in cardiomyocytes	7.792208	6	0.007265	0.007265	BCL2, AKT1, MAPK1, ATP2B1, MAPK14, MAPK3	73	152	8998	4.8655371	0.806141	0.012384
KEGG_PATHWAY	mmu04060:Cytokine-cytokine receptor	10.38961	8	0.00857	0.00857	IL10, IL6, TGFB1, IFNB1, IL1B, TNFRSF10B, PRL, TNF	73	292	8998	3.3769938	0.855795	0.014498

The 463 potential targets of APS were intersected with 778 genes associated with irradition induced intestinal injury to obtain 82 genes, which were then analyzed for KEGG pathway enrichment.P-values are used to measure the degree of differential expression or enrichment of genes or metabolic pathways under different conditions; the smaller the P-value, the more significant the difference or enrichment.

Continued from above

KEGG_PATHWAY	mmu04923:Regulation of lipolysis in adipocytes	5.194805	4	0.010555	0.010555	PIK3CA, AKT1, PTGS2, PTGS1	73	57	8998	8.6498438	0.908145	0.017723
KEGG_PATHWAY	mmu04141:Protein processing in endoplasmic reticulum	7.792208	6	0.012019	0.012019	ERN1, MAPK8, EIF2AK3, BCL2, BAX, NFE2L2	73	172	8998	4.299777	0.934172	0.020032
KEGG_PATHWAY	mmu04730:Long-term depression	5.194805	4	0.012136	0.012136	MAPK1, IGF1, NOS1, MAPK3	73	60	8998	8.2173516	0.935903	0.020078
KEGG_PATHWAY	mmu04725:Cholinergic synapse	6.493506	5	0.012233	0.012233	PIK3CA, BCL2, AKT1, MAPK1, MAPK3	73	112	8998	5.5026908	0.937301	0.020091
KEGG_PATHWAY	mmu04929:GnRH secretion	5.194805	4	0.013846	0.013846	PIK3CA, AKT1, MAPK1, MAPK3	73	63	8998	7.8260491	0.956596	0.022576
KEGG_PATHWAY	mmu04670:Leukocyte transendothelial	6.493506	5	0.014585	0.014585	PIK3CA, MMP2, PLCG1, MAPK14, MMP9	73	118	8998	5.222893	0.963332	0.023609
KEGG_PATHWAY	mmu04726:Serotonergic synapse	6.493506	5	0.020617	0.020617	CASP3, MAPK1, PTGS2, MAPK3, PTGS1	73	131	8998	4.7045906	0.990789	0.033135
KEGG_PATHWAY	mmu04136:Autophagy - other	3.896104	3	0.026831	0.026831	BECN1, MTOR, ATG5	73	32	8998	11.555651	0.9978	0.042815
KEGG_PATHWAY	mmu05410:Hypertrophic cardiomyopathy	5.194805	4	0.036178	0.036178	IL6, TGFB1, IGF1, TNF	73	91	8998	5.418034	0.999749	0.057325
KEGG_PATHWAY	mmu04061:Viral protein interaction with cytokine and cytokine receptor	5.194805	4	0.040307	0.040307	IL10, IL6, TNFRSF10B, TNF	73	95	8998	5.1899063	0.999905	0.062873
KEGG_PATHWAY	mmu04216:Ferroptosis	3.896104	3	0.040507	0.040507	GCLC, HMOX1, ATG5	73	40	8998	9.2445205	0.999909	0.062873
KEGG_PATHWAY	mmu04934:Cushing syndrome	6.493506	5	0.040518	0.040518	POMC, CDKN2A, SP1, MAPK1, MAPK3	73	162	8998	3.8043294	0.999909	0.062873
KEGG_PATHWAY	mmu04672:Intestinal immune network for IgA production	3.896104	3	0.046185	0.046185	IL10, IL6, TGFB1	73	43	8998	8.599554	0.999976	0.071176
KEGG_PATHWAY	mmu04928:Parathyroid hormone synthesis, secretion and action	5.194805	4	0.055289	0.055289	SP1, BCL2, MAPK1, MAPK3	73	108	8998	4.5651953	0.999997	0.084625

The 463 potential targets of APS were intersected with 778 genes associated with irradition induced intestinal injury to obtain 82 genes, which were then analyzed for KEGG pathway enrichment.P-values are used to measure the degree of differential expression or enrichment of genes or metabolic pathways under different conditions; the smaller the P-value, the more significant the difference or enrichment.

Continued from above

KEGG_PATHWAY	mmu00330:Arginine and proline metabolism	3.896104	3	0.069228	0.069228	OAT, NOS2, NOS1	73	54	8998	6.847793	1	0.105246
KEGG_PATHWAY	mmu04114:Oocyte meiosis	5.194805	4	0.072555	0.072555	MAPK1, IGF1, MAPK14, MAPK3	73	121	8998	4.0747198	1	0.109563
KEGG_PATHWAY	mmu04110:Cell cycle	5.194805	4	0.078302	0.078302	PCNA, TGFB1, CDKN2A, CDC25B	73	125	8998	3.9443288	1	0.117453
KEGG_PATHWAY	mmu05332:Graft-versus-host	3.896104	3	0.090302	0.090302	IL6, IL1B, TNF	73	63	8998	5.8695369	1	0.134555
KEGG_PATHWAY	mmu04810:Regulation of actin cytoskeleton	6.493506	5	0.099221	0.099221	PIK3CA, EGF, MAPK1, FGF2, MAPK3	73	220	8998	2.8013699	1	0.146874

The 463 potential targets of APS were intersected with 778 genes associated with irradition induced intestinal injury to obtain 82 genes, which were then analyzed for KEGG pathway enrichment.P-values are used to measure the degree of differential expression or enrichment of genes or metabolic pathways under different conditions; the smaller the P-value, the more significant the difference or enrichment.
