# Supplementary material for: Astragalus polysaccharide promotes the regeneration of intestinal stem cells through HIF‐1 signalling pathway
Source: J Cell Mol Med. 2023 Dec 14;28(3):e18058. doi: 10.1111/jcmm.18058 (PMC10844761; doi:10.1111/jcmm.18058)
Supplement: Supplementary file 4 — Table S4. [file JCMM-28-e18058-s003.pdf]

Total gene results obtained from transcriptome sequencing

id	APS1	APS2	APS3	APS4	MOD1	MOD2	MOD3	MOD4
Akt1	12483	11796	11696	12930	6943	6470	5771	6409
Bcl2	134	174	147	192	157	199	184	173
Egf	13	23	32	8	30	14	12	19
Hif1a	7719	7164	7873	7676	4374	4395	4480	4248
Hmox1	148	104	130	140	142	137	106	137
Igf1	226	219	269	221	264	270	199	202
Il6	0	0	1	0	0	0	1	0
Mapk1	6037	6605	6167	5847	6439	6655	6404	6180
Mapk3	13054	13892	13037	13057	12747	12644	12516	12097
Mtor	2318	2453	2320	2439	2231	2329	2141	2137
Nfkb1	7171	7626	7306	7355	3472	3609	3233	3243
Nos2	2809	3042	1962	1827	2348	3035	3254	2836
Pik3ca	1225	1413	1385	1356	1480	1543	1428	1448
Plcg1	545	607	554	557	438	552	530	525
Rela	1586	1686	1545	1448	1594	1579	1575	1556
Tlr4	29	46	53	55	65	78	103	43
Vegfa	2757	2889	2783	2721	3047	3455	3370	3011
0610009B	702	808	715	693	733	897	773	778
0610010F	259	364	314	376	293	469	378	313
0610010K	545	600	546	529	576	552	461	527
0610012G	2340	2115	2475	2525	2115	2166	2106	2071
0610030E	1210	1375	1293	1255	1473	1540	1463	1398
0610040J	2809	2728	2497	2746	2479	2562	2660	2409
1110002E	2	7	48	9	2	11	0	1
1110004F	1538	1659	1430	1453	1529	1379	1223	1426
1110008P	633	565	665	622	675	617	561	609
1110017D	0	0	1	0	1	0	0	0
1110025M	85	86	45	50	51	61	31	39
1110032A	528	498	422	544	471	580	538	526
1110032F	24	21	10	23	29	10	23	7
1110038F	611	734	585	626	489	470	430	504
1110051M	142	161	175	143	103	142	174	155
1110059E	1112	983	1052	1151	1073	1156	1134	1062
1110059G	655	621	771	722	647	602	694	608
1110065P	424	328	403	399	338	439	347	350
1190005I	1	0	0	0	0	0	0	0
1190007I	260	289	299	223	298	288	245	232
1300017J	1	2	2	1	2	1	3	2
1500009L	66	106	96	51	61	82	74	58
1520401A	0	1	11	1	1	1	1	2
1600012H	1461	1631	1440	1463	1781	1700	1718	1534
1600014C	2162	2486	2134	2035	2431	2370	2175	2185
1600014C	0	0	1	0	0	0	0	0
1700001C	32	47	47	60	32	17	44	18
1700001J	3	0	3	3	8	1	2	2
1700001K	0	0	2	1	1	0	0	2
1700001O	2	6	3	15	6	4	6	7
1700001P	0	0	0	6	0	0	1	1
1700003E	11	8	6	4	6	5	5	4
1700003F	0	0	4	2	2	1	0	1
1700007K	4	0	2	4	1	0	0	1
1700008O	1	3	5	4	1	3	2	0
1700010B	0	0	1	0	0	0	0	0
1700010I1	19	16	2	12	6	18	11	25
1700011L	1	0	0	0	0	0	0	0
1700012B	0	1	0	1	0	0	0	1
1700013H	0	0	0	0	0	1	0	0

Transcriptome sequencing yielded total genetic results for the MOD and APS groups, with a total of 15,936 variables

Continued from above

1700014D	1	0	0	0	1	0	2	2
1700016C	25	32	20	19	9	31	32	30
1700016D	0	0	0	2	0	0	0	0
1700016H	0	6	1	0	0	1	0	0
1700016K	5	9	22	19	6	30	4	22
1700017B	869	1044	851	983	906	982	949	898
1700019A	0	2	4	0	0	0	0	0
1700019D	133	164	132	147	97	83	144	110
1700020A	0	0	0	0	0	0	4	0
1700020L	48	58	52	27	42	47	55	33
1700024J	0	4	0	0	0	0	0	0
1700025G	699	815	765	700	622	662	606	612
1700028K	1	0	1	1	0	0	0	0
1700029I1	33	44	53	48	58	62	42	55
1700029J	8	7	13	2	8	1	7	3
1700030J	45	53	41	16	39	21	47	19
1700030K	52	53	41	41	60	60	76	67
1700037C	132	52	97	72	77	66	84	81
1700037H	571	577	662	674	511	585	524	502
1700056E	11	9	14	26	5	8	6	6
1700066B	270	325	273	315	328	295	300	302
1700066M	150	148	148	184	157	190	156	195
1700067K	5	3	2	0	5	0	2	3
1700088E	41	42	38	55	44	17	38	45
1700092M	2	1	0	2	0	6	1	3
1700093K	0	0	0	1	0	0	0	0
1700094D	47	46	78	65	47	82	63	74
1700102P	8	7	25	11	19	9	8	5
1700109H	5	19	8	19	17	7	11	3
1700122O	1	0	0	0	0	0	0	1
1700123O	1351	1343	1263	1373	1356	1464	1434	1297
1700125H	0	0	2	0	0	3	0	0
1810009A	0	1	0	0	0	2	0	1
1810009J	28	19	1	0	32	76	29	51
1810010H	78	97	83	91	63	53	37	56
1810013L	1562	1676	1518	1610	1729	1726	1795	1850
1810020O	7	4	1	1	14	3	1	2
1810024B	8	14	3	7	7	9	10	8
1810030O	988	1134	1197	1067	1315	1170	1028	946
1810037I1	1734	1712	1718	1778	1884	1740	1701	1717
1810046K	260	247	312	310	213	204	179	195
1810055G	4963	5222	4986	4708	4827	5228	5356	5127
1810058I2	952	1161	977	970	1126	1130	1010	954
1810062G	0	2	3	2	3	2	5	1
1810065E	78	122	124	89	194	178	114	176
2010003K	4537	4219	4167	4657	3482	3942	4004	3914
2010106E	7851	9394	7805	6968	8928	10197	11235	9084
2010109A	1	4	0	0	1	6	1	12
2010315B	758	728	634	715	680	664	721	706
2200002D	8232	8822	8045	8226	7699	8199	8099	7802
2200002J	81	46	84	74	39	49	38	48
2210010C	3163	4431	1910	875	15806	13566	10493	13526
2210016F	8136	8106	7968	8201	7517	7847	7973	7953
2210016L	578	638	595	618	601	585	568	537
2210408I2	30	48	36	46	39	34	23	28
2210418O	3	2	10	0	8	2	33	5
2300009A	281	244	319	282	251	221	262	188
2310009B	129	124	133	165	124	129	91	108

Transcriptome sequencing yielded total genetic results for the MOD and APS groups, with a total of 15,936 variables

Continued from above

2310011J(	2276	2214	2165	2109	2375	2245	2179	1999
2310022A	360	335	334	420	312	333	282	324
2310022B(	778	851	925	889	1008	799	868	879
2310030G	882	895	857	853	885	1024	886	837
2310033P(	474	456	440	504	449	516	472	485
2310039H	466	393	469	493	459	400	344	438
2310057M	146	208	208	237	154	215	177	214
2310061I(	2451	2329	2367	2374	2279	2171	2116	2158
2410002F2	730	830	782	769	721	638	659	636
2410004B:	987	897	854	882	1011	940	919	848
2410004P(	1	5	4	3	1	2	2	2
2510002D	403	331	441	396	431	411	384	356
2510009E(	136	168	170	160	166	155	158	151
2510022D	563	527	556	540	563	432	409	448
2510039O	6028	6025	5717	5637	5916	5835	5698	5606
2610001J(	563	603	581	629	558	618	555	569
2610002M	1301	1129	1225	1150	1259	1123	1260	1072
2610008E:	150	198	157	182	113	150	141	165
2610028H	2	1	0	0	2	0	2	8
2610042L(	2	0	0	0	0	0	0	0
2610044O	107	106	124	164	102	129	101	101
2610301B:	86	87	112	106	69	123	103	85
2610318N	63	77	61	54	58	86	54	83
2610507B:	14748	15541	14916	15197	14568	14626	13988	13317
2610524H	48	62	39	42	29	38	32	30
2610528A	23	19	32	40	54	17	18	19
2610528J1	2039	2054	1985	1932	2011	2123	1808	1946
2700049A	255	220	218	250	200	219	200	212
2700062C	177	179	180	166	172	199	155	185
2700081O	248	227	213	248	229	233	189	199
2700097O	188	199	174	136	205	182	151	215
2810004N	460	491	466	452	524	517	486	438
2810006K:	162	167	199	154	267	218	224	194
2810021J2	174	176	182	136	128	140	124	160
2810408A	182	197	252	169	137	121	141	194
2810459M	1052	906	862	980	803	856	814	774
2900026A	11013	11753	11216	11153	11003	11846	11769	10503
3110009E:	99	83	95	91	85	112	82	64
3110040M	0	1	0	0	0	0	0	0
3110040N	487	398	396	379	478	518	453	480
3110082I1	232	159	230	167	183	172	183	173
3300002I(	724	767	866	1085	894	882	914	862
3425401B:	0	0	1	0	0	4	1	0
3830403N	10	8	7	1	7	13	26	12
3830406C	681	715	641	695	677	705	651	617
4430402I1	249	233	264	214	152	199	148	161
4833420G	902	1023	1003	1079	866	928	890	847
4833439L:	7558	8219	7571	7190	6841	7644	7793	7356
4921504E(	0	0	0	0	0	0	0	1
4921507P(	6	9	13	3	7	10	15	17
4921524J1	222	222	226	239	308	307	262	233
4921528I(	0	0	0	1	0	0	0	0
4921536K:	8	10	9	6	7	12	0	4
4921539E:	2	0	0	0	0	1	0	4
4930402H	3147	3450	3207	2959	3123	3369	3140	2786
4930404N	48	38	33	26	12	28	33	6
4930415O	55	46	56	63	79	66	84	79
4930430F(	76	89	91	61	86	113	73	79

Transcriptome sequencing yielded total genetic results for the MOD and APS groups, with a total of 15,936 variables

Continued from above

4930432K	1	4	2	0	1	8	0	4
4930438A	0	0	1	0	0	0	0	0
4930444P	0	0	0	1	0	0	0	0
4930447C	0	0	0	0	0	0	4	0
4930453N	645	698	610	644	628	702	641	620
4930467E	9	4	30	23	23	7	11	22
4930486L	0	3	3	1	5	0	1	1
4930503B	0	2	6	1	7	0	5	1
4930503L	64	60	93	98	70	64	63	82
4930522L	84	75	93	104	107	94	99	118
4930523C	153	199	144	178	137	109	123	91
4930524J	14	13	14	10	15	6	12	17
4930548H	0	0	0	0	2	0	0	3
4930550C	0	4	2	5	2	7	0	0
4930562C	0	1	0	0	1	2	0	0
4930563E	8	1	5	4	4	1	2	1
4930579G	59	81	51	102	76	81	52	71
4930590J	9	6	6	13	9	14	11	8
4931406C	7689	8992	7189	6042	10694	11597	11228	10197
4931414P	236	281	251	250	339	280	191	284
4931422A	3	4	6	0	4	14	9	2
4931428F	19	62	54	48	63	48	74	26
4931429L	2	0	0	0	0	0	0	0
4932414N	1	4	4	0	2	1	5	0
4932438A	4265	4929	4379	4396	4603	5119	5009	4153
4932438H	54	67	49	59	21	38	52	49
4933407O	3	2	0	5	6	4	1	1
4933411K	0	0	0	0	4	1	10	4
4933417A	45	29	51	42	40	41	41	50
4933427D	262	279	253	239	251	264	292	228
4933428G	0	3	0	0	0	0	0	7
4933430I1	24	23	6	5	12	33	21	18
4933434E	1506	1621	1416	1569	1639	1574	1504	1531
4933440N	165	140	137	126	190	134	126	127
5031439G	1952	2041	2069	2084	1947	1963	1912	2007
5430403G	9	8	12	1	11	2	11	1
5730409E	60	67	87	43	68	87	42	88
5730455P	358	324	340	376	345	355	320	282
5730507C	161	227	227	200	190	202	187	176
5830411N	2	3	0	1	2	0	6	2
6030458C	780	826	748	765	838	789	701	694
6030468B	0	2	0	0	0	0	0	0
6330403K	34	46	23	43	53	22	25	27
6330409D	0	0	0	0	2	0	0	2
6430548M	539	515	562	539	626	675	524	638
6430550D	19	20	14	28	32	14	8	25
6430571L	0	2	1	0	0	1	1	1
6720489N	16	14	13	23	24	32	10	25
6820408C	0	0	0	0	1	0	1	1
8030462N	702	692	712	658	764	778	585	708
8030474K	0	0	0	0	6	8	5	0
9030025P	33	55	27	51	20	22	116	39
9030624G	85	67	31	57	71	42	49	65
9130008F	968	682	868	1200	643	474	534	513
9130019O	405	451	456	425	363	396	403	430
9130023H	230	226	215	250	229	202	127	219
9130401M	250	296	208	307	293	281	309	217
9130409I2	0	0	0	0	2	4	2	0

Transcriptome sequencing yielded total genetic results for the MOD and APS groups, with a total of 15,936 variables

Continued from above

9230112D	0	2	5	0	5	9	1	2
9330159F1	0	10	3	0	4	2	4	6
9430015G	180	225	204	190	235	267	186	245
9430038I0	1127	1039	1087	1030	1013	1153	1200	1179
9430069I0	7	4	2	2	2	3	1	1
9430078G	7	12	25	27	20	10	26	12
9530053A	22	21	22	16	14	27	10	10
9530068E0	2688	2978	2878	2666	2825	2983	2545	2753
9530077C	7	2	3	5	18	3	7	8
9830107B1	0	2	1	5	6	6	2	0
9930012K1	50	37	47	61	29	47	58	29
9930021J0	1417	1489	1376	1463	1301	1322	1385	1230
9930104L0	210	195	188	211	273	271	181	176
9930111J2	16	21	37	21	18	16	23	13
9930111J2	0	2	6	5	7	2	8	6
A130010J1	402	469	460	374	466	391	392	386
A1bg	0	1	0	0	0	5	25	0
A1cf	5522	7041	5903	5600	7226	7714	7124	6384
A2m	1	0	0	10	0	0	1	0
A2ml1	9	15	16	18	18	11	23	27
A3galt2	14	2	0	8	2	2	1	2
A430005L	636	726	701	742	646	728	675	662
A430033K	99	89	105	106	106	127	87	114
A430057M	50	72	98	105	51	58	43	54
A4galt	96	125	113	87	91	82	124	84
A4gnt	44	23	69	53	60	50	30	66
A530016L1	4	4	4	1	2	5	10	1
A530032D	9	10	3	3	1	3	3	1
A530064D	1	0	7	0	8	10	4	5
A630001G	22	21	22	12	22	11	16	13
A630023A	0	0	2	0	0	0	0	0
A730008H	555	647	580	590	557	642	641	474
A730046J1	0	0	1	0	0	0	0	2
A830018L	0	0	8	6	0	0	2	0
A930007A	23	11	17	12	14	12	20	10
A930017K	0	2	0	1	0	0	0	0
A930018C	0	5	0	5	2	9	8	4
A930033H	8	11	2	3	6	2	9	11
AA414768	11	15	8	28	8	11	2	12
AA467197	11115	7382	9830	14078	4587	5005	5336	5332
AA986860	2374	2584	2610	2540	2559	2661	2528	2540
AB010352	68	76	95	82	60	104	90	63
AB124611	7	8	11	28	3	7	8	4
AI182371	2	11	3	3	2	2	2	5
AI413582	189	207	187	158	125	160	169	173
AI429214	17	2	5	17	17	3	6	1
AI467606	364	308	329	285	298	353	318	341
AI593442	1	2	1	0	1	1	0	1
AI597479	416	473	548	556	536	500	467	456
AI661453	3043	3390	3357	3078	3661	3565	3596	3143
AI837181	1127	1109	1072	982	989	1075	1023	1003
AI987944	176	209	186	195	197	246	242	238
AU021092	54	53	26	38	60	27	20	35
AU022252	295	349	368	346	351	318	363	327
AU040320	11540	12227	10719	10459	11046	11404	11746	10633
AU041133	61	87	38	57	70	47	86	68
AW146154	124	123	96	127	140	107	106	68
AW209491	279	271	310	222	301	324	292	240

Transcriptome sequencing yielded total genetic results for the MOD and APS groups, with a total of 15,936 variables

Continued from above

AW54987[illegible]	2396	2642	2595	2546	2440	2513	2490	2268
AW55198[illegible]	11	17	32	9	21	23	35	27
AW55491[illegible]	168	89	173	102	125	157	172	121
AY074887	10	7	14	5	12	9	7	2
AY358078	0	0	0	0	6	1	7	1
AY761185	0	0	0	0	0	0	2	0
Aaas	453	459	424	419	583	508	395	461
Aacs	2318	2397	2781	2498	2309	2170	2008	2151
Aadac	2669	2981	2781	2575	3440	3749	2943	3030
Aadat	1	0	0	0	1	1	1	0
Aagab	1541	1627	1588	1453	1614	1653	1557	1529
Aak1	2142	2504	2318	2308	2525	2719	2691	2425
Aamdc	1920	1883	1889	1984	2083	2199	2044	1791
Aamp	4629	4846	4868	4737	4958	5067	4527	4634
Aar2	1164	1185	1193	1136	1146	1113	1085	1079
Aard	13	30	13	34	30	32	31	10
Aars	5155	5297	4997	4973	4812	4467	4712	4315
Aars2	550	547	636	628	589	658	518	602
Aarsd1	572	609	632	520	529	493	511	477
Aasdh	160	119	127	89	134	147	99	133
Aasdhppt	434	470	427	486	493	495	550	453
Aass	7	5	8	11	6	4	18	6
Aatf	418	496	437	407	535	463	424	417
Aatk	52	54	67	45	76	31	42	36
Abat	3430	3983	3589	3751	3544	3356	3437	3302
Abca1	392	427	301	284	377	386	467	355
Abca13	2	0	6	10	1	0	1	1
Abca14	12	17	9	17	10	13	19	13
Abca2	839	894	833	750	730	802	747	581
Abca3	1522	1422	1485	1419	1468	1434	1369	1365
Abca4	1	0	6	0	0	7	2	1
Abca5	46	49	52	46	34	51	27	36
Abca6	4	8	2	4	8	10	2	2
Abca7	2316	2238	2211	2172	2217	2104	2152	2168
Abca8a	158	142	120	153	189	206	205	179
Abca8b	29	19	23	29	24	23	19	23
Abca9	72	94	89	65	86	83	60	74
Abcb10	3973	4043	3776	3891	3445	3561	3705	3567
Abcb11	11	15	5	11	14	23	16	13
Abcb1a	2414	3279	2484	2111	4196	4576	4288	3780
Abcb1b	42	24	57	51	33	43	53	30
Abcb4	3	3	3	1	0	8	0	0
Abcb6	923	859	946	870	962	895	772	808
Abcb7	2568	2867	2720	2696	2785	2985	2657	2529
Abcb8	3188	3087	3003	3400	3135	3145	2874	3000
Abcb9	889	871	939	819	1002	1077	941	964
Abcc1	409	430	410	401	403	336	386	369
Abcc10	820	904	876	916	977	875	884	815
Abcc12	0	5	8	7	5	2	1	2
Abcc2	5909	6719	6530	6359	7127	7641	8241	7567
Abcc3	6088	7341	6293	5729	7469	7636	7029	6799
Abcc4	210	231	224	214	237	240	212	212
Abcc5	283	278	304	287	332	269	262	254
Abcc6	381	341	384	338	466	417	413	328
Abcc8	48	42	48	62	52	24	74	26
Abcc9	292	295	344	343	342	274	309	293
Abcd1	7296	8011	6578	6036	7822	7828	8001	7492
Abcd2	18	21	14	10	32	21	29	15

Transcriptome sequencing yielded total genetic results for the MOD and APS groups, with a total of 15,936 variables

Continued from above

Abcd3	10159	11351	10619	10316	13053	12856	13068	12386
Abcd4	658	669	658	720	640	649	652	539
Abce1	2560	2786	2401	2434	3106	3093	2777	2645
Abcf1	5212	5456	5147	4911	5365	5672	5167	4796
Abcf2	1658	1846	1745	1718	1895	1687	1766	1771
Abcf3	1967	2008	1895	1921	2268	2113	1994	1945
Abcg1	78	124	132	99	96	93	71	77
Abcg2	5985	6194	4745	4482	5957	6353	6666	5958
Abcg3	192	164	127	206	188	193	189	172
Abcg4	6	4	11	6	7	18	8	9
Abcg5	8195	9209	7463	7374	8679	8739	8907	8222
Abcg8	7814	8714	7047	6593	7558	7392	7775	7357
Abhd10	333	296	407	357	291	287	297	279
Abhd11	1873	1846	1712	1761	1860	1794	1696	1627
Abhd12	4868	4800	4505	4684	4216	4286	4183	4017
Abhd12b	0	0	0	3	4	0	0	1
Abhd13	2488	2381	2417	2544	2804	3007	2817	2403
Abhd14a	562	513	561	525	588	475	431	425
Abhd14b	1461	1474	1489	1583	1415	1371	1202	1315
Abhd15	67	64	71	99	62	89	93	59
Abhd16a	2852	3002	3042	2763	2796	2656	2646	2659
Abhd16b	2	3	1	2	1	5	0	1
Abhd17a	6474	6273	6240	6197	6335	6115	5869	5923
Abhd17b	1650	1640	1659	1770	1658	1746	1609	1759
Abhd17c	6628	6954	6459	6116	6155	5994	6381	6561
Abhd18	747	802	845	824	858	914	955	798
Abhd2	5836	6367	5558	5492	6316	6896	8125	6585
Abhd3	5312	5772	5189	5087	5712	5722	5525	5185
Abhd4	4306	4509	4152	4058	3935	4207	4061	3853
Abhd5	802	899	822	801	955	992	898	800
Abhd6	3663	4148	3722	3297	4994	4775	4847	4563
Abhd8	170	152	130	151	168	182	143	135
Abi1	4630	4711	4384	4196	4828	4972	4715	4498
Abi2	51	64	52	77	43	68	79	48
Abi3	719	723	747	810	693	781	862	824
Abi3bp	85	86	86	89	98	117	120	99
Abitram	374	411	348	365	480	379	395	418
Abl1	4051	4568	4343	4229	4433	4338	4322	3985
Abl2	661	730	870	826	735	859	762	742
Ablim1	3157	3553	2939	2957	3095	3102	3099	3002
Ablim2	41	32	33	16	30	49	33	38
Ablim3	39	46	59	48	38	35	41	38
Abr	16689	17896	16072	15420	16814	17302	16885	15684
Abra	4	5	3	0	1	17	0	4
Abracl	1274	1242	1305	1296	1385	1520	1206	1232
Abraxas1	278	284	248	299	242	310	231	235
Abraxas2	1307	1370	1195	1282	1317	1306	1350	1225
Abt1	427	396	414	399	481	450	363	391
Abtb1	608	688	640	622	658	586	593	628
Abtb2	33	33	50	35	58	33	33	30
Acaa1a	12561	12863	12229	12336	12175	13300	13063	12419
Acaa1b	747	795	656	464	1185	1087	1100	1099
Acaa2	8207	9425	8717	7687	9773	10054	8563	7967
Acaca	1147	1251	1358	1237	1231	1181	1167	1115
Acacb	231	162	231	188	248	295	261	225
Acad10	473	511	448	403	455	449	459	475
Acad11	5997	6949	5840	5690	6959	7248	7383	6560
Acad12	303	301	294	227	317	368	293	282

Transcriptome sequencing yielded total genetic results for the MOD and APS groups, with a total of 15,936 variables

Continued from above

Acad8	763	910	892	938	964	923	843	804
Acad9	1194	1288	1277	1202	1344	1257	1305	1236
Acadl	17870	18952	18695	18417	18045	18236	18100	18114
Acadm	5949	5874	5637	5868	5523	6221	5910	5342
Acads	3428	3475	3303	3508	3617	3462	3138	3250
Acadsb	710	830	860	888	899	984	889	862
Acadvl	9891	9950	9810	10075	10024	10208	9445	9621
Acap1	131	87	120	90	77	105	85	111
Acap2	2660	2817	2321	2511	2544	2633	2749	2435
Acap3	341	326	336	371	304	313	368	399
Acat1	7161	7379	7390	7255	6960	7051	6492	5771
Acat2	1328	1331	1322	1363	1252	1283	1134	1304
Acat3	152	104	126	118	118	152	132	85
Acbd3	2192	2413	2354	2285	2450	2477	2473	2408
Acbd5	2255	2421	2279	2160	2412	2596	2787	2268
Acbd6	591	762	715	574	764	540	593	592
Acbd7	0	0	0	1	4	2	3	0
Accs	84	95	57	84	74	55	36	23
Accsl	0	0	0	1	0	1	0	0
Acd	339	342	308	368	315	373	324	394
Ace	63719	63213	57948	61757	50270	53810	62397	51045
Ace2	35674	36867	31938	33112	34373	38273	43368	36228
Ace3	3	9	1	10	0	2	6	1
Acer1	270	261	308	237	192	301	295	269
Acer2	320	384	439	380	428	413	353	405
Acer3	801	815	752	722	978	1023	899	999
Ache	218	241	229	313	203	227	196	231
Acin1	4014	4313	4221	3926	4177	4344	4024	3939
Ackr2	42	34	14	43	20	26	12	19
Ackr3	196	255	245	263	259	245	230	233
Ackr4	89	75	99	124	100	143	120	117
Acly	3051	3549	3655	3512	3538	3331	3184	3075
Acmsd	0	0	1	1	0	0	0	0
Acnat1	1931	1941	2029	2105	2099	2133	1997	1912
Acnat2	2	5	0	2	2	2	2	0
Aco1	9367	10455	9669	9076	9928	10436	10311	9354
Aco2	43481	43433	41790	43708	41828	42636	42189	40302
Acod1	18	15	16	22	13	4	2	8
Acot1	226	230	256	209	244	192	177	260
Acot10	0	1	1	0	5	2	1	0
Acot11	5394	6060	5465	5288	5956	5756	5864	5671
Acot12	1111	1432	931	633	1473	1529	1548	1366
Acot13	1543	1716	1661	1570	1765	1716	1651	1679
Acot2	191	224	179	198	194	202	153	201
Acot3	0	0	0	6	1	0	1	0
Acot4	753	731	720	746	769	786	773	700
Acot5	0	0	1	1	1	0	0	0
Acot6	88	105	80	83	90	74	73	67
Acot7	2070	2165	2289	2439	2447	2501	2240	2129
Acot8	1798	2023	1821	1716	2079	2086	1755	1871
Acot9	1123	1015	1018	1157	987	1085	1101	940
Acox1	32367	34162	31607	31577	33274	36150	36853	34239
Acox2	276	309	322	235	482	544	502	420
Acox3	4544	4492	3976	4328	4192	4167	4558	3986
Acoxl	0	0	7	1	0	6	0	0
Acp1	1929	1919	1926	1917	2074	2092	2000	2014
Acp2	2138	2246	1852	1945	2267	2195	2129	2003
Acp4	0	0	2	0	2	0	4	0

Transcriptome sequencing yielded total genetic results for the MOD and APS groups, with a total of 15,936 variables

Continued from above

Acp5	6894	7107	7172	7495	7149	7209	6750	7099
Acp6	1780	1753	1693	1660	1738	1808	1529	1593
Acpp	1057	1052	1080	1098	1209	1158	1033	1004
Acr	0	0	0	0	0	0	1	0
Acrbp	67	74	76	65	68	82	70	82
Acsbg1	20	18	35	6	4	16	11	10
Acsbg3	1	1	8	8	7	0	1	0
Acsf2	5013	3486	3603	5089	2510	2804	3372	3140
Acsf3	1894	1927	1774	1650	1663	1710	1755	1733
Acsl1	2066	2258	2175	2084	2329	2478	2551	2204
Acsl3	727	844	909	851	965	969	963	1028
Acsl4	346	371	436	393	350	393	407	291
Acsl5	61525	66634	58575	58087	65560	67088	69272	64230
Acsl6	36	27	59	45	26	56	30	33
Acsm1	1	0	0	0	1	0	0	2
Acsm3	123	194	209	198	204	223	203	172
Acsm5	139	165	130	160	121	128	145	155
Acss1	2819	2167	2557	3735	1636	1485	1534	1784
Acss2	1706	1750	1790	1723	1645	1510	1422	1641
Acss3	9	3	8	10	10	4	16	5
Acta1	655	811	778	612	946	998	1112	1025
Acta2	6182	6472	7427	7520	6567	7175	7690	7175
Actb	104395	110915	104458	104800	89871	97888	100789	98478
Actc1	16	24	13	28	20	10	20	6
Actg1	54517	54277	53208	53779	51027	50850	53740	53258
Actg2	4103	4042	5041	5326	3881	4219	4981	4599
Actl10	0	4	0	0	0	0	0	0
Actl11	1	0	1	2	0	0	1	0
Actl6a	1323	1487	1344	1337	1302	1303	1209	1237
Actl6b	6	2	6	8	0	2	4	4
Actl7b	0	0	1	0	0	0	4	0
Actn1	2876	3220	3273	3133	3165	3115	3050	2768
Actn2	15	24	21	28	21	21	26	48
Actn3	11	1	2	1	0	2	4	2
Actn4	48874	53183	46375	44449	48707	50206	50177	47258
Actr10	2043	2017	2077	2102	2071	2097	2038	2011
Actr1a	7510	7834	7728	7598	7408	7633	7274	7395
Actr1b	3800	4063	4182	4162	4028	4407	3965	4030
Actr2	9152	10305	9289	9658	10169	10489	10678	9940
Actr3	19608	21884	19820	18882	21481	22514	21631	20790
Actr3b	8	3	4	6	2	9	7	3
Actr5	297	328	309	347	297	368	277	283
Actr6	105	106	113	95	126	104	117	106
Actr8	638	809	728	690	719	633	682	589
Actrt2	0	0	1	0	0	0	0	0
Actrt3	0	0	0	0	0	0	0	1
Acvr1	496	436	472	446	424	493	483	407
Acvr1b	3082	3520	3480	3373	3317	3380	3279	3097
Acvr1c	22	51	25	43	28	27	16	26
Acvr2a	1303	1686	1441	1362	1702	1705	1643	1594
Acvr2b	223	213	161	200	223	164	186	229
Acvrl1	546	638	537	696	710	634	564	531
Acy1	6041	6375	6393	6193	5889	6441	6631	6292
Acy3	2290	2757	2155	2187	2795	2855	2878	2854
Acyp1	131	113	116	145	120	130	140	134
Acyp2	22	26	32	41	23	32	35	19
Ada	39408	38594	29368	31384	29395	34253	34010	29929
Adad1	0	0	0	0	0	0	0	1

Transcriptome sequencing yielded total genetic results for the MOD and APS groups, with a total of 15,936 variables

Continued from above

Adad2	20	14	26	7	15	17	27	9
Adal	205	180	226	229	268	260	212	195
Adam10	6232	6785	6118	6518	6716	6436	6408	6129
Adam11	33	34	44	38	47	25	43	40
Adam12	40	17	14	17	22	21	23	3
Adam15	963	963	980	995	1019	1020	945	891
Adam17	1319	1443	1343	1266	1498	1612	1564	1400
Adam19	501	456	417	538	451	452	401	415
Adam1a	87	105	110	146	113	70	121	126
Adam1b	0	0	0	0	0	0	0	1
Adam2	2	4	3	1	6	1	2	3
Adam21	3	1	2	2	4	0	2	1
Adam22	23	37	13	30	25	34	21	11
Adam23	14	27	22	44	19	25	22	15
Adam28	4	3	2	3	0	10	0	0
Adam3	0	0	1	0	0	0	0	0
Adam32	0	0	0	0	0	1	0	0
Adam33	20	13	21	11	30	31	37	27
Adam4	34	49	46	44	48	53	82	52
Adam5	0	0	8	1	1	1	0	6
Adam6b	0	0	0	0	4	0	0	1
Adam7	0	0	0	0	0	0	0	2
Adam8	73	52	60	75	55	58	98	55
Adam9	1216	1173	1209	1184	1382	1283	1268	1212
Adamdec1	4604	4786	4733	4561	4347	4357	3784	3660
Adamts1	196	221	255	254	194	202	182	204
Adamts10	174	205	196	263	206	231	235	221
Adamts12	77	62	81	99	68	52	74	42
Adamts13	22	28	32	10	20	26	22	17
Adamts14	37	39	60	37	23	31	44	30
Adamts15	784	695	864	866	643	475	481	584
Adamts16	0	4	0	0	1	0	0	0
Adamts17	40	63	53	32	50	26	28	30
Adamts18	11	20	23	11	17	13	9	12
Adamts19	18	10	24	8	6	16	17	7
Adamts2	148	135	179	183	153	178	150	153
Adamts20	1	0	0	1	4	0	8	0
Adamts3	25	26	10	15	10	11	17	19
Adamts4	141	191	117	141	142	134	118	152
Adamts5	33	21	18	26	20	18	29	25
Adamts6	27	29	44	25	27	28	15	35
Adamts7	63	47	81	42	48	56	54	36
Adamts8	34	20	21	47	45	32	37	44
Adamts9	95	88	125	104	121	130	149	108
Adamtsl1	47	79	67	40	40	78	59	45
Adamtsl2	12	12	22	17	27	12	23	19
Adamtsl3	31	55	33	52	32	36	45	43
Adamtsl4	245	248	233	171	178	245	248	232
Adamtsl5	2051	1945	2003	1924	1840	1907	1734	1832
Adap1	7968	7766	7427	7156	7535	7867	8129	7406
Adap2	2372	2289	2267	2351	1934	2013	2137	1959
Adar	3907	4909	4600	4144	4483	4258	3909	3889
Adarb1	18	23	39	45	34	35	20	46
Adat1	68	68	60	61	60	71	88	97
Adat2	140	107	77	82	71	92	108	115
Adat3	202	239	187	209	168	188	160	184
Adck1	433	475	400	381	440	463	409	404
Adck2	604	610	683	603	566	642	676	598

Transcriptome sequencing yielded total genetic results for the MOD and APS groups, with a total of 15,936 variables

Continued from above

Adck5	1906	2008	1866	1710	2009	1988	1849	1719
Adcy1	25	47	40	85	39	66	42	39
Adcy10	2	1	1	9	1	0	2	1
Adcy2	23	26	16	37	28	17	18	44
Adcy3	70	80	52	68	65	76	76	42
Adcy4	151	140	145	155	105	142	99	147
Adcy5	240	224	285	262	271	246	259	229
Adcy6	6107	6664	6662	6538	6250	6186	6071	5713
Adcy7	243	298	277	323	239	249	234	252
Adcy8	138	102	118	85	105	141	123	114
Adcy9	1008	1187	1054	1106	1096	1159	1044	982
Adcyap1	0	1	0	0	0	1	1	1
Adcyap1r1	45	55	37	54	29	45	51	48
Add1	6103	6292	6634	6532	5786	6017	5868	5670
Add2	10	6	11	15	13	22	11	9
Add3	3481	3973	4096	3970	4093	4108	3503	3269
Adgb	3	2	7	7	1	6	2	3
Adgra1	0	0	0	1	0	0	0	0
Adgra2	285	289	309	360	336	308	295	217
Adgra3	2316	2478	2359	2071	2602	2691	2464	2208
Adgrb1	90	63	53	70	27	41	47	38
Adgrb2	22	4	1	6	5	8	20	9
Adgrb3	1	6	2	0	10	2	8	5
Adgrd1	320	358	363	347	410	419	420	419
Adgre1	382	433	406	320	362	348	348	297
Adgre4	48	44	37	52	56	66	46	20
Adgre5	1414	1366	1647	1564	1554	1617	1510	1569
Adgrf1	42	80	67	66	37	46	12	40
Adgrf2	0	0	0	0	4	4	0	1
Adgrf3	1	3	3	1	1	1	0	4
Adgrf4	1	2	4	0	0	1	0	1
Adgrf5	699	730	759	729	648	692	585	597
Adgrg1	748	797	908	810	741	778	650	708
Adgrg2	3	10	3	2	6	5	10	2
Adgrg3	62	111	84	104	91	112	99	77
Adgrg4	100	141	99	111	84	104	129	99
Adgrg5	171	187	205	202	171	129	168	201
Adgrg6	126	103	89	117	133	133	169	111
Adgrg7	10575	11916	10464	10194	11727	13027	12685	10696
Adgrl1	281	352	275	340	277	209	335	254
Adgrl2	930	995	918	976	871	821	747	748
Adgrl3	103	127	119	123	116	145	131	117
Adgrl4	239	245	235	210	211	155	129	153
Adgrv1	59	41	56	50	71	75	49	49
Adh1	14460	17194	16816	14901	16861	17817	16722	15662
Adh4	843	1255	1148	964	1355	1298	945	843
Adh5	2903	3324	3176	3203	3308	3394	2986	2842
Adh6a	29345	33599	30553	27741	33042	35873	32584	29575
Adh6b	0	0	0	1	1	0	1	0
Adh7	32	30	45	25	22	41	47	38
Adhfe1	14	28	20	28	22	17	26	22
Adi1	525	564	536	463	515	587	566	502
Adig	0	3	8	9	12	13	11	8
Adipoq	7	2	27	21	60	101	69	44
Adipor1	6079	6461	6213	6172	6349	6275	6298	6128
Adipor2	25107	27268	24756	24589	29053	30406	28409	26356
Adk	1817	1835	1891	1713	1922	1918	1904	1806
Adm	118	167	117	125	116	132	152	155

Transcriptome sequencing yielded total genetic results for the MOD and APS groups, with a total of 15,936 variables

Continued from above

Adm2	5	0	10	2	1	1	1	2
Adnp	2328	2637	2505	2576	2374	2618	2396	2363
Adnp2	651	694	706	609	668	658	630	636
Ado	687	687	737	747	744	704	625	609
Adora1	65	83	74	73	43	68	44	42
Adora2a	68	74	57	75	47	63	92	64
Adora2b	428	376	410	450	427	417	424	326
Adora3	1	0	0	0	7	5	0	9
Adpgk	349	441	399	363	452	453	365	405
Adprh	1047	1094	1155	1066	1119	1070	1034	1096
Adprhl1	5	1	5	6	6	2	0	7
Adprhl2	447	485	492	516	539	547	453	495
Adprm	810	872	651	695	818	862	1005	974
Adra1a	2	1	0	2	3	3	0	4
Adra1b	2	5	0	6	8	0	16	4
Adra1d	6	1	1	4	6	0	0	0
Adra2a	395	423	430	422	455	430	391	445
Adra2b	4	7	4	3	5	1	2	11
Adra2c	7	3	10	15	8	17	2	14
Adrb1	26	25	31	28	30	13	25	23
Adrb2	16	26	20	37	37	18	23	18
Adrb3	6	34	21	34	55	34	33	29
Adrm1	3017	3120	2771	2857	3211	2992	2683	2557
Adsl	1352	1477	1193	1279	1629	1322	1306	1316
Adss	4011	4136	3804	3938	3953	4226	4136	3990
Adssl1	43	32	21	18	15	25	19	18
Adtrp	2389	2310	2270	2247	2758	3139	2743	2699
Aebp1	298	283	260	304	352	260	312	234
Aebp2	1831	2005	1869	1831	1734	1837	1797	1784
Aen	426	403	506	402	527	528	430	450
Afap1	138	118	145	117	108	110	83	106
Afap1l1	335	354	363	325	365	345	281	228
Afap1l2	110	112	112	127	93	94	111	68
Afdn	4474	4755	4570	4584	4156	4411	4259	4068
Aff1	2632	3141	2986	2883	3095	2909	3047	2791
Aff2	0	5	1	3	1	1	0	1
Aff3	46	50	22	36	35	38	44	23
Aff4	3211	3863	3694	3616	3808	4236	4329	3721
Afg1l	820	840	938	986	1079	950	949	818
Afg3l1	2088	1947	2041	1870	2154	2015	1940	2035
Afg3l2	7032	7135	7107	6959	6834	6967	6825	6646
Afm	2	4	6	1	13	20	15	10
Afmid	423	501	473	472	424	443	417	435
Afp	664	654	541	623	560	700	650	634
Aftph	2344	2741	2283	2280	2467	2789	2667	2231
Aga	443	513	514	479	476	444	362	469
Agap1	1413	1554	1591	1400	1502	1589	1580	1445
Agap2	87	56	110	62	66	63	54	72
Agap3	902	849	854	984	891	1019	937	948
Agbl2	1	1	4	1	5	6	7	7
Agbl3	14	18	16	24	4	3	13	10
Agbl4	2	0	0	0	0	0	0	0
Agbl5	311	288	368	312	267	236	256	266
Ager	14	15	22	16	24	6	22	18
Agfg1	3746	4484	3936	3692	4016	4346	4346	3943
Agfg2	350	346	353	361	331	330	312	379
Aggf1	2225	2381	2118	2323	2331	2474	2401	2039
Agk	267	272	294	245	244	313	288	277

Transcriptome sequencing yielded total genetic results for the MOD and APS groups, with a total of 15,936 variables

Continued from above

Agl	974	929	976	992	805	785	927	857
Agmat	618	841	718	578	750	747	711	761
Agmo	2897	3111	2536	2450	3266	3589	3555	2909
Ago1	867	1017	933	955	943	931	827	873
Ago2	2823	3305	3147	3149	3097	2998	3100	2902
Ago3	416	406	428	454	508	499	471	433
Ago4	46	64	70	66	69	38	45	79
Agpat1	7535	7241	6879	7269	6886	7206	6968	6878
Agpat2	10685	9816	9821	10968	9472	9835	9471	9376
Agpat3	5453	5825	5579	5125	5531	5957	5860	5745
Agpat4	266	239	203	257	227	193	188	220
Agpat5	1039	1049	909	992	1090	1105	1012	1074
Agps	2164	2315	2294	2015	2573	2744	2410	2241
Agr2	10504	11296	11609	11643	12700	11682	11266	11841
Agr3	17	25	16	24	23	38	18	18
Agrn	8485	9598	9171	8394	8506	8367	8320	7517
Agrp	0	0	6	2	1	0	0	0
Agt	137	178	178	155	183	147	127	99
Agtpbp1	137	167	133	197	111	121	173	150
Agtr1a	41	33	45	51	15	28	24	50
Agtr1b	1	6	2	6	10	2	7	3
Agtrap	849	756	815	898	778	685	669	691
Agxt	22	16	6	5	14	21	22	21
Agxt2	9	9	7	20	2	9	8	6
Ahctf1	1569	1935	1653	1591	2037	2126	1852	1770
Ahcy	4777	5281	5294	4966	5755	5480	4927	5533
Ahcyl1	13386	14262	13429	13397	14023	14494	14851	14167
Ahcyl2	27833	30379	27914	28033	29144	30064	28774	27185
Ahdc1	765	847	870	788	827	854	769	777
Ahi1	58	69	75	52	57	63	64	69
Ahnak	6616	7232	7600	7573	7921	8675	9319	8214
Ahnak2	33	26	19	56	40	23	41	42
Ahr	1141	1090	1078	1357	1356	1289	1175	1136
Ahrr	71	65	57	94	136	200	126	133
Ahsa1	2343	2450	2562	2854	3366	3126	2669	2703
Ahsa2	569	657	728	755	995	926	832	740
Ahsg	82	114	75	64	51	76	69	50
Aicda	0	0	0	0	1	0	0	0
Aida	872	928	811	990	828	901	848	759
Aif1	193	160	187	196	195	173	193	220
Aif1l	20	13	12	22	24	13	10	24
Aifm1	3621	3664	3661	3618	3577	3423	3397	3469
Aifm2	493	543	529	622	466	488	523	382
Aifm3	55	23	22	41	16	38	25	24
Aig1	1518	1407	1387	1366	1428	1369	1253	1358
Aim2	101	125	106	110	165	158	117	103
Aimp1	2060	2143	1995	2030	2324	2057	2030	2103
Aimp2	978	989	916	871	1010	987	863	863
Aip	1245	1410	1239	1220	1305	1401	1268	1265
Aipl1	0	0	2	0	0	0	0	0
Aire	4	6	4	1	1	0	0	0
Ajap1	9	8	1	7	4	3	3	4
Ajm1	11	4	11	3	2	2	4	1
Ajuba	114	108	133	115	108	127	102	113
Ak1	98	78	69	92	121	96	61	101
Ak2	18145	18918	18917	17790	18460	19172	18664	18080
Ak3	14265	15681	15175	15377	14947	15806	15354	14492
Ak4	159	179	187	266	263	242	259	238

Transcriptome sequencing yielded total genetic results for the MOD and APS groups, with a total of 15,936 variables

Continued from above

Ak5	0	0	0	1	0	0	0	0
Ak6	198	248	261	206	305	309	208	216
Ak7	2	18	7	25	6	12	12	7
Ak8	4	0	2	1	2	0	0	1
Ak9	0	0	1	0	0	0	0	0
Akain1	0	0	0	0	0	1	1	1
Akap1	2529	2562	2453	2534	2617	2549	2361	2367
Akap10	1193	1358	1192	1396	1391	1337	1286	1172
Akap11	1644	1664	1466	1708	1552	1620	1677	1605
Akap12	307	371	363	334	277	349	405	278
Akap13	2830	3048	2849	2972	2672	2798	2592	2402
Akap14	1	4	0	4	2	1	0	0
Akap17a	1481	1415	1508	1372	1540	1492	1330	1464
Akap17b	61	62	50	91	96	91	56	83
Akap3	0	0	0	0	2	0	0	1
Akap5	2	1	5	2	3	6	2	0
Akap6	33	65	53	42	54	62	58	51
Akap7	2119	2130	2074	2092	2081	1953	1904	1880
Akap8	1297	1495	1476	1627	1578	1696	1429	1473
Akap8l	391	463	449	481	461	462	331	368
Akap9	3686	4316	4031	3551	4562	4588	4766	3959
Akip1	179	187	211	235	247	209	196	210
Akirin1	3172	3255	3139	3229	3313	3293	3170	3186
Akirin2	1599	1763	1642	1506	1643	1706	1569	1629
Akna	197	210	250	195	229	229	191	173
Aknad1	1	0	1	4	0	0	4	0
Akp3	10669	12067	5291	4735	11433	10518	6886	7426
Akr1a1	25911	26432	25939	26266	25217	26249	25089	24880
Akr1b10	431	488	445	465	575	571	473	519
Akr1b3	951	1013	1110	1003	1187	1272	1081	1058
Akr1b7	4171	5639	4139	3082	6504	8200	7905	6131
Akr1b8	1021	1354	1082	896	2279	2031	1495	2186
Akr1c12	4637	5256	5032	4735	5883	5888	5410	5294
Akr1c13	5442	6517	6211	5022	7812	7424	5994	6263
Akr1c14	337	390	436	340	312	432	386	305
Akr1c18	0	0	0	0	1	0	0	0
Akr1c19	2088	2762	2725	2192	3588	3267	2647	2867
Akr1c21	0	0	0	0	0	1	0	0
Akr1c6	1	2	4	2	11	4	1	2
Akr1cl	7	0	1	2	2	0	1	0
Akr1d1	4	0	0	0	1	1	2	0
Akr1e1	1052	1068	1047	998	1214	1267	1174	1181
Akr7a5	3985	3704	3768	3861	3745	3712	3583	3592
Akt1s1	2243	2284	2417	2300	2390	2222	1971	2282
Akt2	2759	2808	2744	2747	2694	2731	2734	2596
Akt3	371	359	383	324	274	265	223	201
Aktip	1381	1520	1474	1309	1594	1570	1460	1487
Alad	2449	2633	2385	2254	2380	2609	2616	2386
Alas1	8931	9773	8599	8059	9570	10599	9958	8774
Alas2	2	0	2	0	0	0	0	2
Alb	551	797	614	495	509	775	581	381
Albfm1	0	0	0	2	0	0	0	1
Alcam	1261	1326	1266	1262	1445	1326	1122	1041
Aldh16a1	7177	7065	6794	6405	6704	6984	6560	6319
Aldh18a1	5576	6035	5957	5545	5906	5704	4905	4516
Aldh1a1	6671	8906	6293	4851	10630	10675	9818	9583
Aldh1a2	379	377	338	301	362	330	348	362
Aldh1a3	1549	1558	1665	1686	1354	1434	1390	1192

Transcriptome sequencing yielded total genetic results for the MOD and APS groups, with a total of 15,936 variables

Continued from above

Aldh1a7	674	1017	822	500	1107	1114	956	915
Aldh1b1	17071	18930	19227	17692	17419	17531	15314	15118
Aldh1l1	9513	9732	9286	8979	8810	8978	9719	9544
Aldh1l2	2	24	18	14	20	21	62	34
Aldh2	9901	10772	10421	9956	10150	11144	10510	10226
Aldh3a1	1	0	0	0	1	1	0	0
Aldh3a2	7512	8299	7335	6804	8749	9171	8507	8009
Aldh3b1	2988	3009	2764	2710	2665	2923	2918	2572
Aldh3b2	37	18	27	14	26	29	33	37
Aldh3b3	1	4	6	1	15	16	8	12
Aldh4a1	6746	7338	5587	5281	6699	7762	9393	8460
Aldh5a1	1224	1312	1223	1283	1129	1229	1172	1083
Aldh6a1	790	825	850	892	806	900	873	841
Aldh7a1	312	305	292	280	399	373	290	342
Aldh8a1	2	1	1	11	2	2	5	4
Aldh9a1	17121	18316	16925	16369	17858	19242	18569	17809
Aldoa	23705	22971	25038	25620	23950	23139	21608	23352
Aldoart1	5	1	0	2	1	0	1	2
Aldoart2	2	4	5	2	0	4	9	0
Aldob	102735	114946	103886	97643	116739	127335	138682	125606
Aldoc	472	664	579	496	827	694	654	636
Alg1	583	503	572	592	624	614	489	520
Alg10b	1012	1133	1037	1062	1059	1171	1051	1085
Alg11	941	1111	1015	991	1158	1088	884	944
Alg12	623	723	703	663	680	645	660	647
Alg13	103	97	82	92	51	99	101	56
Alg14	402	417	463	406	439	471	407	432
Alg2	764	799	807	864	875	779	749	843
Alg3	437	476	371	389	432	391	335	404
Alg5	1421	1493	1585	1407	1576	1626	1609	1502
Alg6	834	840	930	878	1107	971	1019	929
Alg8	527	521	603	415	596	513	456	520
Alg9	1202	1400	1379	1230	1480	1361	1326	1284
Alk	0	11	4	1	5	1	5	1
Alkal1	1	0	1	6	0	1	0	0
Alkal2	0	0	0	0	0	0	0	1
Alkbh1	164	152	161	167	202	170	155	155
Alkbh2	74	109	112	86	149	142	92	108
Alkbh3	934	887	960	964	795	690	696	742
Alkbh4	348	413	337	380	440	434	333	347
Alkbh5	4555	4998	4706	4906	5054	5014	4913	4571
Alkbh6	640	613	600	582	484	535	570	607
Alkbh7	601	583	599	629	611	546	550	574
Alkbh8	312	338	303	297	373	365	412	333
Alms1	135	191	156	206	190	240	150	119
Alox12	25	22	34	25	34	18	14	40
Alox12b	0	0	0	1	0	0	0	0
Alox12e	10	8	8	9	4	11	20	1
Alox15	55	78	68	77	77	62	39	43
Alox5	15	32	34	31	37	37	52	52
Alox5ap	27	31	30	41	32	42	28	19
Aloxe3	2	0	0	1	5	1	0	4
Alpi	72966	81738	73197	67827	72690	72540	65521	65140
Alpk1	1616	1711	1762	1876	1847	1900	2074	1933
Alpk2	0	4	0	1	3	0	1	0
Alpk3	18	1	14	3	2	12	9	5
Alpl	66	100	103	94	89	102	59	58
Alppl2	100	108	140	127	159	139	129	113

Transcriptome sequencing yielded total genetic results for the MOD and APS groups, with a total of 15,936 variables

Continued from above

Als2	2787	3331	2772	2810	2916	3090	3153	2686
Als2cl	613	626	648	559	615	544	583	597
Alx3	0	0	0	0	1	0	0	0
Alx4	0	1	1	0	1	1	1	1
Alyref	1550	1868	1668	1664	1785	1662	1479	1568
Alyref2	156	159	110	177	143	190	144	155
Amacr	952	1055	952	948	918	920	953	938
Ambp	60	82	87	102	76	55	87	63
Ambra1	1602	1886	1872	1621	1654	1627	1719	1645
Amd1	1395	1563	1378	1326	1465	1374	1274	1359
Amd2	7	15	8	15	15	14	6	20
Amdhd1	0	0	1	1	2	4	1	2
Amdhd2	2202	2087	2021	2018	2122	2001	2058	1886
Amer1	283	323	239	246	267	298	268	173
Amer2	1	6	0	0	2	1	0	1
Amer3	1	1	1	1	1	0	0	0
Amfr	5544	6013	5573	5742	5949	5763	5973	5281
Amh	12	5	1	3	0	0	0	0
Amhr2	7	4	1	0	18	10	6	1
Amigo1	104	100	80	81	103	90	112	96
Amigo2	139	121	128	110	123	143	122	128
Amigo3	551	509	485	458	398	485	504	441
Ammecr1	479	500	440	436	563	466	437	445
Ammecr1l	2377	2714	2474	2343	2853	2637	2653	2745
Amn	3467	3176	3254	2803	3203	3195	3304	3277
Amn1	256	274	250	233	293	261	228	271
Amot	898	1139	1058	934	938	970	831	819
Amotl1	253	280	352	295	337	320	275	300
Amotl2	484	510	490	466	514	502	468	427
Ampd1	0	0	0	2	0	1	5	0
Ampd2	1178	1297	1093	1122	1305	1297	1222	1310
Ampd3	776	778	761	804	779	834	832	753
Amph	12	20	20	15	5	7	12	11
Amt	36	50	21	34	28	29	15	38
Amy1	5	15	16	3	31	43	46	26
Amy2a1	2988	5029	2271	789	18445	17692	13741	16607
Amy2a2	1	3	1	0	37	3	3	9
Amy2a3	19	43	12	8	108	100	85	115
Amy2a4	18	5	18	1	44	30	42	27
Amy2a5	12	29	21	17	215	225	180	195
Amy2b	25	20	24	3	122	111	92	140
Amz1	94	69	71	86	71	80	63	115
Amz2	696	737	814	842	886	777	765	793
Anapc1	3831	4267	3976	3922	4163	3948	3671	3794
Anapc10	195	171	157	183	239	201	164	192
Anapc11	794	790	725	751	877	905	813	872
Anapc13	1043	1166	1053	1174	1025	1106	852	976
Anapc15	338	396	417	480	426	444	350	359
Anapc16	1144	1157	1177	1199	1216	1185	1069	1108
Anapc2	2492	2608	2507	2668	2088	2089	2146	2278
Anapc4	910	1000	802	827	837	912	860	814
Anapc5	2772	2947	2883	2675	2846	2653	2504	2666
Anapc7	1108	1158	1056	1153	1122	1172	960	1090
Ang	401	395	363	382	413	388	395	367
Ang4	2913	2385	2327	3123	1884	1983	2604	2579
Angel1	52	120	59	61	83	35	57	109
Angel2	2147	2412	2273	2229	2313	2548	2249	2010
Angpt1	7	10	17	14	23	8	10	26

Transcriptome sequencing yielded total genetic results for the MOD and APS groups, with a total of 15,936 variables

Continued from above

Angpt2	85	138	95	118	79	97	61	78
Angpt4	0	1	1	1	2	0	3	0
Angptl1	44	44	69	86	68	76	59	72
Angptl2	214	292	350	288	295	296	270	245
Angptl3	7	1	11	0	1	2	0	1
Angptl4	590	912	1240	739	1099	1329	1485	1565
Angptl6	53	37	22	45	64	54	46	41
Angptl7	0	1	2	1	0	0	1	0
Angptl8	194	168	161	166	178	117	156	161
Ank	2065	2596	2225	2042	2570	2517	2382	2169
Ank1	45	45	67	38	51	36	66	68
Ank2	93	112	96	63	90	77	91	65
Ank3	1771	1943	1948	1931	1522	1638	1812	1677
Ankdd1b	2	1	10	0	0	1	1	0
Ankef1	1	1	0	1	0	0	0	0
Ankfn1	0	0	0	2	0	0	0	1
Ankfy1	5460	6348	6130	5434	6346	6144	5691	5479
Ankhd1	2561	2898	2628	2704	2908	2982	2762	2497
Ankib1	2752	3101	2825	2820	3126	3184	3009	2659
Ankk1	0	0	0	0	0	0	0	1
Ankle1	91	140	108	90	156	116	108	151
Ankle2	933	960	873	755	997	839	833	738
Ankmy1	7	0	10	0	1	3	1	1
Ankmy2	1372	1594	1446	1414	1512	1691	1525	1465
Ankra2	584	633	680	587	606	668	644	648
Ankrd1	0	0	0	1	2	0	0	0
Ankrd10	1125	1320	1380	1299	1318	1409	1226	1140
Ankrd11	5665	6074	5764	5865	5723	5831	5677	5397
Ankrd12	450	414	409	464	465	497	528	452
Ankrd13a	11807	12204	11885	11673	11692	11840	11479	10985
Ankrd13b	242	263	231	185	205	204	211	183
Ankrd13c	2411	2771	2496	2407	2892	2893	2802	2675
Ankrd13d	8	18	19	11	15	25	17	13
Ankrd16	489	493	457	477	457	430	408	398
Ankrd17	4167	4867	4499	4462	4436	4614	4651	3928
Ankrd2	0	0	1	0	0	1	1	4
Ankrd22	20	21	35	29	31	13	16	21
Ankrd23	45	70	82	92	69	51	32	43
Ankrd24	45	47	37	77	87	54	66	57
Ankrd26	124	112	141	133	145	161	182	142
Ankrd27	731	817	667	815	708	655	683	719
Ankrd28	1943	2203	1886	1923	1968	2004	2034	1931
Ankrd29	25	11	21	10	9	7	24	25
Ankrd31	0	1	1	2	5	0	0	6
Ankrd33b	20	4	14	18	20	14	15	8
Ankrd34a	14	0	3	0	12	4	5	1
Ankrd34b	0	0	4	0	0	0	2	0
Ankrd34c	0	0	0	0	0	0	0	1
Ankrd35	14	8	19	11	21	22	19	5
Ankrd37	283	212	279	340	396	421	330	316
Ankrd39	100	128	116	79	74	79	94	66
Ankrd40	2907	3114	2695	2823	2877	2926	2688	2678
Ankrd42	9	20	40	44	27	45	17	35
Ankrd44	238	200	237	206	200	248	188	274
Ankrd45	0	0	0	1	1	0	0	2
Ankrd46	2184	2229	2240	2184	2056	2051	2248	2218
Ankrd49	397	420	387	383	507	455	415	380
Ankrd50	2195	2284	2215	2185	2088	1992	2067	2063

Transcriptome sequencing yielded total genetic results for the MOD and APS groups, with a total of 15,936 variables

Continued from above

Ankrd52	2244	2254	2373	2345	2254	2245	2193	2014
Ankrd53	2	0	0	0	0	2	0	0
Ankrd54	1389	1496	1445	1368	1563	1513	1315	1268
Ankrd55	11	8	5	7	4	7	1	2
Ankrd6	45	16	37	16	34	33	20	28
Ankrd61	5	0	5	2	0	0	1	2
Ankrd63	28	17	14	30	39	11	20	21
Ankrd66	6	0	1	5	3	1	0	6
Ankrd7	1	9	1	1	8	0	6	1
Ankrd9	2010	1928	1910	1912	1965	2174	1988	1987
Anks1	1768	1793	1673	1662	1922	1992	1666	1728
Anks1b	8	0	16	6	5	1	7	5
Anks3	586	741	732	625	727	655	613	573
Anks4b	5411	6176	5640	5533	6317	7063	7465	6360
Anks6	69	50	43	50	51	81	64	63
Ankzf1	786	927	899	916	871	915	856	913
Anln	625	692	612	672	744	638	624	662
Ano1	222	167	254	237	247	222	230	248
Ano10	1054	1011	1132	941	1211	1175	1020	1029
Ano2	28	45	44	35	43	48	41	54
Ano3	0	1	2	1	10	5	1	6
Ano5	25	6	20	13	24	14	9	15
Ano6	8756	9868	8867	8217	8890	9300	10373	9482
Ano7	1022	1175	1300	1210	1112	1080	1010	1038
Ano8	303	280	304	353	271	249	231	236
Ano9	3551	3973	3702	3563	3612	3763	3416	3486
Anp32a	2169	2136	2301	2220	2081	2057	2107	2159
Anp32b	3187	3594	3279	3192	3541	3255	2812	2919
Anp32e	1882	2155	2002	1925	2164	2081	1855	2012
Anpep	245872	275893	235062	217875	253925	266213	276018	249264
Antkmt	1237	1221	1375	1273	1167	1347	1256	1117
Antxr1	383	429	380	359	382	407	361	329
Antxr2	1738	1936	1800	1691	1934	1995	1927	1773
Anxa1	146	232	217	291	271	360	290	274
Anxa10	437	476	429	358	746	508	355	469
Anxa11	6697	6727	6690	6588	6550	6769	6371	6470
Anxa13	8246	10263	8897	7535	10553	10609	9402	9920
Anxa2	28844	31243	28561	28183	30344	31318	29492	29633
Anxa3	296	335	304	356	411	345	371	424
Anxa4	21851	23988	22252	22619	21844	22687	22451	21909
Anxa5	908	958	972	1081	1075	961	910	906
Anxa6	1087	999	986	1068	1045	1027	1112	863
Anxa7	3979	3897	4271	4229	3938	3933	3859	4039
Anxa8	21	0	3	14	9	4	13	22
Anxa9	24	30	39	30	31	28	40	47
Aoah	137	62	108	114	75	110	78	61
Aoc1	21733	24932	22276	20080	22580	23261	23642	21606
Aoc2	194	184	207	156	224	190	255	257
Aoc3	230	285	293	321	360	329	255	283
Aopep	848	774	822	831	796	845	696	733
Aox1	12	19	15	10	22	14	8	9
Aox2	8	1	1	8	2	5	1	0
Aox3	0	2	5	8	5	3	11	5
Aox4	0	0	0	3	0	0	0	0
Ap1ar	4246	4313	4328	4143	3985	4247	4037	3896
Ap1b1	6790	6769	6531	6679	6815	6879	6830	6504
Ap1g1	5013	5519	4905	4987	5283	5426	5402	4868
Ap1g2	3818	3986	3764	3783	3723	3925	3648	3575

Transcriptome sequencing yielded total genetic results for the MOD and APS groups, with a total of 15,936 variables

Continued from above

Ap1m1	1449	1535	1605	1545	1373	1363	1353	1363
Ap1m2	4436	4730	4729	4469	4515	4572	4121	4277
Ap1s1	5720	5628	5348	5627	5670	5692	5492	5329
Ap1s2	85	126	121	104	104	93	111	106
Ap1s3	910	1128	817	806	1086	1139	1094	1075
Ap2a1	3957	3735	3793	3747	3551	3403	3465	3514
Ap2a2	9504	11070	10053	9607	10291	10193	9957	9126
Ap2b1	6478	7379	6988	6244	7365	7011	6925	6602
Ap2m1	12083	11792	11970	11433	11400	12232	11255	11121
Ap2s1	3711	3927	4013	3741	3580	3471	3472	3311
Ap3b1	4044	4441	4112	3863	4607	4817	4545	4104
Ap3b2	11	36	17	28	16	13	14	15
Ap3d1	4923	5500	4914	5047	5242	5122	4957	5041
Ap3m1	2800	2951	2678	2808	3108	3158	2976	2654
Ap3m2	102	115	98	88	87	78	85	109
Ap3s1	2745	3075	2944	2505	2862	2872	2822	2723
Ap3s2	1427	1689	1547	1451	1533	1581	1383	1455
Ap4b1	753	915	879	800	929	815	794	871
Ap4e1	624	602	617	529	569	596	625	490
Ap4m1	670	773	694	755	690	758	726	618
Ap4s1	366	420	383	424	395	478	409	446
Ap5b1	1153	1275	1264	1131	1300	1408	1322	1247
Ap5m1	870	856	803	859	858	816	776	818
Ap5s1	1205	1240	1176	1069	1082	1210	1268	1220
Ap5z1	749	688	653	646	774	683	676	659
Apaf1	6301	6736	5888	5887	6331	6395	6441	5925
Apba1	63	107	77	76	57	73	88	80
Apba2	12	11	20	31	20	4	23	11
Apba3	3160	3252	3132	3039	3267	3507	3444	3371
Apbb1	67	109	93	110	74	82	100	72
Apbb1ip	100	83	96	91	82	146	80	119
Apbb2	643	660	668	619	598	507	625	499
Apbb3	382	314	382	396	354	414	384	343
Apc	2775	3006	2872	3066	3057	3276	3164	2847
Apc2	44	15	18	37	22	28	26	22
Apcdd1	100	111	106	94	90	97	72	55
Apcs	1	2	4	1	1	5	2	0
Apeh	5909	6282	6113	5803	6126	6264	5791	5662
Apela	4	0	0	0	8	0	0	0
Apex1	1089	1293	1145	1084	1130	1137	1009	1068
Apex2	161	161	142	120	168	214	166	191
Aph1a	4830	4751	4388	4330	4645	4475	4212	4041
Aph1b	79	92	86	103	116	69	151	103
Aph1c	27	38	24	21	36	30	12	14
Api5	4243	4460	4419	4257	4703	4764	4348	4068
Apip	159	125	162	155	142	106	117	102
Aplf	211	210	205	190	196	243	211	219
Apln	237	248	265	219	296	256	202	199
Aplnr	113	108	108	127	113	120	87	85
Aplp1	3469	3770	3237	2944	3314	3346	3803	3137
Aplp2	7714	8342	7847	7563	8542	8349	8291	7933
Apmap	1262	1321	1347	1367	1414	1444	1309	1339
Apoa1	248038	254520	243439	239437	227723	251113	261546	246819
Apoa2	122	100	95	127	147	90	90	94
Apoa4	296860	304833	273469	270679	285113	313166	337584	317278
Apoa5	14	13	9	6	12	12	12	17
Apob	110200	129191	97068	95170	120252	133160	151315	123919
Apobec1	2626	2785	2647	2393	2877	2982	2975	2674

Transcriptome sequencing yielded total genetic results for the MOD and APS groups, with a total of 15,936 variables

Continued from above

Apobec2	280	267	250	180	254	227	198	221
Apobec3	2400	2498	2487	2389	2125	2150	2017	2095
Apobec4	0	0	1	0	0	0	0	0
Apobr	88	65	68	80	51	53	67	78
Apoc1	14	29	11	23	29	42	11	12
Apoc2	52	32	64	45	45	35	40	42
Apoc3	5756	5988	5553	5342	5006	5637	7089	6443
Apoc4	6	1	4	6	2	1	10	2
Apod	58	50	50	53	60	37	54	41
Apoe	13160	11664	11706	13571	11610	11468	10312	11415
Apof	14	13	11	10	9	21	16	7
Apoh	38	39	64	28	46	38	44	30
Apol10a	9750	10266	8903	9239	7561	8319	7480	6170
Apol10b	231	199	218	172	192	181	160	135
Apol11a	1	2	1	1	2	0	6	3
Apol11b	0	1	3	0	1	0	1	0
Apol6	91	83	58	81	35	41	51	25
Apol7a	4303	4917	4382	4027	4630	5198	5405	5054
Apol7b	8	8	2	13	7	6	2	7
Apol7c	871	861	754	795	930	1059	970	990
Apol7e	62	83	91	103	62	71	67	58
Apol8	6	4	2	2	0	2	6	7
Apol9a	3809	4878	4355	3128	4176	4576	4352	4345
Apol9b	1150	1540	1773	1155	1716	1672	1494	1478
Apold1	73	58	67	46	45	58	59	34
Apom	4	9	0	2	6	0	5	8
Apon	2	5	1	0	1	2	5	3
Apoo	1664	1733	1484	1675	1415	1591	1603	1461
Apool	1239	1217	1246	1310	1247	1297	1262	1234
App	15638	16418	15294	14726	15266	16013	15068	14965
Appbp2	598	702	710	666	785	825	696	732
Appl1	755	687	673	782	659	704	811	681
Appl2	1252	1327	1154	1123	1257	1348	1093	1170
Aprt	6987	7220	7024	6981	7288	7267	6790	6797
Aptx	384	492	415	384	393	371	386	410
Aqp1	2629	2982	3040	2935	2433	2836	2850	2624
Aqp11	2577	2458	2427	2520	2514	2713	2543	2479
Aqp12	14	22	0	1	62	74	25	37
Aqp3	2003	2022	1748	1589	1812	1843	1973	1782
Aqp4	643	873	870	684	783	855	807	662
Aqp5	0	4	0	0	0	0	0	1
Aqp7	1251	964	985	1343	660	705	819	797
Aqp8	227	250	206	127	321	330	226	128
Aqp9	8	8	2	5	6	9	7	2
Aqr	1673	2007	1782	1694	2119	1859	1765	1665
Ar	6	8	3	13	15	8	13	9
Araf	2357	2420	2437	2147	2370	2183	2330	2156
Arap1	2777	2826	2770	2638	2382	2483	2443	2459
Arap2	1858	2212	1697	1957	1933	2175	2224	1946
Arap3	277	273	243	266	264	315	203	179
Arc	1	8	6	12	5	11	2	8
Arcn1	15699	17011	15281	15010	17320	18120	17818	16224
Areg	874	935	985	838	689	557	589	668
Arel1	2758	3261	2785	2458	2724	2637	2650	2274
Arf1	31744	33037	31700	31097	32948	32982	32172	32262
Arf2	649	747	632	672	669	682	700	739
Arf3	6648	6760	6224	6230	5549	5994	5641	5765
Arf4	16458	18439	16642	16569	17764	17472	17120	16219

Transcriptome sequencing yielded total genetic results for the MOD and APS groups, with a total of 15,936 variables

Continued from above

Arf5	8193	8160	7637	7520	7302	7453	7342	7390
Arf6	14303	14917	13067	12948	13705	14199	15124	14096
Arfgap1	2195	2138	2069	1975	1936	2158	1997	1987
Arfgap2	2583	2747	2706	2668	2611	2867	2609	2578
Arfgap3	6157	6686	6117	6130	6390	6681	6883	6250
Arfgef1	7595	8376	7369	7506	8672	8972	8355	7643
Arfgef2	4651	4682	4453	4765	4309	4469	4549	4322
Arfgef3	214	226	196	212	216	302	251	210
Arfip1	4607	4892	4519	4291	5457	5362	4775	4720
Arfip2	2225	2210	2156	2098	2400	2440	2310	2211
Arfrp1	793	773	883	761	829	873	749	766
Arg1	34	47	28	25	36	26	25	34
Arg2	16077	17996	17585	17217	15717	17426	15362	14199
Arglu1	1141	1232	1402	1275	1229	1181	1218	1165
Arhgap1	2830	3065	2909	2755	3399	3128	2818	3030
Arhgap10	130	134	153	145	179	183	136	177
Arhgap11a	1045	1228	1102	1075	1317	1212	1117	1081
Arhgap12	2178	2399	2225	2227	2501	2694	2680	2304
Arhgap15	94	43	39	48	41	45	23	31
Arhgap17	2856	2982	2791	2616	2590	2568	2588	2405
Arhgap18	2650	2746	2513	2739	2530	2879	2671	2517
Arhgap19	665	770	800	742	779	774	698	758
Arhgap20	41	41	48	43	37	33	33	19
Arhgap21	3500	3604	3527	3700	3257	3289	3152	3126
Arhgap22	24	20	23	31	10	23	28	24
Arhgap23	235	235	243	271	237	162	173	165
Arhgap24	74	92	111	84	105	79	71	85
Arhgap25	83	87	102	81	53	69	44	61
Arhgap26	2403	2409	2227	2220	2191	2495	2297	2170
Arhgap27	3898	3750	3929	3933	4196	4192	4089	4037
Arhgap28	67	126	60	79	82	59	70	58
Arhgap29	481	550	457	499	652	555	525	605
Arhgap30	314	350	258	302	330	312	210	235
Arhgap31	464	745	618	528	740	742	862	742
Arhgap32	6440	7154	6732	6566	6397	6488	6271	5680
Arhgap33	65	84	105	68	59	94	76	71
Arhgap35	1028	1126	998	1194	1065	1132	1040	979
Arhgap39	352	431	360	365	394	457	383	356
Arhgap4	300	336	268	241	228	261	252	254
Arhgap40	0	1	0	0	2	0	0	0
Arhgap42	2283	2709	2194	2311	2749	2707	2685	2452
Arhgap44	25	30	35	38	48	24	14	29
Arhgap45	331	265	248	298	236	288	278	198
Arhgap5	3291	4200	3693	3626	4174	4621	4485	3797
Arhgap6	86	88	70	122	104	75	111	116
Arhgap8	232	239	269	255	238	207	204	191
Arhgap9	200	302	299	218	288	298	219	266
Arhgdia	18136	18294	17495	17509	17236	17237	16452	16767
Arhgdib	350	268	288	332	276	347	295	317
Arhgdig	45	40	36	23	92	61	64	66
Arhgef1	2140	2328	2392	2138	2224	2192	2004	2154
Arhgef10	116	149	178	177	130	156	129	143
Arhgef10l	583	618	511	395	622	603	542	538
Arhgef11	3273	3181	3258	3103	3148	3214	3278	3034
Arhgef12	2613	2810	2892	2819	2891	2943	2616	2683
Arhgef15	111	83	110	106	90	93	72	56
Arhgef16	7164	7425	6921	6525	6269	6454	6930	6479
Arhgef17	303	308	337	321	250	315	280	250

Transcriptome sequencing yielded total genetic results for the MOD and APS groups, with a total of 15,936 variables

Continued from above

Arhgef18	2002	2044	2018	1908	2070	1912	1956	1845
Arhgef19	1904	1938	1847	1903	1893	1809	1908	1895
Arhgef2	3977	3882	3952	3637	3202	3359	3745	3328
Arhgef25	135	146	158	122	144	148	138	184
Arhgef26	1693	1963	1941	1813	1887	1900	1835	1736
Arhgef28	322	379	359	380	273	348	316	228
Arhgef3	620	683	495	562	552	574	693	641
Arhgef33	2	6	4	1	16	19	14	2
Arhgef37	102	77	73	77	67	88	125	101
Arhgef38	882	837	832	848	923	1023	838	761
Arhgef39	635	645	771	642	758	844	571	670
Arhgef4	15	6	11	17	8	21	11	14
Arhgef40	265	261	276	243	258	238	240	236
Arhgef5	5899	6554	6222	5739	6236	6499	6385	5937
Arhgef6	106	122	113	131	124	140	134	121
Arhgef7	2058	2237	1993	1891	2326	2387	2192	2085
Arhgef9	32	39	32	13	34	41	26	20
Arid1a	3722	4189	4144	3996	4142	4085	3975	3531
Arid1b	1710	1957	1689	1616	1724	1786	1698	1661
Arid2	1649	1730	1657	1566	1819	1740	1700	1587
Arid3a	286	348	271	233	349	337	288	236
Arid3b	300	310	256	297	287	287	296	239
Arid4a	707	726	792	755	782	900	851	844
Arid4b	873	1023	796	1056	1062	1153	1090	1015
Arid5a	235	193	193	162	119	171	139	145
Arid5b	546	616	655	653	536	592	477	502
Arih1	3335	3327	3309	3203	3848	3839	3649	3465
Arih2	1916	2115	1962	2116	1991	1993	1811	1860
Arl1	4273	4651	4021	4109	4607	4607	4576	4152
Arl10	384	353	417	334	318	354	318	235
Arl11	13	58	55	56	45	38	55	48
Arl13a	0	0	0	0	0	0	1	0
Arl13b	310	266	255	268	308	398	380	356
Arl14	564	629	504	468	668	681	660	747
Arl14ep	207	259	248	226	240	266	256	213
Arl15	731	805	749	759	895	871	940	885
Arl16	272	211	244	215	209	231	233	243
Arl2	507	480	543	452	520	506	525	439
Arl2bp	1694	1694	1856	1920	1389	1387	1554	1396
Arl3	125	86	121	79	99	115	107	126
Arl4a	1509	1729	1637	1651	1793	1880	1645	1610
Arl4c	196	221	209	186	195	174	207	164
Arl4d	679	592	700	749	484	539	664	624
Arl5a	4918	5399	4822	4894	5416	5577	5846	5099
Arl5b	705	935	859	825	822	822	883	817
Arl5c	13	37	12	33	13	14	23	20
Arl6	24	40	60	54	62	52	50	34
Arl6ip1	10513	12017	11539	11132	11843	12202	11013	11234
Arl6ip4	1071	1043	957	1097	1095	1057	938	977
Arl6ip5	1670	1641	1721	1816	1567	1687	1579	1492
Arl6ip6	804	814	733	783	793	748	664	741
Arl8a	2259	2199	2158	2150	1956	2008	2016	2137
Arl8b	3042	3260	2996	3013	3262	3174	3028	2961
Arl9	7	1	12	2	1	2	2	2
Armc1	1630	1757	1656	1731	1902	1780	1811	1702
Armc10	742	946	791	760	754	783	759	762
Armc2	11	20	9	18	32	39	22	21
Armc3	0	10	1	0	4	0	0	0

Transcriptome sequencing yielded total genetic results for the MOD and APS groups, with a total of 15,936 variables

Continued from above

Armc4	1	0	0	0	0	0	0	0
Armc5	1293	1302	1211	1155	1272	1128	1168	1117
Armc6	400	339	400	432	465	456	416	369
Armc7	355	331	319	322	376	293	323	303
Armc8	2131	2237	1918	1882	1932	2042	2002	1822
Armc9	70	112	94	100	92	107	107	85
Armcx1	37	35	39	20	61	28	50	54
Armcx2	83	118	75	118	117	81	73	64
Armcx3	88	109	120	112	105	151	87	115
Armcx4	28	31	11	44	19	32	39	32
Armcx5	67	72	50	87	58	45	75	58
Armcx6	0	3	12	17	7	15	4	6
Armh1	1	0	1	0	0	0	1	0
Armh3	938	967	1014	977	946	921	791	831
Armh4	41	64	31	20	41	35	29	35
Armt1	726	748	622	588	719	688	664	632
Arnt	1795	1964	1820	1773	1842	2001	1909	1836
Arnt2	116	132	109	84	166	101	153	146
Arntl	1092	1406	1259	1094	1401	1360	1266	1187
Arntl2	44	30	28	33	38	69	36	32
Arpc1a	4402	4369	4282	4409	4285	4299	4078	4116
Arpc1b	11260	12640	11303	10924	11667	11968	11426	11025
Arpc2	10871	10954	10895	10278	10521	10886	10392	10174
Arpc3	6352	6523	6336	6546	6575	6209	6130	6131
Arpc4	5299	5517	5123	5206	5132	5126	5008	5156
Arpc5	11390	12418	11656	11251	11765	12153	12249	11559
Arpc5l	1062	1247	1203	1069	1140	1101	997	1041
Arpin	3777	4286	3994	3765	3972	4015	3706	3359
Arpp19	837	939	850	970	750	933	707	776
Arpp21	1	3	0	4	2	0	4	0
Arrb1	4874	5201	5345	5092	5139	5006	4613	4656
Arrb2	315	305	331	361	291	267	246	227
Arrdc1	1379	1504	1491	1430	1399	1382	1481	1330
Arrdc2	251	288	258	278	287	296	253	300
Arrdc3	904	1055	761	793	893	947	906	797
Arrdc4	1431	1756	1630	1541	1666	1683	1510	1244
Arrdc5	0	0	0	0	0	0	0	1
Arsa	1510	1582	1458	1553	1467	1483	1762	1635
Arsb	422	519	481	510	599	494	398	421
Arsg	473	554	470	512	481	496	636	556
Arsi	40	24	42	35	64	51	30	47
Arsj	5	7	13	1	7	13	7	8
Arsk	203	174	159	181	199	228	162	136
Art1	1	0	0	0	0	0	1	0
Art2b	93	71	79	84	63	91	85	50
Art3	141	126	126	169	144	153	156	98
Art4	7	5	17	20	5	2	6	1
Art5	14	4	11	2	7	12	2	5
Artn	16	7	17	8	14	12	18	13
Arv1	351	392	389	349	320	255	320	266
Arvcf	196	202	206	223	232	211	195	236
Arx	29	27	48	29	19	18	27	26
Arxes1	1	0	2	2	4	3	4	0
Arxes2	3	29	4	12	6	6	3	4
As3mt	1456	1526	1427	1331	1385	1440	1301	1374
Asah1	3544	3857	3795	3524	4017	3799	3721	3753
Asah2	12761	15050	12442	11621	13514	15359	16975	13782
Asap1	697	710	524	534	714	671	765	741

Transcriptome sequencing yielded total genetic results for the MOD and APS groups, with a total of 15,936 variables

Continued from above

Asap2	2430	2620	2371	2561	2309	2234	2198	1983
Asap3	64	78	76	79	62	67	52	50
Asb1	341	284	239	285	267	328	302	239
Asb10	0	0	1	1	1	0	0	1
Asb11	21	9	8	16	6	5	6	5
Asb13	1091	1130	1113	1078	1049	1037	986	969
Asb14	0	0	0	0	0	1	1	6
Asb15	0	0	4	0	0	0	0	0
Asb16	12	6	15	29	22	16	9	21
Asb2	750	740	743	687	908	805	789	879
Asb3	211	201	233	270	262	267	299	278
Asb4	2	7	6	10	12	7	9	4
Asb5	1	1	0	0	5	0	1	1
Asb6	677	662	629	613	614	693	574	567
Asb7	537	535	573	593	484	496	526	501
Asb8	1351	1458	1347	1271	1313	1331	1216	1386
Ascc1	682	659	676	612	619	587	628	632
Ascc2	1923	2056	1914	1989	2077	1917	1901	1844
Ascc3	2322	2598	2385	2182	2413	2562	2783	2458
Ascl1	0	0	0	0	1	2	0	4
Ascl2	218	159	157	177	182	139	187	139
Ascl3	9	1	4	10	4	8	1	4
Ascl4	0	0	1	0	0	0	0	0
Asf1a	347	437	385	368	378	452	413	308
Asf1b	474	505	479	466	605	484	483	402
Asgr1	6	9	1	2	9	9	3	1
Asgr2	7	8	0	1	7	7	6	2
Ash1l	1995	2252	2216	2238	2088	2088	2181	1994
Ash2l	1521	1582	1624	1529	1570	1602	1566	1405
Asic1	39	51	39	53	41	46	18	35
Asic2	6	29	21	17	18	10	21	12
Asic3	26	15	11	12	4	9	7	18
Asic4	5	0	0	1	8	0	2	1
Asic5	41	33	31	32	31	45	31	13
Asl	1088	1178	1057	1011	1095	1100	1029	926
Asmt	0	0	0	0	0	0	0	1
Asns	2044	2120	1850	2073	1984	1957	1929	1999
Asnsd1	768	891	853	793	967	867	792	780
Aspa	2357	2608	2029	2012	2475	2771	3055	2758
Aspdh	8	14	8	5	9	17	17	5
Aspg	91	133	129	141	101	95	119	99
Asph	1486	1571	1502	1463	1548	1404	1507	1420
Asphd1	0	4	1	0	6	5	1	1
Asphd2	29	17	21	35	28	23	16	20
Aspm	362	469	408	410	403	440	410	392
Aspn	262	254	287	240	264	317	291	222
Asprv1	45	14	45	31	23	28	49	36
Aspscr1	2398	2659	2451	2429	2495	2662	2446	2332
Asrgl1	90	84	75	60	87	53	54	72
Ass1	316	302	379	378	430	309	229	258
Aste1	383	302	339	333	363	322	322	325
Astn1	21	19	21	31	5	32	17	12
Astn2	6	23	11	10	9	4	21	7
Asxl1	1219	1353	1305	1304	1320	1457	1250	1136
Asxl2	1443	1523	1626	1655	1509	1447	1560	1304
Asxl3	0	2	2	2	2	3	1	2
Atad1	3940	4183	3677	4134	4190	4367	4181	4070
Atad2	718	792	756	756	895	944	712	738

Transcriptome sequencing yielded total genetic results for the MOD and APS groups, with a total of 15,936 variables

Continued from above

Atad2b	1479	1458	1410	1545	1664	1781	1644	1509
Atad3a	2269	2454	2265	2276	2269	2134	2046	1943
Atad5	247	302	189	234	250	211	259	260
Atat1	265	362	371	383	387	372	351	331
Atcay	10	22	13	18	13	13	11	18
Ate1	1177	1135	1174	1217	1219	1371	1242	1106
Atf1	981	1287	1138	1085	1170	1180	1115	1080
Atf1-ps	1	0	1	1	0	6	5	3
Atf2	1410	1616	1333	1487	1406	1390	1531	1441
Atf3	1890	1972	1989	1851	1798	2044	1928	1658
Atf4	8282	8707	8868	8283	8405	8682	8492	8337
Atf5	853	885	852	835	776	735	625	714
Atf6	2147	2429	2136	2403	2503	2618	2506	2392
Atf6b	1803	1893	1741	1730	2084	1998	1851	1688
Atf7	904	1021	1037	1009	997	1055	978	859
Atf7ip	2163	2393	2392	2310	2298	2145	2460	2075
Atf7ip2	33	30	7	37	27	13	7	11
Atg10	117	139	101	153	89	138	150	129
Atg101	794	767	844	781	848	864	771	844
Atg12	2013	2040	2061	1750	2180	2226	2196	1964
Atg13	1773	2048	1894	1808	2053	2013	1993	1885
Atg14	532	526	555	471	527	621	531	577
Atg16l1	1119	1084	1081	1072	1115	1145	1047	1090
Atg16l2	64	72	76	83	71	105	70	71
Atg2a	2402	2507	2389	2219	2250	2243	2366	2245
Atg2b	1883	2255	1936	2061	2239	2257	2377	2031
Atg3	2051	2213	2131	1928	2175	2307	2124	2067
Atg4a	629	606	586	580	749	702	706	683
Atg4b	1808	1791	1734	1645	1786	1793	1591	1685
Atg4c	579	735	617	604	643	715	760	684
Atg4d	4506	4676	4522	4577	4372	4252	4098	4272
Atg5	1936	2144	2131	2024	2315	2371	2241	1985
Atg7	539	569	614	558	548	504	539	596
Atg9a	4146	4686	4394	4205	4451	4269	4496	4264
Atg9b	30	17	18	8	40	28	22	33
Atic	2255	2437	2267	2148	2438	2239	2255	2223
Atl1	13	15	15	10	33	29	56	24
Atl2	7637	8429	7922	7346	10178	10648	11417	9795
Atl3	1662	1882	1823	1685	1924	1873	1815	1671
Atm	662	848	905	696	739	799	720	558
Atmin	920	1038	1042	1081	1198	1112	962	950
Atn1	3216	3203	3045	3188	2870	2821	2984	2761
Atoh1	596	610	809	725	631	694	485	606
Atoh7	1	0	0	0	0	0	0	0
Atoh8	96	67	97	58	67	81	70	69
Atox1	2081	2229	2068	2054	2210	2122	1980	2122
Atp10a	73	130	143	78	88	77	69	76
Atp10b	10410	12893	10441	9480	12339	12483	11655	11910
Atp11a	323	379	384	321	346	390	344	325
Atp11b	6916	7409	7103	6931	7238	7701	8049	7236
Atp11c	708	856	761	820	845	876	828	800
Atp13a1	4375	4354	4172	3853	4342	4131	3846	4037
Atp13a2	853	807	824	795	805	857	653	774
Atp13a3	3052	3559	3421	3181	3785	3902	3510	3169
Atp13a5	0	0	0	0	0	0	0	4
Atp1a1	194960	194812	186546	187881	179617	187977	202208	188587
Atp1a2	140	124	160	173	147	159	138	157
Atp1a3	126	167	138	177	108	128	118	143

Transcriptome sequencing yielded total genetic results for the MOD and APS groups, with a total of 15,936 variables

Continued from above

Atp1a4	1	0	4	1	2	1	0	1
Atp1b1	84148	83384	78470	82443	75278	79508	82267	78770
Atp1b2	85	92	60	91	46	57	47	38
Atp1b3	1141	1321	1186	1169	1292	1211	1244	1211
Atp23	117	131	103	87	149	113	76	106
Atp2a1	1	1	0	0	6	4	6	4
Atp2a2	12903	14038	12838	13158	15576	14664	12978	13288
Atp2a3	4121	4183	4485	4466	4535	4151	3967	4115
Atp2b1	4141	4607	4244	3994	4463	4750	4441	4040
Atp2b2	4	15	7	6	10	6	12	7
Atp2b3	2	1	1	2	10	9	4	7
Atp2b4	641	773	831	811	828	655	740	689
Atp2c1	1655	1807	1825	1762	1974	1987	1877	1646
Atp2c2	442	498	501	534	492	455	553	546
Atp4a	11	4	7	18	8	2	0	1
Atp5a1	92196	91620	91117	91371	89772	92001	87305	87874
Atp5b	108889	112779	109940	109665	108144	110953	105983	103239
Atp5c1	23447	23886	23298	24530	22718	23091	22318	22036
Atp5d	20648	20102	20483	20853	19485	19842	19001	19201
Atp5e	6438	6511	6589	6917	6229	6457	6192	6026
Atp5g1	8872	8599	8878	9302	8247	8205	7637	7601
Atp5g2	4723	4707	4893	4929	4929	4498	4243	4811
Atp5g3	36376	36957	35613	37069	33923	35250	33024	33016
Atp5h	10725	10413	10185	10899	9710	9946	9756	9497
Atp5j	8214	8323	8431	8586	8214	8768	8280	7755
Atp5j2	14107	14785	14415	15358	14207	15467	14441	13572
Atp5k	3200	3458	3423	3729	3384	3625	3282	3148
Atp5l	6901	7131	6983	7262	6789	7215	6735	6599
Atp5md	6083	6051	6186	6514	5885	6341	6030	5861
Atp5mpl	4969	5345	5136	5478	5021	5172	5100	4775
Atp5o	19702	20082	19800	20259	18643	19404	18760	18757
Atp5pb	22688	23837	23966	24201	24435	25621	24107	23345
Atp6ap1	3251	3615	3445	3565	3458	3553	3458	3456
Atp6ap2	5220	5441	5094	5064	5491	5453	5418	5534
Atp6v0a1	3098	3421	3117	3047	3242	3549	3294	3411
Atp6v0a2	14512	15816	14977	14448	16778	16614	15761	15424
Atp6v0a4	0	2	2	3	4	1	4	1
Atp6v0b	4032	3969	3876	4077	3964	4015	3802	3898
Atp6v0c	5320	5260	5248	5344	5258	5097	4914	5281
Atp6v0d1	2954	3233	2804	3083	3007	2985	3132	3185
Atp6v0d2	1	2	1	4	0	11	9	0
Atp6v0e	4700	5075	4753	4963	5182	5232	5047	5091
Atp6v0e2	490	413	486	554	454	537	484	514
Atp6v1a	2803	3109	2968	2866	3403	3432	3249	3085
Atp6v1b1	1	0	0	0	0	0	0	0
Atp6v1b2	5193	5381	5137	4985	4726	4857	5063	4858
Atp6v1c1	1654	1799	1617	1722	1717	1617	1608	1673
Atp6v1c2	0	0	0	4	0	1	4	0
Atp6v1d	2478	2354	2259	2260	2351	2316	2458	2444
Atp6v1e1	3294	3212	3170	3285	3190	3206	3052	3153
Atp6v1f	1705	1692	1698	1666	1556	1709	1560	1561
Atp6v1g1	2429	2686	2604	2709	2618	2631	2369	2555
Atp6v1g2	60	77	77	70	87	52	45	75
Atp6v1h	2480	2803	2606	2626	2875	2802	2613	2723
Atp7a	1908	1979	1865	1930	2214	2049	2217	2012
Atp7b	1727	1687	1752	1985	1621	1529	1509	1453
Atp8a1	2917	3269	2938	3028	2665	2586	2856	2684
Atp8a2	434	372	442	420	418	407	346	353

Transcriptome sequencing yielded total genetic results for the MOD and APS groups, with a total of 15,936 variables

Continued from above

Atp8b1	15299	16422	15229	15585	15269	15621	15734	15006
Atp8b2	220	257	308	240	295	303	274	206
Atp8b3	0	0	0	2	0	0	0	0
Atp8b4	13	1	1	3	2	4	0	5
Atp8b5	1	1	0	0	0	0	0	1
Atp9a	7443	8087	7135	7056	7521	7399	7416	6862
Atp9b	1343	1451	1403	1401	1342	1311	1297	1282
Atpaf1	781	588	643	678	572	721	572	569
Atpaf2	1147	1180	1271	1233	1110	1006	1006	1132
Atpif1	8406	9044	9058	8824	9226	9473	8758	8608
Atpsckmt	323	320	331	327	395	280	387	373
Atr	477	658	520	517	565	541	506	469
Atraid	1106	1229	1197	1227	1174	1254	1120	1081
Atrip	265	234	316	240	261	239	217	258
Atrn	2301	2632	2118	2074	2445	2386	2341	2275
Atrnl1	1631	1856	1662	1780	1766	1690	1696	1519
Atrx	1422	1589	1695	1651	1530	1696	1601	1444
Atxn1	853	829	903	1047	763	736	835	713
Atxn10	493	540	442	503	483	425	394	402
Atxn1l	1326	1427	1370	1315	1325	1395	1341	1235
Atxn2	1657	1614	1625	1670	1558	1567	1589	1537
Atxn2l	3490	3626	3576	3556	3752	3726	3436	3404
Atxn3	579	697	720	644	746	774	603	699
Atxn7	1355	1478	1571	1669	1525	1747	1596	1477
Atxn7l1	644	639	570	561	517	603	529	572
Atxn7l2	84	104	123	95	103	107	101	73
Atxn7l3	3129	3435	3271	3146	3431	3264	3284	3178
Atxn7l3b	4820	5350	4938	4588	5416	5521	5208	4706
Auh	746	785	764	807	638	685	684	642
Aunip	79	101	88	75	100	100	84	106
Aup1	5554	5507	5834	5242	5420	5271	5097	5168
Aurka	747	784	806	824	805	682	700	763
Aurkaip1	2793	2720	2885	2753	2785	2837	2497	2689
Aurkb	680	802	799	810	817	797	604	743
Auts2	503	562	554	589	570	555	449	447
Aven	397	472	502	438	590	504	417	443
Avil	120	116	99	103	122	125	105	81
Avl9	2195	2549	2293	2279	2568	2447	2659	2643
Avpi1	167	181	221	229	222	247	238	241
Avpr1a	22	26	25	15	23	40	15	21
Avpr1b	9	3	2	1	4	2	2	0
Avpr2	0	4	1	1	4	1	0	7
Awat2	11	6	8	12	11	8	2	5
Axdnd1	5	3	11	5	4	2	6	8
Axin1	1884	1971	1914	1936	2026	2030	1866	1886
Axin2	1417	1464	1392	1297	1424	1524	1190	1205
Axl	842	869	958	1002	746	619	686	684
Azgp1	9	10	16	16	18	13	11	8
Azi2	5739	5994	5328	5303	5154	5400	5274	4747
Azin1	16816	16817	15692	16611	16159	16794	18781	17026
Azin2	64	77	54	43	65	46	55	81
B230219D	1660	1685	1874	1964	1687	1836	1828	1789
B230307C	267	342	281	303	333	264	302	267
B2m	90981	94873	90728	92197	78677	84094	84505	76883
B3galnt1	78	75	100	87	68	55	89	95
B3galnt2	477	556	526	515	590	572	516	439
B3galt1	8	7	14	12	10	8	5	7
B3galt2	1	0	3	6	2	3	1	1

Transcriptome sequencing yielded total genetic results for the MOD and APS groups, with a total of 15,936 variables

Continued from above

B3galt4	550	567	513	529	522	607	463	547
B3galt5	10444	10681	9054	9145	13666	13612	14443	15764
B3galt6	261	252	231	200	189	212	277	195
B3gat2	71	75	73	72	52	75	79	87
B3gat3	968	995	1036	1014	1076	1073	1049	1052
B3glct	1292	1387	1431	1247	1373	1442	1387	1344
B3gnt2	1628	1872	1668	1574	1945	1876	1894	1766
B3gnt3	15019	15657	14658	14382	15749	16023	15066	14675
B3gnt4	0	2	6	0	5	1	2	0
B3gnt5	90	120	110	100	101	93	108	117
B3gnt6	75	69	67	73	92	56	134	111
B3gnt7	5657	5467	4655	4680	4358	4439	4151	4484
B3gnt8	43	29	46	52	36	29	45	25
B3gnt9	140	120	145	119	128	137	93	112
B3gntl1	177	207	166	190	178	174	157	162
B430306N	34	24	22	7	21	10	15	21
B4galnt1	13143	13508	14195	13645	13372	13392	12330	12909
B4galnt2	24313	25511	24413	25394	26161	26875	28054	25874
B4galnt3	2047	2336	1811	1756	1832	2036	2130	1898
B4galnt4	468	476	504	564	524	565	508	453
B4galt1	10037	10593	8788	9370	8618	8867	8926	9019
B4galt2	60	78	73	100	77	71	55	49
B4galt3	1541	1520	1510	1437	1506	1588	1396	1377
B4galt4	1207	1447	1416	1414	1417	1663	1625	1474
B4galt5	8009	7231	7624	8932	6472	6583	6437	6278
B4galt6	3946	4100	3919	3848	3470	3708	3878	3728
B4galt7	379	535	385	412	479	444	414	433
B4gat1	543	598	520	565	543	588	562	475
B9d1	52	80	86	46	77	54	63	69
B9d2	79	100	81	98	54	73	46	75
BC003965	793	846	862	873	917	906	788	855
BC004004	2255	2467	2293	2121	2184	2130	2067	2277
BC005537	7457	8139	7141	7121	7329	7777	7839	7327
BC005624	1191	1199	1048	1111	1143	1335	1225	1185
BC016579	448	364	378	388	469	442	339	413
BC024063	29	56	30	22	26	25	42	29
BC024139	23	16	30	27	32	27	28	15
BC024978	363	416	408	395	389	370	302	302
BC028528	35	41	43	30	30	59	28	26
BC029722	885	839	739	734	793	795	852	832
BC030500	0	0	0	0	0	0	0	3
BC031181	2989	3378	2934	2941	3051	3063	3224	3080
BC034090	29	16	24	21	14	35	43	31
BC035044	7	7	13	4	23	7	12	6
BC048403	91	143	94	116	142	116	90	96
BC049352	2	0	3	1	3	2	0	1
BC049715	5	8	20	13	7	7	2	12
BC049762	7	8	11	3	4	9	6	2
BC051019	0	0	4	1	0	4	0	0
BC051142	45	31	29	26	23	27	23	24
BC055324	152	191	163	228	160	169	175	166
BC107364	0	0	0	3	0	0	1	6
BC147527	35	26	44	19	37	13	13	13
Baalc	1	0	1	1	4	5	0	1
Baat	8	20	16	22	28	9	34	28
Babam1	2445	2500	2200	2220	2112	2305	2154	2247
Babam2	1059	1146	992	1120	999	1128	1026	1094
Bace1	203	181	240	198	130	189	184	133

Transcriptome sequencing yielded total genetic results for the MOD and APS groups, with a total of 15,936 variables

Continued from above

Bace2	362	443	444	513	414	406	352	463
Bach1	3084	3329	3053	2861	3169	3215	3724	3098
Bach2	183	211	112	148	109	120	139	89
Bad	1718	1827	1654	1828	1727	1857	1778	1803
Bag1	8086	8075	7787	8464	7651	7694	7924	7629
Bag2	134	173	120	177	172	189	142	156
Bag3	1402	1552	1462	1413	1794	1602	1461	1615
Bag4	1324	1413	1399	1430	1554	1597	1631	1492
Bag5	669	615	681	737	554	631	647	536
Bag6	5962	6304	5990	5742	5721	5641	5568	5565
Bahcc1	956	940	1080	1097	1087	1199	939	961
Bahd1	1479	1458	1450	1421	1428	1401	1342	1308
Baiap2	145	127	154	189	165	141	118	160
Baiap2l1	5570	6472	5575	4768	6771	6842	6426	5925
Baiap2l2	6295	5879	6352	6183	5653	5479	5361	5719
Baiap3	132	121	147	127	81	99	57	68
Bak1	11000	10978	10562	10229	10450	10460	9899	10117
Bambi	38	45	71	71	48	42	49	65
Banf1	3288	3374	3266	3279	3308	3253	2874	2909
Banf2	0	0	2	0	0	0	0	0
Bank1	46	36	38	44	34	61	35	39
Banp	235	304	241	246	567	347	328	462
Bap1	2062	2127	2140	1931	1983	1982	2038	1936
Bard1	196	190	149	149	183	138	129	132
Barhl1	0	0	0	0	2	0	0	0
Barx1	0	0	0	0	0	1	0	0
Barx2	1496	1900	1495	1038	1882	2062	1870	1673
Basp1	188	134	193	159	159	172	194	139
Batf	43	57	36	41	36	38	44	50
Batf2	1872	1996	1756	1831	1577	1802	1554	1568
Batf3	41	50	41	56	31	12	31	21
Bax	1332	1361	1231	1399	1396	1474	1240	1290
Baz1a	1137	1289	1280	1203	1436	1408	1281	1223
Baz1b	2777	3220	3068	2948	3564	3319	3084	3076
Baz2a	5032	5283	5170	5283	5500	5235	5427	4853
Baz2b	1509	1638	1769	1706	1490	1696	1526	1512
Bbc3	237	243	264	209	248	252	204	192
Bbip1	751	882	828	828	865	918	884	861
Bbof1	15	32	7	27	23	10	23	18
Bbox1	603	796	567	439	824	849	927	894
Bbs1	24	28	38	28	28	30	16	37
Bbs10	21	12	21	12	43	40	26	29
Bbs12	31	22	24	30	33	35	13	44
Bbs2	117	120	148	121	160	103	134	105
Bbs4	88	88	85	96	77	89	86	75
Bbs5	31	52	74	54	56	79	44	63
Bbs7	33	75	59	68	81	41	59	50
Bbs9	58	60	63	83	87	91	73	59
Bbx	260	357	389	373	348	358	339	279
Bcam	388	283	361	303	316	349	313	305
Bcan	6	0	1	1	0	0	0	3
Bcap29	446	441	349	438	470	419	464	379
Bcap31	6995	7796	7359	7091	7070	7425	7017	7156
Bcar1	4505	4553	4511	4282	4649	5029	4889	4599
Bcar3	3963	4198	4174	3966	4023	4203	4433	4168
Bcas1	855	786	951	1006	883	840	803	724
Bcas2	1056	1088	1142	1112	1058	1127	1074	1005
Bcas3	465	532	427	460	509	452	446	459

Transcriptome sequencing yielded total genetic results for the MOD and APS groups, with a total of 15,936 variables

Continued from above

Bcat1	2	7	3	5	2	5	8	3
Bcat2	681	667	650	607	728	797	665	673
Bccip	1199	1295	1375	1351	1426	1420	1330	1345
Bcdin3d	123	101	143	138	158	133	150	125
Bche	6147	6708	6500	7064	6146	6565	6632	6109
Bckdha	1613	1584	1593	1637	1626	1584	1417	1366
Bckdhb	102	90	125	89	169	106	101	92
Bckdk	1677	1810	1708	1621	1526	1629	1369	1419
Bcl10	1811	2033	1908	1990	1875	1993	1924	1936
Bcl11a	98	107	129	113	84	128	115	111
Bcl11b	766	861	877	816	749	878	788	734
Bcl2a1a	31	30	15	32	31	30	23	42
Bcl2a1b	131	151	118	126	111	122	83	91
Bcl2a1d	40	74	57	41	27	37	47	27
Bcl2l1	2225	2160	2215	2102	2097	2037	2254	2010
Bcl2l10	1	0	4	0	0	0	2	0
Bcl2l11	245	219	317	270	259	262	273	254
Bcl2l12	213	199	231	219	256	223	186	193
Bcl2l13	1990	2008	2046	2109	2143	2012	1891	1801
Bcl2l14	1466	1686	1611	1459	1717	1767	1522	1367
Bcl2l15	2887	3012	2977	3056	3045	3094	3347	3422
Bcl2l2	1582	1755	1787	1863	1792	1936	1904	1762
Bcl3	3338	3542	3409	3285	4503	4529	4097	4067
Bcl6	507	511	467	462	427	415	446	441
Bcl6b	46	65	49	89	81	81	58	64
Bcl7a	245	291	239	216	304	290	189	263
Bcl7b	860	947	979	939	944	993	902	842
Bcl7c	614	603	576	571	602	667	624	600
Bcl9	1213	1270	1425	1326	1115	1058	1093	1025
Bcl9l	1510	1384	1464	1480	1306	1389	1303	1314
Bclaf1	2470	2713	2661	2812	2673	2733	2778	2513
Bclaf3	181	125	95	157	106	144	120	79
Bco1	52	27	55	32	25	36	29	38
Bco2	3372	3932	3587	3308	3726	3780	3707	3789
Bcor	595	689	583	586	614	578	556	494
Bcorl1	629	719	653	752	577	707	690	505
Bcr	2704	2852	2720	2736	2588	2813	2997	2875
Bcs1l	363	389	391	374	341	363	314	319
Bdh1	8524	8587	8670	8427	7537	8032	7523	7326
Bdh2	11	7	6	2	26	14	4	9
Bdkrb1	7	23	25	17	22	28	7	24
Bdkrb2	463	478	434	401	351	356	300	332
Bdnf	0	5	1	1	1	0	1	2
Bdp1	916	1143	1020	934	1279	1297	1262	1017
Bean1	50	36	74	22	27	50	25	33
Becn1	4477	4734	4831	4481	4637	4472	4406	4455
Begain	8	26	15	10	10	2	2	21
Bend3	286	343	290	277	278	257	222	234
Bend4	7	7	5	23	9	8	3	9
Bend5	55	67	41	35	58	58	25	39
Bend6	10	5	5	12	5	11	7	13
Bend7	818	907	886	843	892	895	1149	826
Best1	12	11	6	6	1	1	0	2
Best3	0	0	0	0	0	2	2	1
Bet1	1842	1875	1786	1799	2170	2322	2279	1977
Bet1l	869	879	862	877	944	870	977	870
Bex1	1	0	1	10	5	14	14	2
Bex2	25	41	44	33	32	30	16	47

Transcriptome sequencing yielded total genetic results for the MOD and APS groups, with a total of 15,936 variables

Continued from above

Bex3	202	273	218	224	227	211	231	226
Bex4	2	2	4	0	0	1	1	1
Bex6	0	0	0	1	0	0	0	0
Bfar	1166	1279	1198	1383	1356	1605	1520	1287
Bfsp1	378	374	407	325	260	337	293	291
Bfsp2	0	0	0	3	0	7	4	0
Bglap	0	0	0	0	0	0	0	1
Bglap2	0	1	0	0	4	1	0	0
Bglap3	54	52	53	41	85	77	81	95
Bgn	3111	3322	3424	3472	3182	3039	2432	2597
Bhlha15	324	314	307	289	358	334	278	336
Bhlha9	0	0	0	0	1	0	0	0
Bhlhb9	59	54	43	52	50	51	43	40
Bhlhe22	9	17	24	22	18	22	19	11
Bhlhe23	0	0	0	0	0	1	0	0
Bhlhe40	2123	2291	2709	2472	3300	2974	2953	3040
Bhlhe41	36	36	7	35	56	39	26	15
Bhmt	53	36	75	21	38	27	46	16
Bhmt2	1	1	5	1	1	1	6	1
Bicc1	126	138	129	184	123	151	175	93
Bicd1	15	44	36	40	37	57	26	33
Bicd2	414	430	460	520	607	492	527	452
Bicdl1	198	192	202	168	186	193	199	162
Bicdl2	441	436	415	454	428	422	438	478
Bicra	939	983	1042	870	880	894	880	925
Bicral	1029	1142	1076	1106	1245	1259	1070	1141
Bid	442	589	509	494	448	413	441	511
Bik	7	34	6	12	7	12	13	8
Bin1	783	788	681	652	834	787	794	831
Bin2	130	135	162	169	133	136	123	113
Bin3	943	1009	934	953	967	891	855	859
Birc2	1401	1488	1313	1440	1419	1420	1402	1252
Birc3	2175	2437	2031	2111	1920	2156	2389	2079
Birc5	1163	1268	1268	1113	1406	1145	1092	1172
Birc6	4688	5561	4855	5036	5652	5529	5629	5109
Bivm	278	341	341	303	329	421	337	321
Blcap	1862	2075	1948	1863	1815	1875	1865	1861
Blk	3	0	0	6	2	2	2	3
Blm	213	239	188	181	196	195	248	199
Blmh	1331	1303	1318	1169	1456	1266	1220	1230
Blnk	1632	1939	1758	1685	1948	1867	1730	1866
Bloc1s1	809	975	840	859	876	894	845	795
Bloc1s2	723	744	798	809	976	841	775	876
Bloc1s3	697	580	644	563	563	589	598	589
Bloc1s4	448	390	454	408	422	384	390	372
Bloc1s5	404	441	393	441	336	383	382	402
Bloc1s6	1456	1708	1610	1445	1699	1738	1563	1692
Blvra	576	595	615	567	609	567	528	541
Blvrb	894	895	891	929	856	994	777	756
Blzf1	885	966	880	824	1004	995	1012	922
Bmerb1	2	3	8	11	8	1	13	1
Bmf	378	389	417	325	314	410	272	325
Bmi1	728	816	788	710	829	916	855	698
Bmp1	626	675	666	558	685	657	627	578
Bmp2	1153	907	923	929	641	644	630	572
Bmp2k	1701	1906	1818	1949	1728	1850	1702	1659
Bmp3	399	517	562	600	471	541	417	477
Bmp4	455	440	458	426	439	386	325	338

Transcriptome sequencing yielded total genetic results for the MOD and APS groups, with a total of 15,936 variables

Continued from above

Bmp5	327	399	365	417	315	347	304	270
Bmp6	68	125	119	95	118	109	56	66
Bmp7	174	146	174	138	170	149	185	157
Bmp8a	228	253	269	185	312	329	382	320
Bmp8b	56	53	38	69	58	68	56	64
Bmper	22	12	30	30	28	31	26	29
Bmpr1a	3912	4116	4231	4280	4746	4625	4566	4306
Bmpr1b	2	5	3	3	4	3	1	12
Bmpr2	1526	1686	1580	1672	1671	1939	1796	1697
Bms1	1087	1040	1149	1140	1120	1078	1009	944
Bmt2	393	346	351	341	384	373	380	413
Bmx	18	17	28	10	18	5	25	16
Bmyc	90	103	118	88	104	64	60	100
Bnc1	0	0	4	0	5	6	10	12
Bnc2	36	19	27	16	27	31	29	15
Bnip1	680	727	655	678	564	579	603	592
Bnip2	612	612	680	646	687	628	706	573
Bnip3	562	599	651	591	627	761	697	736
Bnip3l	2150	2158	2132	2112	2329	2433	2326	2105
Bnip3l-ps	140	152	159	159	169	139	197	179
Bnip5	3676	3901	2810	2610	3153	3535	3686	3187
Bnipl	2	4	1	23	10	1	6	0
Boc	118	96	145	87	105	79	78	86
Bod1	821	833	892	766	839	894	765	740
Bod1l	928	1001	891	875	968	1073	1034	888
Bok	397	361	379	353	406	429	357	346
Bola1	356	389	374	386	294	380	367	358
Bola2	341	421	405	421	413	380	360	420
Bola3	1523	1439	1429	1556	1349	1499	1264	1313
Boll	5	2	1	1	1	2	2	9
Bop1	1803	1803	1760	1602	1930	1683	1701	1700
Bora	407	455	437	368	387	380	395	330
Borcs5	343	373	305	347	285	391	335	361
Borcs6	497	684	591	565	610	638	561	583
Borcs7	187	220	217	199	174	174	218	200
Borcs8	409	545	421	460	490	464	441	495
Bpgm	3186	3781	3244	2924	3843	3964	3817	3519
Bphl	356	385	436	393	418	383	407	407
Bpi	0	0	0	1	0	0	0	0
Bpifb4	0	0	0	0	1	0	0	0
Bpifb5	0	4	0	0	0	0	0	0
Bpnt1	30189	33177	31659	30374	29968	31969	30664	30632
Bpnt2	3503	3443	3596	3717	3367	3675	3642	3488
Bptf	1799	1918	1826	1954	1817	2089	1909	1794
Braf	602	526	577	592	560	484	553	549
Brap	1654	1832	1857	1699	1696	1871	1665	1706
Brat1	477	459	459	431	522	454	548	461
Brca1	397	413	359	393	412	508	374	359
Brca2	230	207	211	189	166	210	182	160
Brcc3	658	882	768	766	734	889	728	812
Brd1	3038	3259	3305	3180	3370	3576	3527	3133
Brd2	5600	5924	5803	5492	5836	5891	5456	5393
Brd3	1715	1756	1912	1749	1811	1901	1696	1742
Brd3os	17	4	6	7	3	1	12	18
Brd4	3080	3517	3211	3056	3030	2892	2906	2837
Brd7	1502	1518	1427	1415	1673	1670	1514	1557
Brd8	1274	1408	1410	1339	1435	1464	1370	1245
Brd8dc	10	6	5	12	5	11	5	3

Transcriptome sequencing yielded total genetic results for the MOD and APS groups, with a total of 15,936 variables

Continued from above

Brd9	927	976	1007	962	911	861	855	928
Brdt	30	30	24	24	18	15	19	25
Brf1	1313	1404	1183	1343	1253	1350	1233	1164
Brf2	165	206	184	233	172	201	205	219
Bri3	1467	1490	1512	1418	1226	1267	1189	1352
Bri3bp	3175	3419	3075	3069	3385	3404	2945	3242
Bricd5	22	40	29	36	27	29	26	32
Brinp1	0	0	12	9	5	0	1	5
Brinp2	15	10	14	1	22	12	7	12
Brinp3	0	1	0	0	0	0	0	0
Brip1	449	447	462	419	511	451	440	366
Brix1	768	794	828	847	932	794	907	819
Brk1	2603	2584	2539	2496	2505	2579	2619	2143
Brms1	1240	1269	1263	1237	1310	1248	1212	1293
Brms1l	132	207	223	188	209	171	139	187
Brox	3433	3662	3278	3472	3557	3475	3658	3296
Brpf1	1184	1241	1336	1334	1164	1165	1052	1032
Brpf3	4302	4662	4739	4537	4495	4559	4537	4248
Brsk1	41	65	76	51	81	75	47	56
Brsk2	3	5	4	13	7	9	3	23
Brwd1	1339	1546	1468	1574	1573	1717	1570	1567
Brwd3	867	1056	927	1098	1097	1268	1200	1000
Bscl2	881	1005	999	965	998	879	903	930
Bsdc1	2334	2409	2316	2272	2586	2722	2706	2625
Bsg	36304	36477	35353	34992	35002	35855	34552	33975
Bsn	221	226	216	180	203	261	220	222
Bsnd	0	0	1	0	0	0	0	0
Bspry	1519	1558	1494	1639	1501	1376	1447	1607
Bst1	5349	5917	4901	4514	5564	6114	6793	5382
Bst2	3778	4956	4883	3425	4910	4555	4133	4138
Bsx	0	0	4	0	0	0	0	0
Btaf1	1497	1705	1583	1709	1690	1911	1749	1649
Btbd1	2475	2720	2416	2625	2714	2815	2737	2531
Btbd10	183	254	245	211	280	264	240	195
Btbd11	20	7	9	12	13	6	7	9
Btbd16	0	2	0	1	0	1	1	0
Btbd17	1	7	3	11	8	3	5	2
Btbd18	0	0	0	0	0	4	0	0
Btbd19	146	161	135	158	158	179	123	156
Btbd2	1490	1683	1422	1565	1489	1428	1428	1366
Btbd3	544	585	668	574	513	671	547	570
Btbd6	651	568	610	619	561	669	599	553
Btbd7	1869	2060	2139	1941	2200	2244	2140	1994
Btbd8	136	126	109	108	129	129	130	116
Btbd9	1606	1664	1649	1646	1668	1831	1880	1691
Btc	182	223	233	143	227	223	245	198
Btd	1094	1092	1090	990	1083	1211	1158	1101
Btf3	8919	9405	8989	8810	9312	9385	8897	8502
Btf3l4	578	556	553	482	466	592	598	523
Btg1	5585	6213	5423	5126	5637	5730	5406	5263
Btg2	2413	2596	2690	2544	2522	2464	2355	2259
Btg3	152	152	186	157	201	152	183	161
Btk	48	60	61	33	63	41	94	42
Btla	16	11	6	19	8	8	6	11
Btn1a1	1	0	4	0	1	1	0	2
Btn2a2	0	1	0	0	0	0	0	0
Btnl1	12849	13810	12192	11882	13670	13803	13666	12855
Btnl10	0	1	0	0	1	0	0	0

Transcriptome sequencing yielded total genetic results for the MOD and APS groups, with a total of 15,936 variables

Continued from above

Btnl2	5519	6370	5581	5147	5843	6010	6010	5525
Btnl4	4855	5769	5291	5185	4913	5429	5441	4813
Btnl6	12418	13420	12179	11268	11966	12800	13730	12301
Btnl9	6	0	0	4	0	2	0	0
Btrc	344	437	390	325	352	358	392	367
Bub1	337	512	427	446	516	495	482	455
Bub1b	1116	1209	1006	994	1217	1258	976	1081
Bub3	1689	1898	1731	1661	1935	1732	1490	1656
Bud13	384	413	423	334	436	377	346	361
Bud23	1598	1654	1641	1629	1556	1544	1634	1449
Bud31	2428	2460	2336	2248	2501	2705	2361	2301
Bves	31	39	38	40	33	44	48	20
Bysl	539	666	616	528	666	527	510	583
Bzw1	8671	9797	8658	8500	10188	9865	9558	9109
Bzw2	1818	1973	1914	1852	2046	2046	1829	1823
C030006K	426	337	364	458	375	402	387	391
C130026I2	5	6	1	2	5	5	12	6
C130050C	31	38	37	64	37	37	40	63
C130074G	3496	3714	3683	3661	3705	3797	3612	3338
C1d	1691	1794	1724	1680	1853	1750	1950	1788
C1galt1	1529	1764	1577	1621	1836	1857	1849	1724
C1galt1c1	2381	2377	2338	2356	2579	2821	2511	2569
C1qa	2323	2155	2025	2155	1835	1948	1825	1899
C1qb	2051	1721	1748	1914	1481	1464	1558	1609
C1qbp	3778	3929	3834	3698	3940	3722	3254	3598
C1qc	1762	1836	1694	1976	1532	1685	1536	1603
C1ql1	1	17	4	5	0	1	1	1
C1ql3	0	3	1	2	0	0	0	0
C1ql4	0	0	0	0	0	1	0	0
C1qtnf1	163	171	189	203	155	120	102	130
C1qtnf12	2553	2668	2507	2437	2182	2298	2139	2231
C1qtnf2	48	50	65	72	72	63	66	45
C1qtnf3	20	35	28	32	57	21	53	16
C1qtnf4	31	18	37	29	23	24	7	16
C1qtnf6	123	147	129	124	156	147	96	133
C1qtnf7	16	10	18	5	12	5	8	11
C1qtnf9	35	32	55	21	49	27	22	20
C1ra	654	805	773	699	660	688	603	540
C1rb	3	0	2	4	2	1	3	4
C1rl	59	43	40	59	42	56	63	58
C1s1	1216	1391	1241	1430	1186	1196	1126	1056
C1s2	12	7	8	4	1	4	8	7
C2	1548	2000	1671	1403	1844	1777	1578	1391
C2cd2	341	295	268	309	359	371	262	280
C2cd2l	7152	7422	7005	6568	7760	7881	8055	7453
C2cd3	813	844	892	918	890	940	854	830
C2cd4a	1	0	0	1	0	0	1	0
C2cd4b	62	24	58	46	81	53	40	58
C2cd4c	38	47	40	35	40	42	27	40
C2cd4d	1	1	3	0	0	0	5	1
C2cd5	1303	1394	1367	1310	1233	1373	1385	1202
C2cd6	0	0	0	1	0	0	0	0
C3	3000	2649	2922	3294	2890	2913	3029	2701
C330007P	834	828	864	714	838	881	807	743
C330018D	450	440	425	408	505	446	489	480
C3ar1	298	328	224	262	262	294	233	219
C4b	2679	2851	2568	2471	2314	2091	2267	2045
C4bp	49	45	85	43	59	49	74	42

Transcriptome sequencing yielded total genetic results for the MOD and APS groups, with a total of 15,936 variables

Continued from above

C5ar1	141	128	132	148	116	130	157	112
C5ar2	30	9	14	30	18	39	20	8
C6	271	241	268	240	267	205	208	241
C7	148	145	113	153	156	154	143	127
C77080	18334	19218	18453	17572	18036	18553	18425	17540
C87436	276	290	285	246	311	328	240	275
C8a	3	2	1	0	9	0	1	2
C8b	0	0	2	1	1	0	1	2
C8g	135	77	113	97	73	83	161	147
C9	2	0	0	0	12	3	0	3
C9orf72	348	366	364	332	340	400	368	384
Caap1	416	433	255	375	403	385	413	381
Cab39	9196	9641	8931	8939	9770	9760	10357	9524
Cab39l	1635	1830	1728	1636	1672	1695	1715	1717
Cabcoco1	37	55	62	39	40	45	39	61
Cabin1	2845	2953	2850	3044	2874	2800	2644	2621
Cables1	368	422	436	296	424	374	502	443
Cables2	963	924	847	921	959	869	821	834
Cabp1	22	17	23	10	8	9	8	8
Cabp2	0	5	0	1	33	29	14	24
Cabp4	13	14	10	8	10	1	7	5
Cabp7	4	9	11	17	2	4	12	9
Cabyr	4	4	0	6	0	1	0	8
Cacfd1	1547	1611	1433	1472	1536	1725	1633	1565
Cachd1	239	308	336	294	339	295	296	291
Cacna1a	121	157	164	123	148	142	158	164
Cacna1b	37	41	41	56	55	40	63	45
Cacna1c	99	118	102	109	102	84	123	80
Cacna1d	83	85	96	122	88	49	83	60
Cacna1e	94	67	64	71	55	55	47	38
Cacna1f	1	0	1	2	6	0	0	0
Cacna1g	53	87	73	63	78	82	57	50
Cacna1h	285	369	400	387	355	286	266	298
Cacna1i	0	0	1	0	4	1	0	1
Cacna1s	58	45	30	41	37	43	16	29
Cacna2d1	93	139	174	183	142	126	169	133
Cacna2d2	18	17	14	49	15	24	23	27
Cacna2d3	7	10	12	7	14	9	24	14
Cacna2d4	0	0	2	0	0	0	1	0
Cacnb1	39	18	43	24	47	36	51	27
Cacnb2	30	29	23	38	43	36	28	40
Cacnb3	412	402	365	481	425	419	300	335
Cacnb4	4	23	7	9	5	19	1	6
Cacng2	0	0	0	0	0	5	0	0
Cacng4	1	0	8	1	1	7	1	0
Cacng5	0	1	0	1	0	2	0	7
Cacng6	0	0	1	0	0	0	0	1
Cacng7	109	120	145	159	116	152	125	90
Cacng8	1	5	5	1	1	3	7	6
Cactin	1250	1287	1222	1269	1266	1273	1075	1237
Cacul1	2946	2926	2904	2845	3073	3030	3280	2721
Cacybp	1583	1687	1665	1633	2071	2255	2009	1775
Cad	1361	1288	1211	1219	1315	1391	1198	1144
Cadm1	156	184	114	134	161	142	144	124
Cadm2	1	0	3	1	1	4	4	1
Cadm3	70	98	111	74	90	66	72	99
Cadm4	18	35	50	30	34	48	16	16
Cadps	52	22	43	40	33	40	45	47

Transcriptome sequencing yielded total genetic results for the MOD and APS groups, with a total of 15,936 variables

Continued from above

Cadps2	701	646	655	659	635	659	609	648
Cage1	8	15	7	6	11	7	25	20
Calb1	0	1	0	1	0	0	1	0
Calb2	104	94	103	103	114	96	106	118
Calca	1	0	1	1	0	1	0	0
Calcb	35	15	22	13	17	26	21	22
Calcoco1	403	461	454	426	375	405	489	441
Calcrl	190	278	263	239	205	193	218	208
Cald1	1386	1465	1610	1681	1587	1788	1809	1569
Calhm2	69	56	92	67	60	63	42	67
Calhm5	24	29	26	39	30	45	44	34
Calhm6	123	109	78	100	81	65	49	96
Calm1	76437	84629	76128	74104	79971	85290	85539	80510
Calm2	15364	15920	16187	17533	15166	15772	15383	14431
Calm3	16497	18277	17473	16624	18003	18627	17329	16858
Calm5	0	0	4	0	0	0	0	0
Calml4	6438	6912	6257	6310	6487	7100	7116	6577
Caln1	0	0	1	1	1	0	0	1
Calr	27796	30441	29018	26469	33169	31091	28350	28220
Calr3	2	0	0	5	1	8	8	1
Calr4	1	0	2	4	2	1	0	0
Calu	7396	7862	7250	7557	8209	8372	8224	7908
Caly	1	13	2	0	0	1	6	5
Camk1	261	184	245	279	218	236	207	246
Camk1d	2920	3131	3182	3126	2489	2600	2520	2423
Camk1g	12	0	8	4	5	4	9	1
Camk2a	32	26	58	52	34	39	48	15
Camk2b	661	508	613	801	344	413	457	503
Camk2d	5127	5072	5062	5386	4265	4459	4528	4662
Camk2g	428	525	466	488	467	500	385	395
Camk2n1	1490	1592	1589	1653	1457	1514	1502	1344
Camk2n2	42	38	28	30	21	33	22	32
Camk4	13	11	16	12	11	52	20	35
Camkk1	121	120	128	138	125	128	105	124
Camkk2	262	223	194	162	201	197	200	199
Camkmt	431	499	493	433	462	461	411	371
Camkv	0	1	0	0	4	1	0	0
Caml	700	728	710	725	765	695	677	757
Camp	0	0	4	0	0	1	0	0
Camsap1	427	428	491	363	485	491	391	417
Camsap2	124	126	140	150	135	139	115	163
Camsap3	2931	3021	3105	2958	2951	3091	2981	2902
Camta1	96	113	94	102	136	94	114	129
Camta2	3331	3452	3292	3166	3355	3216	3253	3056
Cand1	3551	3992	3802	3812	4092	3908	3841	3741
Cand2	168	210	199	161	178	146	136	133
Cant1	6734	7044	6670	6706	7078	6852	7112	6665
Canx	14973	17559	16246	15905	19470	19489	18124	17141
Cap1	18747	19908	18237	17558	19217	19941	19263	18487
Cap2	153	201	208	159	153	176	211	238
Capg	2455	2375	2481	2616	2707	2503	2293	2513
Capn1	5816	5866	5833	5719	5870	6106	5676	5521
Capn10	943	910	906	944	795	845	950	840
Capn11	14	28	13	11	5	8	2	1
Capn12	16	37	13	13	15	32	18	12
Capn13	1037	722	1067	1430	505	478	476	487
Capn15	2543	2703	2612	2449	2761	2614	2575	2575
Capn2	744	810	722	735	940	735	779	759

Transcriptome sequencing yielded total genetic results for the MOD and APS groups, with a total of 15,936 variables

Continued from above

Capn3	17	7	6	16	27	16	6	3
Capn5	1879	1629	1666	1673	1458	1476	1451	1479
Capn6	0	5	3	5	1	7	1	0
Capn7	1374	1424	1410	1383	1745	1697	1550	1460
Capn8	176	196	219	169	150	182	170	177
Capn9	709	609	700	766	639	652	537	615
Capns1	7605	7933	7650	7634	7447	7257	7026	7144
Capns2	6	0	0	0	2	0	0	0
Caprin1	11731	13191	12130	11775	13727	13723	12978	12517
Caprin2	126	148	133	113	119	122	127	131
Capsl	5	5	4	11	4	8	0	0
Capza1	5233	6107	5223	5418	5801	5996	5715	5424
Capza2	6402	7024	6554	6147	6992	7364	7273	6771
Capzb	11588	12246	11477	11800	11141	11718	10914	11220
Car10	0	0	1	0	0	0	0	0
Car11	8	3	1	16	3	1	4	3
Car12	456	420	491	368	495	414	428	376
Car13	2852	3176	2807	2702	3269	3313	3360	3038
Car14	18	27	32	30	16	19	19	13
Car15	36	42	55	68	49	53	47	42
Car2	3431	3774	3248	2937	2665	2782	2843	2648
Car3	124	89	215	166	472	526	631	295
Car4	14925	8614	12126	19796	4413	5203	6648	6715
Car5a	0	0	1	2	2	3	4	1
Car5b	12	5	4	18	27	40	54	15
Car6	10	12	6	17	10	3	5	17
Car7	15	17	25	25	12	8	19	11
Car8	699	642	610	613	584	553	512	534
Car9	3987	4144	4232	3984	4572	4236	3814	4383
Card10	1135	1008	975	1063	1001	934	1020	1001
Card11	507	650	626	559	576	615	516	518
Card14	652	654	511	516	524	558	476	490
Card19	1152	948	1063	1042	915	974	950	1035
Card6	502	602	563	546	484	521	505	507
Card9	27	25	28	38	36	33	36	17
Carf	129	127	93	107	119	117	132	104
Carhsp1	2456	2818	2714	2763	2958	2767	2585	2557
Carm1	1585	1442	1379	1418	1383	1379	1416	1379
Carmil1	2518	3098	2812	2614	2740	2844	2668	2551
Carmil2	64	36	57	60	16	34	38	23
Carmil3	9	28	12	22	11	9	6	26
Carnmt1	428	408	455	450	501	472	436	362
Carns1	22	29	12	21	27	31	25	11
Cars	1310	1521	1424	1497	1371	1430	1347	1372
Cars2	585	597	614	707	675	719	617	685
Cartpt	16	19	5	12	8	6	24	5
Casc1	1	11	8	8	4	9	4	4
Casc3	1549	1617	1738	1634	1758	1675	1634	1566
Casd1	438	518	478	483	538	429	552	504
Cask	2635	2975	2952	2881	2769	2973	2788	2844
Caskin1	6	7	17	30	8	20	5	14
Caskin2	2308	2348	2382	2417	2419	2394	2222	2341
Casp1	4606	4881	4586	4548	4568	5197	4536	4185
Casp12	154	156	130	160	109	153	141	157
Casp14	0	0	0	0	1	2	1	4
Casp2	1148	1419	1326	1176	1313	1236	1068	1216
Casp3	14616	15148	13329	13679	13141	13469	12015	11370
Casp4	1314	1550	1359	1152	1291	1479	1508	1192

Transcriptome sequencing yielded total genetic results for the MOD and APS groups, with a total of 15,936 variables

Continued from above

Casp6	9415	10454	10364	9852	10346	10445	9806	9155
Casp7	17332	17795	16116	16907	14715	14995	15264	14539
Casp8	4943	5662	4816	4466	4962	5282	5046	4779
Casp8ap2	549	624	593	435	571	645	668	560
Casp9	1347	1491	1323	1354	1415	1375	1330	1113
Casq1	4	6	0	6	0	8	14	1
Casq2	58	105	120	114	123	116	117	82
Casr	11	1	12	2	4	5	2	1
Cass4	9	24	3	12	11	13	5	2
Cast	5164	5650	5044	5177	5529	5937	5902	5244
Castor1	36	35	33	30	38	34	26	42
Castor2	1294	1583	1199	1131	1482	1569	1706	1463
Casz1	5144	5250	5167	5170	4757	5267	5340	4811
Cat	7145	8592	7784	6505	8111	8399	8411	7332
Catip	1	5	8	0	0	1	0	4
Catsper2	90	121	96	75	141	91	94	120
Catsper3	0	4	0	0	0	0	0	0
Catsper4	33	22	15	21	33	30	31	33
Catsperd	0	9	1	1	5	0	11	10
Catspere2	46	25	49	29	48	49	42	44
Catsperg1	6	7	3	6	4	3	5	3
Catsperz	4	0	0	0	0	0	1	0
Cav1	906	926	1068	1074	993	1041	1016	1078
Cav2	136	197	222	212	201	225	229	224
Cav3	12	8	23	13	11	17	20	10
Cavin1	1138	1155	1170	1276	1254	1268	1257	1279
Cavin2	396	418	451	389	464	491	496	423
Cavin3	293	273	274	309	325	280	308	275
Cavin4	0	2	0	0	0	0	0	2
Cbarp	31	58	43	58	60	58	54	35
Cbfa2t2	742	828	777	736	762	862	730	737
Cbfa2t3	261	277	283	264	219	216	201	233
Cbfb	1037	1148	1130	1093	1140	1186	1050	1092
Cbl	2878	3151	2864	3133	3120	3333	3265	2666
Cblb	514	511	463	410	420	477	401	422
Cblc	3530	3604	3664	3538	3609	3944	3586	3550
Cblif	8	0	6	4	2	0	0	0
Cbll1	543	616	626	586	652	651	604	592
Cbln1	4	0	1	2	8	6	1	1
Cbln2	29	29	5	7	5	23	4	14
Cbln3	9	21	21	24	17	19	1	8
Cbr1	9883	10560	9846	9319	10462	10996	9960	10548
Cbr2	0	1	2	24	7	7	6	14
Cbr3	86	73	81	100	107	132	92	131
Cbr4	232	276	270	223	269	298	231	239
Cbs	21	29	19	16	86	48	50	36
Cbwd1	467	532	410	470	585	577	561	503
Cbx1	1190	1222	1249	1135	1303	1235	1301	1128
Cbx2	253	233	258	257	255	211	254	209
Cbx3	790	880	756	885	912	844	786	738
Cbx4	466	479	453	465	398	440	446	346
Cbx5	2327	2461	2317	2426	2531	2677	2340	2198
Cbx6	443	476	456	504	420	455	451	359
Cbx7	556	584	616	676	607	589	537	531
Cbx8	156	199	236	197	209	201	234	174
Cby1	324	330	253	245	305	305	232	310
Cby3	0	2	0	0	0	0	0	0
Cc2d1a	1881	2055	1883	1854	1707	1823	1805	1628

Transcriptome sequencing yielded total genetic results for the MOD and APS groups, with a total of 15,936 variables

Continued from above

Cc2d1b	926	986	1110	954	922	776	903	848
Cc2d2a	122	126	159	125	122	120	71	89
Cc2d2b	0	0	0	0	4	0	0	1
Ccar1	1880	2234	2173	2150	2191	2600	2300	2110
Ccar2	2063	2073	2011	2066	1926	1700	1846	2008
Ccbe1	42	72	76	82	89	90	66	75
Ccdc102a	79	122	114	127	117	141	75	74
Ccdc103	2	0	0	1	0	5	1	8
Ccdc106	7	6	17	15	4	6	11	16
Ccdc107	1377	1351	1538	1441	1204	1359	1253	1187
Ccdc112	78	62	55	67	103	72	50	92
Ccdc114	490	503	486	494	507	524	555	585
Ccdc115	599	580	602	580	639	616	548	563
Ccdc116	473	500	552	560	502	527	556	516
Ccdc117	702	924	858	840	982	889	781	931
Ccdc12	1213	1238	1261	1264	1102	1226	1111	1116
Ccdc120	742	761	836	656	793	952	944	916
Ccdc122	156	108	129	135	96	67	177	142
Ccdc124	1605	1532	1554	1558	1544	1673	1386	1470
Ccdc125	104	98	106	106	66	83	89	82
Ccdc126	77	65	85	70	106	90	54	63
Ccdc127	823	877	857	804	927	881	885	899
Ccdc13	0	1	0	0	0	0	0	0
Ccdc130	206	200	204	256	187	263	207	214
Ccdc134	398	401	374	467	616	482	466	468
Ccdc136	28	24	28	17	23	22	14	14
Ccdc137	470	446	560	473	561	621	490	453
Ccdc138	110	169	155	89	162	174	187	145
Ccdc14	139	89	147	124	140	110	81	86
Ccdc141	68	95	82	64	54	90	102	68
Ccdc142	205	227	249	222	204	225	190	149
Ccdc146	0	0	6	2	3	1	9	3
Ccdc148	6	15	19	14	5	6	9	20
Ccdc149	118	86	104	91	111	107	109	101
Ccdc15	67	121	89	84	90	105	91	74
Ccdc150	0	0	1	0	4	0	1	1
Ccdc151	0	2	0	0	4	0	0	0
Ccdc152	205	179	202	191	79	140	141	103
Ccdc154	2	1	0	1	0	0	0	0
Ccdc157	86	125	98	127	106	113	105	93
Ccdc158	7	2	9	15	18	9	12	18
Ccdc159	23	20	25	29	27	13	40	8
Ccdc160	4	0	0	0	1	2	0	1
Ccdc162	6	9	11	7	9	7	5	11
Ccdc163	174	186	190	204	171	192	173	145
Ccdc166	145	69	53	94	85	66	91	72
Ccdc167	303	427	386	326	360	341	294	402
Ccdc169	0	4	2	3	12	0	0	0
Ccdc17	94	108	96	115	88	103	90	94
Ccdc170	8	8	10	8	11	0	8	5
Ccdc171	33	46	19	46	46	66	56	32
Ccdc173	1	1	1	0	7	5	1	2
Ccdc174	364	394	354	392	376	411	386	374
Ccdc175	2	0	0	1	0	3	0	2
Ccdc177	2	4	1	1	9	0	4	5
Ccdc18	57	54	85	16	42	59	88	58
Ccdc180	0	0	0	0	7	1	2	2
Ccdc181	168	167	159	191	187	166	175	175

Transcriptome sequencing yielded total genetic results for the MOD and APS groups, with a total of 15,936 variables

Continued from above

Ccdc184	10	13	6	6	0	3	5	6
Ccdc186	1998	2072	1868	1887	2284	2340	2648	2291
Ccdc187	1	1	1	2	0	1	0	0
Ccdc189	38	47	27	28	29	23	41	29
Ccdc190	3	7	5	5	3	4	10	9
Ccdc191	79	91	95	85	91	109	88	76
Ccdc192	6	1	0	0	0	0	1	0
Ccdc22	482	476	434	403	512	548	435	437
Ccdc24	10	7	20	6	3	7	1	12
Ccdc25	2082	2228	2140	2034	2082	2335	2393	2051
Ccdc28a	702	697	687	699	649	704	601	618
Ccdc28b	85	91	84	74	53	58	65	86
Ccdc3	257	297	231	317	275	220	242	205
Ccdc30	12	1	1	2	1	4	3	6
Ccdc32	1786	1746	1690	1801	1677	1720	1661	1708
Ccdc33	0	0	0	4	0	0	1	1
Ccdc34	896	966	894	871	1043	944	883	915
Ccdc38	30	15	19	21	32	23	26	16
Ccdc39	14	6	8	16	20	5	11	3
Ccdc40	1	2	3	6	1	6	3	0
Ccdc42	21	14	18	13	25	18	27	7
Ccdc43	529	559	594	604	600	661	547	525
Ccdc47	2741	2931	2822	2730	3147	3233	3268	2865
Ccdc50	1817	2094	2047	1953	2104	2136	2154	1800
Ccdc51	515	576	619	559	614	606	554	563
Ccdc57	39	45	36	47	43	33	37	40
Ccdc58	523	504	531	640	596	562	626	509
Ccdc59	820	821	728	847	865	980	767	805
Ccdc6	3893	4229	4038	3661	4049	3895	4066	3775
Ccdc60	1	9	8	6	3	4	10	1
Ccdc61	410	402	430	421	397	381	365	365
Ccdc62	30	54	37	29	47	32	54	38
Ccdc63	1	0	3	1	2	0	1	5
Ccdc65	1	7	0	2	4	1	1	2
Ccdc66	268	250	266	206	256	313	309	255
Ccdc68	876	955	820	838	827	915	875	808
Ccdc69	47	55	30	40	49	51	35	26
Ccdc71	1335	1247	1379	1381	1352	1384	1241	1181
Ccdc71l	4610	4366	4840	5100	3895	3909	3830	3885
Ccdc73	0	2	5	1	3	0	2	1
Ccdc74a	6	10	10	17	2	11	1	3
Ccdc77	143	225	178	139	170	200	200	210
Ccdc78	1	0	0	0	0	0	0	0
Ccdc8	47	28	51	42	24	66	27	47
Ccdc80	126	92	80	109	99	127	128	87
Ccdc81	1	0	0	0	0	0	0	0
Ccdc82	555	627	613	594	609	559	644	569
Ccdc83	0	1	0	1	1	0	0	0
Ccdc84	172	275	238	294	317	233	256	283
Ccdc85a	6	7	15	8	8	14	1	6
Ccdc85b	263	261	346	297	325	283	296	282
Ccdc85c	4496	4578	4361	4143	4340	4522	4033	3963
Ccdc86	478	498	538	550	539	590	480	557
Ccdc87	34	21	34	5	10	21	21	15
Ccdc88a	49	50	39	58	39	45	70	46
Ccdc88b	962	961	836	856	855	789	852	767
Ccdc88c	3412	3749	3649	3732	4059	4047	3857	3413
Ccdc89	0	1	5	1	2	2	2	2

Transcriptome sequencing yielded total genetic results for the MOD and APS groups, with a total of 15,936 variables

Continued from above

Ccdc9	1200	1203	1295	1259	1092	1073	1061	1147
Ccdc90b	925	1023	961	854	942	1144	1186	954
Ccdc91	1705	1573	1537	1549	1599	1659	1654	1590
Ccdc92	73	92	50	119	71	79	57	84
Ccdc92b	32	35	22	28	25	23	12	15
Ccdc93	1529	1816	1604	1712	1860	1953	1895	1678
Ccdc96	13	19	5	4	8	8	5	15
Ccdc97	1460	1444	1266	1477	1522	1396	1391	1378
Ccdc9b	121	98	99	107	92	102	95	103
Ccer2	0	1	0	0	0	0	0	0
Cchcr1	218	226	203	199	234	240	190	203
Ccin	0	0	0	0	0	0	3	1
Cck	1808	1709	1700	1779	1405	1408	1444	1471
Cckar	35	26	20	16	121	118	82	108
Cckbr	3	0	1	0	1	0	0	0
Ccl1	0	0	7	0	1	1	0	2
Ccl11	361	416	438	444	390	346	319	324
Ccl12	38	29	39	59	42	52	34	34
Ccl17	1	5	4	7	10	11	0	4
Ccl19	12	0	1	14	0	0	0	12
Ccl2	68	38	64	86	97	61	71	84
Ccl20	229	229	227	181	373	434	404	358
Ccl21a	327	306	252	393	209	219	245	202
Ccl22	66	40	25	37	55	45	32	37
Ccl24	186	161	217	226	144	142	119	112
Ccl25	11794	13089	12260	11511	13983	14465	13517	11973
Ccl27a	58	45	71	31	32	33	35	30
Ccl28	1949	2542	2076	1772	3379	3688	2981	2672
Ccl3	23	35	24	29	5	21	16	18
Ccl4	62	41	59	56	15	17	20	17
Ccl5	343	351	352	372	296	237	199	254
Ccl6	4077	3968	4307	4855	3546	3966	4117	4115
Ccl7	34	27	32	55	35	54	36	46
Ccl8	191	223	305	269	131	145	121	142
Ccl9	1147	1252	1239	1246	1222	1157	1088	1261
Ccm2	666	698	581	541	740	656	613	579
Ccm2l	30	56	40	31	21	39	29	28
Ccn1	216	172	257	207	217	218	208	208
Ccn2	317	337	368	414	378	268	318	315
Ccn3	34	26	17	12	24	34	28	24
Ccn4	17	39	29	29	23	39	45	38
Ccn5	2	7	0	2	4	1	0	0
Ccn6	2	5	1	0	0	1	0	2
Ccna1	11	16	11	6	19	4	5	13
Ccna2	2167	2385	2329	2091	2657	2131	1911	2184
Ccnb1	1207	1448	1303	1190	1417	1228	1242	1276
Ccnb1-ps	12	20	18	19	19	16	9	13
Ccnb1ip1	0	0	0	4	0	0	0	0
Ccnb2	1146	1193	1178	1220	1266	1114	1030	1275
Ccnc	696	754	725	775	815	802	748	767
Ccnd1	3078	3374	3071	3054	3293	3214	2952	3131
Ccnd2	3406	3871	3747	3437	3913	3968	3159	3267
Ccnd3	1228	1167	1306	1206	1531	1387	1203	1192
Ccndbp1	1139	1157	1053	1253	1089	1040	1073	1121
Ccne1	352	311	301	353	295	232	288	233
Ccne2	273	261	217	229	262	328	278	262
Ccnf	595	555	601	562	708	641	512	504
Ccng1	3463	3384	3223	3439	4112	4141	3933	3850

Transcriptome sequencing yielded total genetic results for the MOD and APS groups, with a total of 15,936 variables

Continued from above

Ccng2	5354	5747	5082	5259	4421	4922	5659	4878
Ccnh	441	439	508	444	474	523	454	458
Ccni	8401	8453	8181	7873	7358	7866	7769	7507
Ccnj	125	127	138	102	110	151	115	94
Ccnjl	432	519	349	428	382	447	351	304
Ccnk	1397	1612	1458	1456	1574	1378	1359	1385
Ccnl1	1473	1671	1572	1624	1679	1724	1557	1390
Ccnl2	2902	2903	3281	2985	3329	3275	3004	2877
Ccno	4	6	1	0	5	3	1	0
Ccnq	564	484	451	479	484	527	475	510
Ccnt1	1421	1469	1465	1500	1497	1582	1578	1532
Ccnt2	1003	1000	969	1069	946	980	1001	879
Ccny	2625	2689	2645	2609	2757	2663	2709	2502
Ccnyl1	1552	1464	1324	1287	1169	1360	1394	1235
Ccp110	86	103	117	122	97	131	100	101
Ccpg1	764	912	879	800	999	972	920	835
Ccpg1os	38	31	45	54	38	42	46	37
Ccr1	323	368	339	351	363	373	304	360
Ccr10	89	103	108	58	114	95	76	116
Ccr1l1	0	0	0	0	1	1	0	0
Ccr2	43	48	28	43	27	19	9	27
Ccr3	32	24	7	14	20	26	11	6
Ccr4	17	10	23	26	59	25	40	37
Ccr5	230	270	218	275	156	171	201	154
Ccr6	7	2	4	9	2	3	4	0
Ccr7	6	1	12	7	6	1	3	7
Ccr8	4	0	0	0	0	0	0	0
Ccr9	165	132	108	125	102	140	113	95
Ccrl2	293	366	311	284	195	208	258	206
Ccs	1462	1370	1264	1517	1342	1412	1285	1310
Ccsap	82	83	98	65	91	71	83	105
Ccser1	786	844	743	837	691	767	709	603
Ccser2	2957	3108	2863	2748	3302	3431	3724	3382
Cct2	5705	6227	6156	6098	6516	6571	5887	5966
Cct3	5246	5618	5476	5347	6000	5824	5162	5256
Cct4	4495	4781	4522	4411	5072	5389	4518	4611
Cct5	5633	5754	5769	5437	6325	6347	5178	5468
Cct6a	5482	5749	5261	5459	6068	6290	5407	5361
Cct6b	4	4	2	3	7	8	12	4
Cct7	5581	6148	5998	5330	6342	6094	5532	5445
Cct8	4502	5077	4624	4455	5036	5180	4706	4589
Cct8l1	0	0	0	2	0	0	0	0
Ccz1	2691	2912	2915	2706	2707	2741	2552	2580
Cd101	27	61	23	35	30	36	33	23
Cd109	5	8	5	16	10	12	12	11
Cd14	438	417	473	387	460	490	443	490
Cd151	5120	5102	4675	4882	5508	5160	4851	4860
Cd160	21	45	28	35	37	25	33	27
Cd163	28	26	32	40	29	61	50	42
Cd163l1	9	7	2	1	14	1	2	8
Cd164	9768	11064	10110	10125	10795	10997	10936	10316
Cd164l2	0	0	1	1	4	0	0	0
Cd177	405	417	448	416	396	429	353	390
Cd180	32	36	34	38	58	43	19	15
Cd19	12	1	0	5	3	2	0	0
Cd1d1	23	58	22	52	67	60	26	51
Cd2	27	18	15	20	7	15	4	14
Cd200	390	374	472	383	354	421	327	282

Transcriptome sequencing yielded total genetic results for the MOD and APS groups, with a total of 15,936 variables

Continued from above

Cd200r1	15	39	32	12	11	23	27	11
Cd200r2	12	11	8	13	19	10	19	19
Cd200r3	1	0	0	1	4	0	0	0
Cd200r4	51	48	75	56	66	57	66	56
Cd207	1	0	0	0	0	0	0	0
Cd209a	0	0	3	1	6	3	8	1
Cd209b	2	4	13	9	21	18	22	13
Cd209c	0	0	0	0	0	1	1	1
Cd209e	0	0	0	1	1	0	0	8
Cd209f	1	2	3	2	4	15	15	5
Cd209g	0	0	0	1	4	4	0	0
Cd22	97	118	91	143	92	89	71	75
Cd226	22	29	23	33	37	36	25	17
Cd244a	101	86	58	86	119	82	80	71
Cd247	67	44	51	35	29	49	45	26
Cd248	106	99	74	80	78	79	76	85
Cd24a	3306	3595	3516	3424	3397	3347	3377	3324
Cd27	7	6	11	3	6	16	5	3
Cd274	427	527	451	410	279	269	307	226
Cd276	203	202	257	225	176	217	149	171
Cd28	65	41	59	52	54	52	61	41
Cd2ap	7010	7600	7069	6825	7892	8244	8136	7446
Cd2bp2	2266	2281	2151	2172	2145	2202	2139	2123
Cd300a	131	146	117	138	116	86	101	112
Cd300c	0	0	4	0	0	0	0	0
Cd300c2	131	134	118	148	109	90	132	101
Cd300e	2	4	16	19	10	7	12	6
Cd300lb	4	2	3	7	5	1	0	0
Cd300ld	54	40	50	37	63	53	28	29
Cd300ld3	0	0	1	0	0	0	0	0
Cd300ld4	0	1	0	0	2	0	0	1
Cd300ld5	0	0	0	0	0	0	0	4
Cd300lf	41	54	38	18	29	40	45	22
Cd300lg	127	138	140	126	101	97	115	116
Cd302	2088	2387	2165	2003	2328	2329	2342	2280
Cd320	263	298	305	268	307	334	285	297
Cd33	54	60	55	90	61	62	83	73
Cd34	321	408	364	334	393	316	401	321
Cd36	1931	2001	1856	1898	1880	1959	2174	1651
Cd37	64	69	42	86	64	57	44	37
Cd38	5640	6067	5217	5477	6207	6231	5681	5819
Cd3d	34	41	41	48	40	44	38	25
Cd3e	83	106	86	66	61	62	62	60
Cd3eap	211	231	235	233	260	235	229	256
Cd3g	106	131	91	136	143	103	123	120
Cd4	273	258	253	345	276	218	209	230
Cd40	95	102	106	108	38	41	63	46
Cd40lg	25	1	7	4	5	1	6	8
Cd44	1121	1105	1168	1263	1195	1079	1082	985
Cd46	27	31	16	19	27	20	30	18
Cd47	4470	4544	4414	4630	4679	4762	4481	4323
Cd48	155	108	132	115	132	98	78	131
Cd5	54	37	42	39	28	20	23	27
Cd52	369	405	350	343	314	337	316	397
Cd53	346	371	326	417	316	320	287	263
Cd55	3817	2468	3366	5090	1440	1582	1805	1629
Cd55b	7	12	20	30	6	21	15	2
Cd59a	227	182	250	236	243	224	218	226

Transcriptome sequencing yielded total genetic results for the MOD and APS groups, with a total of 15,936 variables

Continued from above

Cd59b	12	10	12	11	21	28	19	13
Cd5l	3	6	3	8	0	6	3	3
Cd6	55	59	66	87	67	60	49	53
Cd63	5876	5853	5814	5929	5896	5760	5769	5486
Cd68	241	212	199	223	210	198	207	186
Cd69	42	53	56	49	35	26	53	34
Cd7	190	186	174	171	113	132	125	100
Cd72	273	203	235	188	128	158	186	144
Cd74	36993	28611	30543	39117	16858	18228	18682	18662
Cd79a	157	196	158	146	141	97	112	144
Cd79b	139	123	129	114	113	133	105	126
Cd80	20	17	19	9	26	34	17	20
Cd81	3520	3743	3734	3537	3617	3727	3562	3427
Cd82	10925	10720	10751	11026	9524	10227	10447	10249
Cd83	142	115	109	110	155	87	92	103
Cd84	54	75	72	80	80	62	78	43
Cd86	61	68	64	49	60	44	57	51
Cd8a	198	222	191	172	174	128	143	108
Cd8b1	27	16	29	13	26	25	8	1
Cd9	1573	1684	1822	1861	1955	1766	1409	1668
Cd93	428	422	458	530	451	537	478	383
Cd96	53	34	50	27	26	50	33	33
Cd99	281	301	233	262	255	286	243	247
Cd99l2	204	203	237	247	215	225	227	207
Cda	7599	7449	7544	7935	6200	6802	6484	6460
Cdadc1	957	923	866	1081	1084	1050	949	898
Cdan1	553	547	560	548	618	641	576	504
Cdc123	2153	1993	1973	2053	2137	2143	2181	1879
Cdc14a	667	716	725	732	810	790	750	752
Cdc14b	1507	1519	1337	1429	1383	1419	1352	1376
Cdc16	924	1095	964	936	1120	1112	1037	1065
Cdc20	1557	1591	1603	1610	1610	1573	1305	1343
Cdc20b	4	0	7	2	4	6	1	4
Cdc23	804	871	807	872	831	823	788	828
Cdc25a	3229	3720	3651	3246	4013	4079	3707	3612
Cdc25b	713	734	650	782	799	631	666	619
Cdc25c	153	187	167	152	181	144	132	140
Cdc26	683	787	800	737	759	802	762	755
Cdc27	1066	1292	1094	1109	1242	1207	1263	1218
Cdc34	2603	2599	2718	2404	2655	2642	2456	2505
Cdc37	4490	4511	4446	4130	4625	4535	4028	4240
Cdc37l1	1219	1290	1487	1166	1274	1289	1406	1212
Cdc40	1069	1270	1189	1162	1138	1072	1229	1216
Cdc42	19443	20022	18967	19019	19393	19962	19205	18443
Cdc42bpa	568	574	743	631	636	631	604	647
Cdc42bpb	7574	7831	7446	7331	7420	7621	7616	7340
Cdc42bpg	3070	3130	3140	3471	2947	2944	2813	2954
Cdc42ep1	534	454	572	474	542	525	506	454
Cdc42ep2	1404	1358	1514	1525	1478	1465	1404	1445
Cdc42ep3	235	265	214	242	233	275	226	208
Cdc42ep4	3069	3207	3038	2959	3209	3367	3080	2875
Cdc42ep5	3161	3277	3147	3094	3235	3207	3016	3042
Cdc42se1	3708	3881	3788	3557	3566	3523	3295	3307
Cdc42se2	5988	6623	5360	5648	6540	6579	6972	6620
Cdc45	311	327	341	329	346	335	268	300
Cdc5l	1526	1798	1621	1551	1986	1917	1781	1796
Cdc6	306	264	279	226	306	315	279	260
Cdc7	143	166	176	136	176	158	133	119

Transcriptome sequencing yielded total genetic results for the MOD and APS groups, with a total of 15,936 variables

Continued from above

Cdc73	1305	1314	1301	1238	1454	1666	1445	1318
Cdca2	370	439	481	436	504	448	406	436
Cdca3	1510	1779	1708	1619	1866	1650	1389	1653
Cdca4	763	837	763	744	698	707	661	773
Cdca5	301	343	347	282	352	362	281	290
Cdca7	1821	1849	1897	1818	1928	1859	1555	1735
Cdca7l	466	500	442	523	483	473	496	409
Cdca8	942	1038	1000	790	1028	959	822	1004
Cdcp1	3382	3862	3602	3573	3880	3871	3898	3474
Cdcp3	6	1	0	0	35	11	12	10
Cdh1	25897	28476	26964	26663	27172	26010	25491	24852
Cdh10	0	1	2	0	0	1	2	0
Cdh11	320	302	362	262	302	400	291	284
Cdh12	0	1	0	0	0	0	0	0
Cdh13	34	35	41	53	54	59	54	46
Cdh15	6	0	0	0	0	0	0	0
Cdh17	59197	63849	55945	57000	58474	58748	60711	56655
Cdh18	1	0	0	0	4	1	1	1
Cdh19	17	9	53	23	21	13	12	17
Cdh2	43	25	46	43	18	28	22	34
Cdh20	1	0	0	0	0	0	0	1
Cdh22	1	1	1	5	0	2	0	0
Cdh23	1	1	0	2	1	4	1	1
Cdh24	57	82	55	67	47	58	40	61
Cdh26	2	0	0	0	4	0	1	0
Cdh3	15	5	8	7	8	6	13	5
Cdh4	5	8	4	12	4	5	14	1
Cdh5	419	447	483	456	383	405	380	430
Cdh6	14	22	22	23	27	25	32	26
Cdh8	2	0	1	0	0	1	10	1
Cdh9	8	1	1	1	2	1	1	0
Cdhr1	1	2	0	2	0	1	1	2
Cdhr2	46922	51224	40786	40061	46657	49308	51049	43579
Cdhr3	0	0	0	1	0	0	2	0
Cdhr4	3	20	17	3	6	11	1	20
Cdhr5	46906	50408	45886	42419	46367	47651	49334	45161
Cdin1	225	269	250	276	331	309	270	239
Cdip1	2656	3242	3180	2941	2931	3027	2664	2694
Cdipt	4535	4798	4446	4447	4518	4699	4694	4799
Cdiptos	22	12	11	17	12	15	7	23
Cdk1	1336	1266	1331	1208	1302	1224	1105	1200
Cdk10	436	453	342	406	359	455	309	350
Cdk11b	3926	4123	3821	4089	3802	4042	4308	3973
Cdk12	1558	1711	1719	1743	1719	1679	1770	1554
Cdk13	2448	2631	2511	2489	2804	2553	2518	2227
Cdk14	38	95	41	74	59	52	53	62
Cdk15	0	1	0	0	1	1	4	0
Cdk16	6809	7096	6484	6955	6656	6262	6117	6209
Cdk17	249	283	329	265	226	344	291	291
Cdk18	7587	8114	6751	6417	7297	7753	7202	6853
Cdk19	518	517	563	643	452	532	461	469
Cdk2	643	663	660	721	658	519	571	558
Cdk20	85	109	114	91	79	86	86	92
Cdk2ap1	1015	981	1076	1007	1078	913	778	1021
Cdk2ap2	2989	3157	2975	3018	3227	3115	2825	3019
Cdk4	3338	3464	3515	3333	3432	3299	2942	3052
Cdk5	626	704	825	687	703	691	655	632
Cdk5r1	8	7	13	21	7	8	11	12

Transcriptome sequencing yielded total genetic results for the MOD and APS groups, with a total of 15,936 variables

Continued from above

Cdk5r2	41	45	34	42	30	24	23	22
Cdk5rap1	338	395	370	294	340	403	330	334
Cdk5rap2	408	455	434	509	475	481	381	370
Cdk5rap3	2267	2571	2099	1992	2459	2549	2303	2272
Cdk6	522	637	592	591	682	604	573	577
Cdk7	802	972	966	850	958	919	887	906
Cdk8	2115	2161	1980	2152	2333	2201	2233	2106
Cdk9	1762	1945	1873	1848	1694	1827	1864	1756
Cdkal1	292	318	336	305	333	337	341	264
Cdkl1	6	2	16	23	18	2	4	7
Cdkl2	147	212	175	130	185	228	197	159
Cdkl3	79	51	62	60	77	64	56	46
Cdkl4	2	2	3	2	5	9	2	3
Cdkl5	29	53	31	33	56	48	60	62
Cdkn1a	8718	8826	8699	9160	8998	9388	9159	8602
Cdkn1b	1726	1668	1746	1669	1665	1645	1556	1633
Cdkn1c	267	323	312	248	316	298	322	265
Cdkn2a	2	2	2	4	1	2	0	9
Cdkn2aip	797	897	692	662	748	903	814	742
Cdkn2aipr	1628	1890	1853	1710	1858	1926	1653	1830
Cdkn2b	5048	4635	4373	4950	3414	3865	3803	3610
Cdkn2c	431	387	361	359	368	345	302	326
Cdkn2d	229	245	245	217	204	253	202	212
Cdkn3	196	176	192	162	191	205	213	198
Cdnf	10	15	6	16	11	21	10	21
Cdo1	543	572	575	566	553	673	483	507
Cdon	192	186	181	145	232	229	271	197
Cdpf1	412	346	378	331	431	388	382	311
Cdr2	7152	7855	7200	6976	9071	9434	10423	9080
Cdr2l	84	90	104	97	70	91	131	86
Cds1	16859	17344	16266	16487	15122	15159	15864	15178
Cds2	2235	2687	2493	2454	2560	2433	2596	2492
Cdsn	24	12	29	9	22	4	11	6
Cdt1	1104	1091	1048	932	948	1075	849	907
Cdv3	4326	4609	4034	4400	4406	4699	4176	4237
Cdv3-ps	25	27	24	24	27	29	18	20
Cdx1	5102	5147	5498	5396	5275	5013	4758	5078
Cdx2	7427	7172	7044	7075	7027	7107	6729	6572
Cdyl	376	427	480	520	465	392	410	358
Cdyl2	17	24	21	26	21	50	38	39
Ceacam1	34128	38154	32104	29798	36362	37411	38990	34904
Ceacam1C	159	156	192	179	129	122	139	183
Ceacam12	0	0	0	0	1	1	0	0
Ceacam16	0	3	0	0	0	1	1	0
Ceacam18	3575	3532	3170	3195	3373	3621	3707	3560
Ceacam2	488	504	489	355	455	418	477	385
Ceacam2C	6236	6290	5030	5581	5240	5799	6821	5841
Ceacam9	0	4	0	0	0	1	0	0
Cebpa	2639	2716	2780	2344	3106	3354	2869	2821
Cebpb	2567	2464	2637	2487	2512	2574	2366	2588
Cebpd	1590	2087	2549	1960	1582	1653	1608	1925
Cebpe	15	14	2	0	8	7	8	4
Cebpg	3176	3479	3386	3547	3805	3779	3840	3381
Cebpz	674	809	754	665	924	928	778	817
Cebpzos	472	521	502	539	511	510	512	477
Cecr2	0	8	3	2	6	10	2	9
Cel	2758	3835	1668	812	10262	9482	6433	8995
Cela1	4253	5838	2392	1108	18871	17318	10537	14015

Transcriptome sequencing yielded total genetic results for the MOD and APS groups, with a total of 15,936 variables

Continued from above

Cela2a	6572	8472	3897	1668	26322	23083	15216	21666
Cela3a	2	0	0	0	0	1	1	4
Cela3b	2034	2566	1179	494	7923	7016	5092	7041
Celf1	5774	5834	5581	5937	6226	6093	5640	5410
Celf2	177	295	222	261	281	207	245	189
Celf3	82	86	86	104	100	58	81	75
Celf4	37	38	35	21	22	27	34	22
Celf5	302	373	458	421	354	257	327	356
Celf6	21	13	39	18	15	11	27	14
Celsr1	476	519	417	477	486	512	499	525
Celsr2	64	56	71	58	52	71	73	75
Celsr3	165	143	111	174	151	160	123	230
Cemip	73	51	36	43	50	69	90	107
Cemip2	1374	1558	1413	1351	1302	1366	1486	1387
Cend1	28	39	40	55	20	19	41	43
Cenpa	865	1008	955	808	1027	923	792	781
Cenpb	8868	8421	8456	8624	7062	7320	7363	6995
Cenpc1	440	480	418	395	424	437	390	430
Cenpe	481	589	587	581	618	673	603	564
Cenpf	441	488	435	424	486	527	453	456
Cenph	120	124	127	106	108	125	95	104
Cenpi	183	191	197	160	236	158	201	176
Cenpj	279	352	280	284	375	367	352	284
Cenpk	100	110	73	109	93	95	79	84
Cenpl	328	382	392	397	402	387	417	394
Cenpm	116	136	127	90	133	81	97	86
Cenpn	83	116	105	111	100	78	90	96
Cenpo	212	198	231	219	176	233	183	185
Cenpp	146	158	137	136	140	154	130	159
Cenpq	161	227	190	227	197	185	215	194
Cenps	80	92	97	95	105	134	103	86
Cenpt	199	248	245	237	215	229	175	173
Cenpu	160	175	150	156	183	157	131	150
Cenpv	2561	2636	2952	2491	2554	2685	2465	2507
Cenpw	236	289	238	253	261	261	250	243
Cenpx	955	950	1002	1008	1168	1007	1010	959
Cep104	692	757	702	713	775	715	731	701
Cep112	8	1	3	0	10	3	6	17
Cep120	585	718	668	671	685	705	720	639
Cep126	11	31	17	12	18	14	15	23
Cep128	183	233	204	221	218	202	159	162
Cep131	80	81	105	71	110	65	71	61
Cep135	163	252	194	214	224	184	238	239
Cep152	170	208	251	226	241	196	167	159
Cep162	197	218	228	192	238	298	211	258
Cep164	431	505	582	489	446	471	457	463
Cep170	101	133	146	96	138	121	136	123
Cep170b	4136	4327	4036	3995	4144	4327	4028	3970
Cep19	666	674	584	712	629	703	730	778
Cep192	695	716	630	735	718	738	658	677
Cep20	1267	1175	984	1152	1104	1131	1235	1265
Cep250	551	607	670	532	542	514	499	459
Cep290	114	104	105	150	145	151	164	119
Cep295	453	436	420	465	496	476	419	439
Cep295nl	2	1	0	0	0	0	0	1
Cep350	4217	4905	4388	4418	4882	5243	5111	4245
Cep41	107	71	91	79	120	95	134	90
Cep43	493	450	489	430	585	458	445	514

Transcriptome sequencing yielded total genetic results for the MOD and APS groups, with a total of 15,936 variables

Continued from above

Cep44	195	198	209	255	205	191	196	213
Cep55	268	336	289	367	408	424	303	331
Cep57	460	464	482	487	467	507	475	515
Cep57l1	134	166	135	172	117	142	147	120
Cep63	460	490	436	452	468	492	419	417
Cep68	301	315	334	308	336	372	322	291
Cep70	218	172	202	175	153	229	206	178
Cep72	178	201	157	195	165	147	171	211
Cep76	364	322	379	302	416	429	325	377
Cep78	90	99	101	66	98	115	103	144
Cep83	534	500	563	564	575	555	523	508
Cep85	376	403	400	383	413	375	384	333
Cep85l	21	50	50	56	43	35	57	34
Cep89	440	492	436	485	363	367	378	386
Cep95	341	345	375	352	412	427	343	319
Cep97	59	82	77	77	88	73	47	57
Cept1	3787	3796	3571	3789	4109	3915	4188	3707
Cercam	51	60	79	77	33	61	73	45
Cerk	2426	2590	2275	2237	2559	2524	2452	2184
Cerkl	6	4	9	8	4	7	2	5
Cers2	6355	6807	6432	6422	6108	6088	6168	5979
Cers3	2	1	0	0	0	7	0	1
Cers4	590	814	792	604	718	614	642	635
Cers5	312	311	314	286	316	273	230	334
Cers6	3480	4195	4214	4325	4310	4436	4250	4025
Cert1	2212	2509	2334	2410	2194	2415	2264	2195
Ces1a	0	0	0	1	0	0	0	0
Ces1b	11	4	2	8	13	12	1	4
Ces1c	108	68	86	75	85	107	57	63
Ces1d	180	165	177	132	165	221	167	128
Ces1e	515	594	522	485	631	620	482	548
Ces1f	7398	9222	7103	6143	10067	10568	9461	8470
Ces1g	56	132	61	60	116	135	87	92
Ces1h	0	0	0	0	0	1	0	0
Ces2a	2820	3099	1586	1468	3230	4111	3516	2663
Ces2b	63	72	52	41	83	113	65	40
Ces2c	7982	7518	7942	9836	7849	7701	10685	7890
Ces2e	27469	31239	25824	24355	28462	30021	30524	28203
Ces2f	15	11	12	15	12	27	26	21
Ces2g	1245	1454	1387	1134	1802	1771	1429	1296
Ces2h	22	31	12	21	34	41	26	37
Ces3a	4	7	12	1	35	11	10	24
Ces3b	4	1	2	3	1	10	0	1
Cetn2	451	471	517	499	498	547	489	548
Cetn3	738	926	744	918	904	830	931	814
Cetn4	3	2	6	8	11	4	8	7
Cfap100	1	1	0	4	2	3	0	0
Cfap126	30	25	33	42	16	31	22	33
Cfap157	13	5	7	12	2	5	5	17
Cfap20	716	662	666	630	659	596	566	575
Cfap206	0	0	0	0	0	1	0	0
Cfap20dc	1	4	4	1	0	4	1	0
Cfap221	0	0	4	1	0	0	0	0
Cfap298	560	625	624	453	570	579	536	533
Cfap300	7	30	18	24	12	11	28	15
Cfap36	170	183	192	166	184	166	157	192
Cfap410	73	83	45	75	76	90	84	68
Cfap43	5	5	5	0	6	1	1	0

Transcriptome sequencing yielded total genetic results for the MOD and APS groups, with a total of 15,936 variables

Continued from above

Cfap44	0	0	0	0	1	0	0	0
Cfap45	2	1	2	1	0	2	1	8
Cfap46	0	1	0	0	0	0	1	1
Cfap47	11	3	3	6	13	7	9	6
Cfap52	0	3	4	5	0	4	1	0
Cfap53	2	0	0	1	2	1	0	1
Cfap54	1	0	0	8	0	0	0	1
Cfap57	0	0	0	0	6	0	1	0
Cfap61	0	1	0	0	0	2	0	0
Cfap65	1	5	7	7	2	4	0	5
Cfap69	25	11	15	14	11	25	22	12
Cfap70	0	0	0	2	1	0	0	4
Cfap73	0	0	0	2	2	1	0	0
Cfap74	45	34	71	40	25	42	11	22
Cfap77	4	2	8	3	2	0	0	0
Cfap97	606	680	552	577	693	685	655	561
Cfap97d2	1	1	0	0	0	0	0	0
Cfap99	0	0	3	0	4	0	0	5
Cfb	7560	8920	8035	7168	8147	8429	8205	7593
Cfd	130	109	339	313	419	658	662	452
Cfdp1	966	1022	1086	915	1108	1272	1097	1119
Cfh	408	564	555	589	630	589	562	467
Cfhr1	0	1	0	1	0	2	1	0
Cfhr2	146	146	155	257	182	217	225	198
Cfi	733	854	816	703	1007	961	902	1032
Cfl1	30586	31796	30181	29876	29222	30690	28662	28669
Cfl2	1328	1369	1346	1369	1331	1239	1291	1284
Cflar	2229	2329	2532	2334	2575	2571	2414	2239
Cfp	260	192	227	268	231	192	225	165
Cftr	4682	5277	5120	5086	5596	5509	5282	4413
Cgas	212	258	227	203	240	245	167	161
Cggbp1	3227	3361	3313	3156	3130	3341	3183	3227
Cgn	5876	6388	6127	6341	5747	6163	6385	6105
Cgnl1	79	83	115	91	136	111	144	76
Cgref1	12374	12677	11879	12082	11753	12759	13129	12174
Cgrrf1	643	728	772	772	794	758	736	828
Ch25h	51	53	55	61	13	36	29	34
Chac1	120	169	115	163	102	96	124	161
Chac2	540	555	558	584	644	646	677	670
Chad	22	23	9	13	32	27	18	13
Chadl	18	19	15	11	13	0	5	16
Chaf1a	717	785	776	666	733	734	621	633
Chaf1b	403	410	332	287	321	306	283	257
Champ1	400	559	409	435	420	386	356	344
Chat	0	1	0	0	1	2	6	0
Chchd1	1051	1091	1124	1126	1131	1116	976	1110
Chchd10	10583	9743	10095	10587	9228	8862	8692	9396
Chchd2	22506	21901	21463	22335	22715	22865	21469	21922
Chchd3	7313	7588	7729	7956	7207	7649	7253	7103
Chchd4	1367	1479	1281	1310	1356	1488	1369	1306
Chchd5	270	251	271	225	255	226	187	212
Chchd6	146	193	160	141	111	167	159	144
Chchd7	2255	2216	2167	2150	2118	2066	1972	2110
Chd1	2255	2474	2278	2350	2381	2610	2712	2362
Chd1l	670	790	739	837	699	682	696	674
Chd2	1987	2311	2087	2218	2052	2196	2310	2123
Chd3	4266	4398	4305	4333	4934	5429	5022	4671
Chd4	6076	6634	6241	6205	5716	5778	5683	5579

Transcriptome sequencing yielded total genetic results for the MOD and APS groups, with a total of 15,936 variables

Continued from above

Chd5	30	37	35	59	33	51	38	13
Chd6	654	657	753	666	598	638	580	558
Chd7	880	1001	914	1048	882	963	854	918
Chd8	2063	2397	2242	2170	2337	2289	2087	1953
Chd9	1449	1690	1546	1616	1643	1830	1770	1407
Chdh	10593	10825	10594	11368	10090	10979	10409	9284
Chek1	168	166	176	150	166	157	151	126
Chek2	436	490	470	430	477	424	402	436
Cherp	2106	2041	2214	2067	2120	2257	1956	2127
Chfr	2332	2417	2335	2228	2387	2409	2231	2164
Chga	1834	1899	2054	1923	1893	1885	1863	1732
Chgb	4977	5587	5618	5677	4841	5151	5009	4640
Chic1	652	670	717	779	555	678	589	595
Chic2	1066	1079	1008	969	1084	1022	942	944
Chid1	1790	2032	1837	1684	2139	2124	2101	2049
Chil1	7	0	1	8	2	4	5	9
Chil3	0	0	1	0	1	0	5	0
Chil4	1	0	0	0	0	0	0	1
Chil5	0	0	0	0	0	0	0	1
Chil6	4	0	0	10	15	7	8	3
Chka	3573	4171	4295	3950	5428	5422	4951	4772
Chkb	2342	2207	2328	2296	2144	2326	2319	2270
Chl1	30	55	47	82	42	50	59	52
Chm	754	896	731	757	775	802	897	736
Chml	345	478	410	367	568	638	538	484
Chmp1a	7794	7640	7410	7363	7014	7228	7358	7192
Chmp1b	4117	4159	3676	3889	3616	3783	3990	3560
Chmp2a	4105	4381	4304	4162	4083	4471	4210	4074
Chmp2b	4722	5293	4701	4506	5170	5249	5206	4874
Chmp3	4729	4774	4717	4728	4777	4894	4871	4752
Chmp4b	12217	13055	12233	11893	12443	12219	11940	11751
Chmp4c	2364	2355	2393	2148	2497	2752	2731	2393
Chmp5	3750	3945	3742	3651	4139	4269	4322	4111
Chmp6	3318	3440	3037	2880	2784	3020	2862	2928
Chmp7	2035	2111	1868	1931	2092	2003	1915	1953
Chn1	38	47	43	28	36	23	31	58
Chn2	1869	1837	1693	1694	1667	1798	1908	1825
Chodl	12	14	15	13	14	28	20	16
Chordc1	1081	1141	1073	1053	2064	2091	1626	1546
Chp1	9411	9860	9266	9165	9636	10116	10126	9616
Chp2	12829	13316	12124	12534	11796	13011	13318	12164
Chpf	460	397	395	420	540	459	383	417
Chpf2	932	917	906	860	734	858	780	819
Chpt1	8900	9789	8962	8704	9971	10603	10194	8991
Chrac1	919	907	815	868	896	800	843	755
Chrd	58	39	24	39	32	36	40	56
Chrdl1	68	94	71	121	48	68	107	86
Chrdl2	0	0	0	0	0	1	0	0
Chrm1	72	118	151	127	131	116	107	180
Chrm2	167	200	194	164	182	202	196	176
Chrm3	57	42	53	88	67	43	67	37
Chrm4	366	287	228	384	230	316	304	283
Chrna1	0	0	0	1	0	1	0	0
Chrna10	6	19	5	22	9	11	9	20
Chrna2	7	13	7	12	16	10	7	2
Chrna3	34	40	47	34	24	30	38	14
Chrna4	0	0	0	0	0	0	1	0
Chrna5	12	1	1	5	1	4	0	2

Transcriptome sequencing yielded total genetic results for the MOD and APS groups, with a total of 15,936 variables

Continued from above

Chrna7	1	1	0	1	1	1	1	0
Chrna9	6	0	0	0	0	0	0	0
Chrnb1	102	88	119	112	113	125	112	103
Chrnb2	56	49	54	52	39	23	46	26
Chrnb3	0	0	1	0	0	0	0	0
Chrnb4	43	57	43	27	29	29	48	38
Chrnd	0	0	0	0	0	0	1	1
Chrne	3	1	0	1	0	1	0	0
Chst1	51	38	54	56	47	44	31	36
Chst10	12	8	8	11	2	11	3	5
Chst11	117	128	94	129	142	109	80	82
Chst12	97	114	130	100	119	93	127	77
Chst13	7	2	2	12	2	6	4	16
Chst14	67	94	53	54	62	54	29	51
Chst15	279	333	335	290	284	248	271	279
Chst2	137	122	142	109	170	160	152	131
Chst3	6	13	5	13	6	2	7	2
Chst4	2646	2924	2553	2592	2617	2617	2493	2657
Chst5	0	0	0	1	0	1	0	0
Chst7	6	10	14	15	27	2	18	4
Chst8	2	16	10	6	17	3	2	2
Chst9	3	7	2	0	1	0	4	2
Chsy1	148	133	159	138	141	112	130	114
Chsy3	16	6	7	10	5	16	2	11
Chtf18	280	245	278	198	279	206	195	213
Chtop	3111	3615	3242	3111	3172	3312	3043	3012
Chuk	5960	6755	5981	6169	5557	5637	6012	5351
Churc1	1340	1487	1270	1398	1288	1390	1250	1337
Ciao1	1285	1333	1168	1279	1254	1246	1293	1175
Ciao2a	4629	4217	4370	4527	4313	4759	4330	4297
Ciao2b	1146	1221	1138	1284	1234	1179	1113	1038
Ciao3	1397	1309	1340	1329	1372	1267	1267	1361
Ciapin1	2347	2362	2235	2124	2420	2191	2249	2029
Ciart	35	35	26	35	36	14	32	37
Cib1	8187	8034	8444	8267	7807	8095	7786	7670
Cib2	56	35	40	55	55	33	59	36
Cib3	0	0	0	0	8	0	1	0
Cibar1	103	88	109	154	156	142	107	121
Cibar2	0	5	0	0	0	1	1	0
Cic	2634	2881	2605	2736	2416	2614	2623	2533
Cidea	1	3	7	4	20	2	4	9
Cideb	8636	9019	8563	8077	8105	8946	8777	8469
Cidec	3630	3323	3200	3388	3759	3957	4186	3760
Ciita	1553	1203	1201	1720	787	774	697	710
Cilk1	2815	3086	2921	2786	2759	2753	2952	2679
Cilp	86	111	59	82	111	57	99	73
Cilp2	1	5	0	0	1	0	0	1
Cinp	415	472	475	451	458	491	471	484
Cip2a	575	685	679	684	798	730	784	688
Cipc	806	815	922	1022	918	975	967	847
Cir1	343	422	368	395	418	434	436	393
Cirbp	713	711	714	754	436	466	470	441
Cisd1	3007	2944	2940	2864	3037	3031	2955	2909
Cisd2	1915	2084	2020	1958	2034	2047	2085	1843
Cisd3	3530	3762	3845	4170	3377	3574	3239	3488
Cish	462	306	336	518	317	221	271	259
Cit	322	348	380	342	301	318	275	266
Cited1	0	0	1	0	4	3	0	0

Transcriptome sequencing yielded total genetic results for the MOD and APS groups, with a total of 15,936 variables

Continued from above

Cited2	2884	2872	2440	2275	2232	2519	2433	2328
Cited4	71	85	106	97	83	90	79	77
Ciz1	1758	1901	1884	1961	1701	1640	1592	1562
Ckap2	330	398	332	332	371	342	298	317
Ckap2l	439	477	500	515	543	483	489	527
Ckap4	724	751	765	707	791	761	654	814
Ckap5	1411	1691	1665	1463	1652	1756	1626	1538
Ckb	49312	44160	44535	46871	44530	44056	41520	42992
Cklf	25	37	38	36	56	27	25	63
Ckm	99	148	127	149	78	149	162	168
Ckmt1	92378	93265	90678	90372	84948	89178	86573	83372
Ckmt2	1	0	2	1	2	4	1	1
Cks1b	350	397	410	420	384	442	337	389
Cks2	674	743	778	747	761	751	591	737
Clasp1	1391	1495	1550	1459	1380	1363	1437	1365
Clasp2	613	710	667	685	564	638	634	541
Clasrp	917	866	790	1000	872	888	806	888
Clba1	50	46	49	28	40	63	38	74
Clca1	30892	29508	28883	33165	29328	30400	27686	24788
Clca2	15	14	6	17	6	23	11	26
Clca3a1	481	630	598	633	651	662	631	578
Clca3a2	482	476	541	529	521	539	534	499
Clca3b	15619	18121	18416	16152	17067	17335	16159	15699
Clca4a	5190	6192	3978	3254	6827	7718	9339	7731
Clca4b	37750	45833	33899	27456	43746	49968	52717	42284
Clcc1	1181	1220	1175	1056	1293	1417	1290	1344
Clcf1	13	16	27	14	5	29	14	7
Clcn1	33	58	26	42	43	24	14	24
Clcn2	8295	8638	7772	7768	7454	7442	7742	7552
Clcn3	5649	6104	5722	5989	6213	6495	6337	6030
Clcn4	2339	2383	2399	2266	2373	2397	2419	2205
Clcn5	1555	1894	1593	1576	1977	1932	1918	1584
Clcn6	127	129	94	154	133	85	98	139
Clcn7	1114	1148	1137	1176	1042	953	1006	954
Clcnka	0	1	0	0	0	0	0	0
Clcnkb	0	0	1	0	0	0	0	0
Cldn1	1	5	4	2	2	2	1	4
Cldn10	7	6	6	1	4	3	12	13
Cldn11	0	0	1	0	2	0	0	1
Cldn12	1592	1709	1590	1567	1858	1762	1673	1675
Cldn14	1	0	1	0	1	0	0	0
Cldn15	27128	26977	25847	27239	26065	26493	26412	26420
Cldn18	4	0	0	0	0	0	0	0
Cldn19	0	0	1	0	0	0	0	1
Cldn2	2550	3354	3136	2439	3893	3697	2905	3166
Cldn20	5	1	6	2	4	1	1	0
Cldn22	0	0	0	0	0	0	1	0
Cldn23	1497	1682	1511	1558	1349	1616	1519	1586
Cldn3	15228	15396	15232	15066	15814	15784	15003	14527
Cldn34c1	7	0	1	1	3	0	1	5
Cldn4	2586	2201	2433	2874	2481	2618	2728	2615
Cldn5	123	103	82	109	106	67	81	66
Cldn6	5	7	1	2	0	2	6	5
Cldn7	29249	30105	28552	26908	28404	28747	27409	27011
Cldn8	4	5	1	1	0	4	4	5
Cldn9	26	4	15	12	5	2	8	5
Cldnd1	4900	4627	4787	5366	3695	4141	4177	3823
Cldnd2	7	0	0	1	0	7	0	2

Transcriptome sequencing yielded total genetic results for the MOD and APS groups, with a total of 15,936 variables

Continued from above

Clec10a	25	21	13	20	29	19	14	35
Clec11a	25	19	14	20	18	30	32	35
Clec12a	3	3	7	15	5	8	1	6
Clec12b	0	1	0	0	0	0	0	0
Clec14a	108	110	147	150	150	111	102	112
Clec16a	1723	1697	1714	1620	1691	1697	1581	1570
Clec1a	22	17	21	19	6	32	31	13
Clec1b	80	67	46	79	54	31	50	53
Clec2d	2127	2596	2506	2159	2302	2557	2241	2180
Clec2e	3946	3877	3627	3739	3604	3647	4113	3687
Clec2f	1	0	1	0	0	0	0	1
Clec2g	7	1	1	2	5	5	1	0
Clec2h	35323	35796	34339	35898	31212	33264	34334	32467
Clec2i	13	18	24	2	18	11	17	6
Clec2l	1	0	5	1	0	3	1	2
Clec3a	0	0	0	0	1	0	0	0
Clec3b	234	267	301	257	316	303	288	257
Clec4a1	107	84	71	68	60	57	68	90
Clec4a2	143	105	125	126	144	119	133	152
Clec4a3	41	66	13	64	28	39	31	23
Clec4a4	8	5	8	2	10	3	9	5
Clec4b1	5	2	4	1	0	7	0	0
Clec4b2	0	0	1	4	1	0	0	0
Clec4d	2	0	0	0	4	1	0	1
Clec4e	6	0	1	1	0	0	1	0
Clec4f	2	2	0	3	1	1	1	3
Clec4g	0	0	0	1	0	1	0	0
Clec4n	246	285	255	289	268	294	290	227
Clec5a	28	40	31	30	24	40	12	35
Clec7a	464	417	432	391	420	369	287	305
Clec9a	2	10	8	17	5	8	8	3
Clgn	2	5	8	2	2	4	7	0
Clhc1	10	1	0	0	0	8	0	0
Clic1	11083	11731	10757	10779	10751	11389	11223	10771
Clic3	6	0	1	2	3	0	0	13
Clic4	3739	3748	3626	3601	3824	3582	3706	3748
Clic5	17859	19093	17205	16987	16415	18271	18949	15510
Clic6	803	1021	847	837	881	875	780	817
Clint1	13085	14507	13380	12734	15155	15325	14664	14231
Clip1	835	918	930	949	1080	1019	954	1030
Clip2	4143	4127	4227	3937	4157	4080	3623	3672
Clip3	86	79	88	87	65	78	90	50
Clip4	17	22	4	8	16	26	20	25
Clk1	1408	1657	1721	1579	1895	1753	1927	1747
Clk2	109	73	91	79	89	104	86	74
Clk3	1684	1705	1665	1769	1980	2133	1665	1908
Clk4	689	684	710	705	739	676	607	658
Clmn	1818	2011	2275	2129	2004	2083	1990	1838
Clmp	437	392	502	462	379	384	421	395
Cln3	333	266	262	244	295	288	275	279
Cln5	429	594	596	565	666	628	632	573
Cln6	469	521	391	437	439	423	396	400
Cln8	530	498	488	492	628	631	543	512
Clnk	22	10	15	20	6	14	15	11
Clns1a	1321	1447	1380	1213	1333	1350	1060	1148
Clock	2887	3072	3215	3263	3291	3474	3566	3023
Clp1	631	713	733	655	659	699	608	678
Clpb	777	779	740	741	765	787	746	830

Transcriptome sequencing yielded total genetic results for the MOD and APS groups, with a total of 15,936 variables

Continued from above

Clpp	984	1002	1056	1132	1000	1007	971	1040
Clps	1001	1286	832	631	3894	3825	2526	3067
Clptm1	9986	10076	9123	9626	9852	9548	9485	9196
Clptm1l	6461	7213	6813	6119	7192	6939	6823	6665
Clpx	2702	2683	2543	2395	2664	2646	2696	2567
Clrn3	10441	11425	10339	9898	11004	11751	12269	11198
Clspn	333	390	355	376	282	364	305	273
Clstn1	6868	7789	7526	7313	7624	7220	6868	6866
Clstn2	15	33	15	28	16	37	19	31
Clstn3	15	13	10	4	13	12	13	10
Clta	8657	8770	8700	8520	8909	8536	8368	8862
Cltb	2734	2784	2799	2769	2652	2791	2597	2518
Cltc	26749	29938	27173	26595	28212	30086	28882	26918
Cltrn	14	4	7	14	5	5	7	6
Clu	781	435	433	701	1378	1318	1178	1241
Cluap1	230	261	211	214	252	264	232	229
Cluh	9177	9421	9684	9015	9503	8985	7818	7930
Clvs1	10	9	9	9	13	11	9	11
Clvs2	1	0	2	0	0	0	1	0
Clybl	665	750	672	894	663	737	689	682
Cma1	0	0	0	0	0	4	1	0
Cmah	109	108	98	135	93	126	117	110
Cmas	5411	5981	5817	5439	6154	6478	6092	5803
Cmbl	3088	3450	3360	3119	3485	3394	3478	3279
Cmc1	651	720	660	707	628	693	642	583
Cmc2	347	328	382	355	375	397	283	333
Cmc4	37	44	44	64	40	47	31	34
Cmip	5741	5999	5794	5880	5001	5195	5434	4917
Cmklr1	144	108	102	148	132	139	125	136
Cmpk1	12727	14011	12737	12625	13912	14178	13611	13227
Cmpk2	3363	5099	3941	2809	3476	3966	3572	3358
Cmss1	127	148	149	131	170	148	132	159
Cmtm3	218	181	227	185	180	194	189	154
Cmtm4	6339	6477	7187	6692	7065	7043	6824	6100
Cmtm5	2	1	6	0	0	0	2	1
Cmtm6	9206	9929	9093	9067	8630	8823	9025	8949
Cmtm7	384	273	316	300	381	372	314	327
Cmtm8	57	74	53	67	66	38	59	58
Cmtr1	3668	4323	3851	3685	4052	3663	3773	3519
Cmtr2	243	254	288	240	247	281	310	247
Cmya5	9	10	6	3	11	3	10	8
Cnbd2	422	370	371	359	292	347	338	297
Cnbp	8440	9020	8604	8322	9170	9485	8876	8452
Cndp1	498	583	494	468	521	566	588	549
Cndp2	24619	26254	24153	22994	23608	26115	29296	26528
Cnep1r1	1841	1918	1759	1895	1768	1985	1761	1819
Cnga1	1	1	1	0	1	0	0	0
Cnga3	3	11	0	1	1	0	4	4
Cnga4	1	2	5	1	0	1	1	0
Cngb1	0	0	0	4	6	5	3	9
Cngb3	1	0	1	1	0	0	0	0
Cnih1	3336	3174	3125	2931	3125	3123	2916	2993
Cnih2	13	21	11	30	4	9	1	7
Cnih3	0	0	0	0	0	1	0	0
Cnih4	3197	3228	3230	3381	2803	2938	3091	2783
Cnksr1	1505	1778	1717	1557	1694	1606	1501	1517
Cnksr2	1	7	4	0	2	1	2	3
Cnksr3	873	1093	911	819	967	934	898	869

Transcriptome sequencing yielded total genetic results for the MOD and APS groups, with a total of 15,936 variables

Continued from above

Cnmd	0	0	1	0	0	0	0	0
Cnn1	2196	2089	2622	2608	2084	2201	2547	2265
Cnn2	1097	987	1016	1163	968	944	957	1051
Cnn3	2387	2673	2577	2301	2292	2437	2151	2128
Cnnm1	73	72	51	65	49	79	74	39
Cnnm2	328	412	310	286	430	443	392	405
Cnnm3	471	363	416	451	410	417	382	438
Cnnm4	6364	6876	6469	6584	6954	6966	6775	6454
Cnot1	8234	9479	8622	8690	9108	9521	9128	8438
Cnot10	1330	1583	1409	1394	1501	1409	1396	1442
Cnot11	1116	1162	1018	1111	1104	1057	1029	1013
Cnot2	1734	1812	1723	1770	1971	1902	1717	1622
Cnot3	1634	1694	1681	1655	1663	1769	1405	1566
Cnot4	679	666	726	684	718	730	690	622
Cnot6	3511	3696	3306	3607	3691	3763	3464	3517
Cnot6l	1534	1769	1807	1912	1829	1798	1817	1542
Cnot7	1641	1770	1778	1852	1781	1718	1692	1614
Cnot8	1079	1070	1158	1234	1230	1162	1137	1136
Cnot9	3343	3534	3057	3179	3287	3260	3162	2983
Cnp	1843	2180	1909	1747	2092	2077	1812	1808
Cnppd1	1715	1951	1899	1823	1824	1781	1727	1807
Cnpy1	379	330	346	360	312	382	361	295
Cnpy2	2453	2614	2420	2525	2587	2775	2702	2632
Cnpy3	950	967	912	971	894	925	868	863
Cnpy4	190	201	165	230	237	265	199	193
Cnr1	64	74	42	72	65	58	69	54
Cnr2	0	17	11	5	2	7	11	2
Cnrip1	37	38	34	31	18	16	25	24
Cnst	1296	1269	1283	1240	1317	1391	1411	1334
Cntd1	44	52	68	86	52	59	71	58
Cntf	34	51	36	39	66	55	30	54
Cntfr	10	9	14	22	16	12	9	6
Cntln	38	30	53	35	32	15	29	27
Cntn1	18	12	16	30	14	21	38	21
Cntn2	3	10	5	0	2	0	2	2
Cntn3	2	3	6	0	6	17	1	4
Cntn5	12	7	5	3	16	17	14	15
Cntnap1	168	178	156	164	268	243	311	272
Cntnap2	11	12	8	3	10	7	12	13
Cntnap3	2	4	1	2	12	0	1	0
Cntnap4	1	0	0	0	0	0	1	0
Cntnap5a	5	10	4	9	11	13	13	6
Cntrl	579	606	715	474	519	626	563	483
Cntrob	391	387	378	355	376	363	304	309
Coa3	3397	3233	3317	3179	3062	3289	2921	3256
Coa4	569	641	608	628	678	710	667	546
Coa5	7118	6865	6717	6996	6728	6694	6331	6065
Coa6	723	701	750	700	782	813	690	740
Coa7	1059	1107	876	1044	1011	1087	1020	1009
Coa8	576	623	649	683	577	618	556	541
Coasy	1266	1453	1355	1344	1428	1430	1328	1246
Cobl	6392	7370	6258	6253	7109	7498	8557	7852
Cobll1	1006	1000	987	992	895	913	928	910
Coch	0	1	1	4	2	4	2	1
Cog1	2683	2747	2910	2837	2655	2697	2644	2600
Cog2	1727	1733	1821	1772	1720	1666	1768	1530
Cog3	1459	1608	1540	1448	1449	1543	1471	1361
Cog4	3026	3195	2907	2844	3264	3169	2992	2973

Transcriptome sequencing yielded total genetic results for the MOD and APS groups, with a total of 15,936 variables

Continued from above

Cog5	1839	2395	2131	2111	2315	2552	2300	2114
Cog6	1852	1941	1800	1889	1931	2117	2077	1829
Cog7	1070	1130	997	969	1046	1138	995	923
Cog8	935	1016	1146	919	1048	972	1008	973
Coil	186	170	222	175	207	182	205	202
Col11a1	6	1	0	1	0	7	1	0
Col11a2	47	49	46	34	45	51	60	49
Col12a1	635	748	819	676	656	588	640	507
Col13a1	91	73	104	99	93	111	93	82
Col14a1	721	895	741	961	740	870	721	735
Col15a1	551	662	693	581	514	578	534	496
Col16a1	269	277	273	236	296	239	235	251
Col17a1	8	9	3	5	2	8	0	0
Col18a1	2098	2358	2449	2183	2198	2082	1949	1843
Col19a1	0	1	5	2	0	1	8	0
Col1a1	3619	3884	4023	3717	3602	3390	3148	3059
Col1a2	3723	4131	4179	3831	3815	3725	3078	3217
Col20a1	106	133	127	75	133	113	113	116
Col22a1	25	27	43	48	25	48	17	22
Col23a1	253	281	225	240	200	171	204	183
Col24a1	25	23	21	6	23	30	18	10
Col25a1	2	3	11	15	8	1	8	7
Col26a1	6	1	4	0	0	2	0	1
Col27a1	139	145	183	134	144	122	144	139
Col28a1	8	1	1	8	20	2	5	8
Col2a1	0	0	0	0	0	1	0	0
Col3a1	8154	9004	8938	8309	8848	8359	7869	6997
Col4a1	4937	5516	5568	5556	4809	4715	4083	4220
Col4a2	3332	3495	3651	3372	2934	2894	2767	2682
Col4a3	27	15	18	18	11	7	21	14
Col4a4	1	3	9	3	1	13	10	14
Col4a5	100	113	157	132	131	131	123	92
Col4a6	34	29	24	47	25	47	42	26
Col5a1	1187	1270	1309	1223	1269	1227	1171	999
Col5a2	912	1004	1066	1017	946	881	915	756
Col5a3	124	106	122	101	128	113	75	82
Col6a1	2930	3267	3116	3053	2868	2953	2434	2429
Col6a2	2641	2973	3134	2680	2700	2739	2323	2383
Col6a3	1613	1956	1954	1974	1746	1790	1494	1550
Col6a4	1545	1754	1677	1575	1393	1529	1331	1210
Col6a5	202	209	199	189	185	237	178	161
Col6a6	0	0	0	1	0	1	0	8
Col7a1	44	42	41	51	39	42	44	49
Col8a1	19	48	50	32	25	44	35	34
Col8a2	10	25	35	43	27	24	38	17
Col9a1	5	1	7	0	4	0	0	0
Col9a2	1	0	15	2	8	2	7	5
Col9a3	8	7	9	1	13	12	17	12
Colca2	92	37	42	104	54	32	45	34
Colec10	120	141	103	124	90	131	162	130
Colec11	5	2	4	7	1	6	1	3
Colec12	183	190	178	206	230	212	192	147
Colgalt1	1763	1897	1993	1918	2005	2055	1875	1822
Colgalt2	19	11	7	22	28	27	14	5
Colq	6	4	1	15	9	4	2	14
Commd1	1572	1864	1695	1652	1768	1803	1662	1600
Commd1(	606	776	635	740	510	557	506	534
Commd2	806	944	933	904	843	858	851	891

Transcriptome sequencing yielded total genetic results for the MOD and APS groups, with a total of 15,936 variables

Continued from above

Commd3	1441	1455	1523	1403	1544	1480	1642	1459
Commd4	827	910	1010	972	914	923	833	845
Commd5	907	845	899	838	690	790	750	775
Commd6	805	795	845	845	914	824	881	784
Commd7	1079	1163	1064	1143	1096	1193	1087	1090
Commd8	801	822	874	812	848	902	825	882
Commd9	818	904	849	851	947	848	792	769
Comp	3	0	7	7	5	1	3	7
Comt	4805	4932	4755	4836	4957	4786	4413	4517
Comtd1	1085	1143	1130	1135	1049	1075	1092	1029
Cop1	2232	2432	2299	2194	2405	2517	2352	2243
Copa	17840	20290	17599	16704	20009	19772	19970	18479
Copb1	9215	9911	9059	8980	9909	10374	10217	9279
Copb2	14548	16028	13852	14324	15824	16857	16353	14689
Cope	7194	7463	7389	7044	6929	7500	6718	6647
Copg1	10112	11097	10189	9494	10525	11014	10893	10077
Copg2	1932	1921	1927	1759	1991	1999	1946	1936
Coprs	327	315	374	353	326	351	340	288
Cops2	2263	2374	2103	2169	2331	2603	2573	2236
Cops3	2428	2463	2356	2427	2511	2540	2345	2305
Cops4	2024	2209	2067	1847	2161	2128	2085	2036
Cops5	2140	2486	2282	2325	2302	2360	2154	2182
Cops6	3095	3396	3009	3032	3042	3251	2883	3159
Cops7a	2789	3001	2833	3015	3297	2951	2802	2805
Cops7b	509	582	529	595	583	639	500	548
Cops8	1271	1379	1088	1194	1276	1088	1265	1233
Cops9	1460	1587	1437	1675	1431	1441	1472	1292
Copz1	9658	9944	9718	9197	10310	10329	10150	9664
Copz2	76	71	49	68	85	102	104	93
Coq10a	331	325	333	406	403	336	345	360
Coq10b	819	907	844	902	936	1095	1080	1036
Coq2	1516	1418	1453	1292	1634	1459	1400	1421
Coq3	420	587	535	488	625	622	526	534
Coq4	1238	1435	1300	1096	1493	1494	1435	1386
Coq5	2219	2369	2053	2253	2141	2229	2042	1860
Coq6	938	1023	1048	1005	1042	991	915	902
Coq7	964	1064	990	982	1081	1037	970	955
Coq8a	879	925	1038	993	822	970	797	767
Coq8b	940	969	869	825	867	988	1022	875
Coq9	3926	3995	3902	3935	4069	3937	3698	3704
Corin	0	0	0	0	0	1	0	0
Coro1a	906	865	764	781	821	953	731	727
Coro1b	14402	14785	13930	14333	14162	14528	13431	13429
Coro1c	11957	13202	11662	11359	12513	12451	12250	12038
Coro2a	21302	22752	21610	21147	21011	21693	21412	21066
Coro2b	42	78	36	69	40	47	36	29
Coro6	2	1	1	1	1	5	5	0
Coro7	2749	2672	2609	2630	2416	2273	2241	2533
Cort	0	0	0	0	0	0	4	0
Cotl1	4820	5726	5550	4804	6047	5689	5115	5375
Cox10	1956	1751	1802	1926	1610	1546	1553	1727
Cox11	1492	1531	1433	1554	1526	1513	1489	1644
Cox14	2904	2786	2898	2923	2600	2586	2550	2540
Cox15	2562	2646	2572	2625	2381	2346	2259	2287
Cox16	132	173	161	173	168	186	158	180
Cox17	2640	2497	2314	2500	2390	2414	2362	2200
Cox18	304	330	287	269	295	325	325	322
Cox19	1564	1571	1607	1438	1620	1629	1482	1597

Transcriptome sequencing yielded total genetic results for the MOD and APS groups, with a total of 15,936 variables

Continued from above

Cox20	687	658	679	749	679	781	717	711
Cox4i1	28576	27716	28623	29734	26187	27281	26758	26187
Cox4i2	6	19	13	17	8	21	11	26
Cox5a	14702	14714	14866	15510	13684	14577	13702	14266
Cox5b	11403	11548	11526	12470	10604	11061	10762	10540
Cox6a1	23260	22782	22755	23736	20744	21572	20606	20887
Cox6a2	12	10	12	10	7	13	3	5
Cox6b1	11870	12099	12358	12442	11589	12136	11364	11304
Cox6b2	40	64	67	76	56	71	52	81
Cox6c	11314	11261	11438	12249	10792	12105	11379	11248
Cox7a1	1883	1842	1677	1897	1568	1635	1633	1430
Cox7a2	8943	8852	8706	9264	8786	9172	8705	8634
Cox7a2l	7214	6951	7246	7524	7191	7303	7037	6960
Cox7b	12786	12621	12514	13498	11847	13075	12885	12411
Cox7c	11994	11811	12485	12241	11412	12376	11374	11414
Cox8a	16355	15777	16259	16380	14595	15350	14104	14441
Cox8b	8	4	1	0	1	5	1	2
Cp	313	501	337	376	376	315	271	276
Cpa1	2682	3864	1768	689	12490	11270	8431	11055
Cpa2	695	1085	487	229	3483	3328	2319	2978
Cpa3	2	2	0	0	1	7	1	4
Cpa6	0	1	4	1	1	3	1	0
Cpb1	3662	5161	2380	958	19348	17695	13119	16698
Cpb2	1	10	9	0	3	7	8	0
Cpd	4786	5405	4711	4915	5189	5104	5017	4862
Cpe	1246	1137	1056	1304	935	1043	1217	1078
Cpeb1	17	19	37	12	19	8	7	1
Cpeb2	1701	1744	1870	1694	1850	1975	1777	1619
Cpeb3	192	260	211	241	212	237	224	309
Cpeb4	3025	3107	2991	3048	3063	3421	3592	3097
Cped1	210	271	258	250	293	271	226	225
Cphx1	4	2	5	0	1	2	3	4
Cplane1	99	90	97	107	126	71	110	79
Cplane2	6	4	2	2	8	10	2	2
Cplx1	10	17	12	10	24	18	14	14
Cplx2	232	261	201	238	204	208	220	201
Cpm	281	326	296	313	343	398	323	335
Cpn1	86	80	70	70	103	75	75	88
Cpn2	481	559	447	404	452	432	448	431
Cpne1	971	1165	1129	983	1058	1094	1049	923
Cpne2	784	896	808	797	913	874	1028	847
Cpne3	3941	4528	4307	4472	4157	4173	4247	4001
Cpne4	4	18	2	17	20	25	10	16
Cpne5	8	4	1	2	0	1	1	1
Cpne6	0	0	0	1	0	0	0	0
Cpne7	21	3	3	9	5	4	8	12
Cpne8	32	22	39	24	26	15	29	43
Cpox	2916	2715	2779	3053	2859	3169	2997	2841
Cpped1	4473	4623	4749	4766	4470	4405	4119	4252
Cpq	869	993	925	953	1006	1053	850	760
Cps1	39586	46592	44152	41033	45585	42787	37761	38326
Cpsf1	2270	2547	2667	2224	2604	2425	2080	2191
Cpsf2	1860	2013	1799	1939	2056	1975	1907	1857
Cpsf3	1507	1823	1506	1503	1739	1693	1434	1491
Cpsf4	867	837	983	846	863	764	706	768
Cpsf4l	0	1	1	0	0	0	0	0
Cpsf6	1608	1693	1719	1730	2022	1995	1690	1562
Cpsf7	1429	1615	1627	1674	1620	1582	1588	1433

Transcriptome sequencing yielded total genetic results for the MOD and APS groups, with a total of 15,936 variables

Continued from above

Cpt1a	6002	6391	6493	6099	7081	6513	5912	6094
Cpt1b	140	170	222	206	228	212	225	243
Cpt1c	42	28	49	41	42	40	35	35
Cpt2	5797	6657	6164	5530	7269	6858	6364	6088
Cptp	2022	2054	1877	1899	1990	1984	1689	1895
Cpvl	4	3	9	3	7	1	1	1
Cpxm1	78	29	28	89	37	45	27	10
Cpxm2	101	134	160	122	94	92	87	128
Cpz	1	4	10	2	16	4	4	10
Cr1l	2490	2771	2540	2336	2514	2614	2427	2238
Cr2	7	0	0	0	0	1	0	0
Crabp1	2	3	11	1	8	1	11	13
Crabp2	6	0	0	0	0	0	0	0
Cracd	5407	5488	4788	4835	4920	5035	5532	5221
Cracdl	1540	1506	1496	1491	1551	1488	1429	1423
Cracr2a	284	367	356	377	324	238	263	269
Cracr2b	533	390	367	469	471	418	389	432
Cradd	261	249	256	252	229	186	208	205
Cramp1l	1128	1085	1108	1122	1146	1068	1042	1034
Crat	6079	6515	5946	5757	6081	6164	6063	5773
Crb1	0	0	0	0	0	3	0	0
Crb2	8	1	2	3	1	6	5	6
Crb3	2595	2522	2530	2522	2345	2548	2359	2390
Crbn	1849	2038	1721	1676	1844	2022	2057	1829
Crcp	631	751	719	668	736	716	635	652
Crct1	0	0	0	0	1	0	0	0
Creb1	1174	1244	1231	1190	1530	1488	1454	1232
Creb3	3167	3034	2724	2874	2588	2887	2794	2804
Creb3l1	698	654	739	829	718	595	617	750
Creb3l2	1714	1836	1556	1644	1724	1695	1910	1741
Creb3l3	17075	17881	16718	16471	16442	17216	17808	16532
Creb3l4	279	234	290	330	337	267	256	277
Creb5	33	30	46	63	19	34	46	45
Crebbp	1114	1295	1219	1236	1104	1205	1214	1133
Crebl2	476	577	570	494	724	733	683	653
Crebrf	1354	1539	1301	1262	1225	1374	1371	1216
Crebzf	1850	1856	1683	1649	1944	2031	1791	1925
Creg1	1554	1714	1712	1611	1764	1764	1559	1619
Creg2	17	9	11	12	14	10	2	4
Creld1	173	176	197	215	247	227	219	149
Creld2	1908	2087	1902	1770	2656	2543	2323	2236
Crem	196	246	149	168	149	163	118	163
Crh	0	0	0	1	0	0	0	0
Crhr2	3	1	5	1	1	0	1	1
Crim1	1482	1649	1252	1410	1292	1292	1604	1157
Crip1	25874	26489	25584	26571	22814	25709	27755	24753
Crip2	449	432	510	507	448	456	467	500
Crip3	0	0	3	0	0	1	1	0
Cript	2100	2205	1991	2044	2150	2221	2253	2197
Crisp2	2	1	1	0	0	0	1	3
Crispld1	4	2	9	8	4	7	3	0
Crispld2	352	398	412	360	349	341	252	288
Crk	6617	7130	6711	6691	7228	7686	7451	7115
Crkl	1911	2038	1890	1922	2047	2180	2121	2060
Crlf1	3	5	4	9	14	4	10	11
Crlf2	470	504	444	486	481	467	433	444
Crlf3	731	762	798	771	570	582	660	579
Crls1	1462	1533	1486	1359	1728	1740	1463	1547

Transcriptome sequencing yielded total genetic results for the MOD and APS groups, with a total of 15,936 variables

Continued from above

Crmp1	40	27	22	44	28	39	46	15
Crnkl1	818	944	885	867	939	1151	852	834
Crocc	97	127	149	148	113	147	84	112
Crocc2	1	8	1	2	0	2	0	0
Crot	6687	7772	6282	6011	8572	9198	8379	7486
Crp	3	13	13	5	7	6	3	1
Crppa	195	132	176	106	126	117	139	146
Crtac1	2	6	9	10	0	5	9	3
Crtam	5	11	6	4	7	3	1	2
Crtap	203	290	228	201	270	230	229	228
Crtc1	409	449	407	490	333	411	420	341
Crtc2	684	878	855	865	763	840	797	831
Crtc3	533	521	522	519	487	496	446	489
Cry1	711	834	714	728	937	970	923	799
Cry2	436	424	460	382	404	440	440	414
Cryaa	0	0	0	1	0	0	0	0
Cryab	160	154	163	148	193	196	206	210
Cryba2	34	1	15	3	15	6	16	10
Cryba4	8	23	14	10	24	11	9	7
Crybb1	5	4	4	0	0	0	1	4
Crybb3	59	49	63	75	56	51	37	42
Crybg1	3239	3615	3252	2823	3000	2999	2972	2627
Crybg2	3054	3090	3068	2997	3388	3403	3485	3140
Crybg3	427	467	582	478	393	502	453	481
Crygn	1	1	0	2	1	3	1	0
Cryl1	6335	6738	6265	6018	6204	6653	6397	6106
Crym	82	63	81	67	105	121	142	98
Cryz	34	33	38	14	36	35	27	25
Cryzl1	964	1094	1065	1231	1020	1013	968	1001
Cryzl2	126	117	99	122	152	154	124	149
Cs	49597	48716	45906	49157	44149	45784	46916	44787
Csad	379	422	438	437	470	390	350	371
Csdc2	8	9	9	14	9	24	19	10
Csde1	6416	6925	6719	6817	7252	7513	7520	6757
Cse1l	1819	2096	2013	1895	2281	2260	2038	1974
Csf1	435	414	444	407	397	335	365	291
Csf1r	2198	2082	1832	1967	1696	1791	1958	1784
Csf2	66	63	49	40	53	59	47	58
Csf2ra	459	428	432	406	433	402	405	392
Csf2rb	334	361	303	364	436	361	338	349
Csf2rb2	168	127	106	167	135	117	152	118
Csf3	0	0	0	1	0	0	0	0
Csf3r	55	72	58	34	61	62	49	26
Csgalnact	16	19	32	22	5	15	14	13
Csgalnact	274	295	212	235	251	322	283	196
Csk	3289	3500	3410	3317	3500	3534	3272	3373
Csl	2	11	5	8	5	2	1	8
Csmd1	4	5	7	2	2	8	7	1
Csmd2	4	0	0	2	0	1	1	1
Csmd3	0	0	0	1	0	0	0	0
Csn3	1	0	1	1	4	6	0	0
Csnk1a1	12667	13697	12330	12257	13466	13675	13425	12608
Csnk1d	6041	6191	6109	6001	5883	6359	5977	5862
Csnk1e	464	494	538	517	487	488	432	518
Csnk1g1	1434	1605	1430	1370	1432	1446	1503	1265
Csnk1g2	6092	6182	6228	6153	5896	5803	5678	5529
Csnk1g3	2442	2714	2569	2463	2649	2819	2886	2624
Csnk2a1	3627	3890	3991	3587	3649	3911	3846	3605

Transcriptome sequencing yielded total genetic results for the MOD and APS groups, with a total of 15,936 variables

Continued from above

Csnk2a2	1091	1110	1171	1114	1288	1137	1136	1013
Csnk2b	4279	4687	4270	4122	4237	4502	3979	4083
Cspg4	236	225	218	295	240	267	226	182
Cspg4b	8	4	4	10	6	10	6	1
Cspg5	7	8	17	11	5	10	11	5
Cspp1	300	295	278	323	324	348	366	296
Csprs	2	0	2	7	1	9	1	4
Csrnp1	307	315	311	310	186	215	245	228
Csrnp2	50	64	35	43	50	41	31	53
Csrnp3	5	2	4	5	4	2	5	0
Csrp1	4225	4708	4881	4876	4112	4149	4297	4216
Csrp2	1918	1944	1935	2103	2163	1958	1717	1997
Cst3	3624	3492	3501	3708	3759	3647	3457	3492
Cst6	2425	2614	2367	2320	2621	2772	2660	2260
Cst7	35	17	15	24	29	11	10	13
Csta2	10	17	9	6	12	11	29	29
Cstad	0	0	5	0	0	0	8	1
Cstb	1558	1525	1344	1455	1592	1534	1456	1501
Cstf1	729	862	787	661	794	712	747	680
Cstf2	1327	1575	1461	1311	1644	1629	1487	1354
Cstf2t	1373	1462	1324	1261	1227	1431	1454	1322
Cstf3	803	897	772	774	1010	945	806	923
Ct55	0	0	0	0	0	0	1	0
Ctbp1	7163	7684	7341	7115	7214	6958	6800	6869
Ctbp2	1687	1793	1740	1774	1840	1862	1750	1684
Ctbs	218	238	194	210	246	280	200	210
Ctc1	525	489	543	530	476	471	428	572
Ctcf	2116	2396	2172	2158	2281	2279	2274	2162
Ctcfl	0	0	0	4	0	0	1	2
Ctdnep1	3584	3626	3418	3351	3399	3396	3173	3338
Ctdp1	650	885	802	783	744	763	837	699
Ctdsp1	4806	4951	4731	4437	4375	4797	4263	4054
Ctdsp2	7946	8432	8132	8003	7218	7631	7766	7141
Ctdspl	1452	1595	1503	1301	1309	1412	1264	1323
Ctdspl2	621	624	646	632	591	610	561	578
Ctf1	23	30	29	27	22	15	44	22
Ctf2	1	0	0	0	0	0	0	0
Cth	2953	3570	3342	2984	3306	3394	3130	2968
Cthrc1	0	1	1	0	0	0	0	0
Ctif	270	292	326	285	278	325	285	294
Ctla2a	75	106	86	102	71	79	74	93
Ctla2b	25	29	28	12	20	16	30	22
Ctla4	11	15	6	24	16	6	16	23
Ctnna1	18541	19881	17822	17854	18211	18865	18681	18193
Ctnna2	13	3	7	12	16	9	2	3
Ctnna3	14	12	7	3	8	8	17	4
Ctnnal1	571	629	604	659	594	624	655	536
Ctnnb1	31347	33011	31079	31315	33510	34204	33601	31367
Ctnnbip1	1209	1205	1280	1238	1329	1438	1266	1263
Ctnnbl1	691	773	793	738	872	760	705	716
Ctnnd1	21445	23741	21191	20600	21798	22984	21805	20524
Ctnnd2	5	3	2	13	10	4	6	4
Ctns	714	770	689	760	646	687	576	661
Ctps	581	600	620	636	682	589	595	574
Ctps2	396	435	454	452	378	426	398	340
Ctr9	1427	1755	1803	1597	1858	1818	1649	1661
Ctrb1	10061	13069	6296	2984	48889	44628	36314	45821
Ctrl	1293	1757	762	385	5989	5279	3496	4502

Transcriptome sequencing yielded total genetic results for the MOD and APS groups, with a total of 15,936 variables

Continued from above

Ctsa	6276	6491	6162	6251	5834	5722	6027	5904
Ctsb	27833	30426	28358	26216	28658	28625	28048	26994
Ctsc	4931	5330	4730	4727	4798	4824	4735	4789
Ctsd	10824	11082	10503	11604	11551	11389	10994	11857
Ctse	40	41	31	41	43	33	30	33
Ctsf	187	141	146	142	139	219	214	149
Ctsh	4496	4585	4369	4467	4572	4568	4263	4532
Ctsk	87	90	107	73	71	74	81	63
Ctsl	2087	2312	1925	2207	2438	2292	2463	2399
Ctso	1307	1278	1147	1182	1165	1102	1167	1144
Ctss	7729	8529	7902	7861	8067	8357	8489	7383
Ctsw	80	78	89	73	59	77	71	55
Ctsz	16865	16178	15722	16286	16250	15919	16375	16710
Cttn	8942	9649	8940	8781	9092	9105	9082	8545
Cttnbp2	79	111	77	63	80	73	72	82
Cttnbp2nl	901	1033	1010	1116	979	1080	818	908
Ctu1	370	470	468	383	463	371	365	403
Ctu2	493	476	485	533	513	494	397	517
Ctxn1	70	59	63	83	100	72	48	59
Ctxn3	42	47	61	40	43	54	48	63
Cubn	66	97	43	43	235	237	157	90
Cuedc1	209	244	258	281	250	251	303	210
Cuedc2	620	472	530	552	495	472	447	503
Cul1	2983	3406	2859	3097	3169	3506	3481	3034
Cul2	1196	1413	1193	1174	1222	1279	1231	1191
Cul3	3188	3473	3333	3301	3324	3786	3540	3287
Cul4a	2596	2770	2596	2486	2719	2714	2460	2526
Cul4b	1034	1302	1050	1162	1183	1308	1213	1184
Cul5	1017	1076	980	1038	1082	1082	1143	1029
Cul7	250	279	235	222	262	251	224	228
Cul9	70	83	85	114	94	84	87	45
Cuta	1468	1480	1400	1520	1466	1477	1238	1364
Cutal	12	16	36	34	35	15	38	22
Cutc	416	434	410	415	425	440	414	409
Cux1	1800	1768	1937	1807	1790	1778	1731	1735
Cux2	48	40	44	38	41	35	33	52
Cuzd1	139	173	93	18	765	743	522	736
Cwc15	1943	2045	1879	1886	1865	1813	1690	1818
Cwc22	633	691	602	622	671	717	743	640
Cwc25	682	773	677	759	619	741	698	657
Cwc27	371	364	376	424	342	312	317	391
Cwf19l1	336	299	320	309	342	363	304	275
Cwf19l2	404	369	428	371	353	459	341	392
Cwh43	29	23	23	22	19	17	14	13
Cx3cl1	7130	7328	6436	6791	6204	6152	5940	5630
Cx3cr1	366	308	355	364	331	326	356	367
Cxadr	4748	5361	4774	4696	5663	5644	5647	5172
Cxcl1	26	23	21	5	25	18	3	19
Cxcl10	746	918	877	704	370	368	404	313
Cxcl12	710	662	684	689	625	708	566	554
Cxcl13	137	83	70	117	82	93	90	51
Cxcl14	743	602	601	686	567	550	465	512
Cxcl15	6	9	8	9	10	12	6	2
Cxcl16	1974	1976	1767	1837	1918	1795	2003	2053
Cxcl2	8	24	12	13	16	8	6	16
Cxcl3	0	2	6	10	0	8	1	2
Cxcl5	71	94	96	67	47	61	39	72
Cxcl9	2453	2572	2166	2193	1050	1214	1123	965

Transcriptome sequencing yielded total genetic results for the MOD and APS groups, with a total of 15,936 variables

Continued from above

Cxcr2	0	2	1	2	2	0	1	1
Cxcr3	36	26	27	20	14	30	19	38
Cxcr4	36	64	39	46	54	30	56	50
Cxcr5	3	9	2	6	10	8	1	1
Cxcr6	73	48	61	61	32	49	42	24
Cxxc1	1543	1581	1575	1571	1486	1527	1401	1456
Cxxc4	23	17	23	18	33	17	25	16
Cxxc5	112	95	128	144	152	134	119	138
Cyb561	4763	4550	4458	4460	4202	4380	4364	4186
Cyb561a3	1494	1777	1475	1413	1732	1687	1612	1717
Cyb561d1	874	957	958	927	932	885	869	900
Cyb561d2	507	455	446	493	513	575	411	451
Cyb5a	9162	9828	9619	9250	10791	11862	11457	11051
Cyb5b	32715	36450	34289	33083	36013	37572	36747	34233
Cyb5d1	768	703	714	668	843	793	730	844
Cyb5d2	230	278	185	149	284	324	257	198
Cyb5r1	390	389	402	433	426	442	406	388
Cyb5r2	1	0	0	0	0	4	0	2
Cyb5r3	20713	22555	21395	19355	19666	21752	20608	19957
Cyb5r4	3052	3455	3086	3048	3328	3459	3442	3174
Cyb5rl	84	105	76	79	84	116	92	79
Cyba	2652	2847	2718	2611	2325	2575	2342	2306
Cybb	758	856	699	797	641	683	595	583
Cybc1	894	884	919	955	1024	974	1004	928
Cybrd1	135	106	119	110	116	130	118	112
Cyc1	24439	24610	24552	24625	22074	22530	20852	22198
Cycs	17008	16654	15847	17323	15653	16052	16486	15792
Cyct	1	1	2	0	0	0	1	1
Cyfip1	5040	5261	4833	4669	4956	5204	4880	4849
Cyfip2	204	279	250	338	299	225	274	244
Cygb	515	574	510	511	457	463	423	321
Cyhr1	4667	4608	4595	4575	4627	4683	4398	4371
Cyld	2364	2705	2314	2133	2381	2467	2371	2008
Cym	76	95	96	44	92	121	108	94
Cyp11a1	4	7	7	6	6	5	10	1
Cyp11b1	0	1	4	0	0	0	0	0
Cyp11b2	0	0	0	0	0	0	0	2
Cyp17a1	0	1	1	2	0	0	0	1
Cyp1a1	11541	9285	7371	10129	36138	41081	38345	33497
Cyp1a2	64	70	60	45	34	92	93	43
Cyp1b1	217	198	238	215	285	278	239	255
Cyp20a1	310	406	367	358	435	304	308	288
Cyp26a1	0	1	8	1	0	4	4	4
Cyp26b1	271	240	387	333	340	258	253	324
Cyp26c1	0	1	2	0	4	0	0	0
Cyp27a1	998	1043	997	957	1025	1032	1207	1317
Cyp27b1	5	1	3	1	3	1	5	1
Cyp2a12	0	1	3	5	2	11	1	4
Cyp2a4	10	2	0	2	0	4	2	1
Cyp2a5	0	0	5	5	0	4	2	0
Cyp2b10	4966	5973	4739	3673	8107	9316	10078	8600
Cyp2b13	1	1	1	0	0	4	0	0
Cyp2b9	0	0	0	1	0	0	5	1
Cyp2c23	63	64	82	47	55	61	68	54
Cyp2c29	89	69	56	39	74	97	141	88
Cyp2c37	1	1	0	2	1	12	3	2
Cyp2c38	0	0	2	0	2	2	2	0
Cyp2c40	7	5	5	2	3	5	3	2

Transcriptome sequencing yielded total genetic results for the MOD and APS groups, with a total of 15,936 variables

Continued from above

Cyp2c50	1	1	2	4	3	4	2	2
Cyp2c54	0	0	0	1	3	3	0	1
Cyp2c55	612	667	282	224	1812	2157	1972	1194
Cyp2c65	6061	7275	5439	4607	9279	9824	8812	7631
Cyp2c66	454	581	358	219	1211	1260	1075	800
Cyp2c67	11	22	16	18	15	5	10	16
Cyp2c68	2035	2521	1921	1892	2426	2726	2561	2057
Cyp2c69	2	2	2	7	0	4	2	0
Cyp2c70	5	8	6	4	7	14	2	5
Cyp2d10	6	2	8	8	19	11	14	22
Cyp2d11	0	0	0	0	0	1	0	0
Cyp2d12	4	0	0	0	1	0	2	2
Cyp2d22	1298	1503	1190	1311	1424	1366	1676	1429
Cyp2d26	8138	9508	7393	6605	9734	9078	9333	8337
Cyp2d34	2	5	1	1	1	5	1	4
Cyp2d40	0	0	1	0	0	0	0	0
Cyp2d9	1	1	2	1	9	0	0	6
Cyp2e1	26	14	47	70	73	137	177	120
Cyp2f2	2	2	1	2	9	3	5	5
Cyp2j11	5	7	0	1	0	4	3	8
Cyp2j12	0	0	0	0	0	0	1	1
Cyp2j5	4	4	7	4	5	10	2	3
Cyp2j6	6767	7715	7480	6917	5969	6662	7004	6601
Cyp2j8	1	2	6	0	0	2	0	2
Cyp2j9	132	127	130	122	93	88	100	59
Cyp2r1	23	20	32	26	27	21	34	25
Cyp2s1	3159	3197	3278	3063	3415	3246	3052	3090
Cyp2t4	1	0	0	0	0	0	0	0
Cyp2u1	173	153	219	274	148	117	178	145
Cyp2w1	14	5	22	15	3	13	3	24
Cyp39a1	184	191	162	136	222	139	168	156
Cyp3a11	8490	9201	3593	2893	13551	17585	17417	13730
Cyp3a13	39293	45980	34886	31711	43851	47243	53220	47471
Cyp3a16	1	1	1	0	2	0	4	0
Cyp3a25	3027	3905	2144	1659	5715	6935	6483	4621
Cyp3a41a	0	0	2	0	0	0	12	4
Cyp3a41b	1	0	0	0	1	2	4	1
Cyp3a44	5	10	20	10	10	28	24	9
Cyp3a59	14	13	4	11	20	7	12	12
Cyp46a1	1	0	2	0	1	0	2	0
Cyp4a10	91	134	48	32	109	130	72	110
Cyp4a12a	0	1	0	0	1	11	8	2
Cyp4a12b	0	0	0	1	0	0	0	0
Cyp4a14	5	0	1	3	0	3	1	0
Cyp4a31	0	0	0	0	2	0	0	0
Cyp4a32	4	0	0	1	0	0	1	2
Cyp4b1	3389	3557	2591	2308	3143	2874	3613	3636
Cyp4f13	1313	1287	1156	1301	1185	1332	1225	1204
Cyp4f14	38255	41841	36047	33206	39612	40648	45552	42942
Cyp4f15	11	6	4	3	11	1	8	3
Cyp4f16	5994	7145	5676	4727	6324	6601	5924	5713
Cyp4f17	18	44	27	46	31	23	30	25
Cyp4f18	177	214	162	133	186	164	191	208
Cyp4f37	1	3	0	4	2	3	2	1
Cyp4f39	14	28	3	20	21	20	17	6
Cyp4f40	2163	2514	2200	1995	2548	2435	2234	2394
Cyp4v3	7920	9129	7546	6682	8008	8869	10128	7990
Cyp4x1	0	1	0	4	0	5	0	0

Transcriptome sequencing yielded total genetic results for the MOD and APS groups, with a total of 15,936 variables

Continued from above

Cyp51	4248	4574	5003	4861	4692	4550	4420	4626
Cyp7b1	43	23	66	49	49	28	53	37
Cyp8b1	1	0	0	0	4	0	0	0
Cypt1	0	2	0	1	1	5	0	0
Cyren	156	168	143	149	149	144	198	183
Cyria	177	137	156	105	127	203	116	143
Cyrib	1761	1810	1851	2035	1517	1695	1613	1402
Cys1	16	20	7	14	14	18	18	17
Cysltr1	70	77	93	111	79	96	89	56
Cysltr2	40	52	42	37	46	26	18	45
Cysrt1	0	1	0	1	4	0	0	0
Cystm1	14689	14814	14256	13969	13396	13921	14686	14032
Cyth1	1971	1793	1945	2100	1739	1821	1834	1734
Cyth2	1901	1795	1815	1862	1650	1652	1686	1845
Cyth3	570	539	674	652	557	579	564	485
Cyth4	162	102	175	150	151	146	109	123
Cytip	260	366	301	305	313	372	328	298
Cytl1	12	16	16	11	12	25	15	18
Cyyr1	108	116	152	151	102	115	85	100
Czib	423	454	550	484	537	535	473	428
D030056L	271	358	387	340	378	379	265	327
D10Wsu1(	690	865	726	698	808	810	724	702
D11Wsu47	317	302	259	296	328	336	320	310
D130037M	201	203	188	195	168	187	144	126
D130040H	12	15	16	12	9	14	22	7
D130043K	779	807	713	560	899	845	934	759
D16Ertd47	102	129	113	111	158	147	140	105
D17H6S53	1484	1516	1421	1456	1535	1560	1474	1496
D17H6S56	5205	6320	7041	5941	6616	6662	5987	6164
D230025D	524	513	545	427	470	498	501	509
D2hgdh	617	624	562	663	670	746	601	614
D3Ertd751	139	171	163	129	215	186	189	171
D430019H	22	25	32	34	46	21	23	17
D5Ertd579	7115	7549	6424	6541	6365	6988	7788	7090
D630003M	36	61	25	31	28	37	34	46
D630023F	11	16	4	8	6	15	4	5
D630039A	1570	1484	1640	1679	1135	1125	1066	1103
D630045J1	10	17	11	11	10	12	7	10
D6Ertd527	26	21	18	9	17	16	28	15
D6Wsu163	444	505	453	404	523	524	413	531
D830030K	1	11	5	2	7	5	16	22
D830031N	48	110	124	128	79	103	93	60
D8Ertd738	1654	1699	1774	1786	1805	1669	1650	1614
D930020B	6	11	3	11	5	7	5	2
DXBay18	4	0	0	0	0	0	0	0
Daam1	1539	1676	1433	1473	1511	1547	1644	1623
Daam2	206	220	197	254	253	226	184	180
Dab1	2698	2719	2344	2400	2169	2407	2549	2246
Dab2	209	284	249	248	266	250	216	257
Dab2ip	5727	5930	5497	5744	5877	5773	5629	5408
Dach1	234	242	293	239	169	227	159	170
Dach2	2	2	0	2	3	7	5	0
Dact1	168	157	165	141	113	124	135	115
Dact2	739	659	665	727	662	777	768	707
Dact3	190	158	212	197	143	151	120	136
Dad1	4778	5040	4838	4870	4935	4989	4618	5004
Dag1	10992	11647	11267	11267	11320	10783	10480	10944
Dagla	281	309	291	249	282	347	247	276

Transcriptome sequencing yielded total genetic results for the MOD and APS groups, with a total of 15,936 variables

Continued from above

Daglb	1353	1514	1357	1274	1453	1517	1372	1318
Dalrd3	1223	1488	1424	1138	1505	1238	1278	1167
Dand5	63	41	73	51	72	64	70	51
Dao	441	552	469	436	413	523	603	569
Dap	2425	2563	2465	2316	2416	2408	2428	2393
Dap3	1905	2136	2041	2019	2208	2197	1823	2020
Dapk1	103	109	103	131	138	172	115	134
Dapk2	1174	1046	1134	1070	1001	1016	832	987
Dapk3	2015	1867	1829	1815	2000	1976	1798	1943
Dapp1	1389	1546	1538	1311	1707	1709	1570	1455
Dars	1854	2184	1983	2004	2175	2405	2078	1961
Dars2	1032	1047	1122	932	1095	1026	974	1073
Daw1	0	0	0	0	0	0	1	0
Daxx	1640	2029	1756	1516	1487	1579	1557	1562
Dazap1	3268	3101	3267	3023	3203	3074	2921	2994
Dazap2	13291	13745	13166	13454	12736	13063	13116	12312
Dazl	1	0	0	0	0	0	0	0
Dbf4	540	617	577	574	688	560	551	578
Dbh	10	11	2	10	0	6	2	13
Dbi	15782	14576	15484	17609	14936	16063	15775	15943
Dbil5	0	0	2	0	1	0	6	2
Dbn1	146	201	143	157	203	190	190	123
Dbndd1	8	2	2	7	1	1	4	5
Dbndd2	1336	1521	1383	1374	1446	1508	1346	1403
Dbnl	7580	7716	6920	6884	7074	6599	6643	6284
Dbp	609	417	511	729	431	484	521	531
Dbpht2	1	6	1	1	1	10	4	2
Dbr1	642	653	665	553	617	577	540	545
Dbt	908	1059	945	925	899	1018	982	989
Dcaf1	1311	1505	1487	1468	1535	1415	1536	1458
Dcaf10	948	1054	942	1015	991	1051	858	950
Dcaf11	5832	6348	6047	5780	6096	5916	5752	5469
Dcaf12	3527	3921	3297	3220	3955	3998	3565	3664
Dcaf12l1	10	4	5	5	1	7	6	9
Dcaf13	1255	1555	1317	1454	1441	1477	1327	1385
Dcaf15	2084	2109	2033	1948	1880	2009	1744	1666
Dcaf17	510	565	546	521	632	548	599	529
Dcaf4	280	264	283	255	279	258	195	259
Dcaf5	1532	1741	1562	1606	1480	1472	1412	1258
Dcaf6	631	612	621	570	519	539	535	533
Dcaf7	5079	6004	4972	4937	5198	5317	5022	4634
Dcaf8	2923	2887	3037	2907	2628	2615	2392	2487
Dcakd	807	859	792	701	751	759	630	777
Dcbld1	2219	2470	2323	2228	2500	2346	2321	2155
Dcbld2	148	186	129	135	129	162	113	115
Dcc	1	0	0	0	0	0	0	0
Dcdc2a	1	3	7	4	4	6	11	9
Dcdc2b	29	24	12	14	17	23	29	12
Dcdc5	0	0	0	0	0	0	0	4
Dchs1	231	250	254	264	211	228	151	261
Dchs2	3	1	0	11	3	0	13	6
Dck	627	707	698	643	730	609	631	673
Dclk1	113	122	138	127	118	111	96	109
Dclk2	35	34	72	46	22	29	54	43
Dclk3	33	38	21	22	30	41	29	47
Dclre1a	319	325	338	295	363	354	275	323
Dclre1b	340	499	468	517	408	476	382	418
Dclre1c	259	262	292	288	330	298	299	306

Transcriptome sequencing yielded total genetic results for the MOD and APS groups, with a total of 15,936 variables

Continued from above

Dcn	3300	3484	3391	3594	3811	3976	3456	3002
Dcp1a	1014	1147	1102	1190	1133	1055	1099	974
Dcp1b	202	276	209	231	255	193	150	191
Dcp2	1844	1962	2138	2012	1902	2189	1946	1805
Dcpp1	0	4	1	0	0	0	0	0
Dcps	589	663	616	466	556	488	467	441
Dcst1	192	190	215	168	202	194	185	201
Dcst2	71	66	76	69	54	39	78	74
Dcstamp	0	0	0	0	0	0	0	4
Dct	1	2	2	1	1	0	1	5
Dctd	227	238	198	160	276	247	182	201
Dctn1	6365	6301	6047	5822	6363	6389	6333	5881
Dctn2	5536	5873	5436	5459	5683	5398	5339	5230
Dctn3	1648	1688	1680	1634	1542	1619	1601	1550
Dctn4	1455	1731	1533	1584	1763	1714	1615	1558
Dctn5	1662	1604	1642	1663	1610	1650	1575	1702
Dctn6	1489	1532	1529	1458	1350	1456	1332	1359
Dctpp1	739	664	762	714	834	734	658	576
Dcun1d1	1687	1749	1557	1617	1793	1804	2008	1859
Dcun1d2	510	531	543	556	534	621	518	466
Dcun1d3	383	424	424	493	481	486	460	371
Dcun1d4	951	1056	918	884	820	981	1003	822
Dcun1d5	1238	1268	1260	1339	1475	1430	1453	1364
Dcx	1	1	0	6	0	0	2	4
Dcxr	869	991	909	888	860	857	847	866
Dda1	2169	2263	2297	2254	2332	2283	2127	2133
Ddah1	276	276	228	208	308	260	321	371
Ddah2	145	134	184	130	122	164	108	130
Ddb1	14221	15559	14766	14063	15482	15436	15069	14697
Ddb2	137	182	172	163	163	127	106	162
Ddc	6499	7726	7177	5789	8574	8637	7908	7321
Ddhd1	1761	1985	1738	1838	2011	2037	1878	1863
Ddhd2	3193	3972	3710	3247	4260	4752	4779	4175
Ddi2	1387	1689	1512	1369	1741	1691	1576	1394
Ddias	211	152	133	130	177	183	116	149
Ddit3	521	578	434	509	535	540	438	463
Ddit4	866	1171	1502	1231	906	937	863	991
Ddit4l	18	17	10	6	33	36	25	32
Ddn	0	0	2	1	0	1	7	1
Ddo	16	13	9	20	25	20	29	8
Ddost	8528	9278	8652	8162	9818	9203	7885	8628
Ddr1	6053	5998	5441	5320	4941	5236	5162	4569
Ddr2	368	422	318	342	335	378	432	293
Ddrgk1	2185	2147	2271	2102	2121	2190	2075	2095
Ddt	1479	1317	1422	1512	1423	1512	1314	1501
Ddx1	2900	3315	3242	3074	3308	3452	2963	2938
Ddx10	571	578	428	483	577	580	559	502
Ddx11	248	218	257	223	246	197	164	201
Ddx17	8580	9338	9102	8900	9564	9966	8834	8438
Ddx18	944	1010	947	834	1121	1000	1014	1002
Ddx19a	877	1009	985	972	1003	1004	938	919
Ddx19b	778	888	907	890	869	804	766	789
Ddx20	522	600	580	527	607	569	600	657
Ddx21	2659	3202	2920	2575	3136	3246	2716	2846
Ddx23	2081	2330	2207	2220	1984	2237	2107	1958
Ddx24	2856	3175	2874	2523	2781	2534	2530	2568
Ddx25	6	0	0	0	5	1	1	7
Ddx27	852	994	906	942	1105	967	986	905

Transcriptome sequencing yielded total genetic results for the MOD and APS groups, with a total of 15,936 variables

Continued from above

Ddx28	297	377	376	313	421	411	380	380
Ddx31	151	205	159	184	194	195	136	205
Ddx39a	2709	2997	2717	2766	2951	2552	2478	2596
Ddx39b	3496	3477	3478	3329	3113	3220	2770	3013
Ddx3x	15884	17765	17333	17145	17718	18891	18762	15925
Ddx3y	920	528	0	338	245	323	744	897
Ddx4	0	0	1	1	0	1	4	2
Ddx41	965	1096	1205	1134	1302	1076	1167	1091
Ddx42	2307	2241	2263	2249	1909	2230	2182	2033
Ddx43	1	6	1	0	1	7	0	5
Ddx46	2071	2197	2603	2383	1824	2008	2036	1855
Ddx47	1282	1411	1374	1359	1521	1489	1326	1439
Ddx49	2617	2688	2694	2547	2747	2642	2475	2409
Ddx5	22010	23687	22922	22122	21468	22902	23185	20240
Ddx50	1306	1369	1379	1258	1482	1488	1479	1308
Ddx51	399	372	391	357	388	410	355	419
Ddx52	1050	1080	991	963	1092	1007	1057	1072
Ddx54	2951	2916	2928	2922	2883	3111	2925	2871
Ddx55	248	260	283	266	269	222	243	234
Ddx56	1104	1109	1076	980	1014	1022	970	863
Ddx58	3967	5033	4322	3554	4352	4162	4049	3629
Ddx59	62	83	81	73	70	87	72	40
Ddx6	6248	6804	6955	6722	6897	7045	6657	6358
Ddx60	7970	10281	7880	5807	9124	9265	9067	7628
Deaf1	755	764	733	650	870	829	738	734
Decr1	5501	5608	5402	5333	5678	5934	5654	4973
Decr2	2225	2432	2157	2395	2311	2592	2384	2244
Dedd	1327	1458	1291	1235	1462	1464	1439	1328
Dedd2	2236	2373	2086	2293	2163	2267	2103	2105
Def6	76	69	53	47	72	99	50	64
Def8	812	850	837	865	798	832	775	768
Defa17	332	346	310	299	275	261	339	298
Defa2	16	13	33	34	16	21	10	41
Defa20	349	266	273	294	506	185	242	342
Defa21	1137	1123	1132	1315	687	782	1169	1055
Defa22	1465	1270	1321	1540	758	1004	1347	1232
Defa23	156	114	104	804	63	72	136	143
Defa24	21268	18897	18120	22473	15261	17715	21423	21102
Defa26	183	174	202	228	172	159	220	233
Defa27	2	0	1	1	0	0	0	1
Defa28	2	0	5	1	0	1	1	2
Defa29	1395	1188	1184	1344	821	919	1230	1069
Defa3	151	128	169	110	159	114	136	112
Defa30	8623	6921	7390	9279	6435	6989	8752	8244
Defa31	27	22	27	23	15	14	40	44
Defa32	16	22	24	71	8	10	26	15
Defa33	1	0	14	0	1	1	2	0
Defa34	1091	984	1084	1255	771	811	993	1153
Defa35	50	47	46	61	25	32	51	46
Defa36	51	67	42	38	41	37	28	377
Defa37	49	25	28	53	18	31	34	38
Defa38	7200	5610	4868	7817	5136	5709	6866	7283
Defa39	8697	7686	7243	8177	5878	6662	8569	8385
Defa40	71	85	55	85	62	73	62	136
Defa41	58	81	76	77	49	76	65	63
Defa42	82	65	89	149	56	63	109	95
Defa43	25	19	22	37	15	18	8	20
Defa5	337	340	301	484	234	283	324	408

Transcriptome sequencing yielded total genetic results for the MOD and APS groups, with a total of 15,936 variables

Continued from above

Defb1	1	0	4	1	4	0	0	1
Defb30	0	0	0	1	0	0	0	0
Defb37	0	0	0	0	0	1	1	1
Defb47	4	0	0	0	0	0	0	0
Degs1	1122	1281	1128	1223	1192	1215	1133	1099
Degs2	5429	5558	5488	5368	5007	5370	5117	5375
Dek	1780	2035	1920	1936	2087	2018	1905	1792
Dele1	1144	1191	1050	1146	1214	1190	1099	1142
Denn2b	1868	2019	2159	1923	2145	2110	1982	1725
Dennd10	805	950	839	799	795	859	831	885
Dennd11	799	902	928	1009	930	897	945	753
Dennd1a	442	518	448	551	420	489	428	403
Dennd1b	2044	2234	1871	1950	2109	2051	2320	2049
Dennd1c	105	139	105	74	88	96	108	115
Dennd2a	91	133	97	86	142	149	147	101
Dennd2c	15	15	18	16	10	20	21	18
Dennd2d	2492	2732	2682	2717	2489	2462	2457	2248
Dennd3	1788	1769	1538	1675	1512	1649	1681	1542
Dennd4a	188	189	184	193	183	197	162	180
Dennd4b	124	102	135	119	122	105	151	126
Dennd4c	2053	2151	2054	2202	2307	2422	2278	2032
Dennd5a	430	568	556	518	557	567	471	464
Dennd5b	1616	2069	1755	1724	2114	2278	2285	1872
Dennd6a	2144	2119	2102	2216	2063	2004	2331	2074
Dennd6b	221	206	198	171	173	127	140	123
Denr	986	1081	1069	922	1131	983	825	943
Depdc1a	120	153	83	122	175	150	115	141
Depdc1b	112	145	134	90	98	91	115	111
Depdc5	628	597	685	599	531	549	515	522
Depdc7	742	864	766	653	686	812	840	821
Depp1	46	51	93	68	58	35	51	46
Deptor	1511	1685	1796	1853	1741	1874	1859	1751
Dera	4727	4845	4534	4362	4452	4497	4399	4466
Derl1	6506	6871	6180	6537	7042	7098	6695	6466
Derl2	2291	2312	2272	2093	2524	2520	2263	2335
Derl3	181	177	153	151	183	140	147	151
Des	4314	4340	5482	5375	4427	4584	4918	4737
Desi1	2578	2396	2487	2617	2280	2469	2188	2001
Desi2	1799	1944	1910	2012	2241	2082	2049	1880
Det1	422	447	391	425	441	394	431	397
Deup1	0	0	0	0	1	0	2	0
Dexi	347	316	308	341	370	306	270	327
Dffa	1391	1443	1329	1310	1352	1328	1238	1400
Dffb	593	651	716	639	654	590	605	552
Dgat1	28174	29434	26450	26020	27387	27948	26898	26232
Dgat2	12686	14964	14556	12199	16688	17282	19367	18328
Dgcr2	4023	4372	4353	4095	4279	4313	3900	3899
Dgcr6	1651	1547	1756	1683	1534	1611	1557	1722
Dgcr8	694	758	836	832	843	819	789	744
Dgka	5246	5232	5207	5566	4482	4936	5114	4658
Dgkb	47	51	27	43	52	36	57	54
Dgkd	6734	6070	6658	7415	6437	6850	6378	6126
Dgke	192	167	184	205	168	188	185	195
Dgkg	52	50	62	69	59	49	25	63
Dgkh	138	180	162	138	151	222	185	179
Dgki	26	15	29	29	27	39	15	18
Dgkk	0	0	1	0	0	0	0	0
Dgkq	4205	4435	4137	4137	3830	4032	4125	3941

Transcriptome sequencing yielded total genetic results for the MOD and APS groups, with a total of 15,936 variables

Continued from above

Dgkz	7520	7459	7263	6864	7482	7342	6685	6567
Dglucy	3848	4117	3776	3576	4161	4190	4094	3902
Dguok	328	416	395	366	379	424	337	348
Dhcr24	7063	7148	7696	7789	7474	7683	7399	7250
Dhcr7	1826	1969	2024	2053	1968	1962	1777	1645
Dhdds	1222	1167	1258	1107	1305	1329	1168	1204
Dhdh	26	14	17	54	32	48	36	37
Dhfr	1661	1668	1485	1611	1385	1600	1551	1413
Dhh	16	16	17	26	8	16	18	26
Dhodh	390	461	399	320	400	430	333	411
Dhps	814	877	794	766	730	815	708	751
Dhrs1	14134	14891	12707	12642	14234	14709	14691	13965
Dhrs11	9406	9671	9293	9172	8898	9314	9201	8736
Dhrs13	136	134	130	182	160	133	126	132
Dhrs2	0	0	0	0	0	0	1	0
Dhrs3	1346	1216	1310	1295	1288	1204	1028	1174
Dhrs4	3187	3401	3516	3162	3933	3905	3439	3175
Dhrs7	890	953	996	876	924	933	760	844
Dhrs7b	2188	2197	2020	2303	2415	2371	2364	2395
Dhrs9	73	82	78	101	146	158	103	135
Dhrsx	1928	1844	1989	1942	1954	1841	1869	1845
Dhtkd1	8	12	19	5	9	2	1	1
Dhx15	3996	4416	4360	4205	4688	4777	4259	4067
Dhx16	1918	2199	2063	1879	2073	2057	1883	1821
Dhx29	495	505	450	521	617	558	545	489
Dhx30	1288	1332	1223	1196	1400	1293	1212	1323
Dhx32	2193	2337	2289	2549	2282	2314	2001	2068
Dhx33	536	627	668	494	599	676	614	615
Dhx34	414	463	406	438	438	442	417	465
Dhx35	878	937	919	1002	733	833	824	752
Dhx36	1358	1468	1249	1321	1473	1298	1356	1192
Dhx37	460	575	505	476	541	556	464	520
Dhx38	2667	2773	2540	2522	2523	2495	2387	2392
Dhx40	1064	1226	1223	1031	1128	1196	1254	1080
Dhx57	748	771	743	746	809	754	865	671
Dhx58	1794	2779	2398	1638	2183	2202	1843	1912
Dhx8	1428	1587	1388	1427	1439	1445	1383	1378
Dhx9	4902	5874	5052	4948	5252	5781	5218	4710
Diablo	885	981	989	1031	1107	1124	1063	1080
Diaph1	5315	6050	5601	5604	5593	5695	5531	5257
Diaph2	222	242	224	263	220	252	194	203
Diaph3	229	247	206	193	280	240	224	244
Dicer1	1873	2332	2202	2149	2217	2254	2109	1871
Dido1	2406	2425	2650	2578	2899	2919	2662	2525
Dimt1	191	186	159	188	176	179	125	169
Dio1	657	790	546	486	892	1144	1116	777
Dio2	4	14	5	9	6	4	6	11
Dio3	0	1	0	0	0	0	0	0
Dip2a	1528	1516	1538	1751	1412	1452	1341	1463
Dip2b	1278	1419	1373	1333	1293	1374	1412	1262
Dip2c	296	261	308	299	278	267	303	307
Dipk1a	267	307	245	276	263	219	232	206
Dipk1b	69	56	37	68	60	49	32	33
Dipk1c	1	9	6	6	6	5	9	3
Dipk2a	600	779	621	490	851	899	878	694
Dipk2b	8	16	4	11	4	15	3	8
Diras1	4	1	10	1	2	5	4	5
Diras2	7	23	11	12	31	19	8	10

Transcriptome sequencing yielded total genetic results for the MOD and APS groups, with a total of 15,936 variables

Continued from above

Dis3	554	684	529	545	628	611	621	537
Dis3l	662	761	680	597	706	695	640	718
Dis3l2	605	577	597	599	539	575	610	594
Disc1	11	13	13	12	7	9	22	16
Disp1	383	323	317	332	292	262	267	353
Disp2	107	133	148	158	143	145	185	138
Disp3	1	2	1	1	3	2	0	0
Dixdc1	62	33	49	50	75	79	54	48
Dkc1	746	947	766	717	912	835	756	711
Dkk2	8	16	14	8	25	19	20	22
Dkk3	567	660	639	620	631	631	501	538
Dkk4	11	15	2	5	4	2	7	10
Dkkl1	5	3	22	8	9	11	1	6
Dlat	5248	5078	5272	5428	5058	5119	4831	4888
Dlc1	322	312	445	359	378	343	373	314
Dld	9091	9543	8822	9142	9148	9958	9695	9257
Dlec1	1	6	3	0	12	5	2	8
Dleu7	9	5	1	13	0	3	10	2
Dlg1	2510	2769	2494	2542	2630	2916	2548	2376
Dlg2	36	31	22	34	30	25	33	28
Dlg3	3543	3467	3319	3431	3178	3383	3527	3489
Dlg4	108	85	59	76	47	66	82	53
Dlg5	829	892	981	924	917	973	850	793
Dlgap1	147	189	78	90	249	219	223	249
Dlgap2	4	2	1	0	1	0	0	1
Dlgap3	2	9	9	5	13	2	8	10
Dlgap4	871	906	941	1097	1016	916	781	865
Dlgap5	320	415	364	317	465	407	307	432
Dlk1	0	5	0	0	0	2	0	6
Dlk2	0	0	0	0	8	1	0	0
Dll1	1328	1645	1539	1368	1613	1505	1572	1437
Dll3	3	4	20	12	13	4	17	13
Dll4	807	913	836	784	807	839	906	809
Dlst	21059	21735	20376	20679	19439	20597	21331	19608
Dlx1	1	1	7	13	0	2	1	2
Dlx2	1	1	0	1	1	1	0	7
Dlx3	4	4	0	4	0	0	1	0
Dlx4	0	0	0	0	0	0	1	0
Dmac1	578	558	665	636	658	690	495	613
Dmac2	1725	1648	1735	1790	1576	1634	1494	1507
Dmac2l	324	370	329	309	356	339	331	334
Dmap1	472	454	508	399	418	416	352	371
Dmbt1	225215	269132	259069	235199	287729	280683	261966	267828
Dmbx1	0	0	0	1	0	0	4	0
Dmc1	1	2	0	0	1	0	0	1
Dmd	428	467	508	442	460	444	417	429
Dmgdh	3	0	3	5	5	3	4	1
Dmkn	8	9	8	5	4	6	18	15
Dmp1	23	30	49	28	15	8	26	17
Dmpk	1318	1335	1641	1529	1244	1486	1493	1268
Dmrt1	0	0	0	1	0	0	0	1
Dmrt2	1	1	1	0	0	0	0	0
Dmrt3	1	0	1	0	3	9	6	2
Dmrta1	0	4	0	0	0	0	0	0
Dmrta2	4	2	8	14	6	8	5	10
Dmtf1	712	861	674	698	786	785	692	764
Dmtn	43	45	28	54	39	39	34	45
Dmwd	162	142	120	172	142	131	173	149

Transcriptome sequencing yielded total genetic results for the MOD and APS groups, with a total of 15,936 variables

Continued from above

Dmxl1	2015	2108	2157	2237	2423	2356	2567	1966
Dmxl2	66	87	106	92	120	117	126	111
Dna2	411	499	447	434	486	442	373	379
Dnaaf1	232	337	299	319	285	327	276	309
Dnaaf2	229	306	252	232	258	280	243	256
Dnaaf3	23	37	46	21	10	18	7	13
Dnaaf4	0	0	1	3	1	1	0	1
Dnaaf5	655	544	509	582	665	604	643	640
Dnah1	5	4	2	2	1	1	4	0
Dnah10	17	24	6	15	25	40	17	51
Dnah11	14	11	11	2	11	12	17	5
Dnah12	2	5	11	8	7	3	6	6
Dnah14	0	0	1	0	0	0	0	0
Dnah17	105	137	54	108	101	112	118	110
Dnah2	492	554	542	434	590	736	766	795
Dnah3	0	0	0	1	0	1	0	0
Dnah5	1	1	0	4	10	2	0	6
Dnah6	2	5	1	1	4	1	1	1
Dnah7a	0	1	1	0	0	0	4	0
Dnah7b	4	7	6	12	7	1	3	7
Dnah7c	1	0	1	0	0	0	0	0
Dnah8	570	589	523	637	550	591	505	492
Dnah9	11	22	9	16	12	10	18	12
Dnaic1	8	8	17	9	3	12	7	10
Dnaic2	13	16	8	18	6	17	7	3
Dnaja1	6840	7232	6521	6510	11524	11243	9460	8816
Dnaja2	6985	7388	6724	6575	7979	8105	7548	7183
Dnaja3	2988	3122	3153	2911	3517	3342	2710	2962
Dnaja4	167	177	151	183	289	211	208	181
Dnajb1	1887	1923	2049	2002	2324	2265	1998	2014
Dnajb11	4522	5192	4438	4352	5419	5553	4836	4954
Dnajb12	2626	2578	2624	2503	2473	2788	2551	2646
Dnajb13	6	3	0	4	4	4	5	7
Dnajb14	841	919	792	886	915	825	975	822
Dnajb2	442	443	460	408	612	601	536	556
Dnajb3	88	102	76	73	60	57	58	60
Dnajb4	519	620	464	469	693	751	633	615
Dnajb5	264	213	258	263	219	230	246	262
Dnajb6	2236	2395	1975	1951	2374	2403	2386	2306
Dnajb7	2	3	10	2	8	1	10	8
Dnajb9	2069	2123	1942	2010	2321	2160	2308	2344
Dnajc1	629	742	688	603	858	840	808	715
Dnajc10	3097	3339	3138	3244	3500	3542	3106	3160
Dnajc11	3120	3194	3030	3127	3100	3042	2726	2777
Dnajc12	39	56	46	44	50	30	74	50
Dnajc13	6158	6558	6072	6219	6404	6405	6500	5925
Dnajc14	2609	2807	2748	2580	2601	2873	2745	2633
Dnajc15	1005	1038	946	1022	967	1031	911	941
Dnajc16	1017	976	1091	1004	980	952	861	885
Dnajc17	276	241	232	252	255	256	215	233
Dnajc18	104	148	103	137	130	164	132	149
Dnajc19	1396	1434	1383	1466	1362	1420	1331	1355
Dnajc2	993	993	846	885	1026	1004	1025	925
Dnajc21	637	627	594	617	737	667	631	606
Dnajc22	2608	2908	2758	2778	2776	2987	2616	2598
Dnajc24	297	284	307	226	249	286	221	298
Dnajc25	580	644	601	668	701	577	690	602
Dnajc27	82	102	100	81	92	101	86	55

Transcriptome sequencing yielded total genetic results for the MOD and APS groups, with a total of 15,936 variables

Continued from above

Dnajc28	61	60	68	62	97	67	97	83
Dnajc3	5150	5657	4830	5198	6811	6701	6459	5785
Dnajc30	1365	1404	1326	1387	1249	1283	1181	1243
Dnajc4	574	558	607	610	588	519	567	533
Dnajc5	3908	4061	4173	4183	3943	4112	4060	3815
Dnajc6	23	7	11	23	13	28	4	15
Dnajc7	4613	4873	4724	4872	4807	4845	4589	4277
Dnajc8	2175	2214	2176	2136	2082	2009	1903	1884
Dnajc9	629	749	739	651	801	748	680	556
Dnal1	21	37	64	35	21	39	48	58
Dnal4	365	424	366	420	361	427	345	387
Dnali1	0	0	0	0	0	0	1	0
Dnase1	9633	11055	9668	8290	8663	10017	9989	7891
Dnase1l1	905	681	694	676	704	723	769	729
Dnase1l2	13	21	16	16	30	17	23	22
Dnase1l3	910	785	847	905	671	730	759	713
Dnase2a	125	147	126	111	56	100	72	122
Dnase2b	1	0	0	0	0	0	0	0
Dnd1	14	19	13	10	3	3	6	13
Dner	15	26	24	52	20	18	29	23
Dnhd1	66	53	57	70	70	64	43	51
Dnlz	723	927	825	827	854	883	795	805
Dnm1	5539	5618	5527	5664	4816	5222	4790	4640
Dnm1l	3846	4031	3623	4070	4002	4005	3935	3902
Dnm2	9267	9872	8769	9084	8906	9248	8439	8396
Dnm3	13	38	36	27	26	29	44	26
Dnmbp	3093	3382	3184	3107	3469	3385	3333	2948
Dnmt1	1857	2179	2010	1943	2115	1864	1743	1732
Dnmt3a	1313	1399	1520	1525	1402	1351	1266	1200
Dnmt3b	70	109	68	60	89	101	55	69
Dnmt3l	5	17	5	18	14	2	13	20
Dnpep	6695	6807	6512	6487	6585	6914	6811	6231
Dnph1	144	137	140	129	91	148	103	85
Dntt	0	0	5	0	0	0	0	0
Dnttip1	973	1090	965	1061	990	1064	1012	970
Dnttip2	1242	1320	1236	1293	1323	1365	1412	1219
Doc2a	10	4	15	9	5	14	1	11
Doc2b	12	19	13	13	17	8	19	8
Doc2g	30	38	43	36	44	38	39	41
Dock1	1350	1711	1496	1484	1345	1339	1340	1321
Dock10	300	270	254	256	247	291	247	208
Dock11	773	859	735	772	762	885	706	568
Dock2	177	231	203	208	227	159	233	161
Dock3	79	84	64	80	47	54	56	38
Dock4	155	140	87	169	128	150	106	115
Dock5	4709	5161	4751	5017	4678	4929	4785	4317
Dock6	1614	1765	1656	1647	1471	1550	1460	1558
Dock7	1601	1737	1714	1828	1583	1564	1459	1318
Dock8	3908	4771	4109	3682	4494	4691	4889	4061
Dock9	590	568	712	692	602	663	563	524
Dohh	1716	1783	1794	1622	1806	1850	1596	1658
Dok1	262	330	266	270	309	299	256	260
Dok2	62	62	114	52	73	94	74	87
Dok3	86	95	96	118	152	64	70	124
Dok4	6190	6194	6037	5649	5878	6191	6019	5438
Dok5	10	2	1	11	9	3	5	1
Dok6	2	0	8	4	7	3	9	1
Dok7	11	10	18	16	9	11	19	7

Transcriptome sequencing yielded total genetic results for the MOD and APS groups, with a total of 15,936 variables

Continued from above

Dolk	1486	1298	1329	1572	1205	1253	1159	1212
Dolpp1	2421	2487	2352	2407	2293	2361	2200	2272
Donson	514	609	455	533	531	543	574	526
Dop1a	1062	1381	1116	1158	1318	1319	1164	1092
Dop1b	3058	3703	3515	3142	3293	3261	3347	3031
Dot1l	3605	3934	3530	3590	3790	3539	3425	3531
Doxl1	0	0	1	0	1	1	0	0
Dpagt1	1480	1521	1497	1395	1538	1506	1386	1502
Dpcd	482	468	477	453	482	528	423	415
Dpep1	11352	10529	10219	11410	9337	10144	10646	9538
Dpep2	19	18	21	29	36	21	31	28
Dpep3	0	0	0	0	1	1	0	0
Dpf1	2	1	5	4	8	1	2	1
Dpf2	2286	2412	2432	2219	2569	2506	2408	2086
Dpf3	64	89	60	49	90	66	64	72
Dph1	229	253	264	195	232	275	209	193
Dph2	284	298	262	297	306	388	292	306
Dph3	1284	1303	1403	1398	1335	1332	1163	1248
Dph5	144	177	146	160	173	179	134	141
Dph6	302	286	280	278	252	289	231	253
Dph7	106	130	120	101	130	133	93	140
Dpm1	978	1125	1139	1028	1035	1048	957	1070
Dpm2	925	927	1052	916	922	851	786	912
Dpm3	494	545	552	577	586	561	492	519
Dpp10	2	1	1	0	0	6	4	2
Dpp3	4432	4834	4539	4530	4900	4914	4438	4575
Dpp4	25382	26397	22785	24352	22915	25438	28732	23850
Dpp6	6	11	14	3	9	14	13	7
Dpp7	418	428	421	420	437	445	446	448
Dpp8	3843	4160	3923	3921	4055	4189	3915	3729
Dpp9	2481	2514	2325	2441	2533	2596	2457	2338
Dppa2	1	0	0	0	0	0	2	0
Dppa3	10	4	4	9	11	9	3	10
Dppa5a	0	4	0	0	0	0	0	0
Dpt	627	569	616	617	603	588	591	554
Dpy19l1	410	417	437	423	489	464	422	435
Dpy19l3	58	82	50	108	70	98	76	69
Dpy19l4	743	941	902	778	792	828	903	889
Dpy30	841	855	864	845	877	894	774	791
Dpyd	5866	6710	5964	5684	5618	5889	6088	5458
Dpys	2	0	2	2	0	3	2	0
Dpysl2	170	214	242	180	216	178	164	177
Dpysl3	291	470	460	501	418	371	405	396
Dpysl4	1	2	6	0	3	1	1	2
Dpysl5	12	13	12	18	8	18	14	17
Dqx1	2539	2778	2771	2669	2544	2625	2420	2470
Dr1	2049	2186	2134	1976	2365	2237	2078	2111
Dram1	148	147	115	120	124	96	124	83
Dram2	3935	4052	3796	3810	4134	4404	4372	4147
Drap1	2154	2548	2488	2263	2315	2259	1918	2216
Draxin	6	1	7	1	2	4	2	0
Drc1	32	38	54	74	27	43	37	51
Drc3	74	92	56	87	65	65	88	76
Drd1	6	0	1	4	0	0	0	1
Drd3	10	5	2	6	12	9	5	1
Drd4	2	6	5	12	6	1	0	2
Drg1	1443	1605	1498	1327	1481	1665	1408	1482
Drg2	1525	1550	1592	1530	1543	1545	1432	1442

Transcriptome sequencing yielded total genetic results for the MOD and APS groups, with a total of 15,936 variables

Continued from above

Drosha	1563	1698	1545	1587	1661	1516	1568	1399
Drp2	22	4	10	8	7	20	12	6
Dsc2	8012	9196	8368	7948	9532	10243	9701	8656
Dsc3	5	2	0	1	0	7	0	0
Dscam	0	1	1	2	0	0	1	2
Dscaml1	3	1	0	2	5	6	2	5
Dscc1	48	68	36	45	52	64	51	31
Dse	138	137	135	119	181	135	153	159
Dsel	47	66	42	50	90	70	37	64
Dsg1a	1	0	0	0	0	0	0	0
Dsg1c	1	1	1	0	0	0	0	0
Dsg2	10217	11485	10612	10209	10959	11206	10907	10374
Dsg4	27	20	22	34	19	23	25	25
Dsn1	206	299	250	204	302	277	249	278
Dsp	8596	9761	9318	8991	10276	10171	10049	9065
Dst	3438	3704	3730	3569	3083	3379	3617	3143
Dstn	47162	51382	46329	46152	49887	51773	52899	50678
Dstyk	127	227	194	192	199	180	157	113
Dtd1	206	263	230	209	203	282	252	172
Dtd2	602	642	572	578	617	629	578	533
Dtl	382	426	369	370	446	442	290	333
Dtna	57	66	86	112	74	73	79	77
Dtnb	127	151	150	132	114	126	131	144
Dtnbp1	660	730	774	673	618	632	558	548
Dtwd1	150	124	176	115	151	156	149	163
Dtwd2	52	68	58	79	90	55	101	26
Dtx1	200	200	140	174	201	150	174	137
Dtx2	314	339	273	342	381	307	344	268
Dtx3	167	164	200	166	155	191	144	151
Dtx3l	7226	9062	6999	6617	6800	7436	6956	6294
Dtx4	1257	1347	1348	1216	1335	1218	1087	1129
Dtymk	733	846	833	866	808	887	671	683
Duox1	13	21	25	10	23	23	14	17
Duox2	21438	14386	17634	25518	12196	11978	12499	12328
Duoxa1	0	2	0	3	1	0	0	0
Duoxa2	11950	6710	8147	14170	4780	4731	4827	5262
Dus1l	1452	1507	1461	1479	1559	1489	1341	1376
Dus2	195	270	242	170	202	248	213	167
Dus3l	1059	1048	1200	1141	1260	1212	1068	973
Dus4l	130	135	124	149	143	142	120	152
Dusp1	589	704	806	737	651	800	667	637
Dusp10	117	118	108	174	119	81	112	105
Dusp11	3495	3956	3983	3769	4110	4192	3995	3949
Dusp12	229	287	231	232	240	241	206	200
Dusp13	10	1	2	0	13	14	3	2
Dusp14	64	70	59	63	62	72	93	81
Dusp16	1860	2112	1877	1985	2090	2201	2201	1915
Dusp18	130	126	152	112	132	139	111	124
Dusp19	254	264	213	281	271	275	299	249
Dusp2	52	48	51	58	20	5	31	33
Dusp22	30	32	28	37	35	35	26	31
Dusp23	94	160	113	143	134	154	141	161
Dusp26	22	20	23	7	16	26	6	26
Dusp27	5	2	5	13	2	8	7	8
Dusp28	261	269	235	257	184	303	159	199
Dusp3	479	508	534	613	518	601	599	542
Dusp4	247	287	240	260	310	253	224	224
Dusp5	1021	902	950	906	1151	965	1129	985

Transcriptome sequencing yielded total genetic results for the MOD and APS groups, with a total of 15,936 variables

Continued from above

Dusp6	3483	4113	3549	3095	3341	3362	3569	3648
Dusp7	192	220	183	213	200	184	196	134
Dusp8	746	830	784	743	781	843	806	722
Dusp9	28	24	45	41	26	45	57	35
Dut	815	910	779	828	897	884	666	758
Duxbl1	2	7	1	1	1	5	5	1
Dvl1	3392	3712	3588	3270	3620	3842	3407	3409
Dvl2	227	201	229	188	158	199	155	206
Dvl3	1319	1422	1406	1423	1420	1372	1368	1386
Dxo	867	927	960	901	933	896	859	865
Dym	622	751	636	706	756	644	675	629
Dynap	0	0	0	0	1	0	0	0
Dync1h1	13391	15109	14214	13808	13961	13855	14046	12039
Dync1i1	9	13	16	7	1	2	11	12
Dync1i2	3378	3656	3868	3599	3627	3882	3586	3494
Dync1li1	2472	2548	2509	2345	2681	2690	2530	2396
Dync1li2	2011	2099	2079	2205	2076	2160	2169	2067
Dync2h1	32	67	60	81	65	100	83	91
Dync2li1	23	32	19	26	29	32	30	16
Dynll1	1689	1700	1885	1846	2444	2330	1874	2197
Dynll2	9953	10044	9947	10466	9421	9940	9227	8961
Dynlrb1	1931	2273	2318	2093	2101	2113	1919	2038
Dynlrb2	0	0	0	1	0	0	0	0
Dynlt1a	799	864	926	927	737	805	857	783
Dynlt1b	985	886	818	972	764	841	770	886
Dynlt1c	164	150	152	151	127	118	177	116
Dynlt1f	609	605	755	696	657	616	589	572
Dynlt3	1194	1298	1219	1267	1412	1526	1400	1432
Dyrk1a	2708	3053	2912	2880	2860	2699	2934	2538
Dyrk1b	806	797	683	721	598	596	580	709
Dyrk2	5145	6041	5391	5515	5993	6485	6691	5556
Dyrk3	57	80	43	30	41	39	60	58
Dyrk4	1	13	1	0	1	3	0	1
Dysf	259	260	281	228	192	213	221	221
Dzank1	12	6	10	8	5	3	17	16
Dzip1	33	76	69	61	42	46	48	26
Dzip1l	26	27	20	23	43	28	26	40
Dzip3	128	103	145	118	163	150	204	117
E030030I0	30	45	33	34	71	49	52	56
E130308A	287	316	264	248	236	321	274	252
E130309D	803	942	981	803	909	940	743	770
E130311K	20	25	30	30	56	33	41	44
E230025N	1	1	11	7	8	3	10	6
E2f1	239	217	232	220	248	169	179	179
E2f2	2349	2847	2368	2300	2558	2695	2182	2309
E2f3	1292	1466	1372	1204	1320	1537	1581	1263
E2f4	2002	2331	2080	2176	2342	2222	1925	2082
E2f5	423	474	465	431	502	411	468	473
E2f6	513	526	538	465	616	629	478	525
E2f7	172	230	242	177	159	149	139	120
E2f8	893	977	1006	972	1032	951	844	829
E330014E	0	2	0	0	0	0	0	0
E330021D	0	0	0	0	0	0	0	6
E430018J2	619	660	559	533	514	594	491	515
E4f1	497	491	438	423	387	388	362	385
Eaf1	2249	2259	2100	2298	2313	2148	2303	2064
Eaf2	32	40	34	63	35	32	23	56
Eapp	1074	1012	1064	1048	973	1101	1036	909

Transcriptome sequencing yielded total genetic results for the MOD and APS groups, with a total of 15,936 variables

Continued from above

Ear2	6	12	6	6	12	12	27	24
Ears2	650	676	610	643	733	779	625	633
Ebag9	811	811	815	793	888	863	834	768
Ebf1	34	30	57	39	43	42	23	39
Ebf2	4	6	4	2	11	0	0	1
Ebf3	9	15	16	14	10	8	9	10
Ebf4	15	7	6	15	9	20	9	14
Ebi3	75	66	48	44	39	64	42	61
Ebna1bp2	1133	1379	1031	1162	1264	1304	1149	1220
Ebp	2804	2916	2888	2940	2908	2808	2681	2900
Ebpl	461	498	437	480	569	573	472	490
Ecd	864	1000	990	923	967	928	990	877
Ece1	3591	3805	3646	3752	3207	3355	2995	3095
Ece2	2	0	2	0	0	2	0	0
Ecel1	0	4	0	2	0	5	0	0
Ech1	6231	6664	6140	6462	6060	6645	6286	6394
Echdc1	1298	1408	1323	1448	1595	1612	1608	1486
Echdc2	311	401	365	464	449	429	405	387
Echdc3	45	57	31	70	46	64	80	36
Echs1	5555	5503	5615	5779	5408	5439	5283	5517
Eci1	1722	1840	1839	1727	1882	1909	1773	1585
Eci2	2110	2277	2163	2248	2159	2341	2022	2197
Eci3	2221	2386	2070	2199	2312	2301	2487	2150
Ecm1	1543	1489	1471	1535	1388	1395	1405	1385
Ecm2	32	63	20	38	41	38	29	27
Ecpas	4207	5032	4598	4697	4601	4727	4716	4451
Ecrg4	0	0	4	0	1	0	0	1
Ecscr	116	172	177	193	154	155	152	179
Ecsit	1173	1262	1287	1342	1300	1316	1051	1085
Ect2	844	944	994	1004	1062	1042	1007	893
Ect2l	0	1	0	1	1	0	1	1
Eda	2	20	25	7	15	12	6	15
Eda2r	79	50	60	70	114	103	78	72
Edar	23	8	10	17	12	6	8	25
Edaradd	30	33	32	16	31	19	38	34
Edc3	1246	1434	1371	1379	1632	1507	1476	1326
Edc4	1229	1406	1243	1335	1295	1189	1236	1166
Eddm3b	0	0	1	0	0	0	0	0
Edem1	6627	7529	6389	6891	7613	7503	7102	7234
Edem2	2463	2776	2689	2563	3040	3035	2601	2664
Edem3	3600	3731	3677	3758	4162	4340	4242	4014
Edf1	9991	10327	9989	10478	9237	9917	9707	9488
Edil3	18	28	21	15	27	18	15	10
Edn1	34	20	24	26	12	26	21	47
Edn2	193	195	216	224	142	201	155	158
Edn3	1750	1554	1480	1531	1194	1235	1349	1106
Ednra	187	197	153	227	179	166	115	116
Ednrb	439	547	548	530	564	500	448	446
Edrf1	686	715	723	740	805	812	711	723
Eea1	1177	1364	1369	1119	1384	1553	1476	1227
Eed	1068	1322	1180	1227	1347	1349	1276	1194
Eef1a1	154009	164313	164686	162071	166335	163389	148360	155768
Eef1a2	27	78	76	58	52	64	43	60
Eef1akmt1	354	390	367	349	522	410	359	367
Eef1akmt2	186	131	165	174	187	127	158	165
Eef1akmt3	7	3	6	8	22	8	10	9
Eef1akmt4	79	114	85	73	122	112	51	101
Eef1aknmt	263	272	203	209	197	244	211	197

Transcriptome sequencing yielded total genetic results for the MOD and APS groups, with a total of 15,936 variables

Continued from above

Eef1b2	11556	12342	12106	12168	12161	12283	11131	12050
Eef1d	3117	3137	3130	3091	3599	3596	3002	3327
Eef1e1	499	649	626	540	604	631	664	614
Eef1g	14248	15266	14890	13773	14702	14705	13296	13736
Eef2	87964	88755	90335	89391	85761	85941	80128	82315
Eef2k	1011	1000	981	984	1138	1071	937	1011
Eef2kmt	450	457	436	489	468	449	504	463
Eefsec	512	571	585	516	572	602	480	491
Eepd1	624	748	642	545	616	657	644	529
Efcab1	7	2	9	6	9	1	3	6
Efcab11	25	19	63	63	27	18	38	21
Efcab12	4	0	5	5	3	9	6	6
Efcab14	5298	5912	5018	4727	5587	5661	5638	5339
Efcab15	0	2	0	2	6	2	0	0
Efcab2	76	88	75	77	104	88	91	91
Efcab3	1	0	3	0	0	0	0	1
Efcab5	42	40	62	69	45	73	68	46
Efcab6	0	0	0	0	1	0	0	0
Efcab7	24	14	22	29	14	24	14	21
Efcab8	7	3	0	12	0	4	2	1
Efcab9	0	0	7	1	9	1	1	0
Efcc1	22	39	23	30	31	35	64	23
Efemp1	319	363	438	360	357	411	378	363
Efemp2	223	178	218	216	270	232	243	256
Efhb	9	14	10	17	1	10	14	12
Efhc1	12	11	1	0	5	7	5	1
Efhc2	4	2	0	2	0	0	4	1
Efhd1	9	19	19	7	17	9	13	8
Efhd2	12809	13070	12660	12770	12027	12493	12249	12897
Efl1	2089	2379	2176	2057	2179	2117	2043	1976
Efna1	3138	3040	2820	3289	3378	3319	3133	3163
Efna2	12	7	18	2	10	11	16	8
Efna3	144	113	102	99	129	133	133	118
Efna4	165	179	212	238	262	190	224	150
Efna5	28	27	22	32	40	28	47	23
Efnb1	3971	4194	4087	3929	3911	4018	4087	3712
Efnb2	4743	5423	4849	4815	4257	4509	4363	4381
Efnb3	41	33	43	58	45	49	43	36
Efr3a	3424	3737	3331	3417	3582	3580	3376	3297
Efr3b	1054	1163	1025	833	1232	1331	1390	1237
Efs	37	52	59	35	42	29	46	18
Eftud2	2218	2481	2351	2048	2281	2184	2075	2084
Egfem1	0	0	1	0	0	1	1	0
Egfl6	2	1	1	1	0	0	0	0
Egfl7	298	270	326	351	318	230	266	267
Egfl8	9	19	16	28	25	9	12	15
Egflam	58	42	55	67	71	61	49	49
Egfr	740	781	738	745	843	895	751	725
Egln1	1654	1596	1650	1985	1638	1711	1757	1686
Egln2	3102	3064	3169	3156	2915	3013	3160	2859
Egln3	3544	2585	2965	4009	2050	2260	2278	2202
Egr1	381	399	382	395	368	290	312	324
Egr2	12	22	18	22	12	14	18	13
Egr3	9	13	8	8	18	14	7	17
Egr4	0	0	0	0	0	0	1	0
Ehbp1	307	330	306	239	316	335	348	319
Ehbp1l1	4712	4821	4421	4605	4565	4645	4460	4317
Ehd1	8598	9047	7926	7655	8435	8707	9371	8622

Transcriptome sequencing yielded total genetic results for the MOD and APS groups, with a total of 15,936 variables

Continued from above

Ehd2	728	627	777	790	756	809	728	712
Ehd3	144	119	123	114	124	123	104	115
Ehd4	2723	3172	2709	2548	2767	2841	2719	2653
Ehf	2634	2755	2585	2700	2543	2339	2381	2402
Ehhadh	8696	9616	8331	8279	8823	9791	9560	8878
Ehmt1	1349	1508	1319	1353	1480	1436	1382	1307
Ehmt2	5031	5184	5164	4812	5065	4963	4551	4727
Ei24	3801	3965	4127	3810	3911	4030	3709	3604
Eid1	405	469	347	457	406	439	451	391
Eid2	44	31	16	40	34	34	57	29
Eid2b	54	57	46	47	93	49	48	60
Eid3	12	1	7	4	28	18	4	4
Eif1	8209	8597	8934	9344	9046	9750	8609	8695
Eif1a	3649	4179	3763	3809	3997	4356	3951	3629
Eif1ad	2716	2826	2554	2716	2739	2693	2623	2380
Eif1ax	1789	1809	1749	1837	1964	1757	1669	1719
Eif1b	558	618	569	595	611	546	504	565
Eif2a	1980	2185	2018	2284	2122	2187	2035	2038
Eif2ak1	3539	3885	3646	3525	3401	3531	3507	3361
Eif2ak2	3936	4909	4468	4072	4244	4393	4387	3938
Eif2ak3	1326	1406	1283	1286	1602	1556	1544	1523
Eif2ak4	198	326	320	271	234	239	307	238
Eif2b1	1261	1413	1107	1235	1357	1282	1014	1382
Eif2b2	1320	1375	1441	1150	1175	1394	1163	1171
Eif2b3	197	209	258	172	194	242	183	172
Eif2b4	1201	1334	1299	1197	1426	1249	1197	1295
Eif2b5	1893	2036	1957	1781	2161	2053	1840	2024
Eif2d	1216	1220	1316	1209	1309	1290	1071	1112
Eif2s1	2149	2282	2325	2053	2800	2585	2335	2268
Eif2s2	4501	4437	4383	4294	4659	4870	4914	4348
Eif2s3x	2673	3135	3570	3142	3505	3615	3024	2680
Eif2s3y	1235	879	0	614	317	411	939	1194
Eif3a	6202	6505	6126	6224	6813	7219	6562	6149
Eif3b	6380	6943	6313	5987	6866	6719	5734	6019
Eif3c	6734	7324	7269	6769	7296	7130	6710	6590
Eif3d	3251	3561	3259	3178	3508	3185	2953	3175
Eif3e	4775	5179	4941	4803	5193	5556	4976	5023
Eif3f	6856	6793	7029	6587	6393	6704	5996	6256
Eif3g	2185	2438	2244	2204	2307	2362	2306	2283
Eif3h	6195	6543	6677	6612	6428	6566	5774	6082
Eif3i	3219	3612	3419	3422	3625	3496	3025	3377
Eif3j1	1118	1269	1051	1060	1265	1389	1180	1264
Eif3j2	24	38	48	151	103	37	78	55
Eif3k	3088	3246	3187	3220	3063	3266	3079	3049
Eif3l	4754	4946	4906	4707	5007	5096	4555	4667
Eif3m	2682	2683	2686	2720	2848	2888	2791	2744
Eif4a1	13843	14911	13663	13880	15343	15120	13735	13993
Eif4a2	8180	8393	7755	8121	9247	9169	9550	8783
Eif4a3	2565	2483	2600	2495	2380	2453	2322	2302
Eif4b	11438	12421	12391	12055	11754	11863	10965	11147
Eif4e	2406	2388	2223	2360	2731	2611	2422	2448
Eif4e2	3138	3412	3100	3379	3486	3448	2943	3165
Eif4e3	1139	1103	1080	1124	936	919	932	868
Eif4ebp1	866	905	770	906	973	845	926	903
Eif4ebp2	2394	2824	2874	2392	2767	2609	2681	2648
Eif4ebp3	454	524	530	542	523	525	551	542
Eif4enif1	1573	1798	1634	1652	1716	1741	1675	1495
Eif4g1	19254	21077	20020	18978	21413	21102	19395	18843

Transcriptome sequencing yielded total genetic results for the MOD and APS groups, with a total of 15,936 variables

Continued from above

Eif4g2	23270	26658	24651	23963	26952	26822	25482	24451
Eif4g3	1942	2207	2125	2031	2120	2135	1965	1923
Eif4h	16573	16874	15595	15519	15980	15957	15467	14526
Eif5	7667	8109	7149	7723	8593	9128	8580	7512
Eif5a	17361	18233	17607	17448	18490	17581	16272	16976
Eif5a2	72	67	42	35	60	86	68	45
Eif5b	2381	2606	2379	2484	2777	3040	2647	2441
Eif6	10379	10929	9800	9584	10130	10963	10845	10069
Eipr1	1052	1128	1028	1044	951	1098	1028	973
Elac1	708	842	773	669	726	741	915	739
Elac2	762	782	755	747	769	724	689	717
Elapor1	1046	1083	1232	1283	1072	1131	993	1153
Elapor2	8	11	15	13	13	2	2	7
Elavl1	2024	2132	2055	2089	2274	2135	2125	2122
Elavl2	10	1	5	1	1	2	2	1
Elavl3	10	2	6	14	7	4	3	4
Elavl4	12	40	30	39	16	21	22	21
Elf1	4175	4713	4375	4279	4285	4357	4099	3832
Elf2	1314	1444	1611	1412	1538	1471	1494	1309
Elf3	15897	16663	16788	16786	16972	16999	14937	16092
Elf4	2624	2909	2635	2562	2643	2654	2899	2548
Elfn1	0	1	6	0	0	0	4	1
Elfn2	17	14	18	19	15	19	10	17
Elk1	142	132	175	122	104	130	98	132
Elk3	308	227	279	279	274	213	259	191
Elk4	643	699	768	759	788	754	607	665
Ell	1617	1659	1524	1637	1605	1645	1520	1463
Ell2	1453	2054	1546	1410	1898	2007	2106	1885
Ell3	41	36	43	53	43	65	43	43
Elmo1	1369	1597	1242	1123	1150	1320	1211	1147
Elmo2	3173	2912	3036	3158	2425	2437	2576	2601
Elmo3	3016	2845	2661	2583	2630	2657	2780	2582
Elmod1	0	1	1	5	1	1	1	0
Elmod2	1408	1656	1321	1298	1463	1719	1687	1537
Elmod3	732	783	782	672	814	895	778	740
Eln	502	506	655	630	568	535	411	490
Eloa	2038	2129	1954	2103	2059	2154	2049	1897
Elob	4412	4507	4392	4356	4197	4242	4154	4039
Eloc	1157	1386	1204	1356	1303	1408	1434	1345
Elof1	1610	1644	1592	1660	1685	1596	1462	1562
Elovl1	3785	3741	3833	3798	3910	3977	4033	3940
Elovl2	5	14	1	7	8	6	7	6
Elovl3	0	0	0	0	4	0	0	5
Elovl4	2	2	7	6	1	7	3	10
Elovl5	916	1096	1092	956	1064	992	903	833
Elovl6	7583	7407	7756	8630	7230	7425	7511	7179
Elovl7	3206	3202	3268	3368	3412	3330	3263	3202
Elp1	500	506	492	502	487	521	499	526
Elp2	1302	1498	1465	1472	1355	1359	1191	1284
Elp3	889	899	865	876	970	937	825	831
Elp4	322	374	355	320	303	362	336	257
Elp5	1111	1194	1140	1146	1043	1175	1049	1069
Elp6	105	131	135	125	198	163	135	154
Emb	125	120	138	160	136	172	128	147
Emc1	519	675	635	551	567	470	427	579
Emc10	3379	3197	3313	3103	3537	3529	3204	3330
Emc2	1247	1238	1236	1298	1248	1279	1346	1232
Emc3	2857	2634	2607	2725	2926	2798	2626	2581

Transcriptome sequencing yielded total genetic results for the MOD and APS groups, with a total of 15,936 variables

Continued from above

Emc4	1118	1371	1291	1317	1402	1341	1097	1216
Emc6	1377	1375	1339	1421	1557	1432	1448	1329
Emc7	1637	1858	1522	1716	1762	1725	1597	1741
Emc8	840	844	900	781	823	874	783	720
Emc9	522	593	567	459	533	543	457	451
Emcn	188	153	198	193	197	167	162	180
Emd	765	829	832	827	876	812	861	855
Eme1	155	189	190	202	194	159	150	172
Eme2	504	555	415	549	532	491	401	405
Emg1	1777	2158	1804	2019	1973	1997	1779	1806
Emid1	187	178	229	180	150	178	180	140
Emilin1	1367	1445	1387	1280	1265	1249	1053	1140
Emilin2	40	45	22	39	23	38	51	40
Emilin3	0	0	0	1	9	6	0	0
Eml1	314	273	320	319	333	349	352	379
Eml2	574	486	535	442	394	482	477	462
Eml3	862	791	936	920	794	821	809	800
Eml4	2532	2856	2792	2729	3190	3310	2812	2738
Eml5	22	41	40	31	18	32	8	25
Eml6	34	38	36	44	55	59	32	20
Emp1	10818	12033	10787	10679	10800	11354	12804	12118
Emp2	285	315	433	345	326	252	279	281
Emp3	123	179	130	132	129	99	144	124
Emsy	518	657	586	571	638	594	588	526
En2	1	0	0	0	0	0	0	0
Enah	169	202	211	167	115	210	191	194
Enam	4	1	0	0	0	0	0	0
Enc1	483	507	556	471	501	425	409	397
Endod1	1242	1268	1307	1163	1211	1340	1206	1217
Endog	1490	1293	1516	1251	1493	1315	1399	1416
Endou	10	1	6	13	2	11	5	17
Endov	790	859	766	778	827	740	706	705
Eng	880	1020	1044	1026	819	824	842	810
Engase	1346	1288	1317	1276	1295	1278	1217	1092
Enho	47	45	60	36	43	51	34	53
Enkd1	93	100	69	92	70	65	83	58
Enkur	2	6	12	7	16	20	3	16
Eno1	40597	39472	39581	40387	41266	41972	42373	42146
Eno1b	562	612	633	478	685	607	641	630
Eno2	46	80	65	43	48	57	59	63
Eno3	931	1022	993	800	703	828	768	854
Eno4	0	1	2	1	0	6	0	1
Enoph1	583	529	666	532	663	609	546	572
Enox1	7	14	10	13	5	13	0	21
Enox2	451	492	424	441	432	452	536	490
Enpep	40005	48032	39948	35182	44769	47408	50430	44867
Enpp1	675	746	718	592	777	816	735	749
Enpp2	86	98	71	99	65	90	58	44
Enpp3	20045	21657	15944	15895	17629	18890	21908	18736
Enpp4	385	478	466	386	415	369	402	387
Enpp5	591	699	667	677	690	663	599	625
Enpp6	7	22	15	16	15	25	26	33
Enpp7	4942	4976	4087	4727	3722	4471	6779	5157
Ensa	4101	4499	4260	4121	4608	4811	4696	4290
Enthd1	0	1	0	0	0	0	0	0
Entpd1	399	474	431	476	479	410	389	388
Entpd2	1445	1449	1409	1240	1278	1386	1297	1235
Entpd3	29	47	18	18	12	29	20	14

Transcriptome sequencing yielded total genetic results for the MOD and APS groups, with a total of 15,936 variables

Continued from above

Entpd4	0	2	1	2	2	3	2	1
Entpd4b	120	15	19	21	38	29	78	66
Entpd5	9752	10804	9146	8777	12644	12486	11402	11177
Entpd6	898	945	1003	1044	974	925	801	802
Entpd7	4169	4329	4424	4653	4126	4592	4783	4782
Entpd8	5737	5941	5849	5570	5702	6059	5941	5670
Entr1	1582	1739	1636	1662	1634	1633	1704	1676
Eny2	1399	1611	1417	1566	1501	1531	1394	1347
Eogt	414	391	430	405	424	440	412	363
Eola1	286	366	291	364	333	313	338	328
Eomes	1	2	0	0	6	6	4	3
Ep300	3290	3755	3445	3462	3756	3786	3812	3378
Ep400	3008	3414	3461	3147	3306	3275	3006	2790
Epas1	1917	2000	1883	1905	1967	1868	1901	1866
Epb41	4513	4250	4469	4733	4420	4261	4026	4165
Epb41l1	622	716	596	573	511	637	616	598
Epb41l2	2240	2469	2380	2356	2723	2415	2363	2229
Epb41l3	17234	17621	16739	17328	15149	17012	17964	15481
Epb41l4a	76	55	50	68	37	57	46	61
Epb41l4b	12951	13504	12244	11959	12724	12936	12908	12091
Epb41l5	589	646	672	610	637	607	586	568
Epb42	28	8	15	6	16	23	13	17
Epc1	892	1024	1011	928	1061	1034	1005	915
Epc2	829	939	902	815	891	939	900	830
Epcam	55047	58325	54820	52171	58274	59419	57464	56008
Epdr1	51	50	47	35	42	62	53	33
Epg5	1884	2195	1854	1887	2088	2143	2223	1905
Epgn	1	0	9	0	5	0	5	0
Epha1	3597	3947	4092	3856	3928	4056	4171	4186
Epha10	0	1	0	0	0	0	0	0
Epha2	2233	2401	2464	2210	3039	3194	2982	2622
Epha3	5	0	3	1	2	1	3	2
Epha4	62	88	36	103	86	65	94	77
Epha5	0	3	1	5	0	4	1	2
Epha7	14	17	19	11	8	17	13	19
Epha8	1	0	1	1	0	10	1	1
Ephb1	19	17	16	14	5	21	4	10
Ephb2	1743	2277	2133	1873	2149	2139	1834	1871
Ephb3	392	414	407	408	333	391	284	303
Ephb4	2353	2456	2729	2455	2237	2313	2046	1995
Ephb6	11	7	6	13	16	6	8	15
Ephx1	511	553	545	467	813	776	660	771
Ephx2	15528	17280	14694	13902	17022	18542	18927	17536
Ephx3	11	8	0	4	9	6	12	13
Ephx4	2	8	1	5	4	5	4	12
Epm2a	14	9	22	8	12	9	23	3
Epm2aip1	906	931	839	851	1038	999	855	979
Epn1	14551	15022	14362	13467	14346	14817	14261	13695
Epn2	393	463	409	459	397	435	350	371
Epn3	225	194	194	254	183	175	173	184
Epop	232	81	73	171	82	108	85	140
Epor	10	14	24	15	16	23	13	18
Epp13	0	0	0	0	0	1	0	2
Eppk1	795	1025	992	984	1043	926	885	761
Eprs	3195	3637	3261	3309	3867	3789	3545	3236
Eps15	2220	2507	2229	2282	2264	2261	2314	2081
Eps15l1	1347	1518	1482	1374	1315	1417	1306	1306
Eps8	11411	12312	10578	10747	11753	12668	12959	11856

Transcriptome sequencing yielded total genetic results for the MOD and APS groups, with a total of 15,936 variables

Continued from above

Eps8l1	113	69	94	97	101	98	105	112
Eps8l2	16622	16776	15522	15832	16015	17173	17494	15270
Eps8l3	12935	15495	15218	13255	15275	16331	17032	16055
Epsti1	2741	3238	2944	2567	2999	3145	2961	2604
Epyc	1	3	0	0	0	1	0	1
Eqtn	1	1	4	0	0	1	4	1
Eral1	528	590	597	559	530	627	584	536
Erap1	5074	5464	5203	5087	5275	5156	5100	4814
Erbb2	6782	7034	6932	6422	6656	6878	6674	6199
Erbb3	15306	18147	16978	15291	16552	17779	17499	16289
Erbb4	0	0	0	1	0	0	0	0
Erbin	8913	9798	8811	8828	9640	10375	10207	8916
Erc1	682	758	658	759	732	714	649	707
Erc2	13	1	18	3	9	2	2	10
Ercc1	230	255	239	226	209	264	145	213
Ercc2	788	799	710	716	946	770	731	788
Ercc3	1627	1801	1609	1573	1615	1690	1510	1559
Ercc4	372	485	486	406	426	464	493	405
Ercc5	410	399	413	471	493	431	352	371
Ercc6	434	481	488	559	465	570	504	491
Ercc6l	303	275	284	262	407	369	327	244
Ercc6l2	652	609	583	573	664	671	599	536
Ercc8	304	238	218	336	273	288	248	205
Ereg	335	418	474	401	341	445	345	412
Erf	1281	1365	1338	1277	1255	1299	1243	1328
Erfe	256	257	331	275	281	252	261	254
Erg	61	89	75	77	90	78	101	90
Erg28	1853	1839	1819	1857	1816	1825	1639	1806
Ergic1	1832	2070	2047	2025	2074	1978	1780	1903
Ergic2	2837	3186	3009	2710	3388	3390	3307	3029
Ergic3	5720	5980	5525	5619	5607	5705	5363	5086
Erh	1755	1777	1717	1685	1965	1777	1507	1706
Eri1	747	893	679	856	920	836	895	888
Eri2	178	230	191	211	319	241	206	222
Eri3	782	800	800	822	715	766	725	692
Erich1	243	222	250	196	244	211	197	173
Erich2	17	14	15	20	36	14	16	20
Erich3	0	0	0	3	1	4	0	0
Erich4	2471	2646	2386	2405	2789	2941	2763	2726
Erich5	0	0	1	4	1	1	9	7
Erich6	0	0	1	1	4	0	0	0
Erlec1	1242	1316	1311	1273	1338	1317	1259	1164
Erlin1	4152	4432	4178	3952	4113	4138	4561	4157
Erlin2	6266	7085	6682	6780	6998	7123	6926	6298
Ermap	33	32	19	15	30	44	22	26
Ermard	165	226	165	158	208	212	161	117
Ermn	4	0	0	1	0	2	0	0
Ermp1	2676	2981	2701	2660	3008	3269	2799	2776
Ern1	1587	1760	1730	1621	1911	1912	1674	1708
Ern2	598	496	578	592	527	489	547	459
Ero1a	3657	3572	3544	3930	4294	4272	4145	4079
Ero1b	889	1084	881	846	1150	1173	1198	968
Erp27	17	44	11	1	112	96	80	102
Erp29	2345	2529	2517	2400	2731	2639	2292	2332
Erp44	5568	5998	5578	5678	6158	5896	5810	5552
Errfi1	1985	2508	2702	1908	2337	2503	2415	2291
Erv3	0	1	0	0	0	0	0	1
Esam	210	307	312	288	243	229	231	230

Transcriptome sequencing yielded total genetic results for the MOD and APS groups, with a total of 15,936 variables

Continued from above

Esco1	882	922	784	1007	1156	1064	1011	1002
Esco2	208	279	281	256	313	283	263	264
Esd	6485	6717	6122	6291	6241	6705	5929	5787
Esd-ps	1	4	4	1	4	4	0	4
Esf1	456	417	412	443	544	542	478	498
Esm1	66	115	90	96	86	106	108	121
Espl1	499	506	503	484	579	572	352	476
Espn	6933	6883	6458	6170	6172	6829	7183	6550
Espnl	1	0	3	1	0	0	0	0
Esr1	6	3	10	3	2	10	1	1
Esrp1	2862	3204	3268	3156	3100	3025	2907	2964
Esrp2	1431	1398	1468	1487	1572	1492	1483	1532
Esrra	15227	15278	15310	14703	14009	14717	14508	14298
Esrrb	15	15	43	13	16	20	9	6
Esrrg	245	268	229	234	211	209	276	176
Ess2	490	504	478	519	495	408	447	456
Esx1	0	0	0	0	6	0	0	0
Esyt1	1148	1081	1140	1176	1168	1198	1012	1156
Esyt2	2943	3196	3184	3171	3173	3237	3484	3226
Esyt3	4	3	0	5	8	2	8	4
Etaa1	216	275	208	255	263	312	233	264
Etf1	6374	6955	6058	6101	6893	6926	6497	6517
Etfa	8916	9302	9098	9053	8592	9288	9144	8750
Etfb	5351	5436	5302	5238	5301	5549	4971	5155
Etfbkmt	352	325	364	419	398	459	433	442
Etfdh	4883	5017	5152	5029	5040	5597	5236	4862
Etfrf1	688	808	750	713	755	776	771	746
Ethe1	731	634	728	711	838	766	727	801
Etl4	2612	3033	3062	2805	2625	2990	3052	3080
Etnk1	6580	7467	6998	6646	8224	8442	8958	8385
Etnk2	12	15	3	8	14	5	14	25
Etnppl	24	24	15	43	21	19	17	17
Ets1	568	506	599	541	535	547	544	491
Ets2	4488	4992	4768	4673	4669	4897	4568	4527
Etv1	99	82	117	119	122	98	76	90
Etv3	4008	4441	4256	4261	4460	4480	4223	3980
Etv3l	0	0	2	1	0	0	1	1
Etv4	50	56	56	53	58	28	27	55
Etv5	233	250	174	180	236	209	205	241
Etv6	2145	2525	2417	2373	2317	2295	2128	2115
Eva1a	91	109	119	109	117	119	103	79
Eva1b	167	172	224	194	192	156	154	121
Eva1c	2	11	6	9	3	8	10	6
Evc	73	67	41	59	88	64	61	50
Evc2	25	68	55	54	30	50	62	43
Evi2a	79	69	59	82	93	80	94	60
Evi5	786	901	873	844	913	1045	963	887
Evi5l	89	76	76	67	77	72	62	70
Evl	245	278	277	259	224	257	228	207
Evpl	1828	1332	1521	2053	1197	1158	1376	1185
Evx1	7	0	6	1	1	1	1	2
Ewsr1	4486	5108	4922	4762	4318	4481	4150	4247
Exd1	9	15	5	7	15	6	7	6
Exd2	492	377	468	398	504	517	365	447
Exo1	221	247	162	201	231	225	169	189
Exo5	359	339	431	359	388	379	404	360
Exoc1	1171	1177	1210	1208	1218	1153	1187	1114
Exoc2	909	1002	984	965	941	869	782	908

Transcriptome sequencing yielded total genetic results for the MOD and APS groups, with a total of 15,936 variables

Continued from above

Exoc3	2441	2629	2292	2217	2654	2776	2577	2444
Exoc3l	35	12	33	42	31	18	8	11
Exoc3l2	283	248	246	257	299	315	211	276
Exoc3l4	12812	13815	11441	11259	12564	13483	14015	13012
Exoc4	1146	1249	1138	1282	1310	1241	1189	1119
Exoc5	1815	1980	1832	1803	2021	2005	1950	1865
Exoc6	1135	1262	1257	1215	1122	1214	1155	990
Exoc6b	1048	1234	1208	1081	1234	1178	1211	1117
Exoc7	1612	1762	1639	1652	1553	1522	1574	1582
Exoc8	847	859	828	833	881	761	943	718
Exog	132	152	210	143	250	190	220	160
Exosc1	275	319	303	248	301	380	273	308
Exosc10	1186	1145	1129	1152	1240	1203	1021	970
Exosc2	512	589	511	523	613	609	472	502
Exosc3	680	701	584	536	613	569	524	558
Exosc4	792	753	631	796	751	702	633	742
Exosc5	642	592	590	593	646	544	497	562
Exosc7	870	874	860	818	867	858	844	840
Exosc8	444	477	452	390	497	485	361	381
Exosc9	996	1072	1002	852	1083	1114	969	832
Exph5	2721	3028	2354	2827	2603	2711	2437	2306
Ext1	3573	3821	3459	3542	3493	3636	3207	2853
Ext2	1448	1442	1496	1404	1317	1288	1207	1234
Extl1	100	101	90	105	77	58	86	92
Extl2	165	229	198	194	174	238	190	169
Extl3	1045	1051	1003	953	1100	947	852	1036
Eya1	13	0	16	1	2	4	5	2
Eya2	25	5	3	8	7	13	19	6
Eya3	1605	1712	1574	1572	1800	1751	1576	1621
Eya4	2	2	1	1	4	6	0	1
Ezh1	1593	1790	1596	1469	1575	1556	1603	1528
Ezh2	1017	1203	970	1001	1058	1011	909	934
Ezr	36327	39298	34235	33470	35532	39285	42359	38012
F10	29	37	15	13	38	32	23	27
F11	1	0	1	0	1	1	7	4
F11r	7563	7875	7194	7361	7267	7583	7600	7323
F12	8	2	3	7	4	6	6	2
F13a1	6	11	6	8	24	18	26	15
F13b	0	2	0	0	4	3	0	1
F2	20	21	15	13	30	30	22	35
F2r	438	474	534	452	478	482	385	365
F2rl1	2196	2080	2036	2133	2042	2144	2210	2112
F2rl2	7	11	11	10	11	7	7	2
F2rl3	50	34	49	47	75	45	62	43
F3	231	279	283	305	250	248	195	202
F5	22	39	36	41	30	34	47	24
F7	4	2	4	4	1	6	1	0
F8	11	6	1	8	12	3	12	1
F830016B(	24	48	42	50	28	19	55	23
F8a	508	429	471	483	525	527	367	463
F9	1	2	1	1	0	4	1	7
Fa2h	3	0	13	4	1	7	6	9
Faah	6229	6859	6266	5717	6267	6598	6647	6382
Faap100	476	543	521	419	441	431	416	399
Faap20	458	452	438	455	411	451	435	410
Faap24	314	316	226	275	304	328	276	241
Fabp1	64370	66244	64296	66281	67754	70722	73892	69630
Fabp12	6	4	0	2	6	0	0	7

Transcriptome sequencing yielded total genetic results for the MOD and APS groups, with a total of 15,936 variables

Continued from above

Fabp2	95597	112592	93092	80602	117378	129351	125453	107355
Fabp3	14	5	7	1	3	5	10	8
Fabp4	209	251	337	402	346	447	334	371
Fabp5	279	191	265	245	239	250	242	221
Fabp6	2	1	7	2	14	5	7	15
Fabp7	5	18	0	0	6	15	8	10
Fadd	887	1105	954	916	921	909	767	777
Fads1	1769	1900	2000	1815	1497	1604	1393	1409
Fads2	4350	5207	5301	4711	4359	3991	3770	3932
Fads3	335	305	347	353	316	309	296	331
Fads6	92	42	61	95	57	89	81	74
Faf1	1687	1785	1735	1711	1828	1815	1718	1698
Faf2	2741	2843	2757	2648	2737	2703	2590	2482
Fah	63	70	76	72	63	69	69	109
Fahd1	6041	6349	6183	6553	5076	5454	5665	5217
Fahd2a	205	234	285	236	254	243	235	227
Faim	207	239	246	250	255	298	221	266
Faim2	8	15	17	20	24	34	21	13
Fam102a	5075	5570	5732	5336	6964	7198	7162	7173
Fam102b	747	825	790	825	734	783	724	823
Fam104a	1281	1399	1449	1463	1413	1387	1143	1267
Fam107a	6	17	20	10	31	21	16	25
Fam107b	2370	2529	2501	2405	2826	2850	2677	2664
Fam110a	254	311	351	349	311	303	305	288
Fam110b	51	68	39	56	29	43	41	58
Fam110c	1523	1716	1401	1405	1355	1527	1601	1385
Fam111a	407	451	399	401	524	508	392	411
Fam114a1	1485	1425	1354	1363	1439	1601	1525	1321
Fam114a2	2483	3075	2830	2649	3065	3144	2932	2892
Fam117a	232	181	177	174	228	173	155	129
Fam117b	1393	1558	1628	1581	1430	1419	1182	1161
Fam118a	851	921	735	659	997	997	987	964
Fam118b	1673	1711	1510	1828	1907	1713	1685	1675
Fam120a	15948	17104	16811	16587	18105	17674	16622	15969
Fam120b	1581	1724	1670	1494	1795	1814	1745	1623
Fam120c	216	233	196	221	285	290	238	266
Fam122a	461	486	404	413	483	417	450	430
Fam122b	235	223	272	215	204	233	187	181
Fam124a	13	24	39	31	25	38	51	38
Fam124b	4	4	4	0	1	1	0	4
Fam126a	500	507	553	572	503	547	480	381
Fam126b	1049	1139	991	989	1058	1383	1380	960
Fam131a	36	58	48	33	28	56	54	51
Fam131b	19	3	4	11	6	6	6	12
Fam131c	6	10	14	13	12	3	13	12
Fam133b	282	255	302	332	379	398	404	361
Fam135a	2316	2484	2486	2386	2403	2639	2499	2362
Fam135b	0	0	1	2	6	2	4	0
Fam136a	2058	2345	2110	2010	2436	2673	2189	2266
Fam13a	1934	2526	2056	1828	2662	2624	2225	1808
Fam13b	2852	3422	3310	3273	3356	3622	3762	3325
Fam13c	12	19	8	22	7	29	12	10
Fam149a	136	98	146	109	140	105	93	120
Fam149b	1101	1107	953	1200	1122	1130	1066	998
Fam151a	77	138	68	94	86	81	100	104
Fam151b	74	87	115	110	86	124	107	85
Fam155a	7	16	7	10	6	6	24	1
Fam160a1	1109	1195	987	1142	1169	1161	1116	1072

Transcriptome sequencing yielded total genetic results for the MOD and APS groups, with a total of 15,936 variables

Continued from above

Fam160a2	2531	2666	2648	2567	2563	2669	2499	2467
Fam160b1	2123	2211	2218	1930	2560	2452	2714	2294
Fam160b2	842	864	822	761	874	815	834	726
Fam161a	7	3	3	9	12	2	7	1
Fam161b	68	47	69	90	88	150	95	97
Fam162a	2146	2311	2168	2236	2808	2561	2475	2929
Fam162b	10	10	12	4	3	7	6	1
Fam163a	1	2	8	1	8	11	10	2
Fam163b	0	0	0	0	0	1	0	0
Fam166a	26	17	9	27	25	13	18	12
Fam166b	0	1	0	0	0	1	0	0
Fam166c	0	0	0	0	0	0	3	0
Fam167a	9	10	22	12	25	26	33	20
Fam167b	1	7	1	7	12	11	0	8
Fam168a	568	594	595	693	676	703	645	599
Fam168b	4478	4627	4432	4429	4541	4834	4355	4380
Fam169a	0	1	11	0	1	0	5	0
Fam169b	83	101	72	68	78	83	132	81
Fam170b	0	1	0	1	0	0	0	0
Fam171a1	259	294	273	332	291	283	241	227
Fam171a2	56	62	47	41	47	47	48	70
Fam171b	67	84	79	71	93	68	57	50
Fam172a	546	519	564	533	535	599	518	437
Fam174a	380	366	339	295	345	368	357	393
Fam174b	633	605	636	663	719	703	608	710
Fam174c	155	224	227	159	240	201	231	188
Fam177a	1	0	8	0	3	1	1	4
Fam177a2	23	17	13	20	21	25	13	20
Fam178b	6	2	2	2	6	1	1	0
Fam180a	0	11	3	5	6	6	12	9
Fam181b	2	0	3	7	4	0	2	1
Fam183b	18	18	24	18	22	36	16	25
Fam184a	2	1	1	8	5	5	0	0
Fam184b	4	1	1	9	4	2	5	0
Fam185a	457	543	496	465	469	511	451	499
Fam186b	0	0	6	1	0	0	0	1
Fam187b	137	125	132	146	177	163	159	160
Fam189a1	33	24	38	20	13	10	10	33
Fam189a2	105	104	100	94	135	124	105	114
Fam189b	112	87	88	65	91	83	88	75
Fam193a	1093	1172	1049	1004	1066	1134	1175	939
Fam193b	696	733	743	729	802	850	732	704
Fam199x	197	215	188	235	239	302	276	197
Fam204a	387	512	472	477	346	412	441	439
Fam205a1	0	1	0	0	0	0	0	0
Fam205c	0	0	0	0	0	0	0	1
Fam207a	1823	1776	1808	1792	2026	2009	1779	1882
Fam209	2	2	8	7	4	1	0	0
Fam20a	559	508	548	451	486	575	601	583
Fam20b	5435	6086	5589	5581	5310	5551	5462	4784
Fam20c	53	78	52	81	69	81	64	91
Fam210a	2064	2363	2265	2166	2135	2179	1934	1984
Fam210b	1326	1435	1306	1332	1411	1382	1373	1255
Fam214a	117	115	123	107	116	172	125	137
Fam214b	4233	4234	4320	3974	4190	4327	4611	4149
Fam216a	53	53	55	35	88	63	57	56
Fam217a	7	3	3	5	4	11	1	5
Fam217b	8	11	14	5	5	21	16	9

Transcriptome sequencing yielded total genetic results for the MOD and APS groups, with a total of 15,936 variables

Continued from above

Fam219a	210	176	170	149	178	188	169	207
Fam219b	170	181	156	190	215	189	140	241
Fam220a	253	184	226	190	210	276	236	238
Fam221a	66	53	51	57	34	51	21	59
Fam221b	0	0	0	4	0	0	0	0
Fam222a	41	64	96	61	39	60	84	74
Fam222b	488	389	554	462	469	432	392	416
Fam227a	4	7	20	11	13	3	12	10
Fam227b	0	2	6	0	0	2	1	2
Fam228a	0	0	2	0	0	0	1	0
Fam228b	1	1	0	0	4	0	4	16
Fam229a	1	9	1	0	1	1	0	1
Fam229b	1	7	0	3	1	5	3	6
Fam234a	12021	12707	11449	10812	13912	14184	13285	11985
Fam234b	2111	2190	2312	2214	2455	2491	2246	2135
Fam241a	608	623	612	607	568	648	535	525
Fam241b	580	578	470	514	517	543	492	525
Fam24b	1	0	0	4	0	0	1	1
Fam25c	0	0	0	0	1	0	0	0
Fam32a	3179	3575	3360	3071	3394	3521	3349	2972
Fam3a	646	660	656	698	569	627	729	640
Fam3b	6952	7150	6431	6916	6686	7265	7404	6843
Fam3c	2920	3091	2854	3065	2527	2861	2739	2554
Fam43a	172	128	193	134	153	167	115	155
Fam47e	1	5	6	1	2	3	1	3
Fam50a	626	598	605	624	676	658	614	607
Fam50b	1	4	0	0	0	0	0	0
Fam53a	977	988	934	781	927	938	893	849
Fam53b	2256	2382	2421	2120	2047	2246	2073	2104
Fam53c	733	863	826	811	897	863	784	788
Fam71a	0	0	0	0	0	1	1	0
Fam71b	0	1	0	4	4	0	0	3
Fam71d	1	0	1	0	0	0	0	0
Fam71e1	10	12	9	4	10	13	6	5
Fam71f2	2	0	0	2	2	0	0	5
Fam72a	34	48	34	38	59	27	44	35
Fam76a	1267	1392	1299	1418	1258	1200	1312	1318
Fam76b	828	871	902	969	846	759	840	776
Fam78a	1178	884	895	1004	792	882	850	784
Fam78b	7	8	2	6	6	18	9	8
Fam81a	1	1	0	1	0	5	1	3
Fam81b	1	0	0	0	0	0	0	1
Fam83a	11	12	5	7	2	7	12	11
Fam83b	2717	2751	2481	2627	2975	3033	3290	2825
Fam83c	32	27	15	13	17	34	31	19
Fam83d	338	387	410	330	385	353	321	302
Fam83e	3956	3868	4222	4140	3754	3997	3756	3948
Fam83f	1488	1452	1301	1406	1134	1242	1094	1202
Fam83g	1938	1752	1908	1786	1998	2062	2297	2031
Fam83h	3995	4212	4066	3943	3777	4040	4255	3932
Fam89a	11	22	17	15	18	24	18	11
Fam89b	424	428	447	462	396	459	424	384
Fam8a1	6586	7161	6564	6240	6917	7293	7391	6462
Fam91a1	4952	5504	5216	5014	5640	5790	5883	5287
Fam98a	1236	1325	1239	1263	1515	1459	1339	1297
Fam98b	926	1182	1037	999	1058	1025	908	980
Fam98c	2644	2643	2540	2564	2418	2559	2384	2288
Fan1	185	176	152	149	151	161	136	132

Transcriptome sequencing yielded total genetic results for the MOD and APS groups, with a total of 15,936 variables

Continued from above

Fanca	469	558	511	504	483	492	400	445
Fancb	76	85	84	63	78	108	101	81
Fancc	163	222	194	214	188	195	184	151
Fancd2	171	211	213	163	201	170	130	189
Fance	405	469	458	395	402	372	383	430
Fancf	190	219	169	193	221	165	193	198
Fancg	200	226	275	239	293	237	253	249
Fanci	149	199	201	149	208	159	133	186
Fancl	150	161	169	129	138	144	173	157
Fancm	148	176	163	168	151	172	184	163
Fank1	1	0	6	0	4	4	0	10
Fap	18	30	18	50	35	17	45	17
Far1	334	333	321	345	440	397	334	308
Far2	2726	3360	3167	2735	3462	3116	2936	2852
Farp1	878	1025	1013	1061	921	957	925	819
Farp2	690	783	732	644	880	787	794	685
Fars2	1398	1454	1459	1600	1280	1241	1238	1255
Farsa	981	1062	1049	1027	1295	1119	888	921
Farsb	2442	2707	2544	2442	2751	2614	2246	2281
Fas	1162	1340	996	1004	994	1121	1122	1008
Fasl	19	29	7	11	17	19	14	13
Fasn	4290	4406	4945	4608	4795	4972	4509	4106
Fastk	3783	3404	3581	3721	3376	3255	2960	3163
Fastkd1	534	540	458	516	460	467	458	485
Fastkd2	803	861	789	892	838	859	824	812
Fastkd3	248	312	298	250	313	215	259	245
Fastkd5	411	497	396	471	537	498	541	462
Fat1	9596	11135	10110	9970	10350	10165	10290	9276
Fat2	0	0	0	0	1	0	0	6
Fat3	0	0	0	5	2	2	16	6
Fat4	168	229	254	239	250	173	199	140
Fau	21009	20530	20245	21087	19333	19565	18410	18658
Faxc	58	54	55	60	47	62	59	44
Fbf1	429	370	351	374	331	305	346	327
Fbh1	3087	3235	3027	2974	3366	3134	3047	2967
Fbl	1649	2030	1861	1759	2034	1814	1568	1667
Fblim1	5246	5372	5543	5451	5318	5147	5199	4804
Fbll1	1	0	4	0	2	0	0	1
Fbln1	5892	5855	5199	5432	5486	5399	5874	5216
Fbln2	220	233	287	304	327	239	257	255
Fbln5	648	553	575	621	540	598	541	556
Fbln7	126	77	104	79	84	75	80	50
Fbn1	972	1167	1221	1178	1080	984	960	798
Fbn2	52	52	61	26	50	33	56	31
Fbp1	364	475	418	323	555	725	595	575
Fbp2	7018	8034	7340	6866	7344	8159	7491	7103
Fbrs	2054	2237	2129	2168	2163	2290	2199	2041
Fbrsl1	1882	1987	1993	1782	1819	1614	1656	1688
Fbxl12	485	484	507	561	539	442	512	371
Fbxl13	0	6	1	5	3	3	1	0
Fbxl14	1330	1585	1434	1293	1659	1488	1510	1538
Fbxl15	639	615	581	617	592	651	731	635
Fbxl16	14	24	34	41	34	26	9	14
Fbxl17	561	612	625	694	670	724	661	678
Fbxl18	860	834	995	865	825	920	866	827
Fbxl19	2542	2614	2293	2206	2907	2799	2475	2390
Fbxl2	8	23	6	5	19	7	5	6
Fbxl20	784	826	895	833	822	846	796	749

Transcriptome sequencing yielded total genetic results for the MOD and APS groups, with a total of 15,936 variables

Continued from above

Fbxl21	6	16	5	7	11	13	6	15
Fbxl22	199	217	245	206	304	265	229	226
Fbxl3	2871	3142	3157	2993	3137	3230	3569	3111
Fbxl4	327	425	330	360	308	321	341	304
Fbxl5	5175	5784	4829	4830	5195	5566	5192	4883
Fbxl6	744	724	777	725	700	661	624	621
Fbxl7	41	32	32	28	22	33	30	41
Fbxl8	531	386	544	515	525	438	462	403
Fbxo10	32	42	43	32	38	18	33	35
Fbxo11	1361	1387	1398	1413	1382	1558	1554	1441
Fbxo15	0	0	1	0	0	0	4	1
Fbxo16	0	0	2	2	0	0	4	0
Fbxo17	24	9	6	1	9	11	12	2
Fbxo2	11	3	25	12	19	11	15	5
Fbxo21	258	338	306	288	348	251	280	269
Fbxo22	1668	1801	1745	1721	1850	1869	1859	1758
Fbxo24	2	12	8	4	12	15	16	0
Fbxo25	4276	4548	4053	3756	4518	4865	4936	4257
Fbxo27	4	0	17	3	3	0	0	1
Fbxo28	1065	1287	1192	1118	1306	1201	1229	1161
Fbxo3	1766	1793	1905	1782	1768	1959	1942	1855
Fbxo30	622	651	634	710	626	631	737	619
Fbxo31	338	359	374	353	419	373	382	344
Fbxo32	180	167	101	133	128	136	184	123
Fbxo33	955	1001	1144	1094	1218	1185	1139	1045
Fbxo34	1830	1985	2027	1852	1998	2017	2115	2062
Fbxo36	97	90	107	94	117	91	99	134
Fbxo38	1944	2111	2021	1939	2140	2023	2029	1940
Fbxo39	1	6	4	0	3	3	1	0
Fbxo4	730	759	713	677	735	729	799	738
Fbxo40	1	1	1	1	9	4	1	0
Fbxo41	1	0	2	1	17	0	0	1
Fbxo42	1424	1570	1545	1471	1545	1761	1493	1499
Fbxo43	0	1	0	1	0	0	0	1
Fbxo44	514	551	425	340	448	408	457	502
Fbxo45	452	542	545	471	585	597	545	530
Fbxo46	424	384	444	392	472	370	402	376
Fbxo47	28	51	52	24	59	26	66	37
Fbxo48	27	24	22	20	40	26	21	39
Fbxo5	308	315	248	269	346	293	224	257
Fbxo6	1098	968	954	1081	1000	978	1014	1002
Fbxo7	501	504	589	519	548	538	502	460
Fbxo8	1983	2076	2041	1928	2054	1982	1966	1836
Fbxo9	1922	2045	1949	1725	2055	2001	2169	2045
Fbxw10	1	2	1	2	1	1	8	7
Fbxw11	3436	3836	3250	3408	3484	3588	3267	3296
Fbxw13	1	0	0	0	0	0	0	1
Fbxw15	0	0	0	0	1	0	0	1
Fbxw17	306	457	351	304	321	338	266	291
Fbxw2	1237	1277	1051	1171	1122	1092	1177	1060
Fbxw23	0	0	0	0	1	0	0	0
Fbxw24	4	0	0	0	1	0	0	0
Fbxw4	316	365	434	350	403	398	250	300
Fbxw5	1638	1832	1703	1700	1501	1507	1373	1418
Fbxw7	439	512	376	445	422	431	386	376
Fbxw8	2369	2423	2372	2245	2558	2281	2185	2257
Fbxw9	1910	1875	1805	1669	1946	2067	1761	1988
Fcamr	10	21	16	21	9	16	5	6

Transcriptome sequencing yielded total genetic results for the MOD and APS groups, with a total of 15,936 variables

Continued from above

Fcer1a	0	0	0	0	1	0	0	0
Fcer1g	385	354	361	392	321	278	343	281
Fcer2a	13	17	7	7	7	18	4	6
Fcf1	613	717	780	666	734	680	690	700
Fcgbp	131603	134927	143753	140940	121715	122513	113425	109394
Fcgr1	156	138	184	194	147	129	111	129
Fcgr2b	172	223	227	224	111	159	200	180
Fcgr3	499	590	469	621	532	525	528	527
Fcgr4	564	489	423	490	253	294	329	328
Fcgrt	5186	5260	5430	4894	5711	5771	5780	5208
Fcho1	106	103	93	78	114	58	101	94
Fcho2	2629	3014	2814	2717	2775	2884	3063	2626
Fchsd1	175	191	135	150	177	146	227	143
Fchsd2	1322	1586	1559	1267	1909	1906	1918	1683
Fcmr	0	0	1	0	0	1	0	0
Fcna	11	24	14	8	16	15	25	15
Fcor	4	0	0	0	1	2	1	0
Fcrl1	11	16	10	17	9	13	19	6
Fcrl5	3	3	0	0	1	0	1	0
Fcrla	33	37	29	26	27	16	36	25
Fcrlb	1	4	0	8	6	0	1	7
Fcrls	4	20	15	23	20	12	22	24
Fcsk	1950	1867	1792	1939	1739	1643	1565	1633
Fdft1	2226	2318	2765	2644	2243	2316	2073	2338
Fdps	3475	3432	3826	3715	3357	3460	3117	3399
Fdx1	1313	1322	1306	1276	1130	1170	1075	1026
Fdx2	421	432	365	455	418	399	360	458
Fdxacb1	272	324	248	273	273	250	229	270
Fdxr	342	365	363	399	337	418	370	329
Fech	1628	1742	1713	1788	1723	1806	1643	1689
Fem1a	2569	2731	2602	2629	2844	2720	2371	2364
Fem1al	0	0	0	1	0	1	0	0
Fem1b	1839	1919	1871	1819	1959	1890	1847	1828
Fem1c	789	996	859	999	938	985	908	868
Fen1	1410	1453	1342	1317	1351	1138	1132	1197
Fer	519	561	583	659	457	546	497	458
Fer1l4	516	334	488	649	151	170	169	221
Fer1l5	4	5	2	3	5	1	0	1
Fer1l6	962	940	1076	1080	909	908	939	857
Fermt1	2072	2183	2130	2081	2278	2325	2149	2023
Fermt2	549	548	634	691	626	637	571	470
Fermt3	232	219	251	276	242	224	212	217
Fes	206	276	202	227	234	200	252	215
Fetub	1	8	7	7	22	22	8	18
Fev	42	32	46	24	40	45	50	28
Fez1	7	25	17	17	27	25	13	24
Fez2	2360	2385	2192	2074	2066	2252	2411	2278
Fezf1	0	0	2	0	4	0	1	0
Ffar1	8	1	7	13	5	5	9	3
Ffar2	135	101	113	94	109	112	121	91
Ffar3	2	2	11	15	11	13	9	12
Ffar4	144	120	184	156	178	150	146	143
Fga	20	42	35	28	82	40	25	44
Fgb	46	56	31	12	70	40	35	29
Fgd1	75	37	65	75	47	43	45	30
Fgd2	224	169	171	168	171	174	161	164
Fgd3	48	74	75	64	95	72	61	68
Fgd4	2294	2259	2157	2072	2355	2549	2590	2407

Transcriptome sequencing yielded total genetic results for the MOD and APS groups, with a total of 15,936 variables

Continued from above

Fgd5	183	178	138	148	165	146	176	143
Fgd6	948	1086	972	961	1059	1067	1041	1052
Fgf1	271	228	261	217	295	284	251	201
Fgf10	21	34	34	15	28	19	21	15
Fgf11	123	133	147	148	175	211	129	158
Fgf12	1	1	10	3	0	6	8	1
Fgf13	54	51	42	43	32	69	22	62
Fgf14	9	40	38	41	25	14	17	22
Fgf16	3	0	11	6	13	5	1	5
Fgf17	1	0	0	0	0	0	0	0
Fgf18	6	0	1	0	1	0	4	1
Fgf2	0	5	4	5	2	3	6	3
Fgf21	1	0	0	0	6	0	1	0
Fgf22	0	0	0	1	0	0	0	0
Fgf3	0	0	0	0	0	0	1	0
Fgf4	0	1	0	4	8	4	0	2
Fgf5	0	0	0	0	1	0	0	0
Fgf7	34	33	47	40	47	40	46	54
Fgf8	0	0	0	0	0	0	2	0
Fgf9	155	123	93	164	91	122	130	101
Fgfbp1	483	607	610	462	528	557	501	570
Fgfbp3	17	33	19	26	16	31	13	26
Fgfr1	281	269	321	302	306	272	310	283
Fgfr1op2	2646	2882	2816	2807	3000	2969	2886	2809
Fgfr2	1087	1219	1192	1196	965	927	1007	984
Fgfr3	441	463	528	467	375	377	318	293
Fgfr4	79	83	115	84	100	88	66	98
Fgfrl1	211	211	253	199	161	159	210	194
Fgg	21	84	14	25	84	54	22	22
Fggy	43	34	61	40	36	28	38	46
Fgl1	18	34	16	30	37	26	35	44
Fgl2	2266	2289	2054	2254	2141	2185	2298	2033
Fgr	143	150	129	150	94	114	76	77
Fh1	6251	6218	6453	6501	6375	6165	6011	6144
Fhad1	7	15	9	9	14	20	26	23
Fhdc1	379	372	392	330	488	575	445	486
Fhit	56	44	53	42	34	37	31	18
Fhl1	1366	1410	1690	1676	1472	1502	1353	1397
Fhl2	2502	2283	2450	2775	1431	1454	1750	1828
Fhl3	42	38	26	39	23	49	51	46
Fhl4	8	9	4	8	0	4	8	7
Fhl5	5	10	4	7	17	17	12	3
Fhod1	498	475	548	596	670	568	526	536
Fhod3	25	9	16	31	37	31	21	24
Fibcd1	10	3	14	15	4	10	11	2
Fibin	54	43	38	31	45	52	56	50
Fibp	1449	1439	1297	1549	1350	1334	1264	1292
Ficd	269	234	240	258	329	230	283	270
Fig4	972	1006	975	901	921	845	926	944
Fign	3	10	2	4	4	1	2	5
Fignl1	387	413	358	286	400	389	333	325
Fignl2	341	328	253	285	359	322	327	327
Filip1	70	94	105	100	88	65	88	75
Filip1l	702	744	782	855	683	761	724	683
Fip1l1	1507	1651	1416	1512	1675	1769	1601	1536
Fis1	4054	4089	4007	4062	3869	4076	3957	3911
Fitm1	0	0	1	6	0	1	0	4
Fitm2	343	354	305	340	406	333	282	415

Transcriptome sequencing yielded total genetic results for the MOD and APS groups, with a total of 15,936 variables

Continued from above

Fiz1	1035	1081	1076	1145	1126	1121	998	996
Fjx1	21	21	27	33	36	23	12	14
Fkbp10	130	169	192	172	162	158	106	110
Fkbp11	170	226	216	236	321	307	255	295
Fkbp14	55	95	84	89	98	104	79	79
Fkbp15	1590	1531	1524	1555	1587	1577	1467	1364
Fkbp1a	5780	5970	6139	5928	6008	5723	5548	5473
Fkbp1b	12	6	3	5	11	8	5	12
Fkbp2	0	2	2	0	0	3	0	2
Fkbp3	695	742	689	669	709	714	691	721
Fkbp4	10106	11584	11089	10199	12754	12466	10451	10370
Fkbp5	1330	1565	1987	1995	2175	2313	2928	3167
Fkbp6	0	0	0	1	0	0	0	0
Fkbp7	84	67	72	99	82	63	103	88
Fkbp8	11091	11128	10713	10645	11652	11774	11146	10825
Fkbp9	603	588	563	563	624	614	550	527
Fkbpl	196	193	165	159	123	159	162	176
Fkrp	672	796	710	679	699	679	727	797
Fktn	406	475	418	442	491	504	560	425
Flacc1	171	156	168	247	162	207	207	182
Flad1	1680	1743	1628	1596	1731	1489	1515	1396
Flcn	833	858	857	851	751	679	746	797
Fli1	81	100	101	74	107	114	86	74
Flii	5747	6331	5900	6106	5815	5784	5703	5772
Flna	5563	5879	6470	6304	5985	6008	6158	5651
Flnb	11374	11849	11792	12146	12437	11937	12201	11449
Flnc	430	432	454	490	389	380	399	379
Flot1	1521	1586	1613	1695	1496	1511	1472	1559
Flot2	660	665	693	753	708	686	774	638
Flrt1	9	5	18	4	4	5	8	1
Flrt2	21	4	8	8	13	38	23	12
Flrt3	1027	764	871	976	792	791	746	719
Flt1	664	762	628	670	637	717	588	573
Flt3	10	8	1	6	13	11	1	8
Flt3l	111	92	150	120	121	107	116	102
Flt4	301	335	341	356	303	282	307	252
Flvcr1	2489	3063	2778	2327	4414	4361	4284	3920
Flvcr2	315	327	255	199	282	281	289	284
Flywch1	1268	1219	1363	1247	1211	1167	1217	1173
Flywch2	63	68	101	56	75	72	80	61
Fmc1	768	643	756	851	686	627	756	626
Fmn1	2069	2194	1910	1921	2142	2395	2545	2183
Fmn2	1	1	2	10	3	1	1	4
Fmnl1	178	120	156	152	102	184	104	122
Fmnl2	280	293	276	280	242	232	218	222
Fmnl3	182	181	187	196	181	210	179	152
Fmo1	319	305	248	192	397	364	333	324
Fmo2	304	388	398	381	426	462	507	387
Fmo3	1	2	1	0	0	3	3	0
Fmo4	1701	2032	1771	1749	1979	1992	1936	1749
Fmo5	11158	11224	11662	12249	11310	11721	11603	10788
Fmod	17	12	4	13	12	18	20	20
Fmr1	1708	1794	1699	1616	1839	1791	1800	1623
Fmr1nb	35	77	43	19	25	20	2	2
Fn1	3865	3976	4097	4127	3667	3671	3329	2961
Fn3k	6	2	6	1	3	0	2	3
Fn3krp	153	123	124	146	150	164	130	172
Fnbp1	712	765	740	765	682	683	580	637

Transcriptome sequencing yielded total genetic results for the MOD and APS groups, with a total of 15,936 variables

Continued from above

Fnbp1l	3783	3988	3864	4173	4221	4434	4518	4163
Fnbp4	961	1033	983	1008	988	1008	875	860
Fndc1	137	153	165	122	130	141	144	129
Fndc10	475	409	389	415	292	346	287	395
Fndc11	7	4	0	1	1	1	1	4
Fndc3a	4949	5681	4989	4773	5395	5421	5234	4892
Fndc3b	884	1026	951	905	1054	1139	1000	1001
Fndc3c1	0	0	0	0	0	1	0	0
Fndc4	13	21	16	3	13	13	8	10
Fndc5	13	11	7	7	10	11	10	8
Fndc7	0	0	1	3	0	6	8	4
Fndc8	1	0	2	2	4	2	1	3
Fnip1	1300	1386	1322	1425	1186	1237	1445	1348
Fnip2	316	285	278	382	310	350	347	319
Fnta	1602	1682	1702	1677	1775	1738	1519	1547
Fntb	483	425	415	431	495	481	409	463
Focad	436	555	463	460	550	478	445	429
Folh1	0	0	0	0	1	0	0	0
Folr1	1	0	0	0	3	1	1	6
Folr2	7	0	5	7	6	1	12	4
Fos	3383	3527	4389	4014	2999	3257	3780	3407
Fosb	187	156	214	203	162	232	242	210
Fosl1	9	9	11	18	11	6	16	17
Fosl2	6104	6706	6278	6299	5935	5957	6325	6289
Foxa1	473	429	430	463	468	429	477	453
Foxa2	87	116	131	94	103	116	91	109
Foxa3	522	604	585	553	673	578	567	643
Foxc1	0	0	1	0	1	0	4	0
Foxc2	0	5	0	0	6	1	6	0
Foxd2	1	2	0	5	3	2	1	4
Foxd3	23	18	15	25	8	20	31	29
Foxf1	549	616	541	580	442	515	465	412
Foxf2	231	222	235	219	243	228	196	185
Foxh1	12	2	4	6	0	0	0	11
Foxi1	0	1	0	0	0	0	0	0
Foxj1	1	0	4	2	0	0	1	0
Foxj2	470	537	568	511	492	639	549	430
Foxj3	2137	2002	2001	2072	1948	1940	2233	1920
Foxk1	2076	2114	2094	2098	2133	2275	2010	1838
Foxk2	1296	1474	1559	1451	1832	1565	1540	1483
Foxl1	74	129	54	72	118	46	85	48
Foxl2	0	0	4	0	0	0	0	0
Foxm1	667	660	706	645	730	649	544	526
Foxn1	4	0	0	0	1	0	0	0
Foxn2	1077	1087	1123	1093	1005	1061	1150	1131
Foxn3	738	774	779	866	839	896	884	759
Foxo1	1355	1358	1321	1196	1281	1288	1429	1388
Foxo3	2303	2551	2385	2327	2736	2664	2708	2406
Foxo4	1103	961	1109	1016	1121	1083	1042	966
Foxo6	246	195	169	250	102	143	118	154
Foxp1	968	1092	1151	1051	929	865	884	842
Foxp2	23	45	10	42	20	35	34	16
Foxp3	14	20	27	3	10	16	8	10
Foxp4	1954	2059	1908	1990	1771	1781	1671	1665
Foxq1	1055	1194	1172	1068	958	869	889	859
Foxred1	1226	1255	1417	1293	1237	1160	1082	1117
Foxred2	46	23	8	18	25	20	21	9
Foxs1	14	24	7	11	12	12	8	10

Transcriptome sequencing yielded total genetic results for the MOD and APS groups, with a total of 15,936 variables

Continued from above

Fpgs	405	388	421	333	463	397	326	431
Fpgt	806	852	743	812	954	901	875	792
Fpr1	27	12	8	15	2	22	17	8
Fpr2	20	17	11	14	3	5	17	15
Fra10ac1	231	259	298	205	260	214	250	233
Fras1	25	25	33	12	24	38	23	24
Frat1	1145	1148	1148	1023	944	938	860	1023
Frat2	1540	1743	1694	1734	1358	1526	1316	1489
Frem1	14	4	10	9	20	15	27	3
Frem2	79	73	75	97	77	84	54	60
Frem3	0	0	0	0	0	2	0	0
Frg1	857	995	909	964	958	985	891	881
Frg2f1	39	30	22	26	33	48	44	19
Frk	6682	7050	6312	6429	6864	7367	7806	6873
Frmd3	87	46	62	46	32	55	61	53
Frmd4a	207	335	227	248	220	258	274	216
Frmd4b	701	783	731	756	767	835	664	721
Frmd5	3	17	18	25	13	7	4	17
Frmd6	219	254	255	287	284	270	264	200
Frmd7	5	0	1	1	0	1	0	2
Frmd8	6166	6502	6110	5888	6715	6875	6892	6425
Frmpd1	1	3	5	0	0	0	5	1
Frmpd2	0	0	0	0	0	4	0	6
Frmpd3	3	1	6	0	1	1	5	1
Frmpd4	3	6	4	8	2	3	11	2
Frrs1	800	993	873	862	925	896	829	817
Frrs1l	1	0	0	1	0	0	4	0
Frs2	1452	1628	1669	1556	1597	1586	1785	1489
Frs3	106	81	94	82	73	84	100	96
Fry	447	433	507	598	463	460	458	486
Fryl	4571	4733	5113	5240	4449	4717	4479	4141
Frzb	45	62	64	75	73	83	63	56
Fsbp	16	2	1	5	6	2	1	0
Fscn1	236	201	202	280	193	215	199	167
Fscn2	3	1	0	0	0	0	0	1
Fscn3	29	3	13	3	15	16	21	9
Fsd1	9	19	24	20	35	18	16	46
Fsd1l	643	672	749	672	790	756	704	666
Fsd2	6	6	0	2	10	4	7	3
Fsip1	1	0	2	0	5	6	1	5
Fst	107	121	134	115	110	136	133	113
Fstl1	840	1100	1121	1034	901	859	887	858
Fstl3	83	134	144	86	109	90	91	128
Fstl4	11	3	22	4	11	5	11	7
Fstl5	2	1	2	6	0	5	6	0
Ftcd	0	2	1	0	1	0	3	1
Fth1	106594	115154	103221	99067	117596	125050	113920	109849
Ftl1	44727	45841	44469	44329	48514	47783	45338	46748
Ftl1-ps2	4	5	4	3	4	5	4	5
Fto	931	1079	948	1002	838	1025	892	786
Ftsj1	437	498	481	484	590	560	457	455
Ftsj3	1039	1185	1090	935	1258	1024	1111	1097
Fubp1	2184	2342	2260	2196	2877	2652	2294	2343
Fubp3	1546	1594	1573	1581	1433	1454	1387	1326
Fuca1	10066	10691	9775	9498	9795	10330	10214	10094
Fuca2	7473	8366	7121	6859	7692	8455	8767	7692
Fundc1	490	520	473	556	654	615	559	461
Fundc2	549	708	672	600	560	623	521	627

Transcriptome sequencing yielded total genetic results for the MOD and APS groups, with a total of 15,936 variables

Continued from above

Fuom	1819	1855	1839	1964	1812	1985	1836	1695
Furin	4957	5390	4808	4676	4871	5109	4939	4657
Fus	4315	4795	4304	4389	3374	3431	3418	3414
Fut1	9	27	16	29	15	30	43	30
Fut10	22	13	25	13	18	29	22	12
Fut11	290	270	283	274	292	260	290	272
Fut2	1407	802	931	1645	1927	1768	1809	2151
Fut4	1320	1594	1672	1612	1364	1315	1595	1527
Fut7	35	27	43	24	48	41	53	26
Fut8	3430	3558	3717	3745	3714	3663	3430	3510
Fuz	150	146	138	127	114	94	131	146
Fv1	90	71	69	77	125	92	99	101
Fxn	282	348	320	356	335	327	306	332
Fxr1	1393	1388	1197	1340	1565	1612	1409	1433
Fxr2	1672	1696	1562	1532	1693	1765	1610	1594
Fxyd1	83	97	93	106	88	110	82	130
Fxyd2	11	7	8	4	12	9	1	17
Fxyd3	491	473	586	516	462	585	453	507
Fxyd5	212	166	184	199	123	162	177	153
Fxyd6	209	275	302	269	283	242	332	250
Fxyd7	11	3	8	11	5	8	9	4
Fyb	406	406	348	383	319	326	337	295
Fyb2	66	38	32	32	36	52	14	57
Fyco1	959	1145	1098	905	982	1018	937	891
Fyn	267	324	309	369	257	228	248	215
Fyttd1	1144	1126	1154	1179	1268	1145	1221	1082
Fzd1	220	192	215	240	163	202	157	130
Fzd10	5	3	0	0	5	4	0	0
Fzd2	145	136	155	134	162	162	122	125
Fzd3	16	13	13	13	13	25	16	16
Fzd4	946	1018	928	926	827	853	919	830
Fzd5	5447	6296	6011	5648	5086	5454	5030	5018
Fzd6	195	213	196	131	205	169	134	161
Fzd7	827	953	632	733	768	942	876	651
Fzd8	148	132	153	92	105	98	97	72
Fzd9	20	18	10	34	3	19	18	18
Fzr1	3001	2744	2738	2755	2846	2824	2862	2859
G0s2	69	71	94	54	97	100	124	88
G2e3	722	749	690	706	812	709	670	693
G3bp1	4613	4904	4917	4303	5391	5460	4590	4526
G3bp2	6901	8513	7441	6680	7480	7670	7619	6877
G6pc	1054	1519	1123	619	2245	2354	1987	1593
G6pc2	0	0	0	0	0	1	0	0
G6pc3	758	877	792	879	992	840	722	860
G6pdx	3929	3983	3756	3988	3570	3391	3389	3279
Gaa	1176	1224	1009	1048	1032	1073	987	1021
Gab1	740	871	660	621	760	753	760	681
Gab2	970	1118	1143	1124	838	905	841	804
Gab3	3	1	4	11	5	8	1	11
Gabarap	12203	12487	11947	12058	11978	12799	12459	12467
Gabarapl1	746	626	641	746	667	697	694	627
Gabarapl2	2493	2507	2300	2551	2589	2714	2490	2568
Gabbr1	175	133	146	139	157	118	174	113
Gabbr2	18	7	3	0	6	4	0	6
Gabpa	1801	2018	1965	1846	1851	1974	1951	1767
Gabpb1	744	814	769	689	749	716	696	603
Gabpb2	1853	2290	2359	2135	2328	2305	2254	1892
Gabra1	0	4	1	1	4	0	1	0

Transcriptome sequencing yielded total genetic results for the MOD and APS groups, with a total of 15,936 variables

Continued from above

Gabra2	0	0	0	0	0	9	0	0
Gabra3	83	98	96	83	78	75	102	74
Gabra4	0	3	0	1	1	2	6	3
Gabra5	0	0	0	1	0	0	0	0
Gabrb1	2	0	4	0	2	3	0	1
Gabrb2	0	4	1	0	0	0	3	0
Gabrb3	4	11	14	18	17	10	12	9
Gabrd	7	10	13	12	10	2	5	29
Gabre	7	5	14	2	6	7	9	13
Gabrg1	0	0	0	0	1	0	0	0
Gabrg2	0	4	0	1	0	0	2	0
Gabrg3	4	0	1	1	0	2	0	6
Gabrq	0	0	0	0	0	1	1	0
Gabrr1	0	0	12	0	0	0	1	0
Gabrr2	0	5	1	1	0	6	4	5
Gad1	0	0	0	0	0	1	1	4
Gad2	4	6	3	11	7	1	0	3
Gadd45a	1512	1539	1624	1605	1512	1779	1791	1511
Gadd45b	578	648	785	742	712	792	630	574
Gadd45g	392	373	394	495	233	285	310	362
Gadd45gi|	1264	1261	1248	1086	1181	1210	1129	1094
Gak	8827	9067	8092	8401	8599	8531	8554	7826
Gal	28	38	33	24	78	55	54	68
Gal3st1	2592	2638	2721	2542	2292	2306	2209	2332
Gal3st2	4078	4622	4060	3478	3620	3854	4349	3905
Gal3st2b	330	418	296	337	361	421	473	484
Gal3st2c	28	25	14	9	52	28	19	18
Gal3st3	1	0	0	0	0	0	0	0
Gal3st4	12	12	8	4	3	7	14	3
Galc	155	180	161	186	141	127	145	153
Gale	7371	7267	7091	7344	7317	6934	6983	6522
Galk1	1471	1504	1515	1519	1516	1386	1511	1395
Galk2	1624	1598	1628	1682	1827	1811	1650	1644
Galm	6777	6999	6899	7061	6716	6796	7353	6535
Galns	1029	1094	1070	944	1008	997	1027	945
Galnt1	10524	11571	10593	10036	11411	11877	11759	10518
Galnt10	5214	5694	5155	5028	5550	5563	5226	5133
Galnt11	507	589	493	473	448	511	534	501
Galnt12	972	966	1051	1170	1119	1008	909	947
Galnt13	0	6	0	2	0	0	0	0
Galnt14	0	0	0	0	0	0	0	1
Galnt15	59	43	38	20	37	39	30	20
Galnt16	20	17	19	30	14	19	10	9
Galnt17	5	15	5	5	4	6	1	1
Galnt18	69	83	44	81	84	67	58	77
Galnt2	3637	3921	3585	3622	4022	3857	3410	3788
Galnt3	1766	1769	1670	1700	2032	2048	1796	1889
Galnt4	3655	4043	3693	3813	4133	4164	4147	3950
Galnt5	217	246	213	198	212	225	250	216
Galnt6	10100	11047	10662	10150	12546	12952	12581	11432
Galnt7	3262	3582	3183	3207	3611	3437	3559	3465
Galnt9	0	0	0	1	0	0	2	0
Galntl6	17	12	18	6	11	15	5	10
Galr1	4	1	18	2	5	14	6	3
Galr2	17	18	4	22	2	17	21	24
Galr3	44	46	37	56	38	29	51	33
Galt	1704	1543	1449	1643	1481	1435	1457	1332
Gamt	73	54	55	79	111	72	104	61

Transcriptome sequencing yielded total genetic results for the MOD and APS groups, with a total of 15,936 variables

Continued from above

Gan	324	335	341	405	263	316	323	358
Ganab	10506	11965	10734	10513	11456	11422	10788	10557
Ganc	711	698	555	594	646	744	728	638
Gap43	40	53	48	52	49	27	44	63
Gapdh	6710	6641	6657	6998	7132	7197	7431	7286
Gapdhs	6	3	6	4	1	6	3	5
Gapt	5	5	7	6	7	4	6	2
Gapvd1	2250	2622	2316	2345	2551	2463	2400	2288
Gar1	580	689	596	539	685	643	537	470
Garem1	273	332	261	347	312	387	387	251
Garem2	0	0	0	0	0	4	0	0
Garnl3	28	35	38	20	17	27	14	39
Garre1	799	1110	997	953	930	1029	878	807
Gars	4282	4693	4565	4453	4245	4022	4041	4157
Gart	1558	1553	1474	1431	1599	1594	1343	1505
Gas1	176	182	205	161	255	234	242	228
Gas2	119	114	135	139	96	140	140	114
Gas2l1	2368	2561	2359	2336	2686	2365	2359	2333
Gas2l2	2	1	5	14	5	8	4	8
Gas2l3	266	244	256	228	308	287	368	299
Gas6	2602	2570	2632	2600	2306	2456	2250	2121
Gas7	234	249	180	239	248	242	219	203
Gas8	112	117	102	142	102	162	102	123
Gask1a	6	1	1	2	8	9	6	1
Gask1b	213	215	220	174	160	180	208	194
Gast	0	1	0	1	0	4	1	1
Gata1	1	0	0	0	1	0	0	5
Gata2	47	13	39	47	37	29	32	28
Gata3	10	8	2	11	15	4	8	8
Gata4	8028	8691	7838	7874	8727	8876	8183	7600
Gata5	3829	3849	3726	3902	3968	3972	4087	3398
Gata6	5093	5144	5004	4466	4526	4605	4694	4674
Gatad1	2126	1913	2149	1985	1919	2003	1850	1866
Gatad2a	3928	3840	3749	3495	3619	3752	3593	3554
Gatad2b	1056	1136	1107	1142	1080	1176	1104	1001
Gatb	521	552	549	568	582	515	484	490
Gatc	510	587	491	497	581	556	550	603
Gatd1	651	707	624	655	694	707	643	713
Gatd3a	1366	1456	1450	1436	1357	1377	1237	1353
Gatm	152	198	105	193	251	179	181	212
Gba	1871	1790	1869	1759	1904	1910	1879	1787
Gba2	266	304	286	257	284	344	288	269
Gbe1	175	197	169	205	186	215	192	174
Gbf1	6015	6814	5726	5762	7161	6914	6923	6355
Gbgt1	289	332	427	324	476	371	319	348
Gbp10	93	103	72	103	52	53	24	29
Gbp2	1881	1872	1588	1867	830	1050	907	848
Gbp2b	332	315	274	358	384	453	412	385
Gbp3	1177	1433	1158	898	1173	1147	1096	839
Gbp4	599	695	596	693	366	443	335	310
Gbp5	414	437	414	449	253	253	314	242
Gbp6	1217	1360	1043	919	685	926	737	563
Gbp7	1235	1592	1307	1172	1002	1242	958	895
Gbp8	241	258	250	306	223	200	163	187
Gbp9	514	537	575	529	394	405	351	333
Gbx2	6	0	0	1	0	0	0	1
Gc	73	54	40	23	59	46	39	38
Gca	595	807	702	713	677	695	602	640

Transcriptome sequencing yielded total genetic results for the MOD and APS groups, with a total of 15,936 variables

Continued from above

Gcat	2203	2235	2208	2119	2624	2337	2073	2295
Gcc1	1019	1075	913	866	1010	1045	960	923
Gcc2	3181	3797	3034	3095	3381	3657	3790	3165
Gcdh	1439	1281	1389	1413	1311	1406	1262	1356
Gcfc2	148	138	129	134	129	123	101	114
Gcg	570	514	610	601	536	531	538	442
Gcgr	13	15	9	21	18	8	2	18
Gch1	1869	2218	2289	1972	2176	2151	2029	2277
Gchfr	940	992	924	1009	739	804	823	1018
Gck	21	12	34	24	26	34	32	25
Gckr	1	1	0	2	1	1	0	1
Gclc	1340	1337	1240	1369	1463	1442	1242	1380
Gclm	3623	3579	3658	3711	4144	4281	4034	4022
Gcn1	2802	3171	3047	2756	3159	2957	2902	2686
Gcnt1	102	98	62	80	59	64	85	79
Gcnt2	2137	2694	2415	2212	2484	2500	2475	2437
Gcnt3	5399	5342	5856	6679	4645	4445	5174	5533
Gcnt4	2716	3147	3282	2976	3225	2988	2768	2723
Gcnt7	0	1	2	0	0	1	0	0
Gcsam	4	1	1	8	7	0	0	1
Gcsh	1350	1286	1180	1334	1463	1307	1242	1333
Gda	63248	65881	55949	59534	58861	65670	71717	65562
Gdap1	14	20	4	6	15	8	3	8
Gdap1l1	3	5	10	14	12	20	14	20
Gdap2	911	902	905	906	919	979	875	938
Gde1	4746	4957	4671	4389	5137	5419	5361	4432
Gdf10	4	40	21	17	11	14	34	27
Gdf11	31	35	19	25	32	29	23	32
Gdf15	29	14	43	36	41	41	58	43
Gdf2	0	0	0	1	0	0	0	0
Gdf3	7	20	21	8	23	20	30	14
Gdf5	1	0	1	0	0	0	0	0
Gdf6	5	1	2	3	4	7	9	9
Gdf7	1	0	0	0	0	0	0	0
Gdf9	33	18	42	17	25	53	43	47
Gdi1	2179	2346	2172	2237	2047	2152	2223	2028
Gdi2	18791	19485	18326	18624	19435	19640	19611	18339
Gdnf	54	43	41	19	63	32	19	10
Gdpd1	4968	5741	5080	4722	6071	6132	5978	5861
Gdpd2	3622	3609	3382	3782	2887	2953	3609	3163
Gdpd3	44	34	67	42	53	30	50	56
Gdpd5	710	770	817	703	695	805	689	629
Gdpgp1	396	353	456	413	460	435	354	340
Gem	145	194	223	157	99	147	108	149
Gemin2	179	215	162	189	184	190	177	165
Gemin4	347	350	373	328	377	378	375	335
Gemin5	640	661	569	602	611	704	584	605
Gemin6	238	255	222	246	275	313	261	283
Gemin6-p	0	0	1	0	0	0	0	0
Gemin7	1269	1214	1159	1262	1256	1165	1099	1134
Gemin8	59	33	73	58	40	37	59	48
Gen1	229	250	200	202	181	246	193	196
Get1	146	164	138	160	155	127	148	139
Get3	2178	2189	2251	2167	2254	2256	2086	2251
Get4	1892	1807	1918	1748	1625	1702	1721	1735
Gfap	22	4	36	21	4	14	20	16
Gfer	712	952	956	732	908	876	815	837
Gfi1	163	141	152	185	134	106	149	142

Transcriptome sequencing yielded total genetic results for the MOD and APS groups, with a total of 15,936 variables

Continued from above

Gfi1b	0	3	4	0	1	11	4	6
Gfm1	2946	2842	2772	3178	2915	2804	2427	2509
Gfm2	1549	1690	1755	1712	1750	1943	1600	1573
Gfod1	241	304	252	304	303	321	369	312
Gfod2	485	630	688	532	705	684	662	575
Gfpt1	14921	16094	14755	14768	16438	16540	16068	15494
Gfpt2	13	29	21	24	35	16	23	25
Gfra1	35	31	24	37	16	36	44	24
Gfra2	29	31	34	48	47	24	20	23
Gfra3	56	69	40	85	56	61	72	43
Gfra4	4	9	13	12	17	19	11	10
Gfus	3742	4100	3972	4041	3670	3783	3563	3565
Gga1	3760	4079	3575	3580	3671	3740	3443	3454
Gga2	231	230	255	230	282	213	188	229
Gga3	1058	988	1037	987	1064	933	899	863
Ggact	2267	2239	2458	2540	2195	2172	2127	1745
Ggct	130	97	105	108	140	172	107	133
Ggcx	278	337	352	363	455	396	325	416
Ggh	448	353	301	394	507	505	486	493
Ggn	4	12	10	5	17	2	11	11
Ggnbp1	19	32	10	10	28	18	10	29
Ggnbp2	3705	3964	3640	3650	3821	4087	4197	3864
Ggps1	809	943	842	896	929	877	934	834
Ggt1	26643	27812	25243	24489	26606	28437	29332	27544
Ggt5	193	211	233	244	185	210	189	219
Ggt6	1463	1332	1143	1205	1258	1198	1153	1266
Ggt7	30	21	10	33	20	27	13	27
Ggta1	202	296	239	272	289	315	331	295
Ghdc	664	606	622	670	662	689	674	613
Ghitm	15705	15948	15220	15950	15779	16213	16531	15266
Ghr	283	287	256	278	278	329	312	302
Ghrh	0	0	0	0	1	0	0	0
Ghrhr	0	0	0	2	4	0	0	0
Ghrl	550	551	595	644	513	571	489	508
Gid4	1055	979	1083	906	1018	1070	966	1010
Gid8	2099	2228	2045	2169	2009	2083	1925	2048
Gigyf1	482	525	446	474	486	388	393	350
Gigyf2	2621	2778	2715	2795	2783	2650	2790	2484
Gimap1	397	427	449	337	392	408	329	345
Gimap3	202	160	232	224	220	211	172	214
Gimap4	214	245	254	236	258	267	195	213
Gimap5	103	106	92	145	128	123	103	104
Gimap6	347	384	301	293	335	290	306	224
Gimap7	91	82	59	90	61	92	67	59
Gimap8	158	88	160	140	102	96	94	132
Gimap9	107	99	99	74	138	119	86	89
Gimd1	1739	2192	2053	1528	1964	1988	2117	1769
Gin1	309	310	350	323	308	364	349	284
Ginm1	1978	2036	2025	1966	1958	2039	1897	1975
Gins1	212	244	277	222	195	232	168	183
Gins2	337	360	338	383	426	328	272	309
Gins3	130	117	84	85	109	86	97	107
Gins4	493	425	423	399	467	441	443	400
Gip	1675	1772	1931	1799	1327	1364	1368	1243
Gipc1	1547	1585	1607	1623	1656	1474	1259	1369
Gipc2	8301	8317	7752	7995	8247	8470	8091	7522
Gipc3	27	25	17	20	9	17	12	20
Gipr	9	16	7	15	5	14	5	14

Transcriptome sequencing yielded total genetic results for the MOD and APS groups, with a total of 15,936 variables

Continued from above

Git1	3805	3909	3893	3820	3941	3965	3792	3839
Git2	629	616	790	711	708	783	654	621
Gja1	554	588	655	662	549	484	495	466
Gja4	61	45	60	78	65	37	58	37
Gja5	28	31	24	46	66	17	33	20
Gja6	4	0	1	0	1	0	0	1
Gjb1	3616	3371	3184	3613	3260	3222	3139	3081
Gjb2	641	716	702	589	603	596	626	585
Gjb3	222	196	225	172	262	220	197	201
Gjb4	4	0	1	1	0	1	0	1
Gjb5	0	0	0	9	1	1	1	2
Gjc1	87	55	53	121	69	63	73	53
Gjc2	48	57	37	49	54	33	31	46
Gjc3	1	0	1	2	2	2	7	2
Gjd2	0	1	4	2	1	0	1	2
Gjd3	5	2	14	6	5	7	1	3
Gjd4	0	0	0	0	0	0	1	0
Gk	11284	10632	10461	12253	8221	8921	10121	9007
Gk5	1253	1274	1239	1377	1237	1391	1242	1237
Gkap1	92	70	91	102	93	86	85	65
Gkn1	1	0	0	0	0	0	1	0
Gkn2	0	0	1	0	1	0	0	0
Gkn3	49	80	67	52	100	58	38	60
Gla	427	435	365	430	429	476	442	420
Glb1	1125	1108	1095	1124	1161	1180	1160	1048
Glb1l	85	85	120	131	107	97	98	125
Glb1l2	2	0	10	1	3	15	3	2
Glcci1	428	489	447	367	410	444	387	384
Glce	1575	1124	1546	1823	892	820	978	1046
Gldc	4	1	5	10	3	4	1	3
Gldn	1	0	0	0	0	0	0	1
Gle1	873	979	949	803	934	1006	836	929
Glg1	3710	3996	3837	3600	3933	3916	3696	3172
Gli1	73	86	114	84	100	95	50	72
Gli2	43	39	43	68	38	23	33	39
Gli3	30	34	27	35	21	45	20	36
Glipr1	358	405	356	440	454	460	412	422
Glipr2	613	590	639	720	515	491	499	432
Glis1	2	5	4	2	16	1	1	1
Glis2	126	95	124	127	109	126	138	107
Glis3	43	55	70	52	61	41	31	45
Glmn	52	116	133	100	81	95	61	60
Glmp	3107	3260	3156	3139	3151	2883	2858	3124
Glo1	3423	3688	3471	3494	3789	3737	3668	3623
Glod4	1506	1785	1701	1679	1873	1799	1857	1716
Glod5	5119	5740	5109	4975	4931	5700	5482	4720
Glp1r	1	18	3	10	2	18	8	1
Glp2r	211	223	195	212	231	215	180	192
Glrb	5	6	6	19	2	6	19	0
Glrp1	0	0	0	1	0	1	0	0
Glrx	13689	15615	13106	12301	13088	14359	14036	12806
Glrx2	606	660	705	774	704	590	529	637
Glrx3	2505	2617	2540	2514	2739	2883	2536	2592
Glrx5	2959	2889	2697	2872	2804	2649	2563	2674
Gls	3537	3746	3714	4112	4089	4089	4017	3789
Gls2	125	95	102	74	131	119	76	127
Glt1d1	29	29	22	33	16	17	31	47
Glt28d2	615	645	645	621	691	694	736	703

Transcriptome sequencing yielded total genetic results for the MOD and APS groups, with a total of 15,936 variables

Continued from above

Glt8d1	240	389	366	361	389	323	355	323
Glt8d2	5	9	17	11	9	6	36	6
Gltp	4907	4908	4881	4997	4219	4166	4322	4239
Gltpd2	138	108	150	147	108	132	145	119
Glud1	8670	9432	9349	8693	9415	9786	9989	9138
Glul	1502	1553	1865	1686	1750	1881	1631	1707
Glyat	1	0	1	0	3	5	1	2
Glyatl3	0	0	1	0	0	4	0	0
Glyctk	688	776	668	676	812	860	871	807
Glyr1	4860	5267	5135	5017	5225	5247	5164	4773
Gm10015	4	2	1	3	2	4	1	1
Gm10029	8	7	8	5	13	10	8	3
Gm10033	72	89	71	79	98	95	103	88
Gm10037	3	7	0	0	0	1	1	4
Gm10044	17	10	28	13	26	27	20	16
Gm10130	92	86	57	59	70	78	62	57
Gm10145	49	38	65	36	48	50	47	40
Gm10157	29	41	24	43	25	25	34	46
Gm10193	0	1	0	0	1	1	2	0
Gm10209	3	3	13	8	23	10	9	6
Gm10220	0	1	0	0	0	0	0	0
Gm10224	2	2	0	1	2	2	0	1
Gm10277	4	0	0	0	0	1	0	0
Gm10320	25	23	8	13	26	25	16	22
Gm10334	0	1	0	0	8	10	0	7
Gm10382	4	19	13	10	28	19	3	12
Gm10420	37	35	37	34	32	49	30	49
Gm1043	11	14	24	17	21	16	13	19
Gm10461	42	30	37	40	38	43	49	57
Gm10476	0	0	2	2	1	0	0	0
Gm10479	0	0	0	0	2	0	0	2
Gm10499	2843	2905	2875	2771	2766	2725	2706	2303
Gm10509	114	84	110	134	78	138	77	79
Gm10591	0	0	1	0	0	0	0	6
Gm10634	8	6	10	8	2	8	4	8
Gm10639	22	27	32	58	43	50	48	47
Gm10654	0	1	0	0	1	0	4	0
Gm1070	1	1	5	2	9	5	2	6
Gm10710	0	0	0	0	1	0	0	1
Gm10767	47	44	49	52	65	44	47	62
Gm10778	7	2	2	2	8	4	12	13
Gm10840	3	0	0	0	0	0	0	0
Gm10913	0	0	1	0	0	4	0	1
Gm11100	32	15	17	19	11	5	7	5
Gm11127	2622	2766	2185	2103	2259	2477	2771	2523
Gm1123	9265	10713	9688	9641	9773	10391	9457	9487
Gm11437	868	1090	983	822	1321	1172	1193	1120
Gm11541	0	0	1	1	0	4	0	0
Gm11544	1	4	1	0	0	0	0	0
Gm11545	4977	5689	5337	5405	5815	5561	5516	5745
Gm11633	9	4	3	1	9	6	10	1
Gm11639	0	0	0	0	1	0	0	0
Gm11703	19	12	16	14	12	11	7	11
Gm11837	0	0	0	0	5	1	4	5
Gm11954	0	0	0	0	0	0	7	0
Gm11964	160	138	186	115	169	176	174	156
Gm11971	0	0	0	0	1	0	0	0
Gm11992	27	47	37	41	33	32	26	35

Transcriptome sequencing yielded total genetic results for the MOD and APS groups, with a total of 15,936 variables

Continued from above

Gm12185	205	192	144	156	79	82	71	100
Gm12250	3587	4501	3836	3297	2890	3044	2746	2258
Gm12253	3	0	2	5	0	0	2	3
Gm12258	89	51	72	52	53	56	73	49
Gm12407	4	0	0	5	0	0	0	0
Gm12500	12	5	19	14	25	18	15	8
Gm12502	10	6	4	5	0	1	0	1
Gm12538	1	3	0	1	0	1	2	1
Gm12643	0	0	0	0	2	0	0	0
Gm12657	27	27	42	28	40	24	35	25
Gm12693	19	28	32	27	12	27	30	20
Gm12712	0	1	2	4	1	0	1	0
Gm12722	0	1	0	0	2	1	7	4
Gm128	0	0	0	0	0	2	0	0
Gm12816	3	3	10	10	11	11	0	4
Gm12854	43	44	46	56	80	67	71	63
Gm13030	0	0	0	1	0	0	0	0
Gm13194	15	7	17	5	7	26	4	4
Gm13212	76	138	110	75	97	94	103	81
Gm13283	0	0	0	0	0	0	1	0
Gm13286	0	1	0	0	0	0	0	0
Gm13420	4	6	13	1	1	1	3	1
Gm13430	2	0	3	0	4	0	0	0
Gm13547	0	0	4	0	0	0	0	0
Gm13570	0	2	0	4	0	0	0	0
Gm13653	0	3	0	4	0	2	1	0
Gm13889	517	504	466	473	426	362	339	417
Gm14124	0	0	0	0	1	0	1	0
Gm14137	170	207	173	147	142	160	182	145
Gm14200	4	6	8	1	4	3	0	5
Gm14279	2	1	0	0	0	0	0	2
Gm14288	0	1	1	1	2	1	2	2
Gm14295	69	78	74	91	85	109	130	100
Gm14296	0	7	2	0	1	13	3	44
Gm14305	66	54	62	84	95	116	99	55
Gm14308	0	0	0	0	4	1	0	0
Gm14322	0	2	2	9	12	17	9	8
Gm14325	107	164	185	111	175	154	203	148
Gm14326	79	129	108	120	144	158	119	102
Gm14391	1	4	4	3	8	6	28	10
Gm14393	6	5	7	11	9	9	9	12
Gm14410	70	81	55	65	107	94	95	85
Gm14421	4	8	1	4	3	5	1	1
Gm14434	0	1	0	2	0	2	0	0
Gm14438	5	5	9	11	12	8	4	14
Gm14440	0	2	0	3	5	1	5	1
Gm14444	0	0	0	0	1	1	0	0
Gm14548	0	0	0	1	0	0	0	1
Gm14569	0	0	0	0	6	0	4	0
Gm14681	256	245	270	209	246	307	294	251
Gm14685	0	0	0	4	0	0	0	0
Gm15056	1	1	6	5	0	1	5	8
Gm15104	1	1	0	0	0	0	0	0
Gm15246	40	26	22	29	23	10	25	18
Gm15448	2	6	2	10	1	2	2	1
Gm15455	4	7	1	12	4	1	6	1
Gm15483	5	4	1	1	9	6	0	2
Gm15501	97	65	65	80	70	77	115	76

Transcriptome sequencing yielded total genetic results for the MOD and APS groups, with a total of 15,936 variables

Continued from above

Gm15682	4	6	2	5	10	5	7	7
Gm15793	2	15	10	7	9	5	3	4
Gm15946	2	1	1	0	0	1	0	2
Gm16440	0	0	1	0	0	0	0	0
Gm16500	0	1	0	0	0	0	0	0
Gm16527	0	0	0	0	0	1	0	0
Gm1673	7	4	12	0	0	10	4	4
Gm16867	25	58	40	6	36	26	40	31
Gm17019	0	0	0	0	0	1	0	0
Gm17149	4	1	4	0	0	0	1	2
Gm17330	10	16	48	25	29	49	37	23
Gm17415	0	1	0	1	0	0	1	0
Gm17434	0	0	0	0	1	0	0	0
Gm17455	0	0	1	0	2	0	1	0
Gm17669	3	4	4	0	2	1	4	1
Gm17748	0	2	0	0	0	0	0	0
Gm17768	0	0	0	0	0	0	0	1
Gm17778	0	0	0	4	0	0	0	0
Gm17783	149	171	139	156	149	130	128	170
Gm19345	5	0	0	4	2	0	1	0
Gm19774	0	2	4	0	0	2	10	2
Gm1988	0	0	0	4	0	0	0	0
Gm2004	6	0	1	5	1	2	3	11
Gm20056	21	13	17	23	34	19	29	16
Gm2007	1	3	0	0	0	5	0	1
Gm2026	0	1	0	2	1	5	3	1
Gm20346	194	149	201	209	199	157	181	191
Gm2036	0	1	0	0	0	0	0	1
Gm20517	4	0	0	0	1	8	0	0
Gm20760	1	0	0	0	0	0	0	0
Gm20815	0	1	0	0	0	0	0	0
Gm20841	0	0	0	0	1	0	0	0
Gm20939	175	185	150	154	164	186	174	148
Gm2102	1	8	18	2	3	2	5	7
Gm21149	1	0	0	0	0	0	1	1
Gm2115	3	1	12	7	8	6	7	14
Gm21188	2	15	10	7	1	11	2	1
Gm21190	1	1	0	0	0	0	0	0
Gm21297	65	55	37	36	89	52	57	16
Gm2137	6	1	0	1	1	1	2	1
Gm21427	0	0	0	0	0	0	1	0
Gm21451	2	4	0	8	12	2	7	4
Gm21571	0	0	0	2	0	1	1	0
Gm21814	0	4	0	2	9	8	8	0
Gm21885	494	578	564	486	698	747	641	704
Gm2237	10	2	3	11	32	9	4	4
Gm2396	0	0	0	0	0	0	0	1
Gm2423	25	43	24	13	27	29	44	32
Gm2427	0	5	2	2	3	0	5	0
Gm2436	0	0	0	1	0	0	0	1
Gm24392	0	0	0	0	1	1	0	1
Gm2446	3	1	0	18	24	0	3	0
Gm266	8	2	0	1	2	1	1	1
Gm2663	1	1	0	0	48	17	11	26
Gm26637	58	53	52	30	32	27	34	31
Gm272	0	1	0	0	0	0	0	0
Gm2808	0	18	4	5	11	29	11	5
Gm28455	10	1	1	2	2	2	7	0

Transcriptome sequencing yielded total genetic results for the MOD and APS groups, with a total of 15,936 variables

Continued from above

Gm28710	7	1	5	2	4	0	1	1
Gm28729	0	0	0	1	0	1	0	0
Gm28802	0	2	1	0	3	0	1	9
Gm2897	1	2	0	9	7	1	4	0
Gm2956	0	0	0	1	0	0	0	0
Gm29667	4	6	3	34	11	4	0	3
Gm29721	1	0	1	0	0	0	1	0
Gm29733	0	0	2	0	0	0	0	0
Gm2974	0	0	1	0	0	0	0	0
Gm29758	2	3	3	0	3	1	2	1
Gm29770	2	0	4	6	1	0	9	2
Gm29776	0	0	1	0	1	1	0	0
Gm29797	3	1	5	4	1	1	1	2
Gm29808	0	0	0	0	0	0	1	0
Gm29825	0	0	0	0	0	0	0	1
Gm2a	918	992	958	1076	1019	1053	932	970
Gm30191	0	0	4	1	1	1	0	0
Gm30447	0	1	2	1	0	2	0	6
Gm30502	5	5	2	0	5	2	7	5
Gm3055	5	0	1	11	8	13	3	0
Gm30599	8	10	11	9	8	3	10	5
Gm30679	3	5	11	5	5	13	5	6
Gm30698	2	0	8	7	2	7	0	4
Gm30732	4	2	11	8	4	1	10	0
Gm30733	0	0	1	0	0	0	0	0
Gm3095	0	0	1	0	0	0	0	0
Gm30990	49	69	103	72	92	33	56	78
Gm31160	57	48	62	78	55	48	51	63
Gm31255	3	16	0	5	6	5	1	8
Gm31332	1	2	0	2	7	1	1	2
Gm31493	0	5	1	0	1	0	0	6
Gm31513	36	10	19	6	16	8	16	24
Gm31526	0	0	0	0	1	0	0	0
Gm31649	12	24	8	6	4	10	0	14
Gm3173	2	0	0	1	0	0	0	1
Gm3194	0	0	0	0	0	0	1	0
Gm32234	2	0	0	3	6	2	11	0
Gm3248	1	1	0	0	0	0	0	0
Gm32584	38	24	24	40	18	30	32	50
Gm3264	2	1	0	0	0	6	3	0
Gm32687	13	25	32	32	21	13	17	16
Gm32717	2	1	0	3	6	3	3	4
Gm32719	82	66	70	117	67	73	71	52
Gm32742	20	14	9	21	15	12	14	7
Gm32802	18	23	12	20	1	12	12	2
Gm32856	54	56	32	41	40	62	57	56
Gm32886	0	0	0	1	1	0	0	0
Gm33049	0	4	4	5	1	0	0	1
Gm33153	188	203	177	208	112	145	107	151
Gm3325	83	77	47	79	75	91	95	90
Gm3336	927	961	928	929	1085	974	910	1166
Gm33666	0	0	0	3	1	2	0	0
Gm3373	0	1	0	0	0	0	0	0
Gm3383	1	1	0	2	0	0	3	0
Gm33851	7	16	14	15	14	16	4	5
Gm33869	0	1	0	1	1	4	0	1
Gm33887	41	10	23	11	32	10	30	18
Gm33933	0	2	0	1	0	0	0	0

Transcriptome sequencing yielded total genetic results for the MOD and APS groups, with a total of 15,936 variables

Continued from above

Gm33989	327	380	374	425	395	438	407	360
Gm34066	1	0	0	0	0	0	0	0
Gm3411	14	15	15	35	27	8	25	12
Gm3417	0	0	0	0	0	1	0	0
Gm34296	1	0	0	0	0	0	1	1
Gm3435	35	8	35	2	13	7	24	40
Gm34362	0	0	0	0	1	0	6	0
Gm34531	1	1	0	0	0	1	1	3
Gm34595	0	0	0	0	1	1	0	0
Gm34653	0	0	1	0	0	4	0	0
Gm3470	1	1	0	0	1	0	1	1
Gm34962	237	190	227	258	218	208	214	193
Gm35060	0	0	0	0	0	0	1	0
Gm35078	4	3	6	3	18	1	0	5
Gm35083	0	0	4	0	0	0	0	0
Gm3512	0	1	0	0	0	0	0	0
Gm35315	18	29	18	16	13	18	6	27
Gm35339	52	53	64	60	80	73	48	65
Gm35364	0	9	4	6	1	10	0	0
Gm35549	0	1	0	0	0	0	0	0
Gm3558	1	4	1	0	1	0	0	0
Gm35857	3	4	3	1	3	2	4	4
Gm35953	0	0	0	0	0	0	0	1
Gm36028	6	1	0	0	0	8	0	0
Gm3604	25	38	20	23	31	20	26	23
Gm36118	1	1	1	0	0	2	4	1
Gm36182	0	0	0	0	3	0	1	0
Gm36298	13	8	14	4	14	8	8	12
Gm3636	21	18	11	6	14	16	14	13
Gm36375	0	1	0	2	0	0	0	0
Gm3646	0	0	0	0	0	0	1	0
Gm3650	2	0	2	0	2	0	2	0
Gm3667	0	0	1	0	0	0	0	4
Gm36712	0	4	1	0	0	0	0	0
Gm36722	6	3	5	2	2	5	4	2
Gm36789	4	0	0	0	0	0	0	1
Gm36864	2	2	11	2	4	2	7	6
Gm3696	0	0	0	2	1	0	0	1
Gm3739	0	1	0	0	1	0	0	0
Gm37500	3	0	0	0	0	0	0	0
Gm3776	1130	1046	775	704	1465	1686	1156	1474
Gm3785	0	0	1	0	0	0	0	0
Gm38396	155	193	187	155	217	198	169	172
Gm38469	1	4	2	3	0	0	3	0
Gm38499	0	0	1	6	1	2	3	4
Gm38510	1	8	5	4	1	4	2	0
Gm38525	1	2	0	2	0	5	5	0
Gm38538	1	0	0	0	0	1	0	0
Gm38574	0	0	2	1	0	1	2	1
Gm38664	52	28	26	29	37	33	22	41
Gm38699	0	1	0	0	0	1	2	1
Gm38702	0	0	1	1	0	0	1	1
Gm39469	180	201	166	149	180	201	208	184
Gm39572	0	2	3	2	2	0	1	1
Gm39701	5	5	7	3	6	2	1	4
Gm39743	3	2	0	1	2	3	0	1
Gm40011	0	1	0	0	0	0	2	1
Gm40095	7	11	13	17	17	11	23	12

Transcriptome sequencing yielded total genetic results for the MOD and APS groups, with a total of 15,936 variables

Continued from above

Gm40190	0	0	5	0	1	0	1	0
Gm40275	0	0	1	1	0	0	0	1
Gm40353	1	0	0	0	0	0	0	1
Gm40363	0	0	1	0	0	2	0	1
Gm40364	0	0	0	0	2	0	0	2
Gm40367	0	0	1	0	0	0	0	0
Gm40369	0	0	0	0	1	1	1	1
Gm40378	7	5	1	1	2	4	0	5
Gm40447	4	4	0	1	0	1	1	0
Gm40453	31	25	56	46	9	2	11	5
Gm40469	1	1	1	6	6	2	0	1
Gm40595	0	7	2	1	3	0	2	2
Gm40811	4	6	3	2	1	2	5	1
Gm40814	0	1	2	6	0	0	0	3
Gm40853	1	0	0	0	1	0	0	0
Gm40892	61	87	100	72	103	137	73	41
Gm4120	0	1	1	0	0	0	0	0
Gm41291	79	80	33	69	50	58	79	56
Gm4131	0	0	1	0	0	0	0	0
Gm41408	10	32	12	30	4	31	13	30
Gm41607	2	0	0	0	0	0	0	2
Gm41844	45	54	40	45	10	12	25	41
Gm4199	8	6	9	10	4	18	7	2
Gm42226	2059	2209	2089	2062	2420	2292	1927	2268
Gm42323	17	19	28	21	32	20	38	8
Gm42337	4	4	0	2	6	0	0	5
Gm42346	0	0	0	0	0	1	0	0
Gm42372	8	3	6	1	8	6	5	7
Gm42427	0	1	2	3	0	6	1	2
Gm42517	6	1	7	7	1	2	5	13
Gm4275	0	1	0	1	0	0	1	0
Gm43247	1	3	0	1	2	4	4	2
Gm44504	2	0	0	1	3	5	2	0
Gm45095	25	29	37	34	35	12	23	26
Gm45521	4	0	0	1	0	1	0	1
Gm45623	2	0	1	0	0	1	0	1
Gm45855	88	104	93	115	148	119	106	126
Gm45871	429	528	623	437	570	592	586	525
Gm45915	0	1	0	1	0	0	0	0
Gm45927	0	0	1	0	0	0	0	0
Gm45975	0	0	0	1	0	0	0	0
Gm45978	4	6	0	2	6	2	5	5
Gm45988	4	8	0	4	0	0	0	4
Gm46058	1	0	0	5	1	1	4	6
Gm4606	1	5	0	0	0	0	0	0
Gm46139	13	26	13	18	17	31	13	15
Gm46142	0	0	0	0	1	0	0	0
Gm46290	6	6	7	2	2	4	2	0
Gm46294	4	4	0	5	12	4	2	2
Gm46305	85	112	163	96	114	128	134	147
Gm4631	0	0	0	4	0	0	0	2
Gm46319	36	28	26	29	41	31	21	34
Gm46345	1	0	1	0	1	0	0	1
Gm46353	8	23	7	11	10	10	3	13
Gm46382	16	28	21	17	28	30	18	11
Gm46415	0	0	0	0	0	1	0	0
Gm46430	74	208	169	119	150	124	128	100
Gm46442	1	1	0	1	3	3	2	0

Transcriptome sequencing yielded total genetic results for the MOD and APS groups, with a total of 15,936 variables

Continued from above

Gm46546	30	35	25	36	21	26	27	31
Gm46608	0	0	0	0	0	0	1	1
Gm46617	42	38	27	59	40	19	30	47
Gm46629	0	4	0	8	0	1	0	0
Gm46731	6	11	8	4	15	2	1	7
Gm46900	0	1	2	0	1	7	1	0
Gm46911	8	9	7	9	2	2	8	6
Gm46915	0	0	0	1	0	0	0	0
Gm46933	64	34	38	30	47	67	46	24
Gm46965	17	9	8	23	23	8	17	20
Gm4724	5	11	4	3	3	3	8	13
Gm4737	904	958	1007	967	1146	1134	946	952
Gm47655	0	0	2	0	1	0	2	1
Gm4767	4	0	0	0	0	0	0	0
Gm4779	6	2	0	2	5	1	12	0
Gm4787	22	29	20	20	21	32	25	24
Gm4788	7	3	3	2	0	6	4	2
Gm4796	1	0	0	0	0	0	0	0
Gm4832	1	0	0	0	0	0	0	0
Gm48350	21	32	16	2	9	15	19	18
Gm4841	31	33	22	17	9	19	26	9
Gm48552	26	26	34	36	42	67	39	46
Gm4861	0	0	0	1	0	0	0	0
Gm48826	0	1	0	0	0	0	0	0
Gm4924	21	18	16	41	24	21	29	29
Gm4925	5	11	16	13	18	11	27	7
Gm4951	163	163	132	147	98	143	108	81
Gm4952	1	1	1	1	1	1	5	2
Gm4963	0	1	2	4	5	2	1	1
Gm4972	9	17	21	3	14	11	6	6
Gm4984	0	0	0	0	0	1	0	0
Gm4995	0	0	0	0	1	0	0	1
Gm50595	0	0	1	0	0	1	0	2
Gm50598	0	0	0	1	0	0	0	0
Gm5108	1	1	0	0	0	0	1	0
Gm5127	0	0	1	0	1	6	1	0
Gm5134	9	4	13	4	7	6	2	14
Gm5141	27	10	8	12	14	5	7	18
Gm51425	46	113	78	92	84	82	110	85
Gm51460	17	10	6	13	4	0	13	15
Gm51464	0	0	0	0	0	4	0	0
Gm5148	8	4	10	2	6	4	6	5
Gm5150	31	46	38	33	57	46	34	62
Gm51579	19	10	6	17	8	1	4	6
Gm51598	1	0	0	0	0	0	0	0
Gm5165	80	122	114	109	94	98	126	102
Gm51877	21	12	13	22	40	25	21	22
Gm51963	0	0	0	1	0	0	0	0
Gm51965	1	0	0	0	0	4	0	0
Gm51999	0	4	0	7	3	2	0	0
Gm52009	7	16	13	3	19	20	4	13
Gm52051	2	2	2	4	3	0	1	1
Gm52140	0	0	2	7	2	0	1	2
Gm5218	0	0	0	0	0	0	1	0
Gm52217	1	3	1	3	2	6	1	2
Gm52229	0	1	0	0	0	0	0	0
Gm52241	3	5	7	12	0	2	0	1
Gm52255	1	0	0	0	0	0	2	0

Transcriptome sequencing yielded total genetic results for the MOD and APS groups, with a total of 15,936 variables

Continued from above

Gm52310	4	3	0	8	9	9	6	8
Gm52351	34	36	42	38	43	48	56	55
Gm52481	1	0	4	0	1	1	1	0
Gm525	3	0	0	0	0	0	0	0
Gm52512	5	2	6	0	0	0	1	0
Gm52523	1	6	0	0	0	0	0	1
Gm52666	4	6	0	6	0	1	2	0
Gm527	13	22	13	17	14	17	18	10
Gm52720	0	1	1	0	6	4	0	0
Gm52800	1	0	1	0	0	0	0	1
Gm52806	0	1	0	0	0	0	0	0
Gm52875	0	0	0	1	0	0	0	0
Gm536	1	0	0	0	0	0	0	0
Gm5431	2572	2889	2588	2172	1838	2042	1886	1658
Gm5451	33	45	53	40	57	43	31	39
Gm5454	12	11	10	9	8	21	11	9
Gm5459	6	0	0	0	0	1	0	0
Gm550	0	0	1	1	3	1	2	1
Gm5528	0	0	0	1	0	0	0	0
Gm5544	0	1	2	0	0	0	0	0
Gm5553	1	1	1	0	0	1	0	1
Gm5576	0	0	0	0	0	1	0	0
Gm5617	224	150	170	140	123	157	154	117
Gm5637	202	188	159	167	216	207	214	168
Gm5640	4	0	0	2	0	0	0	0
Gm5662	0	0	0	0	0	0	1	0
Gm572	12	9	5	3	9	8	12	8
Gm5737	0	0	0	0	6	0	1	0
Gm5741	2	1	0	0	8	4	0	1
Gm5767	0	0	5	4	1	0	0	8
Gm5771	16	18	3	0	60	58	35	32
Gm5784	17	11	20	23	39	13	16	4
Gm5785	117	171	158	146	125	146	104	111
Gm5796	0	1	0	0	1	1	0	2
Gm5820	1	1	1	0	3	0	0	6
Gm5848	0	1	0	2	0	0	0	0
Gm5901	4	3	1	7	2	11	4	4
Gm5928	1	1	1	0	3	0	1	2
Gm5977	4	3	13	11	13	15	9	8
Gm6034	102	155	87	95	80	134	99	105
Gm609	194	215	232	211	225	186	223	199
Gm6109	1	0	0	0	0	0	0	0
Gm6139	17	7	26	11	15	17	18	29
Gm614	0	4	0	1	4	1	0	2
Gm6155	0	1	0	0	6	1	10	1
Gm6158	7	2	3	6	2	7	6	14
Gm6195	5	6	4	5	1	9	15	2
Gm6285	0	0	1	0	3	0	0	0
Gm6293	0	1	3	2	0	1	4	0
Gm6314	0	0	1	0	0	0	0	0
Gm6377	35	59	34	29	23	42	12	38
Gm6569	0	1	0	0	0	0	0	1
Gm6570	0	0	0	0	0	1	0	0
Gm6583	4	0	0	0	0	0	0	0
Gm6625	4	1	5	10	0	2	3	3
Gm6710	4	2	6	42	33	32	2	0
Gm6712	125	131	140	128	166	151	165	103
Gm6729	0	1	0	0	0	0	0	0

Transcriptome sequencing yielded total genetic results for the MOD and APS groups, with a total of 15,936 variables

Continued from above

Gm6749	0	2	3	11	2	2	4	1
Gm6750	14	14	16	9	16	8	6	9
Gm6829	6	8	1	0	10	5	6	12
Gm6871	0	0	0	1	0	0	0	0
Gm6988	26	23	19	15	23	14	22	25
Gm7030	214	167	169	193	135	181	176	147
Gm7072	456	484	423	419	588	475	558	504
Gm7094	0	0	1	0	3	0	0	1
Gm7102	0	1	2	0	0	0	0	0
Gm715	0	0	1	0	1	1	0	0
Gm7168	0	0	1	1	0	0	0	0
Gm7206	25	10	27	39	20	34	33	40
Gm7429	2	1	9	3	2	7	1	1
Gm7579	0	0	1	0	0	0	0	0
Gm7592	12	2	8	7	7	2	2	2
Gm7609	5	19	20	18	8	11	19	9
Gm765	0	0	0	0	0	0	2	1
Gm7694	212	233	228	204	168	238	211	198
Gm7697	1	1	0	0	0	0	0	1
Gm7932	98	95	87	97	89	111	102	88
Gm7972	3	5	10	7	3	8	2	0
Gm8108	0	0	0	0	2	0	0	0
Gm8251	8	6	7	3	4	11	7	1
Gm826	38	33	30	38	42	22	40	17
Gm8290	10	1	1	5	2	1	0	5
Gm8369	2	0	4	0	1	1	0	0
Gm8396	0	2	0	0	0	4	1	0
Gm8587	4	2	1	0	3	0	7	1
Gm8618	0	0	1	0	0	0	0	0
Gm867	0	0	5	3	2	1	2	2
Gm8773	0	1	5	0	0	0	1	0
Gm884	0	0	0	0	1	0	0	0
Gm8909	9453	10368	8748	8038	9551	10296	10440	8615
Gm8947	0	1	0	0	5	4	0	1
Gm8978	0	0	0	0	0	0	0	1
Gm9040	0	1	0	1	0	0	0	0
Gm9045	0	0	0	0	1	0	0	0
Gm9048	0	0	1	0	0	0	0	0
Gm9049	0	1	0	0	0	0	0	0
Gm9182	12	3	12	18	3	3	12	6
Gm9195	0	1	1	0	2	4	0	0
Gm9222	225	230	258	235	313	251	262	275
Gm9234	37	44	39	31	59	37	45	29
Gm9237	5	12	13	8	5	5	4	0
Gm9257	0	0	0	0	2	0	1	1
Gm9595	0	0	1	0	0	0	0	0
Gm960	2	0	1	2	0	0	0	0
Gm9640	0	2	1	0	0	6	1	0
Gm973	9	0	6	4	1	2	5	0
Gm9733	0	0	0	0	0	1	0	0
Gm9805	1	0	0	0	0	1	0	0
Gm9949	6	7	3	7	3	5	2	3
Gm9997	0	2	0	0	0	0	1	2
Gmcl1	1464	1451	1399	1524	1443	1513	1387	1403
Gmds	19612	21861	21410	19889	21254	22487	21940	22582
Gmeb1	615	690	707	662	718	715	717	640
Gmeb2	1032	1204	1032	1043	981	1137	1097	1006
Gmfb	3266	3510	3431	3232	3605	3782	3561	3196

Transcriptome sequencing yielded total genetic results for the MOD and APS groups, with a total of 15,936 variables

Continued from above

Gmfg	501	489	534	476	518	503	470	509
Gmfg-ps	0	3	0	0	1	4	1	8
Gmip	1010	1240	1081	1099	1168	1198	1216	1105
Gml	224	303	247	150	346	372	490	559
Gml2	126	158	117	69	195	178	278	319
Gmnn	969	940	913	1003	952	898	971	853
Gmppa	2982	3257	3003	2873	3286	3146	3245	3183
Gmppb	4715	5364	4505	4219	4781	4778	4598	4565
Gmpr	45	65	44	41	47	42	25	31
Gmpr2	702	682	798	663	687	735	777	681
Gmps	1268	1529	1411	1399	1440	1405	1378	1365
Gna11	41161	40687	41177	42470	36197	38469	37672	35811
Gna12	537	528	506	570	630	538	471	534
Gna13	5891	6430	6321	6362	6241	6679	6492	6031
Gna14	28	14	23	25	23	30	31	35
Gna15	27	11	5	13	19	6	9	6
Gnai1	149	136	133	172	159	113	134	121
Gnai2	10621	11136	10417	10309	10759	10865	10321	10310
Gnai3	9873	10524	9715	9794	11756	11936	11884	11222
Gnal	147	128	137	114	91	91	108	87
Gnao1	158	225	209	207	204	192	202	162
Gnaq	1917	1871	1694	1675	1972	1851	1662	1616
Gnas	15664	16057	16591	15988	15062	16074	14693	14861
Gnat1	2	7	2	3	0	3	0	5
Gnat2	3	27	12	8	26	4	28	26
Gnat3	0	0	1	0	0	0	0	0
Gnaz	19	21	17	14	11	42	2	12
Gnb1	26739	27607	26726	27697	24330	25430	25647	24936
Gnb1l	102	92	107	103	93	78	74	93
Gnb2	11709	11938	11603	11470	10665	10863	10202	10455
Gnb3	1	5	2	0	0	5	0	1
Gnb4	271	292	252	265	210	216	162	211
Gnb5	462	292	333	455	267	292	359	416
Gne	1872	1990	2036	2169	1879	1800	1617	1703
Gng10	393	379	337	329	409	397	347	351
Gng11	131	194	153	133	153	175	125	166
Gng12	15106	16842	15232	15207	15306	16464	16951	15109
Gng13	6	2	4	2	0	1	1	1
Gng2	188	269	284	238	267	198	202	213
Gng3	12	22	19	14	26	10	13	12
Gng4	96	109	104	77	98	113	72	88
Gng5	4636	4937	4820	4722	4561	4849	4752	4486
Gng7	18	11	16	30	12	16	14	13
Gng8	3	6	1	2	8	12	1	1
Gngt1	0	1	0	0	1	7	0	4
Gngt2	121	89	92	112	93	119	145	89
Gnl1	2738	2754	2668	2585	2427	2400	2346	2303
Gnl2	901	866	708	801	811	779	742	863
Gnl3	598	860	740	599	813	801	764	791
Gnl3l	995	1197	1058	1087	1125	1172	1204	1062
Gnmt	20	18	12	19	19	39	17	18
Gnpat	1549	1590	1557	1577	1672	1637	1519	1577
Gnpda1	4047	4292	4025	4005	4118	4032	4191	3664
Gnpda2	358	390	366	397	339	381	331	313
Gnpnat1	1392	1464	1338	1577	1498	1353	1481	1427
Gnptab	1412	1571	1358	1519	1384	1341	1298	1307
Gnptg	987	886	944	773	817	829	905	892
Gnrh1	14	20	18	22	22	32	15	21

Transcriptome sequencing yielded total genetic results for the MOD and APS groups, with a total of 15,936 variables

Continued from above

Gnrhr	0	0	0	0	0	0	4	0
Gns	7045	7151	6512	6438	6439	6255	6809	6751
Golga1	2208	2225	2209	2098	2604	2482	2466	2171
Golga2	3371	3629	3429	3751	3676	3922	4023	3700
Golga3	1399	1509	1385	1433	1617	1431	1556	1438
Golga4	7225	7923	7263	7179	7924	8600	8681	7399
Golga5	1760	2104	1795	1881	1942	2113	2109	1938
Golga7	1803	2138	1878	1875	2166	1997	1854	2072
Golga7b	3	22	6	8	11	10	13	18
Golgb1	3525	3803	3729	3940	3863	3947	4033	3499
Golim4	1526	1549	1397	1484	1584	1707	1540	1534
Golm1	9764	11460	10676	9679	12368	11611	11061	11135
Golm2	196	307	329	298	289	296	274	256
Golph3	7853	8327	8164	8783	7358	7369	7458	7029
Golph3l	3405	3879	3467	3529	4141	3959	3658	3422
Golt1a	3071	3137	3229	2962	2945	2869	2911	2859
Golt1b	655	657	618	566	766	732	649	727
Gon4l	935	1107	996	1021	1023	1046	860	975
Gon7	136	181	209	170	245	265	227	175
Gopc	888	856	782	852	1154	940	865	842
Gorab	403	515	460	434	435	452	409	453
Gorasp1	890	973	1077	953	1008	1064	979	1048
Gorasp2	6047	6242	5870	5928	6485	6632	6194	6082
Gosr1	3169	3247	3105	2993	3211	3262	3048	3003
Gosr2	6345	7121	5895	5783	6889	6908	6784	6219
Got1	9300	8965	8926	10184	7480	8086	8344	8338
Got1l1	0	1	0	0	0	2	0	1
Got2	4486	4475	4677	4553	4654	4478	3881	4086
Gp1ba	0	1	2	1	5	0	1	0
Gp1bb	963	974	940	822	643	722	735	822
Gp2	454	621	309	141	2682	2305	1772	2309
Gp5	5	0	0	2	0	0	0	5
Gp6	2	8	2	2	6	1	0	2
Gp9	6	1	4	8	6	4	2	1
Gpa33	24548	25682	24022	23501	23886	23513	23092	22366
Gpaa1	1928	1893	1914	1704	1981	1895	1732	1827
Gpalpp1	1172	1321	1181	1257	1203	1420	1282	1179
Gpam	552	601	487	469	531	582	502	443
Gpank1	627	690	628	600	641	617	587	615
Gpat2	0	0	1	0	1	0	0	0
Gpat3	6346	7047	5991	5780	6269	6746	7075	6437
Gpat4	1549	1559	1673	1600	1857	1711	1412	1391
Gpatch1	458	601	535	456	521	490	553	574
Gpatch11	509	544	483	557	555	616	469	479
Gpatch2	1205	1471	1429	1201	1373	1304	1426	1198
Gpatch2l	788	845	770	800	800	803	806	758
Gpatch3	354	425	351	352	399	354	357	281
Gpatch4	330	380	346	265	337	391	339	291
Gpatch8	2614	3015	2837	2699	2928	3093	2887	2664
Gpbar1	9	5	3	2	5	4	3	2
Gpbp1	2518	2877	2550	2734	2999	3314	3371	2811
Gpbp1l1	4076	4481	4193	4288	4495	4561	4170	4310
Gpc1	135	166	173	139	148	219	134	155
Gpc2	13	16	6	6	11	29	3	8
Gpc3	90	69	88	76	93	130	87	82
Gpc4	1671	1941	1737	1688	1805	1729	1783	1828
Gpc5	0	0	0	0	0	0	1	0
Gpc6	105	105	89	90	85	83	115	85

Transcriptome sequencing yielded total genetic results for the MOD and APS groups, with a total of 15,936 variables

Continued from above

Gpcpd1	2752	3137	2611	2457	2796	3203	3510	3424
Gpd1	26974	30165	27733	25445	31435	33050	33247	30547
Gpd1l	5738	6199	6276	6091	6247	6108	5969	5718
Gpd2	14163	13391	12760	14757	12058	11487	12980	12551
Gper1	16	15	14	16	33	45	17	32
Gpha2	6	0	0	0	0	0	0	1
Gphb5	0	0	0	0	1	0	0	0
Gphn	1030	1030	1115	1009	973	960	823	750
Gpi1	28187	27028	27764	28691	26564	27373	26644	26902
Gpihbp1	46	40	27	21	55	16	23	15
Gpkow	1342	1328	1220	1354	1180	1282	1315	1329
Gpld1	473	552	592	554	537	468	398	468
Gpm6a	44	41	37	14	57	60	50	29
Gpm6b	68	88	96	91	87	63	60	50
Gpn1	325	362	375	297	406	410	337	315
Gpn2	711	692	630	625	619	646	588	592
Gpn3	528	461	515	526	547	587	449	536
Gpnmb	121	132	78	123	124	129	108	137
Gpr1	0	0	0	1	0	0	1	0
Gpr107	4797	5582	5196	4619	5403	5589	5576	5085
Gpr108	1138	1229	1155	1082	1306	1380	1106	1212
Gpr119	4	3	6	3	12	8	1	1
Gpr12	0	0	0	0	0	0	4	0
Gpr132	37	23	39	61	61	50	21	19
Gpr137	1058	992	899	861	970	1022	1047	1046
Gpr137b	38	45	53	43	36	56	33	37
Gpr137c	7	13	16	13	18	10	14	3
Gpr141	21	14	22	16	18	22	17	13
Gpr141b	1	0	5	4	1	2	1	0
Gpr142	5	0	0	6	1	2	0	1
Gpr143	0	0	0	0	0	1	0	0
Gpr146	152	132	192	122	103	144	122	108
Gpr149	9	10	10	15	6	17	4	9
Gpr15	1	1	2	21	1	4	2	1
Gpr150	0	0	0	0	0	1	0	0
Gpr151	239	282	270	256	217	247	284	322
Gpr152	4	4	0	0	0	0	5	0
Gpr153	151	167	191	139	178	116	180	194
Gpr155	1578	1602	1485	1225	1641	1651	1436	1208
Gpr156	1	2	0	0	1	4	1	4
Gpr157	442	451	472	501	368	358	363	389
Gpr158	1	1	0	1	1	2	0	0
Gpr160	2601	2695	2315	2421	2101	2242	2353	2299
Gpr161	36	55	73	65	57	28	65	36
Gpr162	20	17	19	13	13	19	23	19
Gpr165	0	0	1	0	0	0	0	0
Gpr17	130	147	94	59	173	158	122	111
Gpr171	54	72	87	77	36	40	68	55
Gpr173	2	2	13	5	9	6	5	4
Gpr174	1	5	5	0	9	2	5	14
Gpr176	13	11	12	10	13	21	11	8
Gpr179	14	10	8	10	1	2	3	6
Gpr18	29	27	19	53	36	36	14	27
Gpr180	496	486	563	486	486	557	442	492
Gpr182	43	40	40	30	35	36	36	28
Gpr183	24	19	18	28	24	23	27	21
Gpr19	94	122	126	125	84	71	75	68
Gpr20	96	94	134	114	93	92	125	133

Transcriptome sequencing yielded total genetic results for the MOD and APS groups, with a total of 15,936 variables

Continued from above

Gpr21	0	2	1	0	1	0	7	0
Gpr22	123	101	105	157	106	157	169	110
Gpr25	1	0	1	0	0	0	0	0
Gpr27	46	41	20	39	29	29	13	35
Gpr31b	35	16	17	22	3	20	11	6
Gpr33	0	0	1	0	1	0	0	0
Gpr34	26	35	11	17	27	29	30	20
Gpr35	948	997	1006	986	929	994	886	879
Gpr37	0	2	6	0	1	0	4	0
Gpr37l1	19	14	14	13	16	23	26	28
Gpr39	1162	1336	1238	1037	1138	1238	1053	1112
Gpr4	31	48	62	49	44	25	39	63
Gpr45	4	0	0	0	0	0	0	0
Gpr50	0	0	0	1	0	0	0	0
Gpr52	26	16	12	14	11	35	25	15
Gpr55	714	743	832	849	755	819	659	608
Gpr6	3	6	11	5	0	2	0	5
Gpr62	0	0	0	0	1	0	0	0
Gpr63	0	1	0	4	0	0	0	1
Gpr65	81	48	91	102	74	92	82	73
Gpr68	25	41	45	44	47	28	22	23
Gpr75	0	0	7	5	5	0	4	2
Gpr82	5	1	2	1	0	1	0	2
Gpr83	1	0	0	0	0	0	0	0
Gpr84	1	7	2	0	1	5	1	2
Gpr85	9	21	20	31	15	18	11	18
Gpr88	2	3	2	5	10	1	1	2
Gpr89	3304	3619	3501	3368	3647	3897	3589	3305
Gprasp1	221	182	213	167	195	196	149	201
Gprasp2	7	13	2	25	16	7	12	10
Gprc5a	1794	1720	1597	1691	2169	2239	2709	2353
Gprc5b	94	121	129	113	85	118	84	85
Gprc5c	26	63	64	69	37	45	33	36
Gprc6a	0	0	1	0	0	0	0	0
Gprin1	78	49	74	52	36	66	56	44
Gprin2	1	2	2	0	1	1	2	5
Gprin3	1366	1599	1542	1526	1511	1890	2074	1538
Gps1	1634	1801	1608	1510	1859	1865	1689	1618
Gps2	1065	1045	1108	1038	1004	952	984	932
Gpsm1	88	114	104	110	104	100	92	114
Gpsm2	3549	3826	3761	3350	3326	3712	3480	3525
Gpsm3	112	144	128	104	114	136	101	76
Gpt	12367	12804	11694	10724	12103	12920	13259	13215
Gpt2	221	216	198	317	220	194	197	229
Gpx1	22330	21023	23171	23267	22384	22667	19075	20353
Gpx2	44580	35527	40013	50790	31895	31621	31694	33178
Gpx3	2489	2313	2533	2476	2456	2380	2136	2445
Gpx4	13675	13904	13291	12813	13618	14467	14059	13647
Gpx7	69	55	79	78	58	80	79	77
Gpx8	71	97	119	94	149	145	91	103
Gramd1a	259	262	283	270	319	266	257	245
Gramd1b	2350	2714	2077	2027	2561	2732	3535	3094
Gramd1c	1440	1722	1300	1251	2534	2689	2601	2346
Gramd2	1187	1336	1329	1255	1214	1393	1376	1478
Gramd3	6857	7907	6696	6497	7138	7408	7315	6636
Gramd4	1855	1841	1782	1874	1752	1742	1778	1703
Grap	94	76	102	75	84	77	81	97
Grap2	70	75	79	62	47	77	62	79

Transcriptome sequencing yielded total genetic results for the MOD and APS groups, with a total of 15,936 variables

Continued from above

Grasp	67	71	67	42	60	57	50	58
Grb10	158	192	196	159	167	182	187	138
Grb14	37	72	42	39	38	59	61	49
Grb2	3467	3689	3531	3331	3408	3451	3522	3248
Grb7	3429	3569	3430	3025	3600	3573	3502	3296
Grcc10	1234	1347	1254	1255	1244	1190	1124	1292
Greb1	136	138	134	98	204	183	169	175
Greb1l	6	5	2	8	3	2	2	0
Grem1	238	237	272	301	231	295	374	273
Grem2	75	103	86	90	118	97	123	121
Grhl1	21	44	41	30	42	37	31	39
Grhl2	545	512	464	592	493	609	487	556
Grhl3	2	9	11	22	9	14	2	3
Grhpr	993	1008	952	935	1227	1108	954	938
Gria1	5	7	6	10	8	4	1	0
Gria2	1	2	2	4	2	26	0	6
Gria3	37	10	32	28	21	28	59	28
Gria4	13	10	18	14	16	13	15	24
Grid1	0	4	0	10	5	0	0	17
Grid2	0	0	1	2	0	1	3	0
Grid2ip	0	0	4	0	0	1	0	0
Grik1	0	1	1	0	5	0	0	1
Grik2	0	2	2	2	1	0	2	0
Grik3	12	15	13	19	16	32	3	20
Grik4	7	2	7	1	4	1	7	15
Grik5	69	90	89	128	52	91	92	76
Grin1	13	27	12	20	23	18	12	10
Grin2a	21	15	13	17	5	22	18	10
Grin2c	0	0	2	4	1	6	1	0
Grin2d	290	341	293	317	321	258	318	267
Grin3a	10	6	7	3	5	1	9	12
Grin3b	3	7	7	4	5	2	2	8
Grina	7870	8178	7134	6503	7110	7455	7322	6936
Grip1	0	1	0	10	6	6	0	12
Grip2	15	8	12	4	11	18	19	6
Gripap1	1114	1220	1241	1202	1195	1323	1171	1149
Grk1	0	0	0	1	0	0	1	0
Grk2	3700	3598	3680	3536	3677	3641	3341	3329
Grk3	237	295	231	214	236	253	218	177
Grk4	7	17	5	7	14	29	11	5
Grk5	575	535	458	519	524	573	548	516
Grk6	2410	2299	2433	2405	2340	2094	1949	2125
Grm1	0	2	0	0	0	0	1	0
Grm2	4	0	1	0	4	1	0	1
Grm4	35	31	48	27	31	38	34	24
Grm7	4	9	1	1	1	0	0	0
Grm8	1	5	4	7	5	1	2	7
Grn	4144	4105	4133	4206	3697	3458	3405	3436
Grp	0	0	2	5	0	3	1	0
Grpel1	2716	2820	2701	2779	2960	2945	2639	2608
Grpel2	1204	1184	1166	1103	1340	1263	1332	1339
Grpr	3	1	6	7	9	2	3	8
Grrp1	43	47	45	49	42	59	30	47
Grsf1	2071	2234	2321	2242	2295	2332	2155	2106
Grtp1	1995	2091	2000	1931	2031	2290	1986	1870
Grwd1	529	457	522	433	578	549	464	420
Gsap	90	26	76	70	39	50	59	48
Gsdma	21	27	20	33	27	14	8	17

Transcriptome sequencing yielded total genetic results for the MOD and APS groups, with a total of 15,936 variables

Continued from above

Gsdma2	17	16	13	12	7	6	9	15
Gsdmc	1	0	0	0	0	0	1	0
Gsdmc2	1436	550	1056	1943	192	194	221	154
Gsdmc3	396	141	181	439	11	23	12	32
Gsdmc4	6123	2665	5617	9185	625	754	808	647
Gsdmd	16201	17279	14511	13844	14591	15662	16045	14887
Gsdme	28	39	41	34	59	90	77	74
Gse1	2447	2789	2810	2772	2759	2917	2651	2646
Gsg1	12	1	8	7	7	5	10	15
Gsg1l	3	4	1	1	3	1	1	4
Gsg1l2	3	1	0	0	0	0	1	0
Gsk3a	7515	7481	7338	7203	6975	7284	6883	7186
Gsk3b	3071	3523	3095	3325	3127	3064	3296	3127
Gskip	1911	2000	1913	2001	2083	2188	2231	2121
Gsn	6223	5815	5863	6681	5560	5486	5350	5439
Gspt1	5400	5995	5261	5323	5585	5685	5417	5296
Gspt2	16	21	16	9	22	19	11	18
Gsr	19979	18486	19659	22538	17071	16792	15561	16434
Gss	5096	5479	5228	5649	5235	5231	4955	5173
Gsta1	2734	3092	2860	2421	4722	4403	3806	4218
Gsta2	298	333	253	248	439	522	339	391
Gsta3	283	360	351	309	610	425	433	475
Gsta4	2164	2728	2219	1690	3955	3745	2923	3233
Gstcd	299	429	312	400	413	353	357	353
Gstk1	1224	1229	1094	981	1433	1215	1304	1449
Gstm1	2310	2507	2131	2157	4353	4834	4279	4824
Gstm2	598	533	702	495	797	711	750	772
Gstm3	3841	4753	3749	3341	7650	7625	6635	7625
Gstm4	963	1004	837	910	1276	1195	1232	1280
Gstm5	683	696	750	767	861	769	725	728
Gstm6	1254	1305	1240	1169	1411	1463	1609	1400
Gstm7	767	909	709	789	697	794	858	845
Gsto1	32690	30660	30569	34453	27684	29394	29001	28254
Gsto2	5	10	1	10	2	4	12	0
Gstp-ps	1520	1422	1558	1422	1460	1484	1268	1370
Gstp1	9665	11962	11158	10807	10982	10562	10176	9987
Gstp2	973	756	678	718	584	587	553	579
Gstp3	502	416	381	497	492	392	350	342
Gstt1	2107	2219	2095	1828	2263	2447	2399	1941
Gstt2	1399	1551	1303	1481	1557	1400	1533	1538
Gstt3	2929	3134	3123	2796	3223	3503	3097	3172
Gstt4	0	0	0	0	0	0	1	1
Gstz1	1440	1465	1368	1318	1353	1493	1237	1325
Gtdc1	246	243	281	272	256	295	272	223
Gtf2a1	716	869	770	892	722	758	787	743
Gtf2a1l	0	0	1	0	0	0	0	0
Gtf2a2	1056	1138	1145	1213	1055	1035	1117	1070
Gtf2b	1393	1452	1321	1269	1505	1631	1689	1322
Gtf2e1	427	539	398	407	464	460	435	468
Gtf2e2	504	465	463	508	528	554	511	479
Gtf2f1	2956	3159	2808	2849	2969	3130	3096	2922
Gtf2f2	916	862	1005	799	1066	966	863	872
Gtf2h1	1770	1899	1831	1829	2065	2123	1988	1809
Gtf2h2	430	495	423	523	510	480	453	369
Gtf2h3	695	755	579	707	789	734	701	705
Gtf2h4	454	490	467	462	508	492	440	443
Gtf2h5	871	822	897	840	904	1005	851	823
Gtf2i	1945	2269	2159	2268	2120	2116	1943	1922

Transcriptome sequencing yielded total genetic results for the MOD and APS groups, with a total of 15,936 variables

Continued from above

Gtf2ird1	2308	2530	2255	2186	2118	2062	2069	1897
Gtf2ird2	184	195	168	193	235	223	173	206
Gtf3a	794	861	810	824	820	744	656	733
Gtf3c1	4322	4215	3726	4158	3980	3869	3535	3338
Gtf3c2	2634	2726	2572	2633	2592	2522	2576	2301
Gtf3c3	701	798	661	725	731	783	654	636
Gtf3c4	1085	1289	1240	1198	1304	1397	1181	1192
Gtf3c5	653	673	616	563	668	629	631	610
Gtf3c6	741	790	764	784	820	793	810	722
Gtpbp1	1664	1899	1672	1742	1535	1538	1694	1564
Gtpbp10	559	651	556	574	578	481	570	574
Gtpbp2	4238	4511	4051	4027	3975	4095	4177	3971
Gtpbp3	627	621	627	670	638	622	534	608
Gtpbp4	1219	1256	1180	1204	1349	1312	1237	1204
Gtpbp6	580	606	613	597	531	564	457	512
Gtpbp8	370	369	346	422	317	390	322	303
Gtse1	360	330	340	372	452	405	313	428
Gtsf1	1	0	0	0	0	0	0	0
Guca1a	44	33	51	60	44	41	32	32
Guca1b	7	2	9	11	17	24	13	11
Guca2a	1974	2051	2131	2175	2127	1961	1855	2152
Guca2b	12839	12660	12778	13157	10628	11512	13735	13533
Gucd1	7950	8774	8052	7558	7862	8053	7734	7262
Gucy1a1	333	344	280	349	323	289	301	297
Gucy1a2	10	9	8	8	2	6	5	7
Gucy1b1	322	303	323	341	398	363	342	322
Gucy2c	12655	14265	12264	11472	13239	13600	13661	12832
Gucy2d	0	0	0	0	0	4	0	0
Gucy2g	0	0	1	1	6	2	1	0
Guf1	684	586	568	710	546	555	591	577
Guk1	4173	3953	3820	4289	3732	4033	3841	3615
Gulo	6	5	10	14	3	5	17	6
Gulp1	77	68	65	58	56	54	71	55
Gusb	2093	2038	1988	2064	2208	2290	2178	2102
Gvin1	113	89	203	206	172	48	49	59
Gvin2	27	75	97	72	62	65	108	55
Gvin3	75	100	117	94	75	100	68	57
Gxylt1	728	852	726	778	866	877	721	755
Gxylt2	2	0	0	0	4	4	1	1
Gyg	480	501	495	453	430	485	520	435
Gypa	0	1	0	4	0	0	1	0
Gypc	102	130	120	156	140	89	112	100
Gys1	633	626	661	675	676	645	600	617
Gys2	0	0	1	1	0	0	1	1
Gzf1	1028	946	956	904	969	1064	1072	868
Gzma	1079	1150	1146	1252	725	871	888	675
Gzmb	1028	1077	1147	1200	705	848	775	649
Gzmc	4	11	5	15	12	6	7	8
Gzmk	53	36	40	47	45	58	31	38
Gzmm	15	11	15	23	19	35	1	12
H13	4498	4700	4601	4560	5168	4989	4350	4477
H1f0	11408	12383	11453	11833	10349	10571	10314	10510
H1f1	0	0	0	1	1	1	0	2
H1f10	40	46	41	66	53	59	22	37
H1f2	1258	1344	1165	1062	1200	1216	1190	1210
H1f3	2	17	5	8	21	6	6	8
H1f4	8	5	8	6	1	6	7	10
H1f5	10	2	6	0	1	1	9	1

Transcriptome sequencing yielded total genetic results for the MOD and APS groups, with a total of 15,936 variables

Continued from above

H1f6	0	0	1	0	0	0	0	0
H2-Aa	11152	8564	9022	12487	4994	5230	5831	5938
H2-Ab1	13922	11478	12012	14834	6676	7173	7894	7589
H2-D1	72590	76351	71365	68971	72169	71357	70084	65120
H2-DMa	1972	1465	1779	2055	863	904	906	981
H2-DMb1	1779	1490	1739	2299	718	768	905	948
H2-DMb2	31	25	18	9	33	16	23	21
H2-Eb1	8401	6931	7206	9043	4494	4624	4466	4774
H2-Eb2	15	18	29	12	5	4	17	15
H2-K1	96930	98886	93220	90076	91634	92135	91508	81487
H2-Ke6	552	652	679	627	624	725	574	574
H2-M10.1	0	0	0	0	0	0	0	1
H2-M10.2	0	3	0	0	0	0	0	0
H2-M2	27	36	21	35	15	15	16	10
H2-M3	221	276	226	260	189	195	191	214
H2-M5	0	0	0	0	1	0	0	0
H2-M9	2	14	1	1	2	4	3	9
H2-Oa	9	5	0	5	0	8	0	15
H2-Ob	8	2	4	15	3	4	3	7
H2-Q1	7952	8563	7298	6662	8306	8826	9072	8155
H2-Q10	976	986	948	812	1095	850	859	856
H2-Q2	33093	33467	29355	29365	29278	32443	34251	29359
H2-Q4	5670	5861	5996	5301	4716	4838	4502	4245
H2-Q6	145	140	169	101	113	142	101	108
H2-Q7	236	134	254	195	206	161	107	109
H2-Q9	191	149	177	158	112	154	120	133
H2-T-ps	218	199	184	199	136	170	221	215
H2-T22	4167	4958	4579	4248	4088	3973	3785	3573
H2-T23	20125	22788	20416	18436	19549	19912	20137	18455
H2-T24	347	494	382	315	403	396	364	279
H2-T3	8231	8396	7796	7382	8328	8801	8523	7758
H2ac10	10	4	0	3	7	0	2	0
H2ac11	8	17	7	18	16	7	5	13
H2ac12	0	0	0	0	0	0	5	0
H2ac13	2	4	0	1	4	4	4	6
H2ac15	1	1	0	4	2	7	0	3
H2ac18	29	15	13	11	3	12	21	18
H2ac19	16	7	12	9	15	9	6	11
H2ac20	0	3	1	0	2	6	1	1
H2ac21	0	1	1	0	0	0	6	0
H2ac22	0	0	2	0	0	0	0	0
H2ac23	0	0	2	0	2	3	0	0
H2ac24	1	0	0	1	0	2	0	0
H2ac4	1	5	4	4	6	4	0	1
H2ac6	0	5	2	7	3	5	4	2
H2ac7	0	0	0	0	4	2	3	2
H2ac8	29	19	28	8	30	27	39	21
H2aj	2531	2337	2513	2655	2238	2358	2219	2280
H2al1m	1	0	12	0	0	4	0	5
H2al1o	0	0	0	0	1	0	0	0
H2ax	1977	2005	2032	1811	1945	1930	1705	1798
H2az1	8459	8911	8821	8447	9291	9227	8488	8641
H2az2	2361	2679	2880	2604	2674	2846	2304	2372
H2bc11	10	14	19	1	17	16	3	11
H2bc12	10	1	0	4	8	10	4	0
H2bc13	1	2	1	0	0	1	4	2
H2bc14	1	1	0	6	2	0	4	1
H2bc15	1	3	4	7	1	1	0	1

Transcriptome sequencing yielded total genetic results for the MOD and APS groups, with a total of 15,936 variables

Continued from above

H2bc18	0	1	1	5	4	0	1	1
H2bc21	129	138	110	123	119	127	125	116
H2bc22	0	4	2	0	1	1	0	4
H2bc23	0	0	0	2	5	1	3	1
H2bc24	0	0	1	0	0	0	0	0
H2bc3	1	5	1	1	0	6	2	0
H2bc4	1539	1861	1703	1607	2192	2219	2106	2041
H2bc6	25	22	13	17	37	26	28	26
H2bc7	1	3	11	3	11	5	4	1
H2bc8	7	14	7	13	15	10	8	25
H2bc9	9	7	2	12	12	14	8	9
H2bu2	8	1	12	7	5	4	0	3
H3c1	1	6	3	3	1	0	4	6
H3c10	6	10	1	8	6	4	0	0
H3c11	1	1	4	2	6	12	4	4
H3c13	0	2	1	0	16	2	4	2
H3c14	12	15	21	9	14	15	24	27
H3c15	12	15	5	9	32	12	28	10
H3c2	3	0	0	0	0	0	0	1
H3c3	20	10	22	20	26	19	20	20
H3c4	9	0	1	7	15	4	6	12
H3c6	0	1	8	5	4	0	0	1
H3c7	4	4	4	6	6	4	4	6
H3c8	5	6	17	5	14	8	12	10
H3f3a	14862	15495	14555	14291	14434	15616	14613	14236
H3f3b	16283	16912	15719	15877	14450	14982	14659	14584
H3f3c	63	30	37	58	48	59	44	44
H4c1	0	4	0	6	3	1	1	5
H4c11	3	6	1	16	2	6	3	12
H4c12	17	15	6	7	18	23	25	27
H4c14	0	4	4	1	6	0	0	1
H4c17	4	2	0	3	16	11	6	0
H4c18	6	0	0	1	1	0	2	2
H4c2	6	8	5	4	5	1	6	8
H4c3	0	6	6	9	10	6	2	14
H4c4	4	3	8	5	5	19	5	1
H4c6	1	0	10	1	1	1	1	0
H4c8	6	15	11	2	14	8	17	8
H4c9	105	108	112	151	149	157	138	133
H4f16	0	13	2	0	2	3	1	1
H60b	0	0	0	1	0	0	0	0
H6pd	2196	2606	2368	2272	2669	2596	2420	2672
Haao	4	2	3	2	7	4	2	9
Habp2	177	174	214	230	136	175	203	202
Habp4	606	616	697	684	632	591	595	513
Hacd1	620	640	669	657	699	677	713	672
Hacd2	1004	1060	1138	1002	1050	1086	973	986
Hacd3	1837	2156	2024	1930	2042	2267	1958	2094
Hacd4	127	102	101	126	147	98	113	90
Hace1	956	1138	1065	956	1054	972	1010	852
Hacl1	352	427	387	344	398	442	385	383
Hadh	24471	26016	24420	23386	25026	27148	26136	23672
Hadha	16338	17039	15457	15875	17317	17557	16663	15656
Hadhb	7785	7725	7287	7662	7786	7936	7667	7283
Hagh	5204	4881	5092	5410	4092	4329	4346	4014
Haghl	171	144	187	192	127	136	147	133
Hal	15	4	11	7	7	10	10	10
Hamp	11	1	0	0	0	14	2	0

Transcriptome sequencing yielded total genetic results for the MOD and APS groups, with a total of 15,936 variables

Continued from above

Hamp2	1	4	0	0	6	5	2	15
Hand1	52	35	63	47	52	65	82	41
Hand2	72	71	121	109	52	74	85	84
Hao1	0	4	0	1	2	0	1	0
Hao2	1	2	1	2	9	7	5	3
Hap1	109	149	142	115	80	101	118	127
Hapln1	0	0	0	0	0	1	0	3
Hapln2	0	1	0	0	0	0	0	0
Hapln3	6	6	5	5	2	4	6	0
Hapln4	19	18	20	18	14	13	20	24
Harbi1	212	268	267	242	255	251	240	267
Hars	1549	1766	1508	1569	1723	1644	1623	1507
Hars2	732	711	794	804	849	773	820	763
Has1	0	6	0	13	0	6	3	13
Has2	22	41	16	26	17	30	18	12
Has3	5	6	10	5	7	5	10	6
Haspin	359	413	440	490	417	430	386	302
Hat1	892	1080	923	926	1082	1085	1000	837
Haus1	191	255	230	230	181	167	178	211
Haus2	807	816	716	720	770	731	790	649
Haus3	134	170	169	201	212	199	187	176
Haus4	358	412	423	396	410	352	287	332
Haus5	266	289	231	226	257	289	253	220
Haus6	232	296	240	303	340	303	271	202
Haus7	463	481	470	483	509	469	400	498
Haus8	318	288	271	333	298	308	270	309
Havcr1	0	0	0	0	0	0	6	0
Havcr2	49	46	61	48	29	14	26	51
Hax1	950	1078	973	1065	1085	1128	1111	1022
Hba-a1	0	1	10	3	0	0	0	1
Hba-a2	0	0	1	0	0	0	0	0
Hbb-bs	1	4	12	2	0	0	0	1
Hbb-bt	3	0	3	1	0	0	1	0
Hbegf	592	655	674	666	612	741	862	673
Hbp1	2300	2354	2325	2312	2494	2411	2348	2296
Hbs1l	2213	2231	2226	2234	2613	2378	2371	2319
Hc	2	10	2	3	9	1	1	3
Hcar1	35	72	58	69	84	53	52	54
Hcar2	11	10	8	7	1	14	2	6
Hccs	3258	2693	1820	2362	2331	2528	2857	3407
Hcfc1	4211	4505	4730	4578	4378	4179	3949	3752
Hcfc1r1	1687	1673	1708	1667	1554	1558	1491	1510
Hcfc2	665	652	666	713	789	720	746	625
Hck	345	402	300	363	307	297	304	288
Hcls1	418	385	429	437	321	393	297	351
Hcn1	39	2	18	16	16	11	16	15
Hcn2	410	378	363	453	328	323	311	300
Hcn3	14	15	20	12	14	18	16	17
Hcn4	23	61	36	39	33	43	30	30
Hcrtr1	18	11	30	11	13	14	13	16
Hcst	28	36	23	36	45	54	39	32
Hdac1	3080	3139	3124	3313	3103	3293	3013	2996
Hdac10	266	310	275	270	358	239	255	212
Hdac11	1133	1153	1277	1273	1088	1011	1023	997
Hdac2	1320	1369	1412	1350	1454	1418	1387	1276
Hdac3	2791	3013	2783	2718	2797	2792	2531	2646
Hdac4	1095	1098	1053	1006	1102	1153	996	1047
Hdac5	1041	1127	1069	934	1165	1104	1039	1031

Transcriptome sequencing yielded total genetic results for the MOD and APS groups, with a total of 15,936 variables

Continued from above

Hdac6	277	252	290	272	235	262	280	218
Hdac7	331	322	362	323	323	312	312	300
Hdac8	234	220	213	221	182	191	221	213
Hdac9	56	78	44	57	65	54	41	30
Hdc	22	34	41	25	18	12	22	11
Hddc2	203	240	232	240	317	217	255	269
Hddc3	133	76	118	144	127	120	130	128
Hdgf	6050	6647	6296	6044	6250	6477	5707	5855
Hdgfl2	715	819	747	737	779	846	697	691
Hdgfl3	119	119	96	95	80	72	113	72
Hdhd2	1225	1297	1344	1301	1472	1394	1233	1251
Hdhd3	4104	4184	4008	4068	3882	4170	4081	3903
Hdhd5	377	377	412	450	358	432	316	403
Hdlbp	21503	24299	21744	21498	24253	24462	23190	22404
Hdx	6	5	3	3	5	11	5	1
Heatr1	1012	1116	933	990	1134	1108	994	1001
Heatr3	684	672	592	590	758	727	589	643
Heatr4	7	17	16	7	3	3	3	12
Heatr5a	7274	8229	7393	7011	8573	9017	8506	7316
Heatr5b	1203	1323	1223	1209	1238	1182	1293	1094
Heatr6	1166	1393	1121	1283	1333	1285	1232	1180
Heatr9	2	0	2	4	1	1	2	1
Hebp1	146	91	135	165	139	144	125	123
Hebp2	49	49	57	52	51	43	63	73
Heca	711	827	714	789	746	819	798	728
Hectd1	4700	5250	5003	4947	5339	5408	5098	4831
Hectd2	32	31	30	28	47	43	44	36
Hectd3	8935	9769	8898	8963	8866	9075	8931	8771
Hectd4	3250	3633	3531	3394	3301	3203	3282	3084
Hecw1	0	0	4	6	0	1	0	1
Hecw2	27	39	24	51	45	37	22	43
Heg1	944	1085	966	840	1051	1115	894	929
Helb	649	687	698	564	552	602	599	609
Hells	684	789	807	682	820	921	762	653
Helq	230	262	253	259	289	249	240	236
Helz	1645	1791	1764	1658	1572	1740	1450	1530
Helz2	9931	12034	11219	9830	10794	10746	10019	8877
Hemgn	26	8	33	5	19	8	9	12
Hemk1	132	149	148	133	149	135	181	165
Henmt1	0	0	0	0	0	0	0	2
Hepacam	1	10	13	3	11	5	5	4
Hepacam2	1402	1507	1607	1730	1495	1604	1595	1493
Heph	11774	13816	10603	10031	13518	13797	14653	12334
Hephl1	0	5	4	4	1	3	1	0
Herc1	3344	3624	3558	3429	3322	3618	3471	3108
Herc2	3937	4376	4137	4457	4053	4328	3966	3898
Herc3	904	1095	1024	868	698	732	706	722
Herc4	1841	2061	1606	1745	1692	1891	1866	1684
Herc6	3931	4973	3424	2636	3882	4540	4154	3369
Herpud1	3615	4162	4813	4436	4551	4453	4583	4921
Herpud2	1253	1359	1198	1273	1234	1289	1245	1114
Hes1	1159	1035	1132	1008	1306	1470	1147	1166
Hes2	218	212	187	244	260	294	241	269
Hes5	1	0	0	6	1	0	1	6
Hes6	1553	1490	1658	1515	1497	1557	1429	1508
Hes7	4	0	1	0	1	1	2	5
Hesx1	1	1	1	1	2	0	2	1
Hexa	4178	4276	4188	4277	4453	4377	4235	4210

Transcriptome sequencing yielded total genetic results for the MOD and APS groups, with a total of 15,936 variables

Continued from above

Hexb	2854	3193	2827	2887	2885	3104	2818	2616
Hexdc	471	552	603	594	528	600	514	589
Hexim1	2083	2040	2069	2053	2111	2248	2089	2039
Hexim2	54	26	53	26	48	40	27	29
Hey1	56	38	46	72	38	27	38	61
Hey2	9	26	16	26	16	13	10	5
Heyl	50	48	28	27	23	9	21	15
Hfe	204	282	236	207	317	265	201	270
Hgd	1	4	1	5	3	4	3	1
Hgf	86	92	48	60	77	100	80	50
Hgfac	736	704	760	744	839	803	687	770
Hgh1	290	315	360	287	316	401	310	336
Hgs	3080	3383	3016	2931	3454	3257	3218	3018
Hgsnat	845	996	971	908	1033	972	848	980
Hhat	67	70	70	70	52	53	60	42
Hhatl	9	8	24	13	12	17	19	5
Hhex	40	27	34	42	40	36	33	44
Hhip	664	777	793	757	636	576	627	500
Hhipl1	7	13	18	1	4	6	20	2
Hhipl2	16	29	17	32	31	17	43	35
Hibadh	2373	2646	2523	2473	2762	2789	2390	2425
Hibch	655	698	672	725	772	734	664	641
Hic1	517	455	448	442	525	529	374	419
Hic2	203	245	240	272	259	246	291	240
Hid1	721	750	873	796	740	877	645	698
Hif1an	3146	3448	3125	3058	3363	3683	3494	3201
Hif3a	30	21	22	21	13	28	29	22
Higd1a	1553	1605	1591	1520	1569	1666	1518	1657
Higd1b	1	1	10	5	4	4	0	15
Higd2a	3577	3572	3356	3670	3267	3713	3396	3312
Hikeshi	592	747	614	671	725	780	660	702
Hilpda	79	46	73	47	85	82	64	68
Hinfp	928	887	840	1001	997	915	913	887
Hint1	6270	6787	6824	6256	7246	7514	6485	6531
Hint2	1024	1022	1043	1005	1035	1022	1032	941
Hint3	976	1047	1094	1054	1076	1064	1054	1061
Hip1	292	317	262	337	332	257	289	231
Hip1r	6838	7470	7010	6387	6795	7338	6598	6537
Hipk1	3762	3945	4209	4082	4211	4159	4144	3788
Hipk2	6351	7964	7479	7585	7298	7694	7304	6475
Hipk3	1938	2222	2143	2247	2395	2466	2307	2146
Hipk4	15	13	8	12	10	11	16	13
Hira	1679	1871	1727	1712	1787	1671	1514	1695
Hirip3	331	445	353	372	415	437	302	386
Hivep1	1608	1612	1593	1766	1584	1555	1459	1397
Hivep2	615	748	752	651	774	748	881	714
Hivep3	24	41	35	24	46	20	33	32
Hjurp	4128	4317	4477	4412	4608	4540	4028	4085
Hjv	0	0	0	1	0	0	0	1
Hk1	573	557	546	644	546	472	538	516
Hk2	2169	2581	2106	1958	2614	2503	2339	2495
Hk3	179	196	207	213	184	165	168	168
Hkdc1	16796	16277	16130	17549	13773	13798	14006	13661
Hlcs	248	296	246	248	221	257	206	221
Hlf	15	24	43	25	37	48	37	45
Hltf	758	824	827	800	1112	979	998	836
Hlx	161	118	117	119	117	134	79	125
Hmbox1	1642	1891	1857	1816	1795	1818	1727	1512

Transcriptome sequencing yielded total genetic results for the MOD and APS groups, with a total of 15,936 variables

Continued from above

Hmbs	1396	1546	1360	1490	1399	1455	1302	1437
Hmces	309	358	355	308	314	387	303	288
Hmcn1	215	242	265	291	239	281	208	213
Hmcn2	901	1170	1085	1036	899	949	937	805
Hmg20a	612	713	715	612	771	733	681	657
Hmg20b	2955	2484	2679	2859	2327	2497	2677	2497
Hmga1	3944	3735	3794	3731	3676	3374	3070	3489
Hmga1b	417	399	412	366	369	423	429	406
Hmga2	231	210	215	148	162	181	156	170
Hmgb1	2396	2664	2470	2456	2721	2546	2309	2476
Hmgb2	4286	4565	4391	4004	4845	4382	3920	4361
Hmgb3	1177	1120	1135	1133	1028	951	947	893
Hmgcl	3089	3168	2994	3115	3170	3054	2978	3302
Hmgcll1	2	6	5	2	7	2	2	1
Hmgcr	5377	5274	5505	5560	5176	5283	4994	5166
Hmgcs1	6486	6573	7122	7048	6440	7346	7130	6588
Hmgcs2	510	475	397	416	680	606	455	380
Hmgn1	2124	2124	2025	2102	2233	2160	1985	2029
Hmgn2	2459	2831	2685	2671	2905	2763	2402	2731
Hmgn3	56	63	91	49	76	91	71	59
Hmgn5	838	778	699	814	904	976	948	891
Hmgxb3	967	1097	925	937	1045	978	944	832
Hmgxb4	556	688	519	628	740	717	699	595
Hmmr	567	734	690	650	796	855	713	690
Hmox2	1647	1607	1454	1596	1543	1533	1465	1472
Hmx2	7	6	1	1	1	0	7	3
Hmx3	1	21	5	14	10	9	8	4
Hnf1a	1945	1940	2046	1933	2313	2336	1875	2050
Hnf1b	472	417	452	409	486	498	465	431
Hnf4a	38753	40575	38071	38685	41408	41570	38850	35862
Hnf4g	21060	24099	21531	20299	22336	23564	22906	20360
Hnmt	29	21	19	40	37	60	25	15
Hnrnpa0	6917	7078	6751	6407	6726	6459	5781	6212
Hnrnpa1	4811	5293	5168	5051	4656	4830	4379	4352
Hnrnpa1l2	0	1	0	1	0	1	0	0
Hnrnpa2b	17016	18989	18098	17899	19164	19177	16941	17043
Hnrnpa3	1860	2001	1855	1900	1979	1995	1898	1805
Hnrnpab	13637	14252	14040	13359	14150	14400	12861	13233
Hnrnpc	6081	6767	6451	6292	7356	7445	6543	6213
Hnrnpd	4213	4514	3928	4056	3175	3477	3146	3118
Hnrnpdl	2391	2660	2570	2597	2296	2324	2171	2058
Hnrnpf	8440	8992	8945	8776	9125	8988	8466	8229
Hnrnph1	3482	3669	3559	3640	4264	4355	3957	3909
Hnrnph2	2173	2340	2211	2392	2388	2409	2439	2227
Hnrnph3	667	698	700	730	790	793	718	824
Hnrnpk	13309	14366	13517	13392	14106	13761	12895	12916
Hnrnpl	6645	6811	7141	7252	6480	6242	5989	6024
Hnrnpll	2139	2352	2551	2329	2332	2250	2147	2154
Hnrnpm	5342	5824	5669	5431	6050	6138	5233	5408
Hnrnpr	1667	1743	1682	1638	1722	1852	1619	1546
Hnrnpu	11138	12031	11319	11245	12948	13043	11886	11290
Hnrnpul1	5869	6298	5878	5631	5529	5500	5248	5156
Hnrnpul2	9688	10389	10159	9842	10510	10634	10370	9712
Hoga1	21	18	27	26	36	11	11	23
Homer1	756	766	678	734	874	804	806	673
Homer2	1275	1364	1373	1259	1110	1461	1281	1180
Homer3	163	131	191	131	175	191	154	162
Homez	258	232	236	225	193	192	183	207

Transcriptome sequencing yielded total genetic results for the MOD and APS groups, with a total of 15,936 variables

Continued from above

Hook1	6748	7628	7051	6774	7722	8002	7688	6911
Hook2	5437	5633	5556	5633	5014	5660	5521	5427
Hook3	550	667	617	641	791	782	685	636
Hopx	1428	1562	1672	1698	1899	1882	1524	1652
Hormad1	0	1	2	1	0	0	0	0
Hormad2	5	1	1	1	0	0	0	0
Hoxa1	7	9	1	4	4	7	3	7
Hoxa10	11	7	9	22	2	5	1	0
Hoxa2	30	46	15	37	32	35	24	26
Hoxa3	101	114	122	111	131	126	78	97
Hoxa4	36	56	50	66	41	35	59	66
Hoxa5	86	103	103	93	90	85	100	62
Hoxa6	11	3	9	10	10	8	17	16
Hoxa7	18	18	16	21	5	25	13	22
Hoxa9	22	32	26	22	1	13	11	10
Hoxb1	1	0	0	0	0	0	0	0
Hoxb13	1	2	1	1	13	9	11	6
Hoxb2	89	80	72	76	92	67	50	100
Hoxb3	72	50	46	77	74	86	55	42
Hoxb4	80	59	70	47	60	53	49	50
Hoxb5	55	59	64	79	68	73	88	67
Hoxb6	18	11	21	34	20	41	25	23
Hoxb7	9	15	16	12	13	29	33	15
Hoxb8	1	11	1	3	0	5	5	18
Hoxb9	2	2	5	0	0	0	1	2
Hoxc10	0	1	0	0	0	0	0	0
Hoxc4	30	43	26	40	31	50	22	34
Hoxc5	12	14	27	21	32	23	27	14
Hoxc6	26	11	26	26	11	15	9	16
Hoxd3	0	1	0	7	1	1	1	0
Hoxd4	7	11	7	9	14	13	3	0
Hoxd8	19	4	26	16	5	22	7	11
Hoxd9	5	0	1	3	2	0	0	1
Hp	45	106	73	30	62	60	103	76
Hp1bp3	3433	3965	3872	3585	3636	3644	3423	3327
Hpca	23	20	28	28	20	31	28	31
Hpcal1	1271	1300	1256	1391	1193	1143	1041	1093
Hpcal4	25	19	40	12	23	11	16	20
Hpd	300	262	310	289	278	322	218	326
Hpdl	271	288	311	294	326	299	263	350
Hpf1	335	437	406	417	400	419	369	364
Hpgd	3462	3698	3656	3569	3220	3856	3888	3568
Hpgds	74	72	90	81	71	89	75	50
Hpn	32	17	50	30	28	45	12	49
Hprt	2879	3201	2862	2678	3048	3003	2851	2694
Hps1	460	479	491	511	412	471	418	415
Hps3	452	432	447	537	481	402	414	367
Hps4	738	793	690	772	732	759	751	700
Hps5	2665	2954	2630	2808	2825	2943	2667	2339
Hps6	248	277	292	269	255	226	247	247
Hpse	782	848	862	799	803	741	674	716
Hpse2	82	91	150	124	136	90	110	95
Hpx	68	115	66	40	69	49	78	64
Hr	567	640	684	554	657	606	583	526
Hras	1560	1574	1595	1684	1809	1757	1533	1468
Hrc	9	11	8	25	0	22	17	8
Hrct1	19	20	12	18	12	2	16	13
Hrg	4	7	11	1	10	10	4	0

Transcriptome sequencing yielded total genetic results for the MOD and APS groups, with a total of 15,936 variables

Continued from above

Hrh1	71	52	47	52	59	31	63	34
Hrh2	14	13	10	7	14	23	10	13
Hrh3	2	8	4	1	2	0	1	9
Hrob	128	129	113	102	101	130	76	144
Hs1bp3	408	441	496	462	457	440	380	504
Hs2st1	832	923	798	855	921	858	867	849
Hs3st1	367	348	336	368	370	395	286	346
Hs3st2	2	0	6	0	5	0	2	1
Hs3st3a1	10	6	11	8	7	12	9	18
Hs3st3b1	85	124	96	68	98	112	83	128
Hs3st4	1	5	5	4	1	0	0	0
Hs3st5	10	9	5	1	14	7	13	10
Hs3st6	4	9	19	8	14	2	7	11
Hs6st1	2387	2772	3084	2630	2961	3064	2804	2999
Hs6st2	1	1	7	7	3	0	0	1
Hs6st3	0	0	0	2	0	0	0	0
Hsbp1	2542	2654	2701	2642	2511	2751	2715	2456
Hsbp1l1	9	15	6	6	15	2	8	16
Hscb	393	405	371	375	308	385	361	316
Hsd11b1	265	359	278	307	242	224	242	302
Hsd11b2	173	164	279	180	240	213	196	217
Hsd17b1	1	0	0	1	2	9	1	1
Hsd17b10	1416	1561	1525	1488	1401	1532	1354	1398
Hsd17b11	10378	12019	10024	9262	12509	13326	12829	11762
Hsd17b12	5348	5437	5487	5360	5924	5552	5391	5296
Hsd17b13	521	517	510	411	721	683	587	608
Hsd17b14	2	0	0	0	0	0	4	4
Hsd17b2	564	768	516	465	707	782	704	688
Hsd17b4	7159	8481	7501	7056	8952	9066	8191	7638
Hsd17b6	3299	3644	3100	2677	3734	3790	3870	3945
Hsd17b7	1465	1488	1492	1377	1580	1555	1595	1523
Hsd3b2	1	0	3	7	11	7	2	2
Hsd3b3	40	53	90	15	109	103	75	74
Hsd3b7	953	1023	1057	1015	959	952	982	977
Hsdl1	955	1089	965	959	946	1025	1097	944
Hsdl2	1497	1753	1596	1481	1885	1927	1993	1798
Hsf1	568	621	613	518	671	667	470	622
Hsf2	142	107	100	107	128	113	148	118
Hsf2bp	7	6	5	2	7	0	4	1
Hsf3	21	39	17	15	16	21	26	11
Hsf4	12	17	14	17	11	17	16	17
Hsf5	39	46	27	45	23	28	57	44
Hsh2d	325	358	263	232	331	299	265	269
Hsp90aa1	7597	8247	7765	7579	12729	13150	11509	9362
Hsp90ab1	29733	34851	34392	31191	41156	39984	32164	32883
Hsp90b1	20339	22751	21104	20129	26757	27272	24427	22528
Hspa12a	204	259	223	192	228	249	253	214
Hspa12b	99	118	96	89	81	60	93	79
Hspa13	1438	1435	1365	1271	1840	1777	1678	1636
Hspa14	1266	1189	1096	1162	1183	1179	991	1141
Hspa1a	51	74	49	64	401	211	73	265
Hspa1b	195	156	148	167	1367	720	264	884
Hspa1l	11	14	9	8	8	4	8	2
Hspa2	72	62	90	100	98	52	89	87
Hspa4	8153	8772	8223	7989	9546	9388	8779	8409
Hspa4l	700	873	791	811	1298	1184	1034	973
Hspa5	24476	27262	23797	23483	36824	35394	30320	28917
Hspa8	40833	44766	42624	41634	76463	73118	58676	55066

Transcriptome sequencing yielded total genetic results for the MOD and APS groups, with a total of 15,936 variables

Continued from above

Hspa9	14846	15914	14889	15012	15482	15534	14034	14177
Hspb1	323	286	352	316	348	320	327	344
Hspb11	90	44	83	71	71	85	81	61
Hspb2	8	15	17	23	14	2	13	9
Hspb3	0	0	0	0	0	1	0	0
Hspb6	334	305	330	361	292	218	273	283
Hspb7	152	123	172	190	125	137	168	133
Hspb8	159	189	192	185	234	255	176	206
Hspb9	0	4	2	4	0	0	0	0
Hspbap1	325	302	319	321	380	340	277	400
Hspbp1	899	865	968	793	1034	882	797	836
Hspd1	12097	13637	12824	12137	15072	15222	12783	11665
Hspe1	3631	3880	3830	3756	4661	4387	3731	3596
Hspg2	2561	2885	2996	2924	2465	2563	2500	2260
Hsph1	1674	1869	1742	1633	6421	5794	3536	3939
Htatip2	1976	2041	1950	1868	2144	1962	1770	1941
Htatsf1	897	908	957	824	847	906	823	788
Htr1b	5	2	8	9	6	12	4	1
Htr1d	0	1	5	8	0	8	0	0
Htr2a	0	2	0	0	1	1	0	0
Htr2b	1	21	4	11	12	14	6	15
Htr2c	0	0	0	0	0	0	3	1
Htr3a	46	66	56	72	67	33	59	65
Htr3b	4	3	4	5	1	8	6	7
Htr4	23	41	45	58	55	38	28	28
Htr5a	0	0	0	0	0	1	0	0
Htr5b	0	0	0	1	0	0	0	0
Htr6	52	19	27	26	14	13	11	17
Htr7	5	2	11	4	6	1	3	0
Htra1	535	503	667	593	462	482	428	398
Htra2	651	702	696	658	614	671	602	637
Htra3	484	599	563	572	554	512	555	481
Htra4	13	14	15	10	13	8	5	5
Htt	2087	2232	2267	2018	2052	2054	1808	1898
Hunk	164	202	182	155	170	231	161	160
Hus1	315	271	298	215	318	341	298	246
Hus1b	0	0	1	0	0	0	1	0
Huwe1	7025	8323	8071	7889	7291	7302	7031	6617
Hvcn1	254	199	155	234	242	208	185	188
Hyal1	58	45	54	59	74	33	55	75
Hyal2	3398	3231	3286	3426	3429	3549	3307	3360
Hyal3	10	16	9	12	5	14	1	14
Hydin	1	13	4	7	7	4	5	12
Hykk	60	55	88	42	53	60	53	36
Hyls1	94	104	98	75	117	143	87	153
Hyou1	6102	6893	6047	6029	9437	8854	7552	7521
Hypk	1218	1292	1246	1288	1322	1402	1206	1208
I830077J0	85	107	92	93	97	69	125	82
Iah1	1481	1779	1610	1525	1528	1624	1536	1486
Iapp	10	28	23	19	25	15	18	6
Iars	2297	2485	2499	2216	2522	2368	2136	2250
Iars2	2478	2547	2524	2451	2441	2556	2344	2375
Iba57	682	664	558	775	804	724	749	690
Ibsp	0	1	1	4	0	0	0	0
Ibtk	1931	2295	2143	2195	2311	2253	2216	2110
Ica1	210	252	277	295	286	284	239	229
Ica1l	4	2	7	8	2	1	6	12
Icam1	484	569	503	492	442	379	426	370

Transcriptome sequencing yielded total genetic results for the MOD and APS groups, with a total of 15,936 variables

Continued from above

Icam2	59	50	39	59	40	47	55	54
Icam4	12	6	10	8	6	0	17	7
Icam5	8	5	2	4	2	3	7	1
Ice1	954	1084	1049	1168	1169	1156	1075	1073
Ice2	265	296	290	291	258	274	252	277
Icmt	3199	3344	3071	3000	3300	3377	3402	3310
Icos	24	27	15	28	19	25	29	28
Icosl	329	460	360	378	355	349	282	357
Id1	4204	4337	4489	4138	4302	4512	3930	3927
Id2	893	940	908	891	912	1054	1039	897
Id3	3133	3265	3068	3110	3435	3343	2834	3215
Id4	208	269	312	265	233	233	217	252
Ide	2112	2446	2183	2135	2588	2545	2132	2271
Idh1	11371	13699	13119	11593	15820	16354	15034	14383
Idh2	2827	2710	2818	2807	2886	2836	2412	2730
Idh3a	9535	9474	10097	10130	9221	9429	9180	8781
Idh3b	9909	9567	9841	10260	9519	9601	8825	8880
Idh3g	6056	5961	5966	6138	5423	5518	5484	5318
Idi1	1378	1403	1655	1510	1633	1509	1395	1589
Idnk	1192	1227	1144	1173	1102	1091	1050	897
Ido1	1608	1431	1343	1646	666	816	632	659
Ido2	1	5	2	5	7	8	1	4
Ids	519	567	602	518	636	583	496	501
Idua	363	392	369	341	390	421	406	357
Ier2	2048	2068	2070	1987	2102	2082	1963	1911
Ier3	301	357	284	271	284	274	272	255
Ier3ip1	1590	1524	1532	1667	1674	1702	1596	1603
Ier5	3489	3678	3342	3143	3608	3516	3322	3318
Ier5l	93	80	49	48	72	85	65	85
Iffo1	57	38	88	55	59	63	42	83
Iffo2	241	290	294	267	270	223	257	274
Ifi203	311	410	317	327	237	301	264	174
Ifi204	208	254	223	196	118	183	212	177
Ifi205	86	67	128	64	75	85	82	60
Ifi206	109	136	82	123	54	66	67	70
Ifi207	224	154	203	174	180	147	195	163
Ifi208	75	70	83	65	56	63	44	49
Ifi209	98	106	96	86	51	105	72	93
Ifi211	121	135	109	117	87	84	99	95
Ifi213	90	137	137	162	51	77	74	94
Ifi214	13	2	1	6	6	2	7	0
Ifi27	1103	1023	1181	1056	1212	1221	1205	1122
Ifi27l2a	1258	1381	1349	1217	1331	1202	1106	974
Ifi27l2b	27447	31381	30771	27700	27956	29477	29755	24918
Ifi30	5169	5747	5788	5442	5454	5264	5296	5274
Ifi35	5333	5862	5527	5014	5159	5563	5170	5055
Ifi44	3081	4310	3845	2776	4405	4337	3893	3481
Ifi47	5200	5467	4736	4671	3495	3663	3331	2845
Ifih1	4661	5840	4775	3906	4873	5231	5444	4624
Ifit1	5243	7608	6467	4068	5587	6710	6615	5439
Ifit1bl1	18121	27432	19431	11663	18531	22840	20008	17112
Ifit1bl2	8224	9062	7428	7365	8265	8864	9128	7953
Ifit2	2465	3199	2553	1854	1541	1889	1482	1209
Ifit3	1169	1785	1493	980	1328	1308	1366	1189
Ifit3b	172	267	249	187	238	255	253	199
Ifitm1	447	554	519	449	422	402	429	476
Ifitm10	9	7	2	10	6	11	5	10
Ifitm2	1952	2056	2315	2035	2009	2028	1898	2207

Transcriptome sequencing yielded total genetic results for the MOD and APS groups, with a total of 15,936 variables

Continued from above

Ifitm3	5288	6435	6039	5198	6286	5526	4771	5492
Ifitm5	1	3	0	2	10	2	9	1
Ifitm6	7	1	6	0	5	1	2	5
Ifitm7	0	1	0	0	0	0	0	0
Ifna4	0	1	0	0	0	0	0	0
Ifnar1	2262	2613	2493	2465	2402	2309	2432	2137
Ifnar2	937	1015	966	1162	928	878	958	975
Ifnb1	0	1	4	0	0	0	0	0
Ifne	0	0	0	0	0	1	2	0
Ifng	30	15	8	9	1	0	2	1
Ifngr1	5573	6284	6301	6004	5411	5646	5767	6095
Ifngr2	10394	10134	10402	11340	8972	9195	9715	8902
Ifnl2	0	5	6	0	1	0	0	1
Ifnl3	2	1	1	0	6	0	1	0
Ifnlr1	914	957	937	961	882	956	1024	904
Ifnz	2	0	0	0	0	0	0	0
Ifrd1	2572	2721	2687	2453	3271	3460	3908	3512
Ifrd2	1669	1827	1634	1614	1953	1771	1870	1788
Ift122	83	115	98	89	84	90	107	112
Ift140	447	534	483	578	531	578	517	522
Ift172	1050	1107	1038	1072	999	1068	910	957
Ift20	2842	2878	2683	2707	2606	3191	2958	2739
Ift22	1494	1624	1682	1467	1584	1724	1633	1549
Ift27	186	210	155	128	128	112	139	170
Ift43	411	428	438	433	373	414	318	379
Ift46	816	726	831	810	716	751	710	748
Ift52	932	803	924	873	882	964	799	834
Ift57	38	39	26	32	18	43	50	33
Ift74	106	104	75	86	99	91	60	90
Ift80	110	121	113	91	131	131	110	122
Ift81	42	83	71	57	70	56	58	41
Ift88	190	177	193	212	224	218	229	173
Iftap	107	105	84	73	63	87	86	63
Igbp1	2017	2106	2102	2311	2101	2138	2255	2070
Igdcc3	0	0	1	0	1	0	1	0
Igdcc4	63	47	54	56	68	63	73	59
Igf1r	284	421	361	354	316	314	225	262
Igf2	106	171	80	148	97	103	99	84
Igf2bp1	0	4	4	0	0	0	0	0
Igf2bp2	2267	2202	2142	2007	2301	2182	2148	2297
Igf2bp3	37	70	46	38	36	37	13	42
Igf2r	2991	3396	3282	3233	3478	3479	3416	3125
Igfals	8	1	1	1	7	2	3	2
Igfbp1	2	2	13	15	4	3	3	4
Igfbp2	15	26	40	55	33	28	23	27
Igfbp3	1501	1439	1558	1315	1208	1165	1189	1169
Igfbp4	1901	2108	2126	2018	2213	2039	1860	1849
Igfbp5	452	593	489	475	631	502	544	482
Igfbp6	75	42	123	113	142	150	208	182
Igfbp7	2895	2794	2792	2750	2966	2650	2501	2375
Igfbpl1	4	0	0	2	0	0	0	0
Igflr1	63	78	77	73	102	112	77	90
Ighmbp2	233	335	296	215	324	205	260	249
Iglon5	6	7	1	8	5	18	6	1
Igsf1	0	0	0	0	0	0	0	4
Igsf10	126	220	115	146	115	154	119	129
Igsf11	5	22	17	16	12	4	25	10
Igsf21	14	5	11	5	8	0	1	5

Transcriptome sequencing yielded total genetic results for the MOD and APS groups, with a total of 15,936 variables

Continued from above

Igsf23	7665	8044	7153	7150	8107	8848	8934	7936
Igsf3	1305	1395	1401	1283	1322	1438	1605	1370
Igsf5	3497	3519	3052	3215	3393	3420	3436	3317
Igsf6	91	70	113	59	86	133	104	81
Igsf8	682	575	691	550	654	609	486	529
Igsf9	7032	6875	6466	6717	5308	5321	5194	4710
Igsf9b	380	364	302	447	339	307	245	304
Igtp	12504	14347	11249	10342	7763	8746	6793	6109
Ihh	2876	3008	2808	2916	3096	3404	3102	2728
Iho1	0	0	1	1	0	1	1	1
Iigp1	1815	1956	1698	1764	924	1063	939	692
Ik	2605	2772	2799	2545	2757	2977	2835	2838
Ikbip	282	253	261	221	315	326	329	286
Ikbkb	1434	1420	1373	1359	1355	1355	1240	1409
Ikbke	1906	2001	1795	1808	1590	1684	1650	1646
Ikbkg	1383	1581	1427	1459	1475	1413	1308	1557
Ikzf1	184	180	145	156	154	158	184	135
Ikzf2	24	23	31	41	19	14	12	9
Ikzf3	113	114	99	111	102	101	71	81
Ikzf4	26	17	16	20	17	24	12	16
Ikzf5	575	721	652	661	585	673	684	608
Il10	2	9	1	2	1	7	6	6
Il10ra	165	120	169	171	194	140	130	117
Il10rb	4436	4678	4593	4553	4125	4286	4459	4378
Il11	28	20	25	13	19	9	9	14
Il11ra1	372	499	377	408	409	410	453	457
Il11ra2	0	0	0	0	1	0	0	0
Il12b	11	17	12	6	1	3	3	1
Il12rb1	128	115	117	138	112	83	93	76
Il12rb2	48	55	76	48	29	39	71	56
Il13	0	0	4	0	0	0	0	0
Il13ra1	6243	7390	5429	5025	6544	6790	6599	5782
Il13ra2	0	6	0	0	0	5	0	0
Il15	495	546	493	499	440	499	500	428
Il15ra	537	652	514	636	577	637	564	470
Il16	166	157	157	183	179	170	167	143
Il17a	1	0	1	1	9	2	1	1
Il17b	2	1	2	1	1	4	2	2
Il17c	6	3	0	3	13	19	10	22
Il17d	52	39	36	18	44	43	26	42
Il17f	4	1	1	0	1	5	1	2
Il17ra	695	620	636	777	706	603	655	659
Il17rb	42	70	37	60	54	63	75	61
Il17rc	6814	6567	6658	6653	5991	6186	5934	5982
Il17rd	219	293	302	321	282	299	346	273
Il17re	19	24	16	26	18	20	24	19
Il18	3753	4385	4038	3613	4266	4495	4828	4591
Il18bp	1457	1564	1344	1318	989	1069	1069	1060
Il18r1	57	43	46	52	48	22	39	58
Il18rap	43	24	23	64	23	47	27	12
Il19	0	0	0	0	0	1	1	0
Il1a	61	34	38	29	29	36	8	23
Il1b	78	90	83	81	57	58	65	60
Il1f8	0	1	0	0	0	0	1	0
Il1f9	2	0	0	1	6	0	0	1
Il1r1	239	294	257	283	321	338	281	241
Il1r2	66	31	41	30	44	46	59	67
Il1rap	136	210	226	212	211	281	167	201

Transcriptome sequencing yielded total genetic results for the MOD and APS groups, with a total of 15,936 variables

Continued from above

Il1rapl1	0	0	0	0	4	0	0	0
Il1rl1	283	364	321	272	376	447	362	287
Il1rl2	85	100	111	84	78	130	97	65
Il1rn	106	187	119	150	116	63	87	91
Il2	0	1	0	1	0	0	0	0
Il20rb	58	54	81	51	41	73	53	66
Il21	2	7	4	3	1	0	0	2
Il21r	56	48	41	64	46	40	48	36
Il22	19	5	20	16	13	7	10	12
Il22ra1	5320	6045	5743	5553	6351	6451	5946	5304
Il22ra2	3	21	15	10	22	11	26	15
Il23a	1	0	0	0	1	1	0	4
Il23r	7	6	1	4	4	2	5	2
Il25	0	1	0	0	0	0	0	0
Il27	11	8	6	2	17	14	3	7
Il27ra	41	40	33	40	44	44	41	34
Il2ra	28	22	25	41	17	22	26	24
Il2rb	219	251	227	248	200	166	135	140
Il2rg	312	373	323	352	253	269	209	242
Il31ra	1	1	0	1	1	1	0	0
Il33	1552	1812	1429	1509	1291	1232	1145	1290
Il34	283	302	249	345	251	241	267	247
Il3ra	199	219	238	212	199	185	204	227
Il4	0	4	0	0	1	0	0	0
Il4i1	0	0	2	0	1	0	1	0
Il4i1b	2	0	0	0	4	0	0	0
Il4ra	4787	4923	4762	4680	4473	4516	4576	4216
Il5ra	0	1	4	0	1	2	2	1
Il6ra	125	86	119	116	93	114	76	62
Il6st	1570	1810	1584	1616	1919	1722	1708	1645
Il7	166	149	146	205	159	148	160	129
Il7r	33	72	52	44	48	49	66	45
Il9r	3	5	6	4	1	5	1	13
Ildr1	100	88	63	81	81	46	62	59
Ildr2	3	4	15	7	18	18	25	22
Ilf2	867	929	926	855	1053	996	861	908
Ilf3	889	1017	1044	970	958	1048	881	953
Ilk	2092	1942	2044	1984	2007	1989	1944	1975
Ilkap	863	854	861	776	971	873	809	800
Ilrun	7134	7733	7175	6576	7567	7922	7039	6900
Ilvbl	4283	4560	4016	4049	4339	4315	4277	4190
Immp1l	680	677	663	667	714	796	732	737
Immp2l	333	322	257	333	277	319	288	284
Immt	11780	12215	11676	12393	12142	12344	11542	11393
Imp3	816	889	886	846	942	950	796	831
Imp4	1217	1217	1246	1231	1351	1289	1184	1192
Impa1	4865	5125	5134	5236	5641	5800	5563	5085
Impa2	412	534	506	450	499	511	424	443
Impact	285	312	321	331	281	356	353	260
Impdh1	222	275	263	289	318	270	304	311
Impdh2	1822	2042	1828	1809	2009	1874	1597	1618
Impg1	129	117	161	113	132	161	139	88
Impg2	28	31	47	13	14	14	54	36
Ina	28	15	16	25	9	17	21	6
Inafm2	595	644	616	577	627	591	535	527
Inava	1854	1968	2136	1851	1877	1801	1588	1880
Inca1	17	4	4	9	17	16	16	13
Incenp	1059	1161	1239	1274	1386	1269	1122	1124

Transcriptome sequencing yielded total genetic results for the MOD and APS groups, with a total of 15,936 variables

Continued from above

Inf2	1644	1640	1551	1649	1426	1488	1428	1525
Ing1	858	869	898	912	954	1013	1121	990
Ing2	355	449	375	345	417	409	375	327
Ing3	240	290	242	289	287	268	284	268
Ing4	1501	1476	1448	1465	1420	1429	1486	1327
Ing5	692	624	673	691	638	677	618	552
Inha	14	4	15	10	10	18	15	7
Inhba	72	69	87	96	88	90	99	71
Inhbb	110	147	181	149	113	75	88	74
Inhbc	0	1	0	0	1	0	0	4
Inip	569	593	610	542	561	572	505	498
Inka1	8	13	19	11	13	5	15	7
Inka2	56	59	71	53	70	71	55	65
Inmt	8	2	5	10	11	8	17	13
Ino80	1382	1382	1440	1364	1286	1312	1270	1209
Ino80b	632	632	578	570	540	570	576	598
Ino80c	1767	2003	1936	1877	1883	1911	1817	1808
Ino80d	1308	1486	1634	1604	1519	1496	1536	1510
Ino80e	678	682	733	592	599	643	565	622
Inpp1	965	1064	909	773	798	892	755	735
Inpp4a	816	1063	992	965	923	1103	923	1024
Inpp4b	44	79	84	69	117	82	83	42
Inpp5a	3433	3277	3402	3800	3017	2986	3183	2932
Inpp5b	3087	3455	3402	3448	3172	3682	3577	3177
Inpp5d	231	271	270	235	295	266	276	152
Inpp5e	591	562	488	593	587	559	541	567
Inpp5f	237	193	231	235	135	138	175	205
Inpp5j	660	718	777	780	718	685	696	664
Inpp5k	766	751	771	767	618	687	765	599
Inppl1	4346	4538	4140	4321	4155	4222	4085	4127
Ins1	0	6	0	0	8	1	0	21
Ins2	0	1	0	0	37	41	29	54
Insc	107	115	137	91	87	94	59	89
Insig1	4059	4206	4373	4657	4688	5367	4865	4282
Insig2	1698	1799	1586	1376	1648	1742	1753	1828
Insl3	7	11	6	0	8	1	0	11
Insl5	0	0	0	0	1	0	0	0
Insl6	1085	1013	939	1088	897	1063	986	895
Insm1	183	155	152	151	143	129	137	120
Insr	1906	2281	2253	2222	2033	2288	2306	2097
Insrr	28	62	41	48	39	32	26	21
Insyn1	22	17	17	38	11	34	19	30
Insyn2b	2	2	1	0	0	4	1	6
Ints1	2802	3018	2789	2715	2827	3025	2680	2628
Ints10	969	1055	997	1072	1096	1090	1006	1041
Ints11	1229	1246	1201	1175	1195	1335	1149	1266
Ints12	861	1028	980	874	1035	1034	924	901
Ints13	895	866	943	890	991	995	959	906
Ints14	953	1040	1049	997	1037	1067	997	1080
Ints2	640	726	667	782	795	885	752	798
Ints3	2212	2451	2285	2077	2597	2624	2355	2327
Ints4	1265	1366	1189	1127	1478	1438	1211	1271
Ints5	1163	1190	1197	1163	1366	1154	1166	1213
Ints6	574	719	783	688	766	733	758	680
Ints6l	127	102	142	131	95	152	124	125
Ints7	917	957	1036	942	906	896	881	733
Ints8	804	885	977	913	1284	1176	1037	997
Ints9	650	641	673	647	743	674	647	568

Transcriptome sequencing yielded total genetic results for the MOD and APS groups, with a total of 15,936 variables

Continued from above

Intu	148	157	171	162	166	127	110	171
Invs	256	277	252	285	290	288	346	265
Ip6k1	5202	5776	5404	5454	5332	5165	5516	5086
Ip6k2	1133	1247	1370	1247	1122	1194	1147	1316
Ip6k3	0	1	2	0	1	1	1	0
Ipcef1	22	49	45	41	42	49	35	29
Ipmk	8084	8949	8574	8095	8925	9625	9884	8781
Ipo11	793	774	738	741	748	758	693	694
Ipo13	862	945	772	852	774	901	847	815
Ipo4	962	933	946	862	931	940	895	838
Ipo5	3283	3681	3403	3227	3683	3442	3121	3200
Ipo7	3643	4236	3783	3741	4730	4798	4595	4271
Ipo8	2585	3041	2760	2581	3056	3111	2742	2683
Ipo9	1032	1078	1014	908	937	957	993	864
Ipp	352	303	352	334	259	372	340	288
Ippk	609	598	678	567	756	743	655	609
Iqank1	15	27	17	24	19	15	27	29
Iqcb1	30	44	47	40	53	51	34	48
Iqcc	153	143	140	202	218	216	190	182
Iqcd	7	20	12	8	19	8	10	18
Iqce	84	118	127	122	133	117	97	80
Iqcf4	0	0	0	0	1	0	0	0
Iqcg	66	40	72	57	36	32	50	38
Iqch	1	0	0	0	0	1	0	0
Iqck	14	8	3	10	8	3	8	4
Iqcn	0	1	1	2	3	1	1	8
Iqgap1	9804	10486	10157	9872	9698	10047	9592	8958
Iqgap2	17987	18803	17027	17687	17655	19204	20055	17514
Iqgap3	644	680	677	693	620	629	545	599
Iqsec1	248	323	347	332	336	305	308	268
Iqsec2	767	747	755	753	801	667	619	636
Iqsec3	22	6	13	16	10	5	19	12
Iqub	0	1	0	8	1	0	0	0
Irak1	1670	1685	1686	1678	1675	1562	1565	1536
Irak1bp1	35	33	27	18	27	23	19	19
Irak2	4203	4548	3782	3603	3685	3986	4036	3855
Irak3	54	55	74	53	52	60	67	71
Irak4	1918	1943	1776	1684	2044	1988	2009	1951
Ireb2	3776	4117	4029	3815	4410	4478	4662	3969
Irf1	12777	12490	10943	11504	10445	11462	9702	9393
Irf2	2734	2695	2622	2828	2158	2531	2240	2372
Irf2bp1	972	923	944	874	1100	1089	909	875
Irf2bp2	2556	2691	2875	2782	3115	2834	2497	2762
Irf2bpl	806	817	807	789	794	752	657	765
Irf3	2183	2225	2200	2217	2596	2570	2290	2336
Irf4	223	193	237	256	173	193	171	184
Irf5	249	223	249	390	248	250	194	227
Irf6	8184	8915	8311	8141	8582	8740	8931	7969
Irf7	12412	16142	15227	11526	15737	15003	13644	13525
Irf8	7957	7787	6913	6901	6246	6595	6086	5376
Irf9	1824	2108	1956	2003	2124	2092	1969	1865
Irgm1	6369	7611	6492	5828	4996	5278	4509	4077
Irgm2	5257	6155	5526	5001	4165	4357	3935	3635
Irgq	1359	1249	1359	1465	1411	1262	1309	1228
Irs1	32	48	32	48	53	64	45	44
Irs2	1908	2020	2083	2019	1339	1246	1524	1482
Irs3	0	0	0	2	10	0	1	0
Irs4	6	2	1	8	5	8	4	1

Transcriptome sequencing yielded total genetic results for the MOD and APS groups, with a total of 15,936 variables

Continued from above

Irx2	0	0	0	0	0	5	0	0
Irx3	11	0	5	1	6	6	6	7
Irx4	0	0	1	5	0	2	2	0
Irx5	8	4	4	7	0	0	0	1
Isca1	3173	3356	3254	3322	3385	3174	3257	3204
Isca2	866	960	949	909	872	870	869	866
Iscu	1761	1620	1463	1565	1483	1481	1499	1451
Isg15	5888	8113	7414	5014	6408	6619	6009	5948
Isg20	127	137	126	99	111	130	111	118
Isg20l2	923	868	880	822	881	989	754	865
Isl1	14	39	58	52	46	44	32	67
Isl2	0	9	6	10	3	4	6	5
Islr	187	156	209	223	213	239	188	187
Islr2	19	14	19	40	35	21	22	18
Ism1	5	3	3	17	8	14	9	13
Ism2	2	16	6	11	15	11	2	9
Isoc1	3320	3665	3457	3181	3580	3878	3557	3378
Isoc2a	1032	1025	961	1005	987	1129	1063	1053
Isoc2b	316	344	308	305	399	336	297	355
Ist1	5009	4999	4566	4709	4698	4472	4547	4517
Isx	4391	4623	4620	4312	5948	5452	4839	5223
Isy1	779	796	780	772	912	798	720	739
Isyna1	1797	1881	1856	1660	1910	1805	1773	1957
Itch	4216	4835	4348	4436	4936	5178	4983	4258
Itfg1	3785	3835	3644	3694	4142	4237	4525	3953
Itfg2	355	342	344	336	286	285	322	333
Itga1	454	548	560	438	506	445	490	487
Itga10	20	27	38	52	39	42	29	41
Itga11	105	143	133	112	97	100	71	100
Itga2	1047	1170	1111	1089	1125	1039	1051	982
Itga2b	57	39	10	42	26	25	40	29
Itga3	8733	9678	9316	8798	9582	9545	9287	8821
Itga4	267	313	280	308	252	249	241	204
Itga5	595	598	720	746	649	666	670	666
Itga6	8829	10434	9163	8405	10249	10554	10785	9932
Itga7	89	129	102	77	89	88	124	118
Itga8	139	194	191	141	126	193	141	147
Itga9	559	646	728	679	592	641	589	505
Itgad	11	8	8	19	5	5	4	5
Itgae	176	176	188	134	108	236	145	96
Itgal	271	284	264	320	223	297	284	197
Itgam	658	853	702	711	636	614	622	671
Itgav	1173	1296	1071	1186	1280	1264	1115	1273
Itgax	527	648	514	485	459	465	423	399
Itgb1	9848	11291	10014	9696	11650	11525	11052	10099
Itgb1bp1	359	301	328	371	372	323	297	255
Itgb1bp2	9	21	19	19	7	18	35	24
Itgb2	457	416	450	550	466	423	442	361
Itgb2l	8	3	5	8	5	3	3	6
Itgb3	341	384	417	390	315	331	386	294
Itgb3bp	90	124	111	144	135	120	77	126
Itgb4	5588	6024	5530	5852	5733	5631	5640	5465
Itgb5	1926	2076	1932	1661	2322	2377	2230	1910
Itgb6	305	383	313	279	307	298	343	310
Itgb7	211	179	209	142	160	165	167	146
Itgb8	2	8	16	10	6	8	7	12
Itgbl1	0	2	0	0	0	0	0	0
Itih1	2	1	0	0	3	2	4	4

Transcriptome sequencing yielded total genetic results for the MOD and APS groups, with a total of 15,936 variables

Continued from above

Itih2	8	5	4	7	9	2	3	6
Itih3	29	34	14	34	14	43	18	21
Itih4	21	28	14	21	78	74	78	60
Itih5	520	575	537	607	603	637	540	543
Itih5l-ps	0	0	0	0	0	1	0	0
Itk	40	55	43	52	51	38	43	55
Itln1	14476	13688	13560	16090	11266	12081	14579	14546
Itm2a	36	44	35	38	23	21	22	48
Itm2b	32353	35093	31447	29508	31199	33137	32161	29841
Itm2c	2504	2703	2474	2340	2524	2512	2258	2291
Itpa	1631	1580	1430	1547	1595	1626	1428	1401
Itpk1	13061	13816	12345	11775	11970	12594	12931	12395
Itpka	1265	1449	1418	1250	1054	996	1004	909
Itpkb	422	547	408	358	417	380	379	360
Itpkc	2074	2005	1968	1956	1818	2088	1900	1904
Itpr1	839	1027	804	691	1040	1046	1033	961
Itpr2	508	557	532	511	536	578	648	491
Itpr3	9283	9367	9361	10155	8347	8135	8040	7442
Itprid1	2	2	1	8	2	0	4	1
Itprid2	4249	4794	4293	4210	4065	4362	4779	4594
Itprip	63	115	112	142	59	126	85	92
Itpripl1	256	308	388	346	296	299	273	232
Itpripl2	1594	1813	1875	1828	1415	1398	1553	1538
Itsn1	986	1114	1041	1056	1050	1121	936	988
Itsn2	1799	2038	1872	1933	2268	2178	2130	1879
Ivd	1099	1166	1269	1077	1168	1102	1150	1133
Ivns1abp	10440	11893	11929	10991	12540	12813	12230	11288
Iws1	1705	1851	1904	1990	1931	1842	1879	1787
Iyd	619	513	415	583	395	422	451	382
Izumo1	2	1	6	1	1	7	1	2
Izumo1r	6	1	10	14	4	4	2	3
Izumo4	81	108	147	128	147	116	99	115
Jade1	1179	1207	1146	1041	1250	1423	1321	1133
Jade2	651	872	837	694	773	739	616	718
Jade3	338	350	297	343	304	313	356	322
Jag1	376	461	486	423	431	464	387	363
Jag2	116	137	122	151	179	123	141	117
Jagn1	1009	916	904	807	973	960	772	762
Jak1	5475	6196	5905	5492	6216	6274	5649	5552
Jak2	5748	5981	5745	5680	5391	5964	6276	5481
Jak3	742	776	728	729	811	728	628	750
Jakmip1	964	1124	1199	1073	1142	1254	1029	932
Jakmip2	1	0	5	1	0	7	6	4
Jakmip3	3	2	3	2	2	1	1	4
Jam2	196	205	207	214	172	147	179	168
Jam3	124	112	118	118	116	67	63	130
Jaml	1044	1139	1014	977	1093	1249	1057	988
Jarid2	1449	1643	1386	1564	1572	1609	1431	1530
Jazf1	37	31	29	32	41	20	23	21
Jcad	126	117	195	140	131	159	151	108
Jchain	26833	26194	24618	26698	26419	27733	25525	25527
Jdp2	219	185	220	216	164	147	172	150
Jhy	0	0	0	2	0	0	0	0
Jkamp	534	615	544	568	686	618	656	620
Jmjd1c	2181	2543	2281	2022	2667	2702	2737	2217
Jmjd4	458	583	532	465	474	579	499	417
Jmjd6	1031	1071	1143	1007	1181	1224	1122	1038
Jmjd7	176	166	163	198	156	156	144	150

Transcriptome sequencing yielded total genetic results for the MOD and APS groups, with a total of 15,936 variables

Continued from above

Jmjd8	1253	1232	1132	1043	1147	1133	1098	1159
Jmy	606	652	665	648	630	697	707	636
Josd1	1316	1334	1399	1324	1484	1462	1335	1390
Josd2	595	651	613	706	670	638	605	619
Jph1	12	9	5	8	11	13	14	11
Jph2	359	415	389	366	406	388	358	357
Jph3	7	12	11	8	6	3	6	10
Jph4	6	25	20	25	25	3	46	5
Jpt1	9553	9623	9337	8854	9197	9555	8859	8737
Jpt2	3069	3572	3324	3388	3425	3152	3040	3064
Jrk	91	106	65	100	116	75	144	74
Jrkl	384	400	376	397	419	455	471	459
Jsrp1	0	1	0	0	0	1	0	1
Jtb	1342	1425	1332	1301	1419	1510	1386	1330
Jun	3157	3251	3336	3356	3700	3993	3678	3253
Junb	5897	6225	6087	5657	5572	5895	5460	5529
Jund	13976	12859	13852	13793	13218	13513	13318	13589
Jup	10606	11295	10102	10151	10667	10701	10452	9915
Kalrn	2376	2582	2260	2258	2262	2570	2837	2440
Kank1	425	400	551	496	458	448	448	479
Kank2	537	525	598	539	526	458	516	441
Kank3	120	110	143	116	120	93	106	103
Kank4	9	3	12	13	9	15	4	15
Kansl1	1501	1623	1663	1610	1670	1756	1435	1654
Kansl1l	236	254	205	261	204	213	282	190
Kansl2	1062	1192	1170	1196	1296	1168	1114	998
Kansl3	3171	3289	3367	3266	3065	3176	3014	2718
Kars	5071	5496	5025	4780	4747	4747	4252	4368
Kash5	1	1	0	0	2	1	0	0
Kat14	1308	1345	1340	1289	1387	1406	1238	1251
Kat2a	694	945	881	807	911	971	791	711
Kat2b	1999	2300	2089	1852	1872	1732	1856	1710
Kat5	1317	1237	1231	1302	1147	1138	1057	1132
Kat6a	2613	2945	2681	2764	2983	2968	2941	2608
Kat6b	788	796	851	827	811	757	738	680
Kat7	2341	2517	2261	2498	2262	2444	2429	2240
Kat8	417	489	495	456	563	543	452	450
Katna1	1147	1133	1176	1086	1175	1215	1174	1017
Katnal1	25	28	34	26	27	39	43	41
Katnal2	1	1	0	1	1	0	2	0
Katnb1	599	667	651	668	653	631	596	627
Katnbl1	529	598	618	650	567	602	479	662
Katnip	1435	1649	1547	1715	1540	1592	1523	1317
Kazald1	19	11	24	25	21	16	21	16
Kazn	675	684	607	688	568	603	563	587
Kbtbd11	3499	3582	3509	3444	4134	4210	4243	3690
Kbtbd12	4	12	0	6	1	1	0	1
Kbtbd13	5	5	7	7	15	15	19	3
Kbtbd2	947	1194	987	901	911	910	863	904
Kbtbd3	29	50	59	50	62	46	52	72
Kbtbd4	475	602	586	498	631	644	568	497
Kbtbd6	24	13	11	31	22	22	22	12
Kbtbd7	259	236	278	256	243	291	292	269
Kbtbd8	247	242	249	200	286	305	319	254
Kcmf1	3088	3245	3072	3073	3143	3160	3044	3091
Kcna1	15	38	13	8	21	14	25	15
Kcna10	0	0	0	1	0	0	0	0
Kcna2	27	30	60	31	33	27	58	25

Transcriptome sequencing yielded total genetic results for the MOD and APS groups, with a total of 15,936 variables

Continued from above

Kcna3	16	13	10	19	9	8	13	26
Kcna4	0	0	4	0	5	0	0	1
Kcna5	6	4	10	7	8	18	1	6
Kcna6	11	12	18	9	28	5	5	11
Kcna7	1	5	0	3	0	4	0	1
Kcnab1	7	3	15	5	19	3	6	12
Kcnab2	130	106	129	126	105	100	97	108
Kcnab3	14	0	1	12	5	1	11	1
Kcnb1	63	53	143	121	70	82	68	68
Kcnb2	24	18	18	35	6	29	37	30
Kcnc1	19	14	41	19	10	43	12	32
Kcnc2	1	0	5	5	1	0	1	0
Kcnc3	49	31	56	57	55	100	65	47
Kcnc4	1	12	4	3	4	6	2	1
Kcnd1	30	16	11	11	15	26	17	9
Kcnd2	1	1	5	0	1	1	1	0
Kcnd3	26	17	13	20	24	29	30	57
Kcne1l	0	1	4	1	4	1	4	0
Kcne3	1838	1723	1879	2195	2153	1984	1889	1760
Kcne4	20	20	46	50	16	18	35	11
Kcnf1	155	201	176	147	149	160	114	108
Kcng1	8	5	10	6	9	11	7	9
Kcng2	17	6	11	21	14	7	15	3
Kcng3	10	23	24	30	20	18	31	17
Kcng4	6	0	2	4	9	14	13	9
Kcnh1	26	10	5	15	14	5	16	5
Kcnh2	98	139	139	175	114	104	126	93
Kcnh3	30	37	27	36	39	33	43	36
Kcnh4	2	0	0	1	0	0	0	0
Kcnh6	41	65	68	47	51	58	49	51
Kcnh7	0	1	1	1	4	0	0	9
Kcnh8	3	2	0	7	2	0	4	0
Kcnip1	8	2	6	10	6	1	15	1
Kcnip2	76	89	121	70	107	101	95	104
Kcnip3	41	29	44	43	53	40	61	23
Kcnip4	0	1	0	0	1	2	0	0
Kcnj10	24	39	24	33	42	39	25	32
Kcnj11	6	21	10	27	15	7	14	8
Kcnj12	6	10	8	16	4	8	11	13
Kcnj13	46	46	47	67	6	10	28	19
Kcnj14	7	0	4	1	5	3	0	5
Kcnj15	8	2	1	1	6	14	2	6
Kcnj16	22	8	8	20	8	22	6	19
Kcnj2	428	409	443	512	309	345	291	224
Kcnj3	3	29	19	8	8	6	10	12
Kcnj4	0	0	0	0	1	0	0	1
Kcnj5	18	7	5	5	6	1	32	15
Kcnj6	2	0	1	0	1	1	0	1
Kcnj8	134	170	156	193	249	196	201	222
Kcnj9	1	3	7	5	1	6	2	4
Kcnk1	1511	1547	1682	1611	1853	1609	1562	1647
Kcnk10	474	641	563	543	578	576	589	475
Kcnk13	14	13	18	29	17	5	13	34
Kcnk16	31	39	30	35	32	27	22	36
Kcnk2	28	40	17	20	19	16	5	31
Kcnk3	70	70	90	61	52	97	87	72
Kcnk4	0	0	1	0	0	0	0	0
Kcnk5	3184	3542	4106	3792	3381	3601	4054	3672

Transcriptome sequencing yielded total genetic results for the MOD and APS groups, with a total of 15,936 variables

Continued from above

Kcnk6	942	838	1051	1047	1115	1036	792	1055
Kcnk7	25	8	11	11	11	12	14	5
Kcnk9	0	0	0	0	0	0	0	4
Kcnma1	124	72	101	84	126	79	80	72
Kcnmb1	94	120	117	131	103	109	117	81
Kcnmb2	0	1	2	1	4	0	0	1
Kcnmb3	0	4	10	8	2	6	3	3
Kcnmb4	6	9	12	5	2	14	10	3
Kcnn1	6	5	0	0	3	2	5	14
Kcnn2	1	0	1	1	14	1	1	1
Kcnn3	71	94	78	133	109	118	84	75
Kcnn4	1048	1263	1207	1137	1369	1039	1072	1050
Kcnq1	2191	2029	2223	2547	1761	1781	1733	1764
Kcnq2	18	5	7	13	13	31	22	22
Kcnq3	4	9	3	10	7	10	20	3
Kcnq4	67	45	45	56	62	54	50	22
Kcnq5	22	10	3	5	19	35	20	10
Kcnrg	0	0	0	1	0	1	0	0
Kcns2	0	0	0	0	0	0	1	1
Kcns3	26	15	35	30	43	30	28	38
Kcnt1	23	14	8	6	3	16	12	14
Kcnt2	10	10	14	7	10	6	20	12
Kcnu1	1	6	2	4	3	2	7	5
Kcnv1	2	1	0	4	1	0	0	0
Kcnv2	0	0	1	0	4	0	1	5
Kcp	29	21	27	13	24	27	10	29
Kctd1	25	29	21	42	21	20	45	21
Kctd10	3848	4117	4192	3924	4060	3993	3804	3932
Kctd11	144	185	186	215	207	212	180	203
Kctd12	1054	1038	970	1066	996	1017	1006	860
Kctd12b	126	110	72	102	102	82	119	82
Kctd13	213	203	246	193	163	277	213	214
Kctd14	365	283	341	417	435	479	493	447
Kctd15	64	80	48	49	68	55	72	57
Kctd17	159	194	171	177	169	191	187	132
Kctd18	136	149	127	90	113	140	109	93
Kctd19	0	0	1	1	1	0	2	0
Kctd2	960	900	810	859	827	735	781	766
Kctd20	1360	1583	1405	1279	1474	1522	1555	1432
Kctd21	491	405	436	348	505	463	470	515
Kctd3	1308	1392	1256	1263	1154	1277	1093	1146
Kctd5	5691	5688	5276	5061	5560	5872	5549	5592
Kctd6	366	413	378	398	337	352	349	368
Kctd7	34	52	40	40	62	49	47	51
Kctd8	4	1	1	5	0	1	5	1
Kctd9	914	1033	916	998	957	951	1061	1003
Kdelr1	7773	8078	7689	7892	7571	7984	7553	7590
Kdelr2	9037	9649	9055	8644	9459	9483	9218	8840
Kdelr3	854	751	812	789	982	827	1272	1162
Kdf1	1295	1426	1431	1404	1305	1416	1235	1107
Kdm1a	1273	1295	1307	1375	1470	1207	1260	1289
Kdm1b	689	803	725	821	889	908	849	782
Kdm2a	4637	5286	4763	4667	5191	5335	4847	4879
Kdm2b	507	554	572	543	539	559	510	505
Kdm3a	1476	1453	1737	1768	1781	1897	1644	1548
Kdm3b	2216	2413	2365	2383	2243	2423	2342	2256
Kdm4a	1612	1588	1567	1494	1651	1621	1573	1420
Kdm4b	1018	1007	1008	1091	1095	1167	1022	1032

Transcriptome sequencing yielded total genetic results for the MOD and APS groups, with a total of 15,936 variables

Continued from above

Kdm4c	649	716	735	669	788	802	845	719
Kdm4d	0	1	0	1	0	0	0	0
Kdm5a	1958	2168	1858	1861	2094	2081	1965	1744
Kdm5b	1635	1860	1994	1886	2057	2047	1938	1953
Kdm5c	5581	7322	7825	6815	7898	8026	6875	5626
Kdm5d	458	390	0	252	205	183	495	592
Kdm6a	1213	1757	2004	1585	1785	1884	1665	1299
Kdm6b	3351	3338	3162	3395	3388	3525	3417	3112
Kdm7a	2518	2612	2649	2566	2287	2582	2759	2397
Kdm8	116	153	211	135	220	198	161	212
Kdr	923	1150	999	912	862	929	875	778
Kdsr	813	929	857	884	873	809	816	830
Keap1	2955	3200	3379	2825	3110	3408	3045	2882
Keg1	0	0	0	0	2	0	0	2
Kel	0	0	0	0	0	0	1	1
Khdc1a	18	9	33	28	11	12	15	38
Khdc3	2	5	2	4	7	1	11	1
Khdc4	1384	1440	1618	1499	1778	1689	1534	1483
Khdrbs1	3987	4349	4255	4117	3828	3882	3802	3648
Khdrbs2	0	2	6	1	1	0	0	0
Khdrbs3	58	72	77	69	73	96	56	69
Khk	11162	11664	11123	10171	11188	12387	12378	11654
Khnyn	2990	3120	3241	3245	2960	3069	3155	2867
Khsrp	2745	2751	2922	2926	3139	2828	2449	2761
Kidins220	3215	3460	3344	3584	3317	3431	3484	3147
Kif11	1230	1636	1323	1366	1512	1649	1492	1414
Kif12	30	49	62	28	31	35	45	51
Kif13a	2790	3209	2956	3117	3170	3053	2934	2838
Kif13b	3352	3650	3490	3128	3361	3314	3287	2926
Kif14	267	318	292	244	203	212	254	250
Kif15	527	698	514	526	587	605	567	521
Kif16b	1415	1368	1243	1393	1171	1286	1320	1258
Kif17	4	2	0	0	0	0	1	0
Kif18a	127	139	193	138	224	168	218	190
Kif18b	230	316	312	207	316	333	240	249
Kif19a	36	30	23	14	22	33	28	6
Kif19b	0	0	0	0	0	1	0	0
Kif1a	65	94	67	80	81	66	72	82
Kif1b	3678	4171	3774	4128	4148	4225	4349	3807
Kif1c	11169	11307	11106	10912	10491	11017	10565	9955
Kif20a	748	884	938	758	955	851	704	837
Kif20b	380	539	451	411	527	504	481	457
Kif21a	524	488	573	526	592	631	573	472
Kif21b	2741	2932	2954	2745	2964	3392	3679	3413
Kif22	680	887	707	733	806	677	598	726
Kif23	867	864	940	877	1109	1132	886	994
Kif24	263	279	206	238	246	199	245	164
Kif26a	30	31	71	50	34	51	40	32
Kif26b	19	16	24	5	14	20	8	7
Kif27	34	23	31	63	42	60	78	78
Kif28	0	0	0	0	1	0	0	0
Kif2a	1582	1674	1518	1473	1696	1525	1728	1469
Kif2c	367	382	402	338	439	420	334	337
Kif3a	142	113	166	149	161	176	197	189
Kif3b	1647	1826	1727	1828	1583	1639	1615	1650
Kif3c	59	75	67	70	78	75	63	46
Kif4	684	728	665	731	645	615	521	628
Kif5a	41	66	60	68	85	57	95	82

Transcriptome sequencing yielded total genetic results for the MOD and APS groups, with a total of 15,936 variables

Continued from above

Kif5b	7630	8148	7503	7698	8176	8915	8700	7860
Kif5c	27	55	46	56	62	25	40	51
Kif6	2	5	5	0	9	5	0	3
Kif7	12	13	9	16	16	6	17	14
Kif9	35	50	47	32	52	39	28	34
Kifap3	218	236	237	219	279	261	283	202
Kifbp	709	610	644	588	741	770	667	635
Kifc1	444	469	449	482	527	552	486	503
Kifc2	296	241	309	280	242	282	247	306
Kifc3	1282	1389	1199	1338	1173	1303	1314	1213
Kifc5b	151	150	185	131	185	176	110	144
Kin	365	382	371	347	386	405	297	399
Kirrel	194	219	236	232	273	198	173	175
Kirrel2	1	5	1	1	5	0	5	1
Kirrel3	0	1	2	6	1	2	0	0
Kiss1	71	68	106	88	58	56	58	86
Kiss1r	23	28	12	14	18	14	14	23
Kit	258	260	329	276	260	200	245	241
Kitl	2990	2747	2840	3276	2797	3039	2820	2224
Kiz	137	163	185	142	182	216	171	167
Kl	4	0	12	2	1	14	2	5
Klb	8	4	16	18	5	6	15	1
Klc1	1371	1455	1450	1524	1452	1366	1279	1246
Klc2	335	347	375	336	361	327	323	301
Klc3	33	27	17	30	34	25	39	28
Klc4	9138	9823	9124	8938	9977	10367	10096	9512
Klf1	11	15	8	7	18	4	13	3
Klf10	1166	1149	1155	1215	1213	1174	1127	1179
Klf11	180	244	223	190	222	182	181	208
Klf12	17	19	38	16	18	23	38	12
Klf13	2307	2457	2567	2650	1868	2123	2074	1872
Klf14	0	1	0	0	0	0	0	1
Klf15	112	124	171	122	101	141	180	195
Klf16	663	602	633	565	575	729	697	619
Klf17	0	0	0	0	4	1	0	1
Klf2	200	235	258	246	195	260	156	203
Klf3	7228	7755	7094	7178	6762	7282	7336	6854
Klf4	11688	10842	10412	11583	10019	10265	10812	9610
Klf5	6677	6612	6763	6681	6015	5821	5672	5731
Klf6	5880	6253	6527	6423	5397	5684	6031	5810
Klf7	499	573	522	506	484	503	464	472
Klf8	3	3	13	2	8	4	5	10
Klf9	1232	1231	1417	1445	1126	1200	1360	1136
Klhdc1	21	11	24	26	30	43	28	28
Klhdc10	2025	2185	2005	2099	2063	1990	1962	1917
Klhdc2	714	721	746	758	812	753	722	759
Klhdc3	4453	4321	3948	4126	4011	4276	4285	4367
Klhdc4	1043	1076	996	1024	1129	935	965	1055
Klhdc7a	19	28	34	35	41	34	38	27
Klhdc7b	74	73	48	49	57	61	43	66
Klhdc8a	30	18	24	29	30	35	21	34
Klhdc8b	81	85	118	86	88	89	82	96
Klhdc9	4	2	6	5	5	2	2	7
Klhl1	0	1	2	6	1	1	1	4
Klhl10	0	1	0	2	0	0	0	4
Klhl11	127	147	135	147	148	132	135	116
Klhl12	452	503	507	485	591	517	487	454
Klhl13	47	54	34	38	44	52	45	45

Transcriptome sequencing yielded total genetic results for the MOD and APS groups, with a total of 15,936 variables

Continued from above

Klhl15	111	127	89	117	85	108	99	117
Klhl17	248	190	252	213	204	171	186	154
Klhl18	1131	1045	1198	1103	1219	1250	1163	1144
Klhl2	1174	1257	1217	1348	1273	1233	1153	1283
Klhl20	378	431	466	386	412	394	316	317
Klhl21	506	582	566	490	618	501	483	499
Klhl22	1808	1883	1638	1716	1885	1873	1747	1579
Klhl23	338	447	343	390	340	420	410	322
Klhl24	1073	1234	1377	1241	1118	1090	1334	1242
Klhl25	458	501	491	464	490	571	520	484
Klhl26	530	574	527	445	516	460	453	522
Klhl28	392	363	369	353	473	468	374	392
Klhl29	34	54	35	39	29	24	42	77
Klhl3	4	8	10	9	31	5	11	3
Klhl30	3	11	44	13	15	13	20	12
Klhl31	1	1	0	0	0	0	0	10
Klhl32	3	5	9	16	15	7	3	12
Klhl33	21	23	23	16	19	17	22	10
Klhl34	0	1	0	0	0	0	4	0
Klhl35	0	7	4	0	1	0	1	1
Klhl36	236	134	216	220	209	180	176	167
Klhl38	2	0	1	9	0	1	1	1
Klhl4	8	17	13	15	5	5	17	7
Klhl41	0	8	0	0	0	3	0	1
Klhl42	321	356	409	343	389	350	290	337
Klhl5	596	589	647	541	521	537	510	528
Klhl6	77	38	45	59	38	56	52	45
Klhl7	215	254	300	315	279	253	292	280
Klhl8	52	42	49	54	72	49	35	39
Klhl9	3021	3430	3347	3281	3454	3581	3436	3307
Klk1	2026	2149	1851	1901	3422	3273	2906	3373
Klk10	0	0	0	0	4	0	1	0
Klk13	0	0	0	0	4	0	0	0
Klk1b1	0	0	0	0	0	0	1	0
Klk1b11	0	0	0	0	0	1	4	8
Klk1b16	0	1	0	0	0	0	0	1
Klk1b21	0	4	0	0	0	0	0	0
Klk1b24	1	0	0	1	1	0	1	1
Klk1b26	0	0	0	0	1	0	1	0
Klk1b27	0	0	0	0	1	0	0	0
Klk1b3	0	0	0	0	5	6	8	2
Klk1b4	2	0	0	5	10	1	2	8
Klk1b5	20	4	5	20	55	73	79	62
Klk1b8	0	0	0	1	0	0	1	0
Klk7	1	0	0	1	0	0	2	0
Klk8	16	15	7	21	26	8	22	21
Klkb1	4	12	9	12	3	9	8	8
Klra1	0	4	4	1	9	0	0	0
Klra10	0	0	0	0	0	0	2	0
Klra17	27	18	30	22	22	19	15	8
Klra2	178	128	125	132	117	115	130	83
Klra3	11	2	1	0	1	0	3	2
Klra4	1	0	0	0	0	0	0	1
Klra5	6	0	1	1	2	2	2	1
Klra6	0	4	1	0	0	1	1	0
Klra7	1	1	7	0	1	7	5	1
Klra8	0	1	1	0	0	0	0	0
Klra9	2	0	0	0	0	0	1	0

Transcriptome sequencing yielded total genetic results for the MOD and APS groups, with a total of 15,936 variables

Continued from above

Klrb1	2	6	2	7	12	11	8	11
Klrb1a	6	2	8	6	0	7	2	8
Klrb1b	23	37	36	51	13	30	27	22
Klrb1c	11	10	7	10	7	2	0	3
Klrb1f	3	6	4	15	0	5	8	1
Klrc1	6	2	2	1	0	2	0	6
Klrc2	0	0	0	0	2	1	0	0
Klrc3	1	0	1	0	0	0	0	0
Klrd1	22	20	17	27	17	11	20	11
Klre1	1	1	3	2	0	0	1	1
Klrg1	2	3	2	6	0	13	1	0
Klrg2	5	15	7	22	5	15	19	4
Klrh1	0	0	4	0	1	1	0	0
Klri1	2	9	7	4	0	2	5	0
Klri2	0	1	1	2	1	1	0	1
Klrk1	7	16	28	15	6	11	9	8
Kmo	40	59	28	73	74	42	58	82
Kmt2a	1518	1907	1848	1941	1676	1820	1551	1458
Kmt2b	1867	2096	2378	2142	2071	2014	1997	1970
Kmt2c	2682	2848	2936	2960	2625	2631	2654	2516
Kmt2d	3219	3551	3774	3584	3672	3812	3313	3183
Kmt2e	1910	2263	2262	2277	2197	2276	2246	2003
Kmt5a	5508	5723	5795	5662	6033	6100	5586	5423
Kmt5b	1789	1948	1948	1943	1872	1905	1930	1620
Kmt5c	1747	1817	1843	1973	1848	1896	1924	1878
Kndc1	2	1	5	9	12	4	5	2
Kng1	13	54	9	12	11	12	13	4
Kng2	6	3	3	2	2	13	5	3
Knl1	354	413	454	365	523	472	411	439
Knop1	973	1036	933	986	1176	962	955	922
Knstrn	590	663	677	595	670	650	545	574
Kntc1	276	332	439	368	356	311	332	361
Kpna1	1825	2070	1898	1883	2243	2253	2146	1971
Kpna2	4646	5419	5190	4898	5803	5757	5450	4823
Kpna3	1386	1255	1281	1241	1480	1456	1360	1294
Kpna4	1919	2202	2026	2067	2465	2650	2485	2153
Kpna6	2891	2904	2859	2819	2805	2965	2783	2683
Kpna7	4	0	1	1	13	4	5	1
Kpnb1	7713	8497	7999	8009	8451	8424	7925	7257
Kptn	447	517	477	542	430	447	442	404
Kras	3628	4019	3525	3864	4003	3899	4021	3625
Krba1	146	116	145	134	102	137	116	118
Krcc1	2703	2696	2784	2627	2960	3089	2992	2742
Kremen1	729	749	863	838	756	655	723	735
Kremen2	162	156	180	201	179	157	122	176
Kri1	483	512	442	427	455	494	409	455
Krit1	614	629	728	675	773	765	667	701
Krr1	548	727	633	617	823	841	818	718
Krt1	0	0	1	1	2	0	4	0
Krt10	49	43	76	61	72	45	57	51
Krt12	19	29	15	15	13	46	31	22
Krt13	0	0	0	0	0	0	0	1
Krt14	1	0	0	1	9	2	0	1
Krt15	20	16	17	11	6	11	19	12
Krt16	4	0	0	0	0	0	0	0
Krt17	1	2	7	10	4	1	7	5
Krt18	5651	5400	5497	5659	5929	5723	5047	5324
Krt19	40286	41529	44618	42792	43179	40531	35154	39880

Transcriptome sequencing yielded total genetic results for the MOD and APS groups, with a total of 15,936 variables

Continued from above

Krt20	60221	67214	55216	52155	62305	68625	69617	62138
Krt222	19	30	25	27	16	15	30	16
Krt23	122	124	129	121	117	125	135	107
Krt27	0	0	0	0	0	1	0	0
Krt31	0	0	0	0	1	0	0	0
Krt32	0	0	0	6	0	0	0	0
Krt35	0	1	0	0	0	0	0	0
Krt36	0	1	0	2	6	0	0	0
Krt39	1	0	0	0	6	0	1	0
Krt5	0	0	0	0	0	0	1	0
Krt7	2283	2528	2843	2719	3044	2746	2456	2611
Krt78	18	25	17	22	14	26	21	29
Krt79	0	0	0	1	0	0	1	0
Krt8	115638	118084	115001	113009	120102	118942	113462	115498
Krt80	15	11	5	16	21	20	23	24
Krt87	6	16	28	14	10	12	14	18
Krt88	0	1	0	0	0	0	1	0
Krtap2-4	0	0	1	0	0	0	0	0
Krtap28-1	0	0	0	0	0	6	0	0
Krtap3-1	0	1	0	0	0	0	0	0
Krtap4-16	0	0	2	1	2	0	0	1
Krtap5-3	1	0	0	1	5	0	0	1
Krtap5-5	2	0	0	0	0	1	0	0
Krtcap2	1638	1724	1771	1659	1810	1710	1579	1844
Krtcap3	1333	1365	1322	1405	1245	1414	1163	1283
Ksr1	4895	5336	4649	4702	4395	4864	4601	4357
Ksr2	0	4	0	0	2	6	1	0
Kti12	681	825	670	648	813	674	689	737
Ktn1	1000	1000	914	826	856	810	873	690
Kxd1	1151	1285	1298	1231	1147	1179	1078	1054
Ky	52	91	85	88	57	69	47	79
Kyat1	830	954	1006	881	1023	1039	1198	1131
Kyat3	160	189	173	181	222	161	156	172
Kynu	66	54	64	55	62	59	46	60
L1cam	1309	1533	1539	1379	1436	1401	1175	1366
L1td1	0	0	0	0	0	4	0	0
L2hgdh	1715	1627	1630	1790	1572	1749	1834	1745
L3hypdh	25	19	31	45	17	24	33	12
L3mbtl1	0	4	0	0	0	1	4	0
L3mbtl2	661	827	721	611	829	737	593	661
L3mbtl3	316	351	288	297	287	302	317	271
L3mbtl4	0	0	0	0	0	1	0	0
LOC10003	0	0	0	0	1	0	0	1
LOC10004	1	0	0	0	4	2	3	1
LOC10004	1	0	3	2	0	1	0	0
LOC10004	0	0	0	0	0	0	0	1
LOC10105	5	10	0	13	4	0	3	5
LOC10105	13	3	8	3	1	10	3	0
LOC10263	111	106	119	119	159	138	77	98
LOC10263	3	0	1	0	2	2	1	1
LOC10263	1	4	0	1	0	0	0	1
LOC10816	4	1	0	0	0	2	0	0
LOC10816	27	39	37	28	41	58	30	48
LOC10816	36	31	38	21	11	18	26	18
LOC11484	63	32	50	44	25	37	40	15
LOC11548	0	4	0	1	1	0	0	2
LOC11548	2	12	18	20	0	11	2	15
LOC11548	22	41	40	30	44	32	35	10

Transcriptome sequencing yielded total genetic results for the MOD and APS groups, with a total of 15,936 variables

Continued from above

LOC11548	0	0	0	0	0	0	1	1
LOC11548	14	11	13	6	9	21	11	17
LOC11548	7	6	13	10	16	11	9	8
LOC11548	9	18	16	35	7	12	9	15
LOC11548	0	7	1	0	8	5	7	3
LOC11548	84	75	74	75	72	43	45	43
LOC11548	8	7	11	29	10	14	8	3
LOC11548	33	19	10	26	40	28	38	9
LOC11548	8	3	9	9	7	7	3	2
LOC11548	2	5	4	8	1	2	1	2
LOC11548	0	1	0	0	0	1	3	3
LOC11548	42	44	39	23	37	56	44	39
LOC11548	1	0	4	0	0	0	0	0
LOC11548	10	24	31	14	15	30	20	8
LOC11549	37	37	43	57	78	63	66	77
LOC11856	0	0	0	0	0	0	1	0
LOC11856	9	16	9	16	5	21	16	6
LOC11856	0	0	0	0	0	1	0	0
LOC11856	0	3	4	0	4	0	1	0
LOC11856	0	0	1	0	0	2	0	0
LOC11856	68	11	98	9	94	60	32	57
LOC11856	0	0	0	6	0	0	0	0
LOC11856	0	0	0	0	1	0	0	0
LOC11856	0	0	0	0	0	0	0	2
LOC11856	0	0	0	1	0	1	0	0
LOC11856	2	0	4	0	1	0	0	7
LOC11856	115	93	132	163	36	39	102	50
LOC11856	0	0	0	0	0	1	0	0
LOC11856	0	0	0	0	2	0	2	1
LOC11856	2	12	4	5	0	8	6	6
LOC11856	1093	1309	1336	1179	1216	1102	1077	1035
LOC11856	4	0	4	2	6	2	8	1
LOC11856	0	1	0	0	0	0	0	1
LOC11856	25	42	33	37	41	37	23	30
LOC11856	0	0	1	0	0	0	0	2
LOC11856	1	0	1	0	5	1	1	6
LOC11856	0	0	1	1	0	0	0	1
LOC11856	0	1	0	0	0	0	0	0
LOC11856	0	0	0	0	2	0	0	0
LOC11856	0	0	0	0	1	0	0	0
LOC11856	2	0	7	4	5	4	1	6
LOC11856	4	0	1	0	0	2	0	1
LOC11856	0	5	2	4	0	3	0	0
LOC11856	17	17	6	4	3	2	7	1
LOC11856	3	2	12	2	3	6	0	2
LOC11856	0	10	18	2	0	0	2	2
LOC11856	1	0	0	0	0	0	1	0
LOC11856	0	1	2	0	0	0	0	2
LOC11856	18	0	3	1	3	0	0	4
LOC11856	109	87	103	112	90	76	82	92
LOC11856	3	0	1	2	3	5	4	1
LOC11856	35	32	18	23	30	36	28	27
LOC11856	0	1	0	1	0	0	0	1
LOC11856	8	10	14	15	7	16	13	4
LOC11856	0	0	1	0	0	0	0	0
LOC11856	1053	1127	889	899	777	849	789	756
LOC11856	23	51	39	37	22	51	48	29
LOC11856	3	7	3	1	2	6	0	1

Transcriptome sequencing yielded total genetic results for the MOD and APS groups, with a total of 15,936 variables

Continued from above

LOC11856	1	1	1	0	0	0	0	0
LOC11856	4	1	0	6	2	5	0	2
LOC11856	67	86	67	81	100	85	64	60
LOC11856	0	1	0	0	0	0	1	0
LOC11856	9	17	7	5	14	2	6	10
LOC11856	0	0	0	1	0	0	0	0
LOC11856	0	0	0	1	0	0	0	0
LOC11856	22	11	4	9	4	4	5	10
LOC11856	16	3	4	1	10	5	10	4
LOC11856	7	4	3	2	9	5	1	11
LOC11856	0	0	0	0	0	0	1	1
LOC11856	0	0	0	0	0	1	0	0
LOC11856	0	2	0	0	1	0	0	0
LOC11856	1	1	0	1	0	1	0	0
LOC11856	0	1	0	0	0	2	0	0
LOC11856	38884	42565	37841	37419	37345	36181	37925	46257
LOC11856	0	0	0	0	0	0	0	1
LOC11856	1	0	0	0	0	0	0	0
LOC11856	1	0	0	0	0	0	0	0
LOC11856	1	0	0	0	0	0	0	0
LOC11856	0	1	0	0	2	0	0	0
LOC11856	1	0	0	1	0	0	0	0
LOC11856	7	14	5	1	4	1	4	4
LOC11856	63	72	62	78	41	68	60	36
LOC11856	24	13	14	14	7	12	24	13
LOC11856	0	0	0	4	4	0	0	1
LOC11856	18	1	10	21	7	6	5	18
LOC11856	1	0	0	0	0	1	0	0
LOC11856	1	3	7	3	0	6	1	2
LOC11856	0	0	0	0	0	0	1	0
LOC11856	1	2	2	2	3	2	1	8
LOC11856	146	181	224	200	114	142	166	232
LOC11856	19	38	36	32	41	34	21	37
LOC11856	8	4	6	4	2	4	7	1
LOC11856	2	1	0	1	0	4	0	0
LOC11856	94	111	121	99	143	113	112	124
LOC11856	0	5	2	0	0	1	1	0
LOC11856	13	3	6	4	15	6	3	4
LOC11856	397	395	377	348	421	388	376	347
LOC11856	3	1	4	13	0	7	4	13
LOC11856	0	0	4	0	0	0	0	0
LOC63007	2	2	3	1	3	6	2	1
LOC66633	0	0	0	3	0	0	1	0
LOC68395	499	374	224	176	203	132	97	78
LTO1	721	762	777	726	693	720	657	670
Lacc1	287	290	280	320	227	216	226	203
Lactb	709	823	851	933	880	836	816	870
Lactb2	1151	1230	1281	1308	1378	1399	1273	1218
Lactbl1	1	0	0	0	0	0	0	0
Lad1	10692	11846	10609	10355	10643	10791	10703	10177
Lag3	135	170	169	143	136	152	90	110
Lage3	351	350	385	397	450	431	349	439
Lair1	245	288	306	290	237	195	213	228
Lama1	92	96	131	68	100	100	71	92
Lama2	360	337	366	233	230	314	255	224
Lama3	300	293	181	280	428	383	398	348
Lama4	742	806	738	730	718	703	619	665
Lama5	1150	1207	1353	1323	1162	1180	1003	1165

Transcriptome sequencing yielded total genetic results for the MOD and APS groups, with a total of 15,936 variables

Continued from above

Lamb1	1784	2012	2097	2004	1751	1776	1571	1491
Lamb2	241	302	293	323	288	274	255	244
Lamb3	747	875	534	435	1035	1066	1135	1022
Lamc1	1664	1786	1825	1752	1572	1634	1433	1370
Lamc2	775	804	570	653	671	655	639	709
Lamc3	25	58	53	48	43	45	37	46
Lamp1	24706	25487	24510	24090	24732	25163	25919	24777
Lamp2	12832	14528	12756	12459	14663	15081	14566	13382
Lamp3	0	0	8	4	3	10	0	5
Lamtor1	2461	2512	2621	2504	2465	2278	2235	2197
Lamtor2	1802	1852	1937	1785	1768	1785	1760	1685
Lamtor3	1346	1313	1304	1312	1429	1361	1143	1417
Lamtor4	1113	1116	1079	958	1123	1095	1045	1122
Lamtor5	1176	1231	1179	1166	1279	1166	1107	1083
Lancl1	406	436	445	498	482	427	413	428
Lancl2	551	557	523	504	502	526	484	494
Lancl3	3	10	3	7	6	5	2	6
Lap3	21292	24279	20415	18315	23324	25172	25160	22525
Laptm4a	9140	9687	9613	8856	9339	9270	8669	8331
Laptm4b	2241	2559	2141	2105	2368	2413	2301	2134
Laptm5	981	919	1008	983	890	885	770	751
Large1	261	224	256	232	162	204	178	149
Large2	267	242	267	285	242	273	275	286
Larp1	5396	6129	5573	5391	5843	5686	5384	5056
Larp1b	4007	4317	4190	3893	4472	4448	4270	4152
Larp4	3488	3743	3750	3663	4332	4203	4033	3952
Larp4b	5706	6062	6085	6156	5680	6328	6461	5815
Larp6	17	39	16	15	11	17	9	15
Larp7	956	860	831	779	920	862	825	809
Lars	1762	1708	1633	1642	1632	1527	1558	1510
Lars2	4181	4911	4197	4029	4289	3938	4308	4920
Las1l	726	860	751	788	995	907	801	731
Lasp1	31901	33930	32228	30957	30867	32367	30895	29106
Lat	41	80	98	71	60	80	73	34
Lat2	148	105	115	105	84	105	112	103
Lats1	2432	2922	2474	2690	2764	3001	2813	2628
Lats2	572	591	539	542	613	530	562	523
Lax1	200	100	149	156	186	141	188	156
Layn	25	30	8	14	20	24	27	11
Lbh	1398	1343	1310	1390	1462	1410	1222	1256
Lbhd2	1	0	0	0	0	0	1	0
Lbp	86	101	81	90	82	104	119	103
Lbr	2351	2878	2851	2576	2787	2708	2521	2415
Lbx1	0	0	0	0	1	1	0	6
Lbx2	4	0	1	7	1	0	1	0
Lca5	15	24	25	17	26	18	13	11
Lca5l	1	5	0	3	1	0	1	0
Lcat	11	0	5	1	7	4	11	3
Lck	129	149	132	185	116	124	124	108
Lclat1	3412	3590	3418	3623	3467	3738	3698	3268
Lcmt1	1529	1627	1490	1386	1545	1613	1399	1451
Lcmt2	399	492	467	440	433	484	372	457
Lcn2	178	279	239	193	269	318	369	292
Lcor	878	991	1033	942	1001	951	923	1007
Lcorl	1505	1714	1606	1453	1661	1834	1663	1679
Lcp1	1311	1403	1366	1351	1317	1285	1141	1097
Lcp2	310	350	267	303	236	271	223	238
Lct	14572	18974	12091	8889	21432	22620	21724	16412

Transcriptome sequencing yielded total genetic results for the MOD and APS groups, with a total of 15,936 variables

Continued from above

Lctl	17	11	12	7	23	10	9	8
Ldah	1696	1701	1667	1767	1989	2027	1810	1639
Ldb1	993	993	1155	1075	1017	946	875	911
Ldb2	21	71	17	56	23	36	24	67
Ldb3	2919	3607	2936	2669	3803	3767	3593	3292
Ldc1	0	0	0	0	0	0	2	0
Ldha	101818	97746	99566	108630	96756	96762	96854	97541
Ldhal6b	5	14	5	7	4	4	0	3
Ldhb	128	120	102	154	107	132	153	121
Ldhc	0	1	0	1	0	0	4	1
Ldhd	218	313	293	310	317	275	255	297
Ldlr	4344	4583	5041	4754	4647	5278	4980	4681
Ldlrad1	2	1	5	0	0	0	0	5
Ldlrad3	72	47	45	31	31	70	48	51
Ldlrad4	107	67	114	139	99	107	79	93
Ldlrap1	854	870	984	996	825	826	799	830
Leap2	1255	1521	1665	1567	2108	2416	2466	2502
Lect2	3	0	5	4	0	2	1	1
Lef1	5	4	7	6	2	1	16	0
Lefty1	25	24	31	17	27	43	30	33
Lekr1	69	47	63	97	73	76	96	81
Lemd2	1467	1464	1529	1320	1492	1551	1406	1422
Lemd3	855	855	836	817	892	1008	891	757
Lenep	82	115	101	118	123	122	112	111
Leng1	443	531	583	532	440	459	586	470
Leng8	2118	2185	2488	2510	2380	2263	2090	1971
Leng9	1554	1657	1478	1364	1574	1709	1644	1635
Leo1	504	577	588	517	642	655	579	651
Lep	0	0	0	0	1	6	2	2
Lepr	311	325	366	388	349	456	350	247
Leprot	2612	3059	3064	2766	2766	2983	2797	2640
Leprotl1	1937	1913	1777	1852	1541	1695	1565	1576
Letm1	11462	11202	10857	11291	10910	10672	11087	10458
Letm2	72	28	22	36	49	41	36	43
Letmd1	801	749	735	841	757	756	706	735
Lexm	2	0	1	0	0	1	1	0
Lfng	201	172	224	203	199	212	177	142
Lgals1	758	807	831	848	803	839	774	724
Lgals12	230	297	289	232	296	292	282	281
Lgals2	33654	37287	36944	35976	38029	37751	34365	36878
Lgals3	18112	16819	17088	18394	17051	17520	17874	18188
Lgals3bp	20435	25508	21981	17847	24132	22682	20736	20852
Lgals4	99557	105824	104130	101971	98168	102020	98893	98581
Lgals7	0	0	0	10	1	1	0	1
Lgals8	3805	4160	3799	3673	3803	4078	4071	3620
Lgals9	41524	45444	43439	39940	42298	41192	38091	37618
Lgalsl	1792	1717	1647	1699	1716	1856	1737	1673
Lgi1	6	2	0	5	1	1	0	4
Lgi2	41	71	70	78	60	61	58	87
Lgi3	27	17	6	23	13	19	2	9
Lgi4	36	50	59	67	80	24	51	70
Lgmn	3268	3452	3363	3172	3022	3061	3068	3156
Lgr4	5296	5314	5006	5131	5330	4817	4596	4617
Lgr5	119	163	177	181	150	193	133	161
Lgr6	1	11	1	3	4	4	15	5
Lhfp	437	421	415	441	394	364	340	339
Lhfpl1	2	2	5	10	5	6	1	1
Lhfpl2	1285	1278	1055	1168	1018	1108	1229	1232

Transcriptome sequencing yielded total genetic results for the MOD and APS groups, with a total of 15,936 variables

Continued from above

Lhfpl3	0	0	4	0	0	0	0	0
Lhfpl4	2	8	1	9	8	2	14	7
Lhfpl5	0	1	0	3	1	1	0	2
Lhpp	372	367	425	376	417	482	388	319
Lhx1	1	10	10	12	6	4	5	8
Lhx2	0	0	0	0	0	0	8	0
Lhx3	0	0	5	0	0	1	0	0
Lhx5	8	0	9	4	2	6	1	10
Lhx6	4	14	25	16	8	16	9	12
Lias	1108	1006	1117	1092	1022	1063	1017	990
Lif	186	268	277	278	272	236	207	198
Lifr	262	340	309	333	316	306	274	347
Lig1	1350	1383	1514	1376	1174	1159	1084	1087
Lig3	989	1276	1108	958	1127	1170	1179	1037
Lig4	487	466	424	436	506	590	457	436
Lilr4b	73	87	68	81	69	34	42	40
Lilra5	80	110	85	83	97	58	88	68
Lilra6	0	1	5	1	5	3	1	2
Lilrb4a	78	130	91	127	60	116	90	98
Lima1	16210	16545	15613	15988	15454	16539	15993	15184
Limch1	70	65	58	54	59	51	53	48
Limd1	4511	4709	4432	4251	5120	5020	5027	4573
Limd2	321	277	363	322	303	323	265	218
Lime1	416	412	403	444	433	349	363	422
Limk1	116	113	111	154	155	113	118	121
Limk2	2932	3164	3299	3248	2957	2961	2867	2948
Lims1	1422	1437	1550	1407	1325	1445	1464	1282
Lims2	242	221	224	260	244	260	231	234
Lin28a	0	1	0	1	0	1	1	0
Lin28b	0	0	0	0	1	0	0	0
Lin37	334	420	442	399	433	368	354	345
Lin52	208	182	170	218	196	187	183	219
Lin54	637	778	741	703	774	779	766	727
Lin7a	0	15	10	5	12	7	9	19
Lin7b	7	8	4	1	1	5	9	5
Lin7c	3449	3683	3476	3914	3556	4069	4105	3730
Lin9	308	358	323	299	302	337	232	254
Lingo1	0	2	0	6	12	0	17	2
Lingo2	0	0	0	1	0	1	0	0
Lingo3	5	0	1	0	10	1	4	0
Lingo4	10	5	3	8	2	5	4	0
Lins1	126	137	96	99	99	110	128	121
Lipa	3168	3853	3230	2882	4064	3932	4063	3756
Lipc	21	26	11	18	37	16	20	27
Lipe	4493	4967	4121	4063	3928	4580	4787	4286
Lipg	628	696	603	565	690	706	524	514
Liph	3012	3467	3102	2575	3373	3559	3297	3539
Lipk	7	8	5	6	5	8	1	9
Lipn	0	1	0	0	0	0	0	0
Lipo2	0	0	0	0	3	2	0	0
Lipo3	251	293	302	234	238	347	289	298
Lipo4	0	0	0	0	0	0	1	0
Lipt1	77	75	70	77	104	94	53	85
Lipt2	246	280	255	260	313	257	281	194
Litaf	6936	7083	6357	6066	6124	6387	6584	6380
Lix1	3	3	9	3	9	13	15	3
Lix1l	229	197	193	215	241	206	171	189
Llgl1	1114	1107	1074	1010	916	979	923	940

Transcriptome sequencing yielded total genetic results for the MOD and APS groups, with a total of 15,936 variables

Continued from above

Llgl2	7507	7627	7070	7170	7363	7398	7223	6801
Llph	639	756	678	654	756	707	601	727
Llph-ps2	10	14	8	16	19	13	17	8
Lman1	4655	5122	4759	4688	5532	5552	5140	5047
Lman1l	1	0	0	5	6	0	0	0
Lman2	11126	12122	11359	10810	12910	12626	11473	11448
Lman2l	485	491	443	540	689	689	562	543
Lmbr1	425	395	397	501	478	440	409	422
Lmbr1l	2036	2092	2053	1959	2041	2082	2072	1735
Lmbrd1	3067	3329	3087	2857	3374	3423	3511	3052
Lmbrd2	2523	2742	2673	2559	3032	3272	3216	2922
Lmcd1	261	381	369	345	384	316	305	279
Lmf1	593	554	585	585	658	614	629	570
Lmf2	734	832	794	827	858	747	720	717
Lmln	86	122	90	139	96	119	87	109
Lmna	5431	5439	5773	5664	6105	6039	5486	5856
Lmnb1	2235	2567	2299	2234	2507	2305	2058	2077
Lmnb2	2003	1897	2041	1841	2017	2157	1930	1827
Lmntd1	0	0	0	0	0	4	0	0
Lmntd2	100	103	82	93	81	82	93	89
Lmo1	3	2	2	5	0	5	2	6
Lmo2	362	393	325	348	326	359	243	314
Lmo3	13	12	4	4	16	2	16	8
Lmo4	540	580	515	560	526	483	473	525
Lmo7	9069	9949	8491	8302	9315	10244	10734	9404
Lmod1	331	405	405	322	383	412	444	339
Lmtk2	5442	6096	5435	5109	6012	5897	6218	5780
Lmtk3	34	37	19	21	32	13	7	15
Lmx1a	49	33	29	32	27	27	27	19
Lmx1b	1	5	1	1	6	1	5	2
Lnpep	901	1035	1002	1043	1095	1129	1218	971
Lnpk	532	549	522	556	536	592	523	554
Lnx1	1458	1347	1419	1692	1360	1257	1418	1270
Lnx2	1127	1192	1130	1164	1148	1149	1082	1089
Lonp1	3150	3258	3431	3065	3488	3461	3029	3159
Lonp2	4065	4496	3987	3924	4335	4462	4252	3761
Lonrf1	84	110	74	70	109	74	69	88
Lonrf2	18	18	6	14	7	7	6	4
Lonrf3	124	124	124	142	178	107	135	134
Lor	0	1	5	1	0	5	0	0
Lox	90	98	115	116	72	74	80	79
Loxl1	170	163	206	219	234	199	216	186
Loxl2	502	587	581	560	685	611	696	517
Loxl3	112	142	106	137	121	113	130	123
Loxl4	93	100	114	109	91	88	73	77
Lpar1	1354	1300	1226	1116	1296	1407	1190	1118
Lpar2	90	118	95	115	153	145	136	120
Lpar3	143	197	170	133	232	233	214	175
Lpar4	0	5	3	4	1	2	3	3
Lpar5	2671	2994	2753	2682	2906	2975	2939	2884
Lpar6	288	326	335	336	388	420	320	346
Lpcat1	184	176	221	210	185	211	161	197
Lpcat2	321	345	265	308	260	243	276	242
Lpcat3	13663	14605	13975	13864	13770	13586	13633	13132
Lpcat4	410	386	461	340	488	427	311	434
Lpgat1	27990	28807	26423	27468	28548	30137	32261	29027
Lpin1	674	680	688	811	449	493	394	367
Lpin2	4393	4582	4564	4686	3950	4206	4443	3921

Transcriptome sequencing yielded total genetic results for the MOD and APS groups, with a total of 15,936 variables

Continued from above

Lpin3	3785	4182	3610	3610	3721	3991	4341	3992
Lpl	160	157	206	241	218	254	254	180
Lpo	1	1	0	0	0	1	0	1
Lpp	3864	4246	4288	4445	4187	4293	4493	4192
Lpxn	127	114	89	90	132	113	106	88
Lrat	887	989	991	1005	897	959	925	785
Lratd1	1719	1909	1720	1638	1703	1915	1990	1794
Lratd2	3897	4737	4351	4359	4817	4986	4496	4362
Lrba	6024	6601	6039	6309	5893	6235	6011	5563
Lrch1	827	842	938	919	767	828	643	620
Lrch2	22	18	31	26	29	25	12	24
Lrch3	1065	1198	1277	1023	1185	1061	1070	1065
Lrch4	1731	1652	1541	1556	1594	1370	1324	1405
Lrfn1	19	2	13	11	4	11	17	8
Lrfn2	0	2	0	0	1	1	4	0
Lrfn3	479	425	417	414	443	420	370	391
Lrfn4	472	555	397	346	440	355	320	370
Lrfn5	7	2	0	2	1	0	0	2
Lrg1	331	292	302	276	944	939	961	1316
Lrguk	0	0	0	1	1	4	1	7
Lrif1	392	361	442	466	421	522	408	390
Lrig1	2471	2723	2696	2648	2664	2552	2193	2189
Lrig2	575	586	595	694	578	630	563	537
Lrig3	1079	1125	1160	1167	1053	1119	973	1037
Lrit3	0	1	0	0	0	0	0	0
Lrmda	11	13	5	6	5	8	10	18
Lrmp	117	127	120	108	147	82	69	118
Lrp1	10018	10771	10223	9679	10170	10302	10120	9764
Lrp10	11496	11730	11053	10944	10914	10610	10279	10563
Lrp11	61	48	78	81	61	39	53	65
Lrp12	379	432	357	402	397	437	498	378
Lrp1b	0	0	0	0	0	1	0	0
Lrp2	6	5	1	2	2	1	2	0
Lrp2bp	11	7	3	14	9	3	6	7
Lrp3	56	89	104	77	76	99	81	45
Lrp4	1034	1147	1206	1049	1183	1184	1070	1086
Lrp5	3902	3889	3949	4029	3404	3511	3417	3325
Lrp6	2796	3267	3123	3085	3151	3099	3436	2887
Lrp8	139	136	135	127	136	118	120	162
Lrpap1	771	791	860	763	823	794	723	755
Lrpprc	3503	3646	3838	4049	4035	3942	3573	3402
Lrr1	26	44	39	66	50	57	37	38
Lrrc1	4174	4562	3947	3887	4138	4041	4012	3946
Lrrc10b	8	20	12	4	18	4	14	15
Lrrc14	445	464	374	457	476	463	417	488
Lrrc14b	9	6	7	8	2	2	2	5
Lrrc15	5	0	1	5	5	0	5	0
Lrrc17	17	14	25	22	34	15	48	9
Lrrc18	0	0	1	3	1	8	1	0
Lrrc19	3241	3608	3058	3038	3899	4073	4153	3753
Lrrc2	0	1	0	0	1	1	0	1
Lrrc20	519	506	466	536	507	575	429	499
Lrrc23	1	0	1	0	0	4	4	0
Lrrc24	1	5	7	2	3	4	1	2
Lrrc25	142	112	127	150	110	125	99	115
Lrrc26	735	705	852	791	607	554	618	625
Lrrc27	16	7	11	13	11	16	19	18
Lrrc28	312	398	334	365	384	391	312	346

Transcriptome sequencing yielded total genetic results for the MOD and APS groups, with a total of 15,936 variables

Continued from above

Lrrc29	84	82	83	101	48	73	60	65
Lrrc3	98	218	142	123	114	97	189	170
Lrrc31	528	527	564	514	439	458	505	448
Lrrc32	447	434	369	447	381	351	394	344
Lrrc34	0	0	0	0	0	1	0	0
Lrrc36	0	0	0	1	0	5	1	0
Lrrc37a	0	0	0	1	0	0	0	0
Lrrc39	18	27	32	33	20	27	44	39
Lrrc3b	12	0	7	13	5	6	9	9
Lrrc4	17	15	31	22	16	16	23	19
Lrrc40	821	876	840	909	880	914	926	795
Lrrc41	3339	3308	2951	2853	3181	3188	3085	2960
Lrrc42	652	701	660	630	664	688	650	646
Lrrc45	775	803	833	767	895	797	660	727
Lrrc46	9	1	8	2	14	2	6	13
Lrrc47	2045	2040	1912	1810	2136	2031	1928	2068
Lrrc49	154	146	150	205	173	167	165	139
Lrrc4b	11	3	3	2	2	2	6	12
Lrrc4c	5	10	14	18	10	6	12	9
Lrrc51	43	59	57	44	84	53	52	45
Lrrc55	24	34	29	28	20	38	23	17
Lrrc56	195	189	211	174	195	209	192	190
Lrrc57	869	964	845	866	797	875	939	762
Lrrc58	1051	1090	1054	1119	1161	1164	1123	989
Lrrc59	24960	25826	24057	23986	23828	24255	23582	23066
Lrrc6	0	0	0	0	0	0	1	0
Lrrc61	315	284	359	384	293	342	328	255
Lrrc63	0	1	0	0	0	1	0	0
Lrrc66	1444	1777	1408	1328	1498	1350	1533	1458
Lrrc69	0	1	0	0	0	4	0	1
Lrrc7	6	5	5	0	1	1	5	0
Lrrc70	1	6	7	19	6	2	1	1
Lrrc71	27	26	19	30	11	36	32	16
Lrrc72	0	0	0	0	1	0	0	0
Lrrc73	11	2	5	1	8	4	4	3
Lrrc74b	1	0	5	0	1	0	1	8
Lrrc75a	2421	2764	2697	2387	2608	2542	2688	2301
Lrrc75b	106	87	78	115	101	95	157	96
Lrrc8a	1381	1403	1432	1448	1448	1476	1457	1353
Lrrc8b	3740	3875	3292	3471	4069	4300	4616	3957
Lrrc8c	353	285	303	407	263	276	293	254
Lrrc8d	2807	3221	2840	2463	3213	3142	3241	3001
Lrrc8e	8	5	12	13	3	9	10	0
Lrrc9	0	2	1	0	0	0	1	0
Lrrcc1	152	160	144	165	127	167	147	157
Lrrd1	0	1	9	6	12	3	6	6
Lrrfip1	3741	3768	3496	3810	3392	3743	3826	3671
Lrrfip2	2182	2374	2389	2229	2340	2403	2170	2136
Lrriq3	6	0	0	0	1	2	0	0
Lrrk1	1721	1789	1805	1616	2031	1776	1616	1510
Lrrk2	51	48	67	57	75	57	59	54
Lrrn1	21	4	10	11	15	9	9	18
Lrrn2	17	14	7	21	14	24	13	23
Lrrn3	17	9	10	4	3	8	12	10
Lrrn4	14	21	15	11	15	34	31	51
Lrrn4cl	8	16	18	10	18	11	16	5
Lrrtm1	3	4	2	1	0	2	1	5
Lrrtm2	0	12	3	2	2	1	0	13

Transcriptome sequencing yielded total genetic results for the MOD and APS groups, with a total of 15,936 variables

Continued from above

Lrrtm3	0	1	1	0	3	2	4	4
Lrrtm4	0	1	0	4	4	2	7	0
Lrsam1	444	517	471	443	498	552	444	473
Lrtm2	0	2	0	0	0	0	0	0
Lrwd1	534	596	536	498	562	493	462	468
Lsamp	15	16	16	24	10	29	24	18
Lsg1	597	648	611	592	669	655	657	646
Lsm1	790	810	716	745	855	960	802	813
Lsm10	101	150	171	190	223	187	124	136
Lsm11	220	299	179	198	258	202	210	197
Lsm12	1413	1689	1488	1520	1797	1728	1702	1502
Lsm14a	3167	3382	2990	3156	3322	3156	3214	3039
Lsm14b	905	902	885	997	993	885	881	872
Lsm2	553	612	574	538	650	601	537	594
Lsm3	1019	1203	1193	1141	1292	1294	1094	1138
Lsm4	2388	2446	2466	2231	2458	2353	2029	2291
Lsm5	318	320	287	252	334	336	286	306
Lsm6	695	784	715	670	692	724	569	693
Lsm7	287	321	309	332	317	352	298	336
Lsm8	771	660	782	814	749	805	684	761
Lsmem1	10	4	4	6	9	1	3	7
Lsmem2	30	28	22	27	31	21	16	8
Lsp1	280	266	278	269	266	268	230	234
Lsr	8605	9129	8696	8554	8621	8368	8121	7836
Lss	1542	1581	1792	1709	1421	1392	1346	1334
Lst1	64	65	42	61	58	65	57	55
Lta	5	6	4	1	1	0	3	0
Lta4h	9151	9931	9381	9034	9566	9890	9447	8576
Ltb	94	77	82	76	77	87	51	40
Ltb4r1	782	745	749	744	706	825	721	756
Ltb4r2	1523	1553	1431	1450	1595	1653	1605	1494
Ltbp1	670	714	700	700	700	634	594	526
Ltbp2	179	133	148	173	180	116	150	133
Ltbp3	248	349	368	309	268	251	315	245
Ltbp4	1845	2102	2009	1896	1861	1807	1590	1707
Ltbr	7028	7529	6909	6753	7690	6714	6662	6695
Ltc4s	253	214	202	227	254	220	204	195
Ltf	309	279	314	274	361	305	374	376
Ltk	7	18	21	23	14	17	13	27
Ltn1	2217	2547	2064	2016	2317	2315	2258	2132
Ltv1	882	957	837	861	1011	886	877	877
Luc7l	1158	1143	1247	1141	1471	1324	1103	1098
Luc7l2	2077	2207	2279	2152	2130	2333	2080	2011
Luc7l3	1558	1717	1768	1852	2015	1977	2018	1806
Lum	441	470	465	405	536	457	490	405
Lurap1	6	11	2	12	1	7	12	3
Lurap1l	2979	3195	3174	2858	3226	3433	3149	3152
Luzp1	6960	7938	6712	6653	7558	7835	8210	6848
Luzp2	0	2	0	0	1	0	0	0
Lvrn	0	9	2	1	14	8	2	7
Lxn	455	569	496	417	561	544	477	482
Ly6a	5317	6881	6862	5730	6731	5478	4276	5407
Ly6a2	11	0	1	4	0	1	1	1
Ly6c1	404	444	530	471	335	295	304	262
Ly6c2	273	302	245	262	200	243	252	172
Ly6d	253	257	214	211	304	253	288	312
Ly6e	15522	19170	17424	12921	19463	16852	15277	16104
Ly6f	1	2	6	4	9	6	3	4

Transcriptome sequencing yielded total genetic results for the MOD and APS groups, with a total of 15,936 variables

Continued from above

Ly6g	1	0	0	0	1	0	0	0
Ly6g2	4001	4095	3928	3856	4176	4156	4216	3808
Ly6g5b	12	7	16	13	14	7	17	0
Ly6g6c	1	0	0	0	1	5	1	1
Ly6g6d	30	24	23	45	42	34	26	32
Ly6g6e	5	14	8	4	12	4	7	13
Ly6h	1	0	1	0	12	6	1	1
Ly6i	13	3	29	7	3	0	31	16
Ly6k	5	2	2	4	7	6	6	5
Ly6l	5	1	0	0	3	0	0	0
Ly6m	5427	5154	4975	5569	5020	6146	7355	6486
Ly75	2615	2876	2540	2598	2449	2456	2742	2310
Ly86	205	166	152	157	154	113	149	118
Ly9	87	46	56	47	66	68	42	30
Ly96	143	166	127	143	138	119	116	95
Lyar	551	648	633	581	702	607	536	549
Lyl1	47	74	44	58	29	40	41	31
Lyn	3725	4177	3692	3704	3967	4263	3998	3578
Lynx1	134	181	141	163	175	133	121	173
Lypd1	64	56	57	59	63	50	54	51
Lypd3	0	0	0	0	1	0	1	0
Lypd6	18	21	17	18	15	16	8	18
Lypd6b	1	7	9	6	18	1	0	1
Lypd8	207001	258784	240961	194567	271024	258083	239598	239282
Lypd8l	25342	27647	24009	22501	34816	33970	27639	26074
Lypla1	14204	15621	14416	14725	14042	14833	14966	14034
Lypla2	2465	2662	2392	2497	2471	2375	2387	2354
Lyplal1	60	79	80	63	64	91	78	98
Lyrm1	147	118	126	131	105	125	125	103
Lyrm2	304	327	256	294	312	314	323	270
Lyrm4	210	244	246	193	230	195	197	170
Lyrm7	94	97	109	111	121	107	104	107
Lyrm9	324	323	300	341	353	324	295	378
Lysmd1	200	203	176	222	158	223	201	249
Lysmd2	177	192	148	112	155	135	115	131
Lysmd3	2308	2560	2251	2202	2782	3078	3279	2684
Lysmd4	895	1120	921	906	1036	1212	1151	1044
Lyst	269	317	293	314	313	375	325	295
Lyve1	539	583	652	588	600	653	712	777
Lyz1	36011	32943	33569	36589	25354	27905	35962	36012
Lyz2	3234	2701	2472	3043	3268	3079	2749	2605
Lyzl4	5	3	7	2	4	5	4	1
Lzic	1643	1612	1502	1459	1445	1526	1444	1397
Lztfl1	342	464	299	370	375	389	394	352
Lztr1	1778	1756	1837	1870	1729	1834	1727	1791
Lzts1	13	8	8	8	7	9	2	2
Lzts2	1241	1264	1346	1298	1363	1383	1137	1222
Lzts3	4788	5128	5043	4720	4736	4881	4625	4510
M1ap	0	0	1	0	0	4	0	1
M6pr	5516	5999	5544	5477	6292	5917	5733	5203
Maats1	9	6	2	4	17	2	6	3
Mab21l1	0	6	13	3	10	7	3	4
Mab21l2	220	259	228	164	175	222	219	193
Mab21l3	14	16	21	10	9	11	15	3
Mab21l4	185	203	178	178	167	188	164	202
Macc1	40	33	33	32	48	30	25	33
Macf1	1887	2277	2176	2244	2177	2141	2336	1942
Macir	2556	2680	2568	2298	2428	2621	2617	2424

Transcriptome sequencing yielded total genetic results for the MOD and APS groups, with a total of 15,936 variables

Continued from above

Maco1	1161	1377	1330	1222	1417	1493	1363	1179
Macrod1	474	372	379	393	290	336	314	306
Macrod2	40	74	66	67	80	71	56	57
Macroh2a	4843	5301	5249	5104	5180	5108	4659	4527
Macroh2a	92	101	115	97	87	82	63	84
Mad1l1	464	522	449	426	479	432	413	440
Mad2l1	629	807	758	804	787	816	690	740
Mad2l1bp	743	766	757	744	718	724	702	702
Mad2l2	402	393	416	391	350	426	386	385
Madcam1	84	77	74	97	59	64	47	84
Madd	1065	1069	1028	969	1183	1030	976	986
Maea	4354	4571	4479	4527	4692	4362	4129	4339
Maf	6669	7192	6497	6461	6034	6881	6872	5637
Maf1	2872	3159	2945	3085	2998	2858	2729	2681
Mafa	0	0	0	8	0	1	0	8
Mafb	4570	4904	4568	4698	4141	4455	4776	4429
Maff	603	542	581	606	662	675	686	612
Mafg	1679	1590	1819	1719	1928	1803	1574	1695
Mafk	2783	3046	3031	2630	2859	2785	2752	2561
Mag	15	14	20	15	15	2	10	3
Mageb18	0	0	1	0	0	0	0	0
Mageb3	0	1	1	0	1	1	0	0
Maged1	854	942	878	850	941	1015	929	882
Maged2	177	210	211	217	209	280	222	162
Magee1	67	47	56	43	83	38	66	43
Magee2	0	0	0	0	0	0	0	1
Mageh1	65	45	53	56	67	64	54	60
Magi1	1418	1700	1517	1551	1564	1738	1639	1424
Magi2	27	24	26	21	12	17	22	35
Magi3	2691	2983	2727	2777	3041	3130	3429	2635
Magix	7	12	4	12	5	2	3	26
Magoh	1023	1023	1079	1113	1208	1187	1173	1082
Magohb	200	275	258	291	247	236	214	243
Magt1	4438	5024	4751	4655	4998	5300	4693	4520
Maip1	892	826	813	771	792	762	826	807
Majin	15	27	18	23	9	28	11	12
Mak	0	1	6	1	2	12	1	1
Mak16	539	749	637	690	778	732	640	693
Mal	16	42	16	12	54	33	31	22
Mal2	7710	8490	8020	8013	8320	8847	8290	8496
Mall	11279	12511	10929	10104	12031	12431	11963	11811
Malrd1	1918	2375	1952	1712	1911	2201	2161	1619
Malsu1	748	629	699	605	781	745	743	668
Malt1	695	744	705	737	607	665	697	682
Mamdc2	45	48	60	55	35	70	56	35
Mamdc4	33	23	22	37	25	23	31	23
Maml1	867	957	991	878	843	863	787	825
Maml2	69	94	77	112	67	77	52	48
Maml3	218	251	220	232	239	182	169	202
Mamld1	6	35	18	31	13	12	30	8
Mamstr	1	10	10	9	3	8	1	12
Man1a	6138	7092	6105	5924	6511	6758	6945	6389
Man1a2	3729	3946	3819	3852	4221	4432	4018	3716
Man1b1	3911	4201	4049	4000	4242	4154	3999	3949
Man1c1	163	206	231	170	195	176	177	190
Man2a1	4040	4144	4481	4108	4422	4454	4236	4074
Man2a2	454	548	492	591	532	533	431	498
Man2b1	2990	3457	3143	2890	3187	3012	2705	2789

Transcriptome sequencing yielded total genetic results for the MOD and APS groups, with a total of 15,936 variables

Continued from above

Man2b2	2128	2261	2178	2040	2402	2285	2170	2308
Man2c1	945	926	878	796	902	800	850	803
Manba	1256	1185	1230	1156	1055	1215	1171	1042
Manbal	672	723	704	695	575	674	629	648
Manea	736	746	685	797	730	818	727	613
Maneal	1	11	5	0	1	2	5	6
Manf	5110	5487	5048	4988	6616	6091	5447	5441
Mansc1	509	542	485	486	468	424	442	441
Mansc4	2	9	0	1	2	0	0	0
Maoa	29117	35073	29989	27331	35386	37406	35644	32416
Maob	865	1251	1039	852	1183	1147	1170	809
Map10	91	72	34	38	60	53	29	80
Map11	893	940	997	930	1044	933	963	1011
Map1a	134	185	165	153	113	132	120	120
Map1b	248	309	241	259	244	242	235	255
Map1lc3a	477	470	518	575	569	496	514	514
Map1lc3b	3311	3472	3334	3388	3377	3375	3081	3298
Map1s	449	369	357	423	386	454	402	362
Map2	53	31	34	40	28	58	33	35
Map2k1	6318	6519	6190	5735	6482	6669	6582	6194
Map2k2	11530	11072	11618	11086	10326	10621	10326	10438
Map2k3	5293	5546	5438	5258	5966	5854	5220	5501
Map2k4	3813	4091	3778	3741	3537	3730	3603	3408
Map2k5	477	437	367	403	478	446	345	387
Map2k6	283	306	302	243	269	258	279	268
Map2k7	422	510	479	540	411	394	451	402
Map3k1	1233	1328	1339	1292	1211	1478	1529	1269
Map3k10	382	307	367	365	423	347	329	412
Map3k11	5815	5934	5758	5447	5994	6057	5715	5501
Map3k12	149	191	216	190	179	119	108	150
Map3k13	1419	1445	1339	1574	1426	1431	1570	1288
Map3k14	258	236	277	281	342	301	306	284
Map3k15	199	246	217	238	176	204	188	227
Map3k2	2760	3272	2996	3120	3016	3004	3050	2938
Map3k20	511	525	553	484	541	606	565	547
Map3k21	2139	2242	2227	2228	2298	2236	1977	1850
Map3k3	920	1008	1017	907	906	893	955	794
Map3k4	1511	1541	1561	1433	1445	1382	1267	1184
Map3k5	663	775	722	633	813	792	653	692
Map3k6	263	297	236	203	228	263	273	279
Map3k7	1740	1913	1789	1707	1840	1852	1789	1745
Map3k7cl	11	3	6	4	1	15	1	5
Map3k8	65	43	53	61	40	50	45	31
Map3k9	742	800	757	734	646	690	634	566
Map4	4288	4487	4155	4445	3851	3850	3785	3789
Map4k1	82	70	67	53	89	85	51	77
Map4k2	351	243	345	317	254	351	233	254
Map4k3	1434	1504	1547	1471	1560	1550	1572	1517
Map4k4	2212	2382	2561	2514	2423	2297	2145	2099
Map4k5	328	400	417	409	423	417	396	402
Map6	22	49	33	26	42	60	40	37
Map6d1	1	1	0	0	1	0	4	4
Map7	5389	5759	5314	4909	5946	5770	5749	5753
Map7d1	2518	2173	2136	2445	2037	1964	1969	2006
Map7d2	3	2	3	0	2	5	2	9
Map7d3	0	0	4	0	5	6	5	2
Map9	10	21	22	15	34	11	10	18
Mapk10	6	19	20	28	6	10	30	24

Transcriptome sequencing yielded total genetic results for the MOD and APS groups, with a total of 15,936 variables

Continued from above

Mapk11	37	11	27	23	18	28	20	24
Mapk12	36	58	47	44	30	31	42	44
Mapk13	13666	13748	13068	13496	12197	13141	13180	12904
Mapk14	2685	2929	2590	2770	3118	3055	2941	2764
Mapk15	5	2	9	5	6	5	5	2
Mapk1ip1	181	174	209	205	176	221	185	189
Mapk1ip1	5499	6058	5503	5411	5854	5919	5938	5555
Mapk4	29	30	34	17	24	37	31	40
Mapk6	4266	4601	4554	4527	4423	4672	5089	4826
Mapk7	342	332	376	421	378	393	402	368
Mapk8	2291	2511	2183	2159	2113	2343	2375	2171
Mapk8ip1	84	82	106	65	83	100	100	70
Mapk8ip2	32	38	31	22	37	38	30	36
Mapk8ip3	1230	1135	1182	1313	1175	1064	1156	1070
Mapk9	1597	1540	1547	1631	1548	1589	1534	1334
Mapkap1	2840	3054	2923	2986	2965	3117	3102	2964
Mapkapk2	5836	5794	5570	5632	5307	5195	5165	5108
Mapkapk3	1661	1662	1707	1646	1681	1463	1394	1543
Mapkapk5	2842	2907	2959	2872	3125	3285	3062	3021
Mapkbp1	803	809	676	618	609	683	715	625
Mapre1	3588	3718	3588	3689	3926	3978	3766	3265
Mapre2	1282	1622	1510	1263	1562	1509	1425	1394
Mapre3	959	970	877	905	778	876	1016	960
Mapt	95	135	141	134	145	129	170	143
Marchf1	161	115	88	157	137	122	138	169
Marchf10	0	0	0	0	0	0	0	1
Marchf11	1	0	0	0	0	0	0	0
Marchf2	1339	1486	1284	1389	1486	1497	1467	1335
Marchf3	50	25	39	29	46	24	32	41
Marchf4	12	6	8	7	20	6	10	6
Marchf5	3905	3668	3667	3446	3105	3412	3392	3218
Marchf6	7605	8802	7369	7420	8173	8371	8788	7963
Marchf7	3152	3482	3232	3170	2847	3089	3465	2970
Marchf8	5596	6278	6149	5996	5915	6015	6300	5879
Marchf9	148	107	122	130	79	120	91	106
Marcks	3001	2899	2757	3052	2846	2816	2567	2926
Marcksl1	643	623	668	675	558	633	520	512
Marco	4	0	1	1	4	0	1	1
Marf1	2365	2886	2606	2410	2809	2718	2713	2454
Mark1	50	81	83	48	41	51	72	27
Mark2	5895	6444	6380	5830	5974	6292	5723	5726
Mark3	2529	2492	2495	2269	2728	2648	2515	2496
Mark4	877	1026	1091	983	853	851	836	945
Mars1	2032	2088	1814	1681	1805	1866	1697	1696
Mars2	381	414	405	419	477	441	437	362
Marveld1	412	402	578	409	410	495	487	437
Marveld2	1158	1404	1449	1305	1305	1225	1184	1059
Marveld3	3087	3340	3296	2998	3631	3207	3388	3274
Mas1	0	0	0	0	0	1	0	0
Masp1	13	24	20	25	7	8	17	12
Masp2	25	41	27	11	17	3	15	8
Mast1	9	23	17	30	24	31	28	14
Mast2	6275	7134	6798	6255	7122	7229	6873	6277
Mast3	1259	1270	1133	1206	1202	1242	1262	1097
Mast4	652	654	750	693	745	691	497	629
Mastl	181	193	223	183	198	222	140	151
Mat1a	12	19	13	28	44	77	32	25
Mat2a	3776	4010	3669	3752	4056	4199	3648	3734

Transcriptome sequencing yielded total genetic results for the MOD and APS groups, with a total of 15,936 variables

Continued from above

Mat2b	2485	2614	2603	2718	2782	2601	2586	2539
Matk	24	25	19	42	40	47	38	37
Matn2	330	352	378	308	397	340	278	308
Matn4	7	17	2	8	2	2	7	11
Matr3	2464	2641	2439	2413	2851	2846	2673	2580
Mau2	3592	3858	3774	3736	4103	4001	3783	3795
Mavs	4488	4657	4291	4552	4815	4714	4992	4886
Max	8193	8657	8114	7334	7271	7765	8642	7528
Maz	2958	2893	3056	2764	3023	2745	2440	2761
Mb	1	0	0	0	0	1	0	1
Mb21d2	917	980	921	960	842	847	960	769
Mbd1	1499	1792	1604	1621	1744	1717	1523	1729
Mbd2	4459	4957	4568	4497	4421	4596	4336	4207
Mbd3	1674	1882	1802	1700	1797	1690	1524	1576
Mbd4	108	68	86	112	87	76	89	42
Mbd5	297	301	285	294	358	331	303	345
Mbd6	1954	2033	2062	2075	1897	2041	1913	1961
Mbip	216	233	258	264	231	213	242	237
Mbl1	2	0	0	2	1	0	0	0
Mbl2	507	484	397	469	434	544	447	395
Mblac1	33	35	46	25	29	42	41	50
Mblac2	48	56	73	89	51	88	65	82
Mbnl1	2666	2840	2675	2992	2811	3015	2932	2571
Mbnl2	3680	3774	3711	3816	3870	4369	4169	4124
Mbnl3	713	722	699	722	669	731	732	743
Mboat1	476	556	560	673	803	626	473	682
Mboat2	18	9	15	22	19	16	21	17
Mboat4	10	40	17	10	8	14	7	17
Mboat7	1930	2288	2155	2011	2208	2150	2154	2165
Mbp	260	267	257	250	271	257	246	210
Mbtd1	925	998	930	946	902	981	752	897
Mbtps1	3838	4235	4192	3954	4598	4309	4249	3794
Mbtps2	583	660	580	552	529	584	517	514
Mc1r	0	8	2	0	0	0	0	0
Mc4r	0	1	1	3	0	0	6	0
Mc5r	1	6	5	7	1	1	2	2
Mcam	596	709	645	711	602	579	545	631
Mcat	468	492	465	505	505	501	349	422
Mcc	100	140	159	109	113	143	145	81
Mccc1	831	821	914	920	857	974	931	948
Mccc2	796	763	680	739	733	735	747	762
Mcee	518	524	635	537	667	637	541	572
Mcemp1	5	4	8	5	2	1	1	0
Mcf2l	861	957	952	971	786	846	822	697
Mcfd2	9734	10361	9974	9177	10995	11764	11591	10802
Mchr1	0	13	6	3	2	3	5	6
Mcl1	10181	11289	10969	10483	10868	11467	11170	10950
Mcm10	793	902	821	859	957	864	741	830
Mcm2	1360	1476	1393	1312	1452	1360	1189	1337
Mcm3	1194	1202	1119	1267	1205	1175	1062	1134
Mcm3ap	1344	1454	1389	1235	1265	1353	1286	1244
Mcm4	1163	1126	993	929	1100	1073	1056	922
Mcm5	1662	1810	1711	1795	1899	1790	1599	1544
Mcm6	2297	2590	2422	2264	2693	2518	2138	2107
Mcm7	1811	1885	1736	1662	1738	1720	1423	1590
Mcm8	126	142	128	127	235	191	171	154
Mcm9	513	510	553	505	447	567	467	456
Mcmbp	2172	2383	2128	2223	2308	2204	2255	2183

Transcriptome sequencing yielded total genetic results for the MOD and APS groups, with a total of 15,936 variables

Continued from above

Mcmdc2	2	2	4	0	0	0	0	1
Mcoln1	1157	1059	1062	1073	1310	1185	1139	1047
Mcoln2	671	721	819	731	750	734	729	688
Mcoln3	47	38	66	63	53	35	63	70
Mcph1	317	271	249	308	258	308	288	267
Mcpt1	0	3	0	1	0	0	0	1
Mcpt2	0	0	0	0	0	0	0	6
Mcpt4	0	1	0	0	2	0	0	0
Mcpt8	0	0	0	1	0	0	0	0
Mcrip1	284	301	270	283	324	292	272	271
Mcrip2	859	838	814	897	824	739	720	684
Mcrs1	2631	2840	2633	2662	2425	2515	2415	2279
Mctp1	32	30	31	33	53	77	48	28
Mctp2	135	132	148	185	134	157	128	144
Mcts1	699	732	795	786	772	854	835	765
Mcts2	294	269	271	281	208	246	262	312
Mcu	8622	9161	8987	8445	8200	8504	8321	8417
Mcub	34	22	17	24	26	24	38	6
Mcur1	4127	4176	4028	4233	3774	4082	3928	3605
Mdc1	690	775	708	638	670	700	725	645
Mdfi	63	50	29	44	58	57	27	41
Mdfic	202	230	204	236	219	209	221	206
Mdga1	6	3	8	3	5	21	11	6
Mdga2	0	4	0	0	0	0	0	1
Mdh1	47932	48403	45930	46248	45611	49085	48557	45343
Mdh1b	4	0	0	0	0	0	0	0
Mdh2	68739	70736	69697	68650	70178	71557	67217	68591
Mdk	56	69	72	97	59	82	41	29
Mdm1	91	101	87	117	103	89	104	60
Mdm2	1503	1644	1619	1641	1738	1784	1706	1749
Mdm4	1789	2218	2072	2102	2172	2148	1939	1868
Mdn1	656	792	645	653	882	774	683	615
Mdp1	2362	2501	2382	1947	2224	2204	2265	2074
Me1	4234	2846	3903	5567	3370	3191	2792	2747
Me2	16315	18978	16214	14824	17694	19757	19122	16723
Me3	11	4	8	16	5	11	11	8
Mea1	1723	1849	1828	1769	1795	2120	1746	1847
Meaf6	275	303	331	385	290	267	245	249
Meak7	574	556	528	571	473	484	514	472
Mecom	554	576	580	774	548	503	560	541
Mecp2	964	992	1065	1074	836	825	887	884
Mecr	1340	1390	1462	1349	1418	1397	1235	1267
Med1	2123	2183	2289	2144	1935	2178	1990	2039
Med10	513	485	566	527	520	444	537	403
Med11	594	654	688	601	629	594	696	609
Med12	1206	1354	1391	1260	1317	1307	1252	1212
Med12l	17	14	42	27	27	13	24	27
Med13	3906	4438	4163	3824	4138	4412	4377	3824
Med13l	859	890	1018	998	996	975	1014	811
Med14	2014	2444	2194	2226	2197	2131	1944	1935
Med15	2890	3101	2830	2902	2773	2682	2471	2733
Med16	1225	1235	1275	1170	1173	1124	1008	987
Med17	1042	1273	1164	1055	1222	1264	1033	1111
Med18	65	111	93	75	79	74	60	77
Med19	973	988	984	931	1074	1125	1030	944
Med20	602	566	536	490	646	691	579	547
Med21	664	567	597	684	708	665	615	654
Med22	486	535	486	544	551	432	475	421

Transcriptome sequencing yielded total genetic results for the MOD and APS groups, with a total of 15,936 variables

Continued from above

Med23	1242	1365	1219	1301	1369	1287	1262	1283
Med24	1575	1534	1541	1425	1532	1551	1377	1523
Med25	3638	4100	3668	3927	3581	3623	3663	3604
Med26	366	435	423	369	472	471	421	440
Med27	595	569	611	664	533	527	550	541
Med28	1744	1749	1637	1640	1693	1747	1607	1623
Med29	481	472	435	440	477	463	456	457
Med30	620	466	572	620	604	483	494	520
Med31	230	157	182	153	243	231	180	188
Med4	323	370	375	407	409	399	304	385
Med6	539	524	580	602	571	636	622	515
Med7	346	364	445	360	382	428	345	402
Med8	1273	1417	1353	1317	1431	1378	1401	1308
Med9	841	930	885	836	799	756	798	809
Medag	52	48	75	65	56	55	43	58
Mef2a	1260	1330	1304	1308	1204	1283	1289	1164
Mef2c	140	144	176	157	100	111	149	119
Mef2d	2751	2846	2848	2703	2658	2814	2874	2645
Mefv	48	66	43	76	31	43	51	46
Megf10	5	7	1	4	6	1	2	7
Megf11	6	3	4	5	9	7	8	9
Megf6	42	57	42	33	52	48	53	36
Megf8	1115	1173	1262	1188	1180	1202	1225	1198
Megf9	63	105	83	83	71	74	65	82
Mei1	0	1	0	0	0	0	0	2
Mei4	1	3	1	0	0	1	0	1
Meikin	0	0	0	0	1	0	0	0
Meiob	0	3	1	0	0	5	1	7
Meioc	1	0	0	0	0	6	0	0
Meiosin	0	2	1	0	0	0	0	0
Meis1	55	51	78	42	47	69	89	75
Meis2	202	265	235	202	249	250	220	207
Meis3	63	79	94	89	64	69	80	81
Melk	623	722	736	656	700	703	582	670
Meltf	0	0	0	0	5	1	1	3
Memo1	1052	1045	992	1050	1064	1132	884	987
Men1	1144	1101	1212	1187	1238	1193	1122	1202
Meox1	72	127	74	88	101	101	83	90
Meox2	7	1	6	0	8	6	2	4
Mep1a	1157	1341	1136	911	1382	1511	1589	1430
Mep1b	35937	39699	34151	32749	35933	38974	41473	37210
Mepce	1417	1611	1565	1397	1432	1418	1374	1344
Mertk	4074	5124	4249	3872	5358	5634	6173	5195
Mesd	1068	1050	1046	1067	1135	1148	1004	1048
Mesp1	18	13	10	10	23	6	8	10
Mesp2	3	4	6	8	6	18	2	8
Mest	60	91	67	78	67	80	64	73
Met	2539	2488	2427	2383	2687	2724	2505	2370
Metap1	2612	2736	2571	2316	2913	2956	2690	2612
Metap1d	241	398	274	344	382	393	296	316
Metap2	3540	3921	3656	3544	4237	3940	3893	3841
Metrn	98	162	153	174	170	114	143	158
Metrnl	3004	3083	2836	2776	2978	3157	3023	2780
Mettl1	259	309	374	287	357	301	248	270
Mettl14	640	661	653	676	609	680	654	606
Mettl15	139	135	138	136	144	136	124	151
Mettl16	409	468	518	412	564	528	445	516
Mettl17	357	398	465	393	423	444	411	388

Transcriptome sequencing yielded total genetic results for the MOD and APS groups, with a total of 15,936 variables

Continued from above

Mettl18	83	86	94	82	106	89	69	112
Mettl2	391	434	420	394	500	466	414	429
Mettl21a	162	149	179	142	174	174	170	163
Mettl22	92	116	109	142	107	102	91	74
Mettl23	530	611	611	515	605	640	480	561
Mettl24	32	18	29	7	24	10	8	16
Mettl25	141	155	141	127	141	121	125	129
Mettl26	1082	1000	935	1046	945	1063	886	924
Mettl27	271	278	269	225	208	204	201	197
Mettl3	442	476	372	429	525	447	406	486
Mettl4	401	439	443	364	484	445	452	421
Mettl5	221	269	239	294	226	258	249	242
Mettl6	463	503	482	479	457	524	453	453
Mettl7a1	745	843	866	806	967	875	823	749
Mettl7a2	0	2	1	1	0	1	2	0
Mettl7a3	0	0	0	0	0	0	1	0
Mettl7b	592	729	659	592	713	732	687	618
Mettl8	220	270	271	205	307	261	224	220
Mettl9	3577	3808	3460	3203	3761	4014	3318	3608
Mex3a	63	51	45	40	27	69	72	31
Mex3b	36	40	25	34	19	46	27	34
Mex3c	2551	3120	2816	2662	3120	2929	2702	2757
Mex3d	193	275	323	256	265	204	277	190
Mfap1a	386	414	364	381	397	455	433	387
Mfap1b	415	199	167	161	189	210	146	148
Mfap2	52	26	52	40	36	34	39	44
Mfap3	1633	1722	1575	1548	1604	1769	1698	1533
Mfap3l	40	65	39	60	48	88	48	52
Mfap4	339	392	399	383	348	382	344	266
Mfap5	25	21	48	34	33	19	36	29
Mff	2451	2604	2596	2688	2573	2624	2515	2361
Mfge8	2058	1791	1926	2156	2083	2004	1830	1908
Mfhas1	146	160	130	156	189	169	223	226
Mfn1	1950	1980	1847	1916	1905	1942	1855	1939
Mfn2	7021	7937	7006	6944	6940	7281	7367	6764
Mfng	51	58	56	78	76	70	49	60
Mfsd1	2065	2125	1950	2026	1976	1978	1997	1871
Mfsd10	544	529	540	556	619	491	457	488
Mfsd11	1784	1880	1817	1755	1987	1841	1931	1884
Mfsd12	725	614	654	621	666	603	655	704
Mfsd13a	489	459	495	459	485	415	361	416
Mfsd13b	12	0	0	13	4	8	0	4
Mfsd14a	1513	1562	1476	1552	1831	1630	1508	1488
Mfsd14b	3794	3772	3285	3465	3793	3867	3737	3712
Mfsd2a	1977	2285	2021	1813	2503	2583	2453	2571
Mfsd2b	45	58	77	47	70	42	59	80
Mfsd3	234	204	189	182	165	200	177	145
Mfsd4a	1592	1848	1739	1686	1883	2035	2065	1896
Mfsd4b1	22	9	12	11	28	3	7	29
Mfsd4b4	131	153	126	95	102	69	141	93
Mfsd4b5	385	528	418	363	563	540	574	536
Mfsd5	959	1041	1014	859	961	936	952	1021
Mfsd6	1657	1694	1603	1675	1877	1941	1782	1707
Mfsd6l	952	1062	1006	926	819	816	911	909
Mfsd7a	128	122	160	108	140	129	111	141
Mfsd8	594	633	593	542	646	715	685	564
Mfsd9	1308	1456	1310	1225	1165	1299	1316	1106
Mga	789	968	848	937	1194	1198	1076	948

Transcriptome sequencing yielded total genetic results for the MOD and APS groups, with a total of 15,936 variables

Continued from above

Mgam	79743	94758	81155	77055	90290	100087	116389	102600
Mgarp	9	11	1	2	3	5	1	20
Mgat1	3219	3426	3497	3371	3310	3283	3182	3052
Mgat2	3428	3667	3536	3355	4425	4029	3822	3828
Mgat3	5766	5891	6080	5937	6683	6991	6747	6205
Mgat4a	12410	14027	13340	12879	14654	15136	14458	13754
Mgat4b	4149	4277	4253	4150	4274	4121	3610	4031
Mgat4c	2032	1375	1830	2568	1608	1531	1505	1603
Mgat5	1609	2041	1748	1508	1862	1893	1868	1795
Mgat5b	2	0	0	0	0	4	3	0
Mgl2	71	101	64	71	93	147	116	108
Mgll	558	709	757	691	669	595	657	609
Mgme1	250	299	270	296	356	345	360	362
Mgmt	66	28	52	59	65	40	54	55
Mgp	481	508	441	451	450	526	479	491
Mgrn1	4842	4658	4372	4606	4318	4300	4349	4154
Mgst1	6105	7035	6674	6084	6816	6962	6362	6621
Mgst2	4288	4912	4738	4220	6031	5737	5188	5819
Mgst3	17025	17642	17873	17192	18059	18184	16497	16732
Mia	5	8	8	30	7	1	10	13
Mia2	8616	9387	8263	8168	9270	9660	9711	8516
Mia3	3893	4313	3819	3963	4462	4405	4424	3860
Mib1	1017	1071	1139	1152	1319	1229	1280	1175
Mib2	1473	1533	1299	1440	1492	1488	1621	1414
Mical1	9681	10941	9064	7747	8560	8799	9267	7972
Mical2	1264	1465	1218	1144	1675	1658	1453	1474
Mical3	642	595	677	729	603	598	557	540
Micalcl	289	321	308	283	421	397	377	346
Micall1	1433	1360	1356	1333	1305	1433	1315	1137
Micall2	1895	1964	1943	1911	2059	2099	1981	1983
Micos10	4389	4529	4681	4706	4513	4369	4066	4187
Micos13	2570	2440	2549	2608	2338	2553	2162	2377
Micu1	8400	9235	8138	8274	7795	7872	7983	7721
Micu2	4981	5683	4847	5232	4891	5254	5287	4968
Micu3	50	38	52	50	39	45	40	40
Mid1	602	574	304	317	357	365	409	459
Mid1ip1	1845	1941	1838	1867	1876	2092	1801	1808
Mid2	956	1079	1048	1122	1091	991	1012	887
Mideas	2242	2318	2094	2013	2134	2233	2052	1877
Midn	5997	6623	7315	6600	6110	6296	6699	6112
Mief1	1990	2237	2133	2225	2132	2292	2174	2078
Mief2	640	550	502	514	507	597	510	610
Mien1	1207	1371	1319	1286	1379	1489	1369	1482
Mier1	2189	2615	2508	2507	2696	2849	2605	2456
Mier2	1132	1161	1154	1185	1111	1077	1074	1167
Mier3	1090	1075	885	1138	1005	1119	1145	946
Mif	1886	1988	2048	1911	2515	1974	1853	2123
Mif4gd	1320	1253	1276	1341	1356	1228	1160	1173
Miga1	502	406	459	684	377	396	382	440
Miga2	5317	4700	4852	5651	4639	4625	4512	4405
Miip	403	372	401	383	373	367	338	389
Mill1	1	0	0	0	5	0	0	4
Mill2	51	40	41	76	54	73	35	59
Milr1	88	67	57	72	61	71	85	56
Minar1	4	0	0	1	1	0	1	0
Minar2	12	1	6	7	13	8	15	7
Mindy1	1916	2055	1970	1881	1906	1900	1825	1906
Mindy2	1239	1132	1347	1308	1109	1314	1161	1125

Transcriptome sequencing yielded total genetic results for the MOD and APS groups, with a total of 15,936 variables

Continued from above

Mindy3	1261	1441	1225	1178	1381	1319	1330	1373
Mindy4	4	10	15	19	18	22	23	23
Mink1	2774	2729	3072	2826	2778	2730	2727	2555
Minpp1	3683	4170	3850	3544	4012	3744	3529	3552
Mios	397	356	339	383	407	317	400	347
Mipep	1065	1163	1159	1132	1256	1198	1030	1075
Mipol1	22	13	36	17	29	32	37	5
Mis12	591	599	654	687	778	716	637	645
Mis18a	482	441	503	515	485	452	541	432
Mis18bp1	244	231	184	181	249	260	266	227
Misp	25161	26774	24500	22774	25546	26655	24845	22841
Misp3	53	61	60	72	59	73	63	70
Mitd1	413	477	469	392	435	496	394	457
Mitf	16	59	40	27	35	27	18	23
Mki67	3354	3885	3772	3598	4089	4139	3872	3421
Mkks	1369	1536	1517	1460	1763	1804	1774	1655
Mkln1	2200	2417	2135	2128	2358	2658	2545	2335
Mknk1	834	822	878	799	859	870	807	815
Mknk2	4591	4762	5212	4672	5189	5227	4769	4878
Mkrn1	7660	8105	7570	7193	8396	8062	7750	7522
Mkrn2	585	596	580	568	660	630	584	547
Mkrn2os	3942	4086	3916	3672	4187	4252	4085	3848
Mkrn3	4	2	2	8	3	2	6	4
Mks1	82	84	122	55	101	50	72	87
Mkx	1	3	2	1	2	1	7	0
Mlana	10	4	5	0	8	5	4	3
Mlc1	0	0	0	12	0	1	0	1
Mlec	23369	26331	25588	24415	28874	29741	26023	25958
Mlf1	1	1	2	0	0	0	0	1
Mlf2	13418	13606	12307	12800	13169	13688	13544	12847
Mlh1	536	616	581	545	684	580	493	586
Mlh3	153	129	172	179	153	160	141	137
Mlip	0	0	1	0	1	4	1	1
Mlkl	1841	2255	1918	1857	1885	1837	1835	1671
Mllt1	1565	1685	1550	1519	1669	1580	1568	1414
Mllt10	1285	1406	1496	1385	1451	1355	1417	1281
Mllt11	408	468	415	385	532	462	397	410
Mllt3	841	851	874	859	940	849	924	771
Mllt6	1608	1607	1592	1851	1583	1439	1391	1446
Mlph	1509	1715	1599	1591	1575	1642	1504	1500
Mlst8	1064	1133	1115	1019	1017	1113	991	1083
Mlx	6233	6192	5985	6100	5508	6055	5675	5744
Mlxip	3005	3411	3512	3245	3441	3357	3273	3027
Mlxipl	2761	2934	2695	2595	3225	3278	3208	2799
Mlycd	1723	1694	1561	1600	1826	1789	1581	1608
Mmaa	740	741	775	782	889	855	796	842
Mmab	516	558	625	638	509	508	518	508
Mmachc	255	301	264	259	253	318	242	290
Mmadhc	2124	2187	2283	2014	2225	2186	2318	2208
Mmd	1048	1258	1168	1046	1363	1553	1164	1276
Mmd2	2	1	4	1	14	15	1	5
Mme	25762	28023	22374	22202	25501	29404	31837	25074
Mmel1	0	1	1	1	2	1	0	0
Mmgt1	1309	1387	1405	1367	1319	1572	1484	1436
Mmgt2	529	668	701	637	711	705	637	673
Mmp10	1407	1450	1583	1422	1286	1253	1136	1165
Mmp11	31	42	38	37	37	25	38	27
Mmp12	18	22	34	31	63	57	59	41

Transcriptome sequencing yielded total genetic results for the MOD and APS groups, with a total of 15,936 variables

Continued from above

Mmp13	581	703	638	558	411	419	466	406
Mmp14	2467	2608	2473	2456	2293	2289	2084	1960
Mmp15	971	925	954	1105	851	1001	945	828
Mmp16	9	17	8	9	3	7	12	8
Mmp17	102	89	70	70	89	75	67	49
Mmp19	91	91	139	81	131	88	90	96
Mmp2	1136	1246	1343	1189	1267	1110	964	846
Mmp20	14	18	10	7	3	14	7	12
Mmp23	145	72	122	117	105	117	102	103
Mmp24	58	67	61	45	86	93	85	72
Mmp25	52	40	20	43	31	30	36	25
Mmp27	1	0	0	0	0	1	0	1
Mmp28	1159	1115	1118	968	1352	1194	974	1157
Mmp3	16	31	45	37	43	44	32	33
Mmp7	2948	2840	3090	3397	2797	2658	3223	3189
Mmp8	0	2	1	0	2	0	0	4
Mmp9	747	797	981	984	840	701	696	619
Mmrn1	449	547	489	406	404	441	471	428
Mmrn2	415	432	426	395	358	387	330	364
Mms19	782	893	990	1025	922	823	862	836
Mms22l	288	418	396	381	372	379	323	329
Mmut	1888	2046	2070	1916	1953	1913	2123	1875
Mn1	20	23	46	37	34	20	36	26
Mnat1	381	385	375	370	323	401	347	342
Mnd1	47	38	60	28	56	42	46	43
Mndal	227	238	218	246	168	162	190	139
Mns1	28	43	30	26	40	56	45	25
Mnt	1084	1141	1205	1044	1345	1322	1213	1249
Mnx1	29	38	32	23	57	45	48	50
Mob1a	1448	1770	1619	1545	1783	1676	1817	1556
Mob1b	1249	1373	1324	1392	1619	1700	1437	1568
Mob2	1409	1465	1452	1362	1536	1533	1311	1393
Mob3a	869	875	1092	972	1042	1003	950	875
Mob3b	1289	1614	1274	1276	1475	1408	1346	1286
Mob3c	1255	1528	1498	1593	1305	1416	1420	1373
Mob4	1972	2147	1959	2159	2150	2359	2343	1960
Mobp	1	0	0	0	0	0	0	0
Mocos	2912	3196	2822	2836	3180	3170	2835	2770
Mocs1	4004	4399	4153	3768	3826	4072	3517	3713
Mocs2	5586	5986	5660	4891	5792	6401	6093	5204
Mocs3	509	514	497	464	660	466	418	507
Mog	1	0	0	0	0	0	0	0
Mogat1	0	6	5	5	0	0	0	0
Mogat2	19969	21989	19461	18431	21467	22079	21957	20919
Mogs	1808	1955	1781	1703	2356	2104	1930	1884
Mok	5	11	15	6	8	11	16	6
Mon1a	3063	3083	2888	2783	2987	3169	3007	3012
Mon1b	745	974	842	905	1027	998	900	874
Mon2	4215	4456	4169	3918	4515	4649	4561	4189
Morc1	14	17	10	11	12	31	30	34
Morc2a	854	954	904	861	1095	1045	851	923
Morc3	1475	1905	1715	1580	1767	1781	1748	1583
Morc4	148	175	152	135	238	207	210	221
Morf4l1	3756	3911	3799	3820	3843	4070	3890	3843
Morf4l1b	311	270	249	273	264	282	294	247
Morf4l2	7786	8082	7772	7638	8962	9364	8797	8418
Morn1	84	154	151	136	85	91	75	117
Morn2	87	118	76	82	81	74	99	108

Transcriptome sequencing yielded total genetic results for the MOD and APS groups, with a total of 15,936 variables

Continued from above

Morn3	0	0	1	0	0	1	0	0
Morn4	12	5	13	4	12	12	16	7
Mos	0	0	0	1	0	0	0	1
Mosmo	200	265	207	223	316	232	211	223
Mospd1	515	506	448	497	500	492	484	455
Mospd2	725	761	769	732	836	816	735	767
Mospd3	972	984	855	1014	930	833	826	844
Mov10	6962	8138	7060	5842	7488	8151	7436	7037
Moxd1	207	198	215	217	233	172	180	197
Moxd2	0	0	2	0	0	0	0	0
Mpc1	1704	1516	1519	1759	1690	1651	1565	1503
Mpc2	3814	3588	3645	3832	3433	3785	3529	3527
Mpdu1	2963	2975	2605	2967	2944	2924	2511	2804
Mpdz	89	78	101	109	116	88	110	80
Mpeg1	2560	2717	2585	2677	2401	2245	2230	2272
Mpg	290	249	325	287	273	229	251	270
Mphosph1	400	459	445	360	484	513	489	408
Mphosph6	347	364	332	294	410	394	382	349
Mphosph8	340	395	357	304	441	349	352	345
Mphosph9	185	248	182	255	223	270	205	235
Mpi	4247	4578	4495	4545	4267	4410	4343	4257
Mpig6b	9	17	10	15	21	4	9	14
Mpl	0	0	0	0	0	1	0	0
Mplkip	555	607	722	656	622	645	720	555
Mpnd	4130	4251	4044	3927	3739	3799	3773	3719
Mpo	1	0	0	0	1	0	0	0
Mpp1	15420	15846	14738	14644	14718	15750	15668	14769
Mpp2	21	45	26	33	22	45	35	38
Mpp3	57	56	45	72	89	83	69	72
Mpp4	34	51	57	38	43	44	45	28
Mpp5	2910	3239	2728	2843	3509	3766	4236	3601
Mpp6	538	650	635	515	739	722	667	643
Mpp7	451	394	453	458	477	421	413	389
Mppe1	1559	1622	1556	1689	1517	1555	1583	1540
Mpped2	10	13	6	13	4	10	8	16
Mprip	3330	3444	3483	3345	3496	3496	3160	3234
Mpst	1217	1095	1070	1163	1302	1109	1040	1135
Mptx1	4	2	5	2	0	2	1	3
Mptx2	2653	2335	2532	3229	2035	2177	3080	3331
Mpv17	543	546	502	574	525	555	529	536
Mpv17l	204	128	132	157	157	176	113	177
Mpv17l2	1219	1147	1242	1084	1100	1084	1024	1138
Mpz	4	16	16	6	7	8	15	15
Mpzl1	1013	1248	1085	1136	1216	1134	1172	1103
Mpzl2	1645	1774	1849	1715	1878	1963	1674	1767
Mpzl3	894	890	909	902	997	982	909	903
Mr1	76	45	63	72	61	43	56	33
Mrap	1	5	2	1	0	1	12	9
Mrap2	6	0	1	0	2	0	4	0
Mras	72	79	81	87	101	44	94	96
Mrc1	249	335	318	328	386	413	339	348
Mrc2	174	169	192	189	194	195	158	167
Mre11a	400	574	484	456	471	532	502	390
Mreg	329	390	248	282	304	319	239	261
Mrfap1	4898	4815	4891	4587	5083	5190	4811	4964
Mrgbp	307	298	274	358	355	348	250	201
Mrgpra9	0	0	0	0	0	0	1	0
Mrgpre	60	19	40	34	34	38	43	5

Transcriptome sequencing yielded total genetic results for the MOD and APS groups, with a total of 15,936 variables

Continued from above

Mrgprf	62	54	75	64	65	61	66	100
Mrgprg	0	0	0	0	0	0	0	1
Mrgprh	2	0	0	0	0	0	0	0
Mrgprx2	1	0	0	0	0	0	0	0
Mri1	503	503	543	473	562	411	390	461
Mrln	0	1	2	0	1	2	0	1
Mrm1	394	444	473	443	589	525	505	498
Mrm2	231	292	264	264	251	284	255	322
Mrm3	256	210	275	296	285	255	218	285
Mrnip	67	56	62	52	73	53	42	34
Mro	156	213	122	147	220	227	217	160
Mroh1	2116	2081	2036	2133	2117	2135	2057	1937
Mroh2a	44	55	47	56	26	43	33	35
Mroh3	62	70	71	92	62	58	66	62
Mroh4	1	4	0	0	0	0	0	0
Mroh5	0	0	1	0	0	0	0	0
Mroh6	92	137	161	131	249	245	199	206
Mroh7	97	124	144	113	113	107	169	132
Mroh8	44	89	52	47	52	62	59	58
Mrpl1	846	937	871	778	887	876	855	917
Mrpl10	2250	2139	2270	2084	1959	2072	2087	2093
Mrpl11	918	988	989	981	1056	926	882	1075
Mrpl12	7851	7940	7887	7946	7857	7839	7172	7439
Mrpl13	1125	1280	1147	1232	1345	1268	1222	1063
Mrpl14	1134	1075	1238	1208	1141	1198	1093	1103
Mrpl15	3029	2960	3151	2940	3267	3020	3052	2887
Mrpl16	1181	1184	1286	1222	1193	1210	1176	1099
Mrpl17	1459	1480	1366	1486	1571	1581	1369	1277
Mrpl18	2622	2535	2599	2626	2873	2721	2564	2489
Mrpl19	905	1053	929	873	1014	901	876	906
Mrpl2	1707	1765	1829	1759	1792	1695	1478	1442
Mrpl20	2232	2040	2249	2102	2275	2260	2096	2090
Mrpl21	1063	1021	1118	1130	1208	1109	961	1027
Mrpl22	647	661	637	631	693	719	578	637
Mrpl23	881	928	941	900	891	838	739	734
Mrpl24	2515	2680	2532	2599	2759	2761	2523	2515
Mrpl27	1728	1732	1740	1737	1638	1785	1650	1598
Mrpl28	1645	1718	1800	1640	1719	1664	1475	1607
Mrpl3	1935	1983	2071	2060	2137	2024	1697	2010
Mrpl30	2575	2655	2834	2701	2920	2814	2358	2534
Mrpl32	749	739	802	811	799	802	700	759
Mrpl33	913	1049	944	938	1096	1078	911	932
Mrpl34	1893	1852	1857	2114	1772	1896	1895	1998
Mrpl35	1477	1364	1446	1462	1484	1470	1342	1319
Mrpl36	1400	1600	1572	1484	1563	1517	1428	1391
Mrpl37	1746	1652	1683	1671	1771	1727	1483	1643
Mrpl38	1817	1879	1760	1714	1870	1819	1573	1665
Mrpl39	1240	1260	1284	1387	1294	1284	1249	1181
Mrpl4	2159	2148	2176	2044	2108	2003	1815	2003
Mrpl40	1089	986	1182	1139	1044	1161	991	1006
Mrpl41	877	811	946	907	914	1017	857	891
Mrpl42	1797	1851	1931	2000	2073	1851	1964	1835
Mrpl43	2435	2310	2449	2373	2088	2281	2250	2074
Mrpl44	890	771	849	778	842	829	698	699
Mrpl45	1724	1776	1925	1758	1863	1822	1742	1750
Mrpl46	1153	1219	1123	1272	1255	1146	1075	1105
Mrpl47	792	751	675	743	789	753	787	715
Mrpl48	1223	1401	1155	1279	1450	1289	1163	1136

Transcriptome sequencing yielded total genetic results for the MOD and APS groups, with a total of 15,936 variables

Continued from above

Mrpl49	1300	1287	1357	1419	1394	1320	1349	1213
Mrpl50	1308	1368	1311	1207	1390	1476	1398	1426
Mrpl51	2382	2341	2394	2619	2552	2520	2331	2299
Mrpl52	1257	1372	1348	1307	1377	1237	1110	1212
Mrpl53	1051	1171	1178	1049	1137	1206	1080	1142
Mrpl54	2160	2124	2197	2144	2059	2180	2045	2048
Mrpl55	1281	1345	1247	1316	1334	1229	1208	1137
Mrpl57	1308	1326	1153	1122	1275	1070	1058	1165
Mrpl58	1237	1312	1130	1216	1220	1187	1080	1105
Mrpl9	2588	2679	2674	2851	2800	2722	2542	2567
Mrps10	840	953	888	897	865	845	783	737
Mrps11	781	883	666	770	762	699	700	713
Mrps12	942	940	1039	836	1056	1077	964	1003
Mrps14	1363	1321	1368	1364	1441	1344	1334	1247
Mrps15	1868	2066	1949	1892	2017	1890	1813	1733
Mrps16	769	913	922	888	851	860	854	801
Mrps17	831	772	837	760	837	793	693	819
Mrps18a	1052	962	919	883	1043	949	929	891
Mrps18b	915	943	871	835	1042	962	799	878
Mrps18c	996	1022	1006	1023	1021	1102	1159	995
Mrps2	1376	1342	1243	1242	1422	1376	1313	1294
Mrps21	1394	1409	1507	1473	1386	1424	1482	1437
Mrps22	902	967	917	910	1013	1132	879	913
Mrps23	1996	1912	1798	1880	2004	1935	1842	1924
Mrps24	1929	1982	2080	1906	2014	1978	1724	1832
Mrps25	748	790	820	804	770	755	775	722
Mrps26	1048	1059	1117	1018	959	979	938	985
Mrps27	1322	1267	1375	1301	1340	1174	1148	1131
Mrps28	747	855	808	737	933	904	785	838
Mrps30	1420	1465	1425	1403	1526	1497	1228	1341
Mrps31	1928	2072	2026	1876	2081	2139	1962	1893
Mrps33	718	761	855	780	799	771	726	809
Mrps34	2355	2179	2300	2143	2000	2049	1817	1850
Mrps35	1341	1454	1445	1517	1496	1557	1287	1253
Mrps36	2076	1968	1993	2066	1934	2176	1929	2011
Mrps5	1832	1935	2019	2016	2053	1889	1724	1763
Mrps6	460	396	418	344	468	446	366	409
Mrps7	1799	1750	1786	1735	1976	1891	1794	1671
Mrps9	1477	1517	1610	1609	1522	1470	1271	1288
Mrrf	534	539	535	600	591	618	560	589
Mrs2	3766	3927	3830	3702	4003	3914	3621	3662
Mrtfa	976	930	849	852	929	924	808	791
Mrtfb	1213	1470	1341	1425	1343	1395	1340	1201
Mrto4	602	692	567	482	604	599	495	542
Mrto4-ps1	34	60	47	17	40	27	34	40
Mrto4-ps2	0	6	4	11	8	9	18	8
Mrvi1	363	401	369	383	367	361	333	396
Ms4a1	8	4	4	21	1	1	0	0
Ms4a10	7197	7695	7620	7350	6550	7303	7381	6767
Ms4a12	1740	1855	1672	1607	1714	2019	1696	1819
Ms4a14	20	21	20	14	13	20	15	12
Ms4a15	22	33	22	16	8	20	23	13
Ms4a18	4483	5231	4925	4549	4666	4907	4073	3913
Ms4a4a	93	106	106	123	121	127	100	132
Ms4a4b	63	100	91	55	70	86	49	44
Ms4a4c	61	73	68	62	41	43	30	48
Ms4a4d	393	428	282	383	296	298	381	301
Ms4a5	0	3	4	4	4	1	0	0

Transcriptome sequencing yielded total genetic results for the MOD and APS groups, with a total of 15,936 variables

Continued from above

Ms4a6b	281	297	284	257	243	281	205	234
Ms4a6c	415	429	329	347	413	365	408	405
Ms4a6d	270	299	361	282	226	242	284	264
Ms4a7	275	318	264	305	217	266	247	232
Ms4a8a	6670	6806	6555	6870	6507	6645	6758	6776
Msantd1	16	6	13	3	21	31	20	11
Msantd2	190	234	228	305	272	258	241	243
Msantd3	56	55	47	34	58	57	42	32
Msantd4	1282	1368	1283	1306	1400	1341	1522	1255
Msc	82	94	93	73	85	80	71	83
Msh2	887	922	876	973	869	1073	803	950
Msh3	349	406	371	364	393	433	358	298
Msh4	1	3	2	2	5	0	3	1
Msh5	31	46	41	10	52	27	22	37
Msh6	742	872	859	787	819	821	926	739
Msi1	164	244	241	189	157	138	151	195
Msi2	986	995	1000	1069	998	985	848	834
Msl1	2832	2892	2842	2821	3124	3019	2738	2660
Msl2	1575	1879	1777	1760	1938	1934	1750	1712
Msl3	1376	1678	1619	1580	1750	1848	1646	1480
Msl3l2	33	26	28	18	31	19	23	35
Msln	37	22	58	35	41	54	86	87
Mslnl	1	0	0	0	1	0	7	1
Msmo1	3879	3970	4200	4631	4282	4361	4430	4364
Msmp	27	35	31	26	25	27	15	18
Msn	1278	1425	1406	1377	1207	1155	1168	1154
Msr1	415	370	388	376	333	386	411	427
Msra	2988	2901	3003	2759	2794	3211	2788	2575
Msrb1	1261	1332	1204	1315	1410	1482	1295	1326
Msrb2	8	38	27	18	22	24	27	17
Msrb3	291	353	361	307	282	274	288	276
Mss51	28	19	16	26	13	11	24	17
Mst1	14	6	1	15	5	5	1	10
Mst1r	2009	2153	2264	2034	2365	2353	2107	2093
Mstn	8	0	0	0	4	0	0	0
Msto1	417	418	454	400	385	351	362	379
Msx1	10	7	13	17	6	6	8	5
Msx3	0	6	0	0	0	0	0	0
Mt1	2815	3052	3395	3364	4159	3928	3430	3855
Mt2	823	898	1125	968	1342	1168	1006	1130
Mt3	7	8	10	10	18	12	6	12
Mt4	1	0	1	8	1	0	0	1
Mta1	901	1059	979	896	921	936	816	938
Mta2	5283	5446	5264	5305	5394	5374	5058	4910
Mta3	1676	1848	1869	1610	1476	1594	1503	1529
Mtap	514	534	466	392	545	387	445	498
Mtarc1	15	43	40	25	31	58	31	35
Mtarc2	30683	32617	30994	30162	30810	32202	30881	29233
Mtbp	232	268	281	240	225	244	230	235
Mtch1	5239	5084	5206	5164	4852	4954	4523	4826
Mtch2	14548	15619	15045	14594	15153	15078	14586	13994
Mtcl1	43	68	60	56	38	57	72	44
Mtcp1	55	91	80	91	74	100	68	58
Mtdh	3709	3861	3388	3425	4020	4080	4005	3640
Mterf1a	48	50	80	42	52	58	60	64
Mterf1b	8	20	17	20	37	27	15	31
Mterf2	512	614	522	604	525	640	556	560
Mterf3	584	603	564	621	626	483	507	585

Transcriptome sequencing yielded total genetic results for the MOD and APS groups, with a total of 15,936 variables

Continued from above

Mterf4	225	253	208	287	313	205	298	229
Mtf1	2221	2612	2346	2136	2297	2413	2392	2242
Mtf2	742	842	695	669	832	811	848	649
Mtfmt	502	572	481	503	471	544	514	484
Mtfp1	1241	1230	1314	1130	1211	1149	1063	997
Mtfr1	1290	1530	1444	1322	1412	1680	1437	1462
Mtfr1l	2771	2780	2843	2718	2499	2757	2594	2580
Mtfr2	120	111	86	101	89	89	101	97
Mtg1	327	439	348	335	351	345	265	393
Mtg2	429	358	423	357	361	328	304	344
Mthfd1	2388	2722	2532	2579	2644	2514	2169	2262
Mthfd1l	217	249	264	198	224	220	182	250
Mthfd2	565	642	560	499	537	575	548	577
Mthfd2l	231	286	262	254	279	196	243	246
Mthfr	315	363	251	342	325	284	256	271
Mthfs	73	84	77	77	89	71	55	80
Mthfsd	440	461	420	463	497	514	470	421
Mthfsl	278	358	342	309	318	351	354	319
Mtif2	1090	1245	1137	1267	1289	1291	1226	1179
Mtif3	1420	1223	1435	1405	1348	1497	1340	1248
Mtln	410	559	482	519	585	550	525	489
Mtm1	2125	2134	1937	2198	1897	2161	2443	2230
Mtmr1	1220	1306	1246	1292	1336	1376	1348	1260
Mtmr10	469	477	511	471	472	429	465	483
Mtmr11	575	682	620	407	690	756	725	595
Mtmr12	1860	1935	1967	2019	1866	2057	1965	1804
Mtmr14	1323	1392	1477	1214	1370	1285	1403	1298
Mtmr2	1889	1826	1840	1786	1896	1849	1584	1655
Mtmr3	2538	2749	2388	2493	2451	2705	2536	2396
Mtmr4	7840	8030	7469	7124	8253	8119	8390	7734
Mtmr6	2196	2097	2081	2272	2153	2308	2332	2210
Mtmr7	885	995	808	932	841	870	1064	812
Mtmr9	1663	1853	1811	1613	1700	1628	1625	1658
Mtnr1a	1	31	27	18	27	23	21	27
Mto1	480	497	490	496	521	567	438	506
Mtpap	984	1015	963	1006	987	844	844	868
Mtpn	5670	6171	5942	5939	6143	6036	6062	5764
Mtr	266	288	217	299	249	285	243	326
Mtres1	642	714	673	601	714	653	649	782
Mtrex	1557	1676	1458	1600	1670	1603	1639	1531
Mtrf1	135	143	95	98	135	155	118	102
Mtrf1l	265	232	310	306	381	380	372	333
Mtrr	207	183	172	152	189	178	161	163
Mtss1	895	1015	1014	994	933	963	861	827
Mtss2	142	199	187	210	202	167	140	187
Mttp	127579	143313	121658	115512	136483	141604	142477	132269
Mturn	106	95	114	77	92	94	90	85
Mtus1	3384	3815	3686	3830	3706	3895	3907	3452
Mtus2	138	173	163	159	164	177	126	91
Mtx1	1436	1452	1442	1384	1246	1274	1144	1192
Mtx2	2978	3120	2849	3027	3143	3047	2917	2781
Mtx3	457	540	580	478	418	486	447	429
Muc1	26	33	26	8	80	101	66	84
Muc13	123391	145326	123870	108930	141247	154075	151680	138903
Muc16	2	1	7	6	15	7	6	2
Muc2	26701	30205	32147	33923	28715	27077	25917	26344
Muc20	10	20	21	13	18	15	4	9
Muc3	85869	87740	90383	99057	73923	75432	90560	84328

Transcriptome sequencing yielded total genetic results for the MOD and APS groups, with a total of 15,936 variables

Continued from above

Muc3a	4588	4060	4593	5782	3692	3470	4263	4027
Muc4	3607	4277	4493	4177	4035	4489	4184	3808
Muc5b	0	0	0	0	0	0	1	0
Muc6	5	3	25	15	2	6	10	4
Mucl3	1	2	0	0	1	1	1	1
Mug1	5	2	1	4	39	11	6	10
Mug2	7	10	1	3	3	7	4	5
Mul1	4248	4278	4362	4187	3788	4192	4137	3749
Mup1	0	0	0	0	8	0	0	0
Mup10	0	0	0	0	5	1	0	0
Mup11	0	0	0	0	8	2	5	1
Mup12	0	0	0	0	5	0	0	0
Mup13	0	0	0	0	4	0	1	0
Mup14	0	0	0	0	49	0	0	13
Mup15	0	0	0	0	21	0	0	5
Mup16	0	0	0	0	1	0	0	2
Mup17	0	0	0	0	6	0	0	0
Mup19	0	0	0	0	4	0	0	4
Mup20	4	0	0	3	112	0	0	39
Mup21	0	0	0	0	5	0	0	0
Mup22	2	0	0	0	5	2	5	6
Mup3	3	0	0	0	98	4	3	62
Mup5	1	0	2	0	0	0	0	1
Mup7	0	0	0	0	75	0	0	15
Mup9	0	0	0	0	0	0	1	0
Mus81	300	352	296	286	422	301	292	308
Musk	11	29	11	13	14	6	9	15
Mustn1	172	153	177	180	191	181	148	128
Mutyh	55	32	43	39	56	51	41	47
Mvb12a	2886	2935	3080	2597	2776	2997	2483	2548
Mvb12b	173	206	146	168	176	206	181	143
Mvd	1347	1408	1621	1574	1430	1338	1226	1411
Mvk	990	893	986	1109	837	757	790	834
Mvp	18191	18699	17654	17658	17229	17191	16956	16984
Mxd1	23020	23465	21281	22391	18806	20468	21757	19069
Mxd3	136	127	132	105	108	102	94	127
Mxd4	713	653	714	688	857	717	711	719
Mxi1	8179	8506	7134	7344	7491	8354	8291	7274
Mxra7	490	507	499	507	538	511	556	519
Mxra8	418	430	485	427	408	395	366	309
Myadm	3200	3022	3125	3056	3135	3066	2967	2920
Myadml2	0	1	2	0	0	1	0	3
Myb	1706	1936	1567	1683	1808	1759	1680	1680
Mybbp1a	3122	3237	3126	2736	3374	3201	2775	2690
Mybl1	40	29	13	13	41	21	7	18
Mybl2	501	478	459	443	559	462	376	377
Mybpc2	8	13	5	3	13	2	4	15
Mybpc3	6	0	0	0	0	0	1	0
Mybph	1	0	3	0	3	0	0	0
Mybphl	0	0	0	0	0	0	1	2
Myc	905	996	1014	904	803	811	638	781
Mycbp	921	971	941	919	846	876	946	885
Mycbp2	944	1091	1005	1092	973	1053	945	842
Mycbpap	148	111	145	164	118	169	145	128
Mycl	551	537	538	497	473	443	439	391
Mycn	39	47	19	24	34	23	25	29
Myct1	33	38	44	48	29	57	61	50
Myd88	1686	1852	1663	1784	1629	1680	1585	1752

Transcriptome sequencing yielded total genetic results for the MOD and APS groups, with a total of 15,936 variables

Continued from above

Mydgf	3200	3402	3598	3252	3750	3765	3255	3474
Myef2	571	674	797	653	701	782	696	615
Myf6	0	0	0	0	0	0	0	1
Myg1	630	777	695	671	648	609	559	632
Myh10	219	269	319	253	188	204	192	172
Myh11	5743	6070	6669	6573	5736	6092	6787	6030
Myh14	25248	25207	24278	24939	24709	24691	25550	24525
Myh2	0	0	0	1	0	0	0	0
Myh3	9	8	2	2	0	5	6	2
Myh7	2	1	4	11	11	4	4	2
Myh7b	28	10	17	18	6	14	13	15
Myh9	17750	18015	17813	18602	17100	17203	16440	15906
Myl1	4	31	16	23	11	7	17	14
Myl10	0	0	4	1	0	0	0	0
Myl12a	5140	5486	5305	5180	5142	5447	4988	5253
Myl12b	11851	12303	12413	11792	11892	12412	11858	11654
Myl4	44	29	41	35	33	65	31	22
Myl6	21008	22063	22016	22765	22600	23704	21350	21975
Myl6b	18	38	10	13	13	10	22	16
Myl7	260	351	382	385	404	394	276	336
Myl9	3062	3341	3650	3731	3035	3417	3571	3290
Mylip	103	139	98	107	70	130	97	89
Mylk	11871	13511	13173	12374	13639	14185	13412	12168
Mylk2	0	1	0	0	0	0	0	0
Mylk3	5	1	0	4	10	3	2	7
Mylpf	62	70	50	66	43	86	87	78
Mymk	1	0	0	0	0	4	0	0
Mymx	10	7	6	0	1	4	1	0
Mynn	594	764	692	667	721	716	804	709
Myo10	1814	1918	1725	1641	1510	1541	1544	1482
Myo15	2	2	2	3	2	1	0	0
Myo15b	34471	35754	32747	31764	32931	34091	35031	32662
Myo16	1	1	10	3	0	2	9	3
Myo18a	9963	10186	9913	9606	9915	9887	9787	9325
Myo18b	12	14	23	7	19	38	14	20
Myo19	1030	1003	956	1031	1055	1002	1045	945
Myo1a	46778	50701	46363	43732	47620	50818	50785	46930
Myo1b	584	690	641	607	592	673	626	551
Myo1c	2882	2670	2889	3118	2563	2441	2497	2436
Myo1d	17237	18921	18112	16266	18076	18535	17911	17284
Myo1e	3710	4202	3616	3270	3571	3771	3734	3484
Myo1f	201	222	235	215	202	200	156	174
Myo1g	92	81	103	144	88	108	87	80
Myo1h	79	79	66	104	59	50	47	51
Myo3a	1	0	0	1	0	0	1	0
Myo3b	1	1	0	1	0	1	0	1
Myo5a	148	210	187	208	215	188	215	155
Myo5b	11743	13186	11574	11157	12648	13157	13263	12603
Myo5c	931	1050	1088	1070	1031	1130	1018	1072
Myo6	10103	11523	10334	10117	11090	11211	11159	10254
Myo7a	3237	3868	3401	3191	3615	3659	3508	3192
Myo7b	18420	20310	17545	15815	19565	20556	20583	18314
Myo9a	275	325	231	309	218	254	278	223
Myo9b	1873	1915	1725	1730	1550	1544	1615	1464
Myoc	0	0	0	0	4	0	0	0
Myocd	61	69	121	140	92	84	86	57
Myocos	0	0	0	0	0	0	0	1
Myof	654	634	657	704	686	705	602	599

Transcriptome sequencing yielded total genetic results for the MOD and APS groups, with a total of 15,936 variables

Continued from above

Myom1	152	139	136	162	129	128	193	172
Myom2	0	0	1	6	2	1	0	0
Myom3	150	197	181	207	177	136	196	112
Myorg	797	727	819	644	700	749	704	680
Myot	1	0	0	0	0	1	0	1
Myoz1	1	0	4	0	1	0	0	1
Myoz2	0	0	1	0	0	0	0	0
Myoz3	0	0	0	0	0	0	0	1
Mypn	8	1	0	0	9	0	5	8
Mypop	43	18	5	21	21	33	29	16
Myrf	2098	2045	2171	2066	2033	1956	1905	2037
Myrfl	1997	1978	1899	2083	2039	2071	1973	1952
Myrip	96	93	64	71	64	79	42	58
Mysm1	690	751	839	845	769	813	763	753
Myt1	27	25	18	30	30	33	31	43
Myt1l	0	0	1	0	1	1	3	7
Myzap	1341	1526	1426	1210	1550	1638	1531	1372
Mzb1	536	441	417	491	415	470	352	416
Mzf1	5	9	0	14	12	6	20	8
Mzt1	1210	1196	1196	1200	1101	1095	1147	1081
Mzt2	537	478	573	593	487	498	545	500
N4bp1	2821	3343	3179	2859	3375	3423	3128	3123
N4bp2	668	688	651	703	773	804	706	685
N4bp2l1	178	222	247	225	261	259	304	249
N4bp2l2	1777	1985	1846	1879	2001	2181	2102	1889
N4bp3	199	166	202	177	193	191	158	195
N6amt1	306	312	347	321	262	312	267	270
Naa10	1093	1123	1178	1086	1124	1184	1019	1071
Naa11	0	0	0	0	0	0	0	1
Naa15	2234	2460	2349	2378	2372	2467	2441	2181
Naa16	224	226	222	245	268	267	244	258
Naa20	2357	2505	2545	2391	2451	2410	2454	2446
Naa25	1005	1052	1045	981	1099	1189	997	1077
Naa30	1269	1299	1432	1365	1376	1413	1376	1449
Naa35	2755	3109	2941	2949	3046	2823	2775	2821
Naa38	908	972	1018	900	1118	1172	892	961
Naa40	1070	1135	1091	1005	1142	1125	957	923
Naa50	2142	2319	2038	2086	2301	2449	2244	2314
Naa60	2831	3054	2906	2719	2995	2873	2850	2708
Naa80	477	416	378	481	443	422	441	360
Naaa	2995	2366	2772	3499	1824	1648	1654	1786
Naalad2	40	41	43	29	37	38	21	26
Naaladl1	2799	3159	2332	2128	3058	3557	3869	3228
Naaladl2	46	28	48	22	60	44	63	58
Nab1	1440	1632	1547	1550	1627	1679	1547	1496
Nab2	732	774	758	799	523	636	716	583
Nabp1	354	332	337	326	502	474	528	435
Nabp2	2099	2245	2240	2003	2240	2066	2180	1920
Naca	8378	8890	8765	8719	9182	8469	8223	8274
Nacad	28	36	44	42	36	31	48	38
Nacc1	5152	5287	5260	4653	5293	5375	4893	4728
Nacc2	2256	2272	2258	2288	2030	2136	2125	1939
Nadk	8373	8146	8112	8826	7850	7700	7600	7765
Nadk2	3147	3173	3356	3326	3826	3432	3049	3191
Nadsyn1	2313	2274	2237	2221	2064	2294	2165	1934
Nae1	968	1036	954	886	964	970	1162	876
Naf1	180	214	254	225	293	260	290	247
Naga	2997	3143	2973	2817	2770	2839	2625	2630

Transcriptome sequencing yielded total genetic results for the MOD and APS groups, with a total of 15,936 variables

Continued from above

Nagk	1850	1778	1701	1777	1518	1669	1501	1663
Naglu	446	415	314	364	395	426	388	417
Nagpa	643	669	636	624	636	741	728	663
Nags	3660	3526	3814	3478	3590	3847	3587	3583
Naif1	126	149	142	141	133	102	125	126
Naip1	4229	4517	4335	4224	4439	4574	4585	4370
Naip2	3250	3729	3421	3321	3639	3806	3555	3332
Naip5	2874	2781	3021	3107	2568	2747	2769	2513
Naip6	3881	3849	3917	4001	3619	3689	3585	3239
Nalcn	7	2	5	4	9	10	9	7
Nampt	6959	7838	7029	6476	6816	7525	7204	6434
Nanog	0	0	4	0	0	0	4	0
Nanos1	8	15	16	37	65	34	20	14
Nanos3	0	4	3	0	0	0	0	2
Nanp	36	70	12	6	2	8	71	8
Nans	2794	3130	3093	3077	3278	3208	2874	2950
Nap1l1	1253	1383	1318	1264	1642	1534	1309	1341
Nap1l2	1	1	1	6	4	2	4	1
Nap1l3	20	31	10	10	25	7	2	8
Nap1l4	2889	2982	2886	3123	3065	2964	2835	2972
Nap1l5	17	40	37	37	27	35	29	32
Napa	3195	3033	3258	3104	3122	3151	3142	3023
Napb	22	38	21	23	32	23	34	27
Napepld	357	406	408	411	314	404	347	355
Napg	1164	1231	1128	1110	1207	1306	1222	1111
Naprt	8639	8215	7996	8117	7411	7726	7963	7768
Napsa	30	39	23	29	37	24	41	28
Narf	2565	2512	2337	2569	2796	2814	2626	2380
Nars	11948	13188	11841	11305	13288	13481	12347	12161
Nars2	626	597	532	573	556	503	504	477
Nasp	1445	1376	1640	1456	1619	1666	1434	1435
Nat1	0	0	0	0	0	1	0	1
Nat10	696	706	704	620	797	772	641	728
Nat14	70	41	74	50	76	56	37	66
Nat2	1407	1550	1484	1497	1267	1369	1459	1305
Nat3	1	0	0	0	0	0	0	0
Nat8	1858	1695	1766	1768	1675	1825	1855	1997
Nat8f1	289	315	281	319	335	270	332	307
Nat8f2	36	100	59	48	114	77	101	84
Nat8f3	6	4	2	1	1	0	10	0
Nat8f4	6833	7805	7058	6409	7487	8150	7987	7212
Nat8f5	1671	1863	1908	1893	1929	2142	2105	2099
Nat8f6	39	4	22	25	15	22	20	17
Nat8f7	0	0	0	0	0	2	0	0
Nat8l	5	5	5	5	12	3	8	2
Nat9	526	602	532	475	539	522	478	498
Natd1	313	343	294	302	302	281	288	315
Nav1	484	529	489	508	437	444	489	418
Nav2	421	563	614	680	380	427	456	388
Nav3	0	5	1	4	2	6	6	2
Naxd	1570	1564	1603	1672	1575	1647	1555	1407
Naxe	1098	1101	1149	1109	1140	1162	1046	1078
Nbas	2488	2676	2444	2340	2546	2778	2314	2198
Nbdy	125	139	139	137	144	165	128	153
Nbea	441	596	502	533	512	600	601	520
Nbeal1	1557	1738	1761	1744	1772	1760	2083	1848
Nbeal2	921	859	855	931	1094	924	774	931
Nbl1	980	904	1038	1005	846	939	891	801

Transcriptome sequencing yielded total genetic results for the MOD and APS groups, with a total of 15,936 variables

Continued from above

Nbn	493	454	434	524	526	518	557	486
Nbr1	6287	7183	6177	5908	7380	7330	7625	6881
Ncald	584	658	555	544	622	560	720	670
Ncam1	132	129	126	148	120	146	121	131
Ncam2	3	23	13	14	18	6	7	14
Ncan	0	1	1	3	0	5	0	0
Ncapd2	1761	1759	1917	1680	1737	1661	1456	1607
Ncapd3	608	776	717	729	752	852	718	649
Ncapg	541	631	519	509	617	553	461	551
Ncapg2	463	524	452	454	440	544	438	371
Ncaph	578	698	617	642	697	599	543	588
Ncaph2	3031	3106	3116	3031	3160	3144	2814	2839
Ncbp1	1525	1677	1488	1487	1876	1738	1587	1539
Ncbp2	481	526	517	419	588	657	503	603
Ncbp3	905	989	911	884	974	884	850	873
Nccrp1	0	0	0	0	5	0	0	0
Ncdn	1028	1058	990	1067	1042	993	990	913
Nceh1	607	513	514	470	660	674	632	497
Ncf1	300	257	231	240	256	288	213	225
Ncf2	396	456	347	382	390	476	306	392
Ncf4	153	94	110	103	128	137	114	151
Nck1	493	623	458	534	681	677	642	602
Nck2	1964	2108	2048	2103	1767	1883	1870	1870
Nckap1	8680	9270	8579	8454	9128	9361	9013	8869
Nckap1l	470	486	399	488	385	385	434	389
Nckap5	269	292	209	293	221	226	278	228
Nckap5l	64	49	54	89	69	41	53	51
Nckipsd	315	285	331	309	295	245	256	303
Ncl	7202	8166	7081	7522	7422	7634	7132	6765
Ncln	6333	6851	6406	5972	6628	6657	5860	5734
Ncmap	18	14	17	9	14	16	10	26
Ncoa1	1872	2031	1924	1898	2003	1932	2080	1765
Ncoa2	2366	2634	2606	2580	2482	2503	2575	2296
Ncoa3	1553	1548	1591	1616	1508	1699	1597	1515
Ncoa4	3554	3704	3122	3205	4085	4071	3883	3645
Ncoa5	1156	1118	1054	1162	1157	1103	1023	1077
Ncoa6	1857	2291	2152	1900	1988	1908	1922	1820
Ncoa7	494	673	573	505	617	640	552	536
Ncor1	10679	12087	10657	10204	11332	11690	11567	10367
Ncor2	1478	1442	1636	1537	1314	1451	1368	1374
Ncr1	5	5	7	2	10	1	1	0
Ncs1	24	72	62	41	34	66	26	33
Ncstn	6643	7078	6771	6353	6132	6300	5942	6027
Ndc1	963	999	917	824	1029	949	798	816
Ndc80	150	216	155	166	253	189	150	195
Nde1	570	637	632	584	713	584	550	586
Ndel1	1256	1260	1438	1311	1339	1375	1429	1416
Ndfip1	7099	7286	7098	6800	7655	7902	7542	7278
Ndfip2	7958	8334	7687	7607	8603	9081	9026	8334
Ndn	82	95	76	88	98	101	102	99
Ndnf	4	1	2	7	3	12	0	7
Ndor1	997	939	1057	993	991	992	900	911
Ndp	0	4	0	0	0	0	0	0
Ndrg1	2055	2212	2319	2237	1868	1877	2191	2006
Ndrg2	850	766	893	1042	733	627	687	682
Ndrg3	3794	3538	3760	3967	3616	3558	3380	3416
Ndrg4	91	115	121	106	111	95	121	110
Ndst1	5689	6157	5553	5392	6182	6324	6202	5411

Transcriptome sequencing yielded total genetic results for the MOD and APS groups, with a total of 15,936 variables

Continued from above

Ndst2	884	966	906	878	805	847	858	872
Ndst3	4	6	3	4	9	9	5	2
Ndst4	0	1	1	0	2	0	2	0
Ndufa1	2183	2125	2189	2124	1917	2211	2182	1933
Ndufa10	8582	8569	8584	9074	8069	7853	7540	7665
Ndufa11	4280	4148	4628	4669	4098	4095	3964	3808
Ndufa12	3319	3464	3368	3285	3132	3051	2935	2856
Ndufa13	3791	4503	4127	4286	3678	3969	4069	3488
Ndufa2	2775	2752	2849	2930	2917	3184	2912	2788
Ndufa3	2107	2012	2171	2133	1851	2065	2023	1844
Ndufa4	7861	9030	8378	7681	9126	9835	9173	9213
Ndufa4l2	41	26	13	18	10	37	29	26
Ndufa5	2560	2529	2453	2814	2510	2647	2430	2422
Ndufa6	6151	6215	6196	6583	6179	6480	6519	6187
Ndufa7	3128	3088	3147	3229	2822	3182	2903	2724
Ndufa8	3957	4104	4447	4364	3632	3767	3597	3697
Ndufa9	7422	7538	7518	7424	7183	7409	7045	7213
Ndufab1	5866	5866	6258	6099	5872	5866	5575	5820
Ndufab1-	269	317	353	250	257	271	241	299
Ndufaf1	609	654	570	549	505	601	554	554
Ndufaf2	343	339	354	367	416	345	374	338
Ndufaf3	646	678	631	657	676	678	607	674
Ndufaf4	1143	1164	986	1180	1199	1186	1174	1044
Ndufaf5	429	421	473	470	419	397	395	418
Ndufaf6	301	367	346	410	365	366	269	352
Ndufaf7	536	544	560	508	533	592	468	456
Ndufaf8	908	919	955	954	927	939	851	944
Ndufb1	1677	1863	1730	1818	1740	1855	1785	1593
Ndufb10	6259	6562	6344	7193	5708	6153	5798	5612
Ndufb11	4847	4892	5249	5132	5127	5204	4797	4544
Ndufb2	2559	2684	2563	2854	2665	2691	2522	2626
Ndufb3	2946	2746	2786	2932	2612	2832	2769	2624
Ndufb4	2381	2495	2496	2419	2339	2464	2251	2237
Ndufb4b	58	62	46	83	58	65	90	68
Ndufb4c	20	18	44	38	30	42	23	16
Ndufb5	5335	5481	5275	5539	5330	5870	5056	5148
Ndufb6	2947	3310	3216	3284	3005	3175	2937	3183
Ndufb7	4884	4128	4269	4727	4161	3986	4257	3988
Ndufb8	6830	7167	6829	6946	6405	7012	6283	6432
Ndufb9	9134	9089	8943	9232	8748	9156	8701	8899
Ndufc1	3034	3013	2810	3095	2777	2951	2787	2512
Ndufc2	4116	3935	4146	4267	4137	4103	3815	3809
Ndufs1	7712	8070	7720	8077	7702	8082	7794	7469
Ndufs2	17316	16807	16651	17078	16385	16622	15854	15897
Ndufs3	5041	5116	5078	5146	4753	4966	4612	4520
Ndufs4	3863	3723	3717	4052	3855	4190	3759	3735
Ndufs5	2311	2297	2323	2361	2240	2169	2067	2222
Ndufs6	2780	2751	2773	2738	2630	2769	2582	2669
Ndufs6b	75	81	73	64	96	78	73	47
Ndufs7	5568	5199	5152	5437	5073	5288	4573	4991
Ndufs8	4862	4502	5084	5219	4789	4405	4303	4587
Ndufv1	9595	9274	9154	9938	9098	8937	8575	8635
Ndufv2	9331	9381	9372	9836	8826	9285	9056	8686
Ndufv3	3139	3244	3235	3290	2918	3155	2746	3016
Neb	6	7	7	6	8	1	8	5
Nebl	15	15	19	14	23	5	13	8
Necab1	22	19	23	13	20	8	26	11
Necab2	9	4	16	6	7	7	6	10

Transcriptome sequencing yielded total genetic results for the MOD and APS groups, with a total of 15,936 variables

Continued from above

Necab3	1	0	2	0	0	5	0	3
Necap1	2212	2500	2240	1939	2415	2475	2675	2263
Necap2	2466	2570	2461	2488	2321	2530	2448	2497
Nectin1	1151	1395	1267	1222	1293	1277	1197	1141
Nectin2	8051	8260	8116	7476	8092	8152	7720	7519
Nectin3	5070	5365	4829	4807	5408	5517	5293	5084
Nectin4	25	20	17	34	29	29	27	14
Nedd1	634	779	699	653	738	710	705	642
Nedd4	5738	6482	6479	6085	6129	6258	6041	5497
Nedd4l	3023	3260	2972	2526	3155	3087	3085	2800
Nedd8	2378	2667	2632	2687	2697	2802	2456	2421
Nedd9	1636	1757	1584	1835	1410	1520	1311	1414
Nefh	3	18	11	9	1	17	11	5
Nefl	9	19	22	13	16	13	21	15
Nefm	24	5	11	17	12	19	12	20
Negr1	17	22	27	34	35	19	30	22
Neil1	262	206	230	259	216	216	190	206
Neil2	27	24	17	27	16	8	11	22
Neil3	210	210	195	209	183	258	179	166
Nek1	246	231	286	265	307	334	240	267
Nek10	22	48	24	20	49	49	43	40
Nek11	3	3	1	8	9	0	1	8
Nek2	1010	1067	1143	912	1119	980	825	899
Nek3	514	533	501	448	547	557	556	546
Nek4	288	250	275	273	266	295	297	229
Nek5	12	3	10	9	29	24	16	20
Nek6	1818	2186	1931	1923	2038	1972	2355	2253
Nek7	532	569	535	527	560	681	559	531
Nek8	214	178	194	196	218	191	192	189
Nek9	2748	3137	2942	2883	3056	2955	2860	2716
Nelfa	820	835	812	748	839	838	729	818
Nelfb	2584	2439	2415	2260	2511	2524	2353	2409
Nelfcd	1429	1364	1405	1218	1209	1169	1169	1246
Nelfe	1433	1713	1430	1422	1603	1495	1333	1418
Nell1	19	21	3	3	3	1	1	8
Nell2	6	12	7	17	8	20	2	8
Nemf	937	1002	1004	1041	1053	1101	1185	1107
Nemp1	543	656	603	484	529	522	571	488
Nemp2	79	67	63	61	65	58	67	66
Nenf	221	205	253	243	260	285	241	252
Neo1	2224	2539	2293	2125	2248	2532	2160	2147
Nepn	1	0	0	0	0	0	0	0
Nepro	400	347	435	414	414	424	319	435
Nes	201	199	226	190	278	248	248	216
Net1	14148	14247	13768	14056	11694	12426	12885	12578
Neto1	10	42	30	28	24	26	16	32
Neto2	56	56	66	50	84	45	58	54
Neu1	2072	1960	1803	1949	1876	1794	1921	1916
Neu2	12	12	30	15	19	31	14	26
Neu3	154	137	232	225	200	171	177	97
Neurl1a	42	34	48	32	52	30	45	39
Neurl1b	859	1191	931	967	972	1069	1088	990
Neurl2	12	16	11	18	20	11	9	14
Neurl3	16392	17580	17018	16194	18165	19805	18516	17434
Neurl4	1936	1886	2049	2280	1965	1795	2058	1924
Neurod1	149	136	160	165	129	151	129	193
Neurod2	1	2	16	10	2	3	3	3
Neurod6	0	0	1	0	0	0	0	2

Transcriptome sequencing yielded total genetic results for the MOD and APS groups, with a total of 15,936 variables

Continued from above

Neurog3	27	51	45	32	50	38	41	30
Nexmif	1	1	2	6	0	10	9	6
Nexn	80	45	72	90	60	79	50	74
Nf1	1117	1182	1026	1117	1052	987	1031	1025
Nf2	4628	4711	4428	4541	4203	4524	4322	4164
Nfam1	433	380	417	420	303	364	363	320
Nfasc	40	75	48	43	77	83	67	87
Nfat5	1539	1620	1734	1769	1626	1914	1917	1519
Nfatc1	450	527	408	414	432	450	361	326
Nfatc2	149	167	141	111	135	103	61	133
Nfatc2ip	278	398	353	279	380	337	295	324
Nfatc3	2788	3290	3200	3337	3399	3241	3153	3042
Nfatc4	51	77	62	69	81	66	55	48
Nfe2	3	2	1	8	0	3	1	4
Nfe2l1	3763	4098	4045	3926	4098	3863	3873	3647
Nfe2l2	8696	9882	8951	8268	12067	11620	9835	9999
Nfe2l3	5	8	1	2	5	1	1	1
Nfia	316	378	409	388	412	342	359	422
Nfib	968	1015	1016	1024	945	1069	1060	883
Nfic	675	630	725	641	622	661	524	490
Nfil3	914	784	806	702	753	741	537	601
Nfix	1093	1158	1212	1186	1073	1136	936	878
Nfkb2	1439	1464	1502	1434	1415	1336	1412	1408
Nfkbia	3424	3792	3779	3531	4386	4430	4383	4507
Nfkbib	2128	2285	1923	1695	2147	2179	2044	1978
Nfkbid	88	98	70	137	77	83	89	52
Nfkbie	217	273	221	183	156	166	193	120
Nfkbil1	391	472	477	533	427	482	432	484
Nfkbiz	4049	4519	3826	3210	4287	4549	3831	3896
Nfrkb	711	812	763	678	703	708	645	623
Nfs1	2026	2107	2379	2115	2224	2137	1971	1967
Nfu1	870	947	937	876	919	955	905	911
Nfx1	1668	1989	1671	1783	1876	1682	1702	1696
Nfxl1	832	835	948	854	984	917	882	866
Nfya	1184	1273	1213	1259	1531	1424	1193	1162
Nfyb	710	722	659	626	681	706	654	636
Nfyc	2050	2017	2063	2067	2022	2012	1940	1910
Ngb	8	2	17	5	7	17	19	12
Ngdn	1018	1120	1060	1077	1156	1309	1190	1145
Ngef	1011	1220	1278	1092	1111	1174	1029	981
Ngf	14	13	8	3	2	2	4	1
Ngfr	144	93	125	171	131	97	136	102
Ngly1	1505	1668	1486	1503	1779	1679	1911	1596
Ngrn	733	721	718	811	853	752	803	764
Nhej1	169	173	192	152	172	172	152	167
Nhlh1	2	0	5	0	11	2	3	2
Nhlh2	0	0	0	0	0	0	0	4
Nhlrc1	102	92	105	109	60	85	70	98
Nhlrc2	2248	2318	2314	2218	2346	2278	2252	2129
Nhlrc3	624	698	610	664	711	730	634	683
Nhp2	1356	1332	1278	1286	1380	1279	1170	1194
Nhs	89	195	93	125	105	105	114	63
Nhsl1	6170	6719	6671	6684	6036	6022	5796	5660
Nhsl2	16	37	27	21	17	41	36	22
Niban1	355	449	405	439	407	532	443	457
Niban2	1840	1910	1734	1953	1858	1736	1542	1543
Niban3	16	25	20	26	13	19	13	37
Nicn1	88	83	103	81	61	125	77	70

Transcriptome sequencing yielded total genetic results for the MOD and APS groups, with a total of 15,936 variables

Continued from above

Nid1	1222	1407	1501	1437	1283	1352	1224	988
Nid2	549	531	629	532	607	623	499	480
Nif3l1	542	593	622	523	628	531	570	563
Nifk	813	1009	817	835	883	911	840	841
Nim1k	6	1	6	7	11	17	10	3
Nin	289	307	351	315	312	333	274	274
Ninj1	689	738	651	606	749	684	603	638
Ninj2	0	0	5	0	2	2	0	0
Ninl	69	78	57	50	38	52	49	49
Nip7	727	629	687	599	738	715	603	762
Nipa1	50	44	83	56	38	52	53	53
Nipa2	3642	3672	3429	3629	3640	3639	3760	3644
Nipal1	1232	1458	1422	1219	1319	1377	1237	1227
Nipal2	1113	1164	921	994	1115	1101	1039	996
Nipal3	1227	1421	1332	1338	1572	1372	1453	1457
Nipal4	13	8	17	12	8	23	23	15
Nipbl	2645	3171	2759	2787	3202	3269	3178	2760
Nipsnap1	1311	1390	1428	1378	1379	1324	1127	1274
Nipsnap2	1828	1860	1856	1868	1643	1682	1586	1511
Nipsnap3t	3980	4049	3828	3799	3707	3713	3747	3506
Nisch	5734	6061	5982	5881	5913	5738	5663	5377
Nit1	2274	2655	2319	2254	2470	2653	2634	2536
Nit2	592	610	702	659	572	710	682	610
Nkain1	223	263	205	237	257	304	212	208
Nkain2	0	2	2	2	3	1	7	4
Nkain3	0	1	0	0	1	0	1	4
Nkain4	13	6	22	29	35	32	18	28
Nkap	475	525	453	514	516	545	571	517
Nkapd1	590	617	566	592	540	605	690	580
Nkapl	1	0	0	0	3	4	0	1
Nkd1	176	199	188	226	185	236	164	188
Nkd2	82	133	112	156	125	167	162	134
Nkg7	116	88	105	95	82	74	91	117
Nkiras1	686	767	641	569	659	703	655	574
Nkiras2	4400	4358	4440	4183	4245	4148	4400	4083
Nkpd1	1	0	0	8	2	1	4	0
Nkrf	221	183	179	225	221	204	192	218
Nktr	2844	3390	3055	3137	3430	3465	3213	2983
Nkx1-2	1	0	0	1	2	0	0	0
Nkx2-1	0	1	0	0	0	0	0	0
Nkx2-2	63	35	51	50	45	51	35	42
Nkx2-3	765	732	684	713	746	747	647	707
Nkx3-2	3	0	5	4	8	0	7	5
Nkx6-1	0	1	0	0	0	0	0	0
Nkx6-2	12	13	16	5	3	6	7	5
Nkx6-3	1	1	2	0	1	1	0	0
Nle1	261	321	308	240	305	302	240	308
Nlgn1	6	5	5	1	7	9	1	2
Nlgn2	168	209	181	184	218	197	162	150
Nlgn3	1	6	0	11	1	1	9	15
Nlgn4l	74	88	104	131	125	111	107	110
Nlk	531	608	553	533	593	679	574	575
Nln	1180	1286	1122	1282	1148	1271	1310	1297
Nlrc3	22	29	26	12	23	48	35	31
Nlrc4	2686	2939	2499	2391	3194	3157	3138	3001
Nlrc5	929	1258	1119	1054	799	753	681	669
Nlrp10	5	0	5	0	1	8	5	1
Nlrp12	2	1	7	4	2	1	5	0

Transcriptome sequencing yielded total genetic results for the MOD and APS groups, with a total of 15,936 variables

Continued from above

Nlrp14	0	0	0	1	0	0	0	0
Nlrp1a	26	10	12	14	26	21	14	12
Nlrp1b	524	545	497	565	564	565	591	511
Nlrp3	20	10	15	9	11	31	21	12
Nlrp4e	0	0	1	0	0	0	0	0
Nlrp5	0	0	0	0	0	0	1	0
Nlrp6	15069	15849	16178	15246	18226	18477	15755	14631
Nlrp9b	932	791	892	901	879	1053	1008	929
Nlrp9c	0	0	2	0	0	0	0	0
Nlrx1	428	513	444	357	443	458	478	407
Nmb	22	22	15	23	17	17	14	40
Nmbr	0	4	1	1	2	0	0	1
Nmd3	893	978	1058	943	1009	1156	1087	971
Nme1	2160	2460	2143	2009	2589	2510	2167	2405
Nme2	6502	7179	7398	6683	7628	7466	6625	7236
Nme3	314	317	349	336	337	432	305	391
Nme4	88	119	76	113	109	91	93	131
Nme5	9	4	0	5	0	0	0	1
Nme6	288	258	283	245	312	338	240	223
Nme7	763	772	740	741	788	781	823	752
Nme9	4	1	2	12	5	4	6	7
Nmi	2973	3172	2903	3021	2724	3056	2915	2602
Nmnat1	700	741	689	618	764	693	592	594
Nmnat2	9	10	20	8	5	14	8	19
Nmnat3	384	514	446	374	432	429	485	489
Nmral1	248	237	261	213	245	230	235	169
Nmrk1	148	242	198	222	286	231	235	161
Nmt1	4949	5125	4865	4859	5033	4940	4913	4624
Nmt2	384	476	421	453	520	473	398	376
Nmu	22	17	25	32	22	23	22	16
Nmur1	61	43	45	73	51	65	39	41
Nmur2	10	5	6	25	5	11	1	6
Nnat	56	60	85	74	37	43	45	21
Nnmt	23	29	27	14	20	26	24	31
Nnt	1415	1332	1292	1314	1385	1370	1352	1295
Noa1	790	730	784	818	867	824	754	768
Nob1	538	627	510	528	601	670	450	540
Nobox	0	4	0	0	0	0	0	0
Noc2l	1733	1746	1848	1668	1831	1741	1620	1638
Noc3l	371	436	411	378	552	541	471	400
Noc4l	784	859	719	685	741	723	654	642
Noct	1485	1740	1829	1706	1704	1860	1866	2008
Nod1	415	504	440	421	363	334	377	304
Nod2	94	132	118	117	96	118	102	102
Nodal	2	2	0	0	0	2	0	0
Nog	25	18	11	11	21	6	28	22
Nol10	252	298	282	258	277	261	216	273
Nol11	758	817	841	728	864	784	763	790
Nol12	327	422	333	349	364	369	327	371
Nol3	55	58	51	58	58	59	40	51
Nol4	0	7	3	0	1	0	0	0
Nol4l	3177	3601	3403	3326	3536	3615	3302	3217
Nol6	1052	1162	969	893	1078	1001	878	978
Nol7	2130	2310	2235	2136	2429	2566	2156	2116
Nol8	285	379	269	267	425	433	415	436
Nol9	1184	1193	1078	1045	1448	1403	1191	1224
Nolc1	1474	1640	1519	1531	1660	1750	1361	1593
Nom1	765	832	812	824	922	912	850	778

Transcriptome sequencing yielded total genetic results for the MOD and APS groups, with a total of 15,936 variables

Continued from above

Nomo1	8549	9429	9060	8392	9389	8673	8267	7881
Nono	5980	6257	6105	5933	6605	6330	6079	5773
Nop10	933	883	866	987	1059	904	879	984
Nop14	491	550	539	516	462	659	574	526
Nop16	445	478	418	414	502	432	415	492
Nop2	1305	1228	1265	1251	1516	1513	1248	1326
Nop53	3637	3828	3862	3717	3634	3392	3314	3197
Nop56	1288	1369	1256	1137	1448	1389	1166	1285
Nop58	1267	1682	1301	1350	1758	1570	1504	1420
Nop9	1449	1523	1536	1390	1534	1474	1392	1524
Nos1	27	18	52	40	39	40	46	27
Nos1ap	78	99	81	68	98	66	81	99
Nos3	76	76	81	91	136	119	77	95
Nosip	1489	1524	1459	1496	1425	1404	1286	1309
Nostrin	2328	2710	2310	2234	2634	2763	2746	2623
Notch1	678	940	819	912	1026	837	733	735
Notch2	513	618	561	554	566	570	524	546
Notch3	199	167	263	172	223	185	162	173
Notch4	248	246	256	263	208	226	230	195
Notum	2	2	3	0	2	2	6	4
Nova1	5	13	14	6	4	1	15	9
Nova2	32	24	32	27	20	38	32	26
Nox1	331	563	508	374	377	361	295	324
Nox4	4	15	9	10	5	2	13	3
Noxa1	725	802	740	802	777	744	695	768
Noxo1	1937	1923	1961	1825	1916	1667	1705	1805
Noxred1	7	11	7	5	23	12	6	8
Npas1	1	1	1	0	0	0	0	2
Npas2	1000	1075	771	688	1044	952	1040	990
Npas3	1	0	0	0	0	0	0	0
Npas4	16	18	10	14	14	11	14	3
Npat	449	459	462	467	514	444	415	395
Npb	0	0	0	1	0	0	6	1
Npc1	897	926	921	967	902	989	1028	978
Npc1l1	17334	19624	16336	16332	19174	19988	21009	17958
Npc2	4243	4577	4501	4351	4311	4238	3974	4153
Npdc1	2567	2392	2417	2383	2411	2402	2354	2176
Npepl1	2107	2351	2172	2086	2233	2148	1958	1933
Npepps	6529	6933	6752	6397	7304	7342	6879	6678
Npff	9	10	9	7	5	11	7	5
Npffr1	4	1	0	1	6	0	1	0
Nphp1	76	96	82	97	92	75	79	60
Nphp3	66	93	103	144	58	69	102	124
Nphp4	7	1	1	0	16	6	8	6
Nphs1	0	7	2	1	5	6	1	5
Npl	958	893	889	1015	1192	1072	1475	1555
Nploc4	3550	3672	3383	3264	3497	3483	3220	3326
Npm1	6333	6609	6436	6260	7079	7132	6933	6594
Npm2	2	5	0	7	1	1	0	7
Npm3	797	825	786	666	954	753	791	727
Npnt	912	966	975	923	910	826	696	632
Nppa	1	4	1	0	4	0	0	0
Npr1	80	107	77	111	63	112	100	101
Npr2	114	144	138	124	153	96	118	117
Npr3	20	25	21	28	55	48	36	30
Nprl2	336	363	317	364	388	412	353	424
Nprl3	306	338	284	257	304	296	242	272
Npsr1	0	0	1	0	1	0	0	0

Transcriptome sequencing yielded total genetic results for the MOD and APS groups, with a total of 15,936 variables

Continued from above

Nptn	6009	5893	6148	6434	5510	5795	5614	5407
Nptx1	13	21	18	16	15	11	10	2
Nptx2	54	57	44	42	36	68	66	70
Nptxr	1	1	1	0	5	1	0	0
Npy	32	67	34	44	55	43	35	44
Npy1r	8	16	4	13	11	10	15	10
Npy2r	6	7	20	9	2	4	17	10
Npy4r	2	0	0	4	2	2	4	0
Npy5r	0	0	0	4	0	0	0	0
Nqo1	1389	1550	1435	1401	1579	1585	1364	1424
Nqo2	1371	1407	1406	1448	1491	1562	1396	1462
Nr0b2	111	133	90	73	152	141	94	109
Nr1d1	3334	3109	2866	3226	2166	2518	2870	2464
Nr1d2	1638	1835	1752	1850	1662	2006	2075	1701
Nr1h2	4677	5116	4519	4519	4621	4583	4451	4245
Nr1h3	1810	1927	1773	1665	1998	2010	1977	1799
Nr1h4	2015	2107	1992	2005	2393	2465	2257	2168
Nr1i2	3631	4262	4058	3761	4298	4441	4682	4427
Nr1i3	1379	1687	1479	1439	1652	1612	1502	1486
Nr2c1	599	627	563	557	523	710	641	545
Nr2c2	2116	2395	2250	2455	2095	2364	2297	1970
Nr2c2ap	571	581	629	499	749	689	513	637
Nr2e3	41	26	32	15	16	33	9	21
Nr2f1	6	10	18	12	11	15	6	0
Nr2f2	246	223	202	245	289	277	206	171
Nr2f6	5529	5337	5439	5242	5533	5684	4959	5035
Nr3c1	4891	5227	4787	4765	5135	5320	5139	4400
Nr3c2	1963	1866	1668	1847	1678	1740	1710	1585
Nr4a1	131	203	202	161	101	120	81	107
Nr4a2	158	131	134	126	147	187	131	134
Nr4a3	45	27	26	37	28	26	18	17
Nr5a1	2	0	1	6	0	2	0	0
Nr5a2	813	911	967	915	1066	1019	889	815
Nr6a1	637	664	554	602	573	564	597	511
Nradd	38	45	59	58	54	39	42	30
Nrap	128	113	148	108	103	54	110	79
Nrarp	1035	1074	1256	1074	1250	1299	1064	1100
Nras	1704	1864	1857	1691	1720	1800	1816	1731
Nrbf2	591	753	605	624	679	768	783	650
Nrbp1	4072	4104	4150	3889	3978	4362	4124	3997
Nrbp2	264	277	339	333	260	267	298	277
Nrcam	5	6	1	1	3	5	7	2
Nrd1	2985	3320	3087	2997	3231	3339	3221	3074
Nrde2	675	719	597	570	587	605	479	435
Nrep	113	117	93	168	112	114	112	71
Nrf1	796	893	832	769	928	832	793	767
Nrg1	281	343	328	356	365	261	250	281
Nrg2	30	56	23	38	27	24	41	16
Nrg3	0	2	2	9	0	0	1	0
Nrg4	25	51	53	36	77	80	65	57
Nrgn	19	24	12	24	17	15	24	6
Nrip1	2804	3196	2709	2518	3048	3175	3077	2682
Nrip2	7	4	13	7	17	12	17	14
Nrip3	8	18	6	9	6	12	4	21
Nrm	186	212	189	231	191	191	159	179
Nrn1	22	32	15	10	33	35	16	48
Nrn1l	1	0	1	8	0	0	0	1
Nrp	10	7	13	18	3	7	15	14

Transcriptome sequencing yielded total genetic results for the MOD and APS groups, with a total of 15,936 variables

Continued from above

Nrp1	633	738	683	769	681	687	702	600
Nrp2	404	416	439	519	437	432	462	349
Nrros	73	113	107	111	133	118	63	93
Nrsn1	8	23	12	22	25	28	32	27
Nrsn2	9	2	1	0	0	1	2	1
Nrtn	156	134	174	174	131	153	117	139
Nrxn1	6	19	7	17	3	16	7	16
Nrxn2	48	63	42	21	41	19	37	26
Nrxn3	2	5	10	0	2	3	0	7
Nsa2	3026	3235	3288	3290	3408	3410	3345	3165
Nscme3l	1	0	0	5	0	4	0	1
Nsd1	4801	5322	4972	5133	5259	5383	5087	4696
Nsd2	1633	2037	1713	1885	1902	1868	1679	1597
Nsd3	2093	2133	2144	2167	2027	2095	1993	2020
Nsdhl	1420	1431	1605	1698	1237	1273	1245	1327
Nsf	2176	2476	2345	2220	2400	2465	2326	2385
Nsfl1c	1848	1874	2052	1978	2059	2162	1677	1896
Nsg1	80	46	91	90	92	90	60	67
Nsg2	68	95	85	80	83	108	84	77
Nsl1	195	221	254	213	224	223	185	194
Nsmaf	497	490	451	472	504	420	423	386
Nsmce1	972	970	916	946	894	1013	803	843
Nsmce2	395	398	431	408	433	382	346	346
Nsmce3	567	655	529	594	615	505	524	599
Nsmce4a	1754	2013	1783	1856	1715	1904	1632	1783
Nsmf	889	783	800	855	676	777	799	655
Nsrp1	478	526	460	502	587	602	535	526
Nsun2	2824	3293	2999	2976	3353	3366	2932	2735
Nsun3	777	760	643	663	716	646	647	710
Nsun4	650	671	752	613	607	691	490	579
Nsun5	333	294	299	296	418	347	366	295
Nsun6	223	260	271	252	282	270	264	270
Nsun7	0	2	1	0	1	3	1	0
Nt5c	2444	2828	2685	2581	2738	2706	2543	2442
Nt5c1a	1	0	0	0	2	2	0	1
Nt5c2	802	694	740	740	896	832	835	788
Nt5c3	1336	1476	1229	1273	1348	1438	1222	1328
Nt5c3b	247	208	232	198	289	203	168	172
Nt5dc1	478	622	533	566	507	642	525	454
Nt5dc2	51	36	68	38	24	20	36	37
Nt5dc3	98	97	152	132	134	142	105	108
Nt5e	1074	977	1110	1242	1115	1080	1188	1217
Nt5m	501	598	563	654	622	540	530	532
Ntan1	1137	1323	1187	1234	1157	1080	1222	1042
Ntf3	33	48	55	79	54	79	56	39
Ntf5	0	7	0	2	0	2	0	1
Nthl1	41	36	27	42	33	44	48	63
Ntm	1	1	0	0	1	3	0	1
Ntmt1	472	428	448	426	421	427	438	456
Ntn1	631	560	537	568	504	595	502	505
Ntn3	20	19	17	18	4	5	4	4
Ntn4	142	187	158	130	141	131	200	102
Ntn5	0	0	0	1	5	1	0	0
Ntng1	0	0	5	2	0	1	1	1
Ntng2	70	57	34	72	59	36	61	75
Ntpcr	344	323	281	349	311	283	288	348
Ntrk1	2	2	0	0	0	1	0	2
Ntrk2	10	9	13	4	9	7	7	8

Transcriptome sequencing yielded total genetic results for the MOD and APS groups, with a total of 15,936 variables

Continued from above

Ntrk3	25	8	14	11	20	11	24	17
Nts	176	140	138	220	142	188	183	174
Ntsr1	7	2	8	10	7	8	17	9
Ntsr2	0	0	0	1	0	0	1	0
Nuak1	66	43	56	55	67	28	71	76
Nuak2	2947	3095	2320	2256	2782	2974	2951	2847
Nub1	2729	2938	2635	2641	2805	2752	2854	2587
Nubp1	1563	1480	1452	1465	1220	1288	1357	1217
Nubp2	1439	1487	1352	1404	1380	1493	1328	1303
Nubpl	152	155	144	192	137	181	147	121
Nucb1	12355	13084	11870	11497	12685	12444	12273	12888
Nucb2	351	341	321	314	372	443	391	405
Nucks1	2038	2328	2070	2198	2201	2190	1950	1996
Nudc	1884	1891	1973	1816	2062	2048	1867	1973
Nudcd1	879	899	843	863	995	861	923	921
Nudcd2	1087	1122	1188	1043	1316	1228	1153	1133
Nudcd3	1739	1873	1637	1655	1571	1642	1505	1678
Nudt1	444	465	420	474	502	429	406	402
Nudt10	0	0	1	0	0	4	0	5
Nudt11	9	11	4	2	3	4	6	4
Nudt12	653	764	655	598	687	718	697	654
Nudt13	555	608	499	531	522	488	473	497
Nudt14	863	743	817	850	876	896	797	760
Nudt15	148	119	105	105	77	84	109	130
Nudt16	565	590	632	545	545	568	526	516
Nudt16l1	588	636	663	624	713	623	554	616
Nudt17	10	12	2	5	3	4	10	18
Nudt18	186	148	168	183	189	160	166	159
Nudt19	2825	2594	2827	2724	2794	2691	2559	2487
Nudt2	506	472	587	460	505	479	422	436
Nudt21	1326	1345	1361	1296	1343	1408	1293	1231
Nudt22	521	437	418	439	377	417	414	345
Nudt3	2648	2638	2683	2790	2754	2727	2565	2458
Nudt4	4474	4590	4480	4638	4210	4466	4729	4473
Nudt5	5646	6616	5450	5018	4967	5218	5413	5108
Nudt6	113	116	124	85	128	85	113	92
Nudt7	1102	1003	1057	1021	1197	1123	1061	977
Nudt8	297	358	335	440	358	379	361	414
Nudt9	1019	1033	979	953	1172	1115	969	1003
Nuf2	312	381	356	377	407	389	271	281
Nufip1	431	471	515	511	438	464	392	419
Nufip2	2451	2869	2551	2869	3005	3229	2996	3075
Nuggc	26	23	34	21	17	6	7	16
Numa1	3963	4616	4178	4267	4788	4924	4761	4389
Numb	4880	5051	4741	4689	4717	4788	4721	4481
Numbl	99	78	115	60	96	126	100	103
Nup107	795	833	796	778	913	853	818	765
Nup133	738	845	755	654	896	642	676	668
Nup153	1710	1888	1932	1661	1934	1920	1923	1656
Nup155	673	856	845	770	830	791	762	775
Nup160	852	960	961	828	958	840	901	863
Nup188	1084	1208	1073	1139	1196	985	1047	1043
Nup205	1953	2075	2012	2045	2229	2263	1877	1873
Nup210	813	1042	837	826	853	743	686	819
Nup210l	6	27	28	26	16	33	26	34
Nup214	1103	1438	1236	1112	1263	1230	1157	1111
Nup35	360	441	254	327	490	372	412	344
Nup37	372	499	385	428	478	472	387	410

Transcriptome sequencing yielded total genetic results for the MOD and APS groups, with a total of 15,936 variables

Continued from above

Nup43	253	229	286	205	217	290	207	202
Nup50	3226	3652	3295	3242	3502	3248	3321	3314
Nup54	571	618	660	614	597	652	543	515
Nup62	1398	1711	1483	1496	1405	1430	1359	1511
Nup62cl	1	1	1	0	1	4	5	0
Nup85	795	897	764	751	952	916	765	763
Nup88	1918	1921	1873	1992	1930	2065	1786	1816
Nup93	752	1034	883	786	906	913	726	824
Nup98	2185	2433	2217	2198	2500	2447	2313	2409
Nupl1	2211	2281	2226	2271	2451	2319	2256	2139
Nupl2	229	264	162	194	194	165	168	214
Nupr1	305	340	245	313	449	439	443	494
Nupr1l	4	3	12	2	1	0	1	6
Nus1	4995	5344	4993	4930	5815	5892	5792	5472
Nusap1	690	808	729	718	747	709	713	758
Nutf2	726	856	709	795	897	898	746	800
Nutm1	0	0	1	0	0	0	4	0
Nvl	1001	1090	1110	1113	1260	1210	1130	1154
Nwd1	0	9	2	2	1	2	0	1
Nxf1	2066	2440	2315	2189	2396	2255	2124	2222
Nxf2	1	1	0	4	0	1	0	0
Nxf3	0	0	0	0	0	0	1	0
Nxf7	108	103	111	131	127	98	126	84
Nxn	200	218	247	226	216	214	182	211
Nxnl1	0	0	1	0	0	0	0	0
Nxnl2	15	7	15	18	20	6	10	18
Nxpe2	3	7	0	2	14	2	0	1
Nxpe3	76	86	73	86	81	70	89	50
Nxpe4	25	44	32	43	38	32	9	25
Nxpe5	26	21	29	34	34	11	24	31
Nxph1	0	0	0	1	0	0	0	0
Nxph3	7	5	6	5	7	9	8	1
Nxph4	1	6	0	0	0	1	0	3
Nxt1	293	369	304	342	255	350	310	261
Nxt2	519	639	595	612	612	633	554	547
Nyap1	332	297	334	315	325	394	351	347
Nyap2	5	4	1	0	0	0	4	2
Nynrin	94	88	110	80	57	114	66	69
Nyx	11	22	25	26	7	17	31	36
Oacyl	30	14	11	10	10	7	3	5
Oaf	1235	1265	1276	1183	1237	1141	1134	1129
Oard1	1159	1447	1392	1316	1367	1307	1274	1196
Oas1a	4971	6569	6119	4745	6438	6056	5583	5784
Oas1c	179	220	282	182	177	220	215	202
Oas1d	1	4	7	13	2	6	3	0
Oas1e	236	198	250	246	161	189	191	185
Oas1f	5	1	7	9	3	10	6	10
Oas1g	3490	4151	3941	3126	4278	3924	3750	3792
Oas1h	2	0	0	1	0	4	0	1
Oas2	2223	3456	3185	1985	3589	2955	2527	2295
Oas3	5469	7136	6322	4603	6276	6186	5794	5286
Oasl1	7494	9412	8007	6013	7035	7222	6689	7059
Oasl2	12877	16743	14869	11396	14297	14236	13286	12365
Oat	10440	12005	12533	11074	13107	13231	11356	9623
Oaz1	13530	13149	13472	13162	13625	13666	12931	13269
Oaz2	785	820	845	960	751	874	824	838
Obi1	135	143	141	123	126	134	118	130
Obox2	1	0	0	0	0	0	0	0

Transcriptome sequencing yielded total genetic results for the MOD and APS groups, with a total of 15,936 variables

Continued from above

Obp2a	1	0	0	0	0	0	0	0
Obscn	15	10	4	21	11	3	4	5
Obsl1	28	49	53	49	73	86	28	42
Ocel1	569	475	546	608	517	548	515	468
Ociad1	4807	4792	4866	4772	4806	4766	4683	4551
Ociad2	12104	11971	12272	12058	9958	11354	10805	10524
Ocln	4493	5018	4790	4437	4587	5047	4999	4787
Ocm	126	156	132	125	178	244	275	184
Ocrl	289	295	350	387	271	301	261	277
Ocstamp	144	141	126	143	118	135	89	130
Odc1	5653	6036	5497	4862	6396	6031	5941	5830
Odf2	635	739	715	725	673	645	597	518
Odf2l	755	786	823	917	909	881	839	703
Odf3b	328	326	289	374	304	380	337	349
Odf3l1	2	7	2	1	9	4	2	1
Odf3l2	0	0	0	1	0	0	0	1
Odf4	0	0	0	0	0	1	0	0
Odr4	994	1043	1198	1103	1230	1357	1360	1276
Ofcc1	0	0	0	0	0	1	0	0
Ofd1	172	203	219	200	161	178	266	221
Oga	2680	3142	3154	2821	3203	3246	3121	2723
Ogdh	37457	40651	37560	36684	35233	36573	37947	35391
Ogdhl	43	38	50	35	31	17	30	39
Ogfod1	866	977	939	775	878	887	869	873
Ogfod2	601	570	553	641	648	635	546	535
Ogfod3	574	595	565	577	453	612	510	560
Ogfr	3448	4001	3608	3235	3233	3290	3067	3058
Ogfrl1	119	88	110	114	71	96	132	126
Ogg1	209	278	210	200	203	202	158	194
Ogn	300	359	331	368	384	457	323	285
Ogt	4144	4709	4447	4321	4140	4268	4010	3683
Oip5	150	144	178	180	181	166	131	143
Oit1	13207	13316	12604	13233	12628	13249	13026	12519
Oit3	25	17	11	24	25	4	26	13
Ola1	1701	1902	1996	1903	1959	1882	1677	1716
Olfm1	138	127	140	91	124	73	122	87
Olfm2	3	5	6	4	17	8	3	12
Olfm3	0	0	0	0	4	1	0	0
Olfm4	20031	22735	22928	21502	19842	20179	19377	19175
Olfml1	22	34	22	27	54	32	22	11
Olfml2a	32	22	29	31	31	39	17	14
Olfml2b	80	65	86	101	64	76	66	34
Olfml3	275	279	310	245	276	225	248	262
Olfr1033	36	44	39	50	25	32	57	51
Olfr1034	0	0	1	1	2	1	4	1
Olfr1043	0	1	0	0	0	0	0	0
Olfr111	1	0	4	3	2	4	1	0
Olfr1342	0	0	1	0	0	1	0	0
Olfr1368	0	0	0	1	0	1	0	0
Olfr1392	4	0	0	0	0	0	0	2
Olfr1393	3	4	10	3	8	9	2	4
Olfr1396	1	1	0	0	0	0	0	0
Olfr1444	0	0	1	0	0	0	0	0
Olfr1532-|	0	0	1	0	0	1	0	0
Olfr156	0	0	0	0	0	0	0	1
Olfr1564	0	0	1	0	0	0	0	0
Olfr157	0	0	0	0	1	0	0	0
Olfr165	14	29	26	29	22	30	27	26

Transcriptome sequencing yielded total genetic results for the MOD and APS groups, with a total of 15,936 variables

Continued from above

Olfr18	1	0	0	0	0	0	0	0
Olfr20	0	1	1	0	0	0	0	0
Olfr267	0	0	0	1	0	0	0	0
Olfr286	1	0	0	0	1	0	1	0
Olfr287	1	0	1	1	0	0	0	1
Olfr323	0	0	0	0	1	0	0	0
Olfr339	0	0	0	0	1	0	0	0
Olfr433	1	0	0	0	0	0	0	0
Olfr49	1	0	0	0	0	0	0	0
Olfr520	0	0	0	1	0	0	0	0
Olfr558	5	2	5	13	11	5	5	9
Olfr56	1	1	0	6	5	0	1	0
Olfr561	0	1	0	0	0	0	0	0
Olfr60	0	0	0	1	1	1	0	0
Olfr651	1	0	0	0	0	0	0	0
Olfr658	5	2	0	0	0	2	1	0
Olfr713	0	0	0	0	0	2	0	0
Olfr750	0	0	0	0	0	0	0	1
Olfr78	1	5	1	0	4	3	6	5
Olfr811	0	0	0	0	0	0	6	0
Olfr827	0	0	0	0	0	0	0	1
Olfr920	1	3	5	6	3	6	1	3
Olfr923	0	1	0	0	0	0	0	0
Olfr99	0	0	0	1	0	0	0	0
Olig1	5	2	0	1	0	0	0	1
Olr1	4	0	1	0	0	0	0	1
Oma1	975	951	986	896	982	1017	1008	980
Omd	0	1	0	0	1	0	0	0
Omg	2	0	1	0	2	1	3	1
Omp	21	20	28	11	46	16	14	38
Onecut2	3231	4092	3721	3580	4070	4418	3879	3507
Onecut3	16	9	20	7	2	19	6	20
Ooep	4	5	1	0	1	4	0	1
Oosp1	0	5	2	1	0	0	0	14
Opa1	4446	4458	4341	4604	4391	4302	4146	3973
Opa3	1326	1734	1478	1458	1488	1597	1479	1437
Opcml	2	6	7	2	5	0	2	8
Ophn1	116	151	107	121	138	150	146	120
Oplah	650	693	526	634	655	666	684	679
Opn1mw	0	1	0	0	0	0	0	0
Opn1sw	5	0	4	0	0	1	0	0
Opn3	143	128	148	99	241	151	196	135
Opn4	0	0	0	0	1	0	0	0
Opn5	0	0	0	0	0	4	0	0
Oprd1	1	25	11	12	14	9	9	8
Oprk1	2	1	0	6	5	3	1	2
Oprl1	1	2	2	6	1	1	10	4
Optc	64	68	94	97	87	104	113	45
Optn	7148	7809	6925	6703	6423	7354	7185	6803
Orai1	2178	2027	2008	1985	1641	1729	1731	1766
Orai2	864	943	1028	962	919	1052	1060	918
Orai3	260	332	357	324	282	332	239	306
Orc1	63	157	128	126	163	107	104	124
Orc2	417	437	430	413	426	421	427	386
Orc3	767	832	780	773	912	988	826	794
Orc4	661	782	739	826	863	726	790	788
Orc5	390	487	434	397	456	405	413	389
Orc6	357	319	376	350	389	403	325	324

Transcriptome sequencing yielded total genetic results for the MOD and APS groups, with a total of 15,936 variables

Continued from above

Orm1	4	18	7	1	15	8	11	7
Orm2	1	2	5	1	1	0	3	0
Orm3	0	0	0	0	0	1	0	0
Ormdl1	718	819	788	746	874	913	957	867
Ormdl2	1406	1648	1511	1469	1839	1563	1659	1480
Ormdl3	3682	4388	3879	3629	4751	4906	4841	4382
Os9	9624	10629	9792	8998	10369	10564	9556	9489
Osbp	4528	4731	4582	4647	4910	5183	4956	4741
Osbp2	1	0	1	1	0	4	5	1
Osbpl10	932	1101	902	817	1049	1057	1123	937
Osbpl11	1221	1189	1352	1450	1188	1199	1220	1168
Osbpl1a	340	362	342	297	402	390	420	428
Osbpl2	1367	1397	1555	1407	1618	1558	1443	1421
Osbpl3	1596	1619	1553	1480	1885	1945	2173	1989
Osbpl5	943	841	814	988	627	634	687	624
Osbpl6	1798	2003	1921	1902	2017	2220	2200	2008
Osbpl7	1305	1364	1281	1416	1281	1169	1128	1087
Osbpl8	231	406	273	308	302	266	265	274
Osbpl9	1547	1678	1599	1664	1672	1788	1808	1635
Oscar	1	6	1	0	0	0	0	1
Oscp1	48	34	34	32	25	37	33	16
Oser1	649	611	687	539	649	692	660	621
Osgep	670	664	661	599	621	612	557	522
Osgepl1	268	263	281	267	268	331	314	287
Osgin1	2885	2811	2640	2734	2805	2763	2755	2746
Osgin2	94	85	86	67	109	120	87	112
Osm	8	0	2	9	0	1	1	6
Osmr	254	249	235	177	228	295	222	153
Osr1	26	48	28	46	41	33	59	56
Osr2	122	111	122	166	152	197	176	200
Ost4	3298	3569	3259	3579	3509	3568	3405	3361
Ostc	5115	5500	5221	4969	5900	5989	5669	5523
Ostf1	3670	3771	3683	3557	3685	3706	3314	3436
Ostm1	525	615	516	602	617	530	561	552
Otc	4962	5791	5371	5147	5384	5583	5585	5132
Otoa	1	0	4	1	5	1	3	0
Otof	3	7	3	4	0	0	1	1
Otog	0	0	0	1	0	0	8	2
Otogl	12	0	9	6	5	1	1	4
Otop1	0	0	0	1	0	0	0	0
Otop2	1	0	0	1	1	1	3	0
Otop3	486	512	347	330	481	488	376	261
Otos	0	0	4	0	0	0	1	0
Otub1	2294	2462	2404	2354	2202	2044	2083	2121
Otub2	25	11	24	20	29	21	20	33
Otud1	48	40	60	47	30	31	40	61
Otud3	2645	2548	2320	2778	2430	2272	2144	2383
Otud4	2073	2306	2404	2412	2311	2405	2228	2222
Otud5	3402	3775	3430	3444	3539	3681	3266	3111
Otud6b	1070	1043	919	1000	1132	1240	1065	1077
Otud7a	1	2	4	5	0	2	8	1
Otud7b	2016	2216	2082	1782	2126	2181	2221	1985
Otulin	699	732	741	677	717	729	648	637
Otulinl	258	225	197	240	278	204	168	204
Otx1	0	0	0	0	0	1	0	0
Ovca2	339	309	318	320	323	358	332	343
Ovgp1	17	22	5	7	6	7	7	5
Ovol1	2213	2520	2246	2250	2300	2538	2500	2286

Transcriptome sequencing yielded total genetic results for the MOD and APS groups, with a total of 15,936 variables

Continued from above

Ovol2	670	642	763	689	659	622	627	613
Ovol3	1	2	2	0	0	7	0	2
Oxa1l	2705	2672	2849	2811	2536	2601	2391	2483
Oxct1	23113	21193	21301	24920	19186	20008	20616	20117
Oxct2a	6	7	17	10	18	22	19	18
Oxct2b	11	19	18	7	0	9	5	5
Oxld1	367	337	336	328	322	305	322	273
Oxnad1	1207	1290	1312	1351	1434	1406	1287	1265
Oxr1	875	779	845	785	802	777	864	867
Oxsm	740	792	666	678	711	746	833	821
Oxsr1	2668	2883	2632	2648	3055	2845	2931	2913
Oxtr	1	4	4	2	2	10	2	8
P2rx1	306	390	308	301	244	289	261	306
P2rx2	25	33	35	23	15	24	23	12
P2rx3	3	14	5	2	7	5	8	8
P2rx4	1820	2042	1916	2060	1906	1696	1798	1716
P2rx5	2	3	0	1	3	2	1	2
P2rx6	27	24	16	35	33	43	29	10
P2rx7	173	227	212	212	199	211	198	167
P2ry1	1276	1330	1374	1278	1333	1320	1376	1216
P2ry10	26	31	40	31	19	40	13	28
P2ry10b	1	1	4	10	1	1	6	1
P2ry12	47	27	41	36	45	49	37	37
P2ry13	129	138	115	112	181	124	156	127
P2ry14	221	226	224	273	229	248	179	204
P2ry2	353	411	286	265	374	460	540	435
P2ry4	5	9	14	7	23	11	6	7
P2ry6	184	223	174	170	225	188	208	210
P3h1	111	78	120	127	84	103	88	79
P3h2	50	47	76	79	45	29	50	42
P3h3	159	240	142	178	143	154	129	169
P3h4	84	62	92	79	68	57	72	93
P4ha1	272	248	291	234	394	369	324	303
P4ha2	20	68	26	35	50	63	51	45
P4ha3	6	0	0	6	0	0	1	0
P4hb	112583	127001	111595	102760	124548	126399	122536	118683
P4htm	20	20	25	15	12	20	15	14
Pa2g4	4142	4576	4199	4154	4454	4360	3891	3921
Pabpc1	22383	23122	23097	22707	23419	23595	21024	21618
Pabpc1l	1	4	8	6	16	2	1	6
Pabpc1l2a	1	2	1	1	0	0	0	1
Pabpc1l2b	0	0	0	1	1	0	0	0
Pabpc4	2145	2214	2266	2147	2093	2193	1980	1921
Pabpc4l	4	1	0	5	3	0	6	2
Pabpc5	0	0	0	0	1	0	0	0
Pabpn1	229	241	284	247	202	235	161	236
Pabpn1l	4	0	1	0	0	2	1	0
Pacc1	87	79	112	79	98	117	100	47
Pacrg	2	2	0	0	5	0	1	1
Pacrgl	189	214	200	164	198	148	207	185
Pacs1	181	225	214	185	152	182	163	183
Pacs2	1266	1355	1206	1294	1146	1176	1228	1126
Pacsin1	15	16	26	13	2	10	11	16
Pacsin2	5919	5967	5764	5957	5536	5642	5711	5566
Pacsin3	20	35	34	18	42	25	28	10
Padi1	9	0	2	5	0	0	1	0
Padi2	69	67	49	48	44	74	34	40
Padi3	0	0	0	0	0	0	1	0

Transcriptome sequencing yielded total genetic results for the MOD and APS groups, with a total of 15,936 variables

Continued from above

Padi4	1	5	3	4	2	1	0	0
Paf1	1254	1464	1288	1315	1465	1502	1458	1296
Pafah1b1	7467	8070	7427	7366	8367	8949	8898	8089
Pafah1b2	1928	2113	1992	1917	2146	2015	1942	1962
Pafah1b3	1411	1562	1577	1610	1483	1691	1405	1582
Pafah2	4156	4191	4070	4219	3982	3822	3981	4130
Pag1	1276	1362	1338	1343	1193	1340	1295	1281
Pagr1a	767	847	856	881	939	819	744	865
Pah	14	14	6	7	24	24	27	30
Paics	1097	1095	1121	1022	1124	1270	1005	1020
Paip1	1492	1754	1760	1544	1849	2062	1848	1621
Paip2	1599	1738	1736	1612	1914	1769	1660	1898
Paip2b	548	617	663	661	626	682	551	680
Pak1	4715	4706	4360	4275	4606	4657	4555	4280
Pak1ip1	1645	1780	1844	1809	1645	1865	1623	1800
Pak2	4422	4643	4516	4316	4524	4856	4707	4296
Pak3	25	32	30	36	36	16	28	6
Pak4	3415	3667	3475	3264	3434	3576	3216	3093
Pak5	0	1	0	0	1	1	0	0
Pak6	518	312	390	651	151	175	214	217
Pakap	357	410	400	366	418	415	357	335
Palb2	127	151	112	122	171	105	130	122
Pald1	130	82	117	126	142	101	97	128
Palld	2998	2915	2975	3128	2741	2709	2642	2636
Palm	299	342	400	347	293	364	287	302
Palm3	1	0	1	1	0	0	0	4
Palmd	38	26	34	20	26	33	38	17
Pam	1181	1203	1319	1377	1045	963	1093	1038
Pam16	490	538	542	571	546	545	569	521
Pamr1	133	186	186	216	180	146	180	143
Pan2	1145	1208	1173	1095	1077	1006	1174	1159
Pan3	940	1054	1013	1096	1123	1132	1161	1016
Pank1	887	902	1017	890	955	1020	936	832
Pank2	1907	2113	2128	2074	1875	2169	1971	1794
Pank3	8479	9852	10164	9702	11423	12446	12130	10968
Pank4	590	500	604	531	579	535	599	541
Panx1	2095	2163	2031	1969	2060	1957	2012	1879
Panx2	32	30	29	20	21	25	17	50
Paox	705	734	665	593	764	782	648	707
Papln	402	535	533	530	566	515	372	502
Papola	5970	6264	6043	5958	6808	6954	6507	6039
Papolb	0	0	1	0	0	0	0	4
Papolg	523	521	515	568	575	599	567	529
Pappa	76	104	110	68	132	105	129	131
Pappa2	18	23	16	20	13	9	13	17
Papss1	1997	2308	2322	2285	2319	2495	2482	2055
Papss2	97581	108158	93813	90877	100687	108574	108365	104764
Paqr3	42	49	59	56	45	23	39	27
Paqr4	4172	3969	4029	4345	3796	3579	3682	3763
Paqr5	74	82	118	49	50	90	87	53
Paqr6	4	11	14	7	2	8	1	12
Paqr7	829	1199	842	586	1615	1895	1436	1033
Paqr8	194	228	211	205	335	274	238	291
Paqr9	317	466	365	308	395	411	440	386
Pard3	1961	2078	1876	1932	2129	2093	2063	2058
Pard3b	515	618	567	592	470	556	501	442
Pard6a	128	90	109	75	113	107	72	84
Pard6b	2204	2201	2015	2157	2129	2173	2494	2146

Transcriptome sequencing yielded total genetic results for the MOD and APS groups, with a total of 15,936 variables

Continued from above

Pard6g	71	86	86	137	75	78	75	60
Parg	1219	1350	1107	1210	1291	1446	1260	1207
Park7	2893	2933	2919	2841	2966	2877	2677	2828
Parl	1433	1497	1582	1523	1439	1457	1315	1351
Parm1	489	565	566	483	715	758	591	619
Parn	648	635	708	684	624	627	565	628
Parp1	1169	1381	1151	1310	1296	1304	1131	1310
Parp10	2541	2823	2699	2747	2076	2174	2157	2116
Parp11	727	1096	866	654	903	1001	775	704
Parp12	8599	10092	9021	7640	8663	9266	8515	7737
Parp14	9376	11588	10149	8739	9136	9719	9203	8241
Parp16	480	573	624	523	651	717	580	597
Parp2	692	713	724	713	705	783	684	713
Parp3	771	775	793	952	784	722	628	793
Parp4	2249	2683	2344	2290	2148	2399	2560	2293
Parp6	485	549	457	450	508	496	563	443
Parp8	89	51	66	76	79	59	72	51
Parp9	6700	8214	7156	6465	6307	6868	6408	5840
Parpbp	147	216	186	240	170	191	180	168
Pars2	260	285	281	258	242	266	257	228
Parva	1618	1785	1988	1778	1834	1976	1448	1530
Parvb	133	125	131	127	80	111	68	73
Parvg	37	29	34	56	46	25	38	33
Pask	190	206	204	214	225	181	189	156
Pate2	0	0	1	0	0	0	0	0
Pate4	0	0	0	0	0	1	0	3
Pate6	5	0	5	0	1	0	4	1
Pate7	0	1	1	0	0	0	0	0
Patj	4270	4366	4067	4082	3985	4522	4357	3893
Patl1	1430	1433	1319	1426	1506	1295	1196	1210
Patl2	4	0	4	0	0	1	0	0
Patz1	300	319	291	244	277	289	266	278
Pawr	1367	1448	1389	1432	1481	1502	1515	1389
Pax4	8	12	20	10	17	12	7	13
Pax5	0	0	0	0	1	0	0	0
Pax6	29	31	39	22	42	21	39	28
Pax8	256	246	294	244	262	228	299	325
Paxbp1	1162	1160	1119	1381	1336	1317	1274	1198
Paxip1	1122	1164	1066	1083	1151	1109	1051	1092
Paxx	504	494	513	465	497	584	459	506
Pbdc1	460	552	550	438	582	641	582	491
Pbk	617	714	715	631	767	706	656	674
Pbld1	192	311	298	227	387	422	415	343
Pbld2	3972	4254	4109	4108	3789	4068	4092	3693
Pbrm1	1890	2103	2189	2272	2001	2222	2091	1836
Pbx1	628	768	625	687	585	552	518	503
Pbx2	1271	1276	1106	1165	1092	1250	1106	1092
Pbx3	180	177	154	167	178	194	127	165
Pbx4	14	32	28	24	34	29	37	47
Pbxip1	3254	3219	3380	3411	3125	3087	2910	2901
Pcbd1	925	797	1003	788	905	884	818	765
Pcbd2	455	505	462	471	509	529	385	458
Pcbp1	13605	13991	13701	13216	13561	14046	12817	12613
Pcbp2	8008	8445	8532	8042	7937	7839	7485	7559
Pcbp3	56	61	59	46	77	56	48	46
Pcbp4	161	130	141	155	168	171	152	169
Pcca	828	994	925	939	916	875	885	888
Pccb	4363	4480	4903	4675	4382	4630	4376	4318

Transcriptome sequencing yielded total genetic results for the MOD and APS groups, with a total of 15,936 variables

Continued from above

Pcdh1	5388	5729	5073	5015	5319	5663	5684	5195
Pcdh10	15	4	15	14	13	24	13	4
Pcdh12	94	110	83	113	119	102	108	103
Pcdh15	4	0	1	5	2	0	2	2
Pcdh17	46	104	83	33	44	55	68	68
Pcdh18	79	72	44	82	50	74	69	72
Pcdh19	57	81	57	56	64	53	52	74
Pcdh20	10	23	9	12	9	7	12	14
Pcdh7	254	306	342	268	261	318	252	216
Pcdh8	12	25	8	13	9	10	7	7
Pcdh9	5	24	11	9	5	16	2	13
Pcdha1	0	1	0	0	0	0	0	0
Pcdha10	0	0	1	1	0	1	0	0
Pcdha11	0	0	0	0	1	1	1	0
Pcdha12	4	4	2	0	0	0	1	2
Pcdha2	0	4	1	1	0	0	0	0
Pcdha3	0	4	0	4	1	0	1	0
Pcdha5	0	0	0	0	0	2	0	0
Pcdha9	0	0	4	0	0	0	0	0
Pcdhac2	7	3	0	3	5	6	2	4
Pcdhb10	11	5	3	9	14	5	6	2
Pcdhb11	1	3	12	8	2	12	7	3
Pcdhb12	13	5	3	5	6	14	6	5
Pcdhb13	5	8	2	2	1	1	2	3
Pcdhb14	7	3	18	2	18	11	4	1
Pcdhb15	0	1	2	1	4	1	0	6
Pcdhb16	12	19	27	21	18	15	11	21
Pcdhb17	25	37	46	15	35	37	34	42
Pcdhb18	3	1	2	10	10	5	14	4
Pcdhb19	8	4	19	13	11	4	3	9
Pcdhb2	1	0	0	1	0	1	0	0
Pcdhb20	13	9	7	16	26	9	3	12
Pcdhb21	1	5	9	2	1	12	2	0
Pcdhb22	26	6	22	8	2	4	19	4
Pcdhb3	5	1	3	1	2	5	5	0
Pcdhb4	8	6	14	2	2	1	9	5
Pcdhb5	6	11	3	6	1	3	3	8
Pcdhb6	0	0	2	1	6	0	0	2
Pcdhb7	17	4	1	1	4	6	1	0
Pcdhb8	14	4	0	6	5	1	8	5
Pcdhb9	10	1	4	17	15	9	18	8
Pcdhga1	1	7	3	2	0	2	5	0
Pcdhga10	16	10	21	15	16	21	16	9
Pcdhga11	12	12	7	3	9	12	1	2
Pcdhga12	4	5	20	0	8	0	17	5
Pcdhga2	5	2	0	8	2	8	5	0
Pcdhga3	4	2	3	2	2	10	12	6
Pcdhga4	6	12	5	14	3	10	5	1
Pcdhga5	3	7	19	10	8	4	13	12
Pcdhga6	3	5	17	2	8	2	9	3
Pcdhga7	4	7	6	10	6	17	9	5
Pcdhga8	0	3	1	10	9	2	4	3
Pcdhga9	13	2	0	3	5	2	1	1
Pcdhgb1	15	3	4	11	7	0	0	2
Pcdhgb2	19	15	17	13	14	6	19	3
Pcdhgb4	4	13	9	5	2	10	14	1
Pcdhgb5	33	8	14	6	16	5	11	8
Pcdhgb6	14	17	43	26	36	34	28	25

Transcriptome sequencing yielded total genetic results for the MOD and APS groups, with a total of 15,936 variables

Continued from above

Pcdhgb7	17	17	16	34	21	29	21	18
Pcdhgc3	184	263	276	210	297	196	173	135
Pcdhgc4	6	8	18	39	28	14	36	14
Pcdhgc5	13	0	2	1	0	0	1	2
Pced1a	144	158	160	127	212	220	206	228
Pced1b	23	30	42	38	38	27	24	42
Pcf11	1179	1308	1394	1292	1402	1737	1536	1263
Pcgf1	149	129	141	102	144	109	130	105
Pcgf2	226	234	238	218	187	173	169	175
Pcgf3	952	967	973	988	915	968	1002	982
Pcgf5	1543	1506	1465	1375	1385	1609	1451	1320
Pcgf6	351	471	395	383	451	451	457	416
Pcid2	624	652	711	637	773	661	653	585
Pcif1	1473	1513	1464	1360	1293	1264	1272	1336
Pck1	5737	6352	7047	6489	4418	5415	6511	5384
Pck2	1145	1186	1040	1072	1278	1110	1056	1105
Pclaf	1349	1562	1427	1362	1516	1624	1348	1244
Pclo	21	30	50	27	19	38	21	18
Pcm1	1259	1480	1350	1413	1556	1741	1590	1377
Pcmt1	1526	1426	1547	1587	1554	1631	1534	1473
Pcmtd1	911	950	872	975	876	967	1135	942
Pcmtd2	1279	1401	1564	1236	1185	1397	1406	1186
Pcna	3521	3713	3587	3566	4049	4247	3556	3662
Pcnp	1903	2139	2073	1876	2132	2131	2021	1929
Pcnt	1131	1393	1318	1250	1112	1134	1132	1204
Pcnx	4555	4721	4808	4823	4426	4717	4616	4151
Pcnx2	1	2	1	1	5	12	4	6
Pcnx3	2818	3010	2991	2851	3062	3041	2953	2735
Pcnx4	62	59	44	88	61	84	100	81
Pcolce	334	330	368	356	334	363	273	301
Pcolce2	15	7	13	14	4	5	10	19
Pcp2	0	0	1	2	0	0	0	0
Pcp4	92	116	113	120	93	95	94	87
Pcp4l1	305	226	237	331	292	391	374	330
Pcsk1	384	390	373	352	386	343	401	360
Pcsk1n	144	104	108	116	109	86	153	84
Pcsk2	46	17	25	27	25	34	46	24
Pcsk4	225	235	258	313	269	255	218	249
Pcsk5	4888	5447	4820	4747	5289	5720	5329	4874
Pcsk6	1196	734	970	1397	505	598	497	421
Pcsk7	2974	3177	3175	2951	3049	3006	3005	2700
Pcsk9	1526	1741	2329	2193	1671	1553	1266	1618
Pctp	486	509	473	525	508	483	427	455
Pcx	4358	4192	3877	4250	3337	3539	3619	3424
Pcyox1	2090	2473	2490	2310	2692	2668	2325	2525
Pcyox1l	116	83	105	105	164	133	142	126
Pcyt1a	14871	14857	13212	14100	12665	13295	14021	12349
Pcyt1b	20	24	23	8	26	23	22	11
Pcyt2	8545	8840	8754	8495	7856	8050	8477	7639
Pdap1	2875	2772	2732	2396	2769	2821	2619	2693
Pdcd1	16	6	8	7	12	6	1	2
Pdcd10	4189	4261	3918	3953	3828	4145	4247	3952
Pdcd11	1385	1541	1293	1429	1630	1671	1500	1424
Pdcd1lg2	29	66	40	55	26	65	40	40
Pdcd2	478	562	471	460	524	544	486	486
Pdcd2l	489	453	428	369	407	398	374	321
Pdcd4	2398	2773	2690	2784	2619	2714	2603	2436
Pdcd5	720	639	703	667	654	710	638	702

Transcriptome sequencing yielded total genetic results for the MOD and APS groups, with a total of 15,936 variables

Continued from above

Pdcd6	7312	7956	7223	7514	7568	7960	7638	7243
Pdcd6ip	11826	12513	11281	11388	11887	12079	11794	11304
Pdcd7	505	496	507	520	557	495	467	577
Pdcl	1371	1412	1318	1374	1417	1419	1395	1271
Pdcl3	2496	2418	2580	2550	2430	2577	2705	2615
Pde10a	26	16	28	16	35	23	37	25
Pde11a	68	59	83	118	58	69	72	66
Pde12	1328	1440	1279	1235	1508	1588	1463	1242
Pde1a	77	105	85	80	134	98	85	71
Pde1b	176	184	210	196	154	159	137	180
Pde1c	22	38	52	54	29	33	32	45
Pde2a	572	557	567	654	574	538	500	534
Pde3a	2106	2331	2366	2419	2323	2321	2244	2175
Pde3b	223	231	317	161	219	297	169	220
Pde4a	104	58	63	67	74	86	77	71
Pde4b	229	234	219	161	187	174	178	159
Pde4c	31	29	28	24	23	17	39	11
Pde4d	505	584	487	517	554	585	528	494
Pde4dip	3614	4172	4105	3898	4709	4416	4607	4379
Pde5a	1115	1371	1231	1165	1215	1359	1234	1258
Pde6a	33	37	33	57	36	10	10	24
Pde6b	1	0	0	0	2	0	0	0
Pde6c	0	0	0	0	0	1	0	0
Pde6d	423	488	534	505	474	411	470	456
Pde6g	0	8	11	0	0	0	6	0
Pde7a	678	488	561	763	417	410	410	424
Pde7b	84	108	93	56	59	48	80	70
Pde8a	103	148	149	145	144	134	122	126
Pde8b	293	240	283	349	268	305	310	288
Pde9a	4041	4305	4071	3860	3313	3897	4085	3722
Pdf	669	637	686	618	727	652	540	633
Pdgfa	1064	1010	1004	1092	999	1062	1025	930
Pdgfb	126	123	129	118	155	103	134	135
Pdgfc	392	459	405	389	274	281	342	296
Pdgfd	53	42	36	29	39	48	36	23
Pdgfra	1063	1242	1216	1159	1204	967	983	923
Pdgfrb	597	579	620	602	550	463	442	436
Pdgfrl	0	8	5	0	5	1	10	8
Pdha1	12050	11871	11842	12599	11786	11984	11671	11319
Pdhb	4584	4773	4712	4972	5047	5278	5010	4734
Pdhx	1320	1333	1321	1403	1362	1268	1308	1159
Pdia2	194	226	116	37	778	671	405	639
Pdia3	22942	25593	23870	22314	27072	26442	23847	24020
Pdia4	3763	4589	4079	3786	5447	5270	4581	4706
Pdia5	650	891	791	731	914	924	815	801
Pdia6	7207	8272	7937	7440	9483	8912	7652	8292
Pdik1l	659	681	622	647	708	630	677	637
Pdilt	0	0	4	4	7	2	1	2
Pdk1	2518	2606	2507	2691	2712	2721	2573	2568
Pdk2	3621	3744	3903	3676	3904	3795	4023	3779
Pdk3	1011	1232	1108	1044	1288	1218	1207	1126
Pdk4	496	816	862	596	870	878	918	955
Pdlim1	4587	5020	5142	5017	5432	5325	4815	4664
Pdlim2	2613	2811	2228	2041	2763	2912	3083	2688
Pdlim3	752	649	865	814	689	795	883	809
Pdlim4	40	74	86	82	99	68	65	54
Pdlim5	2980	3357	2948	3226	3477	3402	3690	3204
Pdlim7	429	504	505	463	480	455	455	560

Transcriptome sequencing yielded total genetic results for the MOD and APS groups, with a total of 15,936 variables

Continued from above

Pdp1	98	154	138	96	121	131	117	112
Pdp2	1012	1228	1094	912	1239	1339	1311	1144
Pdpk1	2845	2954	2908	3145	3387	3282	3330	3218
Pdpn	161	157	195	196	171	173	215	160
Pdpr	594	557	656	744	585	615	601	564
Pdrg1	422	494	448	483	482	559	505	478
Pds5a	3389	3765	3427	3349	3848	3833	3861	3371
Pds5b	552	664	473	582	707	639	596	604
Pdss1	1543	1595	1666	1643	1563	1487	1487	1388
Pdss2	890	1007	914	994	1074	974	904	879
Pdx1	1081	1201	1002	994	1178	1174	908	940
Pdxdc1	9418	10164	9413	8943	10641	10842	10247	10063
Pdxk	3188	3526	3519	3702	3207	3362	3294	3087
Pdxp	26	21	12	27	39	18	11	41
Pdyn	2	0	4	4	0	4	0	0
Pdzd11	1551	1621	1618	1616	1646	1749	1571	1562
Pdzd2	664	566	626	696	355	446	446	405
Pdzd3	2454	2357	2303	2196	2287	2532	2442	2390
Pdzd4	16	43	14	34	12	31	18	37
Pdzd7	33	32	34	32	42	29	48	33
Pdzd8	2496	2957	2544	2826	2860	3063	2967	2689
Pdzd9	55	45	42	61	33	36	30	28
Pdzk1	6188	7315	5926	5157	7235	8168	8675	6994
Pdzk1ip1	105	72	83	90	92	93	105	118
Pdzrn3	207	190	214	195	179	202	154	175
Pdzrn4	0	1	1	2	0	11	2	3
Pea15a	541	608	657	597	622	621	491	575
Peak1	2840	3402	2872	2944	3541	3348	3338	2947
Pear1	125	176	153	192	172	128	109	147
Pebp1	4179	4593	4595	4304	4616	4626	3994	4368
Pebp4	0	0	0	0	1	0	0	0
Pecam1	769	880	907	886	757	776	730	769
Pecr	281	293	290	262	288	285	242	318
Pef1	3950	3948	3721	3708	3589	3605	3595	3521
Peg10	5	5	8	5	5	7	4	2
Peg12	3	6	0	3	15	2	3	1
Peg3	116	156	152	202	194	160	139	112
Peli1	2832	3215	2944	2873	2625	2812	2849	2592
Peli2	51	75	92	57	65	74	70	80
Peli3	5	5	26	5	4	8	18	3
Pelo	617	682	609	610	742	670	578	612
Pelp1	729	783	726	625	735	718	574	610
Pemt	55	73	73	57	71	92	44	76
Penk	38	27	25	27	28	43	35	24
Pepd	13077	14664	12951	11909	12960	14919	15689	14117
Per1	570	860	1248	879	728	703	751	706
Per2	460	446	584	550	752	734	680	637
Per3	188	140	193	198	137	207	121	133
Percc1	14	1	5	2	15	8	15	6
Perm1	52	78	72	69	69	71	71	75
Perp	7037	7311	6998	6798	7719	7978	7231	7293
Pes1	1694	1896	1911	1706	2027	1951	1880	1741
Pet100	403	417	441	482	426	466	435	412
Pex1	715	675	651	664	688	648	690	714
Pex10	369	315	320	329	327	319	289	311
Pex11a	2289	2609	2650	2387	2669	2881	2856	2681
Pex11b	694	669	630	707	712	622	616	543
Pex11g	622	590	602	573	640	671	619	629

Transcriptome sequencing yielded total genetic results for the MOD and APS groups, with a total of 15,936 variables

Continued from above

Pex12	336	355	237	265	359	330	308	306
Pex13	1488	1637	1515	1594	1699	1786	1672	1506
Pex14	1467	1498	1468	1500	1535	1437	1642	1535
Pex16	2912	3200	2688	2618	2841	2775	2761	2522
Pex19	5311	5622	5377	5332	5226	5276	5153	4901
Pex2	1934	2193	1989	1951	2236	2463	2349	2098
Pex26	933	924	955	871	923	971	950	912
Pex3	644	726	712	609	613	681	630	641
Pex5	2244	2336	2216	2159	2229	2218	2202	2127
Pex5l	28	44	12	41	19	32	27	8
Pex6	2571	2723	2837	2663	2987	3159	2752	2601
Pex7	1295	1438	1372	1378	1385	1462	1333	1193
Pf4	84	64	72	89	90	88	130	67
Pfas	607	704	628	574	597	675	572	631
Pfdn1	1062	1265	1079	1194	1226	1151	1084	1144
Pfdn2	877	894	950	909	1023	961	875	859
Pfdn4	352	391	395	396	407	418	416	348
Pfdn5	2261	2293	2061	2218	2220	2162	2092	1960
Pfdn6	1097	1090	1093	1120	1032	1115	939	916
Pfkfb1	46	74	70	57	44	56	57	71
Pfkfb2	2416	2505	2539	2546	2750	2894	2985	2796
Pfkfb3	2289	3094	2620	1806	3590	3875	3888	3995
Pfkfb4	8115	7889	7241	7611	6977	7198	7797	7206
Pfkl	2606	2928	3018	3024	3369	3089	2867	3013
Pfkm	574	471	477	520	379	442	322	432
Pfkp	16139	17437	15615	15873	15868	16578	15946	15451
Pfn1	27271	28971	28204	28085	26147	27891	25836	24548
Pfn2	63	78	84	77	88	60	62	71
Pfn4	0	0	0	4	0	0	0	0
Pga5	0	0	0	1	0	0	0	4
Pgam1	11506	10790	11920	12106	11046	11021	10342	10894
Pgam2	11	8	25	9	10	10	10	18
Pgam5	1088	1102	1090	977	1087	1027	1008	1113
Pgap1	1758	1484	1687	2008	1240	1271	1318	1234
Pgap2	923	1145	1156	1147	1078	1011	991	1060
Pgap3	1153	1166	1190	1124	1125	1106	1094	959
Pgap4	1387	1588	1479	1566	1377	1468	1365	1284
Pgap6	8462	8621	8285	7705	8892	8700	8221	8185
Pgbd1	32	16	16	14	15	17	23	20
Pgbd5	19	27	29	16	33	27	28	38
Pgc	14	19	14	19	49	11	25	21
Pgd	22389	22764	22935	23583	22584	22451	20332	21247
Pgf	18	22	20	23	31	24	53	43
Pgghg	534	650	511	539	543	528	376	463
Pggt1b	1132	1432	1197	1289	1376	1448	1448	1277
Pgk1	19586	19071	19461	20196	20639	20076	19469	19944
Pgls	2363	2290	2488	2354	2288	2131	2129	2140
Pglyrp1	4368	4260	4266	4150	4274	4351	4197	4158
Pglyrp2	38	37	22	33	40	37	32	23
Pgm1	4645	4452	4508	4357	4694	4421	4467	4484
Pgm2	7533	7875	7377	7409	7974	8129	8300	7613
Pgm2l1	284	332	339	281	400	339	389	286
Pgm3	1714	1832	1916	1723	2048	2142	2082	1688
Pgm5	901	1016	1088	1116	1008	963	1143	1034
Pgp	4887	4840	4783	4756	5313	5230	4880	5048
Pgpep1	3952	3986	4016	3977	4193	4307	4388	4147
Pgpep1l	3	2	7	0	5	9	8	0
Pgr	4	3	1	11	0	17	0	2

Transcriptome sequencing yielded total genetic results for the MOD and APS groups, with a total of 15,936 variables

Continued from above

Pgrmc1	10095	11002	9860	9143	10753	11316	11240	10241
Pgrmc2	5678	6857	6336	5430	6427	6801	6060	6009
Pgs1	2007	2007	1871	1991	2301	2069	2108	2156
Phactr1	30	44	30	57	57	39	50	22
Phactr2	676	688	732	725	824	845	769	717
Phactr3	0	5	7	10	0	1	4	0
Phactr4	2611	2744	2560	2481	2640	2550	2753	2556
Phax	371	479	453	517	530	471	480	480
Phb	5722	5663	5871	5717	5769	5448	4818	5423
Phb2	8240	8309	8540	8420	7890	8208	6742	7515
Phc1	192	254	139	149	164	162	141	167
Phc2	3160	3207	3011	3209	3037	2930	2747	2603
Phc3	1103	1183	1149	1233	1243	1236	1260	962
Pheta1	7310	7100	7315	6975	6895	7419	7300	6973
Pheta2	12	6	9	23	16	14	9	8
Phex	1	0	0	0	0	0	5	0
Phf1	1083	971	950	1082	885	911	794	938
Phf10	1106	1142	1118	1242	1131	1200	1197	1108
Phf11a	66	58	57	53	54	37	23	61
Phf11b	199	190	189	149	89	146	116	127
Phf11c	8	17	9	12	6	6	5	28
Phf11d	949	1173	1285	985	926	933	671	750
Phf12	1429	1150	1334	1273	1326	1291	1330	1160
Phf13	412	421	354	432	487	435	493	408
Phf14	866	778	777	765	779	791	828	795
Phf19	33	49	35	22	25	40	18	42
Phf2	1030	1113	1117	998	1061	1050	1070	924
Phf20	338	330	345	355	295	275	338	314
Phf20l1	981	1139	1022	915	1046	1216	1042	982
Phf21a	432	589	525	548	457	524	505	469
Phf21b	127	191	189	165	156	193	128	152
Phf23	1221	1268	1254	1420	1348	1364	1317	1418
Phf24	7	12	32	9	20	26	19	16
Phf3	1497	1454	1601	1480	1625	1640	1620	1464
Phf5a	1698	1921	1850	1825	1981	1877	1703	1819
Phf6	406	519	532	501	506	622	455	439
Phf7	160	160	145	114	180	154	157	118
Phf8	813	846	797	782	883	863	765	802
Phgdh	699	654	645	575	855	776	656	704
Phgr1	5271	5004	5471	5366	5077	5270	4984	5076
Phip	695	755	736	691	851	841	906	675
Phka1	167	155	163	123	157	135	113	106
Phka2	346	390	404	378	422	376	330	419
Phkb	1132	1030	1090	1190	1202	1199	1308	1301
Phkg1	6	2	4	0	1	1	2	0
Phkg2	704	627	619	610	611	533	625	589
Phlda1	715	678	698	652	682	605	580	654
Phlda2	50	61	70	40	64	55	44	38
Phlda3	261	249	295	250	311	332	291	325
Phldb1	982	1207	1136	1217	1138	981	906	1014
Phldb2	208	258	235	260	207	233	236	185
Phldb3	212	327	279	216	225	266	243	274
Phlpp1	695	882	700	761	706	818	710	662
Phlpp2	5119	5764	5025	4864	5394	5762	5671	4958
Phospho1	5320	4788	5042	4989	5752	5977	6777	6559
Phospho2	1178	1129	1027	1007	1403	1343	1298	1200
Phox2a	8	12	3	3	8	17	5	11
Phox2b	48	48	33	32	49	46	39	37

Transcriptome sequencing yielded total genetic results for the MOD and APS groups, with a total of 15,936 variables

Continued from above

Phpt1	399	439	475	442	431	358	372	380
Phrf1	2248	2342	2366	2369	2411	2313	2255	2243
Phtf1	302	323	238	307	277	335	317	226
Phtf2	96	81	120	96	110	59	80	87
Phyh	10380	10904	9619	9892	10200	10517	10605	9455
Phyhd1	56	34	44	77	75	53	68	56
Phyhip	10	0	1	0	6	0	3	2
Phyhipl	0	0	2	8	1	5	2	1
Phykpl	698	724	803	657	618	735	684	605
Pi15	1	14	4	8	4	9	17	3
Pi16	2	14	1	10	16	5	6	17
Pi4k2a	1783	2135	1820	1847	1907	1769	1935	1817
Pi4k2b	5819	6432	5814	5774	6200	6939	6553	6022
Pi4ka	2613	2933	2807	2736	2720	2568	2733	2512
Pi4kb	1382	1532	1459	1349	1492	1469	1390	1372
Pianp	11	32	41	26	24	18	31	32
Pias1	929	971	834	826	842	895	857	828
Pias2	724	791	754	664	685	790	619	743
Pias3	412	466	555	426	445	409	454	387
Pias4	1191	1116	1172	1156	1195	1152	1157	1127
Pibf1	135	149	156	178	155	132	148	150
Picalm	11782	13445	11519	11921	12622	13284	13473	12269
Pick1	1202	1417	1293	1225	1427	1432	1258	1290
Pid1	99	138	109	147	119	130	204	143
Pidd1	162	186	188	148	219	214	190	193
Piezo1	659	782	722	679	582	700	565	676
Piezo2	39	56	30	34	63	33	53	27
Pif1	170	173	206	190	218	251	170	227
Piga	884	936	961	988	1087	984	993	1002
Pigb	331	392	405	406	365	350	334	351
Pigbos1	281	313	306	313	321	283	349	298
Pigc	1071	1150	1028	1072	1169	966	1043	1164
Pigf	983	927	868	870	880	925	931	811
Pigg	779	991	825	757	1005	982	970	909
Pigh	382	481	458	433	536	580	432	469
Pigk	623	663	631	709	750	727	689	664
Pigl	622	609	587	599	662	602	548	597
Pigm	1113	939	1108	1007	1068	1069	1067	1054
Pign	1124	1261	979	1148	1142	1117	1117	1065
Pigo	1964	1907	1810	1859	1856	1825	1671	1676
Pigp	773	832	746	821	784	794	682	713
Pigq	3497	3511	3387	3523	3514	3416	3157	3275
Pigr	214118	229950	220818	218960	207791	223823	216956	196338
Pigs	2382	2624	2487	2260	2753	2523	2495	2251
Pigt	5300	5901	5253	4907	5289	5332	4850	5106
Pigu	1033	1091	1202	973	1163	1072	931	1048
Pigv	510	609	656	583	702	657	537	517
Pigw	179	194	198	189	194	198	214	197
Pigx	780	899	858	860	913	928	856	968
Pigyl	662	651	667	657	660	616	609	574
Pigz	71	95	90	93	83	135	116	136
Pih1d1	1055	1214	1146	1192	1146	1215	1049	1030
Pih1d2	18	18	42	13	15	21	32	42
Pik3ap1	438	490	446	415	366	385	352	338
Pik3c2a	4400	5074	4349	4482	4871	5219	5783	4778
Pik3c2b	2340	2310	2430	2514	2087	2137	2137	2090
Pik3c2g	26	27	25	20	13	38	28	26
Pik3c3	1976	1833	1903	2056	1383	1509	1704	1623

Transcriptome sequencing yielded total genetic results for the MOD and APS groups, with a total of 15,936 variables

Continued from above

Pik3cb	816	827	932	823	842	958	785	756
Pik3cd	406	367	324	389	342	364	283	342
Pik3cg	147	146	252	199	167	156	154	165
Pik3ip1	110	111	98	132	82	79	45	73
Pik3r1	2770	3143	3316	3155	3064	3423	3261	2830
Pik3r2	3611	3769	3539	3411	3400	3342	3102	3120
Pik3r3	370	420	438	468	546	550	476	472
Pik3r4	818	809	820	924	888	947	822	808
Pik3r5	97	103	93	111	139	99	122	94
Pik3r6	28	27	44	25	34	24	21	18
Pikfyve	664	954	773	892	829	993	898	836
Pilra	165	142	132	115	173	137	116	118
Pilrb1	97	74	72	63	95	105	52	48
Pilrb2	75	63	80	101	58	27	92	56
Pim1	4547	4518	3499	4057	3038	3127	3134	3008
Pim2	148	172	145	156	162	122	97	186
Pim3	2470	2644	3188	2787	2008	2125	2048	2329
Pimreg	19	14	13	10	5	7	14	8
Pin1	1057	948	966	938	975	933	838	831
Pin1rt1	0	0	0	1	0	0	0	0
Pin4	833	798	815	852	840	930	848	810
Pink1	5423	5887	5546	5569	5155	5403	5431	5089
Pinlyp	0	4	1	0	1	0	1	1
Pinx1	157	182	222	175	205	240	205	183
Pip4k2a	256	207	228	271	267	255	249	231
Pip4k2b	288	334	313	289	339	327	282	288
Pip4k2c	2909	2871	2886	2691	2827	2855	2834	2875
Pip4p1	1199	1221	1282	1152	1125	1135	1068	1027
Pip4p2	1016	935	877	817	1021	1024	937	794
Pip5k1a	1248	1305	1154	1085	1199	1263	1210	1209
Pip5k1b	3758	3965	4067	3872	3306	3663	3500	3499
Pip5k1c	1298	1159	1318	1225	1195	1260	1187	1032
Pip5kl1	4	0	0	0	1	0	0	8
Pipox	965	825	839	936	778	741	788	726
Pir	131	168	149	131	189	175	95	139
Pira1	6	6	6	6	4	2	1	1
Pira2	13	27	9	7	6	11	8	0
Pirb	222	244	157	254	140	149	174	164
Pirt	261	313	227	276	227	230	221	267
Pisd	3446	3814	3841	3803	3710	3799	3749	3584
Pithd1	733	906	768	746	914	926	839	816
Pitpna	19394	20096	18382	18241	19836	20425	19655	18659
Pitpnb	2429	2914	2631	2773	2652	2911	2597	2453
Pitpnc1	145	161	184	171	148	207	129	153
Pitpnm1	2375	2528	2609	2280	2313	2348	2235	2220
Pitpnm2	374	351	399	354	371	402	317	306
Pitpnm3	45	61	28	33	61	47	27	40
Pitrm1	1134	1308	1040	1073	1163	1143	993	1086
Pitx1	441	480	542	530	541	557	464	387
Pitx2	879	821	774	780	633	622	602	703
Piwil2	24	43	42	13	41	43	38	52
Piwil4	22	6	17	24	25	28	11	31
Pja1	4414	4816	4404	4210	4658	5023	4925	4580
Pja2	1838	1766	1663	1684	1717	1790	1740	1696
Pjvk	0	0	0	0	0	1	0	0
Pkd1	1065	1223	1287	1285	1175	1252	1209	1006
Pkd1l1	0	0	0	0	0	0	0	1
Pkd1l2	1	3	6	1	6	6	0	1

Transcriptome sequencing yielded total genetic results for the MOD and APS groups, with a total of 15,936 variables

Continued from above

Pkd1l3	98	131	116	113	74	130	81	77
Pkd2	368	379	448	430	413	476	361	344
Pkd2l1	4	0	2	0	1	1	2	0
Pkd2l2	64	63	77	98	55	93	55	28
Pkdcc	803	902	876	808	909	871	734	762
Pkdrej	0	2	9	2	4	6	12	7
Pkhd1	20	33	21	35	43	54	47	24
Pkhd1l1	12	6	6	11	4	19	9	6
Pkia	44	57	58	77	72	44	80	68
Pkib	24	29	15	36	26	40	25	16
Pkig	1914	1972	2014	2082	2110	1970	1970	1924
Pklr	20860	21750	19901	20264	21098	22381	23644	22314
Pkm	63086	64236	59744	59740	65012	65911	64958	66535
Pkmyt1	476	387	438	416	424	437	328	348
Pkn1	5939	6359	5962	5857	5859	5871	5852	5339
Pkn2	3323	3730	3359	3291	3587	3563	3737	3341
Pkn3	221	207	249	230	218	213	233	211
Pknox1	908	889	919	829	984	855	817	850
Pknox2	21	15	36	37	28	19	21	22
Pkp1	914	1012	991	931	906	872	684	828
Pkp2	2737	2701	2649	2818	2772	2855	2571	2436
Pkp3	5288	5367	5159	5092	4962	5292	4912	4978
Pkp4	3918	4097	3771	3507	3956	4197	3990	3898
Pla1a	68	52	51	43	58	29	15	26
Pla2g10	223	266	216	231	200	217	180	294
Pla2g12a	471	487	491	465	546	500	406	495
Pla2g12b	2677	2771	2645	2674	2623	2840	2637	2719
Pla2g15	172	184	190	237	191	149	202	162
Pla2g1b	120	160	93	21	548	447	394	407
Pla2g2c	0	1	1	0	1	4	6	1
Pla2g2d	328	232	226	216	229	296	176	271
Pla2g2e	4	9	5	4	4	3	4	6
Pla2g2f	238	268	329	297	257	265	313	365
Pla2g3	8	17	31	21	10	8	7	18
Pla2g4a	602	615	646	647	689	727	716	611
Pla2g4b	75	57	26	69	59	68	64	84
Pla2g4c	11471	3337	9100	18131	353	283	582	638
Pla2g4d	0	0	0	0	1	0	1	0
Pla2g4e	0	0	1	0	0	0	0	0
Pla2g4f	5	0	2	1	0	1	1	1
Pla2g5	1294	1377	1288	1026	1753	1575	1474	1499
Pla2g6	1197	1232	1229	1152	1437	1413	1279	1332
Pla2g7	1002	844	841	913	810	953	873	868
Pla2r1	85	110	96	123	80	87	62	94
Plaa	1538	1764	1550	1377	1957	1819	1615	1528
Plaat1	6	5	1	6	2	6	3	0
Plaat3	3775	3753	3470	3639	2818	3001	2716	2685
Plaat5	0	0	0	0	4	2	0	0
Plac8	85514	92655	90317	85047	91262	95564	89568	84239
Plac8l1	0	0	0	0	0	0	4	0
Plac9a	92	111	116	138	160	241	153	152
Plag1	91	100	84	103	94	98	93	84
Plagl1	18	20	45	21	18	46	44	21
Plagl2	2252	2779	2703	2375	2848	2704	2510	2270
Plat	271	238	194	262	264	214	148	184
Plau	353	302	310	325	332	391	266	335
Plaur	162	170	201	148	198	182	125	166
Plb1	22	5	16	8	20	36	18	19

Transcriptome sequencing yielded total genetic results for the MOD and APS groups, with a total of 15,936 variables

Continued from above

Plbd1	1425	1445	1423	1405	1391	1375	1280	1119
Plbd2	935	938	1063	915	1029	974	878	1006
Plcb1	64	54	71	81	76	73	72	86
Plcb2	111	118	84	112	96	111	79	79
Plcb3	27910	29691	28834	27792	27878	28092	27315	26621
Plcb4	142	155	157	112	133	146	159	110
Plcd1	722	622	726	879	644	721	638	646
Plcd3	54	51	71	47	53	38	42	45
Plcd4	0	1	2	0	0	0	5	1
Plce1	960	1033	927	981	930	998	992	959
Plcg2	1792	1991	1754	1842	1759	1802	1758	1881
Plch1	675	795	800	790	904	861	745	793
Plch2	550	605	662	671	513	565	492	528
Plcl1	22	26	43	39	16	44	23	17
Plcl2	1489	1659	1592	1562	1504	1718	1515	1449
Plcxd1	29	22	25	32	18	20	25	15
Plcxd2	635	748	717	558	970	856	754	739
Plcxd3	3	5	7	1	3	16	0	8
Pld1	6893	7397	6869	6793	7186	7419	7206	6911
Pld2	631	688	789	726	716	810	638	600
Pld3	808	800	836	784	887	770	737	749
Pld4	476	320	351	389	261	256	294	243
Pld5	7	10	8	8	10	4	0	2
Pld6	6	3	3	12	19	18	6	8
Plec	16049	17564	16081	15689	17279	18247	18734	16802
Plek	221	257	208	268	177	143	214	194
Plek2	816	807	778	698	937	868	896	832
Plekha1	1634	1642	1453	1480	1752	2027	2002	1906
Plekha2	3061	3670	3299	3178	3479	3633	3185	3036
Plekha3	1250	1186	1391	1266	1187	1321	1072	1038
Plekha4	47	71	62	92	57	34	34	38
Plekha5	1537	1788	1693	1480	1508	1613	1434	1481
Plekha6	4842	5292	4971	4705	5641	5329	5069	4568
Plekha7	3628	3917	3981	3719	3599	3932	3703	3353
Plekha8	1709	1913	1854	1738	2109	2338	2069	2150
Plekhb1	41	108	84	68	59	69	59	54
Plekhb2	17107	17571	15914	16286	16742	17444	17492	16773
Plekhd1	11	9	3	2	7	12	4	10
Plekhf1	689	633	750	754	676	728	748	685
Plekhf2	1894	2050	1986	1953	2004	2133	2162	2020
Plekhg1	489	462	490	581	349	361	393	309
Plekhg2	946	879	782	948	840	903	1031	939
Plekhg3	865	796	850	929	958	795	781	762
Plekhg4	12	5	6	3	10	6	14	9
Plekhg5	166	162	227	200	175	143	192	174
Plekhg6	4063	4481	4053	3710	3857	4173	4248	3905
Plekhh1	3058	3289	3248	3209	3508	3458	3607	3441
Plekhh2	175	193	215	197	123	152	140	132
Plekhh3	1006	1039	1043	1048	1074	1084	1255	1197
Plekhj1	917	938	973	947	1008	970	887	861
Plekhm1	2386	2404	1997	2129	2029	2041	2014	2015
Plekhm2	432	435	441	481	405	434	357	467
Plekhm3	353	485	421	321	432	456	528	469
Plekhn1	609	651	693	656	460	446	492	515
Plekho1	303	274	306	285	300	313	245	273
Plekho2	574	581	510	420	570	610	556	453
Plekhs1	29	44	25	13	51	31	37	24
Plet1	407	385	334	327	455	479	546	669

Transcriptome sequencing yielded total genetic results for the MOD and APS groups, with a total of 15,936 variables

Continued from above

Plg	19	17	16	14	27	19	22	23
Plgrkt	2404	2493	2420	2312	2373	2630	2320	2153
Plin1	13	3	17	14	9	53	43	6
Plin2	3075	3329	3080	2926	3889	3775	3390	3264
Plin3	17285	19067	15760	14665	18487	19536	19371	17556
Plin4	66	86	74	80	59	66	111	47
Plin5	7	8	0	3	7	20	5	5
Plk1	1285	1381	1393	1313	1497	1377	1195	1383
Plk2	194	226	232	234	210	218	195	192
Plk3	757	764	882	896	708	861	897	948
Plk4	394	425	360	360	445	384	347	413
Plk5	7	21	7	8	6	24	25	10
Pllp	641	736	801	715	839	696	749	784
Pln	63	67	83	70	68	59	86	82
Plod1	421	471	516	424	466	516	528	413
Plod2	96	69	89	101	114	130	79	85
Plod3	1048	1175	1286	1111	1136	1268	1004	1142
Plp1	21	34	22	26	8	36	41	37
Plp2	1979	2116	2016	1894	2263	1974	1695	2029
Plpbp	1986	2269	2235	2121	2219	2213	2083	2114
Plpp1	3643	4370	3711	3398	3757	3950	4177	3706
Plpp2	9832	9977	9913	9858	9590	9809	9406	9693
Plpp3	1892	2097	1958	2091	1874	1910	1826	1923
Plpp4	0	0	0	0	1	0	0	0
Plpp5	1257	1370	1281	1330	1385	1463	1234	1297
Plpp6	2427	2702	2594	2578	2322	2280	2456	2341
Plpp7	16	24	13	18	26	26	23	51
Plppr1	0	0	0	0	1	0	1	0
Plppr2	29	50	53	52	49	34	66	42
Plppr3	112	100	169	93	88	132	59	78
Plppr4	1	1	1	2	15	1	13	1
Plppr5	6	30	11	15	3	17	14	21
Plrg1	1232	1244	1281	1144	1329	1403	1151	1181
Pls1	38032	42634	36302	35573	39322	43807	47807	42174
Pls3	441	386	359	441	413	401	414	409
Plscr1	2489	2771	2306	2284	2872	2602	2727	2908
Plscr2	53	54	59	50	64	72	25	33
Plscr3	482	454	464	464	439	419	346	452
Plscr4	56	81	77	59	116	125	100	76
Pltp	413	399	398	366	337	292	299	306
Plvap	2864	2733	2817	3033	2386	2322	2184	2232
Plxdc1	17	19	22	23	20	23	5	3
Plxdc2	86	67	90	121	117	103	97	87
Plxna1	1715	1822	1709	1540	1610	1709	1489	1466
Plxna2	1257	1541	1284	1042	1381	1305	1309	1159
Plxna3	42	42	25	40	61	27	33	25
Plxna4	57	87	73	85	64	73	51	69
Plxnb1	86	117	151	108	106	81	132	126
Plxnb2	18625	19353	17511	17646	18069	18206	18064	17597
Plxnb3	12	9	3	13	21	3	13	7
Plxnc1	32	45	66	44	40	46	34	27
Plxnd1	552	595	496	539	478	462	514	452
Pm20d1	913	897	856	906	842	990	911	890
Pm20d2	160	176	140	157	181	144	200	126
Pmaip1	1766	2184	2127	1774	1905	1934	2191	2070
Pmch	8	0	0	2	0	0	4	1
Pmel	56	57	68	57	84	99	83	71
Pmepa1	1235	1191	1396	1363	1347	1234	1196	1205

Transcriptome sequencing yielded total genetic results for the MOD and APS groups, with a total of 15,936 variables

Continued from above

Pmf1	876	1022	881	831	823	784	745	800
Pmfbp1	0	0	0	0	0	0	0	2
Pml	2977	3538	3281	2689	3211	3065	2982	3019
Pmm1	1324	782	1036	1669	764	739	647	708
Pmm2	6723	7684	7194	7006	7279	7243	7119	7222
Pmp22	669	751	903	828	978	1053	1400	962
Pmpca	3289	3293	3239	3191	3074	3196	2893	3088
Pmpcb	3025	3196	3210	3206	3088	2988	2785	2809
Pms1	123	101	97	118	150	114	124	93
Pms2	270	280	288	242	294	306	274	226
Pmvk	954	910	990	1108	999	900	872	957
Pnck	34	39	33	27	44	28	52	34
Pnisr	1029	1108	1058	1127	1172	1420	1204	946
Pnkd	1284	1212	1308	1262	1131	1138	1016	989
Pnkp	1021	1227	1093	971	1099	1053	1086	990
Pnldc1	11	7	8	9	6	9	10	11
Pnlip	4847	6488	2918	1171	19072	17560	11337	15777
Pnliprp1	1797	2347	1171	535	9323	8799	6972	8068
Pnliprp2	1241	1084	748	906	2117	1911	1643	2046
Pnma1	31	7	22	11	13	18	6	2
Pnma2	6	2	23	20	13	8	3	10
Pnma3	1	0	0	1	0	0	0	4
Pnma5	0	0	0	1	4	0	1	0
Pnmal1	24	26	23	16	39	32	12	24
Pnmal2	43	48	41	22	34	48	33	38
Pnn	1709	1929	1840	1846	2182	2384	1909	1848
Pno1	1290	1314	1166	1141	1385	1280	1223	1314
Pnp	12943	13915	13126	12792	11759	12130	11825	10858
Pnp2	7598	8086	7037	7318	6104	6834	6692	6273
Pnpla1	19	30	29	10	30	39	36	19
Pnpla2	6148	6393	6231	6131	5702	5736	6049	5833
Pnpla3	2	4	5	1	12	10	18	2
Pnpla6	2408	2661	2542	2293	2379	2712	2389	2301
Pnpla7	450	471	350	393	456	353	402	361
Pnpla8	1312	1510	1482	1377	1630	1717	1645	1546
Pnpo	2883	3173	3015	2896	3247	3617	3033	2934
Pnpt1	1620	1955	1828	1692	2047	1996	1785	1652
Pnrc1	4409	4539	3992	3989	4686	4625	4771	4470
Pnrc2	3332	3587	3737	3545	3854	4008	3635	3469
Poc1a	393	353	417	466	412	369	306	407
Poc1b	822	902	904	881	803	806	755	867
Poc5	442	405	422	413	368	406	343	397
Podn	682	728	722	736	746	906	940	832
Podnl1	53	47	38	45	63	39	30	40
Podxl	624	698	659	694	576	673	566	644
Podxl2	32	19	43	23	34	60	28	14
Pof1b	2225	2426	2430	2520	2576	2859	2949	2611
Pofut1	1097	1408	1287	1360	1502	1306	1195	1177
Pofut2	1048	1173	1036	1059	1212	1096	1178	1038
Pogk	283	339	347	414	270	301	293	283
Poglut1	347	322	372	402	352	402	310	375
Poglut2	287	247	285	278	253	298	240	327
Poglut3	320	424	377	388	505	465	429	461
Pogz	522	600	571	617	568	597	537	591
Pola1	461	498	491	495	505	519	547	308
Pola2	794	895	697	893	739	730	746	617
Polb	458	472	473	450	626	562	489	408
Pold1	823	964	898	789	983	927	843	737

Transcriptome sequencing yielded total genetic results for the MOD and APS groups, with a total of 15,936 variables

Continued from above

Pold2	1366	1503	1446	1375	1406	1529	1340	1240
Pold3	1065	1193	1154	1010	1131	1122	1014	979
Pold4	626	742	577	665	604	727	740	687
Poldip2	1753	1648	1706	1617	1578	1639	1593	1590
Poldip3	3614	3617	3358	3398	3564	3826	3613	3483
Pole	386	494	451	431	390	375	421	331
Pole2	136	147	189	153	172	183	134	159
Pole3	653	766	745	647	738	849	764	659
Pole4	641	655	685	621	702	685	684	597
Polg	1794	2119	1992	1927	2037	1903	2043	1898
Polg2	350	495	474	397	366	449	388	366
Polh	217	271	204	221	271	217	251	228
Poli	73	86	99	110	136	132	83	78
Polk	153	147	173	205	217	222	167	155
Poll	277	315	364	289	304	352	238	286
Polm	675	642	677	627	631	650	502	597
Poln	0	3	0	4	0	3	10	1
Polq	166	130	181	148	176	162	161	167
Polr1a	694	889	754	808	893	894	776	838
Polr1b	605	654	538	572	640	538	550	540
Polr1c	691	820	835	745	888	747	807	780
Polr1d	2279	2823	2695	2273	2523	2877	2485	2586
Polr1e	221	247	209	206	265	255	180	230
Polr2a	5108	5446	5355	4670	5278	5215	5287	4701
Polr2b	1971	2039	1895	1798	2184	1990	2008	1693
Polr2c	1060	1184	1018	1203	1172	1192	1039	1014
Polr2d	473	429	473	445	428	443	430	366
Polr2e	1436	1535	1417	1658	1442	1512	1419	1439
Polr2f	1015	1082	945	1058	1020	1072	828	961
Polr2g	629	705	710	609	743	691	637	596
Polr2h	580	629	555	523	692	528	607	600
Polr2i	317	393	383	377	428	378	314	320
Polr2j	793	864	910	865	843	956	890	811
Polr2k	137	134	136	128	122	161	87	153
Polr2l	532	606	610	529	616	670	503	506
Polr2m	3503	3761	3688	3525	3360	3673	3448	3173
Polr3a	591	503	560	510	543	644	558	525
Polr3b	616	646	684	592	630	658	657	598
Polr3c	2697	2832	2675	2642	2548	2636	2547	2330
Polr3d	415	426	322	353	437	472	375	406
Polr3e	856	900	895	870	950	996	852	880
Polr3f	617	691	727	685	595	580	603	643
Polr3g	128	166	138	123	153	212	139	168
Polr3gl	366	356	354	413	405	342	368	304
Polr3h	274	269	285	193	215	217	177	238
Polr3k	851	896	832	911	992	1058	984	968
Polrmt	1217	1184	1236	1090	1206	1252	1031	1172
Pom121	1972	1904	1810	1849	2017	1904	1718	1677
Pom121l2	0	1	4	0	0	0	0	0
Pomc	1	11	7	12	6	2	2	4
Pomgnt1	723	866	713	730	1014	867	800	748
Pomgnt2	160	163	114	103	187	131	142	146
Pomk	228	229	186	167	188	214	149	187
Pomp	4767	4814	4660	4702	4636	4848	4642	4487
Pomt1	568	736	637	562	607	593	583	571
Pomt2	414	615	557	522	530	612	517	576
Pon1	5	0	2	2	2	1	3	1
Pon2	3638	4208	4005	3977	4119	4226	4241	3939

Transcriptome sequencing yielded total genetic results for the MOD and APS groups, with a total of 15,936 variables

Continued from above

Pon3	2796	2766	2579	2593	2779	2800	2824	2533
Pop1	159	241	176	179	245	210	180	169
Pop4	454	461	471	477	548	510	419	532
Pop5	464	500	590	491	542	506	469	523
Pop7	404	378	444	440	441	449	369	313
Popdc2	102	106	121	107	117	114	78	76
Popdc3	0	1	0	6	0	1	1	0
Por	8474	9253	8429	7608	10412	10380	10142	10220
Porcn	50	68	45	74	61	52	49	59
Postn	265	316	335	311	352	314	319	250
Pot1a	320	264	292	246	305	279	281	249
Pot1b	140	211	173	149	155	145	174	161
Potegl	0	0	0	1	1	1	0	0
Pou2af1	469	458	389	438	427	416	397	393
Pou2f1	852	1016	952	795	931	946	809	793
Pou2f2	48	51	57	41	31	34	27	43
Pou2f3	25	11	21	20	17	28	25	13
Pou3f1	2	0	0	0	11	5	0	9
Pou3f2	0	0	1	0	0	0	0	0
Pou3f4	0	0	0	0	0	0	1	0
Pou4f1	8	4	13	21	2	5	3	6
Pou5f1	0	0	0	1	0	0	0	0
Pou5f2	2	0	0	0	0	0	0	0
Pou6f1	69	112	107	106	77	66	76	93
Pp2d1	0	0	0	0	0	0	0	6
Ppa1	18142	20569	18746	17029	21019	21760	20527	19237
Ppa2	1772	1783	1663	1742	1858	1685	1585	1422
Ppan	551	685	642	535	650	723	490	573
Ppara	1405	1776	1842	1551	1948	1992	1685	1710
Ppard	7999	8165	7299	7599	8600	8606	8617	8027
Pparg	559	716	571	530	717	711	579	574
Ppargc1a	1866	1829	1735	2029	1796	1652	1753	1572
Ppargc1b	2565	2557	2828	3053	2232	2048	2152	2232
Ppat	392	416	354	346	501	398	365	399
Ppbp	2	1	0	0	2	0	5	1
Ppcdc	302	336	305	310	408	319	342	310
Ppcs	888	874	873	885	1107	1066	940	1001
Ppdpf	1469	1411	1558	1355	1483	1349	1203	1426
Ppef1	108	39	53	98	83	65	99	100
Ppef2	54	37	53	64	46	18	57	41
Ppfia1	1913	2248	2043	1893	2071	2051	1797	1839
Ppfia2	4	3	4	2	3	6	2	4
Ppfia3	693	699	791	872	787	842	864	784
Ppfia4	226	174	152	169	176	156	189	171
Ppfibp1	831	885	930	954	822	754	837	799
Ppfibp2	906	1067	977	928	1068	1026	1022	1051
Pphln1	709	859	739	763	823	854	806	768
Ppia	21614	22950	22958	22982	25386	24012	21414	23341
Ppib	8359	8980	9068	8640	9764	9242	8726	8998
Ppic	131	126	204	129	163	221	181	112
Ppid	784	802	833	729	1070	997	924	820
Ppie	586	556	597	553	619	672	542	557
Ppif	2324	2330	2388	2310	2393	1999	1971	2101
Ppig	1486	1635	1477	1568	1809	1858	1775	1506
Ppih	622	691	523	535	622	570	573	633
Ppil1	878	1004	1004	849	976	993	884	970
Ppil2	2469	2593	2228	2463	2550	2651	2655	2510
Ppil3	302	290	305	286	307	285	287	321

Transcriptome sequencing yielded total genetic results for the MOD and APS groups, with a total of 15,936 variables

Continued from above

Ppil4	604	672	565	621	638	750	596	625
Ppil6	14	24	20	39	36	31	43	37
Ppip5k1	3497	3639	3574	3708	3407	3473	3308	3072
Ppip5k2	4132	4615	4324	4587	4416	4831	5166	4533
Ppl	352	345	352	379	527	461	478	424
Ppm1a	5777	6558	5998	5971	5933	6328	6007	5902
Ppm1b	4591	4896	4657	4672	4919	4864	4818	4696
Ppm1d	337	232	231	205	250	280	238	199
Ppm1e	37	44	69	28	33	31	41	50
Ppm1f	277	304	334	318	349	298	311	330
Ppm1g	2967	2964	2858	2752	2917	2802	2661	2499
Ppm1h	3755	4207	3388	3510	3594	3747	3842	3411
Ppm1j	1252	1200	1259	1187	1106	1253	1188	1254
Ppm1k	481	691	591	536	519	538	508	438
Ppm1l	261	317	355	283	337	360	392	399
Ppm1m	143	129	130	135	135	84	136	118
Ppm1n	32	31	44	33	60	59	48	54
Ppme1	1815	1721	1891	2064	2117	2134	1800	1967
Ppox	540	591	589	548	628	636	572	639
Ppp1ca	7569	7976	7963	7626	7841	7791	7386	7441
Ppp1cb	8139	8829	8090	7990	9157	9163	9057	8572
Ppp1cc	6060	6134	5982	5906	5994	6343	6064	5789
Ppp1ccb	391	432	360	398	384	336	388	395
Ppp1r10	1668	1747	1668	1787	1829	1834	1772	1830
Ppp1r11	1197	1267	1112	1119	1222	1235	1104	1351
Ppp1r12a	3133	3474	3196	3175	3437	3483	3581	3194
Ppp1r12b	1135	1150	1237	1258	1082	1102	1014	997
Ppp1r12c	1582	1644	1507	1646	1717	1664	1672	1686
Ppp1r13b	2260	2359	2449	2495	2454	2527	2420	2366
Ppp1r13l	137	149	173	149	189	148	155	229
Ppp1r14a	161	104	133	143	122	147	152	147
Ppp1r14b	2289	2200	2126	2074	2297	1971	1964	2101
Ppp1r14c	18	17	17	21	18	20	9	8
Ppp1r14d	5326	5765	5280	5210	5552	6018	5348	5176
Ppp1r15a	336	427	386	372	396	399	329	414
Ppp1r15b	11561	12200	11559	11121	12142	12725	12443	11932
Ppp1r16a	3834	3811	3711	3685	3757	3638	3808	3631
Ppp1r16b	914	854	929	1212	732	794	779	670
Ppp1r17	0	0	0	0	0	4	0	0
Ppp1r18	540	572	524	521	493	504	483	460
Ppp1r1a	24	13	18	15	7	29	10	12
Ppp1r1b	19869	17728	18961	20141	16835	17343	16259	16895
Ppp1r1c	0	1	0	0	0	0	1	0
Ppp1r2	2945	3161	3241	3077	2963	3144	2824	2924
Ppp1r21	864	854	937	818	910	930	937	859
Ppp1r26	71	63	17	50	58	56	51	47
Ppp1r27	1	0	2	1	0	0	0	4
Ppp1r32	2	1	3	3	1	5	1	7
Ppp1r35	588	537	555	643	549	558	523	535
Ppp1r36	0	0	0	1	1	0	0	0
Ppp1r37	3684	3438	3659	3368	3150	3416	3189	3388
Ppp1r3b	100	116	102	125	182	126	131	143
Ppp1r3c	58	43	32	38	59	46	52	49
Ppp1r3d	7	3	15	25	15	15	14	5
Ppp1r3e	169	174	183	192	225	192	139	161
Ppp1r3f	48	25	36	28	50	43	41	37
Ppp1r3g	2	4	4	9	2	4	2	0
Ppp1r42	0	0	0	0	4	0	1	0

Transcriptome sequencing yielded total genetic results for the MOD and APS groups, with a total of 15,936 variables

Continued from above

Ppp1r7	1179	1290	1332	1254	1222	1289	1231	1116
Ppp1r8	1015	868	1045	1033	925	974	953	841
Ppp1r9a	222	279	298	292	284	257	200	140
Ppp1r9b	2820	2686	2799	2905	2899	2796	2771	2834
Ppp2ca	9432	9972	9423	9528	10263	10464	9899	9917
Ppp2cb	3077	2972	3099	3132	3020	2803	2816	2734
Ppp2r1a	6017	6124	6147	5863	5485	5426	5179	5474
Ppp2r1b	759	761	731	674	765	747	668	757
Ppp2r2a	1734	1886	1833	1926	1839	1918	1756	1789
Ppp2r2b	10	24	10	15	21	10	20	23
Ppp2r2c	31	16	33	31	14	13	43	14
Ppp2r2d	1278	1408	1314	1252	1187	1299	1275	1172
Ppp2r3a	823	829	851	963	822	841	859	757
Ppp2r3c	533	562	540	542	686	755	592	596
Ppp2r3d	1103	961	1078	970	882	888	960	996
Ppp2r5a	3732	3765	3797	3923	4258	4192	4097	3840
Ppp2r5b	1580	1616	1619	1682	1687	1583	1550	1497
Ppp2r5c	6366	6643	6144	6249	7081	7169	7164	6810
Ppp2r5d	3589	3924	3766	3776	3780	3708	3829	3875
Ppp2r5e	1544	1499	1558	1596	1631	1699	1593	1554
Ppp3ca	1578	1662	1541	1582	1425	1366	1460	1353
Ppp3cb	2036	2175	2170	2259	2214	2177	2036	1899
Ppp3cc	131	112	127	124	77	115	126	87
Ppp3r1	3540	3793	3408	3483	3772	3861	3810	3566
Ppp4c	3563	3569	3411	3306	3749	3572	3180	3400
Ppp4r1	4506	5145	4308	4033	4723	4678	4881	4351
Ppp4r2	1634	1683	1540	1531	1918	1885	1718	1697
Ppp4r3a	2501	2797	2662	2822	3017	2996	2852	2530
Ppp4r3b	2600	2774	2689	2622	3332	3430	3286	2733
Ppp4r4	3	4	3	2	1	1	8	8
Ppp5c	3093	3350	3488	3515	3561	3368	3232	2982
Ppp6c	2335	2407	2237	2214	2481	2447	2390	2383
Ppp6r1	8027	7757	7641	7563	7760	7673	7469	7448
Ppp6r2	975	1192	1054	962	951	1092	1004	960
Ppp6r3	6989	7179	6746	7161	6535	6783	7160	6403
Pprc1	786	939	839	859	1015	995	828	890
Ppt1	5051	5398	4616	4847	4734	4836	4939	4943
Ppt2	617	687	711	677	650	655	508	598
Pptc7	3857	3832	3660	3750	3616	3825	3893	3462
Ppwd1	245	232	270	270	366	298	288	299
Ppy	1	0	0	0	1	10	1	2
Pqbp1	626	700	640	699	659	709	553	667
Pqlc3	695	676	654	724	707	682	565	674
Pradc1	259	251	292	260	307	244	221	198
Praf2	725	744	749	769	698	713	678	789
Prag1	1327	1371	1447	1251	1484	1340	1375	1168
Pram1	29	26	18	29	39	24	28	28
Pramel12	521	571	549	497	487	549	534	504
Pramel13	0	0	0	0	0	1	0	0
Pramel29	3	9	5	3	5	9	3	14
Pramel34	37	75	16	32	54	45	65	29
Prap1	58042	58633	56644	58322	54003	58491	59198	55167
Prc1	1201	1275	1191	1148	1292	1246	1266	1101
Prcc	1519	1613	1691	1650	1704	1636	1543	1621
Prcd	7	8	3	8	7	10	3	10
Prcp	441	544	505	544	570	538	475	509
Prdm1	854	882	672	713	612	760	721	663
Prdm10	513	578	570	596	696	598	665	565

Transcriptome sequencing yielded total genetic results for the MOD and APS groups, with a total of 15,936 variables

Continued from above

Prdm11	48	38	55	36	56	46	63	69
Prdm12	0	0	0	0	1	0	0	0
Prdm14	0	1	1	0	0	0	0	1
Prdm15	332	375	339	365	474	411	355	332
Prdm16	1671	1789	1793	1848	1871	1876	1635	1480
Prdm2	1910	2047	2065	1789	2047	2194	1968	1862
Prdm4	1237	1243	1295	1204	1305	1378	1292	1219
Prdm5	11	10	10	10	15	11	5	21
Prdm6	1	1	3	0	3	4	0	0
Prdm8	0	0	0	1	0	3	0	1
Prdm9	130	190	159	180	127	151	148	143
Prdx1	43261	39988	42967	47988	40049	40675	38377	37035
Prdx2	7882	7450	7512	7849	7173	7027	7105	6897
Prdx3	3834	3979	4020	4199	4215	4318	3801	4024
Prdx4	1234	1264	1244	1357	1357	1509	1309	1287
Prdx5	14580	14798	14054	13334	13057	13894	13642	13451
Prdx6	13988	15646	15019	13680	17006	16629	15180	16173
Preb	4324	4473	4305	4267	4737	4822	4389	4469
Prelid1	18527	18959	19082	17845	18421	18476	17525	17775
Prelid2	352	399	395	431	441	364	339	350
Prelid3a	1	1	1	5	0	1	0	2
Prelid3b	4938	5037	4633	4944	4803	4968	4831	4513
Prelp	506	621	670	555	645	599	493	539
Prep	2096	2241	2195	2126	2241	2099	2094	2043
Prepl	1391	1381	1330	1315	1515	1403	1415	1321
Prex1	763	667	731	817	657	725	638	598
Prex2	182	167	228	225	151	170	141	126
Prf1	12	19	17	27	17	15	12	11
Prg2	59	129	106	100	85	68	87	94
Prg4	0	14	3	1	8	1	6	11
Prickle1	55	49	73	45	37	51	62	42
Prickle2	117	153	145	147	97	114	91	102
Prickle3	209	209	155	159	212	173	180	197
Prickle4	21	10	4	7	26	7	9	12
Prim1	412	488	460	450	434	436	346	353
Prim2	297	251	275	258	303	242	212	230
Prima1	3	13	19	15	16	3	5	5
Primpol	550	617	638	540	602	644	665	577
Prkaa1	1529	1577	1588	1684	1674	1751	1695	1414
Prkaa2	1647	1639	1697	2047	1480	1702	1596	1601
Prkab1	1639	1672	1497	1557	1605	1523	1533	1538
Prkab2	123	200	189	165	112	164	170	117
Prkaca	2793	2965	2844	2783	2929	2920	2892	2761
Prkacb	483	563	575	585	595	538	434	498
Prkag1	2464	2631	2344	2552	2325	2356	2346	2354
Prkag2	1027	1166	1149	1055	1269	1307	1299	1205
Prkag3	1	0	2	2	0	3	2	1
Prkar1a	5646	6083	5931	5890	5748	5844	5837	5710
Prkar1b	103	120	119	116	115	96	122	107
Prkar2a	9418	9858	9682	9268	9762	10081	9494	9168
Prkar2b	92	85	130	121	113	108	99	79
Prkca	5028	5695	5600	5370	5412	5554	5464	5078
Prkcb	102	162	138	152	107	174	155	79
Prkcd	8913	10052	8340	8436	8564	8839	9380	8533
Prkce	887	1057	1063	948	875	1040	1051	875
Prkcg	16	20	7	7	36	8	18	14
Prkch	141	128	153	169	139	137	120	133
Prkci	2370	2686	2455	2279	2542	2664	2351	2383

Transcriptome sequencing yielded total genetic results for the MOD and APS groups, with a total of 15,936 variables

Continued from above

Prkcq	26	62	54	22	31	53	46	45
Prkcsh	5941	6340	5617	5958	6049	6371	5843	5678
Prkcz	2832	2786	2834	2821	2773	2644	2921	2976
Prkd1	35	51	38	74	33	60	41	36
Prkd2	779	856	783	749	828	833	725	819
Prkd3	335	354	325	309	373	408	258	248
Prkdc	358	483	476	436	534	501	464	423
Prkg1	57	36	63	72	53	82	65	75
Prkg2	4159	4338	3993	4228	3717	4241	4688	3988
Prkn	13	2	9	3	15	5	11	3
Prkra	1511	1535	1467	1492	1583	1531	1541	1466
Prkrip1	398	388	395	396	402	434	367	395
Prkx	749	839	687	747	837	863	783	693
Prl5a1	0	0	0	0	0	0	1	0
Prl6a1	1	1	1	1	9	1	1	1
Prlh	1	1	0	0	0	0	4	0
Prlhr	0	0	1	0	0	0	0	0
Prlr	3909	4356	4086	4174	4175	4641	5002	4098
Prm1	0	0	0	4	0	1	0	0
Prmt1	1603	1704	1634	1543	1754	1708	1539	1615
Prmt2	291	266	299	238	351	320	338	296
Prmt3	424	545	435	393	516	518	439	364
Prmt5	925	1037	997	1001	1233	1081	926	1031
Prmt6	276	307	401	322	335	271	322	270
Prmt7	720	818	691	584	727	694	662	625
Prmt8	1	1	1	1	0	2	1	2
Prmt9	313	390	369	416	382	396	345	374
Prnp	553	573	616	590	513	584	541	514
Prob1	8	17	11	25	10	12	31	17
Proc	66	54	39	67	38	59	57	40
Proca1	3	6	1	5	4	3	4	5
Procr	128	128	132	140	152	107	109	95
Prodh	2270	2073	2184	2307	1842	1876	2010	2101
Prodh2	6	27	7	25	18	7	7	23
Prok1	0	1	0	0	0	0	0	1
Prok2	0	1	1	0	0	0	0	0
Prokr1	2	9	12	10	5	1	5	2
Prom1	3606	4082	4180	3627	4436	4538	4237	4092
Prom2	0	1	0	0	5	10	7	17
Prorp	564	585	548	492	570	572	496	511
Prorsd1	318	377	268	270	366	310	352	334
Pros1	1168	1213	1176	1194	1143	1088	1008	975
Proser1	673	639	757	736	718	662	656	621
Proser2	227	205	227	218	246	265	220	197
Proser3	67	62	60	72	49	91	58	70
Prox1	59	38	41	51	50	39	38	38
Prox2	16	40	11	18	14	20	12	34
Proz	1041	1274	1163	1245	1121	1151	1287	1114
Prpf18	1146	1342	1143	1232	1239	1366	1221	1040
Prpf19	2971	3262	3003	2981	3374	3132	2789	2857
Prpf3	689	765	724	761	825	855	781	776
Prpf31	1004	1012	1024	944	944	946	844	858
Prpf38a	870	1068	1006	917	1089	1061	957	939
Prpf38b	2157	2436	2463	2555	3000	2940	2736	2480
Prpf39	534	537	632	496	560	661	527	569
Prpf4	718	704	589	546	718	674	693	697
Prpf40a	3677	4078	3769	3772	4167	4397	4224	3830
Prpf40b	99	118	115	123	117	138	121	70

Transcriptome sequencing yielded total genetic results for the MOD and APS groups, with a total of 15,936 variables

Continued from above

Prpf4b	2532	2807	2745	2585	3119	3160	3110	2736
Prpf6	1848	2004	1916	1904	2031	1866	1900	1957
Prpf8	7217	8343	7797	7373	7949	8052	7557	7087
Prph	116	118	134	135	98	162	133	128
Prps1	564	617	553	634	617	590	599	557
Prps1l3	685	793	843	749	973	812	779	793
Prps2	1813	1426	1066	1382	1266	1273	1338	1657
Prpsap1	10956	11534	11513	10314	11049	11017	10180	10546
Prpsap2	529	598	493	466	590	609	587	506
Prr11	237	316	308	253	281	319	240	265
Prr12	486	511	479	460	411	511	466	411
Prr13	17773	18618	16311	16238	16056	17213	17708	17637
Prr14	2119	2042	2208	2150	2376	2133	2194	2125
Prr14l	1084	1240	1153	1143	1277	1300	1240	1221
Prr15	2310	2621	2393	2313	2836	2866	3094	3012
Prr15l	6069	6083	6184	6081	6155	6614	6227	5768
Prr16	31	19	16	20	20	19	27	27
Prr18	1	4	2	3	9	10	3	6
Prr19	1	0	0	0	0	1	0	6
Prr22	4	9	34	8	9	8	14	9
Prr29	13	1	4	0	1	6	2	0
Prr3	202	205	251	180	195	178	229	221
Prr36	2	3	3	7	5	1	2	0
Prr5	1421	1433	1481	1320	1364	1356	1364	1346
Prr5l	121	124	138	116	97	88	72	94
Prr7	20	39	26	12	25	28	24	22
Prr9	0	2	1	0	0	0	1	0
Prrc1	3281	3282	3235	3014	3670	3637	3425	3401
Prrc2a	8344	8493	8614	8057	8329	8204	7803	7534
Prrc2b	2111	2314	2167	2215	2150	2024	1914	1792
Prrc2c	4151	5002	4612	4410	4358	4637	4477	3837
Prrg1	16	38	28	39	36	22	37	9
Prrg2	2095	2173	1997	2046	2094	2162	1999	2128
Prrg3	1	8	14	8	7	16	13	7
Prrg4	187	260	268	238	284	251	225	215
Prrt1	36	48	30	23	18	40	23	41
Prrt2	20	14	16	16	34	13	29	12
Prrt3	0	0	0	0	0	0	1	0
Prrt4	2	0	1	1	1	7	9	0
Prrx1	732	949	712	698	788	711	866	741
Prrx2	12	5	11	16	37	20	12	13
Prrxl1	0	0	1	0	0	0	0	0
Prss1	2	0	0	0	4	0	1	7
Prss12	207	286	213	206	249	267	251	296
Prss16	123	178	167	164	238	242	226	204
Prss2	3692	4655	2162	955	18186	16929	13242	16281
Prss21	0	0	1	0	1	0	0	0
Prss22	7	3	6	3	17	3	5	7
Prss23	531	578	593	659	507	568	640	542
Prss27	121	181	91	63	150	139	130	188
Prss3	108	105	52	23	252	204	177	236
Prss30	1924	2444	2012	1526	2331	2572	2648	2251
Prss32	8609	9288	8665	8526	9514	9094	8641	9168
Prss33	1	1	0	0	1	0	0	0
Prss35	5	9	12	11	7	2	0	0
Prss36	23	36	31	45	27	22	21	28
Prss38	0	0	0	6	0	0	0	0
Prss39	0	0	1	0	0	0	0	0

Transcriptome sequencing yielded total genetic results for the MOD and APS groups, with a total of 15,936 variables

Continued from above

Prss41	17	16	13	16	31	8	6	5
Prss50	1	0	0	0	0	0	0	5
Prss53	13	7	15	4	19	8	5	1
Prss55	0	0	0	0	0	0	1	0
Prss57	10	1	1	4	4	8	3	2
Prss8	950	1031	928	1029	1004	926	984	956
Prtg	7	2	9	3	2	10	6	4
Prtn3	1	2	4	6	1	6	8	6
Prune1	845	864	780	852	981	784	891	805
Prune2	67	115	121	106	87	127	92	72
Prx	57	78	93	85	80	84	62	127
Prxl2a	5703	5757	5192	5505	5350	5759	7217	6553
Prxl2b	2946	3101	2924	2699	3061	3411	3356	3043
Prxl2c	775	855	902	853	757	866	812	921
Psap	20402	20663	20170	20775	20888	20763	18326	18880
Psat1	576	663	567	544	685	608	501	638
Psca	1	0	0	1	0	0	1	0
Psd	187	213	250	285	195	276	273	218
Psd2	0	6	0	6	1	6	1	2
Psd3	457	467	534	589	529	503	479	462
Psd4	1842	2126	1848	1834	1841	1894	1812	1905
Psen1	6925	7608	6564	6718	6518	6575	6532	6280
Psen2	393	448	384	420	380	368	337	397
Psenen	2414	2465	2454	2353	2221	2341	2196	2247
Psg16	1	0	0	1	0	0	0	4
Psg17	1	1	0	4	0	0	4	0
Psg19	0	0	1	0	0	0	0	0
Psg22	1	1	0	7	0	1	1	1
Psg25	66	101	124	69	82	113	97	92
Psg26	0	0	0	0	0	0	1	0
Psg27	0	1	0	0	0	0	0	0
Psip1	528	581	511	484	527	508	557	523
Pskh1	764	773	657	575	587	679	670	583
Psma1	5249	5462	5329	5373	5372	5313	4781	5026
Psma2	4183	4402	4291	4051	4086	4153	3744	3742
Psma3	2626	3224	2831	2704	3154	2996	2707	2997
Psma4	6055	6227	6323	6124	6213	6360	5933	5454
Psma5	3272	3331	3232	3273	3353	3100	2776	2816
Psma6	5124	5424	5220	5065	5766	5741	5511	5191
Psma7	5426	5756	5435	5485	5938	5552	4987	5119
Psma8	6	2	8	0	6	2	4	0
Psmb1	5254	5764	5793	5549	5977	5948	5572	5158
Psmb10	11721	11581	11106	11140	9559	10299	9236	8941
Psmb2	3371	3591	3527	3477	3424	3184	2878	3152
Psmb3	4573	4755	4663	4565	4883	4742	3993	4173
Psmb4	6595	6956	6410	6497	7046	6628	6269	6279
Psmb5	3462	3759	3650	3693	3889	3773	3524	3749
Psmb6	3858	4098	3779	4175	3892	3834	3851	3815
Psmb7	4145	4252	4247	4081	4558	4300	3887	4019
Psmb8	10946	10871	10272	10519	8551	9284	8576	7623
Psmb9	5892	5901	5718	5728	4832	4894	4233	4209
Psmc1	4304	4524	4161	4147	4317	4283	3962	4065
Psmc2	2673	2938	2571	2531	2699	2773	2730	2557
Psmc3	5206	5459	5324	5354	5592	5496	4904	5257
Psmc3ip	202	194	253	202	130	194	174	189
Psmc4	2887	3083	3210	2902	2971	2945	2832	2789
Psmc5	3461	3809	3560	3524	3348	3494	3477	3248
Psmc6	2810	2991	2940	2930	3321	3548	3388	2814

Transcriptome sequencing yielded total genetic results for the MOD and APS groups, with a total of 15,936 variables

Continued from above

Psmd1	4759	5424	5148	4692	5209	5034	4806	4431
Psmd10	773	866	711	774	840	918	843	739
Psmd11	2649	2873	2789	2757	2964	2906	2708	2726
Psmd12	2303	2298	2180	2093	2289	2309	2201	2177
Psmd13	4672	4719	4605	4275	4993	4902	4356	4563
Psmd14	2647	2779	2499	2659	2834	2837	2706	2478
Psmd2	6952	7058	6916	6462	7087	7341	6577	6225
Psmd3	5771	6141	5673	5541	5830	6099	5187	5303
Psmd4	3356	3453	3210	3148	3372	3478	3197	2887
Psmd5	938	1185	1110	1061	1211	1124	1030	1013
Psmd6	2552	2580	2339	2358	2600	2678	2477	2387
Psmd7	2729	3108	2614	2790	3006	2922	2823	2725
Psmd8	4655	4835	4796	4606	5030	4876	4687	4627
Psmd9	758	835	692	747	649	727	724	702
Psme1	9988	10685	10306	9728	9579	9343	9255	8436
Psme2	10713	11365	10722	10844	9932	10375	9655	9392
Psme2b	4608	4757	4758	4341	4229	4578	4078	4125
Psme3	4558	4812	4606	4615	5197	4964	4554	4630
Psme3ip1	1637	1667	1641	1649	1615	1594	1599	1559
Psme4	4186	4619	4220	4182	4753	4807	4711	4213
Psmf1	1464	1422	1438	1406	1356	1379	1247	1304
Psmg1	582	569	581	508	632	546	564	504
Psmg2	376	354	388	385	439	402	374	347
Psmg3	553	474	499	514	562	600	455	448
Psmg4	228	197	218	194	210	202	183	182
Psors1c2	0	0	1	4	0	0	0	0
Pspc1	586	590	504	482	460	445	422	468
Psph	193	260	244	277	262	254	278	263
Pspn	0	2	2	0	2	0	0	1
Psrc1	78	80	94	118	118	102	94	111
Pstk	381	436	381	418	393	477	372	416
Pstpip1	53	44	47	43	48	32	30	58
Pstpip2	3481	3593	3432	3300	3451	3376	3766	3600
Ptafr	316	275	243	203	301	345	315	332
Ptar1	762	775	793	814	757	673	659	607
Ptbp1	11082	11532	11548	11257	12305	11465	10479	10419
Ptbp2	205	240	219	228	197	217	183	213
Ptbp3	11014	12099	10543	10643	10209	11118	11539	10588
Ptcd1	1051	1182	1045	1025	1034	1070	1038	1013
Ptcd2	1305	1230	1057	1227	1305	1298	1090	1260
Ptcd3	1292	1371	1459	1401	1561	1435	1402	1302
Ptch1	1058	1102	979	1090	921	973	813	815
Ptch2	12	37	34	30	10	27	44	22
Ptchd1	1	8	2	3	2	5	3	0
Ptchd4	0	3	5	1	0	1	0	1
Ptdss1	4578	4416	4227	4496	4141	4013	4164	3742
Ptdss2	2292	2348	2474	2316	2521	2148	2303	2072
Pten	3752	3725	3713	3645	4081	4153	3869	3847
Pter	213	257	203	200	172	204	233	189
Ptf1a	8	0	0	0	10	5	1	5
Ptgdr	51	70	54	41	28	60	57	67
Ptgdr2	2	6	5	4	5	2	5	4
Ptgds	1	1	0	1	0	4	0	1
Ptger1	143	137	170	172	152	114	150	103
Ptger2	44	72	79	55	44	44	42	53
Ptger3	37	27	16	19	42	69	63	46
Ptger4	1249	1659	1674	1419	1740	1866	1938	1684
Ptges	253	304	299	304	322	294	267	290

Transcriptome sequencing yielded total genetic results for the MOD and APS groups, with a total of 15,936 variables

Continued from above

Ptges2	2730	2409	2674	2599	2443	2549	2393	2505
Ptges3	2827	2815	2663	2399	3113	3265	3129	2852
Ptges3-ps	698	744	666	658	869	912	728	725
Ptges3l	44	32	26	36	68	64	49	29
Ptgfr	2	1	0	1	7	5	6	4
Ptgfrn	7151	7927	7856	6929	7631	7722	8122	7092
Ptgir	15	32	11	8	20	13	14	25
Ptgis	63	64	62	75	54	68	64	55
Ptgr1	4500	5142	5014	4456	5332	5811	5849	5475
Ptgr2	2467	2527	2315	2367	2584	2668	2706	2538
Ptgs1	828	825	875	917	775	825	734	788
Ptgs2	83	106	91	110	122	111	93	69
Pth1r	46	50	72	44	65	59	34	59
Pth2r	0	0	0	0	4	0	0	0
Pthlh	29	39	34	19	33	27	26	34
Ptk2	1856	2209	1801	1892	1878	2055	1856	1687
Ptk2b	2116	1917	1983	1824	1879	2113	1949	1944
Ptk6	2694	2669	1971	1987	1997	2180	2205	2254
Ptk7	272	300	283	335	294	277	257	206
Ptma	19203	20870	19396	19067	20504	18707	16887	17292
Ptms	4095	4150	4435	4475	4078	4220	3587	3883
Ptn	61	75	73	83	75	97	75	58
Ptov1	997	955	1039	947	1054	1023	956	957
Ptp4a1	2212	2527	2269	2285	2545	2683	2687	2641
Ptp4a2	8567	9125	8984	8664	9465	9862	9192	8966
Ptp4a3	430	429	463	439	404	403	444	387
Ptpa	4908	5278	5200	5073	5254	5367	4595	4609
Ptpdc1	34	54	29	20	38	27	49	16
Ptpmt1	2182	2351	2209	2137	2287	2072	2133	2098
Ptpn1	1782	1711	1709	1798	1610	1625	1486	1644
Ptpn11	3297	3694	3296	3638	3675	3851	3563	3084
Ptpn12	1163	1286	1161	1149	1291	1243	1245	1252
Ptpn13	111	113	131	100	113	101	74	67
Ptpn14	175	196	179	174	181	173	183	154
Ptpn18	1826	1811	1771	1786	1649	1567	1685	1602
Ptpn2	1013	1055	1065	987	963	1139	1068	912
Ptpn20	0	0	0	0	0	6	0	0
Ptpn21	538	624	525	452	519	612	511	488
Ptpn22	298	370	368	431	371	376	400	372
Ptpn23	1459	1364	1282	1248	1432	1275	1523	1347
Ptpn3	2567	3015	2581	2704	2892	2936	3108	2735
Ptpn4	471	528	506	553	470	605	510	556
Ptpn5	2	6	5	2	11	3	1	0
Ptpn6	956	1150	966	1033	1002	1132	1066	1020
Ptpn7	113	109	145	130	134	151	75	103
Ptpn9	513	487	535	538	482	461	437	410
Ptpra	1576	1491	1708	1555	1400	1431	1399	1409
Ptprb	649	760	688	771	645	671	711	624
Ptprc	726	725	648	752	647	714	701	666
Ptprcap	104	83	54	95	37	92	59	63
Ptprd	683	739	782	718	850	820	833	699
Ptpre	851	833	784	785	721	793	867	873
Ptprf	14465	16551	14199	13243	15948	16133	16680	14674
Ptprg	1659	1745	1792	1916	1753	1797	1733	1686
Ptprh	10795	10803	9850	10128	9452	10005	10311	9221
Ptprj	1632	1832	1607	1739	1529	1584	1630	1631
Ptprk	2382	2566	2648	2502	2597	2598	2337	2251
Ptprm	194	229	234	256	154	169	191	165

Transcriptome sequencing yielded total genetic results for the MOD and APS groups, with a total of 15,936 variables

Continued from above

Ptprn	123	155	157	147	94	135	139	137
Ptprn2	733	698	762	762	744	750	638	687
Ptpro	67	55	89	52	42	53	59	53
Ptprq	0	0	1	0	0	1	1	4
Ptprr	1526	1448	1299	1321	1055	1171	1213	1187
Ptprs	315	419	475	438	304	331	303	408
Ptprt	15	12	10	4	13	7	11	2
Ptpru	75	47	82	69	35	60	65	94
Ptprz1	16	18	12	7	7	14	2	6
Ptrh1	519	561	510	530	486	454	520	500
Ptrh2	440	559	508	465	556	569	490	406
Ptrhd1	268	232	225	226	253	232	171	228
Pts	1021	1124	1077	1103	1159	1078	1077	1136
Pttg1	449	501	530	488	514	406	420	470
Pttg1ip	8391	9047	8248	8380	8217	8264	8091	7798
Ptx3	5	10	7	7	29	13	2	1
Ptx4	0	0	0	0	1	0	0	0
Puf60	3251	3255	3171	3360	3270	2998	2989	3015
Pum1	3996	4434	4266	3953	4052	4039	4044	3697
Pum2	4035	4720	4393	4527	4703	4733	4521	4209
Pum3	865	1024	996	906	1052	1000	930	1006
Pura	2290	2531	2581	2585	2453	2520	2519	2426
Purb	6014	6374	6171	6338	6494	6806	6389	6078
Purg	27	38	28	29	29	58	38	48
Pus1	1162	1145	1148	1087	1220	1171	970	984
Pus10	690	777	677	649	757	727	776	660
Pus3	139	211	145	169	234	248	232	239
Pus7	487	605	427	444	496	491	400	518
Pus7l	85	121	77	105	138	131	93	101
Pusl1	225	294	270	245	246	215	231	250
Pvr	545	623	534	547	567	557	641	576
Pvrig	8	9	20	18	22	18	11	30
Pwp1	857	836	795	798	925	939	868	839
Pwp2	398	557	444	454	568	410	491	522
Pwwp2a	306	364	360	368	374	387	383	275
Pwwp2b	613	664	635	653	561	631	546	505
Pwwp3a	540	608	659	583	608	564	501	508
Pwwp3b	11	9	5	13	10	22	3	6
Pxdc1	4472	4356	3871	4014	3610	3498	3783	3232
Pxdn	493	551	596	548	498	556	469	405
Pxk	3876	4225	3841	3937	3730	3934	3843	3746
Pxmp2	137	143	100	148	127	153	113	127
Pxmp4	1025	1177	1077	1042	1360	1379	1226	1228
Pxn	7137	7011	7220	6673	7494	7219	6744	6638
Pxt1	0	0	0	0	0	0	1	0
Pxylp1	432	431	511	413	346	428	400	332
Pycard	12494	12999	13475	12405	14534	13710	11707	12529
Pycr1	102	97	94	108	94	93	109	90
Pycr2	658	612	633	580	760	623	570	603
Pycrl	2960	3185	3123	3079	3206	3384	3073	2960
Pygb	1701	1906	1799	1721	1878	1919	1655	1693
Pygl	127	75	87	79	90	131	99	99
Pygm	63	42	55	68	78	48	52	58
Pygo1	17	23	13	15	9	18	19	12
Pygo2	1273	1267	1351	1232	1391	1401	1281	1239
Pym1	880	776	747	811	646	850	639	736
Pyroxd1	975	1021	947	915	908	1060	969	1008
Pyroxd2	245	250	196	236	217	244	188	165

Transcriptome sequencing yielded total genetic results for the MOD and APS groups, with a total of 15,936 variables

Continued from above

Pyurf	770	777	880	809	859	685	715	692
Pyy	6	13	13	2	14	4	12	13
Pzp	50	72	99	41	77	43	67	95
Qars	6404	6854	6617	6050	6607	6623	6517	6256
Qdpr	2820	2868	2847	2824	2628	2872	2644	2505
Qk	359	366	333	334	330	364	341	295
Qpct	70	60	90	82	79	88	49	84
Qpctl	302	292	325	256	364	302	267	321
Qprt	14	9	8	12	6	0	7	10
Qrfp	0	0	0	1	0	0	1	0
Qrfpr	0	0	0	0	0	0	1	0
Qrfprl	2	0	0	0	0	0	0	1
Qrich1	2136	2247	2175	2188	2326	2229	2109	2200
Qrich2	0	2	0	9	8	2	2	6
Qrsl1	475	482	509	539	452	527	516	490
Qser1	781	870	892	819	885	885	757	767
Qsox1	2192	2166	2433	2193	2604	2459	2361	2361
Qsox2	721	827	866	754	787	717	681	785
Qtrt1	241	263	265	229	244	205	196	248
Qtrt2	219	283	230	250	303	255	245	279
R3hcc1	353	389	351	394	307	363	271	300
R3hcc1l	551	575	583	613	639	581	627	586
R3hdm1	1057	1102	1129	1036	1175	1170	1128	1124
R3hdm2	2476	2702	2566	2521	2528	2587	2453	2396
R3hdm4	1690	1610	1849	1789	1771	1767	1438	1517
R3hdml	10	6	17	9	16	16	9	6
Rab10	6272	6728	6253	6408	6701	6769	6413	6243
Rab11a	5191	5351	4887	4818	5115	5225	5310	4902
Rab11b	3887	4042	3864	3817	3664	3707	3668	3551
Rab11fip1	5805	6593	5937	5933	6226	6509	6493	6121
Rab11fip2	114	127	56	102	82	76	97	98
Rab11fip3	2891	3032	2865	2758	3036	3393	3322	2885
Rab11fip4	1204	1163	1137	1163	1164	1181	1248	1028
Rab11fip5	88	113	81	121	98	102	81	93
Rab12	372	348	388	360	349	376	335	382
Rab13	117	129	132	126	144	131	101	131
Rab14	5903	5886	5543	5556	6013	5980	5911	5429
Rab15	1044	1139	1175	1129	1139	1127	1068	1108
Rab17	2388	2319	2193	2483	1840	2095	2067	2044
Rab18	4670	5005	5016	4765	5124	5121	4962	4724
Rab19	545	516	568	515	581	468	470	410
Rab1a	18263	19672	18077	17292	18559	19927	19160	17848
Rab1b	8453	8453	8265	8212	7987	8470	8297	7844
Rab20	855	854	825	846	643	716	760	686
Rab21	2622	2845	2488	2565	2797	2759	2634	2761
Rab22a	2918	2890	2902	2874	2866	2666	2903	2790
Rab23	127	118	121	123	125	139	140	148
Rab24	1662	2173	1818	1709	1586	1702	1800	1684
Rab25	4170	4200	4100	3945	4053	4033	3637	3905
Rab26	4	16	1	6	5	2	7	2
Rab27a	743	745	807	880	785	848	743	727
Rab27b	639	610	637	708	757	728	710	669
Rab28	1100	1022	1079	1055	1101	1080	971	899
Rab29	245	253	259	253	245	223	211	174
Rab2a	6361	6821	6236	6526	6941	7230	6676	6247
Rab2b	123	122	105	66	67	88	108	102
Rab30	716	789	838	951	1399	1476	1982	1952
Rab31	610	652	543	550	716	596	518	532

Transcriptome sequencing yielded total genetic results for the MOD and APS groups, with a total of 15,936 variables

Continued from above

Rab32	595	474	469	545	479	466	484	474
Rab33a	6	7	5	6	3	1	4	1
Rab33b	754	764	741	758	773	731	690	742
Rab34	110	100	118	122	99	114	81	98
Rab35	2836	2625	2743	2557	2608	2661	2583	2535
Rab36	11	9	10	4	4	7	12	4
Rab37	90	92	100	63	54	63	95	59
Rab38	5	14	3	13	10	12	10	2
Rab39	4	13	2	8	4	6	6	5
Rab39b	4	4	3	19	17	19	8	28
Rab3a	174	212	183	203	192	150	160	175
Rab3b	44	22	25	32	33	25	20	34
Rab3c	108	113	139	121	101	126	108	102
Rab3d	1793	1801	1896	1862	1801	1741	1545	1619
Rab3gap1	1882	2045	1797	1923	1862	2060	1954	1870
Rab3gap2	2370	2625	2406	2324	2426	2886	2491	2572
Rab3il1	144	103	98	155	171	119	126	99
Rab3ip	1471	1694	1536	1415	1763	1675	1450	1559
Rab40b	14	10	21	21	15	5	3	1
Rab40c	821	924	965	842	1097	975	847	871
Rab42	13	3	14	5	8	7	2	14
Rab43	6252	7057	6572	6019	7144	7043	6885	6552
Rab44	3	9	4	2	23	10	1	4
Rab4a	1855	1994	1883	1785	1831	1933	1926	1991
Rab4b	1458	1589	1414	1412	1301	1352	1307	1388
Rab5a	2732	2744	2760	2851	2863	3056	2976	2702
Rab5b	2594	2906	2766	2546	2599	2828	2652	2605
Rab5c	15965	15958	14785	14657	14446	14740	14152	13735
Rab5if	5182	5181	5010	4869	5144	5220	4507	4615
Rab6a	7787	8372	7966	8339	9102	9243	8797	8132
Rab6b	91	152	86	105	89	106	131	73
Rab7	8856	9104	8842	8914	8982	9227	8500	8486
Rab7b	73	91	96	107	63	45	57	50
Rab8a	9121	9767	9123	9000	9196	9588	9194	8642
Rab8b	850	1136	814	930	874	997	995	893
Rab9	1137	1319	1121	1230	1159	1249	1277	1286
Rab9b	0	1	0	1	1	1	1	7
Rabac1	1509	1323	1482	1532	1411	1553	1328	1506
Rabep1	1023	1215	1195	1135	1257	1370	1208	1056
Rabep2	493	479	527	488	493	479	517	510
Rabepk	983	1107	1103	900	1163	1252	1072	976
Rabgap1	1117	1245	1073	1163	1164	1218	1099	1021
Rabgap1l	1878	2036	1895	1802	1870	2094	2219	2004
Rabgef1	886	850	831	788	880	833	803	851
Rabggta	904	992	869	1006	896	855	817	831
Rabggtb	1013	1033	1121	1080	1099	1179	1135	1011
Rabif	1354	1395	1450	1365	1340	1416	1364	1249
Rabl2	83	39	80	69	64	71	88	57
Rabl3	721	803	644	777	767	739	800	719
Rabl6	2374	2635	2502	2312	2613	2396	2495	2283
Rac1	17510	17969	16660	16001	16754	17019	16784	16503
Rac2	430	409	335	396	415	364	328	322
Rac3	26	54	42	38	37	47	22	45
Racgap1	1113	1329	1248	1042	1234	1014	1113	1090
Rack1	28535	29909	29986	29843	29713	29974	26345	28090
Rad1	170	178	184	161	232	177	160	189
Rad17	487	449	481	441	552	530	531	473
Rad18	216	248	197	155	147	192	145	139

Transcriptome sequencing yielded total genetic results for the MOD and APS groups, with a total of 15,936 variables

Continued from above

Rad21	6379	7040	6683	6587	6462	6505	6298	6200
Rad21l	1	4	1	11	1	1	0	1
Rad23a	1789	1946	1854	1822	1765	1827	1592	1680
Rad23b	5993	6592	6136	6208	6537	6577	6108	6054
Rad50	631	662	658	610	673	651	577	634
Rad51	440	569	552	506	554	515	500	500
Rad51ap1	176	209	203	187	157	216	127	150
Rad51ap2	0	0	0	0	0	4	0	0
Rad51b	47	31	48	64	48	75	33	44
Rad51c	43	32	20	28	34	23	29	44
Rad51d	586	697	642	612	593	606	647	559
Rad52	120	111	119	77	123	117	104	97
Rad54b	58	122	77	60	80	122	67	51
Rad54l	474	499	501	481	524	488	415	468
Rad54l2	1399	1600	1719	1548	1389	1497	1338	1431
Rad9a	291	331	283	329	290	252	250	283
Rad9b	60	43	52	30	29	36	44	39
Radil	11	20	9	8	22	11	3	20
Radx	39	45	61	53	73	72	43	35
Rae1	1040	986	1054	1062	1114	1036	1006	930
Raet1d	0	1	1	4	0	1	1	1
Raet1e	15	15	25	24	5	42	30	16
Raf1	3655	4125	3909	3702	3764	3898	3570	3189
Rai1	838	770	832	796	815	689	605	664
Rai14	453	474	436	379	483	525	464	441
Rai2	92	58	66	78	107	85	70	73
Rala	2493	2768	2697	2434	2688	2777	2726	2600
Ralb	2615	2740	2684	2707	2795	2810	2635	2782
Ralbp1	3240	3448	3367	3182	3373	3240	3378	3115
Ralgapa1	1231	1371	1265	1378	1374	1353	1314	1249
Ralgapa2	3770	4195	3780	3787	3781	4111	4434	3947
Ralgapb	2775	3164	2900	3131	3352	3164	3247	3012
Ralgds	2604	2804	2726	2613	2613	2658	2678	2696
Ralgps1	780	854	903	781	965	1001	1054	884
Ralgps2	2947	2838	2955	3000	2888	3010	2816	2699
Raly	5059	5009	5132	4850	5465	5255	4922	4894
Ralyl	2	3	5	0	7	1	4	2
Ramac	1600	1596	1528	1759	1616	1688	1708	1559
Ramp1	249	233	250	336	309	280	232	245
Ramp2	157	123	166	161	139	128	127	165
Ramp3	21	22	23	45	34	20	20	35
Ran	6191	7012	6076	6338	6581	6211	5875	5958
Ranbp1	2412	2659	2450	2315	2546	2581	2053	2300
Ranbp10	1248	1276	1361	1327	1221	1234	1114	1138
Ranbp17	5	5	1	7	0	9	1	2
Ranbp2	3239	3906	3489	3588	4257	4083	4377	3676
Ranbp3	1711	1953	1847	1890	1808	1886	1632	1779
Ranbp3l	0	1	0	0	0	1	1	0
Ranbp6	505	448	449	482	595	574	592	534
Ranbp9	1773	1774	1666	1520	1716	1709	1691	1697
Rangap1	2856	3028	2815	2928	3282	3254	2808	2929
Rangrf	402	423	518	516	488	511	420	403
Rap1a	2660	2941	2862	2903	2900	3077	3137	2760
Rap1b	5084	5505	4987	5116	5303	5377	5278	5024
Rap1gap	940	971	1093	969	1088	1013	960	1093
Rap1gap2	1266	1261	1434	1442	1309	1407	1287	1269
Rap1gds1	2267	2258	2294	2264	2156	2162	2242	2060
Rap2a	415	437	450	497	552	446	414	464

Transcriptome sequencing yielded total genetic results for the MOD and APS groups, with a total of 15,936 variables

Continued from above

Rap2b	400	423	401	411	377	395	337	408
Rap2c	3444	4057	3547	3181	3986	4446	4403	4131
Rapgef1	1816	1916	2119	1891	1964	2046	1773	1746
Rapgef2	913	939	987	813	1056	1103	1075	963
Rapgef3	148	157	151	161	187	146	146	157
Rapgef4	25	32	53	59	61	46	57	47
Rapgef5	526	717	606	632	596	656	600	605
Rapgef6	1709	1723	1712	1552	1797	1826	1779	1700
Rapgefl1	820	827	878	809	770	662	718	605
Raph1	5413	5152	4852	5692	4074	4267	4867	4142
Rapsn	0	1	3	1	1	0	2	0
Rara	1174	1171	1202	1223	1188	1116	1050	1091
Rarb	46	68	75	108	63	67	46	54
Rarg	130	136	160	147	171	153	89	128
Rarres1	26	21	14	23	14	15	11	26
Rarres2	754	799	811	834	776	751	752	633
Rars	3907	4183	3840	3972	4602	4488	4223	3832
Rars2	961	1079	1011	1001	1118	1052	1124	1000
Rasa1	1339	1479	1239	1320	1503	1498	1597	1335
Rasa2	1234	1497	1334	1406	1543	1616	1567	1512
Rasa3	916	1074	1085	1030	1006	993	918	879
Rasa4	973	955	1119	1076	879	859	755	851
Rasal1	17	21	13	19	34	22	7	21
Rasal2	398	425	412	407	388	346	355	412
Rasal3	146	99	128	136	123	173	113	155
Rasd1	305	636	545	292	244	298	351	389
Rasd2	254	177	183	161	132	188	146	182
Rasef	823	953	1035	937	1241	1153	1177	1124
Rasgef1a	2	1	4	4	6	1	5	1
Rasgef1b	728	644	592	631	584	757	847	667
Rasgef1c	7	16	4	11	12	10	7	3
Rasgrf1	3	7	6	2	0	1	0	1
Rasgrf2	1270	1216	1125	1384	1182	1295	1059	1030
Rasgrp1	96	65	98	107	120	122	68	97
Rasgrp2	81	84	111	109	92	119	123	110
Rasgrp3	106	113	118	116	123	128	82	118
Rasgrp4	19	33	18	13	31	32	12	30
Rasip1	170	149	177	164	179	142	135	155
Rasl10a	7	5	1	7	1	2	0	1
Rasl10b	5	32	13	17	4	11	33	19
Rasl11a	58	67	47	64	77	60	50	36
Rasl11b	28	53	49	48	74	52	40	51
Rasl12	102	133	107	133	93	113	163	137
Rasl2-9	34	20	21	25	12	17	30	21
Rassf1	1422	1401	1298	1374	1351	1540	1501	1473
Rassf10	6	11	18	3	21	18	27	12
Rassf2	199	226	218	255	205	261	181	209
Rassf3	3055	3174	3354	3296	3387	3845	3279	3186
Rassf4	1696	1836	1820	1541	1934	1926	1890	1899
Rassf5	195	214	203	223	184	206	182	163
Rassf6	709	657	728	636	841	722	760	804
Rassf7	2239	2309	2288	2191	2622	2522	2482	2199
Rassf8	47	112	66	88	89	74	31	66
Rassf9	18	12	16	8	17	12	11	11
Raver1	5628	6030	5867	5518	5613	5489	5139	5057
Raver2	24	20	66	39	45	36	41	38
Rb1	2737	2941	2965	2994	2427	2605	2652	2496
Rb1cc1	1849	2143	1754	1833	2031	2270	2236	2113

Transcriptome sequencing yielded total genetic results for the MOD and APS groups, with a total of 15,936 variables

Continued from above

Rbak	124	109	119	130	131	123	107	90
Rbbp4	3127	3366	3297	3049	3548	3553	3255	3375
Rbbp5	1042	1242	1128	1157	1209	1157	1080	1030
Rbbp6	2099	1936	2106	2327	2105	2249	2144	1835
Rbbp7	4275	4291	4272	4102	4273	4284	3898	4003
Rbbp8	481	535	511	528	637	702	620	556
Rbbp8nl	41	71	42	45	61	44	56	30
Rbbp9	590	565	602	655	448	472	492	438
Rbck1	4326	4389	4152	4100	4059	4109	4125	3951
Rbfa	729	812	829	805	884	857	704	783
Rbfox1	7	7	5	0	4	13	3	4
Rbfox2	933	982	1073	970	984	900	832	873
Rbfox3	1	2	6	9	6	9	1	0
Rbis	562	592	624	599	591	599	596	659
Rbks	681	749	817	720	700	849	824	781
Rbl1	1310	1590	1423	1317	1126	1365	1363	1227
Rbl2	1550	1618	1558	1603	1682	1534	1635	1414
Rbm10	1819	2072	1874	1782	2136	2155	1950	1928
Rbm11	2	1	5	0	5	1	6	1
Rbm12	695	672	753	675	661	738	648	647
Rbm12b1	57	51	35	62	107	93	52	68
Rbm12b2	186	252	202	197	223	237	228	208
Rbm14	1176	1357	1330	1180	1554	1381	1226	1333
Rbm15	576	626	684	562	762	800	682	618
Rbm15b	1877	2218	2123	2135	2340	2224	1968	1959
Rbm17	1347	1403	1389	1142	1545	1472	1329	1299
Rbm18	576	670	586	631	673	622	664	599
Rbm19	555	543	593	558	601	541	586	514
Rbm20	81	115	108	101	137	116	73	85
Rbm22	942	1109	1003	954	1009	940	955	971
Rbm24	54	48	61	65	38	43	44	44
Rbm25	2278	2437	2622	2706	2617	2818	2554	2327
Rbm26	1411	1320	1228	1349	1363	1429	1343	1302
Rbm27	1209	1314	1284	1280	1514	1452	1224	1374
Rbm28	949	1006	892	886	897	916	829	920
Rbm3	5746	5734	5593	5600	5524	5365	4781	4970
Rbm3-ps	171	211	161	145	194	137	165	150
Rbm33	1822	2061	2125	2132	1856	1938	1827	1812
Rbm34	483	500	550	478	578	531	491	496
Rbm38	382	408	421	361	328	360	346	319
Rbm39	5224	5600	5170	5504	5715	6150	5824	5403
Rbm4	21	22	16	11	22	31	23	29
Rbm41	257	282	290	335	308	296	341	275
Rbm42	2882	2982	2935	2815	2851	2846	2716	2526
Rbm43	261	368	299	257	229	292	247	214
Rbm44	5	0	0	0	1	0	0	0
Rbm45	1159	1321	1220	1236	1167	1298	1258	1198
Rbm46	16	19	5	16	9	20	16	6
Rbm47	9832	9984	9642	9449	9152	9507	9567	9419
Rbm48	135	158	147	103	125	135	111	135
Rbm4b	126	99	122	139	155	139	106	124
Rbm5	2143	2248	2402	2243	2441	2507	2185	2246
Rbm6	1599	1818	1711	1714	1837	1934	1813	1715
Rbm7	1743	1768	1723	1594	1947	1961	1871	1641
Rbm8a	1679	1739	1591	1635	1699	1847	1667	1598
Rbms1	417	507	461	512	468	484	428	449
Rbms2	854	1107	931	966	1051	909	775	885
Rbms3	56	102	83	116	115	90	60	68

Transcriptome sequencing yielded total genetic results for the MOD and APS groups, with a total of 15,936 variables

Continued from above

Rbmx	1008	970	903	1032	922	835	806	792
Rbmx2	134	124	156	144	132	133	112	131
Rbmxl1	758	779	850	689	892	748	703	706
Rbp1	102	99	125	149	130	109	134	141
Rbp2	115611	119269	113422	116635	112457	116470	114181	111892
Rbp4	141	133	145	144	126	114	96	117
Rbp7	218	252	224	286	359	328	267	374
Rbpj	880	911	951	1022	868	887	934	920
Rbpjl	3	5	15	9	57	33	29	31
Rbpms	547	592	544	612	488	551	581	603
Rbpms2	99	98	157	115	76	116	134	138
Rbsn	1179	1226	1226	1171	1268	1295	1207	1191
Rbx1	1632	1594	1745	1816	1831	1625	1515	1630
Rc3h1	1830	1972	2120	2102	2438	2466	2261	2131
Rc3h2	1241	1579	1392	1385	1471	1481	1423	1291
Rcan1	1440	1531	1388	1467	1349	1566	1589	1337
Rcan2	10	16	5	8	9	20	12	22
Rcan3	299	327	398	347	265	332	312	263
Rcbtb1	2653	2856	2731	2855	2994	2931	2948	2483
Rcbtb2	534	548	554	524	521	613	525	462
Rcc1	1428	1606	1415	1476	1629	1576	1305	1402
Rcc1l	735	719	599	633	654	707	520	732
Rcc2	3278	3259	3227	2839	3207	2979	2703	2671
Rccd1	125	142	76	127	91	91	112	81
Rce1	755	816	710	737	766	799	702	623
Rchy1	1282	1346	1262	1324	1450	1307	1298	1162
Rcl1	681	749	709	598	750	752	661	677
Rcn1	372	484	428	390	484	467	383	396
Rcn2	685	712	684	682	799	726	645	567
Rcn3	256	234	281	275	299	339	260	280
Rcor1	2751	3137	3123	3230	3243	3077	3018	2825
Rcor2	38	37	45	31	32	23	40	28
Rcor3	733	680	793	691	545	625	675	592
Rcsd1	263	256	201	239	205	191	189	191
Rd3	3	13	5	7	1	12	0	4
Rdh1	8	5	7	3	4	10	6	4
Rdh10	1794	1859	1735	1748	1863	1774	1791	1841
Rdh11	2101	2420	2418	2364	2193	2212	2127	2240
Rdh12	2	10	2	3	14	9	20	5
Rdh13	1229	1318	1440	1358	1411	1410	1346	1214
Rdh14	1135	1284	1085	1132	1178	1112	1179	1121
Rdh16	480	568	425	448	447	475	508	460
Rdh16f2	71	89	73	66	71	71	60	76
Rdh19	4	0	0	2	0	1	1	2
Rdh5	167	136	189	183	143	119	165	159
Rdh7	1716	2130	1808	1514	2045	2432	2197	2070
Rdh9	1735	2059	1578	1443	1732	1908	2101	1834
Rdm1	222	220	250	238	255	213	312	263
Rdx	356	352	417	350	372	378	303	311
Rec114	2	0	0	3	2	0	0	1
Rec8	1563	1662	1811	1720	1571	1655	1592	1444
Reck	88	83	109	69	88	55	90	51
Recql	428	508	517	437	486	490	431	398
Recql4	127	161	158	137	149	159	150	168
Recql5	600	733	522	604	558	541	471	543
Reep1	18	30	28	37	37	11	25	35
Reep2	17	23	19	18	13	15	5	23
Reep3	6242	7300	6271	5860	6518	6762	6596	6392

Transcriptome sequencing yielded total genetic results for the MOD and APS groups, with a total of 15,936 variables

Continued from above

Reep4	1071	1219	1185	1153	1404	1222	1162	1181
Reep5	427	471	523	466	575	539	523	538
Reep6	12160	12858	12930	12064	12128	12132	12114	11546
Reg1	25954	30720	28132	22609	37817	40372	30890	27369
Reg2	1423	1923	1006	482	12559	11946	11510	13638
Reg3a	2777	3274	2408	2016	4781	4814	4888	4816
Reg3b	631218	688290	664631	633129	636004	692439	838602	843442
Reg3d	133	119	79	24	649	697	587	789
Reg3g	250591	266322	256571	255239	267122	287476	342067	341087
Reg4	1495	1548	1630	1563	1676	1641	1539	1588
Rel	276	396	243	233	297	341	292	291
Relb	2301	2239	2067	2190	2120	2396	2435	2219
Relch	2083	2140	2014	2020	1935	2076	1952	1821
Rell1	591	674	693	678	635	659	714	628
Rell2	21	16	7	19	6	8	18	13
Reln	161	195	214	209	166	209	178	172
Relt	47	38	51	41	34	39	33	32
Rem1	36	22	24	37	18	31	31	10
Rem2	4	7	10	5	0	5	2	3
Ren1	6	0	0	1	3	0	5	3
Renbp	130	111	106	87	85	85	99	88
Rep15	444	431	441	495	420	432	477	467
Repin1	156	130	174	139	91	150	116	139
Reps1	447	501	522	546	380	444	378	409
Reps2	697	745	688	828	604	741	681	605
Rer1	7411	7625	7286	7186	7603	7301	7046	6858
Rere	4407	4729	4915	4513	5024	4817	4629	4487
Rerg	54	82	58	60	48	58	29	67
Rergl	0	0	0	1	0	0	0	0
Resf1	1080	1368	1239	1270	1106	1311	1388	1247
Resp18	44	87	52	59	80	50	102	72
Rest	564	636	609	596	639	719	533	566
Ret	275	263	227	269	259	262	305	365
Retn	7	5	18	5	28	68	44	25
Retnla	0	0	4	0	2	2	6	0
Retnlb	389	179	256	462	520	490	564	692
Retnlg	16	31	17	6	12	17	10	2
Retreg1	674	713	750	696	600	788	781	681
Retreg2	2144	1858	1981	1835	2223	2103	2070	1955
Retreg3	4016	4278	4050	3891	4037	4331	4471	3881
Retsat	3814	4128	3091	3062	3980	4036	3603	3267
Rev1	287	339	395	352	345	367	373	318
Rev3l	809	889	837	729	854	890	799	727
Rex1bd	933	952	921	841	964	1035	935	860
Rex2	0	0	0	0	4	0	0	0
Rexo1	1991	2110	2057	1937	1925	2079	1707	1753
Rexo2	658	701	737	650	750	729	715	763
Rexo4	1406	1446	1273	1456	1650	1768	1614	1599
Rexo5	82	98	94	81	89	68	95	83
Rfc1	1011	1079	1022	1140	1142	1126	1044	1039
Rfc2	1185	1156	1105	1169	1253	1128	1085	1076
Rfc3	418	470	481	385	388	423	305	303
Rfc4	454	451	413	533	395	439	441	565
Rfc5	922	944	920	869	954	845	745	760
Rfesd	278	235	262	186	226	213	235	200
Rffl	798	768	748	669	679	692	695	659
Rfk	42485	40078	38868	44210	31999	33819	38925	36555
Rflna	4	2	22	8	12	15	18	11

Transcriptome sequencing yielded total genetic results for the MOD and APS groups, with a total of 15,936 variables

Continued from above

Rflnb	3184	3853	3749	3242	4190	4374	3766	4003
Rfng	1317	1333	1222	1247	1325	1302	1258	1187
Rft1	376	341	334	310	404	350	236	327
Rftn1	171	197	186	276	192	156	157	189
Rftn2	65	58	51	68	54	75	67	47
Rfwd3	2110	2185	2182	2256	2344	2358	2110	1934
Rfx1	1234	1016	1126	1000	1127	1140	1087	1051
Rfx2	52	45	53	48	105	69	92	83
Rfx3	131	176	170	163	211	153	160	140
Rfx4	0	0	1	0	0	0	1	5
Rfx5	516	550	485	494	470	526	385	429
Rfx6	39	60	41	35	72	32	47	59
Rfx7	1231	1297	1241	1284	1372	1510	1221	1084
Rfxank	1169	1179	1283	1221	1177	1262	1028	1290
Rfxap	360	471	418	417	442	408	381	388
Rgcc	127	122	140	97	157	111	98	106
Rgl1	323	276	289	276	269	290	253	228
Rgl2	882	821	890	947	849	751	855	851
Rgl3	49	29	25	38	23	39	27	19
Rgma	71	101	106	77	121	60	67	48
Rgmb	1193	1356	1324	1374	1318	1383	1194	1146
Rgn	262	229	290	248	242	271	200	228
Rgp1	2492	2823	2578	2457	2514	2485	2521	2145
Rgs1	112	180	136	141	132	83	155	131
Rgs10	254	214	168	221	250	264	232	163
Rgs11	8	15	14	10	17	18	7	13
Rgs12	235	217	234	247	201	195	146	167
Rgs13	49	23	12	26	26	36	12	30
Rgs14	103	90	87	68	136	144	99	70
Rgs16	17	5	10	20	13	19	9	3
Rgs17	16	17	17	15	20	10	24	15
Rgs18	1	0	1	0	0	6	0	1
Rgs19	90	81	106	107	100	84	121	87
Rgs2	506	569	549	564	458	498	516	457
Rgs20	1	2	0	0	0	0	1	0
Rgs22	4	5	1	3	0	17	4	1
Rgs3	1316	1260	1266	1296	1372	1321	1211	1163
Rgs4	98	124	114	83	67	89	81	91
Rgs5	2316	2435	2533	2582	2578	2807	2796	2343
Rgs6	19	23	15	40	31	28	19	21
Rgs7	1	1	0	1	3	5	5	6
Rgs7bp	38	58	53	66	39	58	75	44
Rgs8	0	0	0	1	0	1	0	2
Rgs9	131	154	179	198	121	111	156	137
Rgs9bp	0	0	0	5	0	0	0	0
Rgsl1	1	1	0	1	1	0	0	0
Rhag	0	0	1	0	4	0	0	1
Rhbdd1	853	1026	874	963	841	899	810	789
Rhbdd2	576	590	620	554	712	610	547	627
Rhbdd3	298	340	269	287	371	350	260	325
Rhbdf1	384	417	431	405	407	344	382	263
Rhbdf2	1419	1619	1428	1501	1421	1437	1507	1505
Rhbdl1	113	111	158	109	141	160	112	140
Rhbdl2	688	820	730	811	781	775	697	790
Rhbdl3	23	41	46	38	32	23	25	37
Rhbg	805	964	772	815	889	825	784	730
Rhcg	6	0	1	1	0	1	0	0
Rhd	0	0	1	0	1	5	0	0

Transcriptome sequencing yielded total genetic results for the MOD and APS groups, with a total of 15,936 variables

Continued from above

Rheb	1546	1743	1554	1691	1929	1824	1726	1746
Rhebl1	661	788	632	523	597	702	548	527
Rhno1	446	456	440	440	472	439	337	451
Rho	5	1	0	0	5	1	4	1
Rhoa	12897	13488	13154	12415	12689	13459	12578	12278
Rhob	4180	4303	4334	4070	3613	3891	4283	4011
Rhobtb1	136	150	186	165	162	176	133	118
Rhobtb2	408	436	461	460	490	442	400	463
Rhobtb3	406	460	480	337	458	500	477	435
Rhoc	8502	8427	7636	7842	7819	8532	8644	8219
Rhod	2381	2471	2039	2228	2041	2481	2447	2327
Rhof	1806	1788	1550	1732	1789	1984	1946	1737
Rhog	2428	2370	2208	2078	2201	2353	2216	2064
Rhoh	82	109	48	43	55	50	54	62
Rhoj	300	328	330	332	313	297	292	264
Rhoq	1004	1267	1304	1165	995	1106	1087	999
Rhot1	1317	1419	1327	1286	1375	1487	1456	1174
Rhot2	3867	4204	3768	4185	3648	3604	3858	3853
Rhou	3671	4286	4137	3812	5102	5304	5044	4458
Rhov	50	56	58	40	52	73	78	62
Rhox5	0	1	5	4	12	4	10	1
Rhox6	0	4	0	0	1	0	0	1
Rhpn1	1	2	0	1	0	5	1	0
Rhpn2	4501	5231	5266	4936	5302	5013	4906	4703
Ribc1	44	28	29	46	33	39	41	39
Ric1	1303	1544	1340	1369	1445	1501	1411	1284
Ric3	35	34	52	35	21	26	38	34
Ric8a	2097	2006	1840	1747	1951	1762	1849	1846
Ric8b	430	527	496	464	547	587	451	524
Rictor	702	899	740	822	712	771	774	688
Rida	228	238	202	283	266	313	244	276
Rif1	725	798	729	739	965	915	898	814
Riiad1	6	4	5	8	4	4	13	4
Rilp	525	581	586	534	534	603	461	532
Rilpl1	58	59	68	49	77	62	68	96
Rilpl2	1393	1480	1437	1276	1588	1680	1532	1426
Rimbp2	46	74	101	98	61	68	69	56
Rimbp3	6	18	16	4	13	18	4	9
Rimkla	6	11	1	10	6	0	9	5
Rimklb	1	1	0	0	5	0	0	0
Rims1	152	196	230	206	172	175	170	197
Rims2	19	73	57	27	51	68	76	68
Rims3	32	38	24	34	23	22	19	29
Rims4	5	8	2	12	3	16	3	5
Rin1	186	163	165	178	174	181	116	150
Rin2	967	980	889	947	989	827	865	903
Rin3	208	247	184	193	226	226	215	201
Ring1	495	473	449	453	492	539	502	432
Rinl	176	162	149	175	148	193	127	133
Rint1	664	743	725	620	796	849	678	773
Riok1	547	621	532	465	573	670	574	553
Riok2	696	732	655	738	639	660	769	689
Riok3	4559	4764	4154	4418	4699	4914	5137	4321
Riox1	610	582	671	573	658	689	624	642
Riox2	750	772	717	715	747	776	823	759
Ripk1	2589	2803	2547	2637	2747	2888	2801	2765
Ripk2	182	224	176	200	189	178	160	176
Ripk3	2702	2958	2484	2632	2643	2701	2496	2324

Transcriptome sequencing yielded total genetic results for the MOD and APS groups, with a total of 15,936 variables

Continued from above

Ripk4	1656	1746	1607	1756	1784	1807	1728	1752
Ripor1	344	352	337	342	354	325	287	321
Ripor2	41	116	58	86	72	85	46	92
Ripor3	44	52	57	43	63	51	47	46
Ripply1	2	0	0	4	1	4	0	1
Ripply2	0	1	0	0	0	0	0	0
Ripply3	33	36	42	33	24	32	43	39
Rit1	713	804	705	722	663	745	675	735
Rit2	7	6	16	11	9	8	18	11
Rita1	213	159	186	230	201	193	193	164
Rlbp1	0	0	0	1	0	0	1	0
Rlf	691	717	789	711	761	823	904	760
Rlim	2120	2378	2148	2234	2338	2548	2306	2346
Rln3	0	1	1	3	0	1	0	0
Rmc1	922	1007	924	986	1039	1072	1083	997
Rmdn1	612	655	624	603	581	676	632	557
Rmdn2	198	209	212	182	259	200	277	210
Rmdn3	11328	11618	10452	10726	10877	11750	11267	9977
Rmi1	365	394	346	401	438	391	408	476
Rmi2	117	114	94	84	112	89	69	81
Rmnd1	751	737	661	796	795	853	732	798
Rmnd5a	2598	2785	2872	3027	3136	3112	3185	3041
Rmnd5b	550	560	597	617	579	574	608	571
Rnase1	1454	1842	1053	777	6028	5828	4421	5291
Rnase10	0	0	0	1	0	0	0	2
Rnase12	11	8	1	2	0	4	0	2
Rnase2a	1	0	0	0	0	0	0	0
Rnase2b	1	13	6	9	2	0	13	0
Rnase4	2801	2844	2622	2774	2772	2945	2665	2573
Rnase6	100	87	58	88	88	74	54	73
Rnaseh1	275	228	213	270	211	190	234	314
Rnaseh2a	636	616	689	644	546	603	573	543
Rnaseh2b	288	365	304	293	312	322	270	273
Rnaseh2c	1187	1228	1318	1278	1309	1206	1097	1091
Rnasek	2498	2376	2527	2376	2217	2228	2308	2273
Rnasel	7129	7548	6865	7109	6715	6947	7053	6749
Rnaset2a	1643	1589	1553	1579	1445	1692	1512	1483
Rnaset2b	1622	1406	1438	1524	1350	1511	1242	2006
Rnd1	37	43	25	15	37	29	38	34
Rnd2	800	706	772	753	652	831	726	773
Rnd3	488	468	602	469	637	527	478	560
Rnf10	9789	10350	10195	9702	10488	10099	10028	9753
Rnf103	3068	3289	3113	3244	3081	3231	3455	3383
Rnf11	1731	1785	1850	1646	1949	1878	1797	1815
Rnf111	1150	1218	1219	1260	1396	1287	1306	1158
Rnf112	3	2	1	0	0	5	8	2
Rnf113a1	116	90	113	112	131	113	157	118
Rnf113a2	281	328	270	254	301	347	265	308
Rnf114	6082	6231	6423	5924	5926	6257	5933	5984
Rnf115	3637	3928	3456	3590	3390	3621	3622	3457
Rnf121	1048	1321	1176	1092	1244	1270	1279	1136
Rnf122	65	60	69	24	28	59	32	38
Rnf123	1755	1852	1754	1721	1592	1575	1398	1521
Rnf125	2055	2653	2119	1695	2549	2695	2281	2348
Rnf126	1532	1604	1602	1622	1456	1553	1288	1406
Rnf128	13950	14774	13149	13473	14683	16281	16746	15471
Rnf13	1978	2101	1994	2067	2006	2242	2157	2062
Rnf130	421	444	408	438	430	429	463	443

Transcriptome sequencing yielded total genetic results for the MOD and APS groups, with a total of 15,936 variables

Continued from above

Rnf133	0	4	0	4	4	0	0	0
Rnf135	351	306	371	281	467	377	308	349
Rnf138	430	534	537	498	525	545	507	446
Rnf139	1586	1974	1617	1586	1793	2133	2028	1772
Rnf14	2778	2721	2771	2692	2756	2740	2800	2387
Rnf141	2890	3277	3047	2933	3012	3403	3491	3179
Rnf144a	97	113	100	114	131	92	62	110
Rnf144b	333	315	348	271	242	244	295	211
Rnf145	2126	2026	2346	2128	2300	2127	1929	2225
Rnf146	1030	1144	1013	1176	1121	1209	1116	1052
Rnf148	0	0	0	0	1	1	0	0
Rnf149	1680	1656	1444	1575	1607	1661	1505	1533
Rnf150	68	51	43	92	64	89	55	61
Rnf151	1	0	1	0	0	0	1	1
Rnf152	933	844	999	922	859	867	765	719
Rnf157	155	170	209	194	136	151	113	152
Rnf165	1	2	1	0	2	3	2	3
Rnf166	725	755	744	713	818	800	751	793
Rnf167	2229	2264	2204	2131	2388	2373	2263	2054
Rnf168	629	733	729	578	753	739	711	718
Rnf169	437	546	530	513	566	564	472	471
Rnf17	1	0	7	1	2	10	3	3
Rnf170	711	841	674	762	767	849	766	810
Rnf180	8	18	17	27	6	6	10	27
Rnf181	4593	4937	4466	4100	4516	4801	4497	4434
Rnf182	1	0	0	0	0	1	0	0
Rnf183	14	5	9	3	5	1	14	7
Rnf185	2004	2154	2003	2019	2068	2029	1998	1933
Rnf186	7379	6972	5886	6087	5810	5888	5401	5361
Rnf187	3229	3208	3374	2965	3263	3122	2827	2950
Rnf19a	1737	1903	1917	1804	1797	2019	2225	1893
Rnf19b	6297	6773	5886	5601	5254	5617	5558	5129
Rnf2	608	619	569	563	576	597	594	630
Rnf20	1386	1524	1415	1387	1615	1434	1389	1476
Rnf207	3	2	20	2	6	1	9	1
Rnf208	80	66	65	73	37	31	50	38
Rnf212	0	0	0	0	0	4	0	0
Rnf212b	0	0	0	0	2	0	0	0
Rnf213	26463	36110	32422	26249	27440	28335	26609	23759
Rnf214	1118	1187	1278	1146	1274	1265	1358	1121
Rnf215	526	488	448	490	538	462	463	448
Rnf216	1223	1421	1338	1265	1311	1254	1353	1199
Rnf217	159	170	161	204	165	172	156	180
Rnf220	1345	1477	1407	1391	1535	1351	1287	1340
Rnf223	4	10	9	4	10	5	9	3
Rnf225	5	9	2	7	6	9	3	2
Rnf24	440	525	489	396	473	456	530	460
Rnf25	577	620	551	487	516	634	538	546
Rnf26	892	843	843	964	917	934	864	803
Rnf31	2620	2836	2950	2250	2351	2606	2512	2401
Rnf32	61	88	100	73	117	109	115	106
Rnf34	601	609	671	639	723	637	640	670
Rnf38	1208	1451	1471	1456	1263	1366	1303	1280
Rnf39	43	43	56	68	46	44	49	69
Rnf4	3368	3524	3439	3081	3375	3417	3238	3089
Rnf40	2680	2730	2657	2731	2706	2543	2506	2387
Rnf41	700	725	692	681	698	737	758	586
Rnf43	878	912	993	957	1085	918	935	878

Transcriptome sequencing yielded total genetic results for the MOD and APS groups, with a total of 15,936 variables

Continued from above

Rnf44	5425	5675	5419	5283	5368	5138	4922	4925
Rnf5	3521	3476	3713	3481	3303	3395	3340	3224
Rnf6	1704	1803	1822	1844	1844	2049	1880	1768
Rnf7	1429	1408	1420	1444	1556	1438	1425	1378
Rnf8	467	514	522	570	582	491	485	513
Rnft1	1403	1555	1530	1465	1529	1545	1518	1493
Rnft2	27	27	31	31	1	14	11	11
Rngtt	693	762	709	686	692	723	782	710
Rnh1	4846	4950	4896	4817	5212	5257	5001	4799
Rnmt	535	624	606	516	665	606	563	565
Rnpc3	459	401	444	511	431	553	485	436
Rnpep	11528	12554	12017	10994	13276	13068	12075	12075
Rnpepl1	5128	5189	5343	5023	4895	4813	4912	4688
Rnps1	2202	2459	2295	2460	2257	2184	1949	2152
Ro60	194	188	170	206	190	245	184	228
Robo1	52	51	41	44	42	91	66	41
Robo2	68	100	53	81	67	94	66	58
Robo3	16	7	17	2	1	10	0	3
Robo4	214	233	243	172	221	209	201	165
Rock1	1808	1903	1712	1739	1955	2011	2179	1952
Rock2	3129	3640	3448	3229	3993	4390	4423	3681
Rogdi	311	259	241	269	240	276	277	243
Rom1	277	209	173	232	202	268	245	227
Romo1	1163	1255	1256	1234	1316	1347	1129	1176
Ropn1l	9	5	10	1	0	5	15	7
Ror1	35	26	12	33	28	59	36	44
Ror2	139	161	159	149	180	150	130	120
Rora	99	99	131	124	85	135	116	73
Rorb	0	0	5	1	1	0	0	1
Rorc	1441	1737	1583	1518	1833	1901	1785	1447
Ros1	306	460	358	232	561	627	382	173
Rp2	2112	2233	2139	2149	1920	2076	2179	1865
Rp9	1271	1295	1303	1270	1243	1221	1208	1251
Rpa1	1145	1310	1106	1245	1191	1307	1087	1143
Rpa2	559	540	569	544	562	459	521	503
Rpa3	569	697	645	608	661	633	554	617
Rpa4	0	0	0	0	0	0	0	1
Rpain	215	191	193	177	205	182	161	172
Rpap1	877	866	916	896	900	854	812	787
Rpap2	178	213	172	153	216	215	188	172
Rpap3	233	311	263	274	286	263	244	270
Rpe	3057	3187	3017	3146	3366	3764	3616	3382
Rpf1	631	721	679	658	726	708	662	646
Rpf2	369	474	351	331	450	412	354	363
Rpgr	162	188	190	200	213	232	252	218
Rpgrip1	7	11	19	6	17	7	9	9
Rpgrip1l	128	136	139	91	145	166	125	140
Rph3a	13	10	3	14	2	12	14	10
Rph3al	533	568	575	530	599	682	596	477
Rpia	1773	2065	1932	2036	2102	2162	1714	1839
Rpl10	19644	20977	20962	20590	21556	21044	19263	19937
Rpl10a	14427	14512	14839	14474	14988	14901	13164	14328
Rpl11	11883	12158	12283	12606	12369	12216	10635	11375
Rpl12	14384	15484	15354	15198	15895	15468	13995	14709
Rpl13	21057	22228	22466	21513	22443	21940	19534	20930
Rpl13-ps6	16	17	18	7	4	7	14	10
Rpl13a	26640	27374	27552	27509	27444	27275	24753	25828
Rpl14	8886	9312	9361	9137	9199	9401	8352	8538

Transcriptome sequencing yielded total genetic results for the MOD and APS groups, with a total of 15,936 variables

Continued from above

Rpl15	11920	13011	12723	12330	13218	13348	12024	12602
Rpl17	9468	9771	10445	10018	9966	9990	8999	9169
Rpl17-ps8	10	8	14	12	13	13	6	12
Rpl18	14938	15268	15580	14840	15417	15219	13301	14242
Rpl18a	19199	19650	20271	19876	20254	19575	17381	18028
Rpl19	15558	16300	16854	16453	16973	16533	14931	15698
Rpl21	3453	3582	3501	3586	3537	3655	3226	3500
Rpl22	6160	7018	6794	6639	6614	6709	6201	6253
Rpl22l1	2677	2855	2598	2471	2859	2982	2710	2891
Rpl23	7947	8122	8074	8330	8531	8659	7809	7927
Rpl23a	13126	13393	13886	13532	14083	13886	12599	12667
Rpl24	9259	9960	10252	10116	10033	9705	8576	9254
Rpl26	11488	11840	11974	12264	12562	12410	11041	12062
Rpl27	7413	7858	8007	7485	7874	7313	5772	6961
Rpl27a	13353	14146	14592	14168	14756	14207	12761	13455
Rpl27a-ps	21	22	19	29	25	10	12	28
Rpl28	12070	12411	12541	11849	12194	11773	10323	11103
Rpl29	9103	9401	9430	9119	9294	9227	8107	8892
Rpl3	21037	22506	22208	21421	21900	21619	18850	20308
Rpl30	8660	9228	9289	8920	9437	9997	8777	8650
Rpl31	11423	11526	11970	12066	11886	12241	10918	11566
Rpl31-ps1	0	1	2	4	3	1	1	0
Rpl32	13034	14489	14000	13903	13778	13577	11976	12751
Rpl32l	12	3	7	7	12	9	5	3
Rpl34	7775	8247	8016	8297	8260	8202	7571	7885
Rpl34-ps1	818	773	761	808	795	855	711	762
Rpl35	9721	10549	10701	10671	11054	10951	9721	10179
Rpl35a	8521	8619	8915	8853	8745	9127	8002	8199
Rpl36	7330	7525	8067	7781	7896	7385	6530	7083
Rpl36a	4637	5161	4838	4951	5240	5145	4549	4790
Rpl36al	3404	3446	3490	3480	3716	3865	3442	3676
Rpl37	5781	6003	6310	5916	6056	6038	5593	5766
Rpl37a	6694	7313	7388	7040	7489	7056	6456	6715
Rpl37rt	1005	1064	1041	1006	1035	1151	901	1027
Rpl38	4325	4745	4595	4670	4757	4671	4351	4238
Rpl39	7032	7132	7119	7244	7189	7552	6733	6971
Rpl39l	0	5	0	0	4	0	0	1
Rpl3l	11	0	0	0	0	0	2	1
Rpl4	30665	33550	33084	31945	33502	33789	30535	30838
Rpl41	22843	23118	23263	23362	24697	23988	21660	23047
Rpl5	17465	18612	18361	18122	19244	19674	17755	17753
Rpl6	12933	13626	13135	13378	13937	13067	11953	12666
Rpl6l	928	991	964	1071	1042	1013	896	910
Rpl7	21655	22888	22022	22997	24454	24693	22390	22679
Rpl7a	15557	16529	16862	16454	16269	15910	14467	15244
Rpl7l1	2758	3091	2907	2688	3166	3123	2748	2955
Rpl8	19384	19527	20764	19826	19827	19367	17148	18939
Rpl9	10079	10826	10873	10288	10656	10441	9685	9924
Rplp0	48001	50772	51080	49939	52246	50432	44872	48750
Rplp1	27974	28975	30015	29207	29368	29327	26232	27360
Rplp2	10586	10369	11070	10265	10320	10681	9293	10327
Rpn1	12444	13269	12770	11997	14710	13662	12504	12817
Rpn2	11625	12191	11583	10837	12943	12276	11471	11476
Rpp21	448	426	433	434	398	418	440	384
Rpp25	39	12	45	18	15	22	32	16
Rpp25l	398	433	395	499	430	428	355	397
Rpp30	283	254	230	266	244	278	316	281
Rpp38	113	85	75	112	150	134	92	142

Transcriptome sequencing yielded total genetic results for the MOD and APS groups, with a total of 15,936 variables

Continued from above

Rpp40	97	106	91	160	130	146	84	125
Rprd1a	275	278	347	339	272	274	324	344
Rprd1b	1460	1678	1634	1616	1546	1612	1612	1607
Rprd2	1079	1303	1162	1219	1218	1260	1225	1132
Rprm	9	0	5	2	1	1	6	7
Rprml	13	8	7	9	5	15	14	7
Rps10	10119	10300	10631	9772	9985	10284	9289	9564
Rps11	12188	12571	12692	12722	12368	11832	10936	11452
Rps12	13298	13793	13955	13939	14329	14528	12800	13621
Rps12-ps1	2	1	7	3	2	2	2	0
Rps12-ps1	0	1	0	0	0	0	0	0
Rps12-ps2	34	29	36	49	42	39	49	26
Rps13	6993	7561	7523	7659	7520	7271	6172	6724
Rps14	22539	24379	24434	24595	24321	23978	21836	23012
Rps15	12033	13023	12703	12384	12141	12385	10942	11380
Rps15a	9204	9660	9964	9746	10110	9780	9042	9172
Rps15a-ps	0	3	3	2	9	7	2	8
Rps16	14894	15435	16135	15039	15582	16046	13556	14664
Rps17	9604	9938	10340	10176	10531	10389	9592	9760
Rps18	17191	17251	17622	17221	18260	17873	15998	17077
Rps19	11676	11419	12374	11590	11588	11912	10141	10848
Rps19bp1	510	496	457	473	520	468	446	449
Rps2	31086	32865	32942	31349	33972	32585	29329	33441
Rps20	13890	14259	13960	14249	14082	13982	12529	13198
Rps21	5485	5801	5896	5587	5901	5798	5172	5538
Rps23	11206	11988	11925	11866	12279	11900	10279	11082
Rps23rg1	17	15	17	10	5	7	11	16
Rps24	13033	13973	13943	14190	14764	13952	12799	13447
Rps25	9479	10439	10804	10308	10845	10942	9609	10222
Rps26	11431	12043	11918	11908	12116	11863	11004	11582
Rps27	7308	7756	7636	7630	7569	7727	6889	6888
Rps27a	12254	11799	12754	12014	12363	12507	7882	11074
Rps27l	5223	5458	5267	5253	5708	5750	5220	5355
Rps27rt	1037	1094	1078	1180	1071	965	988	1053
Rps28	4940	5269	5414	5395	5091	5277	4717	4979
Rps29	8733	9325	9411	9386	9352	9719	8414	8876
Rps3	27478	28785	28590	27642	29102	28832	27299	27579
Rps3a1	21151	22742	21941	22129	23320	23382	21000	21614
Rps4x	19160	20009	19673	19603	20625	20250	18659	18696
Rps5	19156	19538	19543	19997	20207	19524	17231	18032
Rps6	16854	17517	17284	16713	17206	16758	15314	16270
Rps6ka1	14478	15294	15037	14018	14348	14817	14432	14256
Rps6ka2	39	66	69	37	49	79	60	76
Rps6ka3	2674	2980	2726	2690	2766	2959	2774	2619
Rps6ka4	2464	2513	2386	2534	2191	2289	2431	2428
Rps6ka5	798	805	775	740	731	763	919	662
Rps6ka6	143	160	146	103	164	150	149	136
Rps6kb1	1209	1209	1180	1192	1113	1319	1145	1260
Rps6kb2	769	808	762	710	811	768	592	762
Rps6kc1	904	1054	1005	863	997	929	918	866
Rps6kl1	12	7	6	13	10	12	7	6
Rps7	15738	16270	16269	16561	15785	16711	15189	15742
Rps8	16789	17342	17556	17480	17821	17224	15682	16057
Rps9	16985	17217	17961	17245	17865	17745	15466	16839
Rpsa	32517	33318	33998	32808	36502	34196	29571	32952
Rptor	2306	2397	2261	2231	2216	2434	2244	2133
Rpusd1	194	148	115	160	170	177	146	143
Rpusd2	173	235	164	191	177	210	193	189

Transcriptome sequencing yielded total genetic results for the MOD and APS groups, with a total of 15,936 variables

Continued from above

Rpusd3	135	167	175	133	117	128	157	118
Rpusd4	414	464	467	406	522	455	400	428
Rrad	29	28	21	45	24	32	15	27
Rraga	1340	1425	1361	1503	1444	1578	1424	1456
Rragb	2	4	2	8	8	2	13	11
Rragc	2241	2320	2344	2366	2193	2306	2142	2148
Rragd	4579	5167	5292	5036	5014	4999	5335	4688
Rras	618	637	605	682	665	673	632	623
Rras2	1542	1722	1848	1765	1861	1780	1743	1705
Rrbp1	26059	28299	25823	24906	26865	28372	26307	25267
Rreb1	3502	3732	3749	3623	3404	3574	3440	3329
Rrh	0	1	0	0	0	0	0	0
Rrm1	2138	2497	2391	2203	2408	2432	2016	2301
Rrm2	891	1061	1003	932	1192	1017	863	981
Rrm2b	186	173	177	226	172	202	180	214
Rrn3	979	1161	1183	1176	1313	1242	1226	1188
Rrnad1	310	210	311	344	286	320	335	311
Rrp1	3261	3199	3347	3392	3349	3421	3145	3252
Rrp12	724	731	774	631	913	810	672	730
Rrp15	352	391	338	302	336	356	223	357
Rrp1b	409	452	432	328	459	445	425	409
Rrp36	1034	1114	1031	1020	1115	1139	981	929
Rrp7a	1860	2123	2043	1781	2278	2198	1868	2046
Rrp8	311	345	357	373	443	408	346	356
Rrp9	558	532	504	520	530	538	489	606
Rrs1	715	749	698	705	836	645	642	748
Rs1	1	0	1	0	0	0	1	0
Rsad1	737	435	560	773	308	346	393	407
Rsad2	1093	2230	1912	830	1351	1374	1288	1232
Rsbn1	555	658	590	718	794	693	561	662
Rsbn1l	649	859	812	783	823	792	868	738
Rsc1a1	4	1	1	0	0	0	1	0
Rsf1	876	1036	883	925	919	1014	1032	945
Rskr	9	11	2	17	19	22	5	12
Rsl1	81	107	98	134	136	116	106	119
Rsl1d1	1402	1692	1444	1481	1871	1757	1563	1541
Rsl24d1	699	758	771	828	953	766	847	759
Rslcan18	17	9	19	16	14	16	30	11
Rsph1	10	0	8	1	17	8	11	16
Rsph3a	943	769	677	589	647	815	636	729
Rsph3b	333	383	372	401	372	317	371	384
Rsph4a	4	4	1	1	6	6	8	16
Rsph6a	0	0	0	1	0	0	0	0
Rsph9	20	13	25	11	19	10	6	13
Rspo1	23	12	13	19	22	24	36	16
Rspo2	6	12	7	5	9	7	6	4
Rspo3	58	24	29	54	52	45	26	44
Rspo4	1	0	5	5	2	4	3	0
Rspry1	1074	1058	1033	1037	1241	1167	1206	1141
Rsrc1	566	657	595	688	517	561	534	526
Rsrc2	1264	1331	1273	1300	1333	1462	1444	1251
Rsrp1	6151	6534	7331	7005	6489	6764	6145	6336
Rsu1	329	364	369	347	369	369	379	403
Rtbdn	0	0	0	0	1	0	4	0
Rtca	1378	1337	1264	1557	1176	1383	1212	1272
Rtcb	3910	4362	3979	4058	4111	4226	3873	3906
Rtel1	405	419	432	380	467	451	400	487
Rtf1	1281	1429	1315	1227	1253	1274	1328	1155

Transcriptome sequencing yielded total genetic results for the MOD and APS groups, with a total of 15,936 variables

Continued from above

Rtf2	2973	3107	2830	2745	2824	2918	2708	2726
Rtkn	1210	1295	1371	1356	1317	1164	1264	1113
Rtkn2	1	4	12	6	0	6	0	5
Rtl1	0	0	0	0	0	1	0	4
Rtl3	5	4	11	1	7	1	3	7
Rtl4	0	0	3	0	0	0	0	1
Rtl5	24	32	13	25	33	26	9	15
Rtl6	33	71	35	38	30	56	51	50
Rtl8a	63	76	52	65	63	73	77	56
Rtl8b	68	96	91	71	45	63	71	56
Rtl8c	49	72	67	52	40	50	58	46
Rtl9	0	0	1	0	6	0	0	6
Rtn1	110	114	96	94	119	118	108	78
Rtn2	55	50	44	77	28	49	64	53
Rtn3	8753	9150	8813	9069	9636	9365	9084	8893
Rtn4	2071	1984	2079	1972	1922	1961	1899	1833
Rtn4ip1	865	1090	965	896	929	933	907	915
Rtn4r	541	397	531	518	383	438	512	454
Rtn4rl1	1202	1521	1361	1289	1216	1170	1212	1090
Rtn4rl2	192	139	151	264	64	42	63	93
Rtp3	3	17	3	9	3	6	6	6
Rtp4	2625	3640	3139	2491	2735	2875	2593	2438
Rtraf	3129	3516	3468	3541	3513	3568	3224	3321
Rttn	147	173	166	209	169	160	125	90
Rubcn	1931	2021	1936	1826	2025	2014	2005	1855
Rubcnl	7	8	37	9	4	14	7	19
Rufy1	1021	1038	1152	1053	1017	1085	1007	966
Rufy2	228	262	290	260	176	226	225	232
Rufy3	1163	1158	1137	1187	1163	1312	1273	1171
Rufy4	0	0	9	1	5	0	0	0
Rundc1	1313	1370	1325	1303	1415	1444	1342	1329
Rundc3a	83	80	106	103	109	91	71	73
Rundc3b	311	349	361	323	351	429	358	393
Runx1	217	239	243	244	294	227	217	208
Runx1t1	20	44	28	27	21	29	12	20
Runx2	107	84	58	70	84	61	89	56
Runx3	83	94	90	96	94	106	81	88
Rusc1	1399	1267	1217	1234	1357	1289	1175	1305
Rusc2	164	237	196	205	169	164	181	146
Rusf1	1625	1781	1676	1692	1658	1675	1499	1567
Ruvbl1	710	666	664	515	817	755	605	721
Ruvbl2	1271	1481	1412	1363	1476	1430	1239	1304
Rwdd1	675	728	776	778	824	761	759	722
Rwdd2a	20	26	15	13	21	24	6	5
Rwdd2b	2415	2739	2333	2436	2561	2703	2785	2526
Rwdd3	9	13	6	16	12	15	5	14
Rwdd4a	653	686	677	697	714	722	573	662
Rxfp2	0	0	6	0	0	0	0	0
Rxfp3	0	0	0	0	0	0	1	0
Rxra	6441	6848	6400	6296	6504	7011	6678	6286
Rxrb	1583	1659	1534	1562	1875	1978	1652	1627
Rxrg	0	10	1	0	1	1	1	5
Rxylt1	521	630	554	537	631	531	466	534
Rybp	1097	1202	1063	1112	1289	1384	1453	1219
Rybp-ps	10	9	10	5	7	7	6	5
Ryk	1632	1789	1668	1650	1656	1612	1744	1498
Ryr1	5	6	13	12	8	13	15	11
Ryr2	19	41	41	50	68	39	43	30

Transcriptome sequencing yielded total genetic results for the MOD and APS groups, with a total of 15,936 variables

Continued from above

Ryr3	20	13	25	11	10	22	14	9
S100a1	1157	1029	1077	1232	978	984	1013	1108
S100a10	12268	12886	12531	12415	12859	13558	13218	13111
S100a11	1900	1990	2140	2232	2135	2241	1961	2241
S100a13	273	317	348	354	353	389	339	339
S100a14	188	107	151	166	151	121	128	132
S100a16	1326	1250	1289	1299	1414	1235	1075	1239
S100a3	0	4	0	0	1	0	0	0
S100a4	8	23	22	20	21	17	30	27
S100a5	0	1	0	0	1	0	0	0
S100a6	1418	1392	1502	1535	1599	1662	1496	1602
S100a7a	1	1	0	0	0	0	4	0
S100a8	1	1	1	4	0	0	0	0
S100a9	0	1	1	4	0	1	0	1
S100b	14	16	14	26	12	9	7	17
S100g	1025	1264	878	683	1418	1486	548	556
S100pbp	250	286	333	361	285	308	313	276
S100z	1	11	0	6	2	6	1	1
S1pr1	123	174	241	224	204	193	163	149
S1pr2	119	126	132	118	127	97	121	94
S1pr3	147	128	144	153	145	121	113	143
S1pr4	14	8	19	19	15	23	15	1
S1pr5	2	2	1	1	2	5	1	1
Saa1	77	72	31	54	47	98	80	68
Saa2	0	5	0	0	0	0	1	0
Saa3	181	244	239	208	144	108	149	184
Saa4	1	0	1	1	2	2	3	2
Saal1	351	266	299	311	309	350	314	363
Sac3d1	377	348	383	360	432	425	361	347
Sacm1l	3570	3607	3553	3579	4058	4313	4081	3779
Sacs	34	50	51	43	33	44	42	43
Sae1	2742	3457	2903	2920	3314	3165	3005	3058
Safb	1878	2311	2317	2415	2675	2672	2249	2328
Safb2	1517	1748	1636	1647	1695	1723	1595	1507
Sag	9	1	3	1	3	5	0	5
Sall1	19	44	23	16	20	25	9	14
Sall2	34	45	20	59	34	27	59	36
Sall3	0	0	0	2	0	0	0	1
Sall4	1	4	2	1	0	0	1	0
Samd1	432	481	530	467	532	510	405	454
Samd10	396	469	477	483	481	421	430	417
Samd11	17	23	37	37	10	25	15	26
Samd12	25	31	30	21	26	51	24	21
Samd14	72	100	102	102	90	93	70	63
Samd15	1	1	5	1	4	2	0	0
Samd3	2	1	2	0	1	0	0	4
Samd4	73	92	96	84	62	72	127	87
Samd4b	2635	2668	2589	2304	2676	2442	2393	2293
Samd5	11	16	6	9	9	28	17	22
Samd8	3469	3833	3730	3672	3935	4114	4063	3788
Samd9l	4523	5336	4532	3941	5159	5275	4973	4249
Samhd1	9395	10162	8549	8190	7340	7919	7158	6518
Samm50	6257	6399	6404	6704	5993	6137	5302	5704
Samsn1	34	54	36	38	31	26	37	23
Sap130	1397	1438	1480	1534	1400	1422	1376	1297
Sap18	2879	3077	2857	2647	2765	3027	2557	2849
Sap18b	712	855	691	774	663	774	754	706
Sap25	135	134	191	158	109	156	126	113

Transcriptome sequencing yielded total genetic results for the MOD and APS groups, with a total of 15,936 variables

Continued from above

Sap30	472	493	645	447	522	536	525	424
Sap30bp	1094	1113	973	1077	1022	1133	1086	1016
Sap30l	2445	2197	2285	2565	2010	2015	2026	1931
Sapcd1	1	0	1	2	0	1	0	2
Sapcd2	375	434	392	381	469	434	363	356
Sar1a	3539	3667	3568	3582	3900	4033	3655	3591
Sar1b	9291	10485	9831	8705	11279	11625	11527	11314
Saraf	6564	6836	6363	6099	6307	6866	6404	5998
Sardh	361	407	344	349	413	444	430	340
Sarm1	14	1	6	11	4	8	3	1
Sarnp	488	401	461	409	388	663	481	418
Sars	3953	4157	3982	4022	3737	3894	3641	3771
Sars2	528	496	571	500	514	410	463	451
Sart1	2838	2682	2631	2586	2837	2871	2608	2498
Sart3	1576	1537	1391	1553	1682	1537	1362	1443
Sash1	1505	1578	1474	1512	1434	1331	1383	1319
Sash3	217	162	149	156	134	202	156	134
Sass6	647	722	628	591	678	712	685	620
Sat1	4291	4451	4463	4272	4134	4365	3940	4126
Sat2	71	70	63	43	64	61	93	66
Satb1	17	25	50	43	30	47	35	26
Satb2	70	97	71	84	72	54	58	77
Satl1	1	1	0	0	1	1	2	0
Sav1	1212	1295	1316	1255	1245	1215	1096	1217
Saxo1	2	0	0	1	0	4	0	0
Saxo2	6	9	7	9	10	7	13	5
Saysd1	269	232	235	271	285	282	257	288
Sbds	3253	3332	2986	3205	3432	3561	3395	3176
Sbf1	3677	3883	3560	3683	3799	3761	3368	3443
Sbf2	2606	2816	2581	2649	2570	2682	2798	2399
Sbk1	1166	1154	1106	1176	1461	1653	1661	1489
Sbk2	0	0	0	0	0	0	0	1
Sbk3	0	0	1	0	0	6	0	1
Sbno1	3193	3666	3491	3742	3694	3976	3925	3600
Sbno2	5463	5677	5514	5592	5077	5213	5285	5000
Sbsn	62	73	76	91	70	69	62	66
Sbspon	11	18	16	6	6	12	1	2
Sc5d	4473	4548	4449	4725	4722	5176	4926	4614
Scaf1	3809	3843	3631	3660	3418	3442	3272	3100
Scaf11	3184	3229	3034	3177	3309	3412	3220	2982
Scaf4	2087	2247	2416	2197	2143	2286	1906	1915
Scaf8	1313	1409	1374	1232	1428	1429	1375	1233
Scai	126	120	157	180	166	136	166	117
Scamp1	2534	2887	2714	2495	2533	2484	2646	2379
Scamp2	7193	7264	6809	7023	6980	6758	6731	6534
Scamp3	16	27	36	16	17	24	25	25
Scamp4	2972	2824	2783	2623	2820	2750	2519	2647
Scamp5	7980	8497	7621	6860	8110	8425	7832	7435
Scand1	3009	2712	2836	2782	2498	2375	2374	2426
Scap	1999	1998	2126	1914	2197	1953	1840	1875
Scaper	236	217	231	271	209	254	224	214
Scara3	46	29	50	68	44	58	23	44
Scara5	22	29	30	34	38	37	51	40
Scarb1	1197	1313	1228	1004	1288	1189	1125	1146
Scarb2	3077	3550	3334	3283	3652	3577	3044	3264
Scarf1	151	189	180	156	198	189	163	166
Scarf2	192	193	262	254	225	208	214	241
Sccpdh	1735	2100	1687	1611	2017	1910	1947	1903

Transcriptome sequencing yielded total genetic results for the MOD and APS groups, with a total of 15,936 variables

Continued from above

Scd1	441	420	460	415	1083	1036	1027	384
Scd2	7922	9642	12285	10717	8756	8786	7150	7967
Scd4	2	1	2	2	3	2	1	1
Scel	4	0	8	0	2	0	0	2
Scfd1	2837	3204	2748	2618	2990	3213	3338	2713
Scfd2	499	492	513	461	557	526	491	556
Scg2	471	435	494	459	418	468	437	402
Scg3	88	103	89	74	87	102	55	94
Scg5	37	63	91	52	27	72	68	46
Scgb2b10	1	0	1	0	0	0	0	0
Scgb2b15	0	0	0	0	0	0	0	4
Scgb3a1	11	10	3	12	4	8	8	22
Scgn	103	147	119	105	98	75	97	111
Schip1	6	6	4	1	6	0	5	4
Scimp	25	52	43	36	43	34	44	30
Scin	4265	4498	4217	4535	3780	3612	3830	3662
Sclt1	152	212	216	212	180	203	224	208
Scly	2067	2074	1987	1920	1910	2131	1852	1761
Scmh1	340	478	500	390	385	361	283	338
Scml1	0	0	0	0	0	4	0	0
Scml2	0	0	2	3	2	1	0	1
Scml4	1181	1336	1423	1332	1376	1312	1218	1210
Scn10a	0	0	0	0	0	0	0	4
Scn11a	5	25	19	12	4	20	10	20
Scn1b	165	160	107	158	123	125	125	128
Scn2a	1	1	12	9	2	4	1	3
Scn2b	210	268	194	248	205	187	198	169
Scn3a	59	59	55	59	56	62	57	38
Scn3b	12	10	4	4	16	4	4	6
Scn4b	4	2	0	2	14	3	1	10
Scn5a	10	17	19	20	23	21	14	12
Scn7a	120	230	121	134	183	206	156	145
Scn8a	81	107	82	103	111	64	144	102
Scn9a	6	12	17	12	4	12	14	10
Scnm1	698	720	604	678	748	770	670	602
Scnn1a	426	433	443	504	388	379	391	423
Scnn1b	0	0	0	6	0	0	0	0
Sco1	2160	2583	2354	2067	2400	2441	2234	2235
Scoc	1754	1938	1776	1666	1865	2194	2273	1844
Scp2	19208	20500	18676	18494	18970	20496	21860	19729
Scpep1	1514	1626	1612	1819	1540	1614	1343	1485
Scrg1	1	1	0	0	1	0	0	1
Scrib	2323	2405	2393	2343	2564	2426	2179	2153
Scrn1	19	19	25	18	15	30	23	30
Scrn2	1530	1678	1756	1479	1637	1754	1385	1551
Scrn3	256	215	251	193	314	244	247	274
Scrt1	0	2	0	3	2	0	7	2
Scrt2	0	0	1	0	0	1	0	0
Sct	1208	1235	1400	1365	1122	1131	1103	1182
Sctr	10	14	7	9	4	3	3	7
Scube1	625	632	610	642	594	504	552	485
Scube2	37	30	13	37	26	32	11	4
Scube3	10	32	15	13	8	18	5	6
Scx	3	3	4	2	12	6	0	4
Scyl1	1817	2007	1792	1843	1809	1859	1746	1742
Scyl2	2848	3031	2700	2906	3108	3043	3114	2809
Scyl3	1337	1690	1443	1406	1421	1551	1490	1345
Sdad1	1373	1345	1374	1547	1304	1376	1211	1212

Transcriptome sequencing yielded total genetic results for the MOD and APS groups, with a total of 15,936 variables

Continued from above

Sdc1	3626	3900	3534	3231	3730	3455	3260	3246
Sdc2	359	442	374	384	394	415	310	357
Sdc3	747	817	792	769	586	571	509	434
Sdc4	2893	3251	3112	3218	3065	3235	2638	2831
Sdcbp	14930	15896	14159	14871	16038	16609	17155	15535
Sdcbp2	4561	4833	4211	3965	5301	4981	4945	4577
Sdccag8	207	182	205	231	250	213	173	163
Sde2	807	924	858	868	863	919	964	786
Sdf2	1715	1848	1928	1784	1961	2015	1823	1880
Sdf2l1	1919	2061	2182	1889	2559	2635	2202	2355
Sdf4	9118	9748	9052	8950	9284	9394	9338	8527
Sdha	29078	31258	29518	29889	29135	30085	29151	27751
Sdhaf1	799	856	820	849	745	712	743	738
Sdhaf2	1380	1604	1421	1511	1574	1626	1409	1368
Sdhaf3	124	150	176	167	188	214	181	174
Sdhaf4	337	398	343	324	356	394	367	377
Sdhb	16090	16052	16137	16437	14787	15787	15385	14497
Sdhc	13896	14201	13950	14227	13385	13261	13275	13357
Sdhd	19080	18966	18458	19102	18512	18732	18630	18498
Sdk1	51	42	40	45	34	20	38	28
Sdk2	43	58	92	38	54	60	44	39
Sdr16c5	0	0	0	0	0	4	0	1
Sdr16c6	4	0	0	0	0	1	1	0
Sdr39u1	1291	1240	1231	1194	1080	1064	1130	1079
Sdr42e1	394	348	321	373	359	382	381	366
Sdr9c7	10	3	17	4	12	11	5	29
Sds	3	2	0	1	4	6	2	1
Sdsl	1066	1236	1260	1106	1198	1211	1077	1091
Sec1	0	0	2	1	0	4	0	4
Sec11a	2976	2872	2932	2851	3140	3178	2848	2894
Sec11c	1933	2050	2047	1919	2205	2224	2048	2076
Sec13	11335	12026	11016	10676	11229	11487	10910	10907
Sec14l1	3658	3998	3750	3451	4487	4529	4478	4094
Sec14l2	8188	9104	7937	7187	8715	9435	9513	8248
Sec14l4	3	6	2	0	4	6	3	2
Sec14l5	0	1	0	0	0	0	0	0
Sec16a	5670	6342	5611	5629	6308	6655	6288	5999
Sec16b	4908	4775	4622	4758	5244	5464	5430	4829
Sec22a	1222	1382	1165	1258	1134	1136	1128	1142
Sec22b	3797	4164	3713	3627	4294	4278	4404	4161
Sec22c	2769	2617	2540	2522	2845	3052	3098	2838
Sec23a	13261	14942	13259	12931	14863	15347	15578	13511
Sec23b	5268	5132	5023	5200	5316	5055	4771	4765
Sec23ip	2266	2390	2126	2210	2391	2543	2263	2435
Sec24a	4905	5477	4873	5034	5556	6039	6014	5514
Sec24b	1755	2045	1857	1783	1776	2003	1788	1811
Sec24c	8645	9446	8597	8498	9415	9448	9580	8651
Sec24d	12585	14099	11664	11423	13276	13700	14780	13270
Sec31a	19285	21000	19438	19133	19611	20145	19946	18527
Sec31b	16	12	10	19	12	23	17	18
Sec61a1	13641	14452	13184	13066	16153	15493	13956	14783
Sec61a2	482	496	526	518	529	498	517	537
Sec61b	3692	3798	3871	3842	4172	4145	3872	3848
Sec61g	2291	2373	2410	2485	2799	2583	2487	2464
Sec62	1776	1911	1708	1630	1999	2013	1947	1911
Sec63	3478	3541	3561	3289	3728	3905	3773	3272
Secisbp2	718	755	699	657	833	782	717	693
Secisbp2l	4934	4890	4592	4915	4019	4594	4495	4057

Transcriptome sequencing yielded total genetic results for the MOD and APS groups, with a total of 15,936 variables

Continued from above

Sectm1a	192	164	187	147	169	168	171	154
Sectm1b	18746	21102	17429	15880	18753	21306	21481	18436
Seh1l	1793	2090	2128	2004	2068	2036	1943	1981
Sel1l	5280	5679	5253	5238	6328	6152	5952	5473
Sel1l3	4562	5116	4864	4435	5082	5156	5293	4653
Sele	6	5	5	2	4	3	6	5
Selenbp1	1849	1998	1911	1841	1955	2094	1913	1856
Selenbp2	28	28	53	38	48	45	29	48
Selenof	9482	9912	9764	9554	10465	10841	10344	10311
Selenoh	329	373	350	305	451	345	346	406
Selenoi	5466	6174	5601	4918	6477	6603	6611	6615
Selenok	1748	1810	1722	1786	1929	2029	1995	1857
Selenom	920	938	974	945	926	958	921	1020
Selenon	200	230	233	228	189	179	188	173
Selenoo	1072	1092	1036	1092	1054	952	869	921
Selenop	61212	62433	56855	61164	49931	55526	62261	51117
Selenos	2524	2693	2534	2463	2756	2845	2691	2737
Selenot	7564	8537	7849	8080	8494	8957	8891	8364
Selenov	0	1	0	0	0	1	0	1
Selenow	2958	3092	2961	2786	3185	3220	3095	3092
Sell	3	0	1	4	1	0	5	0
Selp	51	52	70	33	30	71	44	50
Selplg	260	303	272	292	194	261	204	207
Sem1	2109	2299	2216	2203	2333	2458	2210	2146
Sema3a	8	28	32	14	36	31	29	28
Sema3b	2114	2130	2025	2051	2316	2145	2206	2007
Sema3c	202	230	208	238	257	214	236	203
Sema3d	19	24	27	33	20	30	24	35
Sema3e	18	8	3	25	18	12	14	7
Sema3f	470	393	425	364	390	306	325	439
Sema3g	78	138	118	134	100	109	120	94
Sema4a	9605	10436	9394	9016	9332	9227	9399	8922
Sema4b	3460	3388	3257	3294	3249	3311	3434	2989
Sema4c	226	192	199	201	160	192	165	158
Sema4d	521	542	459	459	424	449	343	363
Sema4f	8	1	0	20	5	10	10	6
Sema4g	8232	8761	9205	8918	8300	8372	7765	7337
Sema5a	795	862	896	872	763	769	864	668
Sema5b	6	18	6	9	7	6	10	7
Sema6a	3814	4494	3477	3315	3934	4161	4706	4199
Sema6b	297	296	310	266	373	417	376	411
Sema6c	23	16	15	31	10	19	23	12
Sema6d	2654	3094	2309	2069	3009	3064	2594	2659
Sema7a	691	661	689	745	632	648	711	634
Senp1	1049	1128	1104	1159	1061	1010	1017	944
Senp2	2166	2150	2144	1990	2263	2290	2347	2041
Senp3	2233	2399	2327	2171	2360	2372	2126	2149
Senp5	1199	1339	1333	1240	1432	1351	1283	1375
Senp6	2289	2668	2308	2453	2662	2715	2530	2301
Senp7	612	751	594	598	695	596	652	619
Senp8	121	124	115	185	164	183	140	151
Sephs1	1345	1520	1473	1300	1326	1343	1360	1316
Sephs2	13129	14102	13184	12470	13089	13351	12902	12093
Sepsecs	1746	2121	1857	1793	1802	1893	2022	1776
Septin1	147	116	106	148	176	114	123	108
Septin10	199	248	251	266	214	213	214	236
Septin11	1494	1607	1470	1485	1663	1562	1401	1454
Septin12	1	1	0	0	0	1	0	0

Transcriptome sequencing yielded total genetic results for the MOD and APS groups, with a total of 15,936 variables

Continued from above

Septin2	3808	4237	3832	4239	4528	4472	4406	4041
Septin3	5	7	5	9	6	6	2	4
Septin4	542	425	499	536	409	408	452	364
Septin5	10506	10818	10172	10404	9767	9485	9591	10147
Septin6	195	219	201	200	240	222	250	170
Septin7	3182	3257	2940	2938	3377	3746	3697	3251
Septin8	745	725	749	757	666	717	595	652
Septin9	4136	4127	3800	3706	4151	3967	3773	3794
Serac1	249	177	217	242	229	187	193	192
Serbp1	9022	10092	8879	9081	10477	10297	9375	9290
Serf1	109	140	122	128	113	143	93	108
Serf2	9967	9272	9639	9916	9279	9280	9272	9055
Sergef	144	165	158	139	148	176	169	80
Serhl	2847	3087	2891	3092	2939	2613	2585	2556
Serinc1	3449	3651	3136	3336	3582	3801	3727	3425
Serinc2	6118	6238	5822	5801	6384	6027	5512	5913
Serinc3	28347	32645	30835	29757	32276	33732	34180	31830
Serinc4	67	56	49	58	23	79	34	39
Serinc5	1335	1557	1499	1467	1275	1512	1285	1301
Serp1	14860	15864	14596	14540	17966	17909	16724	16213
Serp2	21	13	22	23	6	13	8	6
Serpina10	1	16	4	1	31	8	9	15
Serpina11	1	3	1	1	2	1	2	1
Serpina12	0	0	0	1	4	1	0	1
Serpina1a	14	32	8	13	11	15	12	8
Serpina1b	67	123	54	54	66	80	104	110
Serpina1c	28	29	18	34	61	33	50	41
Serpina1d	14	10	11	14	42	21	13	11
Serpina1e	33	39	18	47	159	12	18	76
Serpina1f	0	0	0	0	0	1	0	0
Serpina3a	0	0	2	1	0	4	0	1
Serpina3c	0	0	1	5	1	1	6	7
Serpina3f	429	485	457	483	237	247	241	167
Serpina3g	867	869	872	821	485	484	591	498
Serpina3i	12	21	8	7	5	1	3	7
Serpina3k	12	22	17	8	101	26	39	54
Serpina3m	1	3	4	1	3	2	5	2
Serpina3n	185	238	222	239	176	250	238	301
Serpina6	0	19	0	0	0	2	1	0
Serpina7	3	0	5	4	0	1	1	0
Serpina9	0	4	5	2	6	0	1	1
Serpinb10	0	0	0	0	1	0	0	0
Serpinb12	0	1	0	1	0	0	0	1
Serpinb1a	25869	27288	24827	25047	26639	27840	27934	27078
Serpinb1b	0	2	5	0	1	5	2	1
Serpinb1c	1	0	2	0	0	0	1	2
Serpinb2	1	1	11	2	4	10	2	8
Serpinb5	61	65	46	94	82	74	98	107
Serpinb6a	14076	15006	13714	13461	16135	16420	16649	15720
Serpinb6b	47	84	101	96	79	99	73	75
Serpinb6c	1	0	0	0	0	2	0	5
Serpinb7	0	1	0	1	0	1	1	0
Serpinb8	36	30	40	23	48	25	22	20
Serpinb9	320	230	242	271	236	255	180	233
Serpinb9b	0	0	0	2	5	0	0	0
Serpinc1	25	35	39	45	30	40	56	14
Serpind1	1	10	1	6	5	4	4	3
Serpine1	50	27	39	37	22	14	23	38

Transcriptome sequencing yielded total genetic results for the MOD and APS groups, with a total of 15,936 variables

Continued from above

Serpine2	87	120	155	93	124	132	117	116
Serpine3	0	0	0	0	4	0	0	0
Serpinf1	63	73	75	96	87	73	57	87
Serpinf2	126	139	111	106	91	123	80	107
Serping1	2812	2655	2649	2671	2186	2011	1861	2031
Serpinh1	1600	2016	2209	1837	2197	2171	2016	1987
Serpini1	44	55	64	54	40	53	56	31
Serpini2	53	77	18	5	287	270	175	204
Sertad1	1111	1126	1314	1225	1211	1242	1131	1061
Sertad2	1983	2284	2351	2078	2266	2243	2181	2004
Sertad3	603	627	582	538	575	597	592	494
Sertad4	37	39	37	32	44	36	28	24
Sertm1	132	74	104	81	108	75	102	71
Sesn1	2255	2624	2223	1998	2829	3134	2842	2491
Sesn2	315	391	363	326	463	368	384	384
Sesn3	119	117	142	143	137	138	112	117
Sestd1	1344	1575	1459	1577	1503	1554	1493	1292
Set	4684	5234	4922	4381	5426	5042	4475	4433
Setbp1	164	187	182	169	180	206	185	170
Setd1a	1183	1297	1172	1201	1182	1110	1053	1083
Setd1b	1497	1595	1666	1508	1734	1427	1433	1497
Setd2	1924	1943	1922	1942	1739	2014	1986	1805
Setd3	4649	4888	4763	4769	4674	4507	4579	4310
Setd4	87	103	68	56	90	76	59	65
Setd5	2851	3215	3269	3244	2956	2956	2854	2723
Setd6	276	274	251	192	234	234	233	248
Setd7	905	998	1006	973	846	930	845	889
Setdb1	950	1040	1127	1117	1212	1225	1011	947
Setdb2	24	14	5	4	10	19	8	15
Setmar	63	73	46	95	87	87	75	59
Setx	1879	1977	1848	1871	1767	1925	1933	1710
Sez6	12	2	19	6	11	2	9	5
Sez6l	17	17	3	13	8	12	9	7
Sez6l2	61	70	80	38	60	80	38	37
Sf1	5101	5132	5149	4964	5076	5147	4729	4903
Sf3a1	2707	2826	2847	2582	2830	2662	2633	2592
Sf3a2	1287	1314	1326	1315	1264	1252	1184	1177
Sf3a3	1131	1228	1172	1109	1220	1168	1139	1075
Sf3b1	7975	8652	8131	8601	8582	8500	8095	7722
Sf3b2	5875	6326	5671	5598	6221	5909	5932	5937
Sf3b3	3110	3699	3224	3202	3654	3540	2978	3269
Sf3b4	1665	1669	1787	1647	1755	1479	1557	1592
Sf3b5	1561	1642	1731	1671	1708	1600	1414	1709
Sf3b6	1943	2119	1899	1916	1966	1909	1882	1809
Sfi1	313	324	360	330	323	326	302	305
Sfmbt1	320	331	360	377	277	324	253	259
Sfmbt2	3	4	0	1	0	1	0	1
Sfn	6265	6028	5910	5862	7532	7737	7086	6925
Sfpq	5187	5582	5502	5412	6273	6007	5647	5281
Sfr1	947	1073	1003	938	1026	1036	1062	1013
Sfrp1	1018	1162	1136	1070	1093	1012	948	997
Sfrp2	0	7	1	2	0	2	8	1
Sfrp4	0	0	0	0	1	0	0	0
Sfrp5	17	28	13	35	19	14	15	18
Sfswap	1506	1464	1487	1475	1481	1464	1373	1307
Sft2d1	1955	1912	1774	1736	1713	1698	1728	1687
Sft2d2	11076	11624	11388	11759	10779	11379	11176	10492
Sft2d3	216	251	278	189	244	180	250	222

Transcriptome sequencing yielded total genetic results for the MOD and APS groups, with a total of 15,936 variables

Continued from above

Sftpb	2	9	3	3	10	1	0	4
Sftpc	0	1	0	0	0	0	0	0
Sftpd	4	0	0	0	0	0	0	0
Sfxn1	6747	7546	7060	6704	7879	7931	8012	7954
Sfxn2	212	207	154	169	242	190	182	245
Sfxn3	197	233	265	229	208	192	169	217
Sfxn4	17	0	4	4	18	8	5	6
Sfxn5	34	38	26	34	35	20	18	26
Sgca	0	3	3	0	1	0	1	0
Sgcb	252	261	257	270	227	179	253	223
Sgcd	89	156	176	167	181	164	176	176
Sgce	86	135	107	118	148	120	128	119
Sgcg	2	5	2	1	5	0	0	0
Sgcz	1	0	0	0	0	0	0	0
Sgf29	252	220	263	211	232	253	201	218
Sgip1	4	15	16	7	20	11	9	19
Sgk1	2105	3420	4158	2770	3240	3864	4028	4084
Sgk2	1022	908	791	881	984	946	1130	950
Sgk3	204	209	194	231	225	247	290	250
Sgms1	897	936	817	877	852	847	975	958
Sgms2	2940	3129	3106	3018	2726	2879	2936	2697
Sgo1	246	320	257	297	356	322	298	255
Sgo2a	133	129	175	159	222	194	181	147
Sgpl1	15598	18303	15662	14352	15830	17298	17640	15405
Sgpp1	2086	2220	2205	2037	2261	2249	2096	2042
Sgpp2	6832	7288	6839	7165	7741	7126	7072	6653
Sgsh	347	351	420	391	366	354	348	362
Sgsm1	328	237	305	278	314	280	273	242
Sgsm2	364	415	453	482	346	350	313	358
Sgsm3	672	697	741	636	835	694	692	723
Sgta	3896	3776	3866	3797	3862	3592	3722	3660
Sgtb	31	51	34	29	43	28	25	29
Sh2b1	1996	1898	2224	2155	2045	2122	1899	1808
Sh2b2	38	43	40	43	12	31	22	41
Sh2b3	1974	2201	2002	1947	1851	1899	1924	1907
Sh2d1a	1	0	2	6	6	5	0	1
Sh2d1b1	40	32	24	25	16	25	22	22
Sh2d2a	26	43	58	49	34	31	32	32
Sh2d3c	146	154	153	179	135	122	112	153
Sh2d4a	1118	1173	1362	1362	1473	1318	1290	1281
Sh2d4b	38	22	31	36	30	29	34	20
Sh2d5	10	13	12	17	5	12	6	7
Sh2d6	34	47	13	27	42	39	31	31
Sh2d7	33	32	41	50	27	71	31	39
Sh3bgr	83	98	129	113	89	123	117	91
Sh3bgrl	4554	4773	4300	4634	4765	5024	5193	4626
Sh3bgrl2	1858	2237	2214	2236	2038	2083	1915	2060
Sh3bgrl3	1858	1936	2156	2076	1956	2093	1983	1954
Sh3bp1	431	363	371	414	315	316	297	334
Sh3bp2	481	504	442	502	441	457	397	462
Sh3bp4	503	603	611	547	662	523	634	575
Sh3bp5	164	253	258	247	244	182	171	224
Sh3bp5l	1650	1716	1616	1674	1750	1629	1379	1335
Sh3d19	6940	7709	7254	6851	7502	7922	7886	7337
Sh3d21	860	824	943	970	892	833	781	694
Sh3gl1	2175	2168	2241	2239	2218	2413	2219	2218
Sh3gl2	1032	1087	994	1078	1032	985	1041	1001
Sh3gl3	24	26	40	17	16	21	12	28

Transcriptome sequencing yielded total genetic results for the MOD and APS groups, with a total of 15,936 variables

Continued from above

Sh3glb1	4875	5067	5210	4877	5143	5583	5195	4932
Sh3glb2	1919	1847	2073	2280	1695	1664	1645	1547
Sh3kbp1	120	138	120	141	206	191	135	106
Sh3pxd2a	594	607	631	700	731	566	528	524
Sh3pxd2b	280	321	250	309	242	232	278	224
Sh3rf1	1347	1401	1350	1249	1439	1582	1345	1416
Sh3rf2	245	287	331	417	199	240	204	216
Sh3rf3	3	1	0	0	1	4	0	1
Sh3tc1	710	648	731	804	659	649	651	533
Sh3tc2	4	22	11	8	14	14	5	17
Sh3yl1	1109	1054	1095	1126	1041	1076	995	849
Shank1	9	5	12	3	11	7	18	6
Shank2	391	515	496	476	414	483	351	440
Shank3	200	236	232	248	284	228	219	206
Sharpin	1695	1625	1576	1645	1534	1601	1439	1432
Shb	1658	1574	1455	1406	1647	1542	1603	1488
Shbg	0	0	0	0	1	0	0	0
Shc1	3240	3614	3424	3411	3440	3484	3239	3281
Shc2	60	52	53	21	38	43	30	38
Shc3	1	2	2	10	0	2	9	1
Shc4	15	14	14	21	19	4	8	17
Shcbp1	253	323	340	287	362	387	336	308
Shcbp1l	3	1	0	0	1	2	1	0
Shd	2	6	8	7	2	1	1	2
She	116	83	115	103	110	91	88	71
Shf	176	175	166	226	210	157	191	143
Shfl	272	369	302	257	249	240	218	257
Shh	2	15	4	5	3	3	13	3
Shisa2	277	278	352	294	337	246	228	200
Shisa3	55	33	21	41	54	49	35	35
Shisa4	29	29	16	43	31	15	19	12
Shisa5	5865	6537	6327	4977	6243	5918	5262	5592
Shisa6	3	0	0	1	1	4	2	1
Shisa7	10	12	7	8	11	13	10	11
Shisa8	0	0	0	1	4	4	0	0
Shisa9	8	9	11	7	0	4	0	12
Shisal1	78	99	89	87	49	74	80	88
Shisal2a	0	0	0	0	0	0	1	0
Shisal2b	4	10	1	1	0	1	1	10
Shkbp1	1150	1239	1139	1139	1280	1079	1228	1167
Shld1	142	165	191	197	189	223	214	172
Shld2	55	76	70	54	60	61	53	65
Shld3	138	202	122	131	123	157	146	122
Shmt1	1469	1534	1491	1293	1441	1368	1286	1383
Shmt2	1478	1569	1541	1381	1471	1380	1235	1365
Shoc1	2	2	2	2	4	2	2	1
Shoc2	3069	3209	2861	3104	3092	3265	3114	2970
Shox2	0	0	0	0	0	8	1	4
Shpk	1304	1376	1197	1276	1210	1405	1232	1305
Shprh	487	496	492	527	514	631	493	420
Shq1	161	263	172	214	231	253	189	238
Shroom1	156	208	175	139	188	213	176	233
Shroom2	1032	1013	1043	1069	1062	1064	866	863
Shroom3	11456	11889	10719	10172	11551	10810	10655	10215
Shroom4	46	38	33	39	54	28	33	26
Shtn1	1143	1281	1213	1221	1235	1370	1120	1070
Siae	1378	1395	1363	1251	1469	1555	1625	1546
Siah1a	728	855	726	763	703	727	748	696

Transcriptome sequencing yielded total genetic results for the MOD and APS groups, with a total of 15,936 variables

Continued from above

Siah1b	131	188	159	183	142	156	140	161
Siah2	394	454	441	399	497	440	492	489
Sidt1	357	448	440	409	423	357	366	323
Sidt2	8022	8672	7899	7428	7972	8124	8237	7726
Sigirr	941	971	969	848	1124	1106	913	974
Siglec1	117	190	127	115	115	124	105	116
Siglec15	0	0	0	0	0	4	0	1
Siglece	76	71	86	90	38	53	67	92
Siglecf	40	21	25	24	43	44	33	41
Siglecg	0	2	0	8	0	5	0	0
Siglech	4	0	0	0	0	0	0	0
Sigmar1	925	1018	963	748	962	885	807	900
Sik1	348	323	398	300	400	258	300	241
Sik2	807	794	798	726	769	793	811	773
Sik3	990	1119	940	1042	983	987	1001	950
Sike1	526	509	528	595	518	579	552	498
Sil1	645	786	755	660	737	678	627	762
Sim2	0	0	0	0	1	0	0	0
Simc1	151	175	166	154	176	134	135	142
Sin3a	1383	1443	1407	1466	1407	1433	1343	1358
Sin3b	2127	2119	2161	2042	2170	2208	1974	2000
Sinhcaf	354	373	361	410	395	379	324	381
Sipa1	391	351	415	419	464	388	352	353
Sipa1l1	2898	3087	2812	2768	3307	3128	2768	2760
Sipa1l2	1085	1128	1286	1270	1049	1299	1075	1071
Sipa1l3	6944	7126	6904	6428	6610	6657	6554	6062
Sirpa	613	613	531	515	449	496	400	462
Sirpb1a	10	8	11	15	3	1	6	7
Sirpb1b	14	8	9	9	4	8	5	5
Sirpb1c	8	15	12	28	10	7	22	4
Sirt1	626	669	691	697	736	725	808	662
Sirt2	1608	1645	1595	1592	1646	1538	1620	1614
Sirt3	866	707	790	944	643	743	672	723
Sirt4	141	185	182	146	136	139	180	147
Sirt5	129	189	164	195	196	139	127	152
Sirt6	490	505	499	518	465	487	464	432
Sirt7	2797	2769	2832	2645	2877	2974	2776	2657
Sis	78755	88651	85537	85116	99323	110469	130996	106359
Sit1	0	10	4	1	19	3	4	1
Siva1	272	334	251	293	267	241	229	261
Six4	1	1	1	2	1	5	0	5
Six5	116	108	86	109	116	130	96	70
Ska1	94	125	96	75	115	95	121	75
Ska2	43	118	95	145	135	131	104	111
Ska3	197	212	178	192	225	245	172	203
Skap1	16	16	32	12	16	40	38	18
Skap2	2052	2089	2014	2136	2230	2381	2139	2018
Ski	2748	3064	2830	2835	2927	3123	2611	2672
Skida1	4	12	7	7	7	21	8	8
Skil	3093	3690	2993	2672	3849	4144	3853	3023
Skint3	0	0	0	0	0	4	6	0
Skint8	0	0	0	0	0	1	0	0
Skint9	0	0	0	0	0	1	0	0
Skiv2l	3560	3531	3457	3417	3478	3498	3295	3107
Skor1	1	0	0	0	0	0	0	0
Skp1	5787	6202	5730	5927	6687	6778	6659	6367
Skp2	327	349	364	343	391	423	305	349
Sla	208	270	198	175	182	237	186	174

Transcriptome sequencing yielded total genetic results for the MOD and APS groups, with a total of 15,936 variables

Continued from above

Sla2	20	10	21	13	13	6	2	7
Slain1	152	157	150	131	179	116	127	121
Slain2	3676	3648	3437	3200	3448	3524	3538	3374
Slamf1	5	2	4	7	6	10	2	4
Slamf6	23	30	39	25	20	22	25	40
Slamf7	94	138	131	89	105	109	121	99
Slamf8	84	82	85	79	72	59	16	40
Slamf9	81	51	97	66	81	62	27	41
Slbp	1494	1559	1436	1439	1413	1573	1360	1451
Slc10a1	0	0	1	2	4	3	0	1
Slc10a2	10	15	4	10	15	11	21	7
Slc10a3	435	451	423	458	397	461	414	411
Slc10a4	22	17	24	23	15	12	16	13
Slc10a5	1323	1715	1612	1241	2112	2280	2144	1777
Slc10a6	13	27	32	20	9	15	8	10
Slc10a7	505	555	600	570	588	551	549	515
Slc11a1	213	191	157	180	192	167	167	136
Slc11a2	3254	3623	3567	3495	4209	4306	3678	3311
Slc12a1	0	1	0	0	0	0	0	0
Slc12a2	5279	6081	6207	6152	5498	5636	5477	4921
Slc12a3	2	0	5	1	0	0	6	0
Slc12a4	177	145	197	181	158	155	180	184
Slc12a5	11	18	15	6	8	16	4	8
Slc12a6	1537	2034	1909	1871	1961	1832	1898	1661
Slc12a7	7618	7977	7942	7502	7853	7801	7629	7484
Slc12a8	2237	2649	2663	2453	2661	2711	2159	2201
Slc12a9	739	735	722	720	665	700	545	604
Slc13a1	15526	16524	13597	14182	14525	15017	14120	12865
Slc13a2	4780	5516	5102	4020	5783	6773	7832	6830
Slc13a3	7	0	12	2	5	1	3	1
Slc13a4	7	0	1	0	0	0	0	0
Slc13a5	2	0	4	0	0	2	0	2
Slc14a1	24	27	10	24	19	38	21	21
Slc14a2	0	1	0	0	0	0	0	0
Slc15a1	12582	12489	10879	11734	10210	11690	13563	11290
Slc15a2	94	75	71	77	107	85	52	108
Slc15a3	359	363	362	328	271	233	240	211
Slc15a4	386	408	474	448	544	468	422	454
Slc15a5	0	1	0	0	1	0	1	4
Slc16a1	6470	6413	6360	7076	6109	5957	5325	5356
Slc16a10	9679	11187	9575	8529	10820	11148	11899	10792
Slc16a11	73	79	66	63	114	85	76	77
Slc16a12	85	82	119	81	114	83	79	70
Slc16a13	287	320	340	227	427	326	318	373
Slc16a14	0	0	0	0	0	0	0	1
Slc16a2	52	40	58	20	42	28	42	61
Slc16a3	3625	3470	3526	3559	4170	3926	4112	4080
Slc16a4	8	2	3	1	3	3	10	1
Slc16a5	5199	5571	5906	5733	6277	6507	6345	5823
Slc16a6	2745	3235	2714	2217	3904	4074	4433	3830
Slc16a7	50	30	74	43	31	35	64	30
Slc16a8	11	8	5	0	1	2	5	3
Slc16a9	248	227	237	202	318	357	375	354
Slc17a1	0	0	0	4	0	0	0	2
Slc17a2	0	0	1	1	2	1	1	2
Slc17a3	0	3	1	0	6	1	1	0
Slc17a4	651	825	684	558	909	1065	899	705
Slc17a5	662	630	613	698	689	581	674	599

Transcriptome sequencing yielded total genetic results for the MOD and APS groups, with a total of 15,936 variables

Continued from above

Slc17a6	0	0	0	0	0	0	0	1
Slc17a7	1	1	0	1	1	0	0	3
Slc17a8	0	10	1	1	8	1	5	1
Slc17a9	64	81	61	90	106	85	72	87
Slc18a1	443	536	572	502	484	553	511	506
Slc18a2	13	30	25	30	25	14	34	27
Slc18a3	19	3	6	20	7	20	11	31
Slc18b1	2100	2415	2357	1909	2772	2612	2493	2370
Slc19a1	1982	1955	1833	1698	2007	1996	2169	2103
Slc19a2	738	713	876	819	734	638	649	818
Slc19a3	864	1033	824	722	968	919	1021	1211
Slc1a1	2166	1949	2035	2790	1379	1390	1454	1471
Slc1a2	27	7	18	28	20	18	13	30
Slc1a3	152	115	178	168	184	199	150	138
Slc1a4	257	271	294	262	364	247	264	263
Slc1a5	1478	1665	1577	1722	1420	1434	1480	1372
Slc1a6	3	0	0	0	0	0	0	1
Slc1a7	3	13	4	8	10	8	18	6
Slc20a1	1730	1249	1332	2147	1312	1358	1504	1323
Slc20a2	914	1077	994	866	853	930	988	814
Slc22a1	2426	2756	2488	2394	2847	2919	2765	2652
Slc22a12	0	0	1	1	0	0	0	0
Slc22a13	0	4	0	2	0	0	0	2
Slc22a14	0	0	0	0	0	5	0	0
Slc22a15	247	262	287	203	298	203	184	253
Slc22a16	0	0	1	0	0	0	0	0
Slc22a17	44	69	37	47	59	34	63	49
Slc22a18	4998	4748	4741	4684	4158	4420	4234	4033
Slc22a21	366	333	336	341	342	409	340	353
Slc22a23	1946	2299	2019	1970	2267	2436	2489	2349
Slc22a26	0	1	2	1	0	1	1	0
Slc22a27	1	0	0	0	0	0	0	0
Slc22a29	0	0	1	1	0	0	0	0
Slc22a3	5	4	7	5	5	19	3	10
Slc22a30	0	0	0	0	0	1	0	3
Slc22a4	644	705	650	681	701	655	811	671
Slc22a5	3383	3777	3211	2927	4556	4724	4601	4249
Slc23a1	231	299	203	131	318	365	382	325
Slc23a2	2981	3684	3352	2738	3834	3806	3292	3266
Slc23a3	64	54	84	77	40	57	50	34
Slc23a4	2646	2998	2306	2218	2492	2529	2922	2703
Slc24a2	4	2	2	1	1	1	4	3
Slc24a3	178	260	275	276	229	239	246	201
Slc24a4	0	1	1	0	0	1	1	0
Slc24a5	11	22	3	8	2	7	9	18
Slc25a1	7282	7672	7247	7025	7212	6888	6828	6774
Slc25a10	8860	8752	8511	7761	8284	8406	8183	8304
Slc25a11	7612	7879	7198	7481	7109	7633	7354	7208
Slc25a12	926	849	874	1020	866	867	685	867
Slc25a13	1144	1296	1151	980	1321	1469	1231	1148
Slc25a14	123	170	126	150	139	132	115	138
Slc25a15	11801	13825	12380	11433	12635	13207	12519	11761
Slc25a16	807	891	858	861	828	831	818	855
Slc25a17	615	639	660	684	737	711	646	757
Slc25a18	0	0	0	0	1	0	0	0
Slc25a19	399	415	367	362	446	368	388	360
Slc25a2	1	2	1	6	0	1	0	0
Slc25a20	3495	3620	3365	3606	3944	3757	3731	3669

Transcriptome sequencing yielded total genetic results for the MOD and APS groups, with a total of 15,936 variables

Continued from above

Slc25a21	0	1	0	0	1	0	0	0
Slc25a22	4843	5284	4630	3881	5235	5437	5569	4818
Slc25a23	184	238	245	271	208	246	244	224
Slc25a24	7470	7571	6874	6800	6986	7376	7466	6530
Slc25a25	1149	1301	1367	1314	1502	1442	1363	1266
Slc25a26	350	339	276	346	298	311	250	377
Slc25a27	24	32	28	4	27	17	29	16
Slc25a28	2573	2462	2702	2655	2441	2287	2346	2307
Slc25a29	25	31	43	41	29	48	41	41
Slc25a3	56090	55885	52998	53171	52141	53705	51737	51337
Slc25a30	121	130	108	113	133	181	129	119
Slc25a31	1	0	0	0	0	0	4	0
Slc25a32	679	604	729	616	815	786	678	731
Slc25a33	205	151	193	153	255	306	177	218
Slc25a34	1059	1079	1058	1137	1239	1262	1269	1187
Slc25a35	402	340	386	346	317	318	251	283
Slc25a36	2643	2789	2905	2558	2942	3195	3210	2698
Slc25a37	1481	1812	1819	1735	1816	1835	1708	1794
Slc25a38	728	973	812	808	834	851	772	875
Slc25a39	9531	8959	9403	9255	8537	8874	8183	8596
Slc25a4	1345	1092	1183	1276	1144	1235	1137	1242
Slc25a40	259	233	183	236	251	205	223	171
Slc25a41	0	0	4	0	0	0	0	0
Slc25a42	367	369	391	353	402	377	392	380
Slc25a43	49	50	33	64	49	66	44	44
Slc25a44	3578	3906	3340	3306	3907	4187	4146	3661
Slc25a45	8640	9891	9038	7640	10035	10496	10347	9336
Slc25a46	2343	2542	2116	2334	2481	2682	2705	2552
Slc25a47	49	44	51	49	25	33	54	25
Slc25a48	1	2	1	4	4	1	2	5
Slc25a5	75620	79929	76036	75549	72089	75961	76635	73795
Slc25a51	5025	5264	5241	5216	5279	5517	5610	5179
Slc25a53	36	44	40	53	23	14	53	15
Slc26a1	1	1	1	0	3	1	2	6
Slc26a10	265	358	360	305	239	230	262	251
Slc26a11	158	109	116	117	115	122	114	123
Slc26a2	1878	2365	1961	1814	2707	2978	3006	2562
Slc26a3	5241	6235	6069	5537	6869	7013	6822	6661
Slc26a4	9	7	14	11	13	17	0	11
Slc26a5	1	0	0	1	2	0	0	0
Slc26a6	30848	30673	26677	27233	28489	29851	30324	29812
Slc26a7	0	0	0	1	0	1	0	0
Slc26a8	2	5	0	0	9	3	5	2
Slc26a9	0	0	6	0	4	1	3	1
Slc27a1	273	274	233	248	251	343	333	304
Slc27a2	5471	5065	5436	6117	4899	5363	5727	4982
Slc27a3	42	49	64	38	75	35	26	47
Slc27a4	41335	48656	38465	31483	47072	48765	47878	42795
Slc27a5	46	12	25	23	13	38	34	15
Slc27a6	16	15	15	6	29	1	12	10
Slc28a1	5157	3665	4319	5603	2594	2549	2594	2234
Slc28a2	6117	6291	4630	4690	4697	5355	5759	5160
Slc28a2b	37	33	26	29	9	29	20	24
Slc28a3	1245	1319	1319	1380	1381	1271	1284	1265
Slc29a1	669	610	569	656	575	563	414	484
Slc29a2	40	77	56	54	51	34	26	50
Slc29a3	292	316	300	312	298	312	280	261
Slc29a4	31	40	51	39	31	29	41	45

Transcriptome sequencing yielded total genetic results for the MOD and APS groups, with a total of 15,936 variables

Continued from above

Slc2a1	1136	1174	1151	1292	1189	1184	1031	1097
Slc2a10	188	142	101	149	147	119	132	131
Slc2a12	0	0	6	3	1	2	2	10
Slc2a13	30	36	27	37	26	39	21	35
Slc2a2	9163	10117	9334	8768	11396	12151	14362	12286
Slc2a3	49	42	52	51	52	28	42	28
Slc2a4	143	116	182	144	180	169	176	155
Slc2a5	8956	8475	7985	9169	8144	8632	10239	9474
Slc2a6	225	196	128	166	141	160	160	144
Slc2a7	1924	2102	1778	1764	1688	1818	2009	1750
Slc2a8	200	164	166	229	194	177	210	156
Slc2a9	1815	1858	1682	1831	1359	1540	1690	1539
Slc30a1	2034	2245	1907	1753	2281	2226	1971	1899
Slc30a10	2825	2953	2719	2728	2808	2666	2862	2592
Slc30a2	2368	2625	2084	1946	2346	2491	2588	2336
Slc30a3	1	0	1	0	1	0	0	0
Slc30a4	3311	4051	3437	3210	4192	4299	4418	4028
Slc30a5	5743	6387	5601	4999	6326	6312	6370	5654
Slc30a6	1198	1267	1218	1212	1404	1229	1303	1304
Slc30a7	2270	2593	2399	2383	2849	2685	2430	2583
Slc30a9	2447	2586	2591	2756	2727	2723	2552	2628
Slc31a1	11908	11733	11054	12494	10930	11400	12019	11028
Slc31a2	267	273	298	360	246	256	254	289
Slc32a1	1	0	0	0	0	0	0	0
Slc33a1	3196	3206	2747	2954	3200	3124	3236	2963
Slc34a1	6	8	6	6	1	2	1	0
Slc34a2	2470	3355	1472	747	2481	2663	2890	1985
Slc34a3	10	11	8	1	1	9	21	14
Slc35a1	526	657	533	616	644	674	494	514
Slc35a2	1022	1119	1033	982	1207	1058	986	990
Slc35a3	8130	8966	8279	8229	8456	8926	8996	8327
Slc35a4	3641	3845	3702	3591	3712	3678	3523	3465
Slc35a5	2151	2285	2052	1927	2200	2182	2232	2042
Slc35b1	7202	7596	7137	6897	7231	7524	7072	7313
Slc35b2	1848	1901	1742	1921	1890	1651	1719	1799
Slc35b3	1666	1697	1445	1475	1484	1772	1537	1513
Slc35b4	529	523	537	489	440	580	535	459
Slc35c1	5490	5424	5517	5198	6603	6251	5647	5988
Slc35c2	10541	10174	9985	10331	9175	9443	10332	9684
Slc35d1	2823	2945	2516	2787	2611	2675	2762	2549
Slc35d2	700	840	693	693	610	628	650	653
Slc35d3	18	21	9	20	16	15	13	10
Slc35e1	8412	9309	8614	8409	9511	9343	9393	9129
Slc35e2	3221	3464	3023	3283	2853	2974	3070	2900
Slc35e3	421	547	447	450	532	592	551	663
Slc35e4	45	66	60	58	50	76	47	44
Slc35f1	11	10	19	12	4	4	11	12
Slc35f2	4598	5428	4454	3991	5367	5547	5167	4650
Slc35f3	8	1	4	5	4	5	6	4
Slc35f5	4841	5889	4664	4594	6012	6002	5876	5493
Slc35f6	1497	1462	1448	1464	1263	1389	1376	1413
Slc35g1	8278	8477	8242	7828	8942	9072	9175	9015
Slc35g2	64	67	76	73	78	67	42	54
Slc35g3	0	0	0	0	4	0	0	0
Slc36a1	12972	12231	10126	11385	8605	9273	10554	9349
Slc36a2	6	2	9	14	22	8	16	9
Slc36a3	0	0	0	0	3	0	0	0
Slc36a4	138	154	142	122	103	134	93	103

Transcriptome sequencing yielded total genetic results for the MOD and APS groups, with a total of 15,936 variables

Continued from above

Slc37a1	3696	4035	3851	3548	3799	3883	3909	3829
Slc37a2	446	463	424	464	476	440	343	356
Slc37a3	1741	1790	1799	1613	1792	1992	1954	1943
Slc37a4	2209	2445	2290	2431	2842	2867	2953	2785
Slc38a1	1529	1685	1550	1575	1599	1611	1480	1443
Slc38a10	5738	6661	5667	5327	6215	6293	6306	5695
Slc38a11	55	57	71	68	93	41	39	61
Slc38a2	2405	2855	2918	2725	2843	3053	2782	3115
Slc38a3	11	9	6	20	51	34	23	21
Slc38a4	35	2	7	22	6	16	9	8
Slc38a5	1	2	1	0	3	21	1	8
Slc38a6	284	310	277	273	273	292	294	289
Slc38a7	629	749	610	563	799	829	677	661
Slc38a8	35	74	20	44	38	50	46	57
Slc38a9	228	258	256	261	292	314	267	248
Slc39a1	4507	4671	4391	4211	4411	4633	4339	4429
Slc39a10	337	378	318	370	441	400	317	366
Slc39a11	3242	3563	3572	3383	3224	3617	3722	3526
Slc39a12	0	0	4	0	0	0	0	0
Slc39a13	197	174	152	159	186	176	206	196
Slc39a14	4380	5443	5154	4504	6466	6669	6585	6170
Slc39a2	2	4	11	3	9	12	4	14
Slc39a3	948	966	1010	1045	973	1131	902	1084
Slc39a4	6562	7176	6156	5740	6698	6608	6784	6880
Slc39a5	7070	7891	7276	6638	7535	7578	7198	7186
Slc39a6	139	143	124	147	136	155	108	142
Slc39a7	4138	4535	4302	4224	4788	4808	4394	4237
Slc39a8	460	576	593	502	508	578	592	562
Slc39a9	9315	10286	8905	8699	10550	10774	10843	10232
Slc3a1	8833	10155	7895	7575	10825	10973	11510	9847
Slc3a2	11371	11756	11091	10643	11103	11740	11595	10650
Slc40a1	2453	3027	2305	1945	2931	2982	2341	2344
Slc41a1	443	500	455	418	522	499	476	449
Slc41a2	2205	2331	2227	2199	2188	2370	2574	2351
Slc41a3	130	128	101	126	116	106	105	117
Slc43a1	36	28	68	45	43	29	33	39
Slc43a2	17400	18837	15353	14334	15766	17169	18305	15894
Slc43a3	797	715	729	815	603	539	634	537
Slc44a1	5544	5974	5515	5313	5692	5921	5754	5347
Slc44a2	310	263	300	302	325	270	216	243
Slc44a3	1641	1486	1731	1606	1416	1398	1303	1389
Slc44a4	8004	8707	8200	7278	8652	8698	8564	8118
Slc44a5	2	8	0	1	2	0	1	3
Slc45a1	1	4	2	6	5	4	8	5
Slc45a2	0	0	0	1	0	0	0	0
Slc45a3	42	38	49	39	54	44	47	55
Slc45a4	387	362	379	399	434	437	344	339
Slc46a1	7518	8413	6627	5695	8427	8563	8857	7950
Slc46a3	2129	2356	1902	1831	2263	2352	2684	2281
Slc47a1	1026	1035	976	866	1048	1101	1090	1131
Slc47a2	1	1	1	0	4	4	0	1
Slc48a1	493	434	548	555	501	501	407	468
Slc49a4	3338	3289	3110	3279	3461	3240	3311	3194
Slc4a1	0	0	1	0	0	0	1	0
Slc4a10	0	5	0	12	1	8	3	5
Slc4a11	11	4	4	5	5	19	14	3
Slc4a1ap	712	584	688	614	648	643	621	650
Slc4a2	4865	4602	4809	5091	4050	4249	3978	4142

Transcriptome sequencing yielded total genetic results for the MOD and APS groups, with a total of 15,936 variables

Continued from above

Slc4a3	67	62	41	67	51	37	36	97
Slc4a4	4339	4774	4207	4081	4289	4350	4400	4001
Slc4a5	1030	1178	1122	873	1131	1308	1242	950
Slc4a7	5824	6931	5201	4752	6250	6031	6072	5570
Slc4a8	48	34	34	46	38	38	53	45
Slc4a9	0	1	0	0	0	0	0	4
Slc50a1	620	677	683	688	745	690	541	679
Slc51a	6116	6400	5980	5856	5470	5952	5949	5299
Slc51b	2846	2780	2478	2264	2373	2880	2696	2585
Slc52a2	3527	3769	3648	3405	3484	3520	3310	3361
Slc52a3	2362	2249	2264	2313	2202	2186	2588	2623
Slc5a1	102467	98602	92567	103521	88705	94786	112739	103484
Slc5a10	15	8	13	6	10	12	7	12
Slc5a11	2705	3070	2384	2310	2872	3005	3177	2755
Slc5a12	963	1314	912	541	1800	1950	2499	1836
Slc5a2	35	34	30	24	23	24	14	37
Slc5a3	143	272	142	221	283	252	264	217
Slc5a4a	1005	1009	1063	922	848	869	1042	1052
Slc5a4b	1035	1232	1166	996	1127	1151	1085	931
Slc5a5	18	1	6	7	9	4	16	24
Slc5a6	684	728	669	612	666	645	694	654
Slc5a7	17	16	3	7	14	15	28	11
Slc5a8	831	590	587	595	475	506	721	634
Slc5a9	1532	1574	1270	1561	1079	1146	1411	1216
Slc66a1	680	655	686	642	728	738	679	668
Slc66a2	336	289	393	323	365	304	254	345
Slc6a1	1	0	0	1	0	6	1	0
Slc6a11	5	14	10	4	5	9	1	2
Slc6a12	2	1	5	0	0	1	2	2
Slc6a13	0	1	2	2	5	2	2	5
Slc6a14	1	0	4	0	1	0	7	2
Slc6a15	6	4	12	4	7	2	0	6
Slc6a17	39	45	55	38	38	39	63	43
Slc6a18	20	6	7	13	2	24	11	13
Slc6a19	22512	23106	18245	19707	18072	19736	22911	20260
Slc6a20a	5003	5602	4846	4451	4707	4829	4985	3846
Slc6a20b	12	20	11	20	21	15	14	13
Slc6a21	0	1	0	0	0	0	1	0
Slc6a3	85	116	85	104	134	128	233	173
Slc6a4	3776	3796	3519	3670	2961	3126	3268	2885
Slc6a5	0	0	0	1	4	0	0	0
Slc6a6	8424	9605	9370	9097	9673	9408	9589	9358
Slc6a7	45	45	45	46	47	67	49	49
Slc6a8	33200	34656	30863	29302	32091	34163	36662	33673
Slc6a9	276	251	275	286	299	308	289	273
Slc7a1	2737	3231	2902	2874	2923	2720	2365	2569
Slc7a10	0	0	1	10	0	14	6	1
Slc7a11	27	18	20	18	20	22	17	15
Slc7a14	13	14	21	27	25	31	33	30
Slc7a15	1412	1867	1522	1225	1870	2110	2173	1962
Slc7a2	25	41	22	37	41	40	28	11
Slc7a3	5	3	1	8	7	15	5	7
Slc7a4	222	249	253	207	239	223	183	176
Slc7a5	476	621	523	431	508	519	504	507
Slc7a6	208	217	256	266	244	299	288	225
Slc7a6os	768	886	810	825	856	937	722	677
Slc7a7	11062	12184	10778	9865	12213	12676	12691	11188
Slc7a8	9609	10557	9870	9583	9459	10156	10482	9446

Transcriptome sequencing yielded total genetic results for the MOD and APS groups, with a total of 15,936 variables

Continued from above

Slc7a9	10259	10679	9895	9767	10092	10468	10565	9959
Slc8a1	288	290	352	365	260	347	292	224
Slc8a2	38	19	38	33	22	21	23	32
Slc8a3	20	33	21	12	25	9	21	19
Slc8b1	199	236	239	219	203	223	134	195
Slc9a1	2588	2620	2515	2597	2417	2352	2102	2226
Slc9a2	6730	7521	6473	5995	6135	6713	6572	5622
Slc9a3	24458	24487	20038	21789	20083	20046	20332	19138
Slc9a3r1	46752	48098	43094	41775	44301	46359	47936	45242
Slc9a3r2	457	466	547	550	629	533	467	485
Slc9a4	5	11	0	8	11	5	18	13
Slc9a5	72	81	84	49	77	69	106	134
Slc9a6	756	678	681	661	794	719	732	731
Slc9a7	39	15	31	14	36	37	11	7
Slc9a8	1683	1765	1773	1599	1686	1637	1574	1630
Slc9a9	106	80	71	91	42	81	79	91
Slc9b1	3	6	9	11	0	1	1	3
Slc9b2	0	0	1	0	11	1	1	1
Slc9c1	0	0	0	0	0	0	1	0
Slco1a1	0	0	0	0	3	0	0	4
Slco1a4	0	4	1	0	2	2	4	0
Slco1a5	1	0	0	0	0	0	1	0
Slco1b2	4	3	6	10	2	2	7	4
Slco1c1	3	3	1	4	2	5	3	5
Slco2a1	3821	4375	3238	3129	3092	3423	3874	3340
Slco2b1	2645	2634	2619	2782	2416	2420	1899	2064
Slco3a1	504	608	617	529	586	607	515	526
Slco4a1	349	286	335	433	289	331	293	246
Slco4c1	3	0	0	4	1	9	5	0
Slco5a1	4	0	1	6	2	2	7	10
Slf1	299	332	276	303	385	317	343	265
Slf2	613	764	719	671	810	745	613	631
Slfn1	71	80	96	74	50	82	92	72
Slfn2	4884	5167	4710	4361	4153	4297	4259	3803
Slfn3	16	39	33	19	23	6	34	13
Slfn4	1621	2924	2655	1542	1824	1475	1243	1187
Slfn5	2122	2968	2691	1864	2140	2565	2114	1847
Slfn8	117	141	215	136	134	126	111	95
Slfn9	1393	1805	1529	1551	1811	1758	1574	1402
Slfnl1	0	0	0	1	0	0	1	0
Slirp	913	906	1049	975	982	1087	949	958
Slit1	15	13	36	13	14	10	19	19
Slit2	46	82	102	106	100	64	46	72
Slit3	202	270	257	262	232	252	234	213
Slitrk1	5	1	0	0	1	0	2	1
Slitrk2	1	1	0	0	2	4	1	1
Slitrk3	5	1	2	3	1	1	2	0
Slitrk4	0	0	1	0	1	0	4	1
Slitrk5	0	5	5	2	3	0	1	4
Slitrk6	326	280	336	301	325	308	315	281
Slk	3411	3001	3534	3946	3061	3164	3051	2955
Slmap	3870	4191	3718	3861	4006	4223	4216	3739
Slpi	108	100	120	140	76	150	168	145
Sltm	1371	1619	1429	1612	1611	1845	1556	1509
Slu7	1494	1693	1571	1522	1617	1735	1662	1541
Slurp1	0	0	1	0	0	0	0	0
Slx1b	223	234	213	226	207	275	212	202
Slx4	1085	1213	1089	1056	1305	1211	1186	893

Transcriptome sequencing yielded total genetic results for the MOD and APS groups, with a total of 15,936 variables

Continued from above

Slx4ip	341	415	333	333	391	426	424	388
Smad1	2519	2756	2737	2450	2550	2812	2433	2558
Smad2	1100	1265	1171	1133	1149	1310	1196	1187
Smad3	1191	1272	1145	1087	1093	1211	1258	1062
Smad4	9482	10576	8681	8444	8966	9382	9544	8829
Smad5	975	980	1038	1163	937	1076	987	829
Smad6	658	775	631	589	979	863	788	602
Smad7	1524	1489	1610	1531	1675	1907	1599	1416
Smad9	38	91	57	65	38	47	46	52
Smagp	2892	3001	2804	2718	2835	3111	2763	2909
Smap1	3403	3247	3163	3215	3282	3292	3224	3221
Smap2	3083	3045	3042	2892	3175	3092	2848	3178
Smarca1	30	14	30	12	22	6	17	11
Smarca2	696	626	668	651	783	727	744	657
Smarca4	4313	4785	4635	4443	4472	4500	4261	4235
Smarca5	2886	2920	2743	2886	3381	3460	3112	3045
Smarcad1	645	644	569	609	584	574	687	652
Smarcal1	365	332	375	395	452	370	361	357
Smarcb1	1540	1771	1562	1543	1689	1563	1424	1440
Smarcc1	1594	1853	1806	1599	1892	1902	1712	1605
Smarcc2	4842	5426	5046	4958	4647	5105	4756	4488
Smarcd1	780	849	656	834	693	791	703	732
Smarcd2	4633	4675	4377	4308	4617	4806	4355	4439
Smarcd3	74	85	107	65	72	73	65	61
Smarce1	1800	2009	1883	1803	2012	1949	1769	1898
Smbd1	19	40	32	16	26	18	19	25
Smc1a	2091	2351	2149	2186	2245	2487	2416	2130
Smc1b	6	2	0	7	1	1	1	13
Smc2	1009	1015	1097	945	1168	1254	1030	978
Smc3	1150	1336	1194	1194	1265	1419	1271	1173
Smc4	1469	1787	1469	1595	1756	1762	1776	1529
Smc5	412	420	458	450	410	376	472	412
Smc6	1224	1206	1318	1203	1355	1481	1340	1245
Smchd1	1234	1392	1234	1323	1614	1517	1446	1308
Smco3	0	0	0	0	1	0	1	1
Smco4	420	400	360	447	388	431	454	354
Smcp	1	2	6	0	0	12	1	0
Smcr8	1792	1715	1736	1745	1824	1917	1962	1701
Smdt1	5587	5375	5313	5368	5101	5548	5055	5181
Smg1	6123	7185	6237	6590	6801	6734	6644	5982
Smg5	3038	3486	3681	3426	3518	3445	3304	3112
Smg6	919	996	956	1025	942	894	916	803
Smg7	2207	2602	2336	2173	2265	2403	2284	2102
Smg8	705	690	662	680	669	714	659	834
Smg9	565	658	607	564	665	581	522	636
Smim1	78	112	83	81	90	91	92	49
Smim10l1	996	1013	1055	1015	1157	1064	1068	956
Smim10l2a	1	2	1	5	1	2	5	1
Smim11	259	331	349	316	357	376	314	328
Smim12	415	485	456	423	455	407	368	415
Smim13	416	446	430	479	509	481	450	502
Smim14	2386	2354	2423	2414	2431	2477	2385	2431
Smim15	1759	1918	1699	1721	1971	2165	2269	1897
Smim17	1	0	0	1	0	1	1	0
Smim18	0	1	0	0	0	0	0	0
Smim19	329	269	271	289	300	295	239	298
Smim20	1318	1290	1306	1320	1256	1235	1234	1229
Smim22	1577	1472	1601	1759	1293	1631	1389	1407

Transcriptome sequencing yielded total genetic results for the MOD and APS groups, with a total of 15,936 variables

Continued from above

Smim24	41538	42592	40962	40634	38926	42508	43226	39838
Smim26	175	176	189	154	211	192	140	157
Smim27	69	101	77	66	67	68	80	107
Smim3	273	272	326	335	246	282	184	235
Smim31	2930	3205	3098	3205	3481	3687	3708	3374
Smim38	22	7	17	11	26	20	27	16
Smim4	201	238	201	194	178	217	193	213
Smim40	5	4	0	0	1	4	1	1
Smim41	0	0	0	0	0	0	1	0
Smim43	0	0	0	4	8	0	0	0
Smim5	31	37	72	50	35	56	29	50
Smim6	1015	912	1078	1064	999	1046	966	975
Smim7	4181	4221	4136	4324	4150	4155	4189	4253
Smim8	304	360	374	354	326	362	337	357
Smim9	19	23	16	18	26	20	15	7
Smlr1	692	710	745	657	712	779	656	596
Smn1	581	549	506	511	528	541	439	437
Smndc1	1689	1788	1734	1809	1615	1839	1617	1612
Smo	436	337	365	368	376	373	349	316
Smoc1	125	123	136	88	100	120	93	93
Smoc2	1687	1937	2173	1865	1915	1786	1742	1700
Smox	749	792	714	661	917	981	780	810
Smpd1	404	363	369	328	336	373	352	350
Smpd2	2270	2386	2311	1994	2289	2523	2240	2450
Smpd3	7585	7770	7907	7271	7661	7831	7446	7059
Smpd4	668	867	826	748	779	757	619	679
Smpd5	9	9	12	10	14	10	10	11
Smpdl3a	7724	7890	7437	7763	7693	8572	8969	8244
Smpdl3b	7064	7941	6374	5254	7030	7398	7645	7146
Smpx	4	3	10	8	18	4	14	11
Sms	554	610	627	567	667	637	646	654
Smtn	1281	1505	1575	1427	1405	1434	1475	1394
Smtnl1	11	11	7	12	4	5	3	14
Smtnl2	43	50	71	87	87	68	60	49
Smu1	2589	2772	2736	2623	2857	2883	2723	2655
Smug1	601	655	755	743	677	722	596	627
Smurf1	1238	1275	1279	1300	1134	1107	1211	1153
Smurf2	2560	2688	2652	2535	3073	3072	2946	2652
Smyd1	0	1	1	4	3	1	7	5
Smyd2	707	778	658	692	657	626	544	576
Smyd3	112	170	198	142	168	217	169	125
Smyd4	78	52	101	88	86	69	103	84
Smyd5	514	536	371	378	507	475	368	379
Snai1	84	63	54	48	39	67	42	77
Snai2	32	34	13	42	27	24	8	10
Snai3	13	12	14	27	11	10	22	18
Snap23	1541	1678	1583	1686	1679	1745	1758	1632
Snap25	76	75	67	78	66	74	72	58
Snap29	2138	2688	2444	2458	2510	2507	2446	2401
Snap47	470	523	422	402	497	483	434	449
Snap91	23	22	15	30	14	22	36	27
Snapc1	304	507	337	370	444	415	387	382
Snapc2	420	359	403	475	413	343	359	336
Snapc3	391	438	388	421	546	494	410	443
Snapc4	300	256	273	273	301	301	245	257
Snapc5	766	724	726	743	686	804	757	761
Snapin	436	585	544	555	515	499	486	470
Snca	2	10	2	0	6	7	8	6

Transcriptome sequencing yielded total genetic results for the MOD and APS groups, with a total of 15,936 variables

Continued from above

Sncaip	57	37	36	40	49	40	19	18
Sncb	1	0	6	9	5	7	7	0
Sncg	65	56	80	73	85	78	104	63
Snd1	8064	8332	7905	7564	8129	8025	7590	7811
Sned1	33	38	29	9	18	35	31	13
Snf8	1901	1874	2005	2091	1930	1941	1733	1758
Snip1	836	782	824	871	841	662	804	775
Snn	47	95	64	100	74	50	77	54
Snph	776	738	778	836	553	659	614	541
Snrk	1592	1891	1766	1548	1852	1991	2064	1853
Snrnp200	4088	4602	4152	3867	4344	4243	4046	3755
Snrnp25	204	252	219	260	276	267	218	305
Snrnp27	631	732	727	662	640	737	639	663
Snrnp35	324	362	299	344	338	334	291	299
Snrnp40	1133	1206	1191	1142	1282	1158	1032	1098
Snrnp48	322	398	338	348	423	495	339	405
Snrnp70	3209	3510	3517	3317	3666	3186	2967	3115
Snrpa	1989	2068	1991	1972	2047	1959	1866	1813
Snrpa1	694	824	760	779	783	733	731	674
Snrpb	3350	3266	3350	3124	3458	3303	2945	3150
Snrpb2	674	697	711	625	802	834	758	696
Snrpc	1275	1523	1299	1323	1353	1222	1261	1301
Snrpd1	980	1049	900	918	1005	1017	956	821
Snrpd2	1541	1546	1589	1504	1704	1599	1417	1516
Snrpd3	1440	1639	1526	1577	1554	1564	1535	1517
Snrpe	1417	1371	1246	1322	1456	1419	1321	1239
Snrpf	998	947	981	866	1105	801	891	982
Snrpg	963	1228	1137	1067	1318	1231	1039	1054
Snrpn	0	0	0	0	0	2	0	0
Snta1	825	790	829	648	749	816	856	856
Sntb1	64	119	37	61	107	81	126	104
Sntb2	205	256	237	323	257	288	289	250
Sntg1	15	11	16	30	14	21	14	3
Sntg2	85	59	75	112	71	101	46	81
Snu13	1739	1862	1710	1643	1978	1922	1648	1744
Snupn	83	130	96	119	94	104	80	107
Snw1	2186	2550	2495	2415	2475	2637	2344	2415
Snx1	4028	4388	4113	3935	4312	4309	4192	4227
Snx10	663	845	766	789	592	674	696	646
Snx11	795	839	750	738	828	820	800	770
Snx12	1080	1132	1084	1158	1133	1159	1001	1069
Snx13	2393	2783	2467	2513	2604	2849	3007	2451
Snx14	1495	1543	1619	1545	1528	1597	1526	1429
Snx15	924	815	790	823	936	830	846	733
Snx16	202	147	198	213	217	224	190	212
Snx17	3462	3661	3474	3591	3440	3583	3289	3214
Snx18	2197	2073	1994	2206	2013	2023	1932	1978
Snx19	1928	2077	2009	1967	1917	2020	1975	1956
Snx2	1844	2129	2174	2201	1990	1928	1993	1989
Snx20	2240	2461	2592	2314	2684	2726	2600	2747
Snx21	274	189	243	270	240	203	236	254
Snx22	273	316	272	302	201	229	217	233
Snx24	224	279	200	216	328	228	185	227
Snx25	4774	4952	4566	4642	4634	4668	4967	4587
Snx27	1701	1755	1761	1523	1882	1811	1757	1589
Snx29	545	530	518	616	361	414	348	386
Snx3	5442	5757	5624	5848	5861	5916	5447	5494
Snx30	68	98	100	90	109	90	98	108

Transcriptome sequencing yielded total genetic results for the MOD and APS groups, with a total of 15,936 variables

Continued from above

Snx31	0	1	1	0	1	3	1	2
Snx32	28	36	36	21	14	9	18	8
Snx33	115	169	124	147	176	157	157	146
Snx4	3379	3715	3523	3406	3657	3780	3419	3665
Snx5	3677	4271	3729	3618	4171	4393	4271	3990
Snx6	2292	2427	2440	2299	2640	2568	2608	2455
Snx7	2315	2472	2409	2161	2599	2523	2318	2390
Snx8	171	185	184	179	175	190	124	130
Snx9	7659	8104	7310	7307	7489	7526	7718	7439
Soat1	1308	1243	1203	1329	1351	1300	1374	1246
Soat2	8693	9502	8305	7606	8772	8890	8910	8409
Sobp	25	36	39	18	34	33	22	16
Socs1	954	685	722	931	426	456	409	417
Socs2	2252	2003	1989	2425	1460	1370	1305	1348
Socs3	4253	4434	4055	4140	4072	4273	4118	4565
Socs4	2507	2737	2717	3016	2771	3097	3002	2795
Socs5	217	253	288	233	291	247	248	257
Socs6	760	889	858	772	773	903	803	758
Socs7	655	730	748	734	768	795	780	704
Sod1	9626	9827	10123	9568	10552	10949	9670	9774
Sod2	4072	4176	3982	4295	3946	4004	3686	3803
Sod3	384	322	455	455	405	321	350	339
Soga1	349	405	412	445	368	360	318	392
Soga3	6	2	0	2	1	1	7	5
Son	7995	9201	8556	8873	8718	9232	9000	8257
Sorbs1	1993	2106	2137	2025	2084	2041	2316	1941
Sorbs2	200	219	189	223	181	216	206	212
Sorbs3	102	143	124	142	125	116	112	112
Sorcs1	8	9	13	6	0	8	1	4
Sorcs2	221	197	270	270	176	149	208	196
Sord	7104	6725	6707	7276	6946	7168	7786	7076
Sorl1	2054	2434	2357	2399	2259	2320	2040	2115
Sort1	6116	6384	6706	6416	6643	6786	6076	5855
Sos1	1388	1394	1313	1322	1378	1412	1428	1345
Sos2	1490	1675	1575	1716	1739	1779	1611	1557
Sost	0	0	0	0	0	4	1	0
Sostdc1	14	19	14	21	13	9	11	20
Sowaha	1554	1628	1680	1658	1295	1329	1443	1443
Sowahb	3390	3574	3215	3210	3756	3512	3972	3577
Sowahc	1028	1207	1125	1021	993	1025	1100	1070
Sowahd	2	2	2	0	11	2	4	4
Sox1	0	0	1	0	0	1	0	0
Sox10	9	19	9	15	45	28	9	19
Sox11	0	5	6	3	6	0	1	6
Sox12	97	116	116	104	61	66	67	70
Sox13	2348	2380	2204	2022	2277	2562	2535	2296
Sox15	3	0	0	2	6	0	0	0
Sox17	30	28	54	23	32	36	34	41
Sox18	165	167	148	143	176	164	130	137
Sox2	1	1	0	2	0	0	4	1
Sox21	2	0	0	0	0	1	0	1
Sox30	1	0	0	0	0	0	0	0
Sox4	796	877	782	727	821	952	878	737
Sox5	31	12	20	16	28	14	15	42
Sox6	1270	1368	1309	1258	1391	1528	1614	1390
Sox7	28	28	28	39	62	34	28	31
Sox8	0	11	5	1	0	8	4	1
Sox9	494	460	509	579	405	479	425	323

Transcriptome sequencing yielded total genetic results for the MOD and APS groups, with a total of 15,936 variables

Continued from above

Sp1	3445	3840	3718	3701	3787	3791	3747	3640
Sp100	3565	4424	4393	3671	3624	3906	3637	3379
Sp110	554	581	555	554	471	403	378	380
Sp140	552	562	549	543	449	532	400	365
Sp2	782	698	726	777	695	631	751	677
Sp3	2749	2945	2834	2888	2953	3030	3059	2765
Sp4	364	350	352	385	245	279	322	246
Sp5	881	790	875	902	945	956	803	851
Sp6	20	22	7	1	5	11	9	7
Sp7	5	0	0	0	0	0	0	0
Sp9	0	1	1	0	0	4	0	0
Spa17	13	20	13	24	13	6	17	20
Spaar	16	9	30	35	16	23	16	8
Spaca1	1	6	5	0	0	0	1	0
Spaca3	0	0	2	0	0	0	0	0
Spaca4	9	5	2	1	0	1	10	0
Spaca6	10	21	4	27	8	10	10	11
Spaca9	0	0	1	4	6	0	0	0
Spag1	39	42	27	20	45	31	28	26
Spag17	0	6	5	5	5	1	4	7
Spag4	0	0	1	3	0	0	0	4
Spag5	630	789	775	642	777	820	695	612
Spag6	0	4	6	9	0	1	4	5
Spag6l	0	0	0	4	0	1	0	0
Spag7	979	1130	1147	1028	1013	950	937	916
Spag8	0	1	1	1	7	1	2	10
Spag9	3457	3926	3332	3660	3804	4068	4440	3966
Sparc	3222	3641	3815	3591	3634	3640	3268	3289
Sparcl1	1669	1790	1786	1846	1510	1465	1505	1500
Spast	1171	1262	1281	1114	1315	1317	1338	1136
Spata1	16	18	12	5	9	3	5	12
Spata13	1321	1401	1399	1329	1262	1280	1230	1213
Spata17	13	3	13	2	9	1	17	13
Spata18	2	1	0	0	0	4	0	4
Spata2	3495	3513	3673	3556	3494	3337	3459	3339
Spata20	0	0	1	0	0	0	0	6
Spata22	9	20	16	14	9	16	7	12
Spata24	14	43	48	61	31	30	24	27
Spata25	0	0	0	0	0	0	1	0
Spata2l	238	224	244	309	190	170	202	211
Spata31d1	1	0	4	0	0	0	0	0
Spata32	0	2	0	0	0	0	0	0
Spata33	1	5	9	10	7	2	8	0
Spata45	0	0	0	0	6	0	0	0
Spata5	366	357	327	387	367	386	320	341
Spata5l1	363	348	396	318	454	422	397	343
Spata6	34	57	52	78	71	56	66	49
Spata7	53	64	72	58	67	52	48	58
Spata9	4	0	1	0	0	0	0	0
Spatc1l	4	0	0	0	0	0	0	0
Spats1	1	0	1	0	0	0	1	0
Spats2	264	342	324	322	330	305	305	266
Spats2l	1954	2521	2262	1804	2250	2168	2188	1856
Spc24	458	544	601	526	637	594	429	442
Spc25	256	352	320	289	345	298	306	269
Spcs1	3457	3457	3390	3234	3653	3568	3448	3537
Spcs2	4754	5130	4901	4739	5698	5471	5172	5324
Spcs3	2895	3398	2946	3063	3485	3407	3248	3141

Transcriptome sequencing yielded total genetic results for the MOD and APS groups, with a total of 15,936 variables

Continued from above

Spdef	727	671	698	671	726	681	635	672
Spdl1	219	240	290	228	292	254	268	266
Spdya	10	4	11	5	10	2	12	7
Specc1	2408	2411	2367	2301	2179	2336	2236	2053
Specc1l	5646	6022	5880	5424	6146	6424	6423	5786
Speer1	0	0	1	1	0	0	0	1
Speer4a	1	0	0	0	0	0	0	0
Speer4c	0	0	0	1	0	0	1	4
Spef1	344	394	372	408	388	416	315	387
Spef2	1	0	0	0	0	0	1	0
Speg	162	139	173	196	145	174	137	155
Spem1	0	0	2	0	4	0	0	0
Spen	2459	3016	2907	2713	2964	2957	3037	2451
Spg11	1359	1711	1536	1386	1461	1557	1600	1343
Spg20	2645	2606	2333	2312	2276	2432	2521	2214
Spg21	1585	2039	1674	1631	1791	1974	1928	1801
Spg7	3168	3431	3235	3167	3070	3312	2916	3083
Sphk1	848	881	881	911	555	668	679	683
Sphk2	5519	5394	4770	4882	5223	5464	5554	5099
Sphkap	49	22	32	25	41	30	22	30
Spi1	389	270	261	318	278	292	267	231
Spib	50	57	37	54	57	71	54	59
Spic	1	0	0	0	9	1	0	0
Spice1	91	140	90	141	174	194	159	133
Spidr	277	300	275	250	209	331	310	199
Spin1	2396	2747	2757	2468	2784	2707	2550	2517
Spin2c	13	8	6	11	6	5	0	2
Spin4	9	18	8	4	10	14	14	15
Spindoc	529	426	483	445	415	451	377	450
Spink1	9527	10683	9627	9271	9453	11060	12090	10365
Spink10	0	1	1	1	1	1	0	1
Spink2	0	1	1	0	1	0	0	1
Spink4	9112	9084	9596	10468	9462	9466	9675	9987
Spink5	0	1	0	0	0	1	5	4
Spink8	0	1	0	0	0	0	0	0
Spint1	13409	13850	12877	12923	13254	13199	13192	13389
Spint2	29625	30598	29482	28389	28062	28503	26811	27610
Spire1	139	141	167	117	134	142	144	104
Spire2	1555	1665	1757	1554	1776	1679	1618	1599
Spn	84	117	110	125	91	100	124	73
Spns1	2047	2159	2163	2056	2119	2209	1995	2165
Spns2	2971	3281	2827	2791	2883	3373	3199	3093
Spns3	103	97	105	98	78	96	95	47
Spo11	8	2	4	4	0	1	0	0
Spocd1	1	0	0	0	0	0	0	0
Spock1	10	13	4	18	3	10	19	6
Spock2	121	151	110	169	115	155	165	173
Spock3	15	19	14	15	21	8	21	6
Spon1	704	735	632	742	564	527	518	588
Spon2	658	692	664	670	643	666	539	475
Spop	2821	2864	2979	3091	2554	2876	2641	2660
Spopl	426	518	487	452	449	531	556	448
Spout1	707	867	784	736	866	941	798	907
Spp1	17	9	27	5	30	19	16	25
Spp2	1	8	0	1	1	1	1	0
Sppl2a	9857	9954	8773	9504	9092	9435	9758	8847
Sppl2b	1039	1042	1120	1153	1017	1040	935	947
Sppl3	2644	2723	2551	2617	2536	2393	2177	2414

Transcriptome sequencing yielded total genetic results for the MOD and APS groups, with a total of 15,936 variables

Continued from above

Spr	2793	2658	2644	2732	2774	2993	2549	2547
Spred1	585	706	734	686	666	710	640	710
Spred2	1218	1523	1311	1337	1282	1307	1376	1190
Spred3	19	28	36	31	15	47	26	32
Spring1	245	251	276	313	301	265	261	212
Sprn	12	13	2	4	8	9	3	1
Sprr1a	103	95	46	51	62	72	97	88
Sprr2a1	7546	5029	7180	10188	3600	3362	4451	4822
Sprr2a2	6683	3078	4333	7604	1958	2509	3074	2309
Sprr2a3	13373	5387	9278	16878	3726	3358	3177	2975
Sprr2b	8	0	3	3	1	7	0	2
Sprr2d	0	1	0	0	0	0	0	0
Sprr2f	1	0	1	0	2	1	0	0
Sprtn	889	915	1010	974	867	939	893	823
Spry1	276	297	314	299	348	191	258	247
Spry2	363	354	441	424	326	353	311	262
Spry3	157	183	227	130	169	206	204	170
Spry4	128	166	153	146	136	140	96	167
Spryd3	1634	1655	1603	1529	1675	1647	1553	1565
Spryd4	615	652	661	594	720	713	597	596
Spryd7	1538	1629	1635	1625	1613	1690	1533	1525
Spsb1	1422	1366	1391	1383	1505	1410	1372	1566
Spsb2	561	584	491	412	528	483	532	537
Spsb3	1388	1449	1319	1415	1364	1390	1255	1296
Spsb4	21	4	2	2	3	15	4	4
Spta1	0	0	0	0	1	0	1	0
Sptan1	11199	11960	10939	10935	12132	12423	11364	10996
Sptb	21	27	22	18	6	11	25	9
Sptbn1	13155	15113	13366	13201	14525	14950	14917	13636
Sptbn2	25	17	32	11	27	30	24	17
Sptbn4	15	32	24	30	42	16	10	34
Sptbn5	19	7	31	21	22	7	18	22
Sptlc1	2298	2484	2318	2243	2326	2416	2218	2350
Sptlc2	11279	11961	10595	10320	11322	11701	11617	11206
Sptssa	5134	5138	5049	5142	5477	5836	5288	4784
Sptssb	149	172	151	151	197	144	100	152
Spty2d1	1151	1514	1263	1194	1499	1429	1348	1350
Spx	8	0	6	0	6	0	1	3
Sqle	2913	3134	3503	3549	3050	3041	3028	3018
Sqor	4268	4750	4185	4162	4847	4792	5090	4640
Sqstm1	5821	6097	5509	5443	6074	6128	5766	5663
Sra1	2331	2316	2394	2296	2110	2216	2206	2185
Srarp	0	1	0	2	0	0	0	1
Srbd1	412	450	429	403	477	447	438	411
Src	7141	7380	7017	6772	6987	7232	7527	6965
Srcap	3539	4336	4146	4121	3958	4182	3934	3381
Srcin1	8	11	15	15	13	23	18	12
Srd5a1	304	403	413	337	410	390	375	311
Srd5a2	39	68	55	26	35	58	45	31
Srd5a3	793	807	775	727	719	722	752	675
Srebf1	3621	4014	4039	3583	3715	4017	3995	3554
Srebf2	6193	6561	7194	7101	6835	6740	6130	6258
Srek1	797	762	898	878	905	842	835	838
Srek1ip1	974	930	964	885	1012	1022	978	969
Srf	2354	2297	2502	2333	2414	2380	2308	2433
Srfbp1	250	315	230	236	304	278	228	261
Srgap1	80	142	100	85	54	77	120	72
Srgap2	371	449	424	424	406	403	311	288

Transcriptome sequencing yielded total genetic results for the MOD and APS groups, with a total of 15,936 variables

Continued from above

Srgap3	26	44	44	30	43	42	42	27
Srgn	566	501	550	552	474	493	476	425
Sri	9707	9325	9932	9706	9090	8959	8836	9159
Srl	163	234	198	178	203	239	219	190
Srm	603	616	479	505	801	636	532	597
Srms	11	14	10	10	8	4	8	15
Srp14	1252	1279	1300	1314	1349	1309	1220	1288
Srp19	857	924	870	914	1014	990	978	960
Srp54a	176	262	253	266	282	128	270	77
Srp54b	66	390	373	35	22	26	29	26
Srp54c	747	611	567	558	690	767	668	594
Srp68	3296	3421	3177	3410	3701	3643	3390	3305
Srp72	4029	4330	3902	3820	4583	4471	4241	4083
Srp9	2876	2867	2708	2701	3030	3100	2852	2801
Srpk1	2124	2336	2133	2050	2263	2375	2526	2247
Srpk2	288	266	219	263	257	317	282	227
Srpk3	0	2	2	1	2	2	1	0
Srpr	4990	5289	4961	5093	5374	5211	5291	5279
Srprb	1570	1616	1563	1513	1974	1901	1624	1649
Srpx	60	85	83	70	65	108	47	58
Srpx2	196	218	264	186	230	201	164	203
Srr	937	1099	915	923	935	985	1016	976
Srrd	210	232	220	203	250	241	235	197
Srrm1	4585	4921	4538	4580	4399	4324	4172	4142
Srrm2	8750	9874	9868	9735	9832	9780	9354	8431
Srrm3	9	0	2	10	3	17	4	1
Srrm4	10	6	3	4	3	9	7	2
Srrt	2519	2810	2886	2615	2684	2735	2455	2558
Srsf1	6975	7516	7265	6952	8076	8120	7624	7073
Srsf10	2043	2309	2057	2111	2577	2546	2214	2199
Srsf11	3044	2983	3081	3143	3380	3313	2942	2952
Srsf12	1	1	5	1	1	2	1	3
Srsf2	9214	9484	9228	9463	9068	8974	8123	8834
Srsf3	5953	6333	6273	5901	6092	6259	5798	5871
Srsf4	2328	2431	2384	2262	2112	2299	2141	2097
Srsf5	6737	7375	6579	6674	5212	6253	6300	5548
Srsf6	4882	5020	4880	4810	4892	5030	4458	4817
Srsf7	2575	2755	2540	2544	3023	2793	2481	2494
Srsf9	923	980	992	860	1091	1006	979	992
Srxn1	11856	13023	11444	11580	11841	12775	12631	11990
Ss18	1538	1593	1609	1545	1510	1663	1598	1574
Ss18l1	167	221	245	191	256	294	209	271
Ss18l2	518	514	511	534	556	583	520	527
Ssb	2427	2593	2345	2215	2779	2785	2685	2404
Ssbp1	566	648	616	550	622	595	564	513
Ssbp2	88	64	95	91	93	98	86	77
Ssbp3	1231	1260	1255	1174	1182	1161	949	1078
Ssbp4	1241	1238	1327	1236	1109	1238	1144	1117
Ssc4d	74	85	83	101	69	44	66	80
Ssc5d	174	207	243	184	177	217	131	158
Ssh1	399	408	430	450	644	547	477	443
Ssh2	1430	1732	1736	1581	1621	1640	1547	1495
Ssh3	2073	2378	2311	2431	2315	2320	2041	2052
Ssna1	790	805	761	814	765	780	646	656
Sspn	115	89	110	97	114	68	132	107
Sspo	20	14	20	13	10	11	13	18
Ssr1	6988	7817	7433	6777	7942	7880	7354	7366
Ssr2	7304	7707	7655	7496	8077	7824	7010	7357

Transcriptome sequencing yielded total genetic results for the MOD and APS groups, with a total of 15,936 variables

Continued from above

Ssr3	10481	11764	10837	10795	12801	13041	12209	12343
Ssr4	4761	4936	5119	4953	5003	5347	4805	5098
Ssrp1	3002	3419	3134	2980	3456	3124	2813	2902
Sst	823	908	824	765	657	718	720	599
Sstr1	51	71	119	104	54	98	126	87
Sstr2	9	8	11	19	0	7	15	6
Sstr3	3	2	6	2	0	1	1	3
Sstr4	1	2	4	2	14	0	1	2
Sstr5	9	14	14	31	9	8	10	10
Ssu2	9	12	9	9	17	2	7	8
Ssu72	2561	3050	2933	2734	2968	3107	2792	2716
Ssx2ip	3076	3356	3155	3184	3352	3465	3487	3172
St13	2527	2819	2914	2688	3501	3379	3034	2974
St14	15791	16969	15196	14834	16027	16012	15664	15809
St18	18	23	26	27	38	21	7	27
St3gal1	416	385	330	313	413	375	384	316
St3gal2	135	170	175	130	138	196	181	133
St3gal3	192	233	211	202	183	141	199	128
St3gal4	50776	52449	48614	47028	40433	46120	47950	44303
St3gal5	25	23	26	44	30	41	24	25
St3gal6	2071	1943	2008	2163	1925	2032	1875	1860
St6gal1	429	408	394	368	355	345	320	294
St6gal2	0	5	0	0	0	0	0	0
St6galnac1	0	3	1	0	1	0	0	0
St6galnac2	1370	1502	1594	1456	1666	1583	1456	1514
St6galnac3	11	17	36	13	19	19	25	19
St6galnac4	121	131	108	103	91	80	108	102
St6galnac5	0	1	0	0	0	0	1	0
St6galnac6	100	111	111	106	142	118	107	126
St7	268	343	289	264	298	297	241	242
St7l	684	715	679	719	681	754	781	666
St8sia1	5	11	3	16	4	16	7	9
St8sia2	5	4	6	7	11	6	5	5
St8sia3	11	18	18	16	27	17	5	4
St8sia4	134	160	166	164	142	162	190	164
St8sia5	0	0	0	0	0	0	0	1
St8sia6	41	24	25	50	37	36	52	37
Stab1	1207	1159	1325	1132	1312	1179	1118	1190
Stab2	7	15	19	29	11	15	11	18
Stac	3	9	11	18	8	17	3	27
Stac2	20	11	8	8	18	14	7	5
Stac3	18	13	7	13	2	1	6	5
Stag1	1347	1360	1360	1423	1455	1577	1344	1185
Stag2	3691	4238	3959	3800	4319	4699	4542	4097
Stag3	2	7	12	6	4	1	5	1
Stam	1186	1261	1235	1156	1356	1374	1196	1308
Stam2	2130	2238	2061	2200	2176	2304	2164	2139
Stambp	907	915	834	885	798	798	732	855
Stambpl1	374	426	388	480	388	432	390	401
Stap1	11	23	18	19	10	13	14	11
Stap2	6794	6953	6865	6582	6653	6832	6628	6407
Star	28	60	65	31	30	26	23	47
Stard10	7020	7177	7342	6934	7338	7295	6466	6704
Stard13	566	531	524	426	552	539	501	552
Stard3	2379	2091	2162	2514	2266	2148	2223	2224
Stard3nl	832	908	842	825	893	865	819	780
Stard4	6217	6692	6737	7136	7006	7357	7577	7068
Stard5	541	521	561	593	588	500	468	573

Transcriptome sequencing yielded total genetic results for the MOD and APS groups, with a total of 15,936 variables

Continued from above

Stard6	1	4	2	2	6	2	10	4
Stard7	5681	5824	5810	5642	5362	5199	5054	5023
Stard8	149	84	140	148	136	118	149	84
Stard9	101	140	144	144	106	127	92	89
Stat1	9756	11392	10420	9023	8188	8663	7862	7342
Stat2	4065	4658	4330	3842	3711	3911	3623	3524
Stat3	7777	8522	7463	7479	7795	7686	7605	7359
Stat4	36	23	29	38	24	21	14	22
Stat5a	276	269	258	261	267	204	247	235
Stat5b	752	796	822	836	725	718	718	720
Stat6	6414	6797	6197	5842	6796	6874	6750	6514
Stau1	2214	2323	2109	2134	2059	2277	2106	2146
Stau2	396	485	482	451	466	473	424	411
Stbd1	124	104	105	119	109	99	125	144
Stc1	28	21	21	36	23	42	30	32
Stc2	68	52	110	86	79	70	49	70
Steap1	271	332	295	293	357	352	379	342
Steap2	686	695	629	673	646	557	680	643
Steap3	283	296	281	264	253	264	248	199
Steap4	83	101	47	61	115	75	145	94
Stil	217	272	255	241	228	266	220	243
Stim1	1423	1448	1307	1328	1554	1322	1191	1371
Stim2	1537	1622	1703	1516	1388	1496	1381	1428
Stimate	276	297	314	318	284	259	251	249
Sting1	306	351	281	365	351	289	341	338
Stip1	2971	3546	3317	3175	4811	4656	3536	3341
Stk10	966	1216	869	691	1175	1522	1661	1413
Stk11	3550	3760	3705	3542	3724	3725	3609	3473
Stk11ip	464	515	577	565	540	529	514	503
Stk16	3322	3152	3049	3420	3454	3376	3213	3255
Stk17b	2493	2715	2887	2685	3547	3661	3882	3539
Stk19	761	856	740	763	695	735	788	772
Stk24	6889	7591	6702	6528	6647	7146	6888	6687
Stk25	7333	7972	7168	6790	7275	7806	7316	7242
Stk26	13	24	28	7	42	20	27	33
Stk3	425	474	418	457	426	489	461	413
Stk31	1	0	0	0	0	0	1	0
Stk32a	3	2	6	6	2	9	1	2
Stk32c	127	75	109	163	109	71	98	138
Stk33	0	0	0	0	2	4	0	0
Stk35	872	1071	1169	1089	1227	1154	1088	964
Stk36	1	8	6	9	14	6	9	12
Stk38	3066	3542	3395	3267	3879	3636	3403	3493
Stk38l	910	976	982	974	867	977	792	870
Stk39	1767	1971	2099	1849	1987	1978	1894	1730
Stk4	2235	2198	2076	2075	2252	2204	2108	1983
Stk40	2041	2227	2141	2221	1956	2115	1944	1847
Stkld1	2	5	12	7	10	8	7	8
Stmn1	1652	1783	1910	1824	1901	1676	1565	1465
Stmn2	123	131	95	112	121	77	113	82
Stmn3	32	50	52	50	33	44	50	81
Stmn4	3	2	20	9	1	1	2	5
Stmnd1	4	1	7	6	14	11	5	4
Stn1	459	316	442	547	277	308	256	271
Stom	9277	9810	7961	7734	8081	8211	9223	8262
Stoml1	454	525	444	517	488	407	417	462
Stoml2	1970	2178	2119	2084	2221	1977	1983	2091
Stoml3	7	0	1	0	0	0	0	0

Transcriptome sequencing yielded total genetic results for the MOD and APS groups, with a total of 15,936 variables

Continued from above

Ston1	434	463	574	535	417	501	456	484
Ston2	1346	1521	1451	1397	1515	1608	1492	1310
Stox1	3	28	10	4	8	19	2	10
Stox2	76	112	135	132	147	110	82	127
Stpg1	6	12	7	2	12	5	3	14
Stpg3	0	0	0	0	0	0	1	0
Stpg4	5	0	1	6	0	0	0	5
Stra6	12	11	1	8	4	6	4	2
Stra6l	181	241	208	231	162	217	183	172
Stra8	0	1	0	0	0	0	0	0
Strada	156	166	135	129	169	148	138	149
Stradb	919	1010	958	953	942	942	999	944
Strap	3818	4361	3994	4005	4267	4400	3976	4162
Strbp	1546	1672	1545	1616	1807	1692	1747	1660
Strc	22	35	22	46	11	21	14	20
Strip1	2064	2138	2064	2069	1966	1942	2073	1828
Strip2	23	35	48	25	36	16	31	27
Strn	2046	2242	1945	2090	2124	2314	2366	2199
Strn3	1355	1497	1358	1380	1603	1599	1527	1441
Strn4	2177	2342	2338	2294	2295	2246	2160	2097
Sts	69	93	97	118	85	106	95	121
Stt3a	7887	9073	8419	7938	9431	9353	8715	8689
Stt3b	9138	9679	9678	9204	10652	10682	9509	9241
Stub1	2752	2590	2706	2547	2471	2513	2349	2383
Stum	236	227	190	203	172	210	167	199
Stx11	64	54	78	38	43	45	59	49
Stx12	5648	6194	5390	5418	5734	6374	6114	5891
Stx16	1180	1463	1427	1379	1517	1380	1411	1253
Stx17	924	1042	1132	1133	904	943	910	905
Stx18	680	699	668	621	725	709	646	698
Stx19	0	0	0	0	0	1	2	0
Stx1a	80	104	103	94	98	84	66	94
Stx1b	16	7	6	15	15	8	23	11
Stx2	133	143	124	119	149	137	96	89
Stx3	3884	4184	3778	3826	3687	3775	4037	3573
Stx4a	1576	1692	1685	1589	1482	1580	1432	1497
Stx5a	2869	2894	2598	2604	2898	2942	2952	2971
Stx6	889	1085	1054	1005	1005	888	1009	918
Stx7	5093	5847	5173	5109	5214	5485	5494	5439
Stx8	845	944	845	951	951	981	869	886
Stxbp1	493	483	466	604	407	407	469	368
Stxbp2	6050	6289	6047	5771	5416	5911	5744	5622
Stxbp3	2288	2690	2302	2359	2282	2405	2460	2114
Stxbp4	89	63	127	107	113	90	64	88
Stxbp5	1583	1846	1821	1712	1790	1846	1822	1718
Stxbp5l	38	26	16	20	35	22	23	23
Stxbp6	175	221	175	192	199	216	132	212
Styk1	3097	3677	3216	2897	3513	3765	3478	3250
Styx	188	175	207	184	243	209	191	187
Styxl1	78	52	50	45	58	67	51	55
Sub1	1945	2209	2115	2035	2090	2307	2256	2066
Sucla2	7563	7611	7102	7037	7528	8141	8340	7545
Suclg1	11971	12372	11648	11976	11285	12286	11394	10993
Suclg2	5164	5547	5275	4984	5238	5439	5008	4860
Sucnr1	8	0	1	1	2	0	0	1
Suco	1963	2188	1866	1657	2161	2327	2518	2244
Suds3	4168	4774	4406	4073	4605	4731	4536	4528
Sufu	437	409	429	390	474	361	455	408

Transcriptome sequencing yielded total genetic results for the MOD and APS groups, with a total of 15,936 variables

Continued from above

Sugct	110	149	140	143	122	154	150	144
Sugp1	1131	1173	1285	1144	1105	1124	997	1030
Sugp2	417	451	466	476	457	451	466	421
Sugt1	1876	1951	1897	2057	2191	1955	1921	1904
Sulf1	216	187	187	260	249	181	227	215
Sulf2	7525	7982	7273	7479	7583	7510	7643	7742
Sult1a1	78	74	97	97	89	97	66	110
Sult1b1	13997	15927	14323	13539	14399	16347	17020	14102
Sult1c2	21	29	35	36	34	62	19	25
Sult1d1	9566	11021	9818	8993	11836	13198	13930	11898
Sult2a1	0	0	1	0	0	1	0	0
Sult2a2	4	1	0	0	0	5	0	0
Sult2a5	0	0	0	0	0	0	1	0
Sult2a8	0	0	0	0	5	0	0	1
Sult2b1	7502	7184	6971	7647	6344	6665	6649	6775
Sult4a1	30	38	46	45	31	42	41	14
Sult5a1	5	1	2	7	2	2	15	1
Sult6b1	0	0	4	0	0	0	0	0
Sult6b2	7713	8153	7589	7634	11028	10507	9735	11093
Sumf1	1688	1617	1628	1729	1693	1682	1560	1695
Sumf2	142	126	156	108	178	175	159	196
Sumo1	2794	3057	2864	2788	3188	3357	3218	3099
Sumo2	1392	1533	1328	1390	1486	1630	1301	1300
Sumo3	3381	3898	3666	3258	3755	3585	3422	3307
Sun1	1980	2043	2248	2279	2008	1957	2198	1874
Sun2	392	376	406	430	339	361	361	309
Sun3	17	12	23	25	18	12	8	8
Suox	423	335	431	417	515	514	396	455
Supt16	1697	1975	1961	1782	1956	1999	1864	1783
Supt20	949	999	1028	975	1048	1074	1012	1021
Supt3	81	82	86	64	88	81	75	99
Supt4a	867	852	883	868	843	969	815	796
Supt4b	3	3	1	0	5	0	1	4
Supt5	3538	3982	3877	3474	4172	3985	3760	3670
Supt6	4125	4482	4153	3980	4428	4695	4200	3805
Supt7l	559	626	577	590	605	553	529	605
Supv3l1	1235	1145	1148	1082	1080	1082	986	1107
Surf1	1245	1280	1328	1365	1323	1375	1226	1294
Surf2	241	300	288	266	235	266	213	240
Surf4	33850	35420	34090	33317	34071	34812	33717	32574
Surf6	548	545	598	564	582	561	548	446
Susd1	98	122	138	128	108	104	84	81
Susd2	2122	2705	2080	1883	2014	2296	2631	2443
Susd3	12	15	2	11	16	15	4	2
Susd4	5	2	5	0	7	7	14	4
Susd5	0	8	0	0	6	2	1	1
Susd6	5637	5983	5244	5182	5829	6390	6322	5611
Suv39h1	917	1001	1034	819	969	978	921	836
Suv39h2	138	141	119	151	167	164	132	171
Suz12	3583	4018	3598	3729	3684	3719	3753	3305
Sv2a	23	14	22	14	9	20	38	24
Sv2b	14	15	9	9	14	7	21	6
Sv2c	20	28	27	33	15	33	20	19
Sval1	15	10	6	35	23	25	19	23
Sval3	0	0	0	0	0	1	0	0
Svbp	414	370	314	323	427	394	329	368
Svep1	268	379	296	366	278	328	295	250
Svil	1338	1744	1646	1576	1412	1527	1456	1352

Transcriptome sequencing yielded total genetic results for the MOD and APS groups, with a total of 15,936 variables

Continued from above

Svip	965	1032	1041	1168	1059	1033	910	938
Svop	1	11	3	14	10	7	20	4
Svopl	0	3	1	8	0	0	1	0
Swap70	619	649	672	620	684	697	609	522
Swi5	2701	2927	2946	2909	3105	3001	2738	2616
Swsap1	291	314	350	256	263	251	316	274
Swt1	1161	1241	1183	1065	1203	1384	1280	1210
Syap1	3350	3680	3385	3230	3551	3798	3801	3634
Sybu	52	21	41	49	58	41	40	53
Syce1	0	2	0	0	1	0	15	2
Syce1l	1	0	1	0	0	0	1	0
Syce2	141	89	105	86	95	97	97	128
Syce3	20	20	46	31	13	36	20	29
Sycn	331	463	253	93	2246	2150	1459	1800
Sycp2	4	4	0	0	0	5	0	0
Sycp2l	0	4	1	2	6	1	2	9
Sycp3	19	8	35	13	14	17	18	19
Syde1	180	243	231	151	176	241	167	184
Syde2	637	703	701	680	684	717	534	593
Syf2	1817	1923	1626	1807	1724	1661	1627	1632
Syk	728	714	686	758	865	728	621	694
Sympk	3653	3996	3854	3621	4000	4093	3601	3682
Syn1	28	9	27	34	29	17	31	13
Syn2	115	108	97	104	90	118	82	50
Syn3	14	5	18	18	15	16	25	17
Syna	325	294	298	263	276	262	246	253
Sync	45	19	37	37	17	41	21	30
Syncrip	3170	3506	3140	3199	3356	3391	3344	3286
Syndig1	1	12	15	1	7	8	2	9
Syne1	346	483	542	485	477	475	421	386
Syne2	2763	3169	2934	2996	2798	2947	2654	2434
Syne3	228	301	312	231	263	293	275	242
Syne4	38	84	91	43	55	53	49	73
Syngap1	60	54	98	71	89	62	71	51
Syngr1	119	133	107	98	171	114	113	129
Syngr2	4773	4858	4978	4905	4735	4556	4353	4586
Syngr3	23	19	27	40	16	23	33	32
Syngr4	2	6	0	3	4	1	12	4
Synj1	1630	1577	1603	1557	1519	1677	1588	1702
Synj2	381	409	453	421	430	467	373	421
Synj2bp	3507	3631	3373	3708	3746	3964	4208	3828
Synm	811	791	919	859	764	807	893	752
Synpo	6707	7190	6210	5881	6931	7092	7794	6588
Synpo2	729	848	885	867	780	814	801	827
Synpo2l	7	4	4	2	3	14	6	3
Synpr	0	0	0	9	0	1	7	4
Synrg	1709	1711	1654	1560	1746	1765	1885	1637
Syp	99	148	159	111	112	117	135	114
Sypl	5334	5865	5515	5464	5707	5763	5531	5534
Sypl2	38	55	59	43	37	74	35	33
Sys1	2322	2350	2263	2162	2350	2183	2136	1956
Syt1	64	84	67	64	43	44	58	56
Syt10	0	0	1	0	0	0	0	0
Syt11	110	113	125	103	101	101	118	96
Syt12	401	366	323	413	273	299	276	339
Syt13	137	123	134	155	125	123	160	115
Syt14	16	20	22	15	29	24	8	36
Syt15	63	35	20	33	48	29	39	13

Transcriptome sequencing yielded total genetic results for the MOD and APS groups, with a total of 15,936 variables

Continued from above

Syt16	4	10	2	1	3	3	1	6
Syt17	9	18	23	34	28	31	18	20
Syt2	10	13	11	6	14	21	22	16
Syt3	7	4	3	9	1	1	2	5
Syt4	22	26	46	20	24	21	34	35
Syt5	64	100	84	63	107	109	98	88
Syt6	4	2	1	0	4	6	5	1
Syt7	298	285	313	350	297	211	244	271
Syt8	21	20	26	14	29	21	14	44
Syt9	17	14	16	15	7	6	20	20
Sytl1	122	99	126	166	115	119	121	136
Sytl2	722	716	783	780	858	886	850	727
Sytl3	228	271	248	218	243	204	243	213
Sytl4	113	122	146	105	128	116	122	144
Sytl5	152	177	154	199	155	140	183	154
Syvn1	2287	2502	2249	2296	3145	2669	2333	2641
Szrd1	4093	4472	4183	4163	4302	4351	4259	4122
Szt2	1516	1625	1538	1584	1537	1656	1517	1612
T2	0	0	0	1	0	2	0	2
Taar1	1	0	0	0	0	0	0	1
Taar2	0	0	0	0	0	0	0	6
Taar8b	1	1	0	1	1	0	0	0
Taar8c	0	0	1	0	0	1	1	1
Taar9	0	1	1	1	0	0	0	5
Tab1	1187	1261	1023	1155	991	1105	1043	932
Tab2	3602	3873	3472	3668	3510	3857	3764	3623
Tab3	1204	1246	1175	1086	1283	1273	1216	1210
Tac1	273	303	324	298	339	265	253	292
Tac2	4	1	0	0	0	1	0	0
Tacc1	462	374	480	457	441	356	439	359
Tacc2	1602	1835	1727	1786	1786	1642	1603	1617
Tacc3	955	956	913	902	917	830	762	820
Taco1	1179	1315	1210	1216	1241	1272	1185	1154
Tacr1	93	99	115	97	64	93	118	85
Tacr2	23	6	7	10	2	3	7	2
Tacr3	12	1	6	3	4	4	4	1
Tacstd2	0	4	1	0	0	4	1	0
Tada1	557	671	586	593	659	699	577	649
Tada2a	127	157	138	160	169	178	189	150
Tada2b	890	1080	1075	1138	1028	1083	1070	990
Tada3	739	677	736	634	727	803	667	711
Taf1	1267	1340	1365	1412	1356	1470	1395	1316
Taf10	2976	3010	3155	2850	3187	2875	2816	2756
Taf11	488	524	587	525	570	568	500	425
Taf12	853	880	837	753	979	1010	881	904
Taf13	569	593	574	578	556	709	653	548
Taf15	3455	3710	3639	3377	3601	3937	3691	3557
Taf1a	170	207	174	177	180	193	176	174
Taf1b	158	226	183	161	129	132	162	164
Taf1c	410	517	461	474	544	495	420	477
Taf1d	739	757	789	766	852	848	807	846
Taf2	1363	1435	1293	1439	1305	1351	1335	1318
Taf3	285	373	384	322	345	369	254	298
Taf4	934	1077	1122	1084	1110	921	1009	907
Taf4b	146	169	166	130	186	189	155	144
Taf5	454	485	452	457	486	518	419	410
Taf5l	1638	1625	1545	1638	1541	1530	1390	1510
Taf6	969	1083	1004	1037	1169	1182	913	1025

Transcriptome sequencing yielded total genetic results for the MOD and APS groups, with a total of 15,936 variables

Continued from above

Taf6l	186	218	197	204	235	214	205	214
Taf7	243	316	305	264	243	304	274	266
Taf8	577	593	708	630	605	533	595	495
Taf9	1004	1090	1051	1052	1214	1086	1010	1044
Taf9b	89	93	80	61	59	95	41	60
Tafa1	5	3	5	5	8	4	1	4
Tafa2	0	2	0	1	0	2	1	0
Tafa3	58	54	48	51	36	85	39	40
Tafa5	14	28	35	23	20	34	34	12
Tagap	115	102	81	90	71	68	60	63
Tagap1	298	397	328	380	374	366	306	316
Tagln	4229	4275	4978	4951	4229	4628	4797	4796
Tagln2	4784	5121	5165	5427	5363	4933	4276	4533
Tagln3	18	3	5	7	1	3	4	2
Tal1	19	22	6	15	26	30	7	15
Tal2	1	0	0	0	0	1	6	0
Taldo1	16692	17796	17373	16181	17321	17221	16309	16028
Tamm41	755	726	634	697	616	615	463	585
Tanc1	1168	1272	1310	1293	1126	1198	1158	1017
Tanc2	129	127	122	187	127	160	130	126
Tango2	1372	1463	1444	1465	1393	1463	1574	1353
Tango6	169	201	174	179	141	146	167	165
Tank	1574	1693	1513	1458	1648	1685	1630	1432
Taok1	2518	2674	2484	2745	2778	3109	2982	2480
Taok2	1965	2182	2213	2196	2153	2172	2096	1998
Taok3	4302	4535	4367	3962	4759	4874	4522	4168
Tap1	9511	10322	9420	8901	7325	7484	6970	6885
Tap2	5171	5210	4850	4625	4403	4280	3974	3620
Tapbp	14046	15177	13482	13003	11901	12348	11851	11693
Tapbpl	2509	2533	2347	2577	2144	2172	2223	2011
Tapt1	1335	1435	1274	1369	1376	1400	1339	1374
Tarbp1	172	179	153	156	144	167	135	149
Tarbp2	1682	1725	1655	1703	1742	1803	1644	1592
Tardbp	5247	5518	5343	5143	6384	6467	5749	5409
Tarm1	3	1	0	2	4	0	2	1
Tars	4948	4869	4841	5038	4898	4846	4731	4485
Tars2	1301	1349	1291	1267	1377	1278	1170	1219
Tarsl2	209	197	210	185	189	224	156	221
Tas1r1	0	0	0	1	0	5	0	1
Tas1r2	12	12	11	0	4	0	4	7
Tas1r3	76	53	29	51	32	24	16	48
Tas2r135	0	1	0	0	0	0	0	0
Tas2r138	0	0	0	0	0	0	1	0
Tasl	15	8	9	16	21	7	17	11
Tasor	620	598	629	666	706	693	715	600
Tasor2	945	1025	929	876	887	1062	1090	853
Tasp1	112	153	146	188	168	164	177	140
Tat	143	125	121	169	219	250	325	268
Tatdn1	309	279	298	324	337	311	341	345
Tatdn2	1825	1872	1821	1771	1995	1884	1851	2019
Tatdn3	184	174	177	170	199	184	203	191
Tax1bp1	18841	20400	17565	17223	19573	21023	21030	19596
Tax1bp3	2406	2605	2403	2592	2511	2505	2457	2311
Taz	1715	1611	1682	1636	1500	1601	1536	1508
Tbc1d1	2596	2957	2831	2826	2658	2702	2638	2533
Tbc1d10a	581	591	593	644	696	661	647	636
Tbc1d10b	3356	3374	3153	3074	3135	3319	3246	3195
Tbc1d10c	88	91	72	96	107	74	40	37

Transcriptome sequencing yielded total genetic results for the MOD and APS groups, with a total of 15,936 variables

Continued from above

Tbc1d12	259	282	342	326	283	322	291	246
Tbc1d13	6256	5718	5988	6851	5365	5619	5437	5106
Tbc1d14	4425	5002	4272	3948	4674	4582	4308	4053
Tbc1d15	2691	3005	2527	2789	2524	2974	3002	2799
Tbc1d16	214	218	218	214	207	203	229	236
Tbc1d17	890	905	962	856	844	789	788	777
Tbc1d19	60	34	62	56	64	51	47	41
Tbc1d2	321	344	295	331	376	347	317	433
Tbc1d20	5846	5935	5663	5902	5906	6060	6155	5800
Tbc1d21	0	1	0	0	0	0	0	0
Tbc1d22a	2664	2629	2561	2761	2490	2538	2459	2229
Tbc1d22b	2003	1856	1886	1846	1975	2042	2078	1991
Tbc1d23	1533	1514	1464	1559	1217	1481	1326	1360
Tbc1d24	1569	1795	1730	1647	2023	2139	1988	1888
Tbc1d25	181	250	256	244	235	228	192	200
Tbc1d2b	1551	1558	1632	1721	1617	1550	1393	1502
Tbc1d30	517	449	474	485	427	445	427	385
Tbc1d31	348	353	387	362	376	466	356	342
Tbc1d32	463	481	504	602	456	499	519	474
Tbc1d4	257	266	240	234	288	259	215	255
Tbc1d5	835	1032	925	906	922	965	947	801
Tbc1d7	114	141	143	140	100	80	99	111
Tbc1d8	383	402	437	426	403	413	382	376
Tbc1d8b	1737	1837	1657	1824	2255	2307	2483	2067
Tbc1d9	484	568	569	506	543	590	531	475
Tbc1d9b	2173	2314	2163	1894	2297	2220	2138	1846
Tbca	1657	1656	1707	1789	1834	1897	1865	1714
Tbcb	1866	1827	1858	1710	1817	1776	1720	1964
Tbcc	652	689	631	648	624	600	584	525
Tbccd1	561	578	502	542	742	550	527	637
Tbcd	2494	2570	2475	2343	2616	2470	2341	2395
Tbce	648	679	718	738	743	697	606	707
Tbcel	2184	2235	2139	2170	2089	2013	2107	2008
Tbck	820	896	783	713	734	840	824	727
Tbk1	4052	4417	4201	4073	3796	4107	4185	3811
Tbkbp1	146	128	135	129	121	142	93	84
Tbl1x	2294	2493	2451	2245	2271	2156	2086	1996
Tbl1xr1	1826	2288	1847	1945	2408	2414	2305	2233
Tbl2	1187	1188	1214	1108	1406	1325	1296	1203
Tbl3	890	992	905	902	1095	872	836	870
Tbp	577	543	454	487	579	529	493	521
Tbpl1	647	731	690	782	747	731	719	661
Tbr1	1	0	0	5	0	4	2	2
Tbrg1	6311	6280	5536	5380	5906	5958	5396	5573
Tbrg4	3561	3632	3612	3627	3465	3130	3175	3284
Tbx1	23	21	38	16	27	30	20	37
Tbx10	1	1	0	1	1	0	1	0
Tbx15	0	0	6	6	0	0	0	0
Tbx19	0	4	0	0	1	0	1	7
Tbx2	292	332	320	337	226	269	216	204
Tbx20	1	0	0	0	0	2	2	1
Tbx21	8	12	11	12	16	25	9	28
Tbx3	4976	5272	4961	4835	5130	5474	5324	4746
Tbx4	1	1	2	8	2	6	2	6
Tbx6	1	8	8	4	3	2	2	1
Tbxa2r	49	19	42	42	43	36	50	19
Tbxas1	120	98	86	115	132	106	103	109
Tc2n	664	809	695	697	908	1008	914	974

Transcriptome sequencing yielded total genetic results for the MOD and APS groups, with a total of 15,936 variables

Continued from above

Tcaf1	175	207	174	158	198	206	195	181
Tcaf2	259	269	327	303	338	253	210	224
Tcaim	123	137	115	170	201	178	160	192
Tcam1	5	3	1	2	5	5	4	2
Tcap	7	11	0	2	1	4	16	11
Tcea1	2192	2452	2271	2347	2575	2495	2388	2036
Tcea2	11	12	18	17	13	10	7	8
Tcea3	514	456	551	461	500	453	346	389
Tceal1	17	29	12	34	20	17	27	5
Tceal3	8	31	10	13	12	15	13	21
Tceal5	6	15	10	5	4	18	2	2
Tceal6	3	6	4	2	1	1	2	0
Tceal8	61	101	62	80	95	96	91	85
Tceal9	607	629	590	664	625	586	622	652
Tceanc	126	166	122	127	85	99	78	118
Tceanc2	568	696	583	693	682	663	619	528
Tcerg1	1403	1723	1560	1593	1580	1420	1443	1489
Tcerg1l	0	5	1	4	0	5	0	2
Tcf12	2120	2179	2277	2004	2129	2313	2146	1869
Tcf15	8	11	22	18	2	7	10	11
Tcf19	336	468	374	361	422	361	308	269
Tcf20	1739	2072	1793	1741	1858	1932	1887	1683
Tcf21	420	416	403	453	479	443	390	348
Tcf23	6	7	8	11	18	13	13	6
Tcf24	3	7	3	4	5	4	5	7
Tcf25	7647	8294	7414	7417	7873	8103	7827	7545
Tcf3	2565	2501	2533	2583	2254	2316	2177	2122
Tcf4	735	695	628	702	689	634	564	614
Tcf7	168	178	167	189	246	187	137	152
Tcf7l1	56	35	48	32	46	28	67	52
Tcf7l2	1758	1972	1666	1681	1543	1524	1660	1557
Tcfl5	0	0	1	6	0	0	1	1
Tchh	2	1	2	2	15	6	5	2
Tchp	103	113	106	120	107	118	93	142
Tcim	1827	2115	1985	1801	2240	2512	2409	2100
Tcirg1	2211	2289	2207	2194	2392	2186	2056	2033
Tcn2	13412	13694	12549	12973	12514	13081	13603	12209
Tcof1	1162	1431	1304	1203	1354	1190	1136	1172
Tcp1	5277	5573	5706	5475	6391	5895	5437	5350
Tcp10a	1	0	0	0	1	1	0	0
Tcp10b	6	1	2	1	0	0	0	0
Tcp10c	3	0	0	0	0	0	0	0
Tcp11	15	5	13	22	10	6	6	12
Tcp11l1	56	54	32	30	36	30	49	35
Tcp11l2	197	178	208	181	124	154	140	201
Tcp11x2	0	0	0	0	4	1	0	0
Tcta	824	978	900	834	967	912	864	794
Tcte1	0	0	1	0	0	0	0	0
Tcte2	1	5	15	19	19	5	3	12
Tcte3	7	6	0	5	4	3	0	6
Tctex1d2	99	112	75	102	83	109	89	91
Tctex1d4	10	2	5	1	5	2	1	4
Tctn1	226	207	208	169	218	172	172	129
Tctn2	69	98	144	133	109	122	84	68
Tctn3	155	122	136	144	199	132	134	104
Tdg	1666	1953	1890	1804	1767	1807	1577	1672
Tdg-ps	7	3	14	7	5	10	6	4
Tdh	0	1	0	0	16	3	0	11

Transcriptome sequencing yielded total genetic results for the MOD and APS groups, with a total of 15,936 variables

Continued from above

Tdo2	11	10	2	7	4	5	11	9
Tdp1	208	198	207	205	215	215	206	188
Tdp2	2327	2648	2592	2355	2525	2696	2774	2496
Tdpoz3	13	23	1	2	4	8	15	18
Tdpoz9	0	2	1	4	1	1	1	0
Tdrd1	28	29	13	38	41	27	38	43
Tdrd12	20	34	11	23	13	20	27	33
Tdrd3	415	448	457	407	420	369	374	379
Tdrd5	0	3	0	0	0	1	0	0
Tdrd6	0	0	1	0	0	0	0	0
Tdrd7	4951	5465	4821	4376	5436	5625	5558	5118
Tdrkh	2	20	12	12	17	25	14	11
Tdrp	33	45	23	23	33	37	27	25
Tead1	873	1100	1040	953	1225	1194	1025	983
Tead2	77	81	109	87	100	148	80	71
Tead3	1020	991	1061	1115	927	1010	951	887
Tead4	69	66	62	52	72	63	46	29
Tec	491	496	517	524	591	580	584	456
Tecpr1	921	1048	1121	1047	1064	1052	1069	984
Tecpr2	428	431	439	475	441	418	368	377
Tecr	3107	3404	3377	3163	3080	3246	2786	3053
Tecta	0	1	0	0	0	0	0	3
Tedc1	524	503	559	547	518	516	408	464
Tedc2	190	184	221	256	241	276	224	214
Teddm3	6	4	12	4	13	2	9	17
Tef	1958	1842	2027	2389	1733	2039	2223	1901
Tefm	354	391	275	249	297	241	315	273
Tek	148	242	241	214	209	192	196	204
Tekt2	2	1	4	1	5	5	3	2
Tekt3	0	4	0	0	0	0	0	0
Tekt4	1	0	3	4	0	0	4	4
Tekt5	1	0	0	0	4	0	0	1
Telo2	296	232	257	225	265	286	223	210
Ten1	469	476	470	549	484	502	459	434
Tenm1	1	0	0	1	1	0	0	1
Tenm2	0	0	1	1	0	0	0	0
Tenm3	84	89	85	70	102	51	62	71
Tenm4	18	6	42	24	20	30	13	32
Tent2	1964	2188	1886	1820	1885	2061	1857	1800
Tent4a	625	695	824	699	710	773	615	635
Tent4b	2435	2630	2473	2354	2869	2842	2895	2490
Tent5a	3098	4250	3955	3054	3111	3244	3528	3035
Tent5b	32	35	88	67	53	56	59	55
Tent5c	555	704	544	680	608	609	579	591
Tep1	7557	8300	7323	7406	7493	7701	7761	6970
Tepp	4	10	2	8	1	3	0	3
Tepsin	2258	2349	2133	2258	2529	2231	2103	2168
Terb1	0	0	1	0	4	0	0	1
Terb2	0	1	0	0	0	0	0	0
Terf1	378	369	332	347	332	358	350	278
Terf2	563	603	601	669	632	677	602	536
Terf2ip	639	669	642	681	719	708	668	659
Tert	49	24	64	35	90	76	76	58
Tes	5518	5726	5444	5747	5474	5612	5309	5503
Tesc	30	9	24	31	14	24	21	22
Tescl	0	0	0	1	0	0	0	0
Tesk1	2058	1952	2060	1995	2099	1861	1913	1851
Tesk2	1385	1480	1704	1417	1590	1584	1583	1461

Transcriptome sequencing yielded total genetic results for the MOD and APS groups, with a total of 15,936 variables

Continued from above

Tesl1	0	0	0	0	5	0	4	0
Tesmin	10	16	2	3	5	2	6	6
Tespa1	2	7	0	1	1	5	1	5
Tet1	5	18	28	14	18	10	16	15
Tet2	541	713	548	724	514	592	550	462
Tet3	3834	4414	4343	4385	3939	4139	3882	3568
Tex10	493	568	550	489	666	663	578	578
Tex11	0	1	1	0	0	0	0	0
Tex12	90	91	72	70	49	82	106	85
Tex13c1	0	0	0	0	0	0	0	6
Tex14	17	21	11	19	18	33	19	11
Tex15	1	0	2	6	1	1	0	4
Tex19.2	5	0	2	1	3	0	18	0
Tex2	2620	2936	2836	2779	3318	3325	3143	2948
Tex21	0	0	1	1	2	1	0	0
Tex22	0	0	1	0	0	0	0	0
Tex24	0	0	0	0	0	0	1	0
Tex261	4453	4853	4738	4408	4561	4500	4183	4183
Tex264	1635	1556	1599	1569	1585	1569	1387	1446
Tex29	0	0	0	0	0	0	1	0
Tex30	313	377	310	322	329	421	340	371
Tex38	28	31	34	35	29	26	26	46
Tex45	2	1	0	1	2	0	1	1
Tex52	0	0	0	1	0	0	0	1
Tex9	37	57	59	49	40	46	50	33
Tfam	1852	2078	2087	2052	2061	2078	1915	1918
Tfap2a	7	0	0	2	0	6	0	0
Tfap4	316	426	372	274	334	306	283	373
Tfb1m	159	199	199	147	185	167	206	127
Tfb2m	668	643	657	642	653	691	669	642
Tfcp2	181	182	154	169	175	188	184	155
Tfcp2l1	688	795	947	1000	849	852	1075	883
Tfdp1	2702	3274	2864	2715	2923	2851	2709	2620
Tfdp2	1798	2021	1848	1941	1889	2008	1840	1764
Tfe3	1402	1615	1546	1540	1606	1539	1579	1435
Tfeb	730	676	630	703	635	711	610	657
Tfec	10	32	20	21	32	20	35	10
Tff1	1	1	0	2	0	0	0	0
Tff2	58	89	40	12	318	364	234	282
Tff3	29258	29551	31945	32785	27141	28910	28011	26747
Tfg	15043	17374	15415	14757	16288	16992	17127	15434
Tfip11	1987	2125	1854	1822	1809	2017	1849	1853
Tfpi	122	96	135	120	122	134	127	109
Tfpi2	37	47	24	39	42	42	52	48
Tfpt	253	343	288	258	256	277	260	340
Tfr2	5	10	13	13	13	30	19	9
Tfrc	5597	5922	6555	6515	7181	7844	6529	6397
Tg	7	7	20	8	13	10	6	8
Tgds	756	758	731	663	870	766	798	701
Tgfa	1044	1131	980	1095	1064	1095	1092	907
Tgfb1	555	430	481	384	472	507	452	422
Tgfb1i1	458	581	534	500	494	480	509	566
Tgfb2	55	55	58	67	79	52	71	83
Tgfb3	123	136	135	137	151	164	149	151
Tgfbi	13106	10463	11602	14561	7357	7773	8479	7868
Tgfbr1	810	956	938	945	990	952	991	858
Tgfbr2	2377	2598	2436	2508	2525	2381	2162	2324
Tgfbr3	248	224	293	218	281	252	266	191

Transcriptome sequencing yielded total genetic results for the MOD and APS groups, with a total of 15,936 variables

Continued from above

Tgfbr3l	36	30	24	22	39	30	32	32
Tgfbrap1	1367	1451	1332	1330	1373	1343	1306	1179
Tgif1	585	543	736	646	665	622	515	634
Tgif2	82	65	77	63	80	65	70	118
Tgm1	19	20	25	18	30	15	6	13
Tgm2	8584	9102	8595	8192	8396	8694	8354	7713
Tgm3	2	5	1	1	0	1	0	2
Tgm4	12	4	5	7	0	6	6	4
Tgm5	5	2	7	1	12	1	2	10
Tgm7	0	0	4	0	0	0	0	0
Tgoln1	9888	11580	9835	9462	11862	12182	12397	11417
Tgs1	504	552	572	579	617	638	602	512
Tgtp1	445	357	398	428	217	193	178	156
Tgtp2	710	698	629	698	430	439	318	298
Th	9	5	11	14	12	1	9	14
Tha1	1546	1388	1177	1276	1477	1503	1819	1750
Thada	317	413	427	301	378	359	376	357
Thap1	249	290	268	215	388	325	279	318
Thap11	727	669	664	679	810	756	755	738
Thap12	1632	1640	1532	1663	1758	1814	1607	1522
Thap2	557	589	543	628	870	761	722	653
Thap3	181	209	242	170	236	215	211	189
Thap4	2327	2264	2195	2212	2323	2195	2143	2176
Thap7	620	630	653	572	554	635	545	608
Thbd	579	568	537	575	604	515	520	594
Thbs1	1341	1390	1467	1362	1105	1142	1102	1147
Thbs2	152	135	174	185	158	141	156	133
Thbs3	119	165	146	153	139	124	163	133
Thbs4	7	20	5	20	23	0	16	2
Thegl	26	14	27	7	21	24	39	28
Them4	565	667	621	660	687	663	627	609
Them6	109	101	117	107	144	123	106	85
Them7	1	0	0	0	0	0	0	0
Themis	8	12	16	11	22	7	11	22
Themis2	285	241	229	225	221	202	158	181
Themis3	4540	4909	3779	3807	4375	4611	4622	4477
Thg1l	238	266	266	243	297	289	213	268
Thnsl1	188	167	168	183	195	182	198	202
Thnsl2	1527	1634	1505	1364	1618	1656	1491	1525
Thoc1	538	634	496	563	615	646	673	582
Thoc2	1446	1658	1540	1614	1789	1700	1881	1486
Thoc2l	282	357	375	367	327	390	351	320
Thoc3	663	763	666	670	657	695	600	628
Thoc5	970	1046	1087	1103	1178	1189	967	911
Thoc6	612	699	683	632	610	630	491	541
Thoc7	1919	2183	2107	2059	2132	2055	2036	1749
Thop1	909	1009	984	836	913	968	840	867
Thpo	288	350	343	237	281	259	280	308
Thra	3668	3692	3554	3294	3405	3313	3527	3030
Thrap3	4759	5266	4942	4951	4777	5000	4680	4389
Thrb	700	679	650	619	661	724	661	549
Thrsp	34	44	54	76	99	119	101	58
Thsd1	27	25	37	44	27	12	22	34
Thsd4	219	201	225	244	166	216	202	170
Thsd7a	28	11	25	23	28	12	16	9
Thsd7b	3	5	1	3	2	5	7	9
Thtpa	463	438	472	492	488	430	445	426
Thumpd1	720	823	845	643	843	761	753	849

Transcriptome sequencing yielded total genetic results for the MOD and APS groups, with a total of 15,936 variables

Continued from above

Thumpd2	22	58	58	92	59	37	68	55
Thumpd3	730	849	733	782	948	786	781	840
Thy1	165	141	160	127	152	189	155	96
Thyn1	548	509	565	560	658	632	444	523
Tia1	680	666	636	643	759	799	715	644
Tial1	1384	1633	1546	1644	1668	1699	1702	1647
Tiam1	294	351	380	356	387	377	382	316
Tiam2	766	765	725	692	708	800	764	624
Ticam1	3376	3076	3340	3148	3105	3099	3141	3006
Ticam2	6	4	5	10	14	22	14	29
Ticrr	293	354	382	283	410	423	333	282
Tie1	284	283	304	314	274	234	295	216
Tifa	7156	7297	6773	6880	6523	6978	7386	7275
Tifab	307	249	236	240	312	305	206	263
Tigar	475	544	539	598	573	480	574	375
Tigd2	362	370	386	304	400	396	352	371
Tigd3	128	91	94	76	125	94	109	90
Tigd4	40	18	28	29	40	29	38	42
Tigd5	57	44	61	43	70	56	44	64
Tigit	40	43	67	41	29	43	29	19
Timd2	0	0	0	0	3	0	0	1
Timd4	68	43	53	84	40	89	47	67
Timeless	303	328	327	239	246	284	213	219
Timm10	491	577	544	582	635	571	534	508
Timm10b	1061	1150	1119	1083	988	1007	939	1020
Timm13	2815	2713	2837	2738	2783	2689	2540	2365
Timm17a	2234	2201	2210	2176	2048	2154	1986	2047
Timm17b	972	1070	1033	1044	1223	1152	995	954
Timm21	650	643	612	596	615	669	713	670
Timm22	1420	1313	1335	1414	1329	1449	1164	1232
Timm23	2220	2201	3049	2419	2281	2234	2013	1936
Timm29	1497	1584	1575	1604	1636	1573	1559	1503
Timm44	2260	2241	2397	2440	2354	2330	2201	2130
Timm50	1653	1825	1715	1617	1894	1799	1524	1604
Timm8a1	737	796	874	684	757	870	757	754
Timm8a2	0	0	4	2	0	5	8	1
Timm8b	2759	2449	2685	2531	2583	2849	2694	2698
Timm9	701	703	699	636	727	733	598	594
Timmdc1	853	924	821	923	977	1034	895	888
Timp1	18	13	8	28	27	25	29	30
Timp2	924	815	887	867	792	770	799	802
Timp3	1066	1136	1199	1027	962	1128	953	932
Timp4	0	1	3	1	2	3	6	5
Tinag	307	301	270	312	280	332	324	306
Tinagl1	2021	1974	1976	2195	2352	2479	2344	2242
Tinf2	886	1102	1085	932	994	1106	977	868
Tiparp	1021	984	991	1021	1525	1543	1369	1238
Tipin	276	295	273	255	291	308	256	251
Tiprl	1034	1105	1172	1100	1141	1299	1018	1042
Tirap	778	828	793	842	841	806	761	757
Tjap1	1612	1401	1472	1565	1562	1535	1472	1486
Tjp1	3422	4024	3758	3703	3917	4235	3916	3572
Tjp2	5910	6180	5850	5608	5590	5611	5494	5265
Tjp3	9622	9938	9972	9702	9924	10116	9226	9069
Tk1	863	971	927	946	922	850	755	715
Tk2	165	166	143	117	194	139	144	150
Tkfc	15379	17128	15426	14316	18838	20354	20431	18342
Tkt	21461	21129	21020	21710	21449	20922	19861	21003

Transcriptome sequencing yielded total genetic results for the MOD and APS groups, with a total of 15,936 variables

Continued from above

Tktl1	0	0	0	0	0	0	6	0
Tlcd1	144	143	93	101	158	118	112	132
Tlcd2	1426	1898	1359	1010	1907	2079	2016	1565
Tlcd3a	395	350	397	352	319	323	268	362
Tlcd3b	56	75	72	55	48	46	66	41
Tlcd4	935	1015	846	944	1093	1111	1190	983
Tlcd5	17	15	7	20	22	15	10	6
Tldc2	728	857	711	871	817	807	800	843
Tle1	2502	2590	2570	2842	2737	2575	2354	2329
Tle2	24	34	23	36	28	45	21	18
Tle3	2255	2303	1980	1921	2146	2208	2521	2171
Tle4	2595	3040	2729	2594	2881	2810	2591	2365
Tle5	21535	20808	21470	21319	19580	20253	19537	19631
Tle6	32	38	33	23	33	42	25	36
Tlk1	2128	2435	2479	2213	2452	2644	2454	2250
Tlk2	883	1044	837	904	946	861	867	805
Tll1	9	36	26	25	28	13	26	18
Tll2	0	0	1	2	1	0	3	0
Tln1	5919	6390	6217	5876	6098	6338	6154	5446
Tln2	434	462	477	473	439	490	362	395
Tlnrd1	3952	4120	4034	3938	4051	4301	3897	3485
Tlr1	495	530	446	452	484	431	422	411
Tlr11	5	2	7	12	6	3	10	2
Tlr12	353	457	400	425	428	382	361	325
Tlr13	183	222	113	194	189	178	208	192
Tlr2	194	184	163	179	151	193	149	143
Tlr3	1875	1998	1777	1878	1805	1822	1681	1599
Tlr5	3	14	5	9	15	18	11	10
Tlr6	22	30	24	24	31	28	35	23
Tlr7	109	111	65	120	84	93	174	139
Tlr8	85	78	42	62	79	51	89	73
Tlr9	69	85	87	94	47	49	66	44
Tlx2	24	24	18	35	25	14	11	16
Tm2d1	809	959	893	900	1004	858	853	859
Tm2d2	1012	1094	1121	1257	1226	1193	1088	1102
Tm2d3	545	583	512	564	615	556	541	583
Tm4sf1	234	280	278	215	195	218	243	218
Tm4sf19	5	9	12	5	7	5	9	4
Tm4sf20	23082	27037	21963	19277	26618	26754	27444	27394
Tm4sf4	2301	2590	2153	2074	3043	2650	2575	2747
Tm4sf5	21883	25219	22728	19441	24779	25877	24289	23703
Tm6sf1	146	185	241	278	209	201	221	167
Tm6sf2	12877	13294	12491	12218	12560	12517	12729	12303
Tm7sf2	883	760	844	810	877	848	769	893
Tm7sf3	2682	3077	2774	2860	2922	2837	2711	2597
Tm9sf1	5644	5638	5364	5505	5813	5517	5146	5078
Tm9sf2	19821	21443	19510	18585	21031	21817	20976	20397
Tm9sf3	14968	16311	15134	14780	17431	17706	17035	16621
Tm9sf4	2707	3071	2699	2739	3004	3044	2852	2727
Tma16	114	92	77	90	130	112	118	95
Tma7	4194	4562	4276	4114	4046	4403	3782	4025
Tma7-ps	346	414	357	349	312	347	312	321
Tmbim1	8942	9334	8304	8237	8553	8477	8506	8273
Tmbim4	3398	3342	3231	3607	3342	3306	3206	3227
Tmbim6	63093	65741	62067	60939	60491	62069	61622	59514
Tmc1	2	6	0	9	1	2	3	5
Tmc3	2	4	2	7	6	12	4	17
Tmc4	9342	10009	9149	9145	8973	9446	9182	9016

Transcriptome sequencing yielded total genetic results for the MOD and APS groups, with a total of 15,936 variables

Continued from above

Tmc5	6771	7599	6428	5770	6595	7200	7270	6400
Tmc6	2577	2811	2904	2735	2850	3054	2650	2670
Tmc7	177	146	175	180	206	255	225	197
Tmc8	744	648	658	726	698	658	676	712
Tmcc1	1650	1868	1767	1531	1885	1849	1924	1632
Tmcc2	122	76	68	119	75	52	64	77
Tmcc3	3746	4094	3433	3488	4201	4564	4351	3975
Tmco1	2805	2949	2883	2872	3059	3023	2842	2849
Tmco3	1634	1636	1635	1787	1623	1610	1631	1672
Tmco4	3410	3252	2806	2796	2735	2710	2596	2443
Tmco6	312	348	321	332	383	304	325	285
Tmed1	233	280	285	185	246	263	182	249
Tmed10	7177	7608	7414	7066	7690	7441	7199	7176
Tmed10-p	398	377	330	344	400	402	346	328
Tmed11	0	9	1	0	16	20	6	10
Tmed2	8259	8618	8055	8107	8928	8759	8682	8478
Tmed3	1258	1288	1151	1225	1332	1367	1330	1316
Tmed4	7945	8640	7740	7521	7717	8197	7931	7718
Tmed5	1044	1125	1026	1015	1279	1244	1251	1210
Tmed6	95	62	88	55	158	140	133	119
Tmed7	10302	11281	10243	10295	11306	11519	11008	10320
Tmed8	157	198	168	155	155	133	135	117
Tmed9	2191	2380	2300	2291	2561	2491	2251	2270
Tmeff1	116	86	94	87	90	128	58	72
Tmeff2	6	8	5	3	1	7	2	3
Tmem100	16	18	18	38	35	23	29	26
Tmem101	243	311	308	280	275	261	240	311
Tmem102	1941	2032	1779	1835	1852	2004	1794	1841
Tmem104	630	679	650	590	567	566	568	631
Tmem106	3480	3627	3318	3104	3494	3562	3500	3629
Tmem106	1685	1650	1666	1624	1753	1708	1763	1674
Tmem106	179	188	204	217	217	178	175	203
Tmem107	98	99	115	100	127	85	106	101
Tmem108	6	0	4	13	5	0	1	12
Tmem109	582	499	628	534	585	609	569	492
Tmem11	1047	952	991	1042	988	942	960	989
Tmem115	1714	1542	1705	1520	1949	1839	1595	1613
Tmem116	11	16	7	9	5	6	19	13
Tmem117	144	148	109	118	125	133	119	103
Tmem119	288	301	397	352	294	284	298	254
Tmem120	7465	8093	7653	6992	8055	8220	8384	8032
Tmem120	50	32	54	31	71	49	52	55
Tmem121	2	4	7	4	3	7	2	12
Tmem121	0	6	2	2	0	0	4	1
Tmem123	1407	1562	1555	1533	1629	1581	1525	1364
Tmem125	833	885	712	731	883	973	775	797
Tmem126	855	1048	836	915	861	1010	790	875
Tmem126	452	458	423	415	472	567	561	421
Tmem127	4994	5272	4909	4836	5106	5081	5242	4792
Tmem128	1055	977	1042	1096	1139	996	999	1059
Tmem129	1112	1029	1114	1137	1060	1022	1156	1002
Tmem130	12	19	12	15	10	11	30	10
Tmem131	4681	5253	4697	4520	4793	4923	5212	4591
Tmem131	328	355	324	313	288	353	282	268
Tmem132	161	138	167	144	121	178	96	144
Tmem132	1	10	4	6	3	0	1	1
Tmem132	6	0	1	0	8	1	0	1
Tmem132	1	1	7	0	3	0	3	1

Transcriptome sequencing yielded total genetic results for the MOD and APS groups, with a total of 15,936 variables

Continued from above

Tmem132	95	123	85	77	69	96	48	63
Tmem134	2153	2047	1909	1913	1872	1943	1911	1923
Tmem135	3938	4199	4157	3842	4682	4949	4634	4221
Tmem138	127	131	113	139	119	127	123	91
Tmem139	669	926	820	816	983	957	1079	1021
Tmem140	1426	1510	1427	1236	1108	1236	1355	1152
Tmem141	175	144	136	150	111	160	161	184
Tmem143	872	823	891	944	854	932	869	804
Tmem144	450	448	465	413	465	415	462	399
Tmem145	4	1	5	0	1	0	0	1
Tmem147	2374	2450	2384	2479	2377	2248	2093	2354
Tmem14a	120	146	154	127	146	115	165	135
Tmem14c	5462	5649	5693	6020	5531	5702	5142	5128
Tmem150	128	114	112	104	101	102	130	107
Tmem150	2572	3142	2527	2440	2931	2692	2617	2465
Tmem150	5	5	5	3	8	1	2	4
Tmem151	91	75	97	70	50	95	120	77
Tmem151	0	4	3	7	5	14	10	9
Tmem154	130	146	156	180	88	138	100	134
Tmem156	31	33	34	22	29	17	48	18
Tmem158	277	235	232	292	244	228	170	200
Tmem159	440	448	446	403	391	481	415	428
Tmem160	1205	1258	1115	971	1238	1336	1067	1156
Tmem161	1391	1300	1402	1491	1403	1416	1302	1370
Tmem161	1446	1542	1331	1500	1621	1591	1451	1513
Tmem163	2	1	2	5	4	0	3	2
Tmem164	2129	2377	2381	2285	2051	1980	1950	1949
Tmem165	2486	2550	2392	2454	3036	2847	2956	2997
Tmem167	2945	3379	2926	3021	3507	3398	3506	3266
Tmem167	1909	2054	1995	2078	2115	2220	2249	2050
Tmem168	834	871	781	841	865	746	872	799
Tmem169	0	2	1	14	4	5	5	0
Tmem17	23	19	23	20	22	35	23	24
Tmem170	1832	2190	1845	1817	1955	1965	1945	1890
Tmem170	699	643	783	700	662	818	695	675
Tmem171	1318	1387	1260	1404	1457	1448	1174	1219
Tmem174	11	4	12	5	9	13	23	8
Tmem175	425	484	407	455	446	498	484	455
Tmem176	2638	2734	2800	2786	2597	2656	2548	2524
Tmem176	4961	5033	4949	5053	4831	4936	4754	4777
Tmem177	356	363	344	391	296	311	351	324
Tmem178	64	82	72	104	113	81	82	89
Tmem178	4	15	7	11	17	17	8	9
Tmem179	9	9	10	7	17	14	15	14
Tmem179	519	547	569	536	562	572	464	555
Tmem18	841	983	803	903	737	786	749	873
Tmem181	2214	2274	2101	2374	2052	1949	2009	2026
Tmem182	8	13	13	9	7	20	9	14
Tmem183	6694	6911	6609	6542	6522	6876	6729	6347
Tmem184	4886	4930	4870	4698	4948	5355	5267	4819
Tmem184	2451	2717	2491	2317	2608	2534	2592	2578
Tmem184	129	139	138	117	138	134	132	196
Tmem185	522	608	596	515	674	504	462	451
Tmem185	634	697	699	650	637	671	621	646
Tmem186	676	824	759	706	746	809	703	773
Tmem189	1409	1275	1183	1283	1216	1209	1206	1246
Tmem19	4397	4227	4047	4396	3807	4065	4253	3942
Tmem190	2	4	5	4	8	5	0	4

Transcriptome sequencing yielded total genetic results for the MOD and APS groups, with a total of 15,936 variables

Continued from above

Tmem191	65	46	55	56	54	30	47	42
Tmem192	645	626	637	709	686	652	594	543
Tmem196	1	2	4	3	7	1	0	1
Tmem198	24	24	19	21	30	20	21	23
Tmem198	158	93	111	151	110	137	101	115
Tmem199	337	390	362	357	396	360	332	390
Tmem200	8	17	12	17	23	6	6	7
Tmem200	110	109	115	122	123	110	109	108
Tmem200	4	2	0	4	1	3	0	12
Tmem201	339	415	402	420	419	394	349	350
Tmem202	51	23	22	15	43	37	33	32
Tmem203	354	317	259	251	293	271	253	245
Tmem204	181	230	237	190	210	214	207	166
Tmem205	1966	2071	1918	2152	2116	2136	1988	1893
Tmem208	1281	1384	1264	1250	1444	1395	1279	1384
Tmem209	521	591	545	531	544	534	505	447
Tmem210	0	0	0	0	0	1	0	0
Tmem212	4	0	1	0	0	1	0	0
Tmem213	0	3	0	0	0	0	0	0
Tmem214	3047	3111	2960	2755	3340	3426	3080	3192
Tmem215	0	0	0	5	4	1	0	0
Tmem216	70	133	108	104	101	79	113	130
Tmem218	67	97	90	90	67	103	77	41
Tmem219	570	586	559	651	600	612	562	512
Tmem220	331	369	360	348	408	387	463	372
Tmem221	14	17	9	14	39	11	8	11
Tmem222	929	1064	1058	998	1048	981	951	973
Tmem223	877	851	970	755	833	797	817	806
Tmem229	13	14	8	15	5	30	11	16
Tmem229	487	549	528	427	485	497	443	396
Tmem230	806	794	775	764	861	892	767	875
Tmem231	22	16	31	33	29	24	21	15
Tmem234	2743	2961	2942	2876	2539	2760	2586	2522
Tmem235	8	14	3	4	5	2	1	1
Tmem236	5798	7048	5918	5243	5595	6248	6603	5974
Tmem237	77	86	72	76	71	102	95	76
Tmem238	1445	1537	1502	1342	1488	1519	1320	1379
Tmem240	7	2	2	4	8	3	1	0
Tmem241	238	283	292	253	207	202	229	214
Tmem242	1454	1393	1450	1551	1527	1498	1475	1466
Tmem243	1957	2132	1811	1630	1788	1933	1902	1895
Tmem245	2070	2224	1932	2126	2039	2151	2135	1988
Tmem248	1831	2055	1830	1714	2077	1873	1695	1988
Tmem249	0	0	0	0	0	0	0	1
Tmem25	163	134	127	157	193	144	130	112
Tmem250	1942	1829	1802	1988	1922	1858	1768	1918
Tmem251	460	412	465	481	531	498	472	478
Tmem252	808	932	888	853	1122	947	919	1071
Tmem253	2922	2955	2779	2805	2800	2814	2629	2827
Tmem254	1899	2220	2242	2063	2455	2561	2400	1953
Tmem255	6	6	5	7	6	18	7	15
Tmem255	98	78	69	58	73	70	96	90
Tmem256	646	569	710	786	669	683	691	682
Tmem258	2079	2235	2127	2101	2087	2267	1720	2051
Tmem259	5405	5258	5329	4876	4785	4495	4476	4472
Tmem26	20	19	28	26	19	12	7	14
Tmem260	823	924	842	839	1073	985	899	845
Tmem262	19	12	13	10	18	16	6	14

Transcriptome sequencing yielded total genetic results for the MOD and APS groups, with a total of 15,936 variables

Continued from above

Tmem263	1807	1998	1920	1724	1758	2026	1807	1895
Tmem265	185	198	158	178	129	165	145	159
Tmem266	57	45	29	27	43	37	28	45
Tmem267	105	150	124	79	108	139	135	135
Tmem268	898	966	991	866	994	872	844	847
Tmem269	0	1	5	2	1	1	2	5
Tmem273	15	31	31	19	36	51	31	49
Tmem28	4	0	4	1	1	0	2	1
Tmem29	174	246	180	169	199	168	136	193
Tmem30a	5287	5463	5339	5249	5412	5836	6182	5388
Tmem30b	6984	7387	7243	7058	6942	7057	6884	6886
Tmem33	3483	3760	3450	3291	4009	3979	3800	3639
Tmem35a	10	4	4	7	6	14	8	7
Tmem35b	187	194	175	234	240	241	189	156
Tmem37	2776	2905	2951	3080	2636	3088	3237	3141
Tmem38a	318	315	300	355	333	288	314	316
Tmem38b	279	280	303	286	251	264	271	251
Tmem39a	866	938	883	1022	1095	873	861	976
Tmem39b	158	229	185	135	209	139	187	184
Tmem40	1	0	1	0	1	0	1	0
Tmem41a	2974	2993	3164	2836	3080	3155	3236	3032
Tmem41b	2789	3073	3104	2904	3331	3464	3558	3357
Tmem42	133	79	109	91	142	113	124	93
Tmem43	782	901	804	910	939	1017	950	995
Tmem44	39	44	58	38	56	49	19	30
Tmem45a	11	21	13	19	14	15	22	16
Tmem45a	0	0	4	0	0	0	0	0
Tmem45b	13156	14340	12747	12263	12673	13359	13185	11900
Tmem47	97	130	113	94	117	112	114	66
Tmem50a	1939	1916	2037	1982	2042	2027	2105	1932
Tmem50b	4282	4115	4331	4386	3971	4252	3790	3898
Tmem51	2544	2444	2358	2246	2520	2364	2484	2364
Tmem52	5	1	8	12	2	12	4	8
Tmem52b	1	0	0	0	0	0	0	0
Tmem53	154	167	170	119	161	129	119	150
Tmem54	8206	8861	7999	8120	8540	8818	8324	8460
Tmem59	9428	10138	9332	9459	9740	10004	9768	9481
Tmem59l	11	2	8	4	25	16	12	2
Tmem60	304	351	290	302	316	320	334	359
Tmem61	0	0	1	0	0	4	6	4
Tmem62	283	315	305	252	340	355	310	318
Tmem63a	1103	1155	1257	1151	1153	1118	944	1086
Tmem63b	2647	2448	2351	2297	2348	2227	2162	2236
Tmem63c	1	11	8	3	5	4	1	18
Tmem64	787	806	799	791	889	990	1094	907
Tmem65	590	583	632	561	706	688	631	526
Tmem67	66	75	60	60	87	60	78	77
Tmem68	691	700	753	676	785	791	742	743
Tmem69	479	391	472	416	525	474	453	523
Tmem70	1583	1566	1530	1585	1595	1715	1551	1707
Tmem71	30	38	25	47	21	49	34	29
Tmem72	0	0	1	0	4	0	0	0
Tmem74	2	1	0	7	1	2	5	5
Tmem74b	25	5	2	9	2	6	4	6
Tmem79	1497	1548	1623	1631	1806	1757	1490	1572
Tmem80	238	292	277	303	255	262	272	245
Tmem81	121	157	131	116	114	109	124	112
Tmem82	1610	1709	1824	1766	1984	1942	1734	1803

Transcriptome sequencing yielded total genetic results for the MOD and APS groups, with a total of 15,936 variables

Continued from above

Tmem86a	3477	3740	3902	3561	3344	3800	3561	3051
Tmem86b	2062	2227	1971	1730	2216	2182	2505	2437
Tmem87a	1062	1147	1064	1148	1028	1107	1041	990
Tmem87b	3617	3710	3414	3417	3442	3805	3867	3489
Tmem88	74	77	105	87	90	94	76	51
Tmem88b	72	34	42	64	32	19	47	26
Tmem89	107	58	79	80	78	94	90	78
Tmem8b	36	46	52	48	43	34	40	30
Tmem9	268	229	241	223	208	208	163	181
Tmem91	1	0	0	1	0	0	1	0
Tmem92	0	1	1	5	0	1	0	0
Tmem94	3456	3687	3549	3099	3620	3395	3385	3186
Tmem95	9	2	4	4	2	8	3	1
Tmem97	1850	2040	2062	2008	2016	1775	1667	1921
Tmem98	5983	6176	5743	5909	6164	6188	6012	6400
Tmem9b	3799	3915	3969	3758	3834	3966	3631	3929
Tmf1	1653	1649	1588	1595	1742	1593	1770	1646
Tmie	21	23	19	22	23	22	19	22
Tmigd1	186	199	162	114	241	332	505	402
Tmigd3	2	0	9	2	2	8	7	14
Tmlhe	1148	1381	1217	1198	1159	1320	1344	1206
Tmod1	70	75	101	78	89	82	101	56
Tmod2	37	76	60	62	79	75	75	83
Tmod3	4900	5439	5358	5026	5538	5570	5091	5098
Tmod4	8	6	1	7	6	13	14	4
Tmpo	3955	4417	3980	3720	4527	4331	4019	3827
Tmppe	649	689	791	728	699	780	632	674
Tmprss15	4373	5365	3061	2418	5342	5754	6465	5111
Tmprss2	13989	14392	15394	14977	15377	14906	13235	13740
Tmprss3	0	1	1	0	0	1	1	0
Tmprss4	8935	9526	9027	8555	9626	9526	9016	9056
Tmprss5	7	26	23	31	11	5	11	16
Tmprss6	2	5	2	0	1	2	3	6
Tmprss7	11	18	19	16	19	5	11	10
Tmprss9	54	56	37	23	41	49	61	56
Tmsb10	9525	10224	10435	9937	9142	10124	9072	10128
Tmsb15b1	0	4	1	2	7	0	4	1
Tmsb4x	23581	24100	23255	24521	25185	25654	23890	24140
Tmtc1	206	174	205	224	181	168	226	225
Tmtc2	640	770	687	688	751	914	676	693
Tmtc3	761	830	873	712	961	912	826	865
Tmtc4	799	749	802	677	690	729	618	664
Tmub1	820	869	842	655	843	833	833	849
Tmub2	1881	1809	1856	1792	1774	1711	1704	1597
Tmx1	1859	2264	1865	2022	2210	2310	1982	1916
Tmx2	2733	2752	2625	2890	2793	2509	2593	2665
Tmx3	1192	1282	1207	1107	1281	1413	1354	1356
Tmx4	171	208	212	210	220	190	163	135
Tnc	1109	1251	1214	1234	1191	1072	1023	1063
Tnf	116	93	99	132	207	201	144	135
Tnfaip1	7646	7969	7591	7726	7320	7412	7868	7244
Tnfaip2	783	733	646	692	592	572	536	521
Tnfaip3	1473	1732	1650	1618	1893	2053	2012	1910
Tnfaip6	14	12	9	18	16	28	23	25
Tnfaip8	359	335	330	351	332	314	302	291
Tnfaip8l1	323	322	356	357	373	335	370	307
Tnfaip8l2	145	122	121	135	153	123	179	160
Tnfaip8l3	645	707	657	751	602	600	561	519

Transcriptome sequencing yielded total genetic results for the MOD and APS groups, with a total of 15,936 variables

Continued from above

Tnfrsf10b	290	366	343	296	335	283	279	316
Tnfrsf11a	1813	2148	1971	1664	2024	2045	1973	1654
Tnfrsf11b	111	103	93	109	115	112	83	127
Tnfrsf12a	396	377	459	470	376	434	441	470
Tnfrsf13b	108	163	125	153	140	138	157	130
Tnfrsf13c	19	33	6	12	12	10	6	8
Tnfrsf14	2670	2999	2801	2866	2587	2623	2409	2264
Tnfrsf17	46	37	46	36	35	23	13	50
Tnfrsf18	29	24	35	19	30	42	14	21
Tnfrsf19	161	133	127	121	126	181	165	95
Tnfrsf1a	8208	8434	7550	7687	7562	7727	7693	7487
Tnfrsf1b	626	583	645	608	555	531	497	427
Tnfrsf21	1109	907	914	1194	698	640	699	695
Tnfrsf22	24	19	16	14	19	14	8	22
Tnfrsf23	271	282	291	377	228	230	302	278
Tnfrsf25	16	10	7	2	18	22	11	19
Tnfrsf26	15	6	9	11	6	7	11	6
Tnfrsf4	43	39	17	31	25	21	23	15
Tnfrsf8	24	20	27	29	21	48	30	25
Tnfrsf9	18	33	18	33	15	19	11	17
Tnfsf10	4469	5240	4439	4386	4065	4496	4283	3584
Tnfsf11	12	4	4	10	2	8	0	7
Tnfsf12	21	45	33	49	21	26	28	19
Tnfsf13	274	279	312	281	324	312	351	285
Tnfsf13b	317	315	304	308	364	314	210	200
Tnfsf14	5	2	1	2	3	2	4	17
Tnfsf15	12	11	8	25	19	36	13	36
Tnfsf18	0	5	1	9	8	1	5	0
Tnfsf4	0	0	1	0	5	1	2	4
Tnfsf8	19	19	18	22	25	13	9	31
Tnfsf9	17	20	15	21	41	30	19	36
Tnik	2529	2730	2408	2721	2653	2522	2704	2544
Tnip1	5051	5051	4517	4474	4432	4665	5194	4835
Tnip2	166	185	238	215	193	204	247	172
Tnip3	30	26	10	24	19	11	13	9
Tnk1	1369	1350	1415	1382	1256	1253	1376	1320
Tnk2	2103	2267	2240	2059	2297	2192	2145	2133
Tnks	1916	2129	1998	1933	2121	2149	2271	1798
Tnks1bp1	4096	4391	3994	3991	3481	4082	3926	3470
Tnks2	4493	4894	4667	4549	5064	5207	5027	4982
Tnmd	0	0	4	0	1	3	0	0
Tnn	0	1	0	0	0	1	1	0
Tnnc1	0	0	4	5	1	0	4	6
Tnni1	39	43	42	28	46	26	20	18
Tnni2	0	7	2	0	2	5	2	2
Tnni3	4	5	4	0	6	1	0	2
Tnni3k	1	6	0	0	0	0	1	0
Tnnt1	75	52	51	80	37	64	87	59
Tnnt2	96	64	102	78	73	96	104	119
Tnnt3	0	4	3	1	1	1	0	1
Tnpo1	2931	3228	3137	3134	3418	3377	3179	2853
Tnpo2	2171	2582	2432	2336	2462	2516	2136	2173
Tnpo3	2031	2059	2101	2041	2110	2160	2057	2066
Tnr	2	7	2	8	5	2	6	5
Tnrc18	4458	4869	4605	4692	4746	5072	4802	4436
Tnrc6a	2172	2433	2351	2358	2445	2595	2237	2152
Tnrc6b	2007	2188	2268	2171	2213	2385	2280	2135
Tnrc6c	1019	1073	1074	1173	1030	1084	1085	1030

Transcriptome sequencing yielded total genetic results for the MOD and APS groups, with a total of 15,936 variables

Continued from above

Tns1	1380	1390	1534	1562	1429	1437	1361	1270
Tns2	718	687	642	808	597	588	614	519
Tns3	2919	3109	3068	3270	2660	2724	2615	2366
Tns4	1216	1073	1048	1439	961	1012	1086	932
Tnxb	598	635	641	661	697	599	656	507
Tob1	9368	9943	9921	9108	10185	10958	11121	10112
Tob2	2413	2646	2940	2562	2446	2619	2647	2513
Toe1	411	461	409	413	443	437	409	360
Togaram1	778	806	754	774	807	841	790	791
Togaram2	1	2	9	1	0	0	1	1
Tollip	4809	4812	4421	4686	4419	4621	5004	4656
Tom1	365	274	344	306	266	280	253	244
Tom1l1	2909	2961	2686	3361	2717	2663	2785	2607
Tom1l2	5176	5393	4883	5159	4965	5346	6086	5356
Tomm20	3473	3541	3686	3333	3619	3568	3507	3462
Tomm22	4397	4109	4333	4512	4474	4278	3871	4040
Tomm34	687	596	648	671	708	675	652	576
Tomm40	3570	3539	3568	3627	3597	3195	2991	3087
Tomm40l	1706	1636	1606	1697	1596	1420	1401	1503
Tomm5	2022	2077	1800	1869	2219	2083	1825	1935
Tomm6	2157	2224	2326	2137	2385	2166	1946	2155
Tomm7	1781	1791	1791	1732	1853	1928	1721	1766
Tomm70a	6362	7037	6458	6195	7311	7580	7007	6648
Tomt	0	1	2	2	2	2	1	0
Tonsl	1014	1187	1048	1087	965	928	953	961
Top1	3858	4188	3771	3701	4856	5067	4589	4236
Top1mt	239	259	244	184	199	228	190	219
Top2a	4816	5426	5112	4780	5532	5532	4835	4360
Top2b	2801	3036	2909	2915	3302	3523	3127	2865
Top3a	278	366	302	248	329	339	287	340
Top3b	981	1135	985	958	989	1030	898	801
Topaz1	9	2	5	2	5	3	10	14
Topbp1	1293	1627	1577	1435	1491	1580	1467	1550
Topors	1007	1059	999	1056	993	1115	1080	1030
Tor1a	2310	2443	2410	2290	2609	2572	2551	2594
Tor1aip1	1927	2152	2150	1890	1921	1959	1750	1670
Tor1aip2	10676	12445	10672	10247	10729	10975	11381	10403
Tor1b	847	1066	1009	963	1202	1113	973	1088
Tor2a	1982	1887	1944	1891	1889	1753	1718	1702
Tor3a	1025	1367	1265	1019	1081	1163	948	919
Tor4a	1379	1361	1196	1224	1515	1364	1335	1302
Tox	271	290	281	295	326	234	282	258
Tox2	38	26	35	29	32	40	23	21
Tox3	294	336	326	393	328	265	262	260
Tox4	2601	2635	2576	2385	2731	2513	2523	2458
Tpbg	54	70	62	63	60	60	67	45
Tpbgl	50	48	30	42	44	44	63	35
Tpcn1	3378	3597	3359	3127	3505	3492	3708	3591
Tpcn2	591	491	414	492	462	452	408	447
Tpd52	16602	17865	16176	15655	16925	17595	18144	17312
Tpd52l1	18	30	39	25	42	34	40	27
Tpd52l2	1345	1339	1433	1399	1429	1395	1394	1211
Tpgs1	1082	920	1080	921	1080	1047	933	1044
Tpgs2	85	123	142	102	171	139	165	98
Tph1	395	419	416	460	434	388	384	389
Tpi1	39789	40962	39827	40644	40607	41718	41839	40527
Tpk1	1192	1201	1281	1261	1231	1162	1253	1132
Tpm1	14074	14767	14812	14570	14412	15115	14797	14488

Transcriptome sequencing yielded total genetic results for the MOD and APS groups, with a total of 15,936 variables

Continued from above

Tpm2	2726	2960	2949	3158	2735	3099	3354	3024
Tpm3	14686	15496	14564	14190	14379	14319	13294	13649
Tpm4	2981	3389	3478	3297	3634	3532	3156	3243
Tpmt	474	413	443	545	460	475	463	361
Tpp1	1431	1479	1460	1552	1550	1453	1489	1464
Tpp2	2261	2323	2125	2264	2664	2726	2518	2243
Tppp	299	274	312	288	496	598	533	395
Tppp3	301	297	353	396	418	457	434	429
Tpr	2688	2952	2814	2907	2997	3104	3093	2692
Tpra1	1150	1176	1092	1196	1063	1132	1128	1155
Tprgl	2993	3049	2994	2826	3008	3192	2929	2870
Tprkb	1652	1670	1770	1694	1537	1670	1605	1602
Tprn	5519	5670	5036	4800	5006	5386	5211	5041
Tpsb2	2	2	1	3	4	0	9	1
Tpsg1	694	615	659	590	573	525	571	571
Tpst1	202	212	201	182	138	195	160	152
Tpst2	696	719	744	615	577	696	664	642
Tpt1	54923	56961	55776	56492	56170	58693	55069	52788
Tpte	4	4	1	2	7	0	0	0
Tpx2	757	892	908	840	944	833	739	846
Tra2a	1686	1818	1802	1791	2675	2303	1990	2083
Tra2b	3152	3288	3167	3205	3752	3599	3101	3243
Trabd	4797	4838	4682	4628	5346	5169	4684	4967
Trabd2b	31	26	35	12	16	8	29	21
Tradd	4558	4264	4238	4174	4249	4155	4430	4220
Traf1	36	53	58	43	38	76	60	30
Traf2	1078	1072	1119	1119	1129	1088	959	965
Traf3	1089	1179	1147	1076	1119	1213	1180	1157
Traf3ip1	353	286	367	425	340	319	357	272
Traf3ip2	1277	1212	1131	1121	1355	1299	1293	1243
Traf3ip3	49	65	61	47	69	72	61	78
Traf4	2191	2205	2435	2507	2423	2442	2092	2264
Traf5	77	81	94	73	83	61	64	36
Traf6	977	1204	1110	1039	1074	1030	993	861
Traf7	2432	2632	2553	2646	2648	2628	2268	2585
Trafd1	3844	4374	4259	3859	3767	3823	3558	3454
Traip	155	210	220	220	248	201	204	241
Trak1	6038	6036	5944	6747	5739	5489	5598	5349
Trak2	1998	1867	2062	2177	1601	1646	1650	1665
Tram1	4638	5000	4384	4622	5335	5229	5128	4891
Tram1l1	14	12	4	6	4	4	9	5
Tram2	48	56	47	53	88	67	76	58
Trank1	6	2	6	12	4	0	1	2
Trap1	4551	5009	4783	4795	5007	5009	4323	4208
Trappc1	1019	1041	1123	1066	1042	1040	1068	1073
Trappc10	6435	7272	6915	6565	6155	6515	6328	6268
Trappc11	2188	2499	2315	2262	2285	2162	2140	2274
Trappc12	1233	1301	1412	1274	1307	1279	1243	1247
Trappc13	1408	1551	1380	1349	1580	1568	1476	1430
Trappc2	95	124	132	120	151	113	102	118
Trappc2l	1150	1181	1184	1126	1119	1135	1119	1051
Trappc3	2036	2141	2020	2005	1930	2033	1876	1911
Trappc4	1777	1868	1867	1700	1842	1749	1810	1821
Trappc5	1033	1033	1042	1090	1035	1026	994	954
Trappc6a	1030	934	1025	1012	1136	981	929	972
Trappc6b	1182	1301	1130	1138	1174	1229	1289	1111
Trappc8	2984	3371	3169	3280	3766	3462	3716	3233
Trappc9	1429	1525	1553	1489	1446	1411	1404	1283

Transcriptome sequencing yielded total genetic results for the MOD and APS groups, with a total of 15,936 variables

Continued from above

Trarg1	14	28	14	29	36	32	30	24
Trat1	11	4	8	1	4	10	5	2
Trdmt1	56	91	101	134	80	68	55	87
Trdn	1	0	1	0	0	0	4	0
Treh	12345	14040	11543	10530	11210	11862	12877	11543
Trem1	0	2	3	1	8	3	5	4
Trem2	11	16	14	8	13	8	10	5
Trem3	0	0	0	2	4	0	0	1
Treml1	0	0	0	0	0	0	1	0
Treml2	7	6	4	12	9	10	6	3
Treml4	53	62	32	47	42	20	48	36
Trerf1	60	72	65	39	42	44	40	42
Trex1	755	987	949	605	538	689	659	669
Trex2	31	42	22	16	24	33	49	30
Trf	674	1037	893	729	797	828	870	650
Trhde	2	3	3	1	0	2	6	2
Triap1	975	899	991	1054	997	973	844	863
Trib1	1189	1446	1088	1179	1641	1443	1405	1407
Trib2	112	97	136	93	94	106	90	80
Trib3	457	404	450	382	381	370	524	426
Tril	173	167	217	213	198	195	190	151
Trim10	16	14	3	7	13	14	3	6
Trim11	643	744	720	708	819	694	636	630
Trim12a	1933	2511	1976	1662	2296	2365	2219	1775
Trim12c	1302	1717	1354	1345	1580	1713	1801	1456
Trim13	64	91	39	77	64	65	70	52
Trim14	2351	2511	2583	2259	2417	2569	2244	2152
Trim15	2221	2488	2173	2246	1954	2109	2041	2125
Trim16	974	1040	1002	1024	827	965	952	936
Trim17	60	68	81	66	50	48	66	47
Trim2	5026	5530	5593	5240	5329	5305	5280	4845
Trim21	559	552	586	539	555	482	557	446
Trim23	226	218	213	193	209	192	217	242
Trim24	838	881	920	835	823	776	883	891
Trim25	8496	10477	9660	8579	9272	9158	8900	8328
Trim26	2852	3153	3263	2939	2812	2889	2663	2601
Trim27	1109	1065	1122	1128	1310	1214	993	1207
Trim28	3353	3616	3595	3346	3917	3610	3216	3346
Trim29	1	4	1	6	4	6	5	0
Trim3	194	157	156	215	196	138	135	205
Trim30a	2936	4163	3283	2138	3607	3389	3128	2340
Trim30c	25	34	15	18	8	9	27	17
Trim30d	2782	3288	2694	2558	2753	3198	3020	2525
Trim31	7909	8155	7624	7659	8022	8153	7534	7127
Trim32	601	610	641	569	604	827	575	634
Trim33	952	1017	1032	987	1068	1150	1021	927
Trim34a	695	952	1050	757	863	895	825	837
Trim34b	47	58	54	53	50	26	29	38
Trim35	423	400	426	467	436	427	371	392
Trim36	1069	1132	1160	1208	1114	1149	1044	949
Trim37	561	759	731	620	746	716	617	586
Trim38	400	364	445	357	476	440	473	438
Trim39	542	484	575	503	549	590	475	571
Trim40	2376	2593	2276	2198	2256	2469	2470	2293
Trim41	1366	1451	1362	1433	1470	1438	1281	1302
Trim43a	0	0	0	0	0	0	2	0
Trim43c	1	0	1	0	0	1	5	0
Trim44	480	534	479	554	540	605	389	479

Transcriptome sequencing yielded total genetic results for the MOD and APS groups, with a total of 15,936 variables

Continued from above

Trim45	15	26	35	25	18	36	20	11
Trim46	117	137	116	86	177	139	108	158
Trim47	312	262	241	280	247	298	208	244
Trim5	273	310	223	258	207	297	284	249
Trim50	13	8	15	1	32	11	9	11
Trim56	716	847	802	737	789	878	799	684
Trim58	0	0	1	0	1	0	0	0
Trim59	354	416	421	416	461	454	407	331
Trim6	105	100	122	78	107	110	137	86
Trim62	96	97	72	73	73	129	92	81
Trim63	22	17	22	14	6	8	11	18
Trim65	471	514	568	467	647	673	502	587
Trim66	1	0	0	0	5	0	4	2
Trim67	1	0	8	1	1	3	1	12
Trim68	54	66	60	51	94	67	39	84
Trim69	1	1	8	8	13	2	0	4
Trim7	206	193	220	283	247	260	232	259
Trim72	272	332	369	250	428	445	355	314
Trim8	1441	1616	1461	1434	1543	1410	1386	1504
Trim80	48	39	31	35	32	31	47	38
Trim9	0	0	3	1	3	3	1	8
Triml1	0	0	1	0	0	0	0	0
Trio	474	518	507	560	465	465	438	438
Triobp	2917	3309	3205	3071	2936	2994	2773	2766
Trip10	1558	1659	1778	1939	1655	1605	1760	1772
Trip11	2467	2859	2509	2316	2754	3276	3187	2623
Trip12	6638	7277	6760	6463	7142	7093	7334	6519
Trip13	293	324	334	262	321	302	281	289
Trip4	1512	1555	1454	1458	1890	1901	1751	1795
Trip6	163	130	192	107	186	193	140	151
Triqk	57	63	51	55	53	72	68	47
Trir	3237	3331	3557	3339	3168	3425	2952	2950
Trit1	265	292	254	269	265	245	277	226
Trmo	119	111	112	106	137	103	90	138
Trmt1	1000	1173	1152	1097	1183	1229	1013	1011
Trmt10a	156	203	193	162	190	220	160	198
Trmt10b	159	199	169	197	214	177	176	167
Trmt10c	587	632	584	614	675	704	622	617
Trmt11	356	344	322	325	334	336	315	261
Trmt112	422	547	561	478	481	494	471	425
Trmt12	188	179	224	184	158	177	163	219
Trmt13	110	142	160	188	172	162	153	179
Trmt1l	1548	1602	1683	1655	1952	1937	1802	1728
Trmt2a	887	821	838	655	922	910	866	859
Trmt2b	1422	1363	1428	1517	1240	1388	1551	1339
Trmt44	306	419	397	404	472	473	426	413
Trmt5	194	275	217	280	351	300	270	265
Trmt6	680	797	724	727	726	639	670	592
Trmt61a	348	381	381	296	423	327	329	414
Trmt9b	38	15	42	23	18	19	25	7
Trmu	245	262	282	283	237	261	215	211
Trnau1ap	625	602	750	597	652	615	593	581
Trnp1	46	63	81	67	92	99	104	77
Trnt1	925	918	927	849	955	1018	990	786
Tro	7	12	7	29	13	7	5	11
Troap	131	114	114	123	169	143	95	117
Trp53	1826	1925	1980	1751	1882	1873	1434	1725
Trp53bp1	487	656	531	541	609	559	446	480

Transcriptome sequencing yielded total genetic results for the MOD and APS groups, with a total of 15,936 variables

Continued from above

Trp53bp2	1612	1725	1525	1516	1577	1614	1614	1464
Trp53i11	1750	1953	2172	1702	2114	2058	1979	2309
Trp53i13	548	446	496	496	417	559	513	479
Trp53inp1	1011	1280	1188	1218	1081	1166	1116	1134
Trp53inp2	4900	4873	4981	5276	4919	5025	4948	5064
Trp53rka	239	228	231	257	278	267	217	219
Trp53rkb	88	91	126	110	86	83	48	96
Trp53tg5	0	0	3	0	1	1	0	0
Trp63	0	0	0	0	0	4	0	1
Trp73	3	6	4	2	1	5	16	4
Trpa1	162	156	137	172	148	116	126	164
Trpc1	75	61	66	84	94	59	77	83
Trpc3	13	6	33	4	15	28	21	7
Trpc4	7	6	11	10	6	6	9	2
Trpc4ap	4273	4652	4356	4358	4442	4587	4319	4307
Trpc5	0	11	0	2	0	1	1	1
Trpc6	4	9	11	17	8	8	3	10
Trpc7	2	11	4	12	15	2	2	4
Trpm1	0	0	0	1	0	0	0	0
Trpm2	162	162	111	154	114	97	115	106
Trpm3	0	4	1	2	0	7	2	9
Trpm4	6272	6600	6109	5717	5828	6111	6129	5728
Trpm5	345	441	279	167	322	375	490	368
Trpm6	23	43	18	19	62	25	17	33
Trpm7	2211	2349	2250	2305	2285	2404	2596	2258
Trps1	47	63	55	72	44	47	57	63
Trpt1	117	95	118	88	88	120	119	130
Trpv1	0	1	3	1	5	5	0	0
Trpv2	75	91	98	86	85	95	85	67
Trpv3	72	80	87	85	57	106	56	92
Trpv4	33	55	29	29	19	17	21	22
Trpv6	8	4	14	5	18	7	3	0
Trrap	2101	2386	2539	2230	2231	2112	2168	1927
Trub1	299	306	295	284	319	292	248	263
Trub2	1100	1116	1054	899	899	948	984	925
Try10	21	23	26	0	121	99	65	95
Try4	4721	5502	2516	1064	18897	16236	11278	15270
Try5	2978	3644	1488	691	14980	13774	11799	14594
Tsacc	23	17	18	12	16	26	29	30
Tsc1	966	1135	1009	1037	1012	1087	1136	992
Tsc2	1990	2322	2337	2154	2134	2147	2017	1959
Tsc22d1	7460	6293	6913	8268	6428	6880	6360	5625
Tsc22d2	904	978	966	1062	1016	1077	868	899
Tsc22d3	1896	2414	2733	2214	2350	2514	2727	2488
Tsc22d4	3317	3427	3469	3103	3419	3626	3396	3350
Tsen15	107	113	157	152	115	149	116	132
Tsen2	141	144	165	125	222	189	157	213
Tsen34	2292	2178	2267	2197	2292	2057	2005	1943
Tsen54	472	571	505	499	547	553	534	473
Tsfm	957	998	959	875	1055	954	842	838
Tsg101	2765	2904	2641	2906	2527	2854	2511	2470
Tsga10	58	29	21	19	33	34	26	54
Tsga10ip	0	0	3	1	6	1	5	5
Tshr	0	1	5	0	0	11	0	1
Tshz1	702	790	794	942	839	740	775	638
Tshz2	233	258	171	205	206	195	230	158
Tshz3	73	66	90	58	51	58	46	58
Tsks	0	1	0	1	1	3	1	0

Transcriptome sequencing yielded total genetic results for the MOD and APS groups, with a total of 15,936 variables

Continued from above

Tsku	1368	1634	1417	1185	1591	1680	1677	1663
Tslp	7	1	1	1	2	1	1	3
Tsn	1741	1956	1973	1698	1797	1873	1673	1720
Tsnax	1952	1854	1787	1743	1840	1771	1689	1588
Tspan1	2777	2926	2963	2904	3722	3369	2943	3301
Tspan10	5	0	2	1	0	0	0	1
Tspan11	22	27	30	18	18	30	8	9
Tspan12	546	593	527	460	675	676	669	656
Tspan13	5477	5984	5380	5049	6215	6183	6334	6003
Tspan14	2004	1987	2166	2026	1865	1822	1637	1851
Tspan15	8119	7521	8442	8853	6382	6431	6576	5911
Tspan17	202	199	182	169	190	193	157	181
Tspan18	154	91	170	164	130	145	146	105
Tspan2	166	217	195	179	199	186	212	182
Tspan3	15817	17060	16128	15349	16968	17270	17294	16327
Tspan31	3842	3875	3752	3633	4213	4214	3851	3743
Tspan32	38	6	5	32	30	22	13	7
Tspan33	28	15	11	25	6	28	8	9
Tspan4	265	267	268	240	241	209	190	177
Tspan5	934	1087	976	867	986	1148	1308	1145
Tspan6	104	102	111	122	138	144	131	116
Tspan7	3998	4209	3980	3929	4165	3849	3826	4004
Tspan8	15493	15021	15690	16730	15674	15929	16039	16650
Tspan9	388	455	433	339	373	405	366	365
Tspear	9	5	1	3	4	1	0	1
Tspo	2607	2636	2894	2740	2769	2819	2373	2420
Tspo2	50	50	39	43	26	37	41	65
Tspoap1	5	1	6	4	7	10	5	4
Tspyl1	1986	2144	1992	1908	2019	2101	2104	2022
Tspyl2	97	90	99	96	64	73	66	73
Tspyl3	28	49	32	44	47	57	58	27
Tspyl4	82	99	97	114	92	125	81	113
Tspyl5	9	6	1	26	21	10	6	12
Tsr1	1063	1261	1138	1104	1368	1116	1255	1176
Tsr2	284	227	217	211	237	256	235	230
Tsr3	314	302	277	310	293	309	257	331
Tssc4	568	536	593	440	545	505	517	534
Tssk1	3	0	0	1	1	0	0	1
Tssk2	1	0	0	0	0	0	0	0
Tssk4	67	79	59	81	94	80	83	99
Tssk6	14	23	36	1	22	23	30	5
Tst	215	257	285	252	310	249	250	302
Tstd1	1350	1475	1340	1275	1493	1570	1330	1387
Tstd2	1054	1131	1150	1091	1051	1053	989	1067
Tstd3	1334	1410	1238	1363	1310	1389	1471	1320
Tsx	6	2	6	0	6	0	2	1
Ttbk1	9	3	4	5	4	11	3	4
Ttbk2	263	237	288	293	247	223	232	218
Ttc1	911	1025	955	851	980	984	1015	874
Ttc12	5	7	23	28	15	21	17	10
Ttc13	1509	1613	1651	1549	1497	1623	1656	1586
Ttc14	520	526	533	457	577	578	562	551
Ttc16	65	94	81	113	90	69	94	103
Ttc17	1558	1576	1356	1540	1394	1398	1474	1418
Ttc19	837	768	841	779	793	808	890	832
Ttc21a	1	0	0	0	1	1	0	1
Ttc21b	157	170	160	122	130	159	123	109
Ttc22	5564	5507	5161	5075	5215	5574	5584	5312

Transcriptome sequencing yielded total genetic results for the MOD and APS groups, with a total of 15,936 variables

Continued from above

Ttc23	231	270	279	325	235	273	241	248
Ttc24	0	0	0	1	0	0	0	0
Ttc25	2	2	0	1	0	4	1	0
Ttc26	28	16	24	27	21	22	20	20
Ttc27	404	426	469	406	407	476	419	441
Ttc28	159	140	164	169	150	179	130	99
Ttc3	1173	1398	1300	1342	1506	1564	1380	1202
Ttc30a1	77	108	107	105	139	154	105	134
Ttc30a2	0	1	2	0	2	4	1	2
Ttc30b	247	216	257	297	253	227	224	261
Ttc32	205	242	238	236	223	256	236	227
Ttc33	426	589	437	458	639	622	566	480
Ttc34	0	0	0	0	1	0	0	0
Ttc36	38	26	27	33	27	23	41	28
Ttc37	892	1053	884	922	1003	1071	1051	1005
Ttc38	1249	1263	1243	1294	1313	1235	1175	1247
Ttc39a	571	577	636	618	615	577	519	719
Ttc39b	2471	2719	2536	2458	2270	2409	2427	2400
Ttc39c	881	964	700	680	906	905	1020	914
Ttc4	1482	1603	1401	1329	1452	1521	1453	1336
Ttc41	14	39	40	21	32	40	35	27
Ttc5	896	753	777	793	855	757	706	737
Ttc6	41	20	13	20	17	16	12	5
Ttc7	7165	7614	7115	6647	7265	7025	7177	6766
Ttc7b	129	162	161	173	179	188	169	151
Ttc8	27	34	15	29	23	34	21	32
Ttc9	26	21	10	58	16	44	18	29
Ttc9b	4	1	11	1	1	1	0	0
Ttc9c	1869	2042	1926	1814	2251	2207	2002	1906
Ttf1	614	652	699	638	628	670	640	646
Ttf2	319	388	364	333	326	340	300	279
Tti1	558	783	607	586	681	696	627	565
Tti2	349	418	378	373	334	397	373	326
Ttk	265	255	278	228	311	239	196	276
Ttl	123	105	124	95	157	112	102	130
Ttll1	54	27	40	43	61	46	53	51
Ttll10	85	103	59	68	75	57	53	59
Ttll11	17	16	46	7	27	18	22	13
Ttll12	1148	1172	1329	1128	1269	1156	1104	1164
Ttll13	11	9	4	4	1	1	1	4
Ttll2	211	264	190	215	190	246	246	273
Ttll3	117	103	123	134	109	167	132	168
Ttll4	878	972	926	972	982	956	962	827
Ttll5	443	535	573	464	516	538	437	366
Ttll6	0	2	1	6	2	5	1	1
Ttll7	113	87	108	115	112	95	86	100
Ttll8	5	1	0	0	1	9	0	4
Ttll9	0	0	0	0	0	5	1	1
Ttn	5	0	4	5	0	4	6	0
Ttpa	35	20	27	16	29	32	12	17
Ttpal	922	935	921	884	930	932	959	899
Ttr	58	69	83	74	121	88	98	97
Ttyh1	126	120	127	163	96	116	151	94
Ttyh2	4615	4996	4696	4348	4988	5310	5118	4845
Ttyh3	1991	2150	2204	1989	1948	2172	1915	1717
Tub	43	31	24	22	14	32	37	37
Tuba1a	1108	1044	1132	1066	1170	1159	1083	1180
Tuba1b	5419	6131	5339	5292	5200	5424	4681	5291

Transcriptome sequencing yielded total genetic results for the MOD and APS groups, with a total of 15,936 variables

Continued from above

Tuba1c	7107	7400	6988	6617	7002	6880	6799	6729
Tuba3a	1	1	0	0	0	0	0	0
Tuba4a	3882	4098	3898	4059	3554	3555	3618	3560
Tuba8	5	5	10	1	11	5	5	4
Tubal3	2815	3303	2978	2628	2939	3063	3157	2939
Tubb1	0	0	1	0	0	0	0	1
Tubb2a	543	611	602	629	647	752	709	729
Tubb2b	348	296	335	371	318	291	283	253
Tubb3	152	156	131	146	97	101	77	126
Tubb4a	59	101	58	71	53	42	39	68
Tubb4b	16867	18798	17536	16740	16617	16849	16310	16677
Tubb5	5913	6544	6030	6008	5915	5819	5053	5159
Tubb6	395	392	359	417	429	423	454	385
Tubd1	189	318	268	272	371	322	361	259
Tube1	67	114	105	77	101	70	81	77
Tubg1	561	477	508	505	606	526	447	525
Tubg2	10	19	14	5	33	11	19	7
Tubgcp2	464	537	490	465	407	460	426	377
Tubgcp3	859	943	913	854	943	958	799	814
Tubgcp4	1068	1186	1217	1084	1305	1321	1192	1100
Tubgcp5	262	330	304	283	330	339	369	259
Tubgcp6	905	819	827	897	849	847	798	789
Tufm	6266	6100	6313	6283	6194	6044	5484	5933
Tuft1	1258	1195	1153	1069	1126	1097	1211	1137
Tulp1	2	0	0	1	1	0	2	0
Tulp2	1	4	5	6	4	4	6	7
Tulp3	130	189	171	139	172	134	138	119
Tulp4	2132	2297	2120	2265	2241	2350	2382	2082
Tusc1	175	135	155	172	174	122	130	140
Tusc2	1177	1254	1124	1301	1186	1247	1060	1214
Tusc3	703	709	653	663	785	676	593	589
Tut1	721	851	688	656	798	777	782	736
Tut4	544	813	641	511	631	590	563	545
Tut7	5125	6269	5485	5075	5557	6452	5884	5237
Tvp23a	141	150	150	164	115	130	140	124
Tvp23b	1209	1243	1319	1281	1518	1633	1425	1402
Twf1	5062	5449	4988	5200	5200	5683	5189	5153
Twf2	397	510	432	424	514	534	456	475
Twist1	1	0	2	1	10	4	3	6
Twist2	10	4	2	21	9	3	4	2
Twistnb	442	432	436	381	507	490	502	483
Twnk	754	801	737	752	733	720	631	686
Twsg1	2093	2185	1966	1997	2066	2141	1979	1912
Txk	14	13	16	27	13	19	15	3
Txlna	1509	1791	1581	1536	1888	1692	1375	1606
Txlnb	1	4	6	2	5	7	5	5
Txlng	653	642	580	638	741	855	924	678
Txn1	33638	34045	35272	36027	33519	35531	34376	32932
Txn2	5844	6062	5929	5735	5719	5643	5389	5298
Txndc11	1613	1568	1653	1702	1947	1709	1564	1618
Txndc12	1173	1153	1133	1147	1307	1140	1235	1024
Txndc15	1191	1164	1170	1073	1311	1338	1172	1162
Txndc16	342	348	388	424	327	316	354	273
Txndc17	7232	7353	7226	6865	7045	7498	7414	6920
Txndc2	0	0	0	0	1	0	0	0
Txndc5	11496	11946	11906	11744	11778	11199	10564	11257
Txndc8	0	0	0	0	0	4	0	0
Txndc9	2644	2658	2380	2522	2604	2754	2568	2416

Transcriptome sequencing yielded total genetic results for the MOD and APS groups, with a total of 15,936 variables

Continued from above

Txnip	11941	12738	13143	13433	11373	11722	10938	11297
Txnl1	2858	3218	3086	2680	3205	3397	3018	2963
Txnl4a	455	402	393	402	462	453	419	473
Txnl4b	265	297	253	296	304	295	260	282
Txnrd1	14998	16146	15199	14219	17754	17264	16042	16058
Txnrd2	832	851	956	835	822	865	824	854
Txnrd3	170	242	181	180	142	147	135	152
Tyk2	1842	1831	1996	2100	2051	2088	1888	1877
Tymp	522	592	427	373	716	740	992	947
Tyms	896	977	967	878	887	956	747	795
Tyro3	570	435	407	329	517	452	406	406
Tyrobp	392	353	328	365	313	390	355	327
Tysnd1	2180	2265	2299	2055	2429	2249	2172	2156
Tyw1	1061	1215	1066	885	1437	1260	1264	1287
Tyw3	26	23	37	34	43	35	25	30
Tyw5	143	100	127	161	133	151	163	126
U2af1	1257	1289	1258	1322	1506	1223	1350	1175
U2af1l4	727	739	694	736	748	754	707	687
U2af2	4375	4575	4442	4653	4567	4472	4215	4149
U2surp	2031	2101	2098	2113	2138	2066	2064	1989
Uaca	2737	2934	2950	3063	2920	2908	2904	2573
Uap1	3019	3597	3193	3336	3581	3397	3392	3361
Uap1l1	3973	4440	3857	3882	3895	4153	4154	4309
Uba1	14055	15489	14404	13323	14070	13803	13355	13174
Uba2	2438	2670	2415	2466	2657	2477	2551	2358
Uba3	2093	2179	2133	2166	2129	2195	2227	2030
Uba5	1954	1854	1577	1752	2022	2193	2009	2253
Uba52	16725	16891	17014	17125	16739	16804	14588	15615
Uba6	526	678	672	571	829	911	938	812
Uba7	4217	4845	4211	3818	4154	4430	4107	3953
Ubac1	1795	1782	1883	1954	1833	1838	1745	1675
Ubac2	1524	1824	1664	1503	1605	1667	1669	1529
Ubald1	590	519	602	532	575	576	534	497
Ubald2	4665	4865	5264	5114	4933	5001	5228	5379
Ubap1	2798	3043	2814	2721	2966	3078	3006	2725
Ubap1l	17	17	25	27	29	19	32	20
Ubap2	1619	1876	1734	1757	1942	1723	1575	1626
Ubap2l	3691	3561	3562	3406	3489	3624	3532	3454
Ubash3a	17	4	7	8	9	7	3	3
Ubash3b	267	256	261	275	159	143	187	182
Ubb	36259	37766	35713	34472	36974	38213	35558	35492
Ubc	5152	5660	4943	4654	5256	5548	5214	5140
Ubd	4173	3913	2945	3639	1775	2115	2314	2015
Ube2a	2058	2095	2096	1992	1944	2248	2041	2098
Ube2b	2880	3304	3121	3110	3275	3156	3258	3164
Ube2c	1694	1817	1843	1639	1685	1651	1416	1508
Ube2cbp	33	37	55	32	36	66	57	52
Ube2d1	473	546	559	530	549	493	470	457
Ube2d2a	2154	2308	2289	2266	2080	2522	2167	2135
Ube2d3	14253	15268	13765	13749	14173	14431	14080	13147
Ube2e1	1500	1596	1427	1453	1593	1425	1308	1377
Ube2e2	137	198	206	158	196	218	150	150
Ube2e3	1167	1296	1199	1181	1175	1128	1157	1136
Ube2f	1155	1206	1136	1186	1299	1280	1256	1192
Ube2g1	1598	1830	1814	1805	1594	1685	1625	1595
Ube2g2	1213	1418	1359	1239	1698	1545	1353	1483
Ube2h	2044	2347	2267	1879	2191	2274	2141	2300
Ube2i	4140	4288	4206	4086	4404	4378	3948	4007

Transcriptome sequencing yielded total genetic results for the MOD and APS groups, with a total of 15,936 variables

Continued from above

Ube2j1	2992	3254	3035	2999	3316	3383	2958	3107
Ube2j2	3717	3728	3476	3412	3559	3667	3567	3492
Ube2k	2266	2469	2342	2309	2451	2412	2233	2163
Ube2l3	6126	6307	6233	5957	6383	6326	5937	5823
Ube2l6	5053	5799	5129	4686	4751	4971	4629	4170
Ube2m	4263	4020	3943	4165	3663	3771	3598	3787
Ube2n	2722	3035	2761	2754	2982	3026	2888	2897
Ube2o	475	535	543	469	536	612	480	522
Ube2q1	4108	4266	4097	4056	3703	3736	4044	3872
Ube2q2	641	596	583	634	724	599	697	634
Ube2ql1	4	3	3	7	0	4	5	13
Ube2r2	1967	2182	2140	2015	1874	1974	2035	1943
Ube2s	1958	2094	2061	2076	2221	2146	1925	2151
Ube2t	103	103	108	97	101	74	68	118
Ube2u	0	0	0	0	1	0	0	0
Ube2v1	2332	2159	2148	2052	2048	2073	2080	1844
Ube2v2	161	180	120	163	198	147	178	174
Ube2w	505	529	453	450	625	525	530	480
Ube2z	4605	4814	4678	4450	4534	4963	4879	4449
Ube3a	1580	1708	1611	1538	1932	2114	1884	1733
Ube3b	4970	5216	5001	5208	4774	4988	4855	4516
Ube3c	2365	2914	2798	2582	2809	2830	2678	2404
Ube4a	2786	3206	2965	2931	3366	3436	3218	3037
Ube4b	5937	5419	5644	6095	5407	5841	5706	5616
Ubfd1	2325	2521	2632	2383	3153	2928	2825	2781
Ubiad1	678	709	676	643	643	630	666	780
Ubl3	3468	3494	3299	3307	3369	3406	3433	3205
Ubl4a	701	765	831	828	833	743	748	741
Ubl5	2513	2777	2719	2608	2779	2919	2621	2631
Ubl7	1545	1544	1584	1649	1443	1573	1419	1441
Ublcp1	768	786	659	718	879	774	683	750
Ubn1	2457	2557	2562	2572	2757	2821	2700	2403
Ubn2	1119	1408	1302	1465	1461	1480	1379	1284
Ubox5	320	366	298	275	284	269	222	236
Ubp1	3207	3587	3556	3418	3633	3431	3409	3188
Ubqln1	6328	6767	6298	6273	7241	7322	6763	6717
Ubqln2	616	669	625	621	728	616	624	611
Ubqln4	2373	2514	2373	2227	2371	2299	2033	2122
Ubr1	657	752	698	675	611	706	712	667
Ubr2	1162	1191	1312	1281	1597	1372	1142	1208
Ubr3	4222	4623	4137	4052	4033	4192	4395	4102
Ubr4	8353	9413	8489	8499	8526	9046	8272	7510
Ubr5	4412	5196	4459	4720	4673	4623	4692	4430
Ubr7	999	1095	1090	999	1042	921	970	1025
Ubtd1	285	314	308	294	274	317	249	246
Ubtd2	118	94	72	108	113	108	125	101
Ubtf	3302	3424	3239	3431	3597	3618	3265	3320
Ubxn1	2827	2782	2896	2583	2769	2673	2471	2688
Ubxn10	19	13	11	20	38	36	35	19
Ubxn11	15	19	8	2	13	23	6	16
Ubxn2a	5484	5911	5628	5709	6026	6655	6800	6026
Ubxn2b	149	165	173	180	260	179	199	208
Ubxn4	2803	3096	2672	2626	3694	3524	3153	3179
Ubxn6	2253	2347	2238	2137	1940	1992	2207	1994
Ubxn7	696	731	742	773	759	748	728	657
Ubxn8	1357	1317	1291	1298	1253	1204	1124	1207
Uchl1	153	174	186	189	104	157	151	177
Uchl3	488	647	579	552	690	588	576	552

Transcriptome sequencing yielded total genetic results for the MOD and APS groups, with a total of 15,936 variables

Continued from above

Uchl4	20	44	12	20	38	17	24	18
Uchl5	800	882	847	805	892	844	811	783
Uck1	1758	2009	1922	1974	2175	2087	1924	1972
Uck2	1878	1983	2037	1964	2021	1942	1851	1955
Uckl1	1739	1596	1711	1626	1612	1605	1555	1479
Ucma	0	0	0	0	1	0	0	4
Ucn2	0	0	0	1	0	0	0	1
Ucn3	12	24	10	11	9	7	15	10
Ucp2	22705	21949	23181	23911	21653	21062	19703	20766
Ucp3	0	2	7	4	4	1	9	2
Uevld	770	821	768	813	775	747	690	677
Ufc1	1815	1911	1707	1784	1901	1953	1781	1670
Ufd1	1295	1266	1196	1185	1202	1396	1261	1203
Ufl1	1349	1473	1387	1299	1471	1618	1448	1545
Ufm1	3231	3333	3406	3585	3539	3556	3487	3298
Ufsp1	175	185	168	214	154	171	126	153
Ufsp2	1641	1789	1610	1609	1885	1868	1997	1821
Ugcg	3913	4775	4112	4118	3501	3673	3938	3794
Ugdh	18030	19709	19084	18506	22073	22222	20778	20791
Uggt1	4235	4852	4649	4278	4981	5028	4441	4460
Uggt2	25	49	22	13	24	21	11	8
Ugp2	3992	4351	3959	3835	4306	4555	4370	4121
Ugt1a1	1680	2425	1687	1199	2236	2740	2458	1892
Ugt1a2	1	1	2	0	2	1	7	4
Ugt1a5	5	0	2	0	1	4	1	0
Ugt1a6a	273	250	207	164	285	200	177	259
Ugt1a6b	9	2	1	2	6	5	10	4
Ugt1a7c	9798	10676	9267	8916	9825	10562	11051	10264
Ugt1a8	1	0	0	2	0	1	1	5
Ugt1a9	94	94	141	126	54	85	73	99
Ugt2a3	1809	2051	1671	1562	2062	2513	2305	1729
Ugt2b1	1	4	2	1	4	1	3	5
Ugt2b34	19459	21795	19580	17822	24338	25481	23913	21985
Ugt2b35	1429	1642	1569	1442	1710	1754	1690	1518
Ugt2b36	103	91	72	51	127	150	96	111
Ugt2b37	0	0	0	1	1	4	1	0
Ugt2b38	85	79	76	30	140	152	91	56
Ugt2b5	182	213	169	110	383	378	306	268
Ugt3a1	0	0	0	1	0	0	0	1
Ugt3a2	1	0	1	0	2	5	4	0
Ugt8a	0	1	1	0	1	1	2	0
Uhmk1	3449	3737	3717	3807	3762	3742	3763	3649
Uhrf1	1243	1412	1291	1185	1307	1175	1090	1210
Uhrf1bp1	381	408	382	392	394	393	325	296
Uhrf1bp1l	2160	2301	1988	2058	2043	2031	1988	2063
Uhrf2	878	927	1124	1022	1191	1178	1133	989
Uimc1	474	452	420	502	518	502	374	463
Ulbp1	17	14	10	16	10	9	6	14
Ulk1	1065	1129	1113	1106	1022	1003	987	1000
Ulk2	785	874	716	824	905	1057	929	786
Ulk3	350	331	380	371	371	310	334	338
Ulk4	14	5	20	7	9	5	1	17
Umad1	1045	1168	1076	1077	1048	1091	968	1159
Umod	0	0	0	0	1	1	4	0
Umps	577	563	552	495	642	671	494	507
Unc119	191	145	138	140	182	134	184	116
Unc119b	1811	2023	2042	1869	2186	2008	1838	1961
Unc13a	26	40	18	41	28	31	29	41

Transcriptome sequencing yielded total genetic results for the MOD and APS groups, with a total of 15,936 variables

Continued from above

Unc13b	930	1000	987	976	961	939	905	863
Unc13c	0	0	0	0	0	0	0	4
Unc13d	362	233	227	230	243	241	178	251
Unc45a	1616	1737	1773	1739	1580	1496	1478	1455
Unc45b	28	38	50	19	24	39	41	14
Unc50	1371	1570	1480	1562	1666	1660	1491	1506
Unc5a	172	133	218	158	155	172	157	155
Unc5b	2384	2429	2101	2314	2539	2530	2978	2527
Unc5c	27	20	36	21	36	32	15	21
Unc5cl	10427	10553	9130	9004	10187	10885	10662	9810
Unc5d	0	0	6	1	0	0	0	0
Unc79	3	6	7	8	5	12	2	4
Unc80	9	18	9	8	21	13	18	3
Unc93a	821	631	719	929	328	339	518	508
Unc93a2	3819	3576	3017	3506	2955	3001	4047	3577
Unc93b1	2926	2979	2729	2801	2608	2822	2505	2474
Uncx	1	0	0	0	0	0	0	0
Ung	363	310	299	256	334	290	270	288
Unk	540	555	525	580	568	548	515	516
Unkl	194	277	192	211	285	242	272	236
Uox	8	19	9	23	24	7	1	21
Upb1	497	519	500	601	551	483	684	587
Upf1	3714	3920	3708	3967	3949	3965	3693	3582
Upf2	1082	1229	1036	1061	1089	1266	1321	1185
Upf3a	539	620	580	600	642	586	578	594
Upf3b	435	418	392	391	455	369	470	451
Upk1a	29	26	27	24	42	29	49	16
Upk1b	160	175	138	148	189	202	228	203
Upk2	0	0	4	0	1	0	0	0
Upk3a	0	14	12	2	8	7	7	9
Upk3b	40	40	54	63	54	97	119	82
Upk3bl	2	14	5	25	5	1	0	15
Upp1	11276	11220	9972	10069	8029	8616	8459	8219
Upp2	41	34	12	43	37	62	46	34
Uprt	1543	1750	1627	1804	1838	1923	2029	1704
Uqcc1	1643	1803	1644	1604	1810	1751	1715	1681
Uqcc2	732	871	852	757	951	868	783	856
Uqcc3	365	381	384	360	373	329	264	304
Uqcr10	5996	5997	6348	6434	6331	6286	5840	5936
Uqcr11	4340	4405	4777	4989	4438	4723	4178	4220
Uqcrb	5591	5955	5540	6109	5324	5576	5901	5407
Uqcrc1	38966	37421	37163	38330	34689	35253	34913	35006
Uqcrc2	20095	21232	20146	20510	20667	20865	19831	19680
Uqcrfs1	20746	20524	19811	20750	18913	19255	19089	18621
Uqcrh	10595	11065	11044	11730	11187	11476	10875	11009
Uqcrq	10534	10557	10422	11114	10076	10560	9759	9630
Urad	37	40	25	22	41	35	19	25
Urah	431	501	462	420	503	466	470	500
Urb1	245	335	283	212	376	358	318	273
Urb2	784	917	824	781	818	908	776	727
Urgcp	4128	3960	3842	3756	4109	4294	4072	3677
Uri1	913	1127	933	923	978	1048	969	832
Urm1	1121	1337	1190	1288	1374	1229	1260	1181
Uroc1	2	3	2	0	0	1	0	2
Urod	885	900	917	862	948	929	829	916
Uros	405	365	468	387	412	426	358	382
Usb1	570	597	581	610	552	607	573	553
Use1	2265	2255	2150	2240	2068	2147	2016	2021

Transcriptome sequencing yielded total genetic results for the MOD and APS groups, with a total of 15,936 variables

Continued from above

Usf1	1484	1560	1539	1482	1353	1307	1253	1213
Usf2	1112	1147	1400	1332	1181	1228	1156	1062
Usf3	1971	2268	2161	2076	2198	2165	1918	1894
Ush1c	8961	8972	8702	8846	8586	9020	9024	8324
Ush1g	1	1	0	1	1	8	1	0
Ush2a	0	4	1	2	1	0	1	2
Ushbp1	120	93	86	90	74	66	113	60
Uso1	7516	8181	7940	7848	8212	8420	8541	8133
Usp1	2267	2350	2177	2246	2648	2492	2530	2313
Usp10	2654	2898	2858	2521	2914	3152	2728	2591
Usp11	127	77	68	76	130	97	86	60
Usp12	5069	5430	5068	4974	5436	5596	5345	5064
Usp13	1	1	0	2	3	12	6	2
Usp14	1789	1998	1917	1797	2091	2010	1801	1729
Usp15	2277	2514	2216	2187	2485	2653	2709	2316
Usp16	2125	2212	1884	1923	2377	2439	2401	2175
Usp18	4455	5385	4858	4265	3256	3516	3477	3416
Usp19	3311	3589	3392	3478	3513	3658	3262	3287
Usp2	2047	1925	2057	2200	1866	2033	2130	2151
Usp20	371	337	340	337	376	358	308	372
Usp21	728	829	754	682	773	702	693	668
Usp22	2841	2887	2833	2826	2658	2760	2700	2617
Usp24	2385	2731	2532	2479	2630	2699	2417	2038
Usp25	4493	5057	4478	4168	4268	4622	4382	4086
Usp27x	9	15	18	12	11	12	5	33
Usp28	429	430	498	479	395	371	461	386
Usp29	0	0	4	2	1	1	7	1
Usp3	2763	3071	2803	2618	3140	2861	2774	2679
Usp30	592	628	624	522	585	585	529	495
Usp31	208	211	216	218	259	236	190	224
Usp32	2246	2177	2098	2340	2170	2054	2064	2018
Usp33	2395	2683	2486	2688	2714	2828	2811	2534
Usp34	2982	2956	3143	2959	2906	3359	3287	2798
Usp35	69	97	70	87	103	98	99	79
Usp36	830	869	846	832	926	911	781	856
Usp37	744	888	645	807	839	905	833	833
Usp38	1980	2079	2012	2098	2132	2417	2141	2069
Usp39	1052	1089	939	957	1114	1123	1050	932
Usp4	4431	5045	4879	4315	4817	4719	4421	4094
Usp40	1760	1989	1810	1820	1682	1864	1888	1688
Usp42	803	861	856	783	784	878	742	772
Usp43	1351	1410	1454	1486	1617	1568	1448	1608
Usp44	4	5	1	6	8	0	3	4
Usp45	1295	1376	1355	1411	1504	1435	1299	1336
Usp46	417	582	571	549	562	497	547	510
Usp47	4626	5383	4791	4683	5316	5235	5467	4641
Usp48	1461	1699	1572	1565	1685	1741	1562	1461
Usp49	644	661	648	562	588	666	512	487
Usp5	3143	3181	3303	3142	3276	2982	2960	3106
Usp50	0	1	0	1	0	0	2	0
Usp51	0	0	1	6	0	0	0	1
Usp53	566	647	603	629	615	576	697	494
Usp54	125	183	138	154	158	170	178	155
Usp6nl	1616	1791	1842	1779	1916	1827	1733	1700
Usp7	4079	4458	4139	4192	3819	4217	3834	3781
Usp8	2805	3239	2859	2839	3027	3227	2991	2989
Usp9x	3631	4405	3806	3857	4399	5013	4921	3965
Uspl1	654	728	654	605	845	845	662	765

Transcriptome sequencing yielded total genetic results for the MOD and APS groups, with a total of 15,936 variables

Continued from above

Ust	27	21	20	22	11	19	19	15
Utp11	549	565	529	641	654	636	562	591
Utp14a	552	702	599	564	661	732	719	476
Utp14b	151	166	106	119	205	235	253	221
Utp15	699	680	725	613	779	719	626	766
Utp18	576	534	584	566	617	639	583	639
Utp20	739	868	747	858	1022	912	836	733
Utp23	332	415	334	389	390	373	303	346
Utp25	224	303	257	235	317	337	245	256
Utp3	855	801	791	800	800	926	804	802
Utp4	629	627	521	533	689	587	552	574
Utp6	1028	1340	1223	1283	1416	1441	1263	1283
Utrn	3442	3612	3339	3435	3310	3466	3297	3216
Uts2b	0	1	0	0	0	0	0	0
Uty	585	269	1	213	109	134	434	708
Uvrag	856	830	905	913	790	963	825	846
Uvssa	325	330	352	355	444	357	353	292
Uxs1	467	590	434	533	464	511	478	477
Uxt	409	434	429	415	514	429	373	307
Vac14	1165	1072	1127	966	1055	1107	953	1025
Vamp1	110	134	125	177	155	173	139	117
Vamp2	920	958	1121	996	941	989	792	898
Vamp3	5359	5721	5288	5135	5149	5424	5058	5067
Vamp4	397	427	433	399	481	400	401	418
Vamp5	278	279	323	282	230	276	236	273
Vamp7	1764	2063	2031	1950	2250	2518	2176	2016
Vamp8	4874	5301	5161	4860	4887	5087	4927	4942
Vamp9	0	0	0	0	0	2	0	0
Vangl1	118	133	163	108	136	132	113	85
Vangl2	25	19	24	35	13	23	12	32
Vapa	6745	7364	6778	6556	7203	7315	7677	7207
Vapb	4580	4720	4309	4403	4473	4782	4326	4197
Vars	3768	4221	4072	3647	4209	4202	3722	3793
Vars2	807	934	802	788	894	794	780	774
Vash1	70	122	184	122	114	126	92	126
Vash2	16	16	18	17	24	15	5	18
Vasn	62	100	91	52	62	84	70	120
Vasp	9894	10032	9825	9421	9751	9801	9283	9323
Vat1	5783	6056	5873	6084	6234	6358	6337	6603
Vat1l	14	38	23	22	40	25	24	32
Vav1	182	162	228	219	144	193	172	124
Vav2	3499	3611	2957	3169	3414	3463	3526	2967
Vav3	99	123	125	94	101	112	124	114
Vax2	0	0	0	0	0	1	0	0
Vbp1	1094	1099	910	1014	1198	1203	1092	1149
Vcam1	408	333	365	446	298	281	247	231
Vcan	133	160	165	192	198	180	180	123
Vcl	3362	3278	3383	3572	3349	3353	3237	2871
Vcp	12840	13510	13199	12090	13675	14359	12354	12592
Vcpip1	2427	2771	2557	2371	2448	2883	2768	2507
Vcpkmt	380	436	333	350	373	415	448	382
Vdac1	16632	16564	16707	17319	16399	16109	15799	15857
Vdac2	24759	25318	24220	24616	23033	24072	23835	23477
Vdac3	4524	4699	4515	4782	4514	4413	4186	4094
Vdr	15919	17708	16575	15828	19173	20643	19192	17288
Vegfb	326	277	313	313	268	301	207	250
Vegfc	156	161	160	128	148	154	144	132
Vegfd	11	24	40	16	2	2	21	15

Transcriptome sequencing yielded total genetic results for the MOD and APS groups, with a total of 15,936 variables

Continued from above

Vezf1	1275	1347	1336	1373	1504	1438	1638	1388
Vezt	647	790	761	692	778	774	721	689
Vgf	27	31	29	40	25	26	34	27
Vgll3	20	11	13	18	27	25	20	10
Vgll4	1828	1879	1754	1833	1936	1745	1514	1663
Vhl	598	701	669	709	728	752	635	659
Vil1	98211	108094	97035	90983	98812	106127	108285	98609
Vill	60	54	51	32	52	67	60	44
Vim	1664	1914	2127	2063	1949	2035	1869	1854
Vinac1	1	2	1	1	1	7	0	0
Vip	402	402	397	397	458	381	409	478
Vipas39	2146	2411	2204	2351	2337	2340	2236	2229
Vipr1	11141	12810	13104	12077	12408	12618	11851	11070
Vipr2	38	45	30	36	20	40	34	17
Virma	1743	1803	1540	1714	1689	1914	1699	1608
Vit	3	1	2	6	1	7	14	2
Vkorc1	365	467	445	423	454	488	395	413
Vkorc1l1	2113	2085	1967	1961	2427	2213	2307	2027
Vldlr	127	96	93	143	110	136	81	100
Vma21	784	847	836	904	750	830	757	841
Vmac	212	217	211	275	164	210	174	207
Vmn1r181	0	1	0	0	0	0	0	0
Vmn1r199	0	0	0	1	0	0	0	0
Vmn1r4	1	1	0	0	0	0	5	1
Vmn1r53	1	1	1	0	0	0	0	1
Vmn1r54	0	0	0	0	0	0	0	6
Vmn1r91	15	19	26	35	5	15	28	4
Vmn2r1	0	0	0	0	5	0	0	0
Vmn2r118	0	1	0	0	0	0	0	0
Vmn2r18	1	0	1	0	0	0	1	0
Vmn2r26	0	0	0	0	0	0	0	1
Vmn2r29	2	14	2	3	3	6	7	8
Vmn2r31	0	0	0	0	0	0	0	2
Vmn2r57	0	2	0	0	0	0	0	0
Vmn2r96	0	0	1	0	0	0	0	0
Vmo1	1	4	10	2	1	0	1	0
Vmp1	13570	15274	14050	12600	15547	15512	15094	13701
Vnn1	3477	3522	3029	3015	3386	3700	4180	3583
Vnn3	29	19	10	14	13	11	32	5
Vopp1	375	388	385	437	416	442	414	383
Vpreb1	2	4	0	1	4	4	1	0
Vpreb3	24	15	22	11	11	17	16	16
Vps11	1677	1935	1803	1611	1635	1658	1619	1597
Vps13a	1523	1583	1488	1440	1510	1733	1719	1376
Vps13b	1651	1814	1811	1845	1812	1851	1763	1597
Vps13c	431	568	639	603	569	600	566	450
Vps13d	4875	5390	5128	5261	5076	4746	5182	4562
Vps16	972	1017	992	978	894	917	1037	904
Vps18	1710	1614	1560	1627	1617	1637	1618	1619
Vps25	2089	2487	2261	2251	2201	2180	2078	2105
Vps26a	3228	3276	3338	3550	2944	3145	3116	3187
Vps26b	1555	1586	1551	1403	1469	1498	1389	1365
Vps26c	1878	1814	1622	1572	1795	1733	1741	1694
Vps28	3345	3467	3315	3270	3205	3205	2917	2882
Vps29	2268	2281	2267	2251	2312	2307	2249	2120
Vps33a	1347	1402	1396	1235	1535	1333	1275	1362
Vps33b	680	678	653	653	700	733	757	635
Vps35	6475	6668	6064	6668	6992	7257	7272	6829

Transcriptome sequencing yielded total genetic results for the MOD and APS groups, with a total of 15,936 variables

Continued from above

Vps35l	1890	1966	1828	1886	2008	2042	1993	1812
Vps36	1852	1975	1895	1731	2004	2073	1966	1908
Vps37a	2019	2126	1949	2029	2107	2160	2317	1942
Vps37b	1668	1731	1642	1777	1745	1720	1817	1773
Vps37c	1137	1223	1249	1199	1293	1312	1196	1286
Vps37d	112	118	129	124	106	81	128	102
Vps39	1283	1250	1195	1151	1118	1148	1128	1133
Vps41	1982	2161	1934	2058	1953	2144	2308	1840
Vps45	799	981	886	715	803	902	815	757
Vps4a	2070	2069	2128	1959	2059	2220	2039	2110
Vps4b	4725	5252	4472	4565	4885	5384	5295	4931
Vps50	917	1114	1035	976	1146	1145	1004	1014
Vps51	1412	1362	1519	1572	1532	1462	1342	1432
Vps52	1970	2204	2014	2108	2202	1935	1942	2020
Vps53	2536	2544	2222	2451	2471	2687	2254	2210
Vps54	3369	3640	3455	3544	3325	3574	3716	3323
Vps72	1205	1147	1128	1228	1272	1238	1170	1096
Vps8	488	506	514	434	426	418	469	397
Vps9d1	602	695	659	724	500	523	621	556
Vrk1	521	572	440	513	540	581	438	545
Vrk2	219	216	197	239	192	212	188	190
Vrk3	1637	1810	1768	1654	1640	1595	1686	1654
Vrtn	4	0	0	1	0	0	0	0
Vsig1	2	0	0	1	0	0	0	0
Vsig10	1551	1722	1771	1574	1639	1610	1441	1425
Vsig10l	78	63	69	49	78	65	97	36
Vsig2	17	18	11	14	25	34	18	8
Vsig4	1	1	0	0	0	0	1	1
Vsig8	4	0	6	8	4	7	0	0
Vsir	735	760	852	795	800	722	741	659
Vsnl1	5	0	9	0	2	1	8	1
Vstm2a	0	1	4	2	0	1	1	0
Vstm2b	0	0	1	0	1	4	4	0
Vstm2l	204	225	231	315	261	243	253	245
Vstm4	173	237	185	209	224	148	167	164
Vstm5	670	781	853	756	801	748	725	771
Vsx2	1	0	0	0	0	0	0	0
Vta1	1393	1673	1488	1393	1482	1567	1464	1317
Vtcn1	0	0	0	0	0	0	0	2
Vti1a	1272	1377	1434	1182	1274	1254	1227	1269
Vti1b	3066	3612	3329	3084	3453	3610	3482	3384
Vtn	41	50	36	23	78	68	52	46
Vwa1	1200	1347	1326	1103	1557	1847	2168	1825
Vwa2	14	11	52	22	15	37	14	29
Vwa3a	0	0	1	0	0	0	4	0
Vwa3b	4	9	3	24	4	1	6	2
Vwa5a	3250	3468	3279	3242	2995	3236	2760	2437
Vwa5b1	3	0	5	3	8	2	11	5
Vwa5b2	73	101	80	114	107	66	123	50
Vwa7	62	60	41	73	55	63	40	53
Vwa8	3913	4342	3842	3776	4101	4024	3984	3788
Vwc2	12	15	10	3	37	4	12	14
Vwc2l	0	0	0	6	0	0	0	1
Vwce	387	477	460	414	428	431	440	333
Vwde	0	0	0	1	0	0	0	0
Vwf	863	725	857	892	638	665	679	653
Vxn	0	0	0	0	1	0	0	0
Wac	2673	2726	2695	2810	2988	2883	2934	2589

Transcriptome sequencing yielded total genetic results for the MOD and APS groups, with a total of 15,936 variables

Continued from above

Wapl	4331	4642	3969	4294	5185	5258	5111	4551
Wars	5084	4762	4657	4619	3865	3900	3437	3691
Wars2	385	404	339	437	459	425	412	421
Was	89	124	112	86	120	120	85	97
Wasf1	14	23	18	12	10	10	24	10
Wasf2	5783	6373	5746	5808	5694	6049	5829	5513
Wasf3	44	36	20	30	34	28	27	28
Washc1	789	862	852	746	758	845	693	823
Washc2	1953	2196	1903	1929	1966	2047	2023	2001
Washc3	605	766	654	581	724	557	626	675
Washc4	1027	1146	1108	1021	1229	1298	1255	1129
Washc5	1686	1749	1874	1678	1655	1702	1630	1473
Wasl	3388	3490	3392	3381	3484	3782	3554	3375
Wbp1	2367	2356	2333	2403	2298	2376	2218	2113
Wbp11	2549	2669	2489	2583	2569	2527	2348	2537
Wbp1l	2865	3265	3038	2850	3108	3019	3106	2944
Wbp2	5125	5443	5170	4965	4699	4940	5080	4834
Wbp4	789	902	849	914	865	917	876	918
Wdcp	78	73	69	65	91	76	82	94
Wdfy1	530	600	581	588	541	596	628	598
Wdfy2	481	527	531	541	563	514	547	460
Wdfy3	1348	1399	1365	1492	1330	1398	1345	1161
Wdfy4	98	95	121	116	107	52	95	71
Wdhd1	459	498	483	430	430	435	429	383
Wdpcp	685	773	597	527	636	649	735	590
Wdr1	12441	12930	12327	12660	12639	12823	12328	12785
Wdr11	2460	2531	2563	2499	2427	2415	2501	2333
Wdr12	398	440	400	371	464	432	367	418
Wdr13	1605	1809	1930	1834	1698	1677	1763	1669
Wdr17	1	1	0	0	0	1	0	1
Wdr18	1278	1268	1260	1256	1468	1393	1133	1283
Wdr19	35	63	55	58	49	28	46	43
Wdr20	999	981	1057	1006	1113	1177	959	1037
Wdr20rt	0	0	0	0	0	1	0	0
Wdr24	1040	1083	1111	1112	1040	1014	984	966
Wdr25	57	88	105	88	113	59	94	89
Wdr26	3309	3631	3650	3560	3706	3703	3693	3434
Wdr27	8	6	3	22	6	13	6	24
Wdr3	546	589	594	554	714	638	622	604
Wdr31	41	31	37	55	48	34	26	60
Wdr33	1705	1782	1837	1948	2008	1964	1877	1836
Wdr34	145	149	121	161	99	147	159	128
Wdr35	50	36	43	33	45	44	27	26
Wdr36	1257	1480	1216	1250	1326	1361	1262	1362
Wdr37	758	728	697	640	616	622	640	586
Wdr38	1	1	1	2	13	0	1	1
Wdr4	736	765	854	676	811	772	659	738
Wdr41	990	1008	970	986	977	934	987	802
Wdr43	1235	1285	1063	1150	1312	1286	1132	1171
Wdr44	1016	1023	1010	1066	976	992	925	863
Wdr45	696	629	658	678	600	516	621	575
Wdr45b	1994	2001	1843	1845	1939	2081	1830	1767
Wdr46	841	827	850	826	992	801	759	749
Wdr47	82	120	110	104	134	139	128	111
Wdr48	1313	1396	1483	1339	1486	1429	1383	1359
Wdr49	0	1	0	0	0	2	0	1
Wdr5	1436	1481	1541	1338	1605	1523	1435	1439
Wdr53	217	248	215	203	261	240	208	191

Transcriptome sequencing yielded total genetic results for the MOD and APS groups, with a total of 15,936 variables

Continued from above

Wdr54	5	6	5	6	12	12	7	10
Wdr55	387	441	416	341	418	376	412	386
Wdr59	303	309	284	202	265	229	212	262
Wdr5b	120	111	112	95	82	86	73	89
Wdr6	565	659	739	666	470	519	589	576
Wdr60	45	50	48	67	42	66	59	65
Wdr61	1124	1245	1210	1064	1138	1396	1170	1170
Wdr62	130	119	128	146	100	98	103	104
Wdr63	2	5	1	7	2	0	0	7
Wdr66	17	7	19	13	5	16	11	10
Wdr7	966	1122	955	974	983	1053	1081	959
Wdr70	403	414	394	444	471	431	414	462
Wdr72	0	0	1	0	1	0	2	1
Wdr73	260	333	320	318	291	385	299	276
Wdr74	543	594	535	426	620	532	624	505
Wdr75	438	549	398	447	502	534	541	454
Wdr76	293	301	314	321	329	303	242	255
Wdr77	1103	1142	1090	955	1119	1235	1008	1098
Wdr78	18	27	16	25	23	48	18	22
Wdr81	1089	1060	1114	1039	1061	1230	988	1052
Wdr82	3299	3716	3596	3483	3858	3751	3462	3446
Wdr83	558	606	557	541	584	567	553	565
Wdr83os	1089	1075	1061	981	1005	993	855	1003
Wdr86	20	18	9	14	13	3	7	2
Wdr88	0	0	0	1	0	0	0	0
Wdr89	834	805	851	827	950	911	790	1004
Wdr90	144	126	126	162	139	141	130	131
Wdr91	136	84	89	115	105	118	70	54
Wdr92	507	553	567	517	598	574	531	543
Wdr93	2	5	0	5	9	1	2	13
Wdsub1	306	362	313	300	353	210	306	241
Wdtc1	3091	3099	3002	2998	2790	2879	3024	2644
Wdyhv1	272	278	250	268	333	340	324	253
Wee1	572	532	626	542	677	643	632	556
Wee2	5	12	5	15	7	11	16	0
Wfdc1	139	101	142	145	186	152	189	145
Wfdc13	0	1	0	0	0	0	0	0
Wfdc15b	1	1	0	0	0	1	4	0
Wfdc17	296	224	205	261	147	197	152	162
Wfdc18	9	5	0	6	4	1	7	7
Wfdc2	6	15	14	4	15	9	4	12
Wfdc21	2	8	2	1	3	3	0	8
Wfdc3	10	6	5	5	2	1	5	6
Wfdc6a	4	0	0	0	0	0	0	0
Wfdc6b	0	0	0	1	0	0	0	0
Wfikkn1	9	20	15	26	7	4	24	17
Wfikkn2	18	9	21	27	16	11	24	11
Wfs1	1472	1600	1528	1516	1583	1492	1530	1515
Whamm	1131	1186	1099	919	968	1061	990	897
Whrn	294	279	299	234	306	310	248	307
Wif1	2	13	9	5	0	13	1	9
Wipf1	1427	1504	1499	1533	1447	1550	1449	1361
Wipf2	2380	2478	2353	2344	2248	2451	2401	2183
Wipf3	753	881	742	748	828	908	950	687
Wipi1	368	365	378	385	285	353	242	344
Wipi2	2214	2379	2207	2230	2359	2243	2325	2086
Wiz	1514	1401	1456	1374	1285	1420	1199	1201
Wls	545	699	625	557	655	585	562	492

Transcriptome sequencing yielded total genetic results for the MOD and APS groups, with a total of 15,936 variables

Continued from above

Wnk1	3136	3689	3419	3407	3591	3594	3517	3151
Wnk2	1004	972	959	1018	864	882	850	798
Wnk3	3	5	2	2	12	1	1	1
Wnk4	94	100	110	102	116	120	100	147
Wnt10a	0	1	0	0	0	0	0	4
Wnt10b	0	0	0	1	1	1	1	3
Wnt11	71	40	64	55	61	55	56	54
Wnt2	6	3	12	8	13	2	10	17
Wnt2b	174	185	243	186	191	216	222	174
Wnt3	55	46	38	40	47	30	29	37
Wnt4	268	236	217	198	266	359	336	319
Wnt5a	212	264	301	255	252	239	202	178
Wnt5b	54	35	10	21	23	14	11	37
Wnt6	70	41	56	66	58	55	57	45
Wnt7a	0	0	0	1	0	0	0	0
Wnt7b	1	0	0	1	1	1	0	0
Wnt9a	29	6	2	28	14	9	8	12
Wnt9b	16	19	15	16	12	24	6	11
Wrap53	437	492	593	519	505	513	469	513
Wrap73	292	394	390	334	400	308	303	287
Wrn	932	984	910	892	872	861	863	757
Wrnip1	1546	1550	1559	1525	1838	1771	1598	1611
Wsb1	1631	1809	1820	1756	1589	1942	1843	1693
Wsb2	3965	4181	4234	4124	4344	4238	4314	4229
Wscd1	41	30	18	29	17	35	20	25
Wscd2	64	76	103	71	65	52	79	63
Wt1	0	0	2	2	3	2	10	4
Wtap	3219	3277	3067	3410	2951	3024	2951	2803
Wtip	946	520	749	926	744	636	702	831
Wwc1	3522	3499	3342	3623	2950	3319	3286	3103
Wwc2	196	225	226	192	297	241	314	286
Wwox	291	189	280	252	210	257	189	197
Wwp1	649	721	682	681	664	640	635	705
Wwp2	3645	3965	3397	3177	3004	3211	3329	3229
Wwtr1	508	435	524	578	409	407	437	361
Xab2	1289	1300	1336	1203	1186	1184	1237	1219
Xaf1	1157	1532	1404	1080	1295	1251	1128	1055
Xbp1	12467	12955	12064	12013	12993	12840	12675	13122
Xcl1	7	16	16	15	12	1	3	4
Xcr1	3	2	9	1	1	3	8	2
Xdh	41648	46298	39670	37290	38833	41761	41227	36059
Xiap	4756	4958	4784	4860	5227	5644	5433	4995
Xirp1	1	4	1	6	0	0	1	4
Xk	808	1058	823	748	1079	1047	1050	978
Xkr4	12	9	2	5	8	1	11	3
Xkr5	8	16	23	12	13	22	7	1
Xkr6	12	14	10	15	13	16	16	8
Xkr7	7	2	6	5	4	7	16	6
Xkr8	213	213	226	150	234	235	226	223
Xkr9	1598	1721	1580	1718	1566	1621	1829	1700
Xkrx	4	16	4	21	7	10	3	10
Xlr	4	26	7	15	20	18	30	9
Xlr3a	29	10	10	51	5	6	13	20
Xlr3b	9	29	25	13	3	2	6	15
Xlr3c	0	7	0	0	0	0	1	0
Xlr4a	34	6	16	52	12	11	14	8
Xlr4b	5	0	6	3	2	7	15	0
Xlr4c	5	1	2	9	0	0	1	6

Transcriptome sequencing yielded total genetic results for the MOD and APS groups, with a total of 15,936 variables

Continued from above

Xlr5a	6	0	0	1	2	0	1	0
Xlr5b	1	0	0	0	0	0	0	0
Xlr5c	1	7	0	0	0	1	0	0
Xndc1	91	103	100	80	82	152	101	93
Xpa	252	198	230	240	218	249	203	242
Xpc	339	423	392	389	440	454	431	400
Xpnpep1	16235	17833	15493	14351	19106	20535	20015	18092
Xpnpep2	1430	1406	1358	1441	1168	1230	1238	1349
Xpnpep3	212	251	240	280	300	262	268	230
Xpo1	2815	3091	2671	2713	3294	3447	3313	3131
Xpo4	891	969	812	930	1021	994	909	851
Xpo5	1182	1241	1210	1106	1258	1229	1014	1210
Xpo6	2010	2253	2139	1972	2047	2004	1900	1884
Xpo7	2295	2470	2378	2389	2800	2741	2418	2399
Xpot	2465	2693	2522	2441	3017	3081	3059	2741
Xpr1	3096	3232	2954	2971	3162	3209	3353	2885
Xrcc1	620	621	696	642	639	664	622	665
Xrcc2	67	66	92	69	77	61	71	35
Xrcc3	234	256	226	235	206	199	154	160
Xrcc4	189	265	231	179	241	184	234	156
Xrcc5	335	407	334	379	382	319	331	328
Xrcc6	758	876	847	895	830	831	725	769
Xrn1	1653	1892	1884	1688	1413	1790	1624	1394
Xrn2	2486	2883	2846	2761	2415	2633	2628	2473
Xrra1	0	0	0	1	0	1	1	0
Xxylt1	685	458	545	580	585	567	479	509
Xylb	402	424	403	376	486	399	505	465
Xylt1	84	74	69	75	57	80	62	79
Xylt2	638	616	666	676	585	549	574	525
Yae1d1	301	370	395	396	481	363	417	450
Yaf2	353	333	336	370	407	443	416	414
Yap1	2316	2513	2418	2326	2793	2398	2389	2458
Yars	2081	2068	1859	2115	1949	2059	1869	1784
Yars2	659	718	676	632	696	679	600	643
Ybey	106	154	147	101	148	122	130	113
Ybx1	31225	32682	31751	30988	31949	31392	30515	31181
Ybx2	75	78	66	74	54	67	61	64
Ybx3	5659	5927	5653	5565	6106	5710	5294	5545
Ydjc	1032	1011	960	944	1162	1068	1118	984
Yeats2	677	782	638	698	739	738	624	588
Yeats4	1029	1119	1062	1136	1166	1091	1099	939
Yes1	1494	1566	1429	1558	1720	1768	1751	1528
Yif1a	3691	4045	3570	3780	3758	3738	3733	3944
Yif1b	501	567	523	420	538	568	483	466
Yipf1	2559	2566	2464	2350	2638	2549	2579	2444
Yipf2	866	896	941	934	883	921	840	895
Yipf3	3102	3209	2771	2671	3090	3146	2863	3011
Yipf4	2467	2509	2486	2370	2730	2769	2669	2701
Yipf5	3301	3590	3041	3184	3698	3830	3636	3625
Yipf6	1871	1997	1787	1897	2008	1972	1966	1780
Yjefn3	14	8	12	8	8	15	1	6
Yju2	305	288	279	288	309	256	235	283
Ykt6	8298	8699	7931	8104	8625	9115	8764	8710
Ylpm1	894	987	1075	1083	1008	1027	989	907
Yme1l1	4117	4967	4372	4432	4959	5185	5293	4776
Yod1	255	311	303	292	325	268	267	324
Ypel1	5	6	17	14	10	2	8	15
Ypel2	924	1242	1014	794	1251	1160	1012	1074

Transcriptome sequencing yielded total genetic results for the MOD and APS groups, with a total of 15,936 variables

Continued from above

Ypel3	516	474	501	507	432	435	485	478
Ypel4	3	10	10	5	6	6	5	6
Ypel5	1397	1502	1395	1348	1513	1554	1585	1404
Yrdc	889	746	837	797	827	816	661	772
Ythdc1	1444	1431	1424	1265	1650	1636	1398	1464
Ythdc2	910	926	842	818	932	860	802	765
Ythdf1	1699	1804	1876	1705	1755	1625	1655	1674
Ythdf2	2102	2193	2184	2079	2255	2306	2177	2242
Ythdf3	3300	3626	3567	3397	3701	3647	3838	3498
Ywhab	8586	8885	8741	8467	9240	9242	8875	8146
Ywhae	12348	12732	12411	12123	12841	12878	12016	11592
Ywhag	2883	3232	2888	3007	3267	3139	2794	3032
Ywhah	8434	9031	8811	8633	8946	8936	8339	8119
Ywhaq	4739	4930	4587	4877	4897	4949	4615	4595
Ywhaz	15828	16377	15968	16071	15917	15522	15709	15507
Yy1	3346	3443	3382	3365	3617	3610	3367	3180
Yy2	73	44	68	58	68	52	73	96
Zadh2	1943	2007	2075	2032	1979	2056	1829	1790
Zan	157	219	139	178	133	163	102	156
Zap70	97	92	83	77	68	78	60	62
Zar1	0	0	0	0	0	0	1	1
Zbed3	445	485	472	439	446	484	411	464
Zbed4	553	634	602	589	626	635	633	560
Zbed5	305	300	347	317	283	298	214	268
Zbed6	1039	1317	1149	1355	1274	1246	1347	1029
Zbp1	7033	7828	6953	6412	6268	6528	6117	5613
Zbtb1	436	450	448	433	584	515	493	396
Zbtb10	44	33	30	27	11	27	31	23
Zbtb11	717	694	689	729	778	753	766	760
Zbtb12	132	109	105	85	106	68	115	104
Zbtb14	263	330	309	278	313	359	334	294
Zbtb16	17	38	47	47	37	40	31	35
Zbtb17	850	920	872	965	965	839	850	859
Zbtb18	1286	1308	1268	1454	1303	1427	1091	1158
Zbtb2	375	387	403	414	439	355	359	356
Zbtb20	274	343	309	296	348	396	318	267
Zbtb21	492	451	491	461	540	522	488	477
Zbtb22	1730	1657	1658	1553	1394	1556	1461	1461
Zbtb24	843	1003	859	911	1017	1017	1005	877
Zbtb25	70	84	88	135	73	106	76	85
Zbtb26	171	187	206	135	211	158	214	190
Zbtb3	40	33	30	59	69	50	51	52
Zbtb32	8	2	12	18	5	2	0	8
Zbtb33	851	975	913	1051	1129	1063	1002	954
Zbtb34	220	279	270	258	276	252	214	230
Zbtb37	253	240	236	181	234	225	258	263
Zbtb38	793	792	776	756	921	883	908	799
Zbtb39	708	919	910	771	818	765	779	813
Zbtb4	579	537	539	523	482	562	486	442
Zbtb40	650	652	595	598	814	858	736	702
Zbtb41	550	653	603	683	666	768	676	662
Zbtb42	699	838	980	809	791	653	726	684
Zbtb43	1216	1217	1404	1222	1353	1323	1247	1200
Zbtb44	543	565	530	632	618	604	578	554
Zbtb45	499	515	548	459	512	534	458	474
Zbtb46	35	40	42	36	57	78	65	49
Zbtb48	210	206	233	274	210	211	186	187
Zbtb49	162	165	137	215	120	164	166	128

Transcriptome sequencing yielded total genetic results for the MOD and APS groups, with a total of 15,936 variables

Continued from above

Zbtb5	376	557	371	381	410	353	381	355
Zbtb6	1042	1154	977	1059	1151	1156	1070	954
Zbtb7a	8174	8249	8534	8861	8040	8551	8440	7818
Zbtb7b	11388	11705	11014	10899	11952	12032	11721	10817
Zbtb7c	35	12	12	8	12	11	18	23
Zbtb8a	299	342	301	319	307	331	317	269
Zbtb8b	0	0	1	0	0	0	0	0
Zbtb8os	887	978	879	958	1088	1064	961	975
Zbtb9	488	511	494	394	482	468	511	503
Zc2hc1a	90	84	106	115	94	105	98	98
Zc2hc1c	9	3	6	8	12	7	14	8
Zc3h10	525	479	571	573	560	638	517	573
Zc3h11a	3388	3716	3891	3824	4092	4018	3724	3810
Zc3h12a	2794	2992	2776	2623	3216	3257	2972	3048
Zc3h12b	8	12	7	10	9	5	0	17
Zc3h12c	51	50	28	34	35	44	70	49
Zc3h12d	931	1068	1020	987	932	937	808	852
Zc3h13	1049	1262	1029	1077	1222	1075	1195	1023
Zc3h14	1604	1766	1702	1573	1631	1792	1579	1560
Zc3h15	2652	2998	2635	2733	3109	3307	3069	2867
Zc3h18	2841	3233	2944	2848	3054	3015	2859	2990
Zc3h3	835	802	733	829	736	857	734	671
Zc3h4	3566	3764	3541	3225	4140	4092	3730	3514
Zc3h6	50	82	53	81	29	29	44	51
Zc3h7a	2586	2742	2580	2531	2619	2504	2591	2536
Zc3h7b	891	1062	1069	992	785	838	823	791
Zc3h8	27	55	63	51	56	49	38	55
Zc3hav1	5037	5684	5216	4796	5186	5406	5245	4848
Zc3hav1l	62	85	85	45	42	57	49	32
Zc3hc1	302	335	345	316	327	283	328	270
Zc4h2	12	20	22	17	29	23	12	22
Zcchc10	133	163	103	126	145	154	153	143
Zcchc12	25	20	36	21	30	29	24	22
Zcchc14	2152	2356	2192	2403	2069	2187	2222	2110
Zcchc17	840	917	821	829	998	998	877	821
Zcchc18	34	45	56	48	24	53	56	21
Zcchc2	741	946	851	802	909	830	721	717
Zcchc24	428	431	466	454	455	446	480	343
Zcchc3	55	72	48	68	50	54	38	43
Zcchc4	396	493	435	401	466	470	380	399
Zcchc7	402	473	423	419	488	505	547	445
Zcchc8	1343	1489	1653	1522	1553	1489	1493	1289
Zcchc9	627	725	762	639	622	740	665	698
Zcrb1	1250	1207	1228	1190	1125	1307	1306	1245
Zcwpw1	16	11	18	6	9	20	7	10
Zcwpw2	0	2	0	2	2	3	0	1
Zdbf2	1	1	0	1	0	2	2	6
Zdhhc1	203	169	139	200	199	154	181	156
Zdhhc11	0	0	0	0	0	0	1	1
Zdhhc12	910	989	935	918	1070	1022	893	1035
Zdhhc13	1779	1830	1955	1722	2123	2116	1885	1960
Zdhhc14	196	141	166	121	170	168	113	151
Zdhhc15	336	263	340	341	372	311	374	328
Zdhhc16	720	774	736	682	608	757	646	667
Zdhhc17	798	688	723	795	781	846	729	734
Zdhhc18	668	903	912	833	874	861	801	876
Zdhhc19	12	13	17	6	2	11	15	15
Zdhhc2	702	892	761	823	859	887	757	776

Transcriptome sequencing yielded total genetic results for the MOD and APS groups, with a total of 15,936 variables

Continued from above

Zdhhc20	2007	2056	2140	2126	1940	2113	2068	1927
Zdhhc21	1632	1749	1698	1806	1831	1867	1794	1756
Zdhhc22	1	0	0	0	6	4	0	0
Zdhhc23	819	799	903	867	909	773	744	762
Zdhhc24	313	370	355	312	367	361	381	378
Zdhhc3	6346	6810	6505	6137	6127	6019	5710	5958
Zdhhc4	612	565	630	556	545	509	495	480
Zdhhc5	6417	7195	6547	6703	6786	7068	6585	6113
Zdhhc6	1012	1119	1039	942	1066	992	967	951
Zdhhc7	7125	7248	6999	7166	7552	7540	7196	7063
Zdhhc8	160	140	172	141	192	98	109	153
Zdhhc9	3941	4277	3707	3526	4208	4026	4226	3949
Zeb1	132	207	148	250	118	114	152	166
Zeb2	402	450	363	454	438	367	308	381
Zer1	1025	1087	1036	1227	938	1025	1074	895
Zfand1	550	530	581	600	443	470	480	516
Zfand2a	608	600	505	541	743	695	946	866
Zfand2b	1129	977	1045	1122	945	879	820	1027
Zfand3	2563	2663	2539	2651	2733	2567	2528	2435
Zfand4	20	15	16	19	25	18	3	23
Zfand5	2927	3135	2801	2901	2923	3103	2815	3000
Zfand6	4281	4668	4375	4432	4691	4689	4577	4080
Zfat	105	167	137	122	131	131	151	135
Zfc3h1	1470	1526	1668	1707	1807	1844	1696	1551
Zfhx2	200	296	256	247	365	338	262	335
Zfhx3	126	139	138	135	140	101	150	142
Zfhx4	8	21	13	9	15	13	7	16
Zfp1	143	151	165	169	166	151	125	130
Zfp101	164	216	151	196	195	182	193	197
Zfp105	26	15	24	35	16	15	11	38
Zfp106	6242	7042	6478	6730	6548	6644	6427	6201
Zfp108	13	36	12	17	29	24	15	16
Zfp109	53	79	70	55	52	66	75	61
Zfp11	67	48	57	47	30	37	52	40
Zfp110	888	1072	966	1019	1029	1069	975	1007
Zfp111	140	170	134	141	124	132	146	103
Zfp112	58	41	45	33	37	56	30	33
Zfp113	310	366	222	316	284	315	272	298
Zfp114	1	1	0	0	0	4	0	0
Zfp119a	64	76	78	69	46	79	85	76
Zfp119b	89	87	70	65	87	79	70	53
Zfp12	97	105	133	123	113	144	77	111
Zfp120	347	402	371	322	412	411	404	410
Zfp128	25	6	13	22	5	15	25	13
Zfp13	78	101	76	89	74	88	57	73
Zfp131	699	736	724	636	753	816	786	552
Zfp14	34	29	29	17	38	34	22	35
Zfp141	126	162	109	131	162	209	184	183
Zfp142	406	415	394	436	425	472	403	402
Zfp143	663	709	764	653	690	787	805	730
Zfp146	615	655	666	639	716	676	703	669
Zfp148	1769	1831	1743	1689	1885	1988	1672	1652
Zfp157	177	178	202	197	178	213	174	171
Zfp160	375	383	333	350	355	358	406	326
Zfp169	582	612	637	660	543	547	530	515
Zfp174	69	38	29	46	18	52	58	39
Zfp180	556	608	545	608	610	644	514	534
Zfp182	181	197	172	205	168	177	140	203

Transcriptome sequencing yielded total genetic results for the MOD and APS groups, with a total of 15,936 variables

Continued from above

Zfp184	10	26	13	13	21	24	34	11
Zfp185	2	7	19	1	15	5	2	17
Zfp189	33	22	27	53	17	40	48	32
Zfp2	13	26	35	24	59	50	38	22
Zfp202	23	20	30	14	17	41	29	28
Zfp207	4241	4232	4311	4340	4819	4327	4030	3970
Zfp212	448	386	358	416	310	419	347	317
Zfp213	360	411	428	394	417	427	421	401
Zfp217	1517	1638	1555	1584	1603	1520	1416	1539
Zfp219	1617	1696	1722	1685	1801	1611	1428	1521
Zfp229	28	68	67	76	42	63	47	62
Zfp235	127	170	167	151	183	149	188	145
Zfp236	1277	1459	1422	1270	1314	1284	1280	1243
Zfp239	344	405	353	346	417	427	379	368
Zfp24	851	1114	943	1025	1199	1043	1100	1085
Zfp248	18	11	2	5	10	5	6	5
Zfp251	146	193	157	177	166	164	145	158
Zfp26	449	551	595	555	473	583	556	532
Zfp260	817	987	903	939	1006	1139	1097	1026
Zfp263	628	603	638	664	643	699	548	630
Zfp266	868	1000	863	881	999	921	891	911
Zfp267	228	157	156	179	155	200	184	196
Zfp27	237	283	227	241	263	245	272	239
Zfp273	113	95	82	93	114	126	131	99
Zfp275	305	267	306	329	367	302	288	321
Zfp276	639	616	708	659	736	701	638	666
Zfp277	596	733	679	651	634	673	647	627
Zfp28	28	42	35	47	14	35	23	42
Zfp280b	708	817	866	704	751	749	678	696
Zfp280c	178	161	164	192	125	233	195	115
Zfp280d	378	406	417	407	476	430	410	413
Zfp281	298	324	284	344	342	346	350	328
Zfp282	638	664	630	580	587	619	574	538
Zfp286	8	8	12	2	5	3	2	7
Zfp287	78	98	87	88	59	98	71	60
Zfp292	1273	1301	1309	1338	1541	1622	1478	1287
Zfp296	18	31	44	20	26	31	8	43
Zfp3	92	95	153	89	77	89	109	106
Zfp30	10	13	12	4	14	22	6	11
Zfp300	1	2	6	5	4	3	6	10
Zfp316	95	88	94	62	56	40	48	56
Zfp317	412	398	461	399	451	558	421	398
Zfp318	212	211	257	251	272	270	272	305
Zfp319	512	557	455	541	530	486	434	497
Zfp322a	404	442	553	466	547	568	544	463
Zfp324	289	299	267	221	267	293	296	241
Zfp326	565	615	535	497	615	660	516	538
Zfp329	146	168	201	198	186	180	178	158
Zfp330	663	748	688	714	862	780	774	732
Zfp334	25	44	22	18	28	30	9	12
Zfp335	1287	1285	1254	1181	1184	1200	1112	1028
Zfp341	147	141	160	120	149	113	122	175
Zfp345	0	0	0	0	0	4	0	1
Zfp346	258	251	184	213	266	225	222	243
Zfp35	605	730	676	623	730	713	665	748
Zfp354a	41	51	55	46	49	27	25	29
Zfp354b	1	3	0	3	3	2	8	1
Zfp354c	21	15	34	11	36	24	28	38

Transcriptome sequencing yielded total genetic results for the MOD and APS groups, with a total of 15,936 variables

Continued from above

Zfp358	237	238	225	264	173	225	254	186
Zfp36	5694	6099	6040	5612	5170	5639	5813	5382
Zfp362	397	388	377	321	366	274	333	282
Zfp365	8	2	7	7	2	22	4	9
Zfp366	28	51	27	8	44	30	28	21
Zfp367	393	364	401	399	338	410	382	345
Zfp369	38	96	73	112	59	69	47	59
Zfp36l1	1770	1939	1898	1800	1817	1850	1671	1746
Zfp36l2	4807	5071	4952	4551	5363	5254	4759	4781
Zfp36l3	0	0	1	0	0	0	0	0
Zfp37	117	128	102	114	75	76	112	97
Zfp382	13	17	36	25	34	13	5	8
Zfp383	36	62	56	49	29	77	49	73
Zfp384	2044	2347	2133	2041	2146	2015	2103	2016
Zfp385a	119	126	137	119	144	119	139	131
Zfp385b	83	90	108	82	56	85	104	88
Zfp385c	95	134	129	140	104	111	124	105
Zfp386	419	510	374	480	502	483	513	503
Zfp39	37	41	48	33	62	50	68	57
Zfp395	376	318	425	396	311	309	327	300
Zfp397	501	628	575	569	637	700	669	600
Zfp398	327	378	377	361	339	361	379	336
Zfp40	35	50	20	18	40	57	35	40
Zfp407	597	667	616	727	649	647	536	557
Zfp408	411	453	401	458	383	373	384	348
Zfp41	57	93	79	79	77	67	107	47
Zfp410	1742	2137	1802	1744	1992	2063	2022	1792
Zfp414	276	249	271	287	228	212	216	175
Zfp418	34	50	59	47	27	43	44	41
Zfp420	36	50	39	40	24	49	46	51
Zfp422	597	680	742	674	584	641	583	570
Zfp423	44	23	23	18	17	10	26	25
Zfp426	418	497	429	503	475	450	488	416
Zfp428	64	108	69	59	102	91	62	85
Zfp429	196	253	155	172	262	227	185	200
Zfp433	23	9	14	23	13	25	15	28
Zfp438	27	72	46	67	66	46	41	49
Zfp442	214	235	228	302	262	276	247	262
Zfp444	341	365	306	308	319	260	262	267
Zfp445	2300	2689	2538	2480	2612	2755	2525	2444
Zfp446	75	72	84	70	64	70	88	62
Zfp449	11	28	28	14	9	6	10	12
Zfp451	188	216	235	211	283	229	243	224
Zfp454	0	7	3	1	4	0	2	4
Zfp455	37	39	40	33	45	38	24	29
Zfp456	106	96	102	112	94	115	108	84
Zfp457	1	0	0	1	2	5	1	5
Zfp458	27	60	30	39	47	46	55	35
Zfp459	46	35	54	55	49	57	67	33
Zfp46	346	445	465	458	447	429	411	424
Zfp462	94	222	141	133	112	134	130	137
Zfp467	143	203	158	168	141	125	128	116
Zfp469	8	6	12	15	27	16	20	23
Zfp472	211	233	275	227	225	242	252	218
Zfp473	15	13	22	30	46	24	20	28
Zfp474	0	1	0	0	0	0	0	0
Zfp493	42	52	25	23	29	22	25	25
Zfp503	160	178	163	200	199	226	156	145

Transcriptome sequencing yielded total genetic results for the MOD and APS groups, with a total of 15,936 variables

Continued from above

Zfp507	703	782	737	693	756	770	762	836
Zfp51	217	218	172	164	222	245	266	232
Zfp511	280	296	338	313	299	247	270	292
Zfp512	404	447	529	521	489	519	405	475
Zfp512b	363	446	431	415	275	314	289	306
Zfp513	823	1040	948	966	864	975	837	754
Zfp516	678	668	771	730	587	577	609	553
Zfp518a	602	654	584	577	728	778	703	639
Zfp518b	81	64	86	61	81	68	84	66
Zfp52	62	59	47	40	53	60	56	35
Zfp521	24	29	44	44	28	25	42	30
Zfp523	307	324	357	302	284	309	293	302
Zfp524	530	494	593	567	564	564	529	588
Zfp526	746	768	787	813	709	601	611	639
Zfp53	424	471	394	468	549	500	495	443
Zfp532	59	55	67	55	70	60	53	42
Zfp534	6	3	0	0	0	2	0	2
Zfp536	10	14	21	10	13	10	5	9
Zfp54	139	163	141	146	128	166	159	160
Zfp551	27	14	24	21	18	30	24	21
Zfp553	377	466	429	386	411	374	401	333
Zfp558	26	24	21	14	38	13	16	26
Zfp560	85	77	71	70	82	63	70	88
Zfp563	137	170	109	148	152	143	146	136
Zfp566	16	26	23	11	21	15	11	12
Zfp568	407	461	509	490	494	544	456	481
Zfp57	23	29	10	21	20	11	16	29
Zfp574	713	697	816	756	817	955	716	720
Zfp575	7	14	13	5	1	8	0	1
Zfp579	137	101	143	117	95	84	92	104
Zfp58	118	123	103	127	134	158	115	114
Zfp580	32	24	24	28	15	16	14	47
Zfp583	0	6	5	7	2	1	4	2
Zfp59	148	142	135	73	98	130	125	90
Zfp592	1762	2036	2052	2034	1970	2147	1859	1848
Zfp593	329	350	288	344	393	277	236	293
Zfp595	85	119	86	110	117	172	113	129
Zfp597	264	315	353	317	330	385	299	327
Zfp598	2713	2687	2626	2651	2774	2871	2632	2593
Zfp599	109	127	103	101	111	110	96	111
Zfp60	180	183	183	170	169	212	182	207
Zfp605	52	60	40	48	35	93	68	49
Zfp606	159	229	203	177	171	222	198	143
Zfp607a	47	75	36	35	40	45	72	39
Zfp607b	5	7	20	9	8	9	15	8
Zfp608	611	618	698	727	622	536	510	509
Zfp609	1196	1245	1224	1227	1026	1062	1072	1007
Zfp61	43	16	28	43	54	38	42	28
Zfp612	143	144	128	97	120	117	160	141
Zfp617	675	649	639	686	648	710	720	608
Zfp618	39	29	27	27	42	30	42	19
Zfp619	22	19	35	34	22	30	35	34
Zfp62	400	416	418	382	416	403	419	429
Zfp622	1666	1857	1674	1699	1891	1808	1768	1822
Zfp623	354	367	364	293	466	393	353	407
Zfp626	288	281	338	311	322	330	261	338
Zfp628	660	635	609	550	577	576	610	574
Zfp629	630	638	775	719	553	626	576	549

Transcriptome sequencing yielded total genetic results for the MOD and APS groups, with a total of 15,936 variables

Continued from above

Zfp637	348	396	368	393	409	355	379	359
Zfp638	981	1070	1022	1027	1404	1467	1368	1185
Zfp639	443	442	488	482	527	451	548	466
Zfp64	519	537	563	432	563	489	442	459
Zfp641	7	16	10	5	9	13	14	10
Zfp644	552	540	495	490	544	545	580	450
Zfp646	1472	1484	1702	1458	1569	1735	1446	1322
Zfp647	2	22	6	18	16	8	11	18
Zfp65	246	265	248	225	283	272	256	278
Zfp651	138	150	173	168	146	172	163	138
Zfp652	544	583	532	504	566	547	470	499
Zfp653	160	108	172	134	124	148	178	162
Zfp654	589	563	616	692	668	769	672	595
Zfp655	1107	1291	1252	1260	1317	1379	1313	1275
Zfp658	38	50	42	43	28	31	34	31
Zfp660	0	0	5	0	4	1	4	0
Zfp661	84	78	43	52	84	73	62	89
Zfp663	0	0	0	0	0	0	0	1
Zfp664	1555	1667	1612	1704	1864	1923	1616	1589
Zfp667	13	14	4	3	13	24	17	17
Zfp668	251	304	285	246	316	297	330	325
Zfp672	1016	1022	1003	995	1133	1090	855	988
Zfp677	21	33	12	14	23	16	17	19
Zfp68	375	380	396	404	536	532	568	465
Zfp687	1412	1470	1490	1444	1439	1495	1383	1367
Zfp688	335	363	301	304	271	295	313	356
Zfp689	123	154	123	143	113	96	82	117
Zfp69	22	43	34	43	41	29	33	17
Zfp691	93	110	109	120	126	143	108	139
Zfp692	246	281	226	259	278	335	242	257
Zfp697	4	14	26	32	17	25	16	10
Zfp7	319	307	397	402	375	432	287	422
Zfp703	2304	2303	2470	2406	2285	2202	2214	2274
Zfp704	908	897	1022	957	926	955	958	835
Zfp706	7876	7574	7573	8073	7300	7653	7058	7096
Zfp707	273	232	204	249	213	196	200	199
Zfp708	38	35	41	35	67	52	28	22
Zfp709	116	95	100	101	107	89	89	111
Zfp710	2868	2828	2604	2455	2535	2501	2546	2381
Zfp711	8	8	7	21	10	4	17	7
Zfp712	45	40	24	21	46	34	32	39
Zfp715	329	342	342	341	434	377	470	353
Zfp719	270	326	267	279	340	418	336	295
Zfp72	34	18	22	28	48	21	35	15
Zfp729a	307	356	310	373	329	431	430	360
Zfp729b	352	354	390	402	406	401	450	334
Zfp738	181	153	134	181	175	203	183	163
Zfp74	55	41	39	41	28	32	45	28
Zfp740	1866	2010	1815	1814	1863	1848	1730	1714
Zfp746	757	851	669	726	753	772	710	713
Zfp747	1323	1461	1371	1216	1250	1326	1280	1205
Zfp748	277	240	345	249	308	329	345	285
Zfp750	0	2	10	7	1	1	0	4
Zfp758	311	295	317	300	332	323	351	329
Zfp759	41	39	33	58	52	62	45	63
Zfp760	71	99	116	67	113	133	102	155
Zfp763	70	85	124	94	94	99	96	97
Zfp764	262	228	261	264	250	237	263	287

Transcriptome sequencing yielded total genetic results for the MOD and APS groups, with a total of 15,936 variables

Continued from above

Zfp768	1743	1683	1646	1820	1643	1605	1801	1500
Zfp770	180	259	219	219	265	302	237	221
Zfp771	387	394	396	424	374	349	424	345
Zfp772	32	70	94	66	85	91	54	73
Zfp773	4	7	2	4	9	7	5	2
Zfp775	65	60	62	50	55	47	42	46
Zfp777	638	671	758	639	694	554	603	563
Zfp78	0	9	4	2	1	2	7	3
Zfp780b	171	197	186	207	249	239	238	162
Zfp781	4	5	1	7	7	5	4	0
Zfp784	211	181	196	204	184	211	173	159
Zfp786	30	26	18	15	14	23	12	8
Zfp787	819	826	781	839	866	854	784	840
Zfp788	154	104	110	149	97	127	138	124
Zfp790	363	364	303	369	379	329	332	350
Zfp791	21	62	38	50	35	34	38	37
Zfp799	338	314	272	314	227	265	248	220
Zfp800	574	649	811	666	900	814	707	739
Zfp804a	6	4	8	6	11	11	1	2
Zfp808	13	20	47	21	51	39	20	27
Zfp809	380	474	542	603	573	568	504	520
Zfp81	185	164	144	115	194	207	151	194
Zfp810	194	253	174	239	170	151	205	160
Zfp811	0	5	16	10	5	9	6	2
Zfp819	0	0	1	5	4	2	5	5
Zfp82	16	20	28	28	20	9	15	17
Zfp820	129	172	173	168	163	197	162	158
Zfp821	163	159	224	156	126	168	137	158
Zfp825	243	265	207	189	274	234	242	213
Zfp827	1017	1048	1015	1047	1198	974	1015	920
Zfp830	551	520	573	508	517	575	523	480
Zfp831	8	8	6	19	18	9	5	10
Zfp839	226	287	236	223	230	243	260	235
Zfp84	531	594	551	589	721	620	658	468
Zfp846	168	162	150	179	177	162	140	121
Zfp85	110	75	105	84	126	132	115	119
Zfp850	92	84	37	86	82	77	82	60
Zfp853	2	2	8	0	1	0	2	0
Zfp865	628	624	686	662	589	608	490	520
Zfp866	398	427	509	380	456	472	375	388
Zfp867	70	113	87	87	48	67	82	85
Zfp868	445	467	426	434	444	443	468	502
Zfp869	711	717	752	676	741	657	740	711
Zfp87	214	266	228	225	213	216	179	192
Zfp870	192	266	260	262	253	253	269	211
Zfp871	1918	2163	2218	2175	1863	1861	2073	1876
Zfp872	2	2	2	1	6	0	1	5
Zfp873	106	93	97	80	92	88	92	73
Zfp874a	103	134	153	153	138	153	140	132
Zfp874b	75	93	85	93	114	79	64	92
Zfp879	0	2	1	6	5	1	1	13
Zfp882	29	42	58	16	49	37	12	39
Zfp9	51	58	44	52	63	40	44	54
Zfp90	74	62	78	68	63	95	82	81
Zfp91	6310	7174	7091	6683	6782	7210	6626	6094
Zfp92	66	75	65	67	48	38	57	37
Zfp93	92	68	51	53	78	56	71	65
Zfp930	171	222	160	150	200	175	172	183

Transcriptome sequencing yielded total genetic results for the MOD and APS groups, with a total of 15,936 variables

Continued from above

Zfp931	101	95	75	105	128	94	99	129
Zfp932	152	174	144	130	186	198	144	122
Zfp933	140	233	209	247	221	253	191	214
Zfp934	39	44	24	29	50	65	43	47
Zfp935	172	160	178	180	192	245	220	167
Zfp937	247	242	212	235	273	217	291	267
Zfp938	89	85	71	78	60	68	57	71
Zfp939	96	52	81	94	81	96	76	79
Zfp94	27	21	18	25	25	18	26	25
Zfp940	8	20	13	16	11	8	17	15
Zfp941	9	16	8	14	6	10	15	12
Zfp942	399	489	393	464	506	515	543	424
Zfp943	335	338	419	390	460	414	410	447
Zfp944	323	309	347	401	342	366	339	332
Zfp945	130	200	117	139	99	121	120	117
Zfp946	751	795	752	717	798	825	822	727
Zfp947	36	45	65	61	39	63	59	39
Zfp948	219	255	219	231	268	271	259	287
Zfp949	139	144	155	152	108	93	124	120
Zfp950	567	602	585	634	730	734	699	696
Zfp951	33	38	23	20	14	52	10	44
Zfp952	363	396	345	379	353	326	333	327
Zfp953	145	169	186	114	167	209	164	198
Zfp954	486	536	477	523	531	601	481	483
Zfp955a	327	273	262	296	313	324	286	324
Zfp955b	296	292	252	270	280	305	321	309
Zfp956	245	146	183	214	203	222	197	181
Zfp958	152	136	135	169	163	198	157	187
Zfp959	75	115	92	103	116	93	105	114
Zfp960	24	26	19	36	18	35	39	21
Zfp961	760	728	767	684	830	747	694	743
Zfp963	146	129	123	137	152	146	131	129
Zfp964	6	15	3	1	17	3	5	6
Zfp965	4	36	0	0	8	4	6	3
Zfp966	8	3	13	2	5	1	1	2
Zfp967	0	1	0	0	0	10	0	1
Zfp968	0	2	1	0	1	0	1	1
Zfp97	119	109	122	143	90	125	129	121
Zfp970	152	175	183	164	170	196	159	146
Zfp971	404	398	434	493	485	579	541	419
Zfp972	25	25	20	18	31	18	23	18
Zfp973	8	0	0	2	0	1	7	1
Zfp974	73	73	101	69	86	95	112	105
Zfp975	50	35	30	48	61	13	37	79
Zfp976	27	73	53	38	79	36	59	40
Zfp977	9	12	6	8	1	10	4	0
Zfp978	0	0	0	0	1	0	0	0
Zfp979	2	2	4	8	17	7	9	10
Zfp980	0	0	0	0	0	0	0	4
Zfp982	13	16	6	2	5	5	20	8
Zfp983	141	158	154	191	203	233	208	173
Zfp984	331	444	346	385	472	376	454	397
Zfp985	5	2	0	1	0	4	1	0
Zfp987	8	31	17	25	21	10	16	8
Zfp988	0	0	0	1	0	0	0	0
Zfp990	0	0	1	1	0	0	0	0
Zfp991	50	64	47	44	53	66	69	45
Zfp992	40	52	48	60	51	54	60	28

Transcriptome sequencing yielded total genetic results for the MOD and APS groups, with a total of 15,936 variables

Continued from above

Zfp994	253	313	246	305	285	283	270	342
Zfp995	196	246	253	240	247	201	287	293
Zfp996	1	0	0	2	1	1	0	0
Zfpl1	1088	1121	979	1102	1008	1006	1051	1030
Zfpm1	7357	7541	7084	6705	7431	7432	6982	6679
Zfpm2	12	5	10	2	1	12	6	7
Zfr	2544	2579	2352	2549	2905	2943	3096	2555
Zfr2	100	105	101	73	147	142	114	109
Zfx	1070	1093	1025	931	1128	1151	1054	1008
Zfyve1	2547	2606	2391	1951	2548	2593	2426	2329
Zfyve16	458	518	540	536	583	622	586	488
Zfyve19	1401	1495	1391	1440	1245	1366	1270	1238
Zfyve21	1886	2226	2248	2131	2134	2162	2160	2145
Zfyve26	1043	1100	1094	1056	1010	1049	1119	1058
Zfyve27	929	1042	1001	965	926	902	947	920
Zfyve28	21	7	16	17	23	20	15	4
Zfyve9	1018	1332	1179	1109	1208	1272	1160	1100
Zg16	60366	60483	62701	64547	56510	59522	59298	56463
Zglp1	2	4	10	8	9	6	6	6
Zgpat	880	964	767	938	951	967	887	913
Zgrf1	66	88	72	89	108	89	85	82
Zhx1	1182	1126	1127	1103	1298	1317	1180	1119
Zhx2	673	798	727	785	681	721	800	743
Zhx3	348	347	364	377	409	355	432	298
Zik1	1	12	13	10	5	6	8	17
Zim1	5	0	4	6	0	0	1	1
Zkscan1	1170	1354	1179	1216	1280	1279	1279	1200
Zkscan14	348	309	298	245	320	343	327	328
Zkscan16	1	5	4	0	0	5	0	0
Zkscan17	504	551	525	621	492	433	460	404
Zkscan2	1	2	4	4	0	0	4	3
Zkscan3	1377	1411	1334	1452	1533	1578	1347	1363
Zkscan4	0	7	7	4	6	4	10	8
Zkscan5	430	400	339	404	370	342	380	414
Zkscan6	397	468	364	342	460	348	313	340
Zkscan7	146	215	191	198	195	215	182	235
Zkscan8	262	247	271	355	308	297	228	247
Zmat1	17	32	12	27	17	24	42	15
Zmat2	1578	1810	1653	1683	1885	1683	1673	1720
Zmat3	624	656	621	600	663	573	677	692
Zmat4	23	29	50	42	16	29	35	45
Zmat5	562	510	560	549	557	536	478	535
Zmiz1	5545	5919	5325	5519	4966	5569	5812	5192
Zmiz2	4210	4218	3876	4052	3945	3828	3744	3472
Zmpste24	1728	1729	1540	1622	1973	1795	1685	1657
Zmym1	123	150	185	161	188	122	159	114
Zmym2	1038	1149	1106	1047	1077	1119	1089	1039
Zmym3	468	608	530	555	436	543	345	391
Zmym4	276	346	315	376	298	359	307	245
Zmym5	1029	1096	1104	1089	1133	1247	1145	1044
Zmym6	181	196	135	215	248	187	227	200
Zmynd10	2	1	1	1	8	1	1	1
Zmynd11	1619	1789	1728	1600	1561	1705	1602	1549
Zmynd12	5	0	2	1	2	1	0	0
Zmynd15	116	95	82	101	85	105	87	93
Zmynd19	509	592	487	597	677	621	573	597
Zmynd8	1449	1680	1554	1642	1743	1580	1599	1497
Znfx1	6258	7870	7000	5635	6095	6530	6146	5720

Transcriptome sequencing yielded total genetic results for the MOD and APS groups, with a total of 15,936 variables

Continued from above

Znhit1	1477	1421	1542	1494	1439	1499	1378	1409
Znhit2	941	1052	896	828	842	907	821	920
Znhit3	240	275	272	244	291	325	290	273
Znhit6	118	152	164	155	159	167	141	137
Znrd1	464	530	508	518	490	512	433	475
Znrd1as	130	130	99	147	163	114	94	134
Znrd2	318	273	278	239	281	279	227	239
Znrf1	337	380	374	391	490	405	356	334
Znrf2	2227	2285	2152	2209	2091	2086	2128	2030
Znrf3	181	208	203	171	225	209	193	220
Zp2	1	5	0	0	1	0	4	1
Zpbp	0	0	0	0	0	6	0	0
Zpbp2	8	15	3	1	13	5	6	15
Zpld1	0	1	0	0	0	0	0	1
Zpr1	905	965	837	810	953	816	816	802
Zranb1	637	644	639	641	597	698	723	625
Zranb2	1248	1498	1210	1240	1489	1569	1409	1316
Zranb3	71	104	106	88	91	88	96	105
Zrsr1	168	172	223	212	214	214	200	177
Zrsr2	458	473	558	526	544	499	431	433
Zscan12	100	114	99	91	125	137	99	71
Zscan18	5	11	4	13	2	10	0	2
Zscan2	30	33	36	22	52	33	7	28
Zscan20	119	137	108	117	115	96	151	105
Zscan21	592	562	655	612	557	646	566	540
Zscan22	178	199	177	130	201	153	214	208
Zscan25	429	481	465	387	487	476	486	504
Zscan26	768	904	811	795	817	785	672	760
Zscan29	885	923	889	801	991	952	1010	762
Zscan30	26	37	33	34	27	25	62	21
Zswim1	256	259	274	258	211	218	214	272
Zswim3	206	193	236	228	233	237	192	228
Zswim4	671	628	697	650	639	592	607	658
Zswim5	1453	1639	1349	1184	1629	1649	1689	1350
Zswim6	318	328	365	365	378	302	257	318
Zswim7	336	342	313	269	280	345	279	315
Zswim8	1956	2023	2093	1940	1742	1883	1759	1796
Zswim9	71	46	71	66	31	49	50	53
Zup1	452	546	527	523	505	622	494	451
Zw10	650	677	649	691	662	674	657	646
Zwilch	338	373	368	352	470	334	270	365
Zwint	908	1032	967	1071	965	1095	966	936
Zxdb	139	179	104	134	128	153	172	151
Zxdc	671	881	668	711	860	884	874	919
Zyg11a	211	215	216	203	275	203	272	165
Zyg11b	2590	2821	2493	2549	2971	2837	2734	2607
Zyx	5005	5062	4967	4922	4913	5155	5197	4990
Zzef1	12299	13270	12321	11799	12485	13348	13066	11914
Zzz3	733	813	770	817	835	885	863	820
a	12	1	4	6	0	4	1	1
ccdc198	1	15	5	1	3	6	4	6

Transcriptome sequencing yielded total genetic results for the MOD and APS groups, with a total of 15,936 variables
